# Deep sequencing and genome-wide analysis reveals the expansion of MicroRNA genes in the gall midge *Mayetiola destructor*

**DOI:** 10.1186/1471-2164-14-187

**Published:** 2013-03-18

**Authors:** Chitvan Khajuria, Christie E Williams, Mustapha El Bouhssini, R Jeff Whitworth, Stephen Richards, Jeffrey J Stuart, Ming-Shun Chen

**Affiliations:** 1 Department of Entomology, Kansas State University, Manhattan, Kansas, 66056, USA; 2 USDA-ARS and Department of Entomology, Purdue University, West Lafayette, IN, 47097, USA; 3 International Center for Agricultural Research in the Dry Area, Aleppo, Syria; 4 Department of Molecular and Human Genetics, Human Genome Sequencing Center, Baylor College of Medicine, Houston, Texas, USA; 5 Department of Entomology, Purdue University, West Lafayette, IN, 47097, USA; 6 Hard Winter Wheat Genetics Research Unit, USDA-ARS, 4008 Throckmorton, Kansas State University, Manhattan, KS, 66506, USA; 7 Current Address: Department of Entomology, University of Nebraska, Lincoln, NE, 68583, USA

## Abstract

**Background:**

MicroRNAs (miRNAs) are small non-coding RNAs that play critical roles in regulating post transcriptional gene expression. Gall midges encompass a large group of insects that are of economic importance and also possess fascinating biological traits. The gall midge *Mayetiola destructor*, commonly known as the Hessian fly, is a destructive pest of wheat and model organism for studying gall midge biology and insect – host plant interactions.

**Results:**

In this study, we systematically analyzed miRNAs from the Hessian fly. Deep-sequencing a Hessian fly larval transcriptome led to the identification of 89 miRNA species that are either identical or very similar to known miRNAs from other insects, and 184 novel miRNAs that have not been reported from other species. A genome-wide search through a draft Hessian fly genome sequence identified a total of 611 putative miRNA-encoding genes based on sequence similarity and the existence of a stem-loop structure for miRNA precursors. Analysis of the 611 putative genes revealed a striking feature: the dramatic expansion of several miRNA gene families. The largest family contained 91 genes that encoded 20 different miRNAs. Microarray analyses revealed the expression of miRNA genes was strictly regulated during Hessian fly larval development and abundance of many miRNA genes were affected by host genotypes.

**Conclusion:**

The identification of a large number of miRNAs for the first time from a gall midge provides a foundation for further studies of miRNA functions in gall midge biology and behavior. The dramatic expansion of identical or similar miRNAs provides a unique system to study functional relations among miRNA iso-genes as well as changes in sequence specificity due to small changes in miRNAs and in their mRNA targets. These results may also facilitate the identification of miRNA genes for potential pest control through transgenic approaches.

## Background

MicroRNAs (miRNA) are small (~22 nucleotides long), non-coding RNAs that regulate gene expression post-transcriptionally by complementarily pairing to mRNAs of protein-coding genes, resulting in either translational suppression or degradation of targeted mRNAs [1,2]. miRNAs are produced through multiple mechanisms. Some miRNAs are produced by processing independent transcripts of individual miRNA-coding genes, whereas others are derived from co-transcripts of genes encoding polycistronic miRNAs [3,4]. In addition, miRNAs can also be produced by processing introns of protein-coding genes [5]. Since the first report in 1993, identification of new miRNAs has been advancing rapidly [6]. By the end of July 2012, over 25,141 miRNA sequences have been deposited to the database miRBase [7]. miRNA genes are estimated to represent up to 1% of total genes in a eukaryotic genome, and therefore, database sequences may represent only a small portion of existing miRNA genes [8]. Among the known miRNAs, some are evolutionally conserved among different organisms, whereas others are species-specific. Conserved miRNAs are likely to regulate common genes with basic functions, whereas species-specific miRNAs may be involved in traits associated with individual species. miRNAs are reported to be involved in cell differentiation, metabolism, disease development, immunological defense, and stress response [9-13]. Through regulating the expression levels of different types of genes, miRNAs may be implicated in every biological process.

The gall midge *Mayetiola destructor*, commonly known as the Hessian fly, is a member of the Cecidomyiidae, one of the largest families within the order of Diptera [14]. Hessian fly is a destructive pest of wheat and may cause an average of 5% annual loss in wheat production in the US [15]. The insect possesses many interesting biological traits, including its ability to manipulate the development of its host plants [16], fast adaption to host resistance [17,18], genomic imprinting [14,19] and extensive E-chromosome elimination [14,20]. A single Hessian fly larva can effectively convert a whole host plant into a gall by inducing formation of nutritive cells at the feeding site, irreversibly inhibiting plant growth, and suppressing plant defense [16,21-23]. At the population level, Hessian fly can rapidly adapt to changes in host plants and defeat plant resistance mediated by resistance genes within as short as three years [24]. It is likely that miRNAs are involved in some of these traits associated with Hessian fly since miRNAs are involved in regulating a wide range of genes [9-13]. The Hessian fly is also becoming a model organism for studying plant – insect interactions due to its well-characterized genetics, availability of genome sequence, and ease of culture in the laboratory [25]. Identification of miRNAs and examination of their function may reveal the potential roles of miRNA in Hessian fly biology and host interactions, and provide a foundation for studying miRNA involvement in gall midge biology in general.

Multiple approaches can be adapted for identification of miRNAs. Computational prediction of potential miRNA-encoding genes can be made from genome sequences based on conservation of mature miRNAs and the characteristic ‘hairpin’ structures of their precursors [8,26]. Predicted miRNAs can be further validated by expression methods such as microarrays and PCR analysis. miRNAs can also be identified directly by deep sequencing miRNA libraries constructed from isolated small-RNA samples. Because miRNAs regulate gene expression by direct pairing, potential regulatory targets can also be predicted computationally by searching for complementary sequence similarity [26,27]. The objectives of this study are to use a combination of deep sequencing of Hessian fly larval miRNA transcriptomes and computational prediction to systematically identify miRNA species in the Hessian fly genome and to identify specific miRNAs that might be involved in insect – plant interactions.

## Results

### Identification of miRNAs by deep sequencing the miRNA transcriptome

The first instar larva is the life stage of Hessian fly that determines the outcome of an infestation, resulting in the host plant becoming resistant or susceptible [25]. To identify miRNAs likely to be involved in establishment of the interaction between Hessian fly larvae and their host, total RNA was isolated from first instar larvae. Small RNAs, with 15 – 50 nucleotides long, were size-selected through gel electrophoresis, and were used for library construction. The libraries were then sequenced on an Illumina GAIIx system (see Methods). A total of 15,749,022 reads were obtained. The raw data are available at the National Center for Biotechnology Information (NCBI) short-read archive (accession number SRX213994). The raw sequences were processed through several steps of filtering to remove: 1) low quality sequences (sequences with ≥80% A, T, G, or C; with ≥3 Ns; or consisting of only A and C, or of only T and G), which represented 0.8% of the raw sequences; 2) sequences with copy number <3 and sizes either <15 or >26 nucleotides, which represented 22.3% of high quality sequences; and 3) other non-coding RNAs including rRNA, tRNA, snRNA, and snoRNA, as well as degraded mRNA species, which represented 27% of the processed sequences (Additional file 1: Figure S1).

**Figure 1:**
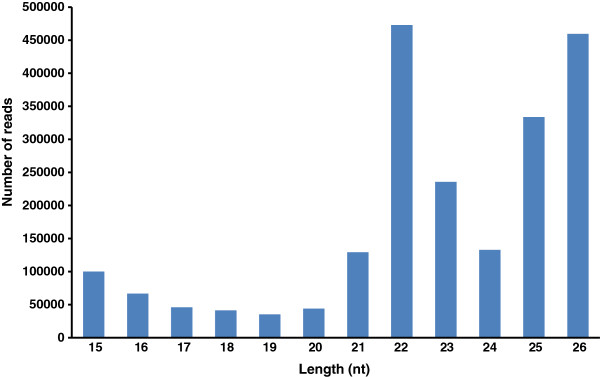
**Size distribution of miRNA candidates identified by deep sequencing of a Hessian fly larval miRNA transcriptome.** The predominant forms were 22 and 26 bp, representing 22.5 and 21.9% of the miRNA species, respectively.

The resulting 2,098,391 sequences were kept as candidates for miRNA analysis. The size distribution of miRNA candidate sequences is shown in Figure 1. The majority (84%) of the sequences were 21 to 26 nucleotides long with 22 and 26 groups as the predominant species, each representing 22.5 and 21.9%, respectively. The high proportion of 26 nucleotide sequences was characteristic of the Hessian fly larval miRNA transcriptome, which was different from similar studies with other organisms [28]. After clustering miRNA candidate sequences based on sequence similarity, 52,928 unique sequences were obtained, which represented 273 miRNA families (Additional file 2: Table S1). Among the 273 families, 89 were similar or identical to existing miRNAs in miRBase, whereas the remaining 184 were novel miRNAs. The 89 similar miRNAs had an average frequency of 5480, whereas the 184 novel miRNAs had an average frequency of 88 (Additional file 2: Table S1).

### Genome-wide survey of potential miRNA-encoding genes

**Figure 2:**
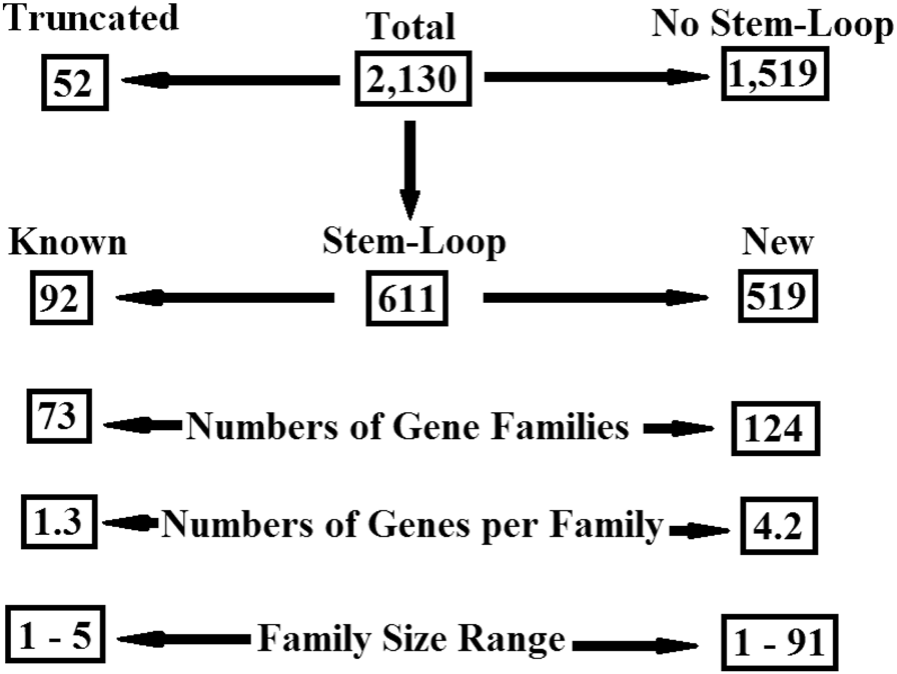
**Results of miRNA prediction by sequence similarity and the existence of a stem-loop structure based on the draft Hessian fly genome sequence.** A total of 2,130 loci were found to contain sequences identical or containing at most 3 mismatches differing from either known miRNAs from other insect species or Hessian fly miRNAs newly identified through deep sequencing in this research. Of the 2,130 sequences, 1,519 contained no flanking sequences that can form a stem-loop structure, and 52 sequences were truncated at ether the 5^′^- or 3^′^-end. The remaining 611 sequences were predicted to form a stem-loop structure that is characteristic of a pre-miRNA species, and therefore were likely to encode miRNAs. Of the 611 putative miRNAs, 92 were identical or very similar to known miRNAs from other insect species, and represented 73 families. The families averaged 1.3 genes with the largest family containing 5 members. The 519 newly identified putative miRNA genes represented 124 families. The families averaged 4.2 members, and the largest family contained 91 members.

To systematically identify orthologs of other known insect miRNAs in the Hessian fly genome and paralogs of the new miRNAs identified through deep sequencing, a total of 2,919 insect mature miRNA sequences were downloaded from miRBase [7]. After removing identical sequences, 1002 unique insect miRNAs were obtained. These unique insect miRNA sequences and the newly identified Hessian fly miRNAs were used to search a draft Hessian fly genome sequence to identify potential miRNA-encoding genes using a locally developed algorithm [29]. The parameters of the algorithm were set so that two mismatches were allowed for miRNA sequences with 22 nucleotides or less and three mismatches were allowed for miRNA sequences with more than 22 nucleotides. Under these criteria, a total of 2,130 loci were identified in the Hessian fly genome sequence from both DNA strands. A DNA sequence from each locus, containing a putative mature miRNA coding region and additional flanking sequences of 140 nucleotides from both the 5^′^- and 3^′^- regions was extracted from the genome sequence, and used to determine the existence of a stem-loop pre-miRNA structure using Mfold [30]. The results are shown in Figure 2 and Additional file 3: Figure S2. Of the 2,130 loci, 611 have extended sequence that can form a stem-loop structure characteristic of miRNA (Additional file 3: Figure S2). Among the 611 putative miRNA genes identified in the Hessian fly genome, only 92 genes were found to match with known mature miRNA from other insects; the remaining 519 (84.9%) putative miRNA genes encode novel miRNAs. The 92 miRNA genes corresponding to known insect miRNAs contained 73 different miRNA families with an average of 1.3 members per family (a miRNA family was defined as identical miRNAs or similar miRNAs with three or fewer mismatches). Strikingly, the 519 miRNA genes corresponding to newly identified Hessian fly miRNAs contained only 124 families, with an average of 4.2 members per family. The relatively larger number of genes per family of newly identified miRNAs was due to the existence of several expanded families. The three largest families, PC-5p-57811, PC-3p-54311, and PC-5p-67443 contained 60, 66, and 91 different genes, respectively (Additional file 3: Figure S2).

### Different miRNAs exhibited different levels of abundance in Hessian fly larvae

**Figure 3:**
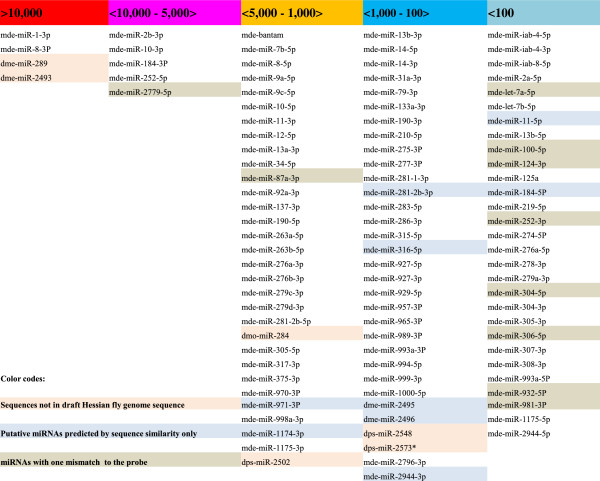
**Relative abundance of miRNAs in Hessian fly larvae based on microarray analysis.** Microarray hybridization signal intensity is given on the top.

To examine the expression of the identified miRNA genes in Hessian fly larvae, we took advantage of an existing microarray that contained probes corresponding to 536 known insect miRNA (Additional file 4: Table S2). The raw data of the microarray analysis were deposited in NCBI database with accession number GSE43680. Sequence analysis revealed that 83 probe sequences matched perfectly and 10 probes had one mismatch with identified Hessian fly miRNAs (Additional file 4: Table S2). To examine the expression levels of different miRNAs, microarray analysis was conducted with RNA samples extracted from first instar larvae that fed on susceptible plants for one or three days. Validation by real-time PCR (qPCR) was carried out with selected transcripts (see below). Overall, only a few probes detected high levels of hybridization signal (signal intensity above 5,000), about one-third detected intermediate levels of hybridization signal (5,000 to 1,000), one-third detected low levels of hybridization signal (1,000 to 100), and one-third detected essentially no signal (below 100) (Figure 3). Probes corresponding to mde-miR-1-3p and mde-miR-8-3p detected the highest levels of hybridization signal (over 10,000). Interestingly, probes corresponding to *Drosophila* miRNAs dme-miR289 and dme-miR-2493, for which the corresponding Hessian fly miRNAs have not yet been identified, also detected the same high levels of hybridization signal. Probes corresponding to mde-miR-2b-3p, mde-miR-10-3p, mde-miR-184-3p, mde-miR-252-5p, and mde-miR-2779-5p also detected relatively high levels of hybridization (10,000 to 5,000).

### Abundance of miRNAs changes at different larval growth stages

Several miRNAs exhibited significant differences in abundance between one- and three-day old larvae (Table 1). Some miRNAs were less abundant in three-day Hessian fly larvae compared with one-day larvae, whereas others were more abundant in three-day larvae than in one-day larvae. The miRNAs mde-miR-10-5p, mde-miR-137-3p, mde-miR-190, and the one corresponding to dmo-miR-284 were relatively abundant in one-day larvae. The abundance of these four miRNAs decreased more than two fold in three-day Hessian fly larvae. On the other hand, the abundance of miRNAs mde-miR-305-5p, mde-miR-9c-5p, and the one corresponding to dme-miR-289 increased more than two fold in three-day Hessian fly larvae in comparison with that in one-day larvae.

**Table 1:** Growth stage variation in miRNA abundance

**miRNA**	**Hybridization-signal intensity (average ± standard deviation)**		**Fold change**
	**1-day larvae**	**3-day larvae**	
**Abundance decreases with time**			
mde-miR-87a-3p	2485 ± 389	1706 ± 266	−1.46 ± 0.13
mde-miR-375-3p	4461 ± 466	3461 ± 471	−1.29 ± 0.11
mde-miR-10-5p	2808 ± 366	1273 ± 161	−2.21 ± 0.17
mde-miR-11-3p	1135 ± 200	612 ± 100	−1.85 ± 0.08
mde-miR-34-5p	1860 ± 181	1168 ± 131	−1.59 ± 0.13
mde-miR-1000-5p	943 ± 169	388 ± 98	−2.43 ± 0.19
mde-miR-137-3p	2728 ± 331	1518 ± 228	−1.8 ± 0.05
mde-miR-190-5p	2507 ± 400	701 ± 143	−3.58 ± 0.21
mde-miR-970-3P	3764 ± 501	2821 ± 318	−1.33 ± 0.14
**dmo-miR-284**	**1132 ± 108**	**443 ± 76**	**−2.56 ± 0.22**
mde-miR-316-5p	182 ± 35	12 ± 10	−15.17 ± 3.1
**Abundance increases with time**			
mde-miR-92a-3p	861 ± 100	1294 ± 143	1.5 ± 0.09
mde-miR-276a-3p	2199 ± 279	3536 ± 368	1.61 ± 0.14
mde-miR-305-5p	1305 ± 257	2696 ± 265	2.07 ± 0.17
mde-miR-79-3p	262 ± 51	432 ± 69	1.65 ± 0.12
mde-mir-92b-3p	551 ± 70	928 ± 114	1.68 ± 0.09
mde-miR-9a-5p	952 ± 113	1689 ± 268	1.77 ± 0.13
mde-miR-9c-5p	468 ± 80	1639 ± 208	3.5 ± 0.22
mde-miR-965-3P	190 ± 32	514 ± 58	2.71 ± 0.19
**dme-miR-289**	**5333 ± 403**	**19781 ± 927**	**3.71 ± 0.25**

RNA samples were extracted from 1-day and 3-day old larvae, converted to cRNAs, and hybridized to microarrays. Data were derived from three biological replicates with three duplicates for each replicate. Rows in boldface represent hybridization results of probes produced according to miRNAs from other insect species, to which the corresponding miRNAs from Hessian fly have not yet been identified. *P* ≤ 0.05.

### Host plant genotypes affect miRNA gene expression

**Figure 4:**
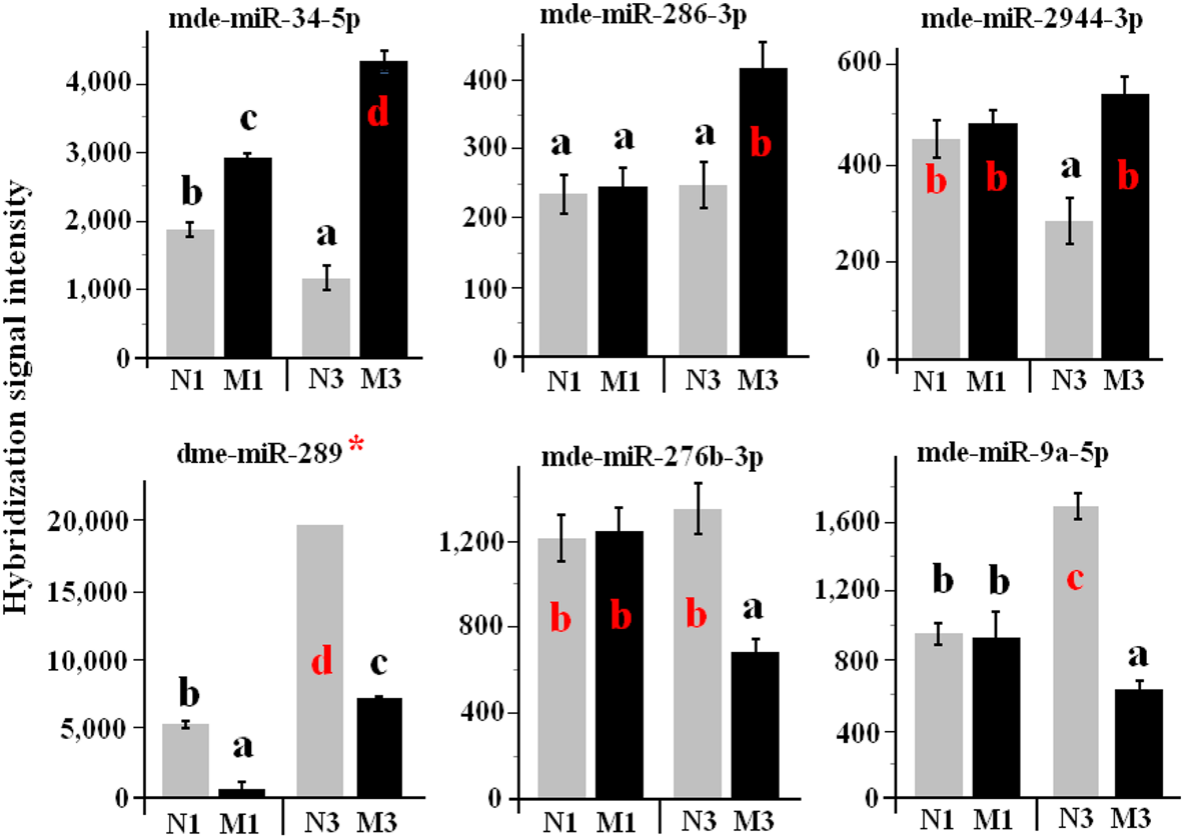
**Abundance of miRNAs affected by host genotypes.** miRNA names are given on the top of each graph. N1, M1, N3, and M3 represent one-day larvae feeding in Newton (a susceptible cultivar) seedlings, one-day larvae in Molly (a resistant cultivar) seedlings, three-day larvae in Newton seedlings, and three day larvae in Molly seedlings, respectively. Three biological replicates were conducted for each assay. The error bar in each graph represents standard error (SE). The small letters in each graph indicate different groups based on t-test with *P ≤ 0.05*. The symbol ‘*’ indicates that the miRNA has not been identified in Hessian fly and the results were based on the ortholog from another insect.

Some miRNAs exhibited very different levels of abundance in Hessian fly larvae depending on the plant genotypes on which they were feeding (Figure 4). Newton and Molly wheat lines are nearly isogenic lines, with Newton the susceptible recurrent parent of Molly, which contains the resistance gene *H13*[31]. A range of different patterns in miRNA abundance were observed in larvae feeding in resistant Molly plants in comparison with those feeding on susceptible Newton plants. The patterns of differences included: 1) Higher miRNA abundance in both one- and three-day old larvae feeding in Molly plants in comparison with larvae feeding in Newton plants during the same period, represented by mde-miR-34-5p (Figure 4), mde-miR-994-5p and mde-miR-263b-5p (Additional file 5: Figure S3); 2) Higher miRNA abundance specifically in three-day larvae feeding in Molly plants, represented by mde-miR-286-3p, mde-miR-2944-3p (Figure 4), mde-miR-210-5p, mde-miR-989-3p, mde-miR-971-3p, mde-miR-11-3p, and mde-miR-87a-3p (Additional file 5: Figure S3); 3) Lower miRNA abundance in both one- and three-day old larvae feeding in Molly plants, represented by the miRNA corresponding to dme-miR-289 (Figure 4) and mde-miR-2502 (Additional file 5: Figure S3); and Additional file 6: Figure S4) Lower miRNA abundance specifically in three-day larvae feeding in Molly plants, represented by mde-miR-276b-3p, mde-miR-9a-5p (Figure 4), and mde-miR-2779-5p (Additional file 5: Figure S3).

## Discussion

Using deep sequencing and homology-based prediction, we identified 611 putative miRNAs and their encoding genes from the gall midge *Mayetiola destructor*, an important member of a large group of insects that have unique biology in terms of insect – host plant interactions [25]. Among the identified miRNAs, 89 are identical or highly similar to known miRNAs from other insect species. These common miRNAs are likely to play common roles in insect growth and development.

In addition, a total of 530 new miRNAs or putative miRNAs were identified in this study. One interesting and so far unique feature of these new miRNAs is the dramatic expansion of several miRNA gene families. The expanded gene families include PC-3P-59454 with nine genes, PC-5p-61169 with 10 genes, PC-3p-19591 with 11 genes, PC-3p-58746 with 13 genes, PC-3p-36826 with 14 genes, PC-5p-66343 with 22 genes, PC-3p-47103 with 24 genes, PC-5p-39989 with 28 genes, PC-5p-57811 with 60 genes, PC-3p-54311 with 66 genes, and PC-5p-67443 with 91 genes. The numbers of genes in these expanded families only included those genes that encode putative pre-miRNAs that can form a typical stem-loop structure with a 140 bp region surrounding the miRNA coding region. There are also many sequences that can form stem-loop structures within a longer sequence range, namely 210 bp regions surrounding the putative miRNA coding region (data not shown). These DNA sequences could also encode miRNAs, but were not included in this report. This expansion phenomenon of gene families was not observed with miRNAs that share high sequence similarity with known miRNAs from other insect species. A literature search revealed the existence of multi-gene families of miRNAs in other species. For example, there are 16 paralogs of miR395 in the maize genome [26]. However, so far the expansion of miRNA genes found in the Hessian fly genome is the most dramatic, with as many as 91 genes in a single family.

**Figure 5:**
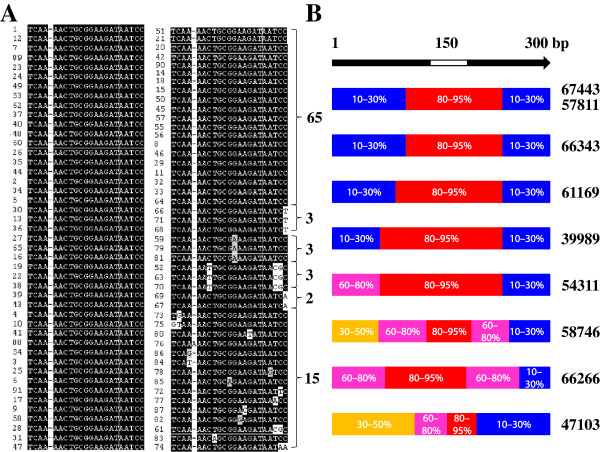
**Sequence conservation of the expanded miRNA gene families. A**: Sequence alignment of 91 putative mature miRNA coding regions of the PC-5p-67443 family. There are 65 sequences identical to the miRNA identified by deep sequencing, three with a base change from C to T at the last residue, three with a base change from G to A at position 13, three with two base changes from TC to CG at positions 21–22 and C to T at position 8, two with a base change from C to A at the last residue, and 15 with single or double changes at various positions. **B**: Schematic presentations of sequence conservation of different regions among genes in different families. The black portion of the arrow on the top of the figure represents the approximately 300 base pairs of the sequence flanking the 150 base pairs of a mature miRNA coding region, which is indicated by the white space in the middle of the arrow. The color rectangles indicate various degrees of conservation/divergence among family members with percentages of sequence identity given within each rectangle. Overall, the coding regions and the immediate surrounding sequences are highly conserved (indicated by red), indicating that the genes are likely functional, but the remaining flanking regions are divergent. The family names (numbers) are given on the right side of the figure.

The biological implication of the dramatic expansion of the Hessian fly-specific miRNAs remains to be determined. Examination of putative miRNA genes within a family revealed that mature miRNA coding regions and the corresponding complementary regions for the stem-loop structure were highly conserved (Figure 5, Additional file 6: Figure S4). For example, 65 out of the 91 members in the PC-5p-57811 family were identical in the miRNA coding region, and the remaining members differed by only one to three residues (Figure 5A). In contrast, the regions surrounding the miRNA coding and stem-loop region exhibited little sequence similarity (Figure 5B, Additional file 6: Figure S4A). Gene members in other expanded families showed similar conservation patterns but some families were less typical (Figure 5, Additional file 6: Figure S4). The conservation in miRNA coding regions and diversification in non-coding regions suggest that these miRNA genes are functional. miRNAs that vary by single or multiple residues, especially in the seed region (position 2–8), can have a different target spectrum and may perform very different regulatory functions since minor changes in sequence could remarkably affect target specificity or regulatory efficiencies. However, the reason remains to be determined for the existence of so many different genes in the Hessian fly genome that encode identical miRNAs. Theoretically, identical products should have identical function. We hypothesize that these iso-miRNA genes with different flanking regions may allow Hessian fly to fine tune the expression levels of the same miRNA product during different developmental stages or under different environments. If that is the case, the existence of so many iso-genes would suggest that the levels of the corresponding miRNA need to be strictly regulated. Further studies will have to be completed to determine the reason for the existence of so many iso-miRNA genes and their regulatory mechanisms.

Another interesting feature of the identified Hessian fly miRNAs is existence of the second peak at 25–26 nucleotides for the length distribution (Figure 1), which has not been reported from other species. Since piRNAs, another type of non-coding RNAs, have a length distribution at around 26–29 nucleotides [32], some of the identified miRNAs with 26 nucleotides may actually be piRNAs. Indeed, all miRNAs with 26 nucleotides have a U residue at position 1, which is characteristic of piRNAs. However, 61% of the 89 miRNAs that are similar to known. This is miRNA from other insects also have a U at position 1; and 46% of Hessian fly-specific, non-26-nucleotide miRNAs have a U at position 1, too. It seems that U is the residue that occurs most frequently in miRNAs of all sizes identified in this study. The genes coding for 26 nucleotide miRNAs are scattered as single locus across the genome, whereas most known piRNA genes are derived from discrete loci. All identified Hessian fly pre-RNA sequences for the miRNAs with 26 nucleotides can form typical stem-loop structures that are observed in pre-miRNAs, but not in pre-piRNAs. Based on these observations, the non-coding RNAs of 26 nucleotides identified in this study are likely miRNAs, not piRNAs. The significance of the existence of a large number of miRNAs with 26 nucleotides specifically in Hessian fly genome remains to be determined.

The expression profiles of different miRNAs varied dramatically in Hessian fly larvae. Some miRNAs, for example mde-miR-1-3p and mde-miR-8-3p, were expressed at very high levels, whereas others, such as mde-miR-iab-4-5p and mde-miR-2a-5p, could not be detected at all in first instar larvae (Figure 2, Additional file 4: Table S2), indicating that miRNA expression is strictly regulated as needed in Hessian fly development. Indeed many miRNAs were stage-regulated (Table 1). For example, the abundance of mde-miR-10-5p, mde-miR-1000-5p, and mde-miR-190-5p in one-day larvae decreased by more than 50% in three-day larvae compared to that in one-day larvae. In contrast, the abundance of mde-miR-305-5p, mde-miR-9c-5p, and mde-miR-965-3p increased more than 50% over the same two-day period. The strict regulation of miRNA abundance in Hessian fly larval development indicates opportunity for utilization of miRNAs in pest control. For example, artificial introduction of miRNAs or analogs that are not expressed in Hessian fly larvae, through a transgenic approach could disrupt Hessian fly larval development, resulting in insect death. Small RNA molecules ectopically expressed in host plants can be ingested by insects and transgenic plants with small interfering RNA have been successfully developed for insect and other pest control [33-35].

The potential application of miRNAs as tools for pest management was also highlighted by the fact that abundance of miRNAs was affected by host plant genotypes (Figure 4, Additional file 5: Figure S3, Additional file 4: Table S2). Some miRNAs such as mde-miR-34-5p and mde-miR-994-5p became more abundant in Hessian fly larvae feeding in resistant plants, whereas some others such as the ones corresponding to mde-miR-289 and dps-miR-2502 became less abundant in insects feeding in the resistant plants. Further research on potential target genes of the miRNAs affected by host genotypes will increase our understanding of miRNAs in Hessian fly developmental control and in host plant resistance mechanism to the insect.

## Conclusions

We have identified and characterized, for the first time, a large number of miRNAs from a gall midge belonging to a large group of insects that are of both economic importance and broad biological interest. The dramatic expansion of some miRNA families that encode the same or very similar miRNAs is an interesting phenomenon and further research on these gene families could shed new light on miRNA expression regulation and their interactions with targeted mRNA species. The distinct expression profiles of miRNA species in Hessian fly larvae suggests that manipulation of miRNA abundance through transgenic plants may provide us opportunities to control this economically important insect pest.

## Methods

### Insect and maintenance

A Hessian fly colony derived from a field collection was used in this study. The field population was collected from Scott County, Kansas in 2005. The majority of the population is biotype GP that is avirulent to known Hessian fly resistance (R) genes, but a small portion of the insects are virulent to several R genes [36]. The insect colony has been maintained on the susceptible wheat cultivar Karl92 in a greenhouse.

### Wheat cultivars and infestation

Two wheat cultivars were used in this study, Newton and Molly. Newton is a susceptible winter wheat that contains no Hessian fly R gene, and Molly is a nearly isogenic line of Newton, but contains the R gene *H13*[31]. Twenty germinated wheat seeds were planted in 10-cm-diameter pots filled with PRO-MIX ‘BX’ potting mix (Hummert Inc., Earth City, MO) in a growth chamber programmed at 20:18°C (L:D) with a photoperiod of 14:10 (L:D) h. When wheat seedlings reached the 1.5 leaf stage (stage 11 on Zadoks scales) [37], the plants were infested with 10 Hessian fly females per pot (0.5 female flies per plant on average) by confining the insects in a screened cage. The infestation condition yielded ~8 larvae per plant.

### Total RNA extraction and small-RNA fractionation

Hessian fly larvae were collected by rinsing dissected plants in a beaker. Total RNA was extracted using a miRNA isolation kit (miRNeasy kit, Qiagen) according to the manufacturer’s instructions. RNA concentration was determined using a Nanodrop *ND-1000* spectrophotometer (NanoDrop Technologies Inc., Wilmington, DE). Quality of the total RNA samples was determined with an Agilent 2100 Bioanalzer (Agilent Technologies, Palo Alto, CA). Total RNA (~200 μg) was size-fractionated on a 15% tris-borate-EDTA-Urea polyacrylamide gel. The RNA fragments of 15–50 nucleotides in length were isolated. The small-RNA fraction was then used for miRNA library construction.

### Small-RNA library construction

A small-RNA library was generated according to Illumina’s sample preparation instructions (Illumina, San Diego, CA). Specifically, the SRA 5^′^ adapter was ligated to 50 ng of the aforementioned RNA fragments with T4 RNA ligase (Promega, Fitchburg, WI). The ligated RNAs were size-fractionated on a 15% tris-borate-EDTA-Urea polyacrylamide gel and the RNA fragments of size ~41-76 nucleotides were eluted from the gel. Then the SRA 3^′^ adapter was ligated following the same procedure. A second size-fractionation was carried out under the same gel conditions. The RNA fragments of size ~64-99 nucleotides were isolated through gel elution and ethanol precipitation.

The ligated RNA fragments were reverse-transcribed to produce single-stranded cDNAs using M-MLV reverse transcriptase (Invitrogen, Carlsbad, CA) with Illumina RT-primer sets. The cDNAs were amplified with PFX DNA polymerase (Invitrogen) in 20 cycles of PCR using Illumina’s small RNA primers sets. The PCR products were purified on a 12% TBE polyacrylamide gel and a slice of gel containing amplicons of ~80-115 bps was excised. This fraction was eluted and the recovered cDNAs were precipitated before quantification on both the Nanodrop and on TBS-380 mini-fluorometer (Turner Biosystems) using Picogreen® dsDNA quantification reagent (Invitrogen). The concentration of each sample was adjusted to 10 nM and a total of 10 μl was used in sequencing reactions.

### miRNA deep sequencing

The purified cDNA library was used for cluster generation on Illumina’s Cluster Station and then sequenced on an Illumina GAIIx following vendor’s instructions for running the instrument. Raw sequencing reads were obtained using Illumina’s Pipeline v1.5 software following sequencing image analysis by the Pipeline Firecrest Module and base-calling by the Pipeline Bustard Module. The extracted sequencing reads were then used in standard data analysis (see below).

### Sequence data analysis

A proprietary software package, ACGT101-miR v3.5 (LC Sciences, Huston, TX), was used for analyzing sequencing data. After the raw sequence reads were extracted from image data, low quality reads, copy number less than 3 and length less than 15 nucleotides or larger than 26 nucleotides, adapter sequences, and junk sequences (such as ≥80% A, C, G or T; ≥3 Ns; only A, C, or only G, T) were removed from further analysis. In addition, the sequences mapping to the mRNA databases, RFam (release 9.1) and Repbase (version 15.07) were also removed. The remaining sequences were used to BLAST against the miRbase database to identify known miRNAs (mismatch: <2 bases; E-values: <0.005) (73, 74). The remaining sequences not matching known miRNAs were mapped to the Hessian fly genome draft sequence to identify potentially novel miRNAs. Novel miRNAs were predicted if the extended sequences at the mapped positions were predicted to form hairpin structures.

### Microarray analysis

Hessian fly larvae were reared on the Molly and Newton plants as mentioned above. Larvae were collected one and three days after egg hatch from susceptible Newton and resistant Molly plants. Total RNA was extracted as mentioned above. Quality of the total RNA samples was analyzed with an Agilent 2100 Bioanalyzer (Agilent Technologies, Palo Alto, CA). Three biological replications were used for each treatment and at each time point. For the microarray assay 0.2 μg of total RNA was used. The RNA samples were first subjected to dephosphorylation to remove 5^′^ phosphate groups from miRNA molecules. Then an oligonucleotide adapter was added to the 3^′^ end of sample (3^′^OH containing) RNA sequences using T4 RNA ligase. The adapter sequence contains a tag segment for capturing fluorescent dye during a later dye staining process. Hybridization was performed on a μParaflo™ microfluidic chip using a micro-circulation pump. Inside the microfluidic chip, each detection probe consisted of a chemically modified nucleotide-coding segment complementary to target microRNA (from miRBase) or other RNA (control or customer defined sequences). The coding segments of the probe molecules are extended away from the corresponding substrate surface by polyethylene glycol spacers, which reduce steric hindrance and surface charge effects during hybridization. The probes were synthesized *in situ* using a photogenerated reagent (PGR). Probe/target pair melting temperatures were balanced by chemical modifications and sequence length adjustment to the coding segments of the probes. Hybridization reactions were carried out in 100 μL 6xSSPE buffer (0.90 M NaCl, 60 mM Na_2_HPO_4_, 6 mM EDTA, 25% formamide, pH 6.8) at 40°C overnight. Then the chips were stained using a tag-specific fluorescence dye (Af3 from Invitrogen). Hybridization images were collected using a laser scanner (GenePix 4000B, Molecular Devices) and digitized using Array-Pro image analysis software (Media Cybernetics). Data were analyzed by first subtracting background and then normalizing signals using a LOWESS filter (Locally-weighted Regression). *P*-values of the t-test were calculated and signals were considered differentially detected if the *p*-values were less than 0.05.

### Real-time PCR analysis

Total RNA was extracted using a miRNA isolation kit (miRNeasy kit, Qiagen) according to the manufacturer’s instructions. RNA concentration was determined using a Nanodrop *ND-1000* spectrophotometer (NanoDrop Technologies Inc., Wilmington, DE). One μg of total RNA was used for synthesis of the first strand cDNA using the NCode™ VILO™ miRNA cDNA Synthesis Kit (Invitrogen). One μl of the cDNA sample was used as a template for real-time quantitative PCR (qPCR). Three biological replications, each with two technical replications were used for qPCR analysis. qPCR primers were designed following the recommendation provided in the manual of the NCode™ VILO™ miRNA cDNA Synthesis Kit. The forward primers (mde-1-3p: GGCTGGAATGTAAAGAAGTATGGAG and der-miR-263a-5p: CACTGGAAGAATTCACGGGA) were used along with the universal qPCR Primer provided in the kit. The qPCR analysis was performed using a SYBR green kit (Bio-Rad) and Bio-Rad iCycler iQ5 real-time PCR detection system at the Kansas State University Gene Expression Facility. The expression of the *U6* small nuclear RNA gene was used as an internal control [38]. The forward and reverse primers for the *U6* gene used are TGGAACGCTTCACGATTTT and TTGGAACGATACAGAGAAGATTAGC respectively. qPCR cycling parameters included 95°C for 5 min, 40 cycles each consisting of 95°C for 15 sec, 55°C for 15 sec, and 72°C for 30 sec. At the end of each PCR reaction, a melting curve was generated to confirm single peak and rule out non-specific product formation. Data was subjected to one-way analysis of variance (ANOVA) and fisher’s least significant difference (LSD) multiple comparisons were used to identify the significant difference between the treatments. All the statistical analyses were performed using ProStat software (Poly Software International Inc., Pearl River, NY, USA). The results of qPCR are shown in Additional file 7: Figure S5.

## Competing interest

The authors declare that they have no competing interests.

## Authors’ contributions

CK performed experiments and analyzed data, CEW contributed reagents and edited the manuscript, MEB analyzed data and edited the manuscript, RJW contributed reagents and edited the manuscript, JJS, analyzed data and edited the manuscript, MSC and CK designed the experiment and wrote the paper. All authors read and approved the final manuscript.

## Supplementary Material

Additional file 1: Figure S1 Sequence data filtering and database mapping. A total of 15,749,022 reads were initially processed by removing low quality sequence reads. The remaining 15,623,905 high quality sequences were further processed by removing sequences shorter than 15 bp and longer than 26 bp, and sequences with copy numbers smaller than three. Finally, other non-coding RNAs including rRNAs, tRNAs, snRNAs, snoRNAs, and degraded mRNAs were removed by blasting various related databases. The resulting 2,098,391 sequences were kept as candidates for miRNA analysis. Click here for file

Additional file 2: Table S1 miRNAs identified from Hessian fly larvae by deep sequencing. Click here for file

Additional file 3: Figure S2 Name, sequence, identification method, and loop-structure of 530 novel and 126 known miRNAs. The region of miRNA in the stem-loop structure is bold and in red color. Click here for file

Additional file 4: Table S2 Microarray targeted Hessian fly miRNAs, probe name, microarray hybridization data, and probe sequence. Newton is a susceptible wheat cultivar. Molly is a resistant cultivar that contains Hessian fly resistance gene H13. RNA samples were obtained from 1- and 3-days old larvae feeding in Newton and Molly, respectively. Three biological replicates were conducted and each replicate had three duplicates. Click here for file

Additional file 5: Figure S3 Abundance of miRNAs affected by host genotypes. miRNA names are given on the top of each graph. N1, M1, N3, and M3 represent one day larvae feeding in Newton (a susceptible cultivar) seedlings, one day larvae in Molly (a resistant cultivar) seedlings, three day larvae in Newton seedlings, and three day larvae in Molly seedlings, respectively. The small letters in each graph indicate different groups based on statistical analysis. Click here for file

Additional file 6: Figure S4 Nucleotide sequence alignments of regions surround miRNA coding regions. The miRNA coding regions and 5’- or 3’-complementary regions are marked with signs “├starts and ┤ends”. Click here for file

Additional file 7: Figure S5 qPCR validation of microarray data and further analysis of miRNA levels in other developmental stages of Hessian fly. Samples were collected from several life stages of Hessian fly reared on Newton plants, as well as, from larvae reared on Newton and Molly plants. The expression of the U6 small nuclear RNA gene was used as an internal control. Three biological replications and two technical replications were used in this analysis. The bars with and asterik (*) were used for qPCR and microarray data comparison. Egg 1 and 3: one and three days old eggs. ML1 and ML3: larvae collected after one and three day of incompatible interaction (reared on Molly). NL1, NL3, and NL8: larvae collected after one, three, and eight days of compatible interaction (reared on Newton). Click here for file
