# Supplementary material for: Deep sequencing and genome-wide analysis reveals the expansion of MicroRNA genes in the gall midge Mayetiola destructor
Source: BMC Genomics. 2013 Mar 18;14:187. doi: 10.1186/1471-2164-14-187 (PMC3608969; doi:10.1186/1471-2164-14-187)
Supplement: Additional file 1: Figure S1 — Sequence data filtering and database mapping. A total of 15,749,022 reads were initially processed by removing low quality sequence reads. The remaining 15,623,905 high quality sequences were further processed by removing sequences shorter than 15 bp and longer than 26 bp, and sequences with copy numbers smaller than three. Finally, other non-coding RNAs including rRNAs, tRNAs, snRNAs, snoRNAs, and degraded mRNAs were removed by blasting various related databases. The resulting 2,098,391 sequences were kept as candidates for miRNA analysis. [file 1471-2164-14-187-S1.pptx]

## Slide 1
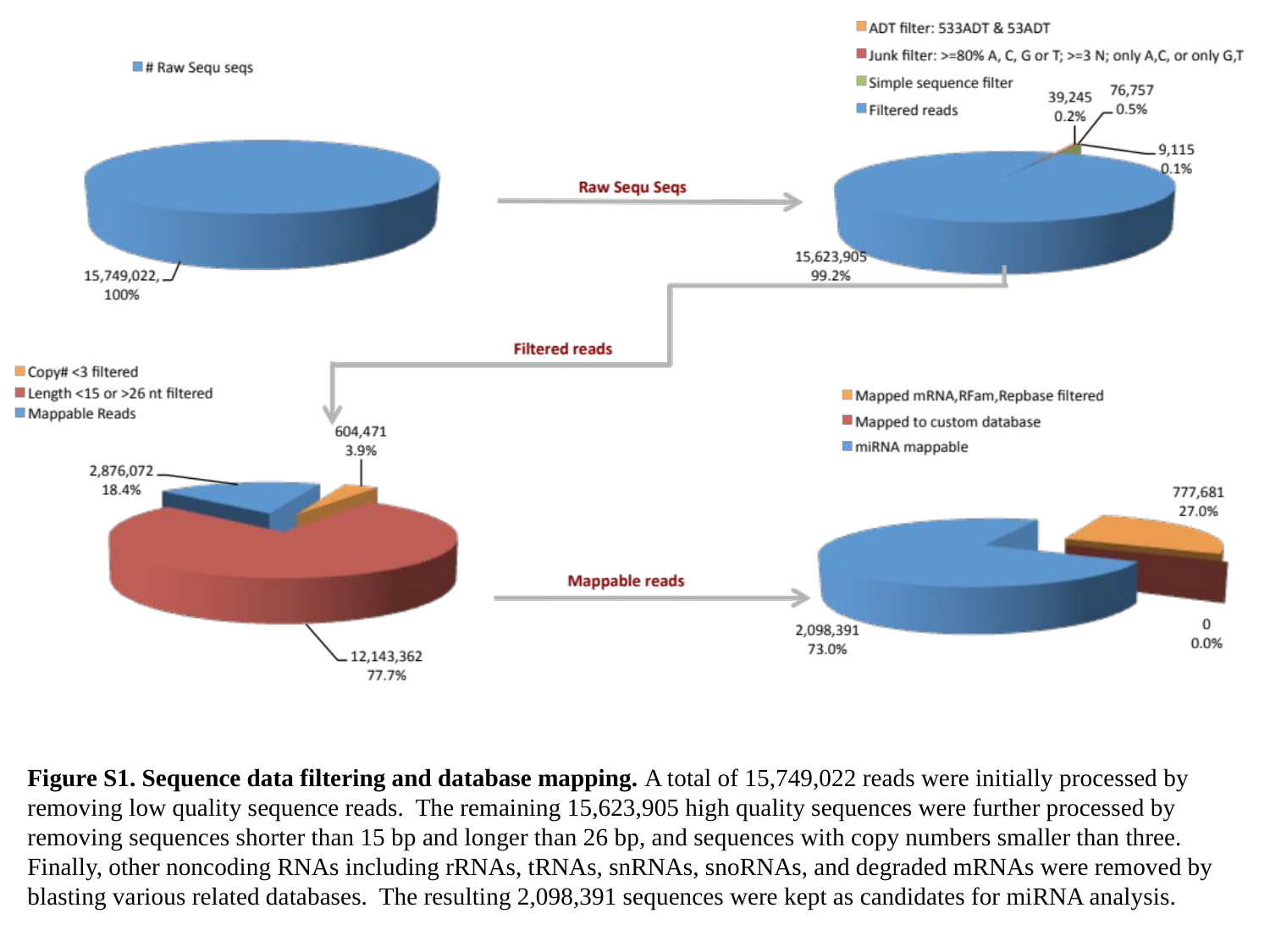

Figure S1. Sequence data filtering and database mapping. A total of 15,749,022 reads were initially processed by removing low quality sequence reads. The remaining 15,623,905 high quality sequences were further processed by removing sequences shorter than 15 bp and longer than 26 bp, and sequences with copy numbers smaller than three. Finally, other noncoding RNAs including rRNAs, tRNAs, snRNAs, snoRNAs, and degraded mRNAs were removed by blasting various related databases. The resulting 2,098,391 sequences were kept as candidates for miRNA analysis.
