# Supplementary material for: Deep sequencing and genome-wide analysis reveals the expansion of MicroRNA genes in the gall midge Mayetiola destructor
Source: BMC Genomics. 2013 Mar 18;14:187. doi: 10.1186/1471-2164-14-187 (PMC3608969; doi:10.1186/1471-2164-14-187)
Supplement: Additional file 2: Table S1 — miRNAs identified from Hessian fly larvae by deep sequencing. [file 1471-2164-14-187-S2.doc]

**Table S1. miRNAs identified from Hessian fly larvae by deep-sequencing.**

| miR_name | Sequence | | Length | | | copies of isoforms | | Homolog from other insects |
| --- | --- | --- | --- | --- | --- | --- | --- | --- |
|  | | |  | |  |  | |  |
| **miRNAs similar/identical to known miRNA (89 unique plus 15 variants)** | | | |  |  |  | |  |
| mde-miR-iab-4-3p | CGGTATACCTTCAGTATACGTA | 22 | | | | | 30 | dme-mir-iab-4 |
| mde-miR-iab-4-5p | ACGTATACTGAATGTATCCTGA | 22 | | | | | 1017 | dme-mir-iab-4 |
| mde-miR-iab-8-5p | TTACGTATACTGAAGGTATACCG | 23 | | | | | 7 | dme-mir-iab-4as |
| mde-miR-252-3p | CCTGCTGCCCAAGTGCTTATCA | 22 | | | | | 4 | aae-mir-252 |
| mde-miR-957-3p | TGAAACCGTCCAAAACTGAGGC | 22 | | | | | 116 | dme-mir-957 |
| mde-miR-100-5p | AACCCGTAGATCCGAACTTGT | 21 | | | | | 4 | bmo-mir-100 |
| mde-miR-285-5p | TAGCACCATTCGAAATCAGTCC | 22 | | | | | 5 | dme-mir-285 |
| mde-miR-2944-5p | AAGGAACTCCCGGTGTGATAT | 21 | | | | | 97 | aae-mir-2944b |
| mde-miR-13a-3p | TATCACAGCCATTTGATGAGCT | 22 | | | | | 255 | dme-mir-13a |
| mde-miR-2779-3p | TCCGGTTCGAAGGACCAT | 18 | | | | | 64 | bmo-mir-2779 |
| mde-miR-927-5p | TTTAGAATTCCTACGCTTTATC | 22 | | | | | 435 | dme-mir-927 |
| mde-miR-133a-3p | TTGGTCCCCTTCAACCAGCTGT | 22 | | | | | 29 | dme-mir-133 |
| mde-miR-79-3p | TAAAGCTAGATTACCAAAGCAT | 22 | | | | | 161 | dme-mir-79 |
| mde-miR-993a-5P | TACCCTGTAGTTCCGGGCTTTT | 22 | | | | | 1838 | dme-mir-993 |
| mde-miR-1175-5p | AAGTGGAGCAGTGGTCTCATCG | 22 | | | | | 185 | aae-mir-1175 |
| mde-miR-278-3p | TCGGTGGGACTTTCGTCCGTTT | 22 | | | | | 31 | dme-mir-278 |
| mde-miR-31a-5p | TGGCAAGATGTCGGCATAGCT | 21 | | | | | 800 | dme-mir-31a |
| mde-miR-307-3p | CACAACCTCCTTGAGTGAGCGA | 22 | | | | | 4 | dme-mir-307 |
| mde-miR-252-5p | CTAAGTACTAGTGCCGCAGGAGT | 23 | | | | | 4 | dme-mir-252 |
| mde-miR-252-5p variant | CTAAGTACTAGTGCCGCAGGAG | 22 | | | | | 636 | aae-mir-252 |
| mde-miR-87a-3p | GTGAGCAAATTTTCAGGTGTGT | 22 | | | | | 174 | aae-mir-87 |
| mde-miR-219-5p | TGATTGTCCAAACGCAATTCTTG | 23 | | | | | 31 | dme-mir-219 |
| mde-miR-306-5p | TCAGGTACTGAGTGACTCTCAG | 22 | | | | | 38 | aae-mir-306 |
| mde-miR-2a-3p | TATCACAGCCAGCTTTGATGAGT | 23 | | | | | 3 | bmo-mir-2b |
| mde-miR-2a-3p variant | TATCACAGCCAGCTTTGA | 18 | | | | | 3 | dme-mir-2b-2 |
| mde-miR-2b-3p | TATCACAGCCAGCTTTGATGAGCT | 24 | | | | | 1577 | dme-mir-2a-2 |
| mde-miR-2b-3p variant | TATCACAGCCAGCTTTGATG | 20 | | | | | 30 | dme-mir-2c |
| mde-miR-2c-3p | TATCACAGCCAGCTTTGAAGAG | 22 | | | | | 1049 | aae-mir-2a |
| mde-miR-2838 | TACGGGTTGAAATGGTTTAAATTCAG | 26 | | | | | 4 | bmo-mir-2838 |
| mde-miR-34-5p | TGGCAGTGTGGTTAGCTGGTTGT | 23 | | | | | 1122 | dme-mir-34 |
| mde-miR-190-3p | CCCAGGAATCAAACATATTATTA | 23 | | | | | 16 | dme-mir-190 |
| mde-miR-9e-5p | TCTTTGGTATTCTAGCTGTAGA | 22 | | | | | 247 | dme-mir-9c |
| mde-miR-375-3p | TTTGTTCGTTTGGCTTGAGTTAA | 23 | | | | | 598 |  |
| mde-miR-375-3p variant | TTTGTTCGTTTGGCTTGAGTTA | 22 | | | | | 10333 | aae-mir-375 |
| mde-miR-1-3p | TGGAATGTAAAGAAGTATGGAG | 22 | | | | | 313172 | dme-mir-1 |
| mde-miR-184-3P | TGGACGGAGAACTGATAAGGGC | 22 | | | | | 4362 | dps-mir-184 |
| mde-miR-125a | TAATCCCTGAGACCCTAACTTGTGA | 25 | | | | | 3 | tca-mir-125 |
| mde-miR-281-2a-5p | AAGAGAGCTATCCGTCAACAG | 21 | | | | | 3 | dpu-mir-281 |
| mde-miR-999-3p | TGTTAACTGTAAGACTGTGTCT | 22 | | | | | 941 | dme-mir-999 |
| mde-miR-998b | TAGCACCATGAGATTCAGCTCA | 22 | | | | | 5 | bmo-mir-998 |
| mde-miR-305-3p | CGGCACGTGTTGGAGTACACT | 21 | | | | | 23 | aae-mir-305 |
| mde-miR-8-3P | TAATACTGTCAGGTAAAGATGTC | 23 | | | | | 4703 | dme-mir-8 |
| mde-miR-13b-3p | TATCACAGCCATTTTGACGAGTTG | 24 | | | | | 4 | nvi-mir-13b |
| mde-miR-13b-3p variant | TATCACAGCCATTTTGACGAGTT | 23 | | | | | 1255 | dme-mir-13b-1 |
| mde-miR-1000-5p | ATATTGTCCTGTCACAGCATTA | 22 | | | | | 1212 | dme-mir-1000 |
| mde-miR-1000-5p variant | ATATTGTCCTGTCACAGCATTAG | 23 | | | | | 69 |  |
| mde-miR-92a-3p | TATTGCACTTGTCCCGGCCTATT | 23 | | | | | 45 | tca-mir-92a |
| mde-miR-92a-3p variant | TATTGCACTTGTCCCGGCCTAT | 22 | | | | | 749 | dme-mir-92a |
| mde-miR-9e-3p | TAAAGCTTTAATACCAGAGGTC | 22 | | | | | 26 | cqu-mir-9 |
| mde-miR-993a-3P | GAAGCTCGTCTCTACAGGTATCT | 23 | | | | | 3508 | dme-mir-993 |
| mde-miR-281-2b-5p | AAGAGAGCTATCCGTCGACAGT | 22 | | | | | 2380 | cqu-mir-281 |
| mde-miR-276a-3p | TAGGAACTTCATACCGTGCTCT | 22 | | | | | 12979 | aae-mir-276-2 |
| mde-miR-276b-3p | TAGGAACTTAATACCGTGCTCT | 22 | | | | | 3 | dme-mir-276b |
| mde-miR-137-3p | TATTGCTTGAGAATACACGTAG | 22 | | | | | 479 | dme-mir-137 |
| mde-miR-10-5p | ACCCTGTAGATCCGAATTTGTT | 22 | | | | | 745 | aga-mir-10 |
| mde-miR-190-5p | AGATATGTTTGATATTCTTGGTTG | 24 | | | | | 7451 | dme-mir-190 |
| mde-miR-10-3p | CAAATTCGGTTCTAGAGAGGTTT | 23 | | | | | 136605 | aga-mir-10 |
| mde-miR-981-3P | TTCGTTGTCGACGAAACCTTTA | 22 | | | | | 7 |  |
| mde-miR-981-3P variant | TTCGTTGTCGACGAAACCTT | 20 | | | | | 3 | dme-mir-981 |
| mde-miR-210-3p | AGCTGCTGGCCACTGCACATGAT | 23 | | | | | 8 | dme-mir-210 |
| mde-let-7a-5p | TGAGGTAGTAGGTTGTATAGT | 21 | | | | | 44 | dme-let-7 |
| mde-miR-932-5p | TCAATTCCGAAGTGCATTGCAGT | 23 | | | | | 26 | dme-mir-932 |
| mde-miR-7b-5p | TGGAAGACTAGTGATTTTGTT | 21 | | | | | 335 | dme-mir-7 |
| mde-miR-965-3P | TAAGCGTAGAGCTTTTCCCCTTTT | 24 | | | | | 3 |  |
| mde-miR-965-3P variant | TAAGCGTAGAGCTTTTCCCCTT | 22 | | | | | 89 | dme-mir-965 |
| mde-miR-9a-5p | TCTTTGGTTATCTAGCTGTATGA | 23 | | | | | 798 | dme-mir-9a |
| mde-miR-279a-3p | TGACTAGATCCACACTCATTA | 21 | | | | | 43 | dme-mir-279 |
| mde-miR-279a-3p variant | TGACTAGATCCACACTCA | 18 | | | | | 3 | dpu-mir-279 |
| mde-miR-281-1-3p | CTGTCATGGAATTGCTCTCTTT | 22 | | | | | 24 | cqu-mir-281 |
| mde-miR-137-5p | ACGCGTATTCTTGGGTTATTA | 21 | | | | | 10 | dme-mir-137 |
| mde-miR-927-3p | CAAAGCGTTTGGATTCTGAAGC | 22 | | | | | 86 | dsi-mir-927 |
| mde-miR-927-3p variant | CAAAGCGTTTGGATTCT | 17 | | | | | 3 | bmo-mir-927 |
| mde-miR-12-5p | TGAGTATTACATCAGGTACTG | 21 | | | | | 502 | dme-mir-12 |
| mde-miR-929-5p | AAATTGACTCTAGTAGGGAGT | 21 | | | | | 13 | dsi-mir-929 |
| mde-miR-993b | TACCCTGTAGCTCCGGGCTTTT | 22 | | | | | 9 | dpu-mir-993 |
| mde-miR-308-3p | AATCACAGGAGTATACTGTGAG | 22 | | | | | 567 | aae-mir-308 |
| mde-miR-9c-5p | TCTTTGGTGGTTTTAGCTGTAT | 22 | | | | | 117 | dme-mir-9b |
| mde-miR-11-3p | CATCACAGTCTGAGTTCTTGCT | 22 | | | | | 660 | dme-mir-11 |
| mde-miR-998a-3p | TAGCACCATGAGATTCAGCTC | 21 | | | | | 238 | dme-mir-998 |
| mde-miR-2765-5p | TGGTAACTCCACCACCGTTGG | 21 | | | | | 4 | aae-mir-2765 |
| mde-miR-210-5p | CTTGTGCGTGTGACAGCGGCTAT | 23 | | | | | 51 | dme-mir-210 |
| mde-miR-124-3p | TAAGGCACGCGGTGAATGCCAA | 22 | | | | | 8786 | dme-mir-124 |
| mde-miR-970-3P | TCATAAGACACACGCGGCTAT | 21 | | | | | 3496 | dme-mir-970 |
| mde-miR-970-3P variant | TCATAAGACACACGCGGCT | 19 | | | | | 6 | bmo-mir-970 |
| mde-miR-274-5P | TTTGTGACCGACACTAACGGGT | 22 | | | | | 27 | dme-mir-274 |
| mde-miR-317-3p | TGAACACAGCTGGTGGTATCTCAGT | 25 | | | | | 852 | dpu-mir-317 |
| mde-miR-317-3p variant | TGAACACAGCTGGTGGTATC | 20 | | | | | 7 | dme-mir-317 |
| mde-miR-286-3p | TGACTAGACCGAACACTCGCGT | 22 | | | | | 23 | aae-mir-286a |
| mde-miR-276a-5p | AGCGAGGTTTAGAGTTCCTACG | 22 | | | | | 164 | dme-mir-276b |
| mde-miR-8-5p | CATCTTACCGGGCAGCATTAGA | 22 | | | | | 2299 | dme-mir-8 |
| mde-miR-263a-5p | AATGGCACTGGAAGAATTCACGGG | 24 | | | | | 32798 | dme-mir-263a |
| mde-miR-307-5p | ACTCACTCAACCTGGGTGTGATG | 23 | | | | | 3 | dme-mir-307 |
| mde-miR-305-5p | ATTGTACTTCATCAGGTGCTCTGG | 24 | | | | | 1101 | dme-mir-305 |
| mde-miR-263b-5p | CTTGGCACTGGGAGAATTCACAG | 23 | | | | | 137 | dme-mir-263b |
| mde-bantam | TGAGATCATTTTGAAAGCTGATTAT | 25 | | | | | 11 | dme-bantam |
| mde-miR-279d-3p | TGACTAGATTTTCACTCATCT | 21 | | | | | 91 |  |
| mde-miR-279d-3p variant | TGACTAGATTTTCACTCATC | 20 | | | | | 8 | bmo-mir-279d |
| mde-miR-989-3P | TGTGATGTGACGTAGTGGAT | 20 | | | | | 169 | dme-mir-989 |
| mde-miR-304-5p | AAATCTCAAATTGTAATTGTG | 21 | | | | | 4 | tca-mir-304 |
| mde-miR-304-5p variant | AAATCTCAAATTGTAATTGTGGG | 23 | | | | | 36 |  |
| mde-mir-92b-3p | CATTGCACTAGTCCCGGCCTGC | 22 | | | | | 439 | dme-mir-92b |
| mde-mir-14-3p | TCAGTCTTTTTCTCTCTCCTAT | 22 | | | | | 750 | dme-mir-14 |
| mde-miR-277-3P | TAAATGCACTATCTGGTACGACA | 23 | | | | | 42 | dme-mir-277 |
| mde-mir-275-3P | TCAGGTACCTGAAGTAGCGCGCG | 23 | | | | | 3404 | dme-mir-275 |
|  |  |  | | | | |  |  |
| **Novel miRNAs (184)** |  |  | | | | |  |  |
| PN-965-5p-1354 | TATTCTGGCGAAGAAATCGACGAAT | 25 | | | | | 199 |  |
| PC-3p-30842 | TAGAGATTGACCATACGGAGAGCAGT | 26 | | | | | 6 |  |
| PC-5p-27661 | TGAAATCGTACAGCGCATTGAAGATA | 26 | | | | | 6 |  |
| PC-5p-54858 | TACGAAATTGAACTTATTGTTGTTTT | 26 | | | | | 3 |  |
| PC-5p-68330 | GTAGATAGATATTTTGAAATTACA | 24 | | | | | 3 |  |
| PC-3p-28787 | ATTCGTTGAATTTGTTTGAA | 20 | | | | | 6 |  |
| PC-5p-176 | CAATATCAATTGGTAATTCTGG | 22 | | | | | 1530 |  |
| PC-3p-81 | TGACTAGATTTACACTCATCCA | 22 | | | | | 3242 |  |
| PC-5p-51244 | TATGGGACACGCAATTAATGAA | 22 | | | | | 3 |  |
| PC-5p-67443 | TCAAAACTGCGGAAGATAATCC | 22 | | | | | 3 |  |
| PC-3p-54311 | GAAAATTAACATAGTCGACTG | 21 | | | | | 3 |  |
| PC-5p-50354 | TGTTTGTAGAAATTTCCAGTCGATGT | 26 | | | | | 3 |  |
| PC-5p-30305 | TGTTATATGACGATACGTTCGGTAA | 25 | | | | | 6 |  |
| PC-5p-60782 | TGCATGTGAATGTGATGAATAGGTCC | 26 | | | | | 3 |  |
| PC-5p-45621 | TGGGAATATGGACCATGACAGGTGAA | 26 | | | | | 4 |  |
| PC-5p-31567 | AAACACCATTGTATTCGTTTCAT | 23 | | | | | 5 |  |
| PC-3p-5216 | ATCACAAGAATAATCGTTTGG | 21 | | | | | 42 |  |
| PC-3p-45698 | TCAGTCGCCGATCCGTTTGACA | 22 | | | | | 4 |  |
| PC-3p-16936 | TATTGATTGCATGTCCCATATG | 22 | | | | | 11 |  |
| PC-3p-7013 | AATGTTGTTTCGGGACATTA | 20 | | | | | 30 |  |
| PC-5p-32133 | AAATGAGTTGATTGAATTGCT | 21 | | | | | 5 |  |
| PC-3p-15390 | TAGAATCTCTCCATCGGATTAA | 22 | | | | | 12 |  |
| PC-5p-39054 | TGGTTTCAGATTCGTCGTCGATTATT | 26 | | | | | 4 |  |
| PC-3p-55176 | TATTTTCTTAGACAAATCGGCGGA | 24 | | | | | 3 |  |
| PC-5p-9391 | CCGGATGACAGTCTCAAACGGTC | 23 | | | | | 22 |  |
| PC-5p-5408 | TTAGATCATTTTTGTGGTAATCGGCG | 26 | | | | | 41 |  |
| PC-5p-57282 | TATTGTACGACGGTTAAGCGTGTATG | 26 | | | | | 3 |  |
| PC-3p-50487 | TACATTCCGGCTACTTTCTATGCAT | 25 | | | | | 3 |  |
| PC-5p-52274 | ATGTATCGCACCTTCTCTTTC | 21 | | | | | 3 |  |
| PC-3p-29582 | TACCAGGATAACGCGATCACCAAACA | 26 | | | | | 6 |  |
| PC-5p-64118 | TGTTATTGTTTTATCGGAATGGTATT | 26 | | | | | 3 |  |
| PC-3p-66779 | TAACTTCAATTTTGATTATCGGTAA | 25 | | | | | 3 |  |
| PC-3p-87 | GTAGGCCGGTGAAACTACTTTC | 22 | | | | | 2935 |  |
| PC-5p-2948 | TGAAGATACAAGATAAGATGAAGATA | 26 | | | | | 81 |  |
| PC-5p-5905 | GCTGAAATCTCGTGGATCTGCA | 22 | | | | | 37 |  |
| PC-5p-39757 | TAATGTAGAGCATTGGCGCAATTTTA | 26 | | | | | 4 |  |
| PC-3p-39629 | TAAAGTACTTTGAATTAGATGAATGA | 26 | | | | | 4 |  |
| PC-3p-50597 | TGGATTAATGTGATCGTCAGAAAATA | 26 | | | | | 3 |  |
| PC-5p-12050 | TCATCCTCGGCGTCATATATAGGGAA | 26 | | | | | 16 |  |
| PC-3p-22425 | TAAGAGAAAGACATGCAGACTTTTCT | 26 | | | | | 8 |  |
| PC-5p-36494 | TGAGAATGATATTGTGAT | 18 | | | | | 5 |  |
| PC-5p-46942 | TAGGAGAATGCAAACATGATTCATGC | 26 | | | | | 4 |  |
| PC-5p-36861 | AGCGATTGTGAAAAGACG | 18 | | | | | 5 |  |
| PC-5p-29265 | TGGGAATGTCAATGGCGAGA | 20 | | | | | 6 |  |
| PC-3p-27532 | TTGGACTGAGTGATGTGGTATATAAT | 26 | | | | | 6 |  |
| PC-5p-57969 | TGTTTGCCTTCAACAATCGTTGTGAC | 26 | | | | | 3 |  |
| PC-3p-31500 | TCGGTATTGCTGCATGAAGGGTTTTC | 26 | | | | | 5 |  |
| PC-3p-1975 | CGGCACGTGTTGGAGTACACTCA | 23 | | | | | 129 |  |
| PC-3p-13166 | CAGTGCTTATGTTATGCTCGCA | 22 | | | | | 15 |  |
| PC-3p-58746 | ATTGGCATCATGAGATTTGAC | 21 | | | | | 3 |  |
| PC-3p-34325 | ATGTTCTTGGTTTTGATTGTG | 21 | | | | | 5 |  |
| PC-3p-59454 | TCTGAAATGCGTAGAAAAACA | 21 | | | | | 3 |  |
| PC-3p-36826 | TATGGCTTGATGTAGACGATAGGTTG | 26 | | | | | 5 |  |
| PC-3p-1632 | AGAGATTCCACTTTTCTAACTTAT | 24 | | | | | 159 |  |
| PC-3p-66266 | TTTGGACCAGTTCGGACCAGTT | 22 | | | | | 3 |  |
| PC-5p-47339 | TAATGGATTCTTCGAATT | 18 | | | | | 4 |  |
| PC-3p-2253 | TAAAGCTAGTTGACCGAAGTTA | 22 | | | | | 110 |  |
| PC-3p-24964 | TAAGTCTATGATTTCGTTCGGAGCAT | 26 | | | | | 7 |  |
| PC-5p-35949 | CTCTCAAAATGACTGTGAAATG | 22 | | | | | 5 |  |
| PC-3p-15252 | TAGCGTATGTTAGGCGTCGTTGATTG | 26 | | | | | 12 |  |
| PC-5p-19199 | TAGAGGATTCGGAAGTTTCGGAAGTT | 26 | | | | | 9 |  |
| PC-5p-36969 | TAGAATGTCTGTGAATAGAAACGAGC | 26 | | | | | 5 |  |
| PC-5p-63046 | TCGTTGATCGGCATATGGGTGGCGT | 25 | | | | | 3 |  |
| PC-3p-53754 | AGAAATCAAGATACTGAAATCG | 22 | | | | | 3 |  |
| PC-3p-21315 | TCAATGCAAATCGTTTGTT | 19 | | | | | 8 |  |
| PC-3p-55944 | TCAGACGATGAAATCGACATA | 21 | | | | | 3 |  |
| PC-5p-40924 | TAATTACTGAGTTCGTTTTATCGGAT | 26 | | | | | 4 |  |
| PC-3p-5899 | CATTGCACTAGTCCCGGCCTGT | 22 | | | | | 37 |  |
| PC-3p-45385 | ATGTATCCAGGTATTGCTGA | 20 | | | | | 4 |  |
| PC-5p-54448 | TTCGAGATACGGCTTACATGACTTTT | 26 | | | | | 3 |  |
| PC-5p-24815 | GTTAGTTTTGAACGGGTTTTGGT | 23 | | | | | 7 |  |
| PC-5p-34434 | TGCAATGGAACTTGATGAAAGGCGTT | 26 | | | | | 5 |  |
| PC-5p-10538 | ACTCACAAAGTGGATGTGAAATG | 23 | | | | | 19 |  |
| PC-5p-27853 | CCGCTGATCGGTGAGGGGCAA | 21 | | | | | 6 |  |
| PC-5p-40470 | TGCAAGGCTGAGCGCTGCAACGGCAA | 26 | | | | | 4 |  |
| PC-5p-13305 | TTCATATGGGACACGCAATTAA | 22 | | | | | 14 |  |
| PC-5p-34290 | TCCGATCGTTGTAGCAGTATTGATGA | 26 | | | | | 5 |  |
| PC-5p-56048 | TCGGAATTGCTCAGTAAGTTGGTGTT | 26 | | | | | 3 |  |
| PC-5p-43858 | GTTGTGATTTGTATTGGTGTT | 21 | | | | | 4 |  |
| PC-5p-53148 | TATGACTGCACCTTTATTTTGTATGA | 26 | | | | | 3 |  |
| PC-5p-34685 | TGGATAGTTGATATATATGT | 20 | | | | | 5 |  |
| PC-5p-2087 | GGGTTCAAAGGAGAGCATGTA | 21 | | | | | 121 |  |
| PC-5p-57422 | TAGTGAACTGGAAGATGTTGTGCCAT | 26 | | | | | 3 |  |
| PC-5p-4157 | TCGTGAAAATGGTTGTGCAATG | 22 | | | | | 55 |  |
| PC-5p-39653 | TCAGAACGGAGCAAGACAAATTGATC | 26 | | | | | 4 |  |
| PC-5p-60610 | AAGGTCAAGGAAGTTGGTG | 19 | | | | | 3 |  |
| PC-3p-15868 | TAAGTGAAGAATTAGAATTTACAGA | 25 | | | | | 12 |  |
| PC-3p-52772 | TACGACTTATTGCATCAGCTCGGCCT | 26 | | | | | 3 |  |
| PC-3p-16994 | TGACTAGATTCAATGCTCATCT | 22 | | | | | 11 |  |
| PC-5p-66350 | TGATGTTTGTTTATGGGTTGAA | 22 | | | | | 3 |  |
| PC-5p-46103 | TCCTCAGAGCTCGTAATTTCGGCG | 24 | | | | | 4 |  |
| PC-3p-43520 | TAATATAAATCGGCGTCGTTACCAAA | 26 | | | | | 4 |  |
| PC-5p-60000 | TAAGACATTTTCGTTCGGTTTGAAT | 25 | | | | | 3 |  |
| PC-5p-28286 | CCATTCTTCCTTGCATTCGATA | 22 | | | | | 6 |  |
| PC-3p-4640 | AGCCACTATCCGCCCTGCCATT | 22 | | | | | 48 |  |
| PC-5p-43996 | AATAAATCGATCTTTTTTGGC | 21 | | | | | 4 |  |
| PC-5p-36024 | TGGATTGTAAACGCCGCGTATGAAAT | 26 | | | | | 5 |  |
| PC-3p-16219 | TCACTGGGCTTTGTTTGTCGC | 21 | | | | | 11 |  |
| PC-5p-5576 | TGGGGTACTCCCTGTGGTCGCT | 22 | | | | | 39 |  |
| PC-3p-41277 | AGTTCTCAGTCCGACGATC | 19 | | | | | 4 |  |
| PC-5p-31927 | CGGTACGGGTTTCTTTTCGAG | 21 | | | | | 5 |  |
| PC-5p-12784 | TTTGTCTTAAGCTGTACGAGGCA | 23 | | | | | 15 |  |
| PC-5p-12191 | TGTATTATTTATTGGATTAC | 20 | | | | | 16 |  |
| PC-3p-50458 | TCAGATATGGTGGCTAAATTTGGAAT | 26 | | | | | 3 |  |
| PC-5p-60454 | TATTCGCAATAGACGGAATGGACGAT | 26 | | | | | 3 |  |
| PC-3p-24245 | TGCGAATTATATGTGCCGAAGGATGA | 26 | | | | | 7 |  |
| PC-3p-22886 | GAGAGTGCTCTTGTAGCTGGTC | 22 | | | | | 8 |  |
| PC-5p-20866 | TGCAGAACAACGAACCAGTGGACAGC | 26 | | | | | 9 |  |
| PC-3p-67661 | TATCTTTTATCTCATCTTCATCTTGA | 26 | | | | | 3 |  |
| PC-3p-20321 | TGAAGACGATGTGTAGTACGTTGGAG | 26 | | | | | 9 |  |
| PC-5p-43905 | ACATCTTAGAATTTGCTTTTTACCT | 25 | | | | | 4 |  |
| PC-3p-31364 | TAGCTGGGTGACGTGACCTGAG | 22 | | | | | 5 |  |
| PC-3p-42445 | ATGAGTTGATTGAACTGCTT | 20 | | | | | 4 |  |
| PC-5p-19011 | ATATGAGGTAGTAGGTTGTATAGT | 24 | | | | | 10 |  |
| PC-5p-121 | GGGAGCGAGATCGGGGCTTACT | 22 | | | | | 2032 |  |
| PC-5p-66343 | TAAGAGCTTGATATGACCGACGGTGC | 26 | | | | | 3 |  |
| PC-5p-61169 | GAGCGCGGTTAGTACTTGG | 19 | | | | | 3 |  |
| PC-5p-19388 | TAAAATGCGTGTTGTTAGTTTGACGG | 26 | | | | | 9 |  |
| PC-5p-24944 | TCTTTGGTGTTCTAGCTTATGA | 22 | | | | | 7 |  |
| PC-5p-34092 | TATCCATCGTTGCCTGTAATGGTGTC | 26 | | | | | 5 |  |
| PC-3p-8699 | CGTGATCTCTTGGTGGCATCAT | 22 | | | | | 24 |  |
| PC-5p-27025 | TCGATTGAATTGTTGAGGTTGGGCAT | 26 | | | | | 6 |  |
| PC-5p-58311 | CATTCTGTTTTCGAAAAGT | 19 | | | | | 3 |  |
| PC-3p-44517 | TGTGTTAATCGTTGTTGTAATTGGTA | 26 | | | | | 4 |  |
| PC-3p-45501 | TATCACAGCCATTTGATGAGCTA | 23 | | | | | 4 |  |
| PC-3p-66589 | TGCAGACGGCAAATTCTTTTGAGGCA | 26 | | | | | 3 |  |
| PC-3p-44258 | TCAGTGTTCTGTAAAGATTAAGATAA | 26 | | | | | 4 |  |
| PC-5p-23638 | ATCAGGAATAATCGTTTGGTC | 21 | | | | | 7 |  |
| PC-3p-28519 | TACGGTCAGACTAGGCAAATATTTGC | 26 | | | | | 6 |  |
| PC-5p-808 | TATCACAGTTGCTATTTCTGTCA | 23 | | | | | 348 |  |
| PC-5p-39989 | AGCGATATTGAACCTTATTCACT | 23 | | | | | 4 |  |
| PC-3p-30583 | TAAAGCTGGGCCATCACTGTGA | 22 | | | | | 6 |  |
| PC-3p-14036 | TAGCTGCCTCGTGAAAGACGATA | 23 | | | | | 14 |  |
| PC-5p-52354 | AGCTGGTTGGTTCCGGGTCAGAT | 23 | | | | | 3 |  |
| PC-5p-36542 | ATTGTTTTTTCATTCGTTTGTT | 22 | | | | | 5 |  |
| PC-5p-33179 | TAAGTATCTGTTGTTGTTGT | 20 | | | | | 5 |  |
| PC-5p-8731 | AAGTGGAGCAGTGGTCTCATT | 21 | | | | | 23 |  |
| PC-5p-62589 | TAAGAACACAAGGTGACAACGGATCT | 26 | | | | | 3 |  |
| PC-3p-66604 | ATCAAATCGAATGGATG | 17 | | | | | 3 |  |
| PC-3p-22471 | GGATCAAGAGATTGTCGGT | 19 | | | | | 8 |  |
| PC-3p-6839 | AGGATTGTATCTGGACGTCGTT | 22 | | | | | 32 |  |
| PC-3p-19591 | TCCTGGTCAAGTACTTCTTGAGGCT | 25 | | | | | 9 |  |
| PC-5p-607 | TTTTGATTGTTGCTCGAAATTC | 22 | | | | | 468 |  |
| PC-3p-11315 | TATCCTTATATGTGTTGCTTCGGGAA | 26 | | | | | 17 |  |
| PC-5p-29991 | TACTAATCGAAAAGCCTAATACCGG | 25 | | | | | 6 |  |
| PC-5p-17380 | AGGGGTTTCTTTCGGCCTCCCG | 22 | | | | | 11 |  |
| PC-5p-1662 | AGCGATATTGAACCTTATTCAC | 22 | | | | | 156 |  |
| PC-3p-39779 | ATTAAATAAAATGATTCGTGC | 21 | | | | | 4 |  |
| PC-3p-59244 | TCGAGATGGTTGTTGACGATAATTTC | 26 | | | | | 3 |  |
| PC-5p-54579 | TCGAGGAGGAAATACGTAGCATGGAA | 26 | | | | | 3 |  |
| PC-5p-8545 | TAGACATGTAAAAAGGAAGATGAATG | 26 | | | | | 24 |  |
| PC-3p-62130 | TGTATGAAATATGGACGCGGCTGAAA | 26 | | | | | 3 |  |
| PC-3p-17249 | TGCATGACGACTTTTAAATTCATCGA | 26 | | | | | 11 |  |
| PC-5p-59796 | AGAGGAGGTATGATAATGGGG | 21 | | | | | 3 |  |
| PC-3p-150 | CATACTCTCCTTTCGATCTCA | 21 | | | | | 1724 |  |
| PC-3p-2808 | TGATTCAAAACTGATAAAT | 19 | | | | | 86 |  |
| PC-3p-67513 | TGGATATGCAGAATCGCCCAAAATAA | 26 | | | | | 3 |  |
| PC-3p-6605 | ATATGAATGAAAAAGAACAA | 20 | | | | | 33 |  |
| PC-5p-41510 | TAGTAATGAACAAGTAAGTGCCTTAG | 26 | | | | | 4 |  |
| PC-3p-1992 | TGATTCAAAACTGATGAAT | 19 | | | | | 128 |  |
| PC-5p-55109 | TCGGATTGTGGATTGACTTT | 20 | | | | | 3 |  |
| PC-5p-46215 | TATTGAATATTGAATACAC | 19 | | | | | 4 |  |
| PC-3p-52837 | TCAATTGTTTCGATTTCGATCTCGGC | 26 | | | | | 3 |  |
| PC-3p-36814 | TACGATTATCAGATGATGCTTGAACT | 26 | | | | | 5 |  |
| PC-3p-62087 | TCATTGTCGTCTTCAGTTGGCATTTC | 26 | | | | | 3 |  |
| PC-5p-46633 | CAATACTTTGTAAGAGCTGGA | 21 | | | | | 4 |  |
| PC-5p-63727 | CAAACAGCCATTATTCATGCA | 21 | | | | | 3 |  |
| PC-3p-18350 | TATTGTATTGCTTAAGGCTTGACGTC | 26 | | | | | 10 |  |
| PC-3p-28593 | TGGGTTGATTAGTGCAGTTAT | 21 | | | | | 6 |  |
| PC-3p-67441 | TCGGGAACTGCAGTTGAACGTTCTAG | 26 | | | | | 3 |  |
| PC-3p-3802 | TATCACATCCGAAAGGTTTCCC | 22 | | | | | 60 |  |
| PC-3p-47103 | AGTGTGACAACTGCCGGACA | 20 | | | | | 4 |  |
| PC-5p-334 | GGCATCAAATTTGGCTGTATTA | 22 | | | | | 839 |  |
| PC-3p-30302 | CAATGGAAGTTGGAGCTACCGCG | 23 | | | | | 6 |  |
| PC-5p-56089 | TCAGAATCGAAAAGTGACGATGAAAT | 26 | | | | | 3 |  |
| PC-5p-41281 | ATTGGAATATTGCTCAAGGAAT | 22 | | | | | 4 |  |
| PC-3p-760 | CACGTTGCAGATTGGGGTTACT | 22 | | | | | 372 |  |
| PC-3p-61755 | TTGGTGATTTGGAATGAA | 18 | | | | | 3 |  |
| PC-5p-57811 | TGGGACTTCTCAAAACTGCGGA | 22 | | | | | 3 |  |
| PC-3p-1586 | TATAGACGTGGAAAATCGTTTA | 22 | | | | | 164 |  |
| PC-3p-6908 | CGGAATTCCAACTGATATACGC | 22 | | | | | 31 |  |
| PC-5p-18554 | GATGAGTGTTAGTTTGGTGCATA | 23 | | | | | 10 |  |
| PC-5p-57896 | TGGAATCTTGATCGAGCACGAATTTC | 26 | | | | | 3 |  |
| PC-3p-36631 | TAGGAGTTTTGATCACGGTAAAGTCA | 26 | | | | | 5 |  |
