# Supplementary material for: Deep sequencing and genome-wide analysis reveals the expansion of MicroRNA genes in the gall midge Mayetiola destructor
Source: BMC Genomics. 2013 Mar 18;14:187. doi: 10.1186/1471-2164-14-187 (PMC3608969; doi:10.1186/1471-2164-14-187)
Supplement: Additional file 3: Figure S2 — Name, sequence, identification method, and loop-structure of 530 novel and 126 known miRNAs. The region of miRNA in the stem-loop structure is bold and in red color. [file 1471-2164-14-187-S3.docx]

**Figure S2. Name, sequence, identification method, and loop-structure of 530 novel and 126 known miRNAs.** The region of miRNA in the stem-loop structure is bold and in red color.

**530 novel putative miRNAs**

PN-965-5p-1354:

UAUUCUGGCGAAGAAAUCGACGAAU

Identified by deep-sequencing

Stem-loop structure:

AAU**UAU** -- AU AUUUU A U A CUU U

**UCUGGC** **GAAGAAAUCGACGAAU**AUC UUCGA AUUGA AUAGCGUG UUGAUUCAG UUGAAU CGUGCGA G

GGGCCG CUUCUUUAGCUGCUUAUAG AAGCU UAGCU UAUCGCAC AACUAAGUC GACUUA GCACGCU G

AUAUGC UU -- CUUU- C U - U-- A

PC-5p-54858:

UACGAAAUUGAACUUAUUGUUGUUUU

Identified by deep-sequencing

Stem-loop structure:

**C** - **CU** **U** **U**AG

A**UA** **GAAA UUGAA UAUUGUUG** **UU** U

UAU CUUU GAUUU AUAACAGC AG U

A C UU C CGU

PC-5p-68330:

GUAGAUAGAUAUUUUGAAAUUACA

Identified by deep-sequencing

Stem-loop structure:

AU **G**- **A** **AC** UA U

UCA UGG **UAGAUAG UAUUUUGAAAUU A**UUAG AU C

AGU AUC AUUUAUC AUAAAGCUUUAA UAAUU UA C

-- GG A CC GG A

PC-3p-28787-1:

AUUCGUUGAAUUUGUUUGAA

Identified by deep-sequencing

Stem-loop structure:

C CA **A**

UAUUCG AAUAAAUUC AAC **U**

AU**AAGU** **UUGUUUAAG** **UUG** **U**

- -- **C**

PC-3p-28787-2:

AUUCGUUGAAUUUGUUUGAA

Identified by sequence similarity

Stem-loop structure:

C UU AU- - UAAAUU

AUUUUA UA UUCAAA AUUCG CGA C

UAAAAU AU **AAGUUU UAAGU GCU** C

A -- **GUU U** **UA**ACGU

PC-5p-51244:

UAUGGGACACGCAAUUAAUGAA

Identified by deep-sequencing

Stem-loop structure:

**C** G U UAU

AUGGGGAACUUCA**UAUGGGACA GCAAUUAAUGAA** UA ACUU U

UACCCCUUGAAGUAUACCCUGU CGUUAGUUAUUU AU UGAA A

A A U UUA

PC-5p-57811-1:

UGGGACUUCUCAAAACUGCGGA

Identified by deep-sequencing

Stem-loop structure:

**CA** **C** CCUA AAA

UGAAUCGCUGAGAAUAAAUA**UGGGACUUCU** **AAA** **UGCGGA**AGAUAAU GCAU \

ACUUAGCGACUCUUAUUUAUACCCUGAAGA UUU ACGCCUUCUAUUG CGUA A

AC A ---- AAU

PC-5p-57811-2:

UGGGACUUCUCAAAACUGCGGA

Identified by sequence similarity

Stem-loop structure:

**A C C** A CCCA AAA

AAUUGAAUCGCUGAGAAUAAAUA**UGGGACUUCUC** **AAA UG GGA**AG UAAU GCAU \

UUAACUUAGUGACUCUUAUUUAUACCCUGAAGAG UUU AC CUUUC AUUG CGUA A

C A A G ---- AAU

PC-5p-57811-3:

UGGGACUUUUCAAAACUGCGGA

Identified by sequence similarity

Stem-loop structure:

**A C** CCCA AAA

GAAUCGCUGAGAAUAAAUA**UGGGACUUUUC** **AAA UGCGGA**AGAUAAU GCAU \

CUUAGUGACUCUUAUUUAUACCCUGAAAAG UUU ACGCUUUCUAUUG CGUA A

C A ---- AAU

PC-5p-57811-4:

UGGGACUUCUCAAAACUGCGGA

Identified by sequence similarity

Stem-loop structure:

C G **U** **A C** CCCA AAA

UUGUACAAUUGAAU GCU AGAAUAAAUA **GGGACUUCUC AAA UGCGGA**AGAUAAU GCAU \

AAUAUGUUAACUUA CGA UCUUAUUUAU CCCUGAAGAG UUU ACGCCUUCUAUUG CGUA A

A G U C A ---- AAU

PC-5p-57811-5

AGGGACUUCUCGAAAUUGCGGA

Identified by sequence similarity

Stem-loop structure:

A- C **A** C---- UUA

UAUU UACAAUUGAAUUGCU AGAAUAAAUA **GGGACUUCUCGAAAUUGCGGAA**GAUAA GCAU \

AUAA AUGUUAACUUAGCGA UCUUAUUUAU CCCUGAAGAGUUUUGACGCCUUCUAUU CGUA U

AC C A AGGGU UUU

PC-5p-57811-6:

UGGGACUUCUCAAAACUGCGGA

Identified by sequence similarity

Stem-loop structure:

C C A **A C** CCCA AAA

UGGAA UA AAUUGAAUC CUGAGAAUAAAUA**UGGGACUUCUC** **AAA UGCGGAA**GAUAAU GCAU \

ACCUU AU UUAACUUAG GACUCUUAUUUAUACCCUGAAGAG UUU ACGCCUUCUAUUA CGUA A

U - C C A ---- AAU

PC-5p-57811-7:

UGGGACUUCUCAAAACUGCGGA

Identified by sequence similarity

Stem-loop structure:

**A C** CCCA AAA

GUAGUAUAUAUGAAUCGCUGAGAAUAAAUA**UGGGACUUCUC** **AAA UGCGGA**AGAUAAU GCAU \

CAUCAUAUAUACUUAGCGACUCUUAUUUAUACCCUGAAGAG UUU ACGCCUUCUAUUG CGUA A

C A ---- AAU

PC-5p-57811-8:

UGGGACUUCUCAAAACUGCGGA

Identified by sequence similarity

Stem-loop structure:

C **A C** U CAGCAUA

AAUUGAAU GCUGAGAAUAAAUA**UGGGACUUCUC AAA UGCGGA**AGA AAUGC \

UUAACUUA CGACUCUUAUUUAUACCCUGAAGAG UUU ACGCCUUCU UUGCG A

A C A C UAAAUAA

PC-5p-57811-9:

UGGGACUUCUCAAAACUGCGGA

Identified by sequence similarity

Stem-loop structure:

U **A C** CCCA AAA

AUAUAUAUGAA CGCUGAGAAUAAAUA**UGGGACUUCUC AAA UGCGGA**AGAUAAU GCAU \

UAUAUAUACUU GCGACUCUUAUUUAUACCUUGAAGAG UUU ACGCCUUCUAUUG CGUA A

U C A ---- AAU

PC-5p-57811-10

UGGGACUUCUCAAAACUGCGGA

Identified by sequence similarity

Stem-loop structure:

C **G C A C**  CCCA AAA

AUAUAUAUGAAU GCUGAGAAUAAAUA**U GGACUU UC AAA UGCGGA**AGAUAAU GCAU \

UAUAUAUACUUA CGACUCUUAUUUAUA UCUGAA AG UUU ACGCCUUCUAUUG CGUA A

A A A C A ---- AAU

PC-5p-57811-11:

UGGGACUUCUCAAAACUGCGGA

Identified by sequence similarity

Stem-loop structure:

**A C** CCCA AAA

UGAAUCGCUGAGAAUAAAUA**UGGGACUUCUC AAA UGCGGA**AGAUAAU GCAU \

ACUUAGCGACUCUUAUUUAUACCCUGAAGAG UUU ACGCCUUCUAUUG CGUA A

G A ---- AAU

PC-5p-57811-12:

UGGGACUUCUCAAAACUGCGGA

Identified by sequence similarity

Stem-loop structure:

C **A C** CCCA AAA

AAUUGAAU GCUGAGAAUAAAUA**UGGGACUUCUC AAA UGCGGA**AGAUAAU GCAU \

UUAACUUA CGACUCUUAUUUAUACCCUGAGGAG UUU ACGCCUUCUAUUG CGUA A

A C A ---- AAU

PC-5p-57811-13:

UGGGACUUCUCAAAACUGCGGA

Identified by sequence similarity

Stem-loop structure:

**A C** CCCA AAA

AUUGAAUCGCUGAGAAUAAAUA**UGGGACUUCUC AAA UGCGGA**AGAUAAU GCAU \

UAACUUAGCGACUCUUAUUUAUACCCUGAAGAG UUU ACGCCUUCUAUUG CGUA A

C A ---- AAU

PC-5p-57811-14:

UGGGACUUCUCAAAACUGCGGA

Identified by sequence similarity

Stem-loop structure:

**C A C** CCCA AAA

UUGAAUCGCUGAGAAUAAAUA**UGGGACUU UC AAA UGCGGA**AGAUAAU GCAU \

AACUUAGCGACUCUUAUUUAUACCCUGAA AG UUU ACGCCUUCUAUUG CGUA A

A C A ---- AAU

PC-5p-57811-15:

UGGGACUUCUCAAAACUGCGGA

Identified by sequence similarity

Stem-loop structure:

**C A C**  CCCA AAA

AUAAAUA**UGGGA UUCUC AAA UGCGGA**AGAUAAU GCAU \

UAUUUAUACCCU AAGAG UUU ACGCCUUCUAUUG CGUA A

U C A ---- AAU

PC-5p-57811-16:

UGGGACUUCUCAAAACUGCGGA

Identified by sequence similarity

Stem-loop structure:

**A C** CCCA AAA

UUGAAUCUCUGAGAAUAAAUA**UGGGACUUCUC AAA UGCGGA**AGAUAAU GCAU \

GACUUAGGGACUCUUAUUUAUACCCUGAAGAG UUU ACGCCUUCUAUUG CGUA A

C A ---- AAU

PC-5p-57811-17:

UGGGACUUCUCAAAACUGCGGA

Identified by sequence similarity

Stem-loop structure:

UU C **A C** CACA AAA

UAUA UGAAUUGCUGAGAA AAAUA**UGGGACUUCUC AAA UGCGGA**AGAUAAU GCAU \

AUGU ACUUAGCGACUCUU UUUAUACCCUGAAGAG UUU ACGCCUUCUAUUG CGUA A

UU A C A ---- AAU

PC-5p-57811-18:

UGGGACUUCUCAAAACUGCGGA

Identified by sequence similarity

Stem-loop structure:

U **A C** CCCA AAA

AUAUGAAUCGCU AGAAUAAAUA**UGGGACUUCUC AAA UGCGGA**AGAUAAU GCAU \

UAUACUUAGCGA UCUUAUUUAUACCCUGAAGAG UUU ACGCCUUUUAUUG CGUA A

C C A ---- AAU

PC-5p-57811-19:

UGGGACUUCUCAAAACUGCGGA

Identified by sequence similarity

Stem-loop structure:

**A C** CCCA AAA

AUA**UGGGACUUCUC AAA UGCGGA**AGAUAAU GCAU \

UAUACCCUGAAGAG UUU ACGCCUUCUAUUG CGUA A

C A ---- AAU

PC-5p-57811-20:

UGGGACUUCUCAAAACUGCGGA

Identified by sequence similarity

Stem-loop structure:

CU C G **A C** C CCCA AAA

UUAUA UA AUAUGAAU GCUGAGAAUAAAUA**UGGGACUUCUC AAA UGCGGA** GAUAAU GCAU \

AAUAU AU UAUACUUA CGACUCUUAUUUAUACCCUGAAGAG UUU ACGCCU CUAUUG CGUA A

U- U G C A U ---- AGU

PC-5p-57811-21:

UGGGACUUCUCAAAACUGCGGA

Identified by sequence similarity

Stem-loop structure:

**AA C** CCCA AAA

ACAAUUGAAUCGCUGAGAAUAAAUA**UGGGACUUCUC AA UGCGGA**AGAUAAU GCAU \

UGUUAACUUAGCGACUCUUAUUUAUACUCUGAAGAG UU ACGCCUUCUAUUG CGUA A

CC A ---- AAU

PC-5p-57811-22:

UGGGACUUCUCAAAACUGCGGA

Identified by sequence similarity

Stem-loop structure:

**A C** CCCA AAA

UGAAUCGCUGAGAAUAAAUA**UGGGACUUCUC AAA UGCGGA**AGAUAAU GCAU \

ACUUAGCGACUCUUAUUUAUACCCUGAAGAG UUU ACGCCUUCUAUUG CGUA A

C A ---- AAU

PC-5p-57811-23:

UGGGACUUCUCAAAACUGCGGA

Identified by sequence similarity

Stem-loop structure:

C CACA AAA

UGGAUCGCUGAGAAUAAAUAUGGGACUUCUCAAAA UGCGGAAGAUAAU GCAU \

ACUUAGCGACUCUUAUUUAUACCCUGAAGAGUUUU ACGCCUUCUAUUG CGUA A

A ---- AAU

PC-5p-57811-24:

UGGGACUUCUCAAAACUGCGGA

Identified by sequence similarity

Stem-loop structure:

- C A **C** CCCA AAA

AAGAA GAU AAUUG AUCGCUGAGAAUAAAUA**UGGGACUUCUCAAAA UGCGGA**AGAUAAU GCAU \

UUUUU CUG UUGAC UAGCGACUCUUAUUUAUACCCUGAAGAGUUUU ACGCCUUCUAUUG CGUA A

G A C A ---- AAU

PC-5p-57811-25

UGGGACUUCUCAAAACUGCGGA

Identified by sequence similarity

Stem-loop structure:

CCCA AAA

AUAUGAAUCGCUGAGAAUAAAUA**UGGGACUUCUCAAAACUGCGGA**AGAUAAU GCAU \

UAUACUUAGCGACUCUUAUUUAUACCCUGAAGAGUUUUGACGCCUUCUAUUG CGUA A

---- AAU

PC-5p-57811-26:

UGGGACUUCUCAAAACUGCGGA

Identified by sequence similarity

Stem-loop structure:

**A C** CCCA AAA

UAUAUAUGAAUCGCUGAGAAUAAAUA**UGGGACUUCUC AAA UGCGGGA**GAUAAU GCAU \

AUAUAUACUUAGCGACUUUUAUUUAUACCCUGAAGAG UUU ACGCCUUCUAUUG CGUA A

C A ---- AAU

PC-5p-57811-27:

UGGGACUUCUCAAAACUGCCGA

Identified by sequence similarity

Stem-loop structure:

AA - A A **U C C** CC GAAUA

AAG CAU AUAU UAUGAAUCGCUGAGAAUAAAU **UGGGACU CUCAAAA UGC GA**AGAUAAU CA A

UUU GUA UAUA AUACUUAGCGACUCUUAUUUA ACCCUGA GAGUUUU ACG UUUCUAUUG GU A

AG C A C U A C C- AAAUA

PC-5p-57811-28:

UGGGACGUCUCAAAACUGCGGA

Identified by sequence similarity

Stem-loop structure:

C **G A C** CCAA AAA

UAAUACAAUUGAAU GCUGAGAAUAAAUA**UGGGAC UCUC AAA UGCGGA**AGAUAAU GCAU \

GUUAUGUUAACUUA CGACUCUUAUUUAUAUCCUG AGAG UUU ACGCCUUCUAUUG CGUA A

A A C A ---- AAU

PC-5p-57811-29:

UGGGACUUUUCAAAACUGCGGA

Identified by sequence similarity

Stem-loop structure:

A U **A C** ACCCA AAA

UAUAUGAAUC CUGAGA UAAAUA**UGGGACUUUUC AAA UGCGGA**AGAUAA GCAU \

GUAUACUUAG GACUCU AUUUAUAUCCUGAAAAG UUU ACGCCUUCUAUU CGUA A

C U C A G---- AAU

PC-5p-57811-30:

UGGGACCUCUCAAAACUGCGGA

Identified by sequence similarity

Stem-loop structure:

**C A C** CCCA CUA

CAAUUGAAUCGCUGAGAAUAAAUA**UGGGAC UCUC AAA UGCGGA**AGAUAAU GCAU \

GUUAACUUAGCGACUCUUAUUUAUACCCUG AGAG UUU ACGCCUUCUAUUG CGUA A

A C A ---- AAU

PC-5p-57811-31:

UGGGACUUUUCAAAACUGCGGA

Identified by sequence similarity

Stem-loop structure:

UU **A C** UCCA AAA

UA UGAAUCGCUGAGAAUAAAUA**UGGGACUUUUC AAA UGCGGA**AGAUAAU GCAU \

AU ACUUAGCGACUCUUAUUUAUACUCUGAAAAG UUU ACGCCUUCUAUUG CGUA A

UU C A ---- AAU

PC-5p-57811-32:

UGGGACUUUUCAAAACUGCGGA

Identified by sequence similarity

Stem-loop structure:

G **A C** CCCA AAA

UAUAUGAAUCGCUGA AAUAAAUA**UGGGACUUUUC AAA UGCGGA**AGAUAAU GCAU \

GUAUACUUAGCGACU UUAUUUAUACCCUGAAAAG UUU ACGCCUUCUAUUG CGUA A

A C A ---- AAU

PC-5p-57811-33:

UGGUACUUCUCAAAACUGCGGA

Identified by sequence similarity

Stem-loop structure:

**GU A C** A CCCA AAA

AUAUGAAUCGCUGAGAAUAAAUA**UG** **ACUUCUC AAA UGCGGA**AG UAAU GCAU \

UAUACUUAGCGACUCUUAUUUAUAC UGAAGAG UUU AUGCCUUC AUUG CGUA A

UC C A G ---- AAU

PC-5p-57811-34:

UGGGACUUCUCAAAGCUGCGGA

Identified by sequence similarity

Stem-loop structure:

**AA C** UCCA AAA

CAAUUGAAUUGCUGAGAAUAAAUA**UGGGACUUCUC AG UGCGGA**AGAUAAU GCAU \

GUUAACUUAACGACUCUUAUUUAUACCCUGAAGAG UU ACGCCUUCUAUUG CGUA A

CC A ---- AAU

PC-5p-57811-35:

UGGGAAUUCUCAAAACUGCGGA

Identified by sequence similarity

Stem-loop structure:

G **A A C**  CUCA AAA

CAAUUGAAU GCUGAGAAUGAAUA**UGGGA UUCUC AAA UGCGGA**AGAUAAU GCAU \

GUUAACUUA CGACUCUUAUUUAUACCCU AAGAG UUU ACGCCUUCUAUUG CGUA A

G G C A ---- AAU

PC-5p-57811-36:

UGGGACUUCUCAAAACAGCGGA

Identified by sequence similarity

Stem-loop structure:

**A CA** CCCA AAA

GUACAUAUGAAUCGCUGAGAAUAAAUA**UGGGACUUCUC AAA GCGGA**AGAUAAU GCAU \

CAUGUAUACUUAGCGACUCUUAUUUAUACCCUGAAGAG UUU CGCCUUCUAUUG CGUA A

C AA ---- AAU

PC-5p-57811-37:

UGGGACUUUUCAAAACUGCGGA

Identified by sequence similarity

Stem-loop structure:

U A A UGAU **A C** AUCUCA AAA

UGUGA UGAU UAG AUAUAUAUGAAUCGC AAUAAAUA**UGGGACUUUUC AAA UGCGGA**AGA GCAU \

GCAUU AUUA AUU UAUGUAUACUUAGCG UUAUUUAUACCCUGAAAAG UUU ACGCUUUCU CGUA A

U - G UAAC C A AUUG-- AAU

PC-5p-57811-38:

UGGGACUUUUCAAAACUGCGGA

Identified by sequence similarity

Stem-loop structure:

**A C** CCCA AAA

GAAUAAAUA**UGGGACUUUUC AAA UGCGGA**AGAUAAU GCAU \

UUUAUUUAUACCCUGAAAAG UUU ACGCCUUCUAUUG CGUA A

C A ---- AAU

PC-5p-57811-39:

UGGGACUUCUGAAAACUGCGGA

Identified by sequence similarity

Stem-loop structure:

A G UU **C**- **AC** CCCA AAA

AUAUAU UGAAUCGCU AGAA AAUA**UGGGACUU UGAAA UGCGGA**AGAUAAU GCAU \

UAUAUA ACUUAGCGA UUUU UUAUACCCUGAA GCUUU ACGCCUUCUAUUG CGUA A

A A CU AA A- ---- AAU

PC-5p-57811-40:

UGGGACUUUUCAAAACUGCGGA

Identified by sequence similarity

Stem-loop structure:

**AC A C** CCCA GAA

AUAUGUAUCGCUGAGAAUAAAAA**UGGG UUUUC AAA UGCGGA**AGAUAAU GCAU \

UAUAUAUAGCGACUCUUAUUUUUACCC AAAAG UUU ACGCUUUCUAUUG CGUA A

AA G A ---- AAU

PC-5p-57811-41:

UGGGACUUUUCAAAACUGCGGA

Identified by sequence similarity

Stem-loop structure:

---- **A C A** CCCA CAAA

UAUAUGAAUCGCUGAGA AUA**UGGGACUUUUC AAA UGCGG** AGAUAAU GCA \

AUAUACUUAGCGACUCU UAUACCCUGAGAAG UUU ACGCC UCUAUUG CGU A

UAUU C A A ---- AAAU

PC-5p-57811-42:

UGGGACUUCUCAAAACUGUGAA

Identified by sequence similarity

Stem-loop structure:

UU G **CA C A**  CCCA AUAA

AUAUA UGAAU GCUGAGAAUAAAUA**UGGGACUUCU AAA UGUG** AAGAUAAU GCA A

UAUGU ACUUA CGACUCUUAUUUAUACCCUGAAGA UUU ACGC UUCUAUUG CGU A

UU G AC A C ---- AAAU

PC-5p-57811-43:

UGGGACUUCUCAAAAUUGCGGA

Identified by sequence similarity

Stem-loop structure:

A G **AA** CCCA AAA

ACAUAUGAAUC CUGA AAUAAAUA**UGGGACUUCUC AAUUGCGGA**AGAUAAU GCAU \

UGUAUACUUAG GACU UUAUUUAUACCCUGAAGAG UUAACGCCUUCUAUUA CGUA A

C A CG ---- AAU

PC-5p-57811-44:

UGGGACUUUUCAAAACUGCGGA

Identified by sequence similarity

Stem-loop structure:

C **A C** CCCA AAA

UA AUAUGAAUCGCUGAGAAUAAAUA**UGGGACUUUUC AAA UGCGGA**AGAUAAU GCAU \

AU UAUACUUAGCGACUCUUAUUUAUACCCUGAAGAG UUU ACGCCUUCUAUUG CGUA A

A C A ---- AAU

PC-5p-57811-45:

UGGGACUUCUCAAAACUGUGGA

Identified by sequence similarity

Stem-loop structure:

**A C** CCCA AAA

CAUAUAAUUGAAUCGCUGAGAAUAAAUA**UGGGACUUCUC AAA UGUGGA**AGAUAAU GCAU \

GUAUAUUAACUUAGCGACUCUUAUUUAUACCCUGAAGAG UUU ACGCCUUCUAUUG UGUA A

C A ---- AAU

PC-5p-57811-46:

UGGGACUUCUCAAAAUUGCGGA

Identified by sequence similarity

Stem-loop structure:

**C** CG UU U

UUGAAUCGCUGAGAAUAAAUA**UGGGA UUCUCAAAAUUGCGGA**AGAUAA CU UAU \

AACUUAGCGGCUCUUAUUUAUACCCU AAGAGUUUUGACGCCUUCUAUU GG GUA U

A AG UC U

PC-5p-57811-47:

UGGAACUUCUCAAAACUGCGGA

Identified by sequence similarity

Stem-loop structure:

A U **A A C** CCCA AAA

AUG AUAUGAAUCGCUGAGAAUAAA A**UGG** **ACUUCUC AAA UGCGGA**AGAUAAU GCAU \

UAU UAUACUUAGCGACUCUUAUUU UACC UGAAGAG UUU ACGCCUUCUAUUG CGUA A

G C C C A ---- AAU

PC-5p-57811-48:

UGAGACUUCUCAAAACUGCGAA

Identified by sequence similarity

Stem-loop structure:

**A A C A** UCUCCCA AAA

GCUGAGAAUAAAUA**UG** **GACUUCUC AAA UGCG A**AGAU GCAU \

CGACUCUUAUUUAUAC CUGAAGAG UUU ACGC UUCUA CGUA A

C C A C UUG---- AAU

PC-5p-57811-49:

UUGGACUUUUCAAAACUGCGGA

Identified by sequence similarity

Stem-loop structure:

A **U A C** CCCA AAA

UAUAUGAAUCGCUGAGAA AAAUA**U** **GGACUUUUC AAA UGCGGA**AGAUAAU GCAU \

GUAUACUUAGCGACUCUU UUUAUA CCUGAAAAG UUU ACGCUUUCUAUUG CGUA A

A C C A ---- AAU

PC-5p-57811-50:

UAGGACUUCUCAAAAUUGCGGA

Identified by sequence similarity

Stem-loop structure:

C- **A C C** C---- UUA

GUUAUA UAUAUAUGAAUUGCUGAGAAUAAAUA**U** **GGACUU UCAAAAUUG GGA**AGAUAA GCAU \

CAAUAU AUAUAUACUUAGCGACUCUUAUUUAUA CCUGAA AGUUUUGAC CCUUCUAUU CGUA U

AU C A A AGGGU UUU

PC-5p-57811-51:

UGGAACUUCUCAAAACUGCAGA

Identified by sequence similarity

Stem-loop structure:

G U **A** **A C A** CCCA AAA

ACAAUUGAAUCGCU AGAAUAAA A**UGG** **ACUUCUC AAA UGC GA**AGAUAAU GCAU \

UGUUAACUUAGCGA UCUUAUUU UACC UGAAGAG UUU ACG CUUCUAUUG CGUA A

A U C C A C ---- AAU

PC-5p-57811-52:

UGGGACUUUUCAAAACUGCCGA

Identified by sequence similarity

Stem-loop structure:

**C C** UCCA AAA

UUAAUUGAAUCGCUGAGAAUAAAUA**UGGGACUUUUCAAAA** **UGC GA**AGAUAAU GCAUA \

AGUUAACUUAGCGACUCUUAUUUAUACUCUGAAAAGUUUU ACG UUUCUAUUG CGUGU U

A C ---- AAA

PC-5p-57811-53:

UGGUACUUUUCAAAACUGCGGA

Identified by sequence similarity

Stem-loop structure:

UU A **A A C** CCCA AAA

AGAUACA UGAAUGGCACAGAAUGAAA **UGGU** **CUUUUC AAA UGCGGA**AGAUAAU GCAU \

UCUAUGU ACUUACCGUGUCUUACUUU GCCA GAAAAG UUU ACGCCUUCUAUUG CGUA A

UU C G C A ---- AAU

PC-5p-57811-54:

UGGGACUUCUCCAAAUUGCGGA

Identified by sequence similarity

Stem-loop structure:

A **C C** C---- UUA

UAUAUAUAUAU AAUCGCUGAGAAUAAAUA**UGGGA UUCUC AAAUUGCGGA**AGAUAA GCAU \

GUAUAUAUAUA UUAGCGACUCUUAUUUAUAUCCU AAGAG UUUGACGCCUUCUAUU CGUA U

C A C AGGGU UUU

PC-5p-57811-55:

UGGGACCUUUCAAAACUGCGGA

Identified by sequence similarity

Stem-loop structure:

**C A C**  GUCCCA AAA

AAUUGAAUCGCUGAGAAUAAAUA**UGGGAC UUUC AAA UGCGGA**AGAUA GCAU \

UUAACUUAGCGACUCUUAUUUAUACCCUG AAAG UUU ACGCCUUCUAU CGUA A

A C A UG---- AAU

PC-5p-57811-56:

UGGAACUUCUCAAAACUACGGA

Identified by sequence similarity

Stem-loop structure:

AA U **A A CUA** CCCA AAA

UCUA UAUGAAUCGCUGAGAAUAAA A**UGG ACUUCUC AAA CGGA**AGAUAAU GCAU \

AGAU AUACUUAGCGACUCUUAUUU UACC UGAAGAG UUU GCCUUCUAUUG CGUA A

-- U C C AAC ---- AAU

PC-5p-57811-57:

UGGGACUUCGUAAAACUGCGGA

Identified by sequence similarity

Stem-loop structure:

C **GUA C** CCCA AAA

UGAAU GCUGAGAAUAAAUA**UGGGACUUC AAA UGCGGA**AGAUAAU GCAU \

ACUUA CGACUCUUAUUUAUACCCUGAAG UUU ACGCCUUCUAUUG CGUA A

A AGC A ---- AAU

PC-5p-57811-58:

UGGGAAUUCUCAAAACUACGGA

Identified by sequence similarity

Stem-loop structure:

**AUU A CUA** CCCA AAA

AUAUGAAUCGCUGAGAAUAAAUA**UGGGA** **CUC AAA** **CGGA**AGAUAAU GCAU \

UAUACUUAGCGACUCUUAUUUAUACCCU GAG UUU GCCUUCUGUUG CGUA A

GAU C AAC ---- AAU

PC-5p-57811-59:

UGGGACUUCUCGAAAUUGCGGA

Identified by sequence similarity

Stem-loop structure:

C UG A U **GA C** C---- UUA

AUGAAU GC AGAAU AA A**UGG** **CUU UCGAAAUUGCGGA**AGAUAA GCAU \

UACUUA CG UCUUA UU UACC GAA AGUUUUGACGCCUUCUAUU CGUA U

C UG C U AG A AGGGU UUU

PC-5p-57811-60:

UGGGAUUUUUCAAAACUGCGGA

Identified by sequence similarity

Stem-loop structure:

**A CUG** CCCA AAA

UUGAAUUGCUGAGAAUAAAUA**UGGGAUUUUUC AAA CGGA**AGAUAAU GCAU \

AACUUAGCGACUCUUAUUUAUAUCCUGAAAAG UUU GCCUUCUAUUA CGUA A

C AAA ---- AGU

PC-5p-67443-1:

UCAAAACUGCGGAAGAUAAUCC

Identified by deep-sequencing

Stem-loop structure:

C G **A C** **CC**AA AAA

UAAUACAAUUGAAU GCUGAGAAUAAAUAUGGGAC UC**UC** **AAA UGCGGAAGAUAAU** GCAU \

GUUAUGUUAACUUA CGACUCUUAUUUAUAUCCUG AGAG UUU ACGCCUUCUAUUG CGUA A

A A C A ---- AAU

PC-5p-67443-2:

UCAAAACUGCGGAAGAUAAUCC

Identified by sequence similarity

Stem-loop structure:

**CA C** **CC**UA AAA

UGAAUCGCUGAGAAUAAAUAUGGGACUUC**U AAA UGCGGAAGAUAAU** GCAU \

ACUUAGCGACUCUUAUUUAUACCCUGAAGA UUU ACGCCUUCUAUUG CGUA A

AC A ---- AAU

PC-5p-67443-3:

UCAAAACUGCGGAAGAUAAUCC

Identified by sequence similarity

Stem-loop structure:

A **A C** **CC**CA AAA

CAAUU AAUGGCACAGAAUGAAAAUGGUCUUUU**UC AAA UGCGGAAGAUAAU** GCAU \

GUUAA UUACCGUGUCUUACUUUUACCAGGAAAAG UUU ACGCCUUCUAUUG CGUA A

C C A ---- AAU

PC-5p-67443-4:

UCAAAACUGCGGAAGAUAAUCC

Identified by sequence similarity

Stem-loop structure:

**A C C A** **CC**CA AAA

AAUUGAAUCGCUGAGAAUAAAUAUGGGACUUC**UC** **AAA UG GGAAG UAAU** GCAU \

UUAACUUAGUGACUCUUAUUUAUACCCUGAAGAG UUU AC CUUUC AUUG CGUA A

C A A G ---- AAU

PC-5p-67443-5:

UCAAAACUGCGGAAGAUAAUCC

Identified by sequence similarity

Stem-loop structure:

**C A C CC**CA CUA

CAAUUGAAUCGCUGAGAAUAAAUAUGGGAC **UCUC AAA UGCGGAAGAUAAU** GCAU \

GUUAACUUAGCGACUCUUAUUUAUACCCUG AGAG UUU ACGCCUUCUAUUG CGUA A

A C A ---- AAU

PC-5p-67443-6:

UCAAAACUGCGGAAGAUAAUCC

Identified by sequence similarity

Stem-loop structure:

- UA C A **A C** **CC**CA AAA

UAUAA UGGAUAUCAUA UGAAUGGCA AGAAUGAAA UGGUCCUUU**UC AAA UGCGGAAGAUAAU** GCAU \

AUAUU ACCUAUAGUAU ACUUACCGU UCUUACUUU GCCAGGAAAAG UUU ACGCCUUCUAUUG CGUA A

G -- A C C A ---- AAU

PC-5p-67443-7:

UCAAAACUGCGGAAGAUAAUCC

Identified by sequence similarity

Stem-loop structure:

**A C** **CC**CA AGA

GGUCCUUU**UC AAA UGCGGAAGAUAAU** GCAU \

CCAGGAAAAG UUU ACGCCUUCUAUUG CGUA A

C A ---- AAU

PC-5p-67443-8:

UCAAAACUGCGGAAGAUAAUCC

Identified by sequence similarity

Stem-loop structure:

AC A **A C** **CC**CA AAA

UACAUAUGAAUG ACAGAAUGAAA UGGUCCUUU**UC AAA UGCGGAAGAUAAU** GCAU \

AUGUAUACUUAU UGUCUUACUUU GCCAGGAAAAG UUU ACGCCUUCUAUUG CGUA A

CA C C A ---- AAU

PC-5p-67443-9:

UCAAAACUGCGGAAGAUAAUCC

Identified by sequence similarity

Stem-loop structure:

G **A C CC**CA AAA

UAUAUGAAUCGCUGA AAUAAAUAUGGGACUUU**UC AAA UGCGGAAGAUAAU** GCAU \

GUAUACUUAGCGACU UUAUUUAUACCCUGAAAAG UUU ACGCCUUCUAUUG CGUA A

A C A ---- AAU

PC-5p-67443-10:

UCAAAACUGCGGAAGAUAAUCC

Identified by sequence similarity

Stem-loop structure:

**A C CC**CA AAA

GGUCCUUU**UC AAA UGCGGAAGAUAAU** GCAU \

CCAGGAAAAG UUU ACGCCUUCUAUUG CGUA A

C A ---- AAU

PC-5p-67443-11:

UCAAAACUGCGGAAGAUAAUCC

Identified by sequence similarity

Stem-loop structure:

GU **A C A CC**CA AAA

AUAUGAAUCGCUGAGAAUAAAUAUG ACUUC**UC AAA UGCGGAAG UAAU**  GCAU \

UAUACUUAGCGACUCUUAUUUAUAC UGAAGAG UUU AUGCCUUC AUUG CGUA A

UC C A G ---- AAU

PC-5p-67443-12:

UCAAAACUGCGGAAGAUAAUCC

Identified by sequence similarity

Stem-loop structure:

C C A **A C CC**CA AAA

UGGAA UA AAUUGAAUC CUGAGAAUAAAUAUGGGACUUC**UC AAA UGCGGAAGAUAAU** GCAU \

ACCUU AU UUAACUUAG GACUCUUAUUUAUACCCUGAAGAG UUU ACGCCUUCUAUUA CGUA A

U - C C A ---- AAU

PC-5p-67443-13:

UCAAAACUGCGGAAGAUAAUCC

Identified by sequence similarity

Stem-loop structure:

**A C CC**CA AAA

GUAGUAUAUAUGAAUCGCUGAGAAUAAAUAUGGGACUUC**UC AAA UGCGGAAGAUAAU** GCAU \

CAUCAUAUAUACUUAGCGACUCUUAUUUAUACCCUGAAGAG UUU ACGCCUUCUAUUG CGUA A

C A ---- AAU

PC-5p-67443-14:

UCAAAACUGCGGAAGAUAAUCC

Identified by sequence similarity

Stem-loop structure:

UU A A **A C CC**CA AAA

AGAUACA UGAAUGGCACAGAAUGAAA UGGU CUUU**UC AAA UGCGGAAGAUAAU** GCAU \

UCUAUGU ACUUACCGUGUCUUACUUU GCCA GAAAAG UUU ACGCCUUCUAUUG CGUA A

UU C G C A ---- AAU

PC-5p-67443-15:

UCAAAACUGCGGAAGAUAAUCC

Identified by sequence similarity

Stem-loop structure:

A **A C CC**CA AAA

AAUUGAAUGGCACAGAAUGAAA UGGUCCUUU**UC AAA UGCGGAAGAUAAU** GCAU \

UUAACUUACCGUGUCUUACUUU GCCAGGAAAAG UUU ACGCCUUCUAUUG CGUA A

C C A ---- AAU

PC-5p-67443-16:

UCAAAACUGCGGAAGAUAAUCC

Identified by sequence similarity

Stem-loop structure:

U **A C CC**CA AAA

AUAUAUAUGAA CGCUGAGAAUAAAUAUGGGACUUC**UC AAA UGCGGAAGAUAAU** GCAU \

UAUAUAUACUU GCGACUCUUAUUUAUACCUUGAAGAG UUU ACGCCUUCUAUUG CGUA A

U C A ---- AAU

PC-5p-67443-17:

UCAAAACUGCGGAAGAUAAUCC

Identified by sequence similarity

Stem-loop structure:

A A **A C C** AGCAUA

AAUG GCAUAUGAAUGGCACAGAAUGAAA UGGUCCUUU**UC AAA UGCGGAAGAUAAU C**C \

UUAU UGUAUACUUACCGUGUCUUACUUU GCCAGGAAAAG UUU ACGCCUUCUAUUG GG A

A C C A C AAAUAA

PC-5p-67443-18:

UCAAAACUGCGGAAGAUAAUCC

Identified by sequence similarity

Stem-loop structure:

**UC AC CC** GAAUA

UUCAUAUGAAUGGCACAGAAUGAAAAUGGUCCUUU **AAA UGCGGAAGAUAAU** CA A

AAGUAUACUUACCGUGUCUUACUUUUACCAGGAAG UUU ACGCCUUCUAUUG GU A

GC A- C- AAAUA

PC-5p-67443-19:

UCAAAACUGCGGAAGAUAAUCC

Identified by sequence similarity

Stem-loop structure:

C G C **A C CC**CA AAA

AUAUAUAUGAAU GCUGAGAAUAAAUAU GGACUU **UC AAA UGCGGAAGAUAAU** GCAU \

UAUAUAUACUUA CGACUCUUAUUUAUA UCUGAA AG UUU ACGCCUUCUAUUG CGUA A

A A A C A ---- AAU

PC-5p-67443-20:

UCAAAACUGCGGAAGAUAAUCC

Identified by sequence similarity

Stem-loop structure:

A **A C CC**CA AAA

CAUAUGAAUGGCACAGAAUGAAA UGGUCCUUU**UC AAA UGCGGAAGAUAAU** GCAU \

GUAUACUUACCGUGUCUUACUUU GCCAGGAAAAG UUU ACGCCUUCUAUUG CGUA A

C C A ---- AAU

PC-5p-67443-21:

UCAAAACUGCGGAAGAUAAUCC

Identified by sequence similarity

Stem-loop structure:

U C **A C** **AAUCC**CAGCAUAAAAUAAACAAUA

AUAUGAAUGGCACA AAUGAAAAUGGUC UUU**UC** **AAA UGCGGAAGAU** A

UAUACUUACCGUGU UUACUUUUACCAG AAAAG UUU ACGCCUUCUA U

C A G A GCAAUAGAAGAAUAUAAUAACAAA

PC-5p-67443-22:

UCAAAACUGCGGAAGAUAAUCC

Identified by sequence similarity

Stem-loop structure:

**A C CC**CA AAA

GAAUCGCUGAGAAUAAAUAUGGGACUUC**UC** **AAA UGCGGAAGAUAAU** GCAU \

CUUAGCGACUCUUAUUUAUACCCUGAAGAG UUU ACGCCUUCUAUUG CGUA A

G A ---- AAU

PC-5p-67443-23:

UCAAAACUGCGGAAGAUAAUCC

Identified by sequence similarity

Stem-loop structure:

C **A C CC**CA AAA

AAUUGAAU GCUGAGAAUAAAUAUGGGACUUC**UC** **AAA UGCGGAAGAUAAU**  GCAU \

UUAACUUA CGACUCUUAUUUAUACCCUGAGGAG UUU ACGCCUUCUAUUG CGUA A

A C A ---- AAU

PC-5p-67443-24:

UCAAAACUGCGGAAGAUAAUCC

Identified by sequence similarity

Stem-loop structure:

**A C CC**CA AAA

AAUUGAAUCGCUGAGAAUAAAUAUGGGACUUC**UC** **AAA UGCGGAAGAUAAU** GCAU \

UUAACUUAGCGACUCUUAUUUAUACCCUGAAGAG UUU ACGCCUUCUAUUG CGUA A

C A ---- AAU

PC-5p-67443-25:

UCAAAACUGCGGAAGAUAAUCC

Identified by sequence similarity

Stem-loop structure:

U A **A C CC**CA AAA

CAUAUGAAUG CACAGAAUGAAA UGGUCCUUU**UC** **AAA UGCGGAAGAUAAU** GCAU \

GUAUACUUAC GUGUCUUACUUU GCCAGGAAAAG UUU ACGCCUUCUAUUG CGUA G

C C C A ---- AAU

PC-5p-67443-26:

UCAAAACUGCGGAAGAUAAUCC

Identified by sequence similarity

Stem-loop structure:

**A C CC**CA AAA

GAAUAAAUAUGGGACUUU**UC** **AAA UGCGGAAGAUAAU** GCAU \

UUUAUUUAUACCCUGAAAAG UUU ACGCCUUCUAUUG CGUA A

C A ---- AAU

PC-5p-67443-27:

UCAAAACUGCGGAAGAUAAUCC

Identified by sequence similarity

Stem-loop structure:

GAGAA C **A C CC**CA AAA

GAAUCGCUGAGA AUAAAUAUGGGA UUC**UC** **AAA UGCGGAAGAUAAU** GCAU \

CUUAGCGACUCU UAUUUAUACCCU AAGAG UUU ACGCCUUCUAUUG CGUA A

----- U C A ---- AAU

PC-5p-67443-28:

UCAAAACUGCGGAAGAUAAUCC

Identified by sequence similarity

Stem-loop structure:

A A **A C CC**CA AAA

GAA UAUAUGAAUGGCACAGAAUGAAA UGGUCCUUU**UC** **AAA UGCGGAAGAUAAU** GCAU \

CUU GUAUACUUACCGUGUCUUACUUU GCCAGGAAAAG UUU ACGCCUUCUAUUG CGUA A

A C C A ---- AAU

PC-5p-67443-29:

UCAAAACUGCGGAAGAUAAUCC

Identified by sequence similarity

Stem-loop structure:

AA A A C **A C CC**CA AAA

UACA UGAAUGGCACAGAAU AAA UGGU CUUU**UC** **AAA UGCGGAAGAUAAU** GCAU \

AUGU ACUUACCGUGUCUUA UUU GCCA GAAAAG UUU ACGCCUUCUAUUG CGUA A

AA C C A C A ---- AAU

PC-5p-67443-30:

UCAAAACUGCGGAAGAUAAUCC

Identified by sequence similarity

Stem-loop structure:

**A C CC**CA AAA

UUGAAUCUCUGAGAAUAAAUAUGGGACUUC**UC** **AAA UGCGGAAGAUAAU** GCAU \

GACUUAGGGACUCUUAUUUAUACCCUGAAGAG UUU ACGCCUUCUAUUG CGUA A

C A ---- AAU

PC-5p-67443-31:

UCAAAACUGCGGAAGAUAAUCC

Identified by sequence similarity

Stem-loop structure:

A **A C CC**CA AAA

UGACAUAUGAAUGGCACAGAAUGAAA UGGUCCUUU**UC** **AAA UGCGGAAGAUAAU** GCAU \

ACUGUAUACUUACCGUGUCUUACUUU GCCAGGAAAAG UUU ACGCCUUCUAUUG CGUA A

C C A ---- ACU

PC-5p-67443-32:

UCAAAACUGCGGAAGAUAAUCC

Identified by sequence similarity

Stem-loop structure:

**AA C C** AGCAUA

CUUU**UC** **AA UGCGGAAGAUAAU C**C \

GAAAAG UU ACGCCUUCUAUUG GG A

CA A C AAAUAA

PC-5p-67443-33:

UCAAAACUGCGGAAGAUAAUCC

Identified by sequence similarity

Stem-loop structure:

**A C** **CC**CA AAA

UGAAAAUGGUCCUUU**UC** **AAA UGCGGAAGAUAAU** GCAU \

ACUUUUACCAGGAAAAG UUU ACGCCUUCUAUUG CGUA A

C A ---- AAU

PC-5p-67443-34:

UCAAAACUGCGGAAGAUAAUCC

Identified by sequence similarity

Stem-loop structure:

U **A C CC**CA AAA

AUAUGAAUCGCU AGAAUAAAUAUGGGACUUC**UC** **AAA UGCGGAAGAUAAU**  GCAU \

UAUACUUAGCGA UCUUAUUUAUACCCUGAAGAG UUU ACGCCUUUUAUUG CGUA A

C C A ---- AAU

PC-5p-67443-35:

UCAAAACUGCGGAAGAUAAUCC

Identified by sequence similarity

Stem-loop structure:

AC **A C CC**CA GAA

AUAUGUAUCGCUGAGAAUAAAAAUGGG UUU**UC** **AAA UGCGGAAGAUAAU** GCAU \

UAUAUAUAGCGACUCUUAUUUUUACCC AAAAG UUU ACGCUUUCUAUUG CGUA A

AA G A ---- AAU

PC-5p-67443-36:

UCAAAACUGCGGAAGAUAAUCC

Identified by sequence similarity

Stem-loop structure:

A U **A C CC**CA AAA

UAUAUGAAUCGCUGAGAA AAAUAU GGACUUU**UC** **AAA UGCGGAAGAUAAU**  GCAU \

GUAUACUUAGCGACUCUU UUUAUA CCUGAAAAG UUU ACGCUUUCUAUUG CGUA A

A C C A ---- AAU

PC-5p-67443-37:

UCAAAACUGCGGAAGAUAAUCC

Identified by sequence similarity

Stem-loop structure:

**AA C CC**CA AAA

ACAAUUGAAUCGCUGAGAAUAAAUAUGGGACUUC**UC** **AA UGCGGAAGAUAAU** GCAU \

UGUUAACUUAGCGACUCUUAUUUAUACUCUGAAGAG UU ACGCCUUCUAUUG CGUA A

CC A ---- AAU

PC-5p-67443-38:

UCAAAACUGCGGAAGAUAAUCC

Identified by sequence similarity

Stem-loop structure:

CA AAC- **A C CC**CA AAA

UAUACAUA UAUGAAUCGCUGAGAAUAAAUAUGG UU**UC** **AAA UGCGGAAGAUAAU** GCAU \

AUAUGUAU AUACUUAGCGACUCUUAUUUAUACC GAAG UUU ACGCCUUUUAUUG CGUA A

-- CUAA C A ---- AAU

PC-5p-67443-39:

UCAAAACUGCGGAAGAUAAUCC

Identified by sequence similarity

Stem-loop structure:

AAC- **A C CC**CA AAA

GUAAAUAUGG UU**UC** **AAA UGCGGAAGAUAAU**  GCAU \

UAUUUAUACC GAAG UUU ACGCCUUUUAUUG CGUA A

CUAA C A ---- AAU

PC-5p-67443-40:

UCAAAACUGCGGAAGAUAAUCC

Identified by sequence similarity

Stem-loop structure:

---- **A C A CC**CA CAAA

UAUAUGAAUCGCUGAGA AUAUGGGACUUU**UC** **AAA UGCGG AGAUAAU** GCA \

AUAUACUUAGCGACUCU UAUACCCUGAGAAG UUU ACGCC UCUAUUG CGU A

UAUU C A A ---- AAAU

PC-5p-67443-41:

UCAAAACUGCGGAAGAUAAUCC

Identified by sequence similarity

Stem-loop structure:

**A C CC**CA AAA

UGAAUCGCUGAGAAUAAAUAUGGGACUUC**UC** **AAA UGCGGAAGAUAAU** GCAU \

ACUUAGCGACUCUUAUUUAUACCCUGAAGAG UUU ACGCCUUCUAUUG CGUA A

C A ---- AAU

PC-5p-67443-42:

UCAAAACUGCGGAAGAUAAUCC

Identified by sequence similarity

Stem-loop structure:

U A **A C CC**CA AAA

AUAUGAAUG CACAGAAUGAAA UGGUCCUUU**UC** **AAA UGCGGAAGAUAAU** GCAU \

UAUACUUAC GUGUCUUACUUU GCCAGGAAAAG UUU ACGCCUUCUAUUG CGUA A

C C C A ---- AAU

PC-5p-67443-43:

UCAAAACUGCGGAAGAUAAUCC

Identified by sequence similarity

Stem-loop structure:

- G **A C CC**CA AAA

CAU UAUAUGAAUCGCU AGAAUAAAUAUAGGACUUU**UC** **AAA UGCGGAAGAUAAU** GCAU \

GUA GUAUACUUAGCGA UCUUAUUUAUAUCCUGAGAGG UUU ACGCCUUCUAUUA CGUA A

U A C A ---- AAU

PC-5p-67443-44:

UCAAAACUGCGGAAGAUAAUCC

Identified by sequence similarity

Stem-loop structure:

**A C CC**CA AAA

AUAUGAAUCGCUGAGAAUAAAUAUGGGACUUU**UC** **AAA UGCGGAAGAUAAU** GCAU \

UAUACUUAGCGACUCUUAUUUAUACCCUGAAGAG UUU ACGCCUUCUAUUG CGUA A

C A ---- AAU

PC-5p-67443-45:

UCAAAACUGCGGAAGAUAAUCC

Identified by sequence similarity

Stem-loop structure:

UU A **A C CC**CA AAA

UUGUACA UGAAUGGCACAGAAUGAAA UGGUCCUUU**UC** **AAA UGCGGAAGAUAAU** GCAU \

AACAUGU ACUUACCGUGUCUUACUUU GCCAGGAAAAG UUU ACGCCUUCUAUUG CGUA A

UU C C A ---- AAU

PC-5p-67443-46:

UCAAAACUGCGGAAGAUAAUCC

Identified by sequence similarity

Stem-loop structure:

**A C CC**AA AAA

GGUACAUAUGAAUGGCACAGAAUGAAAAUGGUCCUUU**UC** **AAA UGCGGAAGAUAAU** GCAU \

UCAUGUAUACUUACCGUGUCUUACUUUUACCAGGAAAAG UUU AUGCCUUCUAUUG CGUA A

C A ---- AAU

PC-5p-67443-47:

UCAAAACUGCGGAAGAUAAUCC

Identified by sequence similarity

Stem-loop structure:

A **A C CC**CA AAA

UGAAUGGCACAGAAUGAAA UGGUCCUUU**UC** **AAA UGCGGAAGAUAAU** GCAU \

ACUUACCGUGUCUUACUUU GCCAGGAAAAG UUU ACGCCUUCUAUUG CGUA A

C C A ---- AAU

PC-5p-67443-48:

UCAAAACUGCGGAAGAUAAUCC

Identified by sequence similarity

Stem-loop structure:

G AC A UC **A C CC**CA AAA

AUGAAU GC AGAAUGAA AUGG CUUU**UC** **AAA UGCGGAAGAUAAU** GCAU \

UACUUA CG UCUUAUUU UACC GAAGAG UUU ACGCCUUCUAUUG CGUA A

G AC A CU C A ---- AAU

PC-5p-67443-49:

UCAAAACUGCGGAAGAUAAUCC

Identified by sequence similarity

Stem-loop structure:

U A **A C CC**CAC AAA

GUUG UAAUUGAAUGGCACAGAAUGAAA UGGUCCUUU**UC** **AAA UGCGGAAGAUAAU** CAU \

CAAC GUUAACUUACCGUGUCUUACUUU GCCAGGAAAAG UUU ACGCCUUCUAUUG GUA A

U C C A C---- AAU

PC-5p-67443-50:

UCAAAACUGCGGAAGAUAAUCC

Identified by sequence similarity

Stem-loop structure:

C A U **A C CC**CA AAA

UAUAUGAAUGG ACAGAAUGAAA UGGUCC UU**UC** **AAA UGCGGAAGAUAAU** GCAU \

AUAUACUUACC UGUCUUACUUU GCCAGG AAAG UUU ACGCCUUCUAUUG CGUA A

U C U C A ---- AAU

PC-5p-67443-51:

UCAAAACUGCGGAAGAUAAUCC

Identified by sequence similarity

Stem-loop structure:

**C CC**CA AAA

UCGCUGAGAAUAAAUAUGGGACUUC**UCAAAA UGCGGAAGAUAAU** GCAU \

AGCGACUCUUAUUUAUACCCUGAAGAGUUUU ACGCCUUCUAUUG CGUA A

A ---- AAU

PC-5p-67443-52:

UCAAAAUUGCGGAAGAUAACGC

Identified by sequence similarity

Stem-loop structure:

**C**---- UUA

UGGAUCGCUGAGAAUAAAUAUGGGACUUC**UCAAAAUUGCGGAAGAUAA** **GC**AU \

ACUUAGCGACUCUUAUUUAUACCCUGAAGAGUUUUGACGCCUUCUAUU CGUA U

AGGGU UUU

PC-5p-67443-53:

UCAAAACUGCGGAAGAUAAUCC

Identified by sequence similarity

Stem-loop structure:

**A CUG CC**CA AAA

UUGAAUUGCUGAGAAUAAAUAUGGGAUUUU**UC AAA CGGAAGAUAAU** GCAU \

AACUUAGCGACUCUUAUUUAUAUCCUGAAAAG UUU GCCUUCUAUUA CGUA A

C AAA ---- AGU

PC-5p-67443-54:

UCAAAACUGCGGAAGAUAAUCC

Identified by sequence similarity

Stem-loop structure:

G C **C**---- UUA

AUACAUAUGAAUG CACAGAAUGAAAG GGUCCUUU**UCGAAAUUGCGGAAGAUAA** GCAU \

UAUGUAUACUUAC GUGUCUUACUUUU CCAGGAAAAGUUUUGACGCCUUCUAUU CGUA U

A A AGGGU UUU

PC-5p-67443-55:

UCAAAACUGCGGAAGAUAAUCC

Identified by sequence similarity

Stem-loop structure:

U A **A C CC**CA AAA

UAUACAUAUGAAUG CACAGAAUGAAA UGGUCCUUU**UC** **AAA UGCGGAAGAUAAU** GCAU \

GUAUGUAUACUUAC GUGUCUUACUUU GCCAGGAAAAG UUU ACGCCUUCUAUUG CGUA A

C C C A ---- AAU

PC-5p-67443-56:

UCAAAACUGCGGAAGAUAAUCC

Identified by sequence similarity

Stem-loop structure:

A **A C CC**CA GAA

AUAUAUGAAUGGCACAGAAUGAAA UGGUCCUUU**UC** **AAA UGCGGAAGAUAAU** GCAU \

UAUAUACUUACCGUGUCUUACUUU GCCAGGAAAAG UUU ACGCCUUCUAUUG CGUA A

C C A ---- AAU

PC-5p-67443-57:

UCAAAACUGCGGAAGAUAAUCC

Identified by sequence similarity

Stem-loop structure:

AAA **A C CC**CA AAA

ACAAUUGAAUGGCACAGAAUGA UGGUCCUUU**UC** **AAA UGCGGAAGAUAAU** GCAU \

UGUUAACUUACCGUGUCUUACU GCCAGGAAAAG UUU ACGCCUUCUAUUG CGUA A

CUC C A ---- AAU

PC-5p-67443-58:

UCAAAACUGCGGAAGAUAAUCC

Identified by sequence similarity

Stem-loop structure:

A **A C CC**CA AAA

CAUAUGAAUGGCACAGAAUGAAA UGGUCCUUU**UC** **AAA UGCGGAAGAUAAU** GCAU \

GUAUACUUACCGUGUCUUACUUU ACCAGGAAAAG UUU ACGCCUUCUAUUG CGUA A

C C A ---- AAU

PC-5p-67443-59:

UCAAAACUGCGAAAGAUAAUCC

Identified by sequence similarity

Stem-loop structure:

U--- G UC **A C AAUCC**CA AAA

UUGUGUAU UGAAUC CUGAGAAUAAAUAUGGG UUU**UC** **AAA UGCGAAAGAU** GCAU \

AAUAUAUA ACUUAG GACUCUUAUUUAUACCC AAAAG UUU ACGCUUUCUA CGUA A

CUUU A UA C A AUG---- AAU

PC-5p-67443-60:

UCAAAACUGCGGAAGAUAAUCC

Identified by sequence similarity

Stem-loop structure:

**CC**CA AAA

AUAUGAAUCGCUGAGAAUAAAUAUGGGACUUC**UCAAAACUGCGGAAGAUAAU** GCAU \

UAUACUUAGCGACUCUUAUUUAUACCCUGAAGAGUUUUGACGCCUUCUAUUG CGUA A

---- AAU

PC-5p-67443-61:

UCAAAACUGCGGAAGAUAACGC

Identified by sequence similarity

Stem-loop structure:

**C**---- UUA

AUAUGAAUCGCUGAGAAUAAAUAUGGGACUUC**UCAAAACUGCGGAAGAUAA** **GC**AU \

UAUACUUAGCGACUCUUAUUUAUACCCUGAAGAGUUUUGACGCCUUCUAUU CGUA U

AGGGU UUU

PC-5p-67443-62:

UCAAAACUGCGGAAGAUAAUCC

Identified by sequence similarity

Stem-loop structure:

C **C CC**CA AUAAA

UUGAAUCGC GAGAAUAAAUAUGGGAUUUC**UCAAAA** **UGCGGAAGAUAAU** GC \

AACUUAGCG CUCUUAUUUAUACCCUGAAGAGUUUU ACGCCUUCUAUUG CG A

A A ---- AAAAU

PC-5p-67443-63:

UCAAAAUUGCGGAAGAUAACGC

Identified by sequence similarity

Stem-loop structure:

C **CG** UU U

UUGAAUCGCUGAGAAUAAAUAUGGGA UUC**UCAAAAUUGCGGAAGAUAA** **C**U UAU \

AACUUAGCGGCUCUUAUUUAUACCCU AAGAGUUUUGACGCCUUCUAUU GG GUA U

A AG UC U

PC-5p-67443-64:

UCAAAACUGCGGAAGAUAAUCC

Identified by sequence similarity

Stem-loop structure:

C **A C CC**CA AAA

CAAUUGAAUGGCACAGAAUGAAAAUGG CCUUU**UC** **AAA UGCGGAAGAUAAU** GCAU \

GUUAACUUACCGUGUCUUACUUUUACC GGAAAAG UUU ACGCCUUCUAUUG CGUA A

A C A ---- AAU

PC-5p-67443-65:

UCAAAACUGCGGAAGAUAAUCC

Identified by sequence similarity

Stem-loop structure:

A U A **A C CC**CA AAA

AUG AUAUGAAUCGCUGAGAAUAAA AUGG ACUUC**UC** **AAA UGCGGAAGAUAAU** GCAU \

UAU UAUACUUAGCGACUCUUAUUU UACC UGAAGAG UUU ACGCCUUCUAUUG CGUA A

G C C C A ---- AAU

PC-5p-67443-66:

UCAAAACUGCGGAAGAUAAUCU

Identified by sequence similarity

Stem-loop structure:

A **A C CU**CA AAA

GCUGAGAAUGAAUAUGGGA UUC**UC** **AAA UGCGGAAGAUAAU** GCAU \

CGACUCUUAUUUAUACCCU AAGAG UUU ACGCCUUCUAUUG CGUA A

G C A ---- AAU

PC-5p-67443-67:

UCAAAACUGCGGAAGAUAAUCA

Identified by sequence similarity

Stem-loop structure:

UU C **A C CA**CA AAA

UAUA UGAAUUGCUGAGAA AAAUAUGGGACUUC**UC** **AAA UGCGGAAGAUAAU** GCAU \

AUGU ACUUAGCGACUCUU UUUAUACCCUGAAGAG UUU ACGCCUUCUAUUG CGUA A

UU A C A ---- AAU

PC-5p-67443-68:

UCAAAACUGCGGAAGAUAAUCU

Identified by sequence similarity

Stem-loop structure:

A **A C CU**CA AAA

ACAUAUGAAUGGCACAGAAUGAAA UGGUCCUUU**UC** **AAA UGCGGAAGAUAAU**  GCAU \

UGUAUACUUACCGUGUCUUACUUU GCCAGGAAGAG UUU ACGCCUUCUAUUG CGUA A

C C A ---- AAU

PC-5p-67443-69:

UCAAAACUGCGGAAGAUAAUCA

Identified by sequence similarity

Stem-loop structure:

**C CA**CA AAA

UGGAUCGCUGAGAAUAAAUAUGGGACUUC**UCAAAA** **UGCGGAAGAUAAU** GCAU \

ACUUAGCGACUCUUAUUUAUACCCUGAAGAGUUUU ACGCCUUCUAUUG CGUA A

A ---- AAU

PC-5p-67443-70:

UCAAAAUUGCGGAAGAUAACGC

Identified by sequence similarity

Stem-loop structure:

A - UUUAU

GAUUG AUCGCUGAGAAUAAAUAUGGGACUUC**UCAAAAUUGCGGAAGAUAA** **CGC**A U

UUGAC UAGCGACUCUUAUUUAUACCCUGAAGAGUUUUGACGCCUUCUAUU GUGU U

C A CGUAU

PC-5p-67443-71:

UCAAAACUGCGGAAGAUAAUCU

Identified by sequence similarity

Stem-loop structure:

A **A C CU**CA AAA

UGGACAAUUGAAUGGCACAGAAUGAAA UGGUCCUUU**UC** **AAA UGCGGAAGAUAAU** GCAU \

ACCUGUUAACUUACCGUGUCUUACUUU GCCAGGAAAAG UUU ACGCCUUCUAUUG CGUA A

C C A ---- AAU

PC-5p-67443-72:

UCAAAACUGCGGAAGAUAAUUC

Identified by sequence similarity

Stem-loop structure:

**A C UC**CA AAA

UGAAUCGCUGAGAAUAAAUAUGGGACUUU**UC** **AAA UGCGGAAGAUAAU** GCAU \

ACUUAGCGACUCUUAUUUAUACUCUGAAAAG UUU ACGCCUUCUAUUG CGUA A

C A ---- AAU

PC-5p-67443-73:

UGAAAACUGCGGAAGAUAAUCC

Identified by sequence similarity

Stem-loop structure:

G UU C- **AC CC**CA AAA

UGAAUCGCU AGAA AAUAUGGGACUU UGAAA UGCGGAAGAUAAU GCAU \

ACUUAGCGA UUUU UUAUACCCUGAA GCUUU ACGCCUUCUAUUG CGUA A

A CU AA A- ---- AAU

PC-5p-67443-74:

UCAAAACUGCGGAAGAUAAUAA

Identified by sequence similarity

Stem-loop structure:

**A C AA**CA AAA

UUUCAAUUGAAUGGCACAGAAUGAAAAUGGUCCUUU**UC** **AAA UGCGGAAGAUAAU** GCAU \

AAAGUUAACUUACCGUGUCUUACUUUUACCAGGAAAAG UUU ACGCCUUCUAUUG CGUA A

C A ---- AAU

PC-5p-67443-75:

GUAAAACUGCGGAAGAUAAUCC

Identified by sequence similarity

Stem-loop structure:

C GUA C CCCA AAA

UGAAU GCUGAGAAUAAAUAUGGGACUUC **AAA UGCGGAAGAUAAU** GCAU \

ACUUA CGACUCUUAUUUAUACCCUGAAG UUU ACGCCUUCUAUUG CGUA A

A AGC A ---- AAU

PC-5p-67443-76:

UCAAAAACUGCGGAAGAUAAUC

Identified by sequence similarity

Stem-loop structure:

UU A - **C AAC CC**CA AAA

GAUAUA UGAAUGGCACAGAAUGAAA UGG CCUUUU**U** **AAA UGCGGAAGAUAAU** GCAU \

UUAUAU ACUUACCGUGUCUUACUUU GCC GGAAAAG UUU ACGCCUUCUAUUG CGUA A

UU C A C A-- ---- AAU

PC-5p-67443-77:

UUCAAAACUGCGGAAGAUAAAC

Identified by sequence similarity

Stem-loop structure:

A U **A C ACC**CA AAA

UAUAUGAAUC CUGAGA UAAAUAUGGGACUUU**UC** **AAA UGCGGAAGAUAA** GCAU \

GUAUACUUAG GACUCU AUUUAUAUCCUGAAAAG UUU ACGCCUUCUAUU CGUA A

C U C A G---- AAU

PC-5p-67443-78:

UUCAAAACUGCGGAAGAUAGUC

Identified by sequence similarity

Stem-loop structure:

GC- U C **A C** **GUCC**CA AAA

UGAAUGGCAUAGAAUGAAA GA CCU U**UC** **AAA** **UGCGGAAGAUA** GCAU \

ACUUACCGUGUCUUACUUU CU GGA AAG UUU ACGCCUUCUAU CGUA A

AAC - A C A UG---- AAU

PC-5p-67443-79:

UCAAAACUGCGAAAGAUAAUCC

Identified by sequence similarity

Stem-loop structure:

A A **A C A** **CC**CAGCAUA

AUAUGAAUGGCACAGAAUGAAA UG UCCUUU**UC** **AAA UGCG AAGAUAAU** \

UAUACUUACCGUGUCUUACUUU AC AGGAAAAG UUU ACGC UUCUAUUG A

C C C A C CAUAAAUAA

PC-5p-67443-80:

UCAAAACUGCGGAAUAUAAUCC

Identified by sequence similarity

Stem-loop structure:

**A C U CC**CA AAA

AUAUGAAUGGCACAGAAUGAAAAUGGUUCUUU**UC** **AAA UGCGGAA AUAAU** GCAU \

UAUACUUACCGUGUCUUACUUUUACCAGGAAAAG UUU ACGCCUU UAUUG CGUA A

C U C ---- AAU

PC-5p-67443-81:

UCAAAACUGCGAAAGAUAAUCC

Identified by sequence similarity

Stem-loop structure:

**A C A CCCA** AAA

AUACAUAUGAAUGGCACAGAAUGAAAAUGGUCCUUU**UC** **AAA UGCG AAGAUAAU** GCAU \

UAUGUAUACUUACCGUGUCUUACUUUUGCCAGGAAAAG UUU ACGC UUCUAUUA CGUA A

C A C ---- AAU

PC-5p-67443-82:

UCAAAACUGCGGGAGAUAAUCC

Identified by sequence similarity

Stem-loop structure:

**A C** **CC**CA AAA

UAUAUAUGAAUCGCUGAGAAUAAAUAUGGGACUUC**UC** **AAA UGCGGGAGAUAAU** GCAU \

AUAUAUACUUAGCGACUUUUAUUUAUACCCUGAAGAG UUU ACGCCUUCUAUUG CGUA A

C A ---- AAU

PC-5p-67443-83

UCAAAACAGCGGAAGAUAAUCC

Identified by sequence similarity

Stem-loop structure:

**A CA** **CC**CA AAA

GUACAUAUGAAUCGCUGAGAAUAAAUAUGGGACUUC**UC** **AAA GCGGAAGAUAAU** GCAU \

CAUGUAUACUUAGCGACUCUUAUUUAUACCCUGAAGAG UUU CGCCUUCUAUUG CGUA A

C AA ---- AAU

PC-5p-67443-84:

UCAUAACUGCGGAAGAUAAUCC

Identified by sequence similarity

Stem-loop structure:

A **AU C** **CC**CA AAA

AUAUGAAUGGCACAGAAUGAAA UGGUCCUUU**UC** **AA UGCGGAAGAUAAU** GCAU \

UAUACUUACCGUGUCUUACUUU GCCAGGAAAAG UU ACGCCUUCUAUUG CGUA A

C CU A ---- AAU

PC-5p-67443-85:

UCAAAACUGCAGAAGAUAAUCC

Identified by sequence similarity

Stem-loop structure:

G U A **A C A** **CC**CA AAA

ACAAUUGAAUCGCU AGAAUAAA AUGG ACUUC**UC** **AAA UGC GAAGAUAAU** GCAU \

UGUUAACUUAGCGA UCUUAUUU UACC UGAAGAG UUU ACG CUUCUAUUG CGUA A

A U C C A C ---- AAU

PC-5p-67443-86:

UCAGAACUGCGGAAGAUAAUCC

Identified by sequence similarity

Stem-loop structure:

A **AG C** **CC**CA AAA

UCAAUUGAAUGGCACAGAAUGAAA UGGUCCUUU**UC** **AA UGCGGAAGAUAAU** GCAU \

AGUUAACUUACCGUGUCUUACUUU GCCAGGAAAAG UU ACGCCUUCUAUUG CGUA A

C CU A ---- AAU

PC-5p-67443-87:

UCAAAACUGCGGACGAUAAUCC

Identified by sequence similarity

Stem-loop structure:

G **A C C** **CC**CA AAA

AUAUGAAU GCUGAGAAUAAAUAUGGGACUUC**UC** **AAA UGCGGA GAUAAU** GCAU \

UAUACUUA CGACUCUUAUUUAUACCCUGAAGAG UUU ACGCCU CUAUUG CGUA A

G C A U ---- AGU

PC-5p-67443-88:

UCAAAACUGCGGAAGAUAAUCC

Identified by sequence similarity

Stem-loop structure:

**A C** **CC**CA AAA

GAAUCGCUGAGAAUAAAUAUGGGACUUU**UC** **AAA UGCGGAAGAUAAU** GCAU \

CUUAGUGACUCUUAUUUAUACCCUGAAAAG UUU ACGCUUUCUAUUG CGUA A

C A ---- AAU

PC-5p-67443-89:

UCAAAACUGCGGAAGAUAAUCC

Identified by sequence similarity

Stem-loop structure:

C G U **A C** **CC**CA AAA

UUGUACAAUUGAAU GCU AGAAUAAAUA GGGACUUC**UC AAA UGCGGAAGAUAAU** GCAU \

AAUAUGUUAACUUA CGA UCUUAUUUAU CCCUGAAGAG UUU ACGCCUUCUAUUG CGUA A

A G U C A ---- AAU

PC-5p-67443-90:

UCAAAACUGCGGAAGAUAAUCC

Identified by sequence similarity

Stem-loop structure:

A U A **CAA C CC**CA AAA

AUAU AAUG CACAGAAUGAAA UGGUCCUUU**U** **AA UGCGGAAGAUAAU** GCAU \

UAUA UUAC GUGUCUUACUUU GCCAGGAAAA UU ACGCCUUCUAUUG CGUA A

C C C ACC A ---- AAU

PC-5p-67443-91:

UCAAAACUGCGGAAGAUAAUCC

Identified by sequence similarity

Stem-loop structure:

A **CAA C** **CC**CA AAA

GAAUGGCACAGAAUGAAA UGGUCCUUU**U** **AA UGCGGAAGAUAAU** GCAU \

CUUACCGUGUCUUACUUU GCCAGGAAAA UU ACGCCUUCUAUUG CGUA A

C ACC A ---- AAU

PC-3p-54311-1:

GAAAAUUAACAUAGUCGACUG

Identified by deep-sequencing

Stem-loop structure:

G C CCAAAUUC

CUUCAGAACAGUCGACUAUGUUGAU UUCGACUGUGUA CGGUCG \

GAAGUCUU**GUCAGCUGAUACAAUUA** **AAG**CUGGCAUGU GUCAGC A

**A** U UAUACUUU

PC-3p-54311-2:

GAAAAUUAACAUAGUCGACUG

Identified by sequence similarity

Stem-loop structure:

G C CCAAAUUC

GGACACGACUUCAGAACAGUCGACUAUGUUGAU UUCGACUGUGUA CGGUCG \

CCUGUGCUGAAGUCUU**GUCAGCUGAUACAAUUA** **AAG**CUGGCAUGU GUCAGC A

**A** U UAUACUUU

PC-3p-54311-3:

GAAAAUUAACAUAGUCGACUG

Identified by sequence similarity

Stem-loop structure:

UGAC A C CACAAAUU

GGACACGACUUCAGAACAGUCGACUGU GUUGAU UUCGACUGUGUA CGGUCG A

CCUGUGCUGAGGUCUU**GUCAGCUGAUA** **CAAUUA** **AAG**CUGGCAUGU GUCAGC A

---- **A** U UAUACUUU

PC-3p-54311-4:

GAAAAUUAACAUAGUCGACUG

Identified by sequence similarity

Stem-loop structure:

C G C CAAAAUUC

ACGACUUUAGAACAGUCGACUA GUUGAU UUCGACUGUGUA CGGUCG \

UGCUGAAGUCUU**GUCAGCUGAU** **CAAUUA** **AAG**CUGGCAUGU GUCAGC A

**A A** U UAUACUUU

PC-3p-54311-5:

GAAAAUUAACAUAGUCGACUG

Identified by sequence similarity

Stem-loop structure:

C G C UACC UCGC

AAUAUCGGACACGACUUCAGAACAGUCGACUA GUUGAU UUCGA UGUG GG \

UUAUAGCCUGUGCUGAAGUCUU**GUCAGCUGAU CAAUUA** **AAG**CU AUAC CU C

**A A** - CUUA UAAA

PC-3p-54311-6:

GAAAAUUAACAUAGUCGACUG

Identified by sequence similarity

Stem-loop structure:

----- GU- U - CAAAUUC GCAU CUCG

GUCAAAGUCGAAAUAUC CGACU GA CGGUCG C AUU ACCG \

CAGUUUCAGUUUUAUAG GCUGA CU **GUCAGC** **G UAA** UGGC U

CCUGU AGU U **U AUACAAU** **AAG**U AUGU

PC-3p-54311-7:

GAAAAUUAACAUAGUCGACUG

Identified by sequence similarity

Stem-loop structure:

C G G AC CCAAAUUC

UCAAAGUCGAAAUAUCGGACACGACUUCAGAACAGUCGACUA GUUAAU UUCGGCU UAU CGGUCG \

AGUUUCAGCUUUAUAGCCUGUGCUGAAGUCUU**GUCAGCUGAU** **CAAUUA** **AAG**CUGG AUG GCUAGC A

**A A** A CU CAUACUUU

PC-3p-54311-8:

GAACAUUAACGUAGUCGACUG

Identified by sequence similarity

Stem-loop structure:

U AC UACGACG AUGA UG

UCAAAGUCGAAAUAUCGGACACGACUUCAGAACAGUCGACUAUGUUAAU UUCG CU AUCGGU AA \

AGUUUCAGCUUUAUAGCCUGUGCUGAAGUCUU**GUCAGCUGAUGCAAUUA** **AAG**C GA UGGCCA UU A

**C** C- CAUA--- GCGG UA

PC-3p-54311-9:

GAAAAUUAACAUAGUCGACUG

Identified by sequence similarity

Stem-loop structure:

CA GUUA C CCAAAUUC

AGUCAAAGUUGAAAUAUCGGACACAACUUCAGAACAGUCGACUA UUGAU GACUGUGUA CGGUCG \

UCAGUUUCAACUUUAUAGCCUGUGUUGAAGUCUU**GUCAGCUGAU** **AAUUA** CUGGCAUGU GUCAGC A

**AC** **AAAG** U UAUACUUU

PC-3p-54311-10:

GAAAAUUAACAUAGUCGACUG

Identified by sequence similarity

Stem-loop structure:

- A A C G C CCAAAUUC

GUCAAAGUCGAAAUAUCGGACAC ACU CAGAACAGUC ACUA GUUGAU UUCGACUGUGUA CGGUCG \

CAGUUUCAGCUUUAUAGCCUGUG UGA GUCUU**GUCAG** **UGAU CAAUUA** **AAG**CUGGCAUGU GUCAGC A

C A **C A A** U UAUACUUU

PC-3p-54311-11:

GAAAAUUAACAUAGUCGACUG

Identified by sequence similarity

Stem-loop structure:

A G C A CCU C AAUUU

AAAGUCGAAAUAUUGGACA GACUUCA AACAGUCGACUA GUUAAU UUCGACUGUGUA GUCG UA \

UUUCAGCUUUAUAGCCUGU UUGAAGU UU**GUCAGCUGAU** **CAAUUA** **AAG**CUGGCAUGU CAGC AU A

G A **A A** UGC C ACUAU

PC-3p-54311-12:

GAAUAUUAACGUAGUCGACUG

Identified by sequence similarity

Stem-loop structure:

C CA U U C-- AACG G UGAUA

AAAGUCGAAAUAU GGACA ACUUCA AACAGUCGACUAUGUUAAU UUCGAC GUAC GUCG UA \

UUUCAGCUUUAUA CCUGU UGAAGU UU**GUCAGCUGAUGCAAUUA** **AAG**CUG CAUG CAGC AU U

A UC C **U** ACA GA-- G UUAAA

PC-3p-54311-13:

GAAAAUUAACAUAGUCGACUG

Identified by sequence similarity

Stem-loop structure:

GUGUUAAUGUAA C CCAAAUUC

GUCAAAGUCGUAAUAUCGGACACGACUUCAGAACAGUCGACUAUGUUAAU ACUGUGUA UGGUCG \

CAGUUUCAGCAUUAUAGCCUGUGCUGAAGUCUU**GUCAGCUGAUACAAUUA** UGGCAUGU GCCAGC A

**AAAG**C------- U CAUACUUU

PC-3p-54311-14:

GAAAAUUAACAUAGUCGACUG

Identified by sequence similarity

Stem-loop structure:

C A C CCAAAUUC

GUCAAAGUCGAAAUAUCGGACACGACUUCAGAACAGUCGAUUA GUUGAU UUCGACUGUGUA CGGUCG \

CAGUUUCAGCUUUAUAGCCUGUGCUGAAGUCUU**GUCAGCUGAU** **CAAUUA** **AAG**CUGGCAUGU GUCAGC A

**A A** U UAUACUUU

PC-3p-54311-15:

GAAAAUUAACAUAGUCGACUG

Identified by sequence similarity

Stem-loop structure:

C G UACA CCAAAUUC

AAAGUCGAAAUAUCGGACACGACUUCAGAACAGUCGACUA GUUGAU UUCGACUGUG GGUCG \

UUUCAGCUUUAUAGCCUGUGCUGAAGUCUU**GUCAGCUGAU** **CAAUUA** **AAG**CUGGCAU UCAGC A

**A A** GUUG UAUACUUU

PC-3p-54311-16:

GAAAAUUAACAUAGUCGACUG

Identified by sequence similarity

Stem-loop structure:

C G AGUACCGG CCCUA

GUCAAAGUUGAGAUAUCGGACACGACUUCAGAACAGUCGACUA GUUAAU UUCGACUG UCG A

CAGUUUCAGCUUUAUAGCCUGUGCUGAAGUCUU**GUCAGCUGAU** **CAAUUA** **AAG**CUGGC AGC A

**A A** AUGUUGUA UAUAC

PC-3p-54311-17:

GAACAUUAACGUAGUCGACUG

Identified by sequence similarity

Stem-loop structure:

U C-- AACAUUCGAUAUGAA

AUAUCGGACACGACUUCAGAACAGUCGACUAUGUUAAU UUCGAC GUAC A

UAUAGCCUGUGCUGAAGUCUU**GUCAGCUGAUGCAAUUA** **AAG**CUG CAUG U

**C** ACU GCCAGGCGGGUUUAG

PC-3p-54311-18:

GAAAAUUAACAUAGUCGACUG

Identified by sequence similarity

Stem-loop structure:

CA A C G A C CC-- UU

UCGAAAUAUCGGA CGACUUC GAACAGUCGACUA GUUGAU UUCG CUGUGUA CGGUCG GAA \

AGCUUUAUAGCCU GCUGAAG CUU**GUCAGCUGAU** **CAAUUA** **AAG**C GGCAUGU GUCAGC CUU C

AC C **A A**  C U UAUA UA

PC-3p-54311-19:

GAAAAUUAACAUAGUCGACUG

Identified by sequence similarity

Stem-loop structure:

C A C CCAAAUUC

AAAGUCGAAAUAUCGGACACGACUUCAGAACAGUCGACUA GUUGAU UUCGACUGUGUA CGGUCG \

UUUCAGCUUUAUAGCCUGUGUUGAAGUCUU**GUCAGCUGAU** **CAAUUA** **AAG**CUGGCAUGU GCCAGC A

**A A** U UAUACUUU

PC-3p-54311-20:

GAAAAUUAACAUAGUCGACUG

Identified by sequence similarity

Stem-loop structure:

C G C CCAAAUUC

GUCAAAGUCGAAAUAUCGGACACGACUUCAGAACAGUCGACUA GUUGAU UUUGAUUGUGUA CGGUCG \

CAGUUUCAGCUUUAUAGCCUGUGCUGAAGUCUU**GUCAGCUGAU** **CAAUUA** **AAG**CUGGCAUGU GUCAGC A

**A A** U UAUACUUU

PC-3p-54311-21:

GAAAAUUAACAUAGUCGACUG

Identified by sequence similarity

Stem-loop structure:

C G CU GCCAAAUUC

GUCAAAGUCGAAAUAUCGGACACGACUUCAGAACAGUCGACUA GUUGAU UUCGACUGUGUA GGUC \

CAGUUUCAGUUUUAUAGCCUGUGCUGAAGUCUU**GUCAGCUGAU** **CAAUUA** **AAG**CUGGCAUGU UCAG A

**A A** UG AUAUACUUU

PC-3p-54311-22:

GAAAAUUAACAUAGUCGACUG

Identified by sequence similarity

Stem-loop structure:

C G AG C CCAAAUUC

UCGAAAUAUUGGACACGACUUCAGAACAGUCGACUA GUUGAU UUCGACUG UA CGGUCG \

AGCUUUAUAGCCUGUGCUGAAGUCUU**GUCAGCUGAU** **CAAUUA** **AAG**CUGGC GU GCCAGC A

**A A** AU U UAUACUUU

PC-3p-54311-23:

GAAAAUUAACAUAGUCGACUG

Identified by sequence similarity

Stem-loop structure:

G C G C CCAAAUUC

GACUUCA AACAGUCGACUA GUUGAU UUUGACUGUGUA CGGUCG \

CUGAAGU UU**GUCAGCUGAU** **CAAUUA** **AAG**CUGGCAUGU GUCAGC A

A **A A** U UAUACUUU

PC-3p-54311-24:

GAAAAUUAACAUAGUCGACUG

Identified by sequence similarity

Stem-loop structure:

C A C CCAAAUUC

GUCGAAAUAUCGGACACGACUUCAGAACAGUCGACUA GUUGAU UUCGACUGUGUA CGGUCG \

CAGCUUUAUAGCCUGUGCUGAAGUCUU**GUCAGCUGAU** **CAAUUA** **AAG**CUGGCAUGU GUCAGC A

**A A** U UAUACUUU

PC-3p-54311-25:

GAAAAUUAACAUAGUCGACUG

Identified by sequence similarity

Stem-loop structure:

C G U CC-- UU

UAUCGGA ACGACUUCAGAACAGUCGACUAUGUUAAU UUCGACUGUGUA CGGUCG GAA \

AUAGCCU UGCUGAAGUCUU**GUCAGCUGAUACAAUUA** **AAG**CUGGCACGU GCCAGU CUU C

U **A** U CAUA UA

PC-3p-54311-26:

GAACATTAACATAGTCGACTG

Identified by sequence similarity

Stem-loop structure:

U C CAA A A AU

ACGACUUCAGAACAGUCGACUAUGUUAAU UUCGAC GUG CGGUC GU UGAA \

UGCUGAAGUCUU**GUCAGCUGAUACAAUUA** **AAG**CUG CAC GCCAG CG GCUU G

**C** A AUA - - AA

PC-3p-54311-27:

GAAAAUUAACAUAGUCGACUG

Identified by sequence similarity

Stem-loop structure:

C C G CC CCAAAUUC

AUCGGA ACGACUUCAGAACAGUCGACUA GUUGAU UUCGACUGUGUA GGUUG \

UAGCCU UGCUGAA**GUCUUGUCAGCUGAU** **CAAUUA** **AAG**CUGGCAUGU UCAGC A

A **A A** UA UAUACUUU

PC-3p-54311-28:

GAAAAUUAACAUAGUCGACUG

Identified by sequence similarity

Stem-loop structure:

C G C CCAAGUUC

GUUGAAAUAUCGGACACGACUUCAGAACAGUCGACUA GUUGAU UUCGACUGUGUA CGGUCG \

CAGCUUUAUAGCCUGUGCUGAAGUCUU**GUCAGCUGAU** **CAAUUA** **AAG**CUGGCAUGU GUCAGC A

**A A** U UAUACUUU

PC-3p-54311-29:

GAAAAUUAACAUAGUCGACUG

Identified by sequence similarity

Stem-loop structure:

C C G C CCAAAUUC

UAUCGGACA GACUUCAGAACAGUCGACUA GUUGAU UUCGACUGUGUA CGGUCG \

AUAGCCUGU CUGAAGUCUU**GUCAGCUGAU** **CAAUUA** **AAG**CUGGCAUGU GUCAGC A

A **A A** U UAUACUUU

PC-3p-54311-30:

GAAAAUUAACAUAGUCGACUG

Identified by sequence similarity

Stem-loop structure:

C G AGUAC CCAAAUUC AUA

GUCAAAGUCGAAAUAUCGGACACGACUUCAGAACAGUCGACUA GUUGAU UUCGACUG CGGUCG AUUUCAUA \

CAGUUUCAGCUUUAUAGCCUGUGCUGAAGUCUU**GUCAGCUGAU** **CAAUUA** **AAG**CUGGC GCCAGC UAAAGUAU U

**A A** AUUUU UAUACU-- AAG

PC-3p-54311-31:

GAAAAUUAACAUAGUCGACUG

Identified by sequence similarity

Stem-loop structure:

A C G AG C CCAAAUUU

CGACUUCG AACAGUCGACUA GUUGAU UUCGACUG UA CGGUCG \

GCUGAAGU UU**GUCAGCUGAU** **CAAUUA** **AAG**CUGGC GU GCCAGC A

C **A A** AU U UAUACUUU

PC-3p-54311-32:

GAACAUUAACGUAGUCGACUG

Identified by sequence similarity

Stem-loop structure:

A AG U A ACAA G UAAAA

AUUUCGACUAGUCAAAGUCGAAAUAUCG ACACGAC UUCAGAACAGUCGACUAUGUUAAU UUCGACUGU UAU CGGUCG UA \

UAAAGCUGAUCAGUUUCAGCUUUAUAGC UGUGCUG AAGUCUU**GUCAGCUGAUGCAAUUA** **AAG**CUGACA AUG GCCAGC GU U

C -- **C** C ---- G UUAAG

PC-3p-54311-33:

GAAAAUUAACAUAGUCGACUG

Identified by sequence similarity

Stem-loop structure:

-- C G C---- CC-- UU

UCGAAAUAUCGGACACGAC UUCAGAACAGUCGACUA GUUAAU UUCGACUGUGUA CGGUCG AAA \

AGCUUUAUAGCUUGUGCUG AA**GUCUUGUCAGCUGAU** **CAAUUA** **AAG**CUGACAUAU GCCAGC UUU C

UC **A A** AUGUU CAUA UA

PC-3p-54311-34:

GAACAUUAACGUAGUCGACUG

Identified by sequence similarity

Stem-loop structure:

A AG U A ACAA G UAAAA

AUUUCGACUAGUCAAAGUCGAAAUAUCG ACACGAC UUCAGAACAGUCGACUAUGUUAAU UUCGACUGU UAU CGGUCG UA \

UAAAGCUGAUCAGUUUCAGCUUUAUAGC UGUGCUG AAGUCUU**GUCAGCUGAUGCAAUUA** **AAG**CUGACA AUG GCCAGC GU U

C -- **C** C ---- G UUAAG

PC-3p-54311-35:

GAAAAUUAACAUAGUCGACUG

Identified by sequence similarity

Stem-loop structure:

C C CCAAAUUC

GAAAUAUCGGACACGACUUCAGAACAGUCGACUA GUUGAUUUUCGACUGUGUA CAGUCG \

CUUUAUAGCCUGUGCUGAAGUCUU**GUCAGCUGAU** **CAAUUAAAAG**CUGGCAUGU GUCAGC A

**A** U UAUACUUU

PC-3p-54311-36:

GAAAAUCAACGUAGUCGACUG

Identified by sequence similarity

Stem-loop structure:

A C-- AA AU-- GAA

GAAAUAUCGGACACGACUUCAGAACAGUCGACUAUGUU AUUUUCGAC GUAC CAGUCG AU \

CUUUAUAGCCUGUGCUGAAGUCUU**GUCAGCUGAUGCAA** **UAAAAG**CUG CAUG GUCAGC UA A

**C** ACA -- GGUU AGU

PC-3p-54311-37:

AAAAAUUAACAUAGUCGACUG

Identified by sequence similarity

Stem-loop structure:

CC C G C C CC-- UU

GACACGA UCAGAACAGUCGACUA GUUGAU UU GACUGUGCA CGGUCG AAA \

CUGUGCU AGUCUU**GUCAGCUGAU** **CAAUUA AA** CUGGCAUGU GUCAGC UUU C

AA **A A A** U UAUA UA

PC-3p-54311-38:

GAACAUUAACGUAGUCGACUG

Identified by sequence similarity

Stem-loop structure:

U A A A A U G .-AUA| AU

UCAUUUCGACU GUCAAAGUCGAAAUAUC GA ACG CUUCA AAUAGUCGACUAUGUUAAU UUCGA UG UGAA \

AGUAAAGCUGG CAGUUUCAGCUUUAUAG CU UGC GAAGU UU**GUCAGCUGAUGCAAUUA** AAG**UU** AC ACUU G

U C G A C **C** G \ ---^ AA

PC-3p-54311-39:

AAAAAUUAACAUAGUCGACUG

Identified by sequence similarity

Stem-loop structure:

G C G C GUGU GUCGCCAAAUU

GACUUCAGAACAGUCGA UA GUUAAU UU GACU GUACCG C

CUGAAGUCUU**GUCAGCU** **AU CAAUUA AA** CUGG CAUGGC A

**G A A** **A** ---- AACCAUACUUU

PC-3p-54311-40:

GGAAAUUAACAUAGUCGACUG

Identified by sequence similarity

Stem-loop structure:

C C G C C C CCAAAUUU

CGAAAUAUCGGACACGACUU AGAACGGUCGACUA GUUGAU UUCGA UGU UA CGGUCG \

GCUUUAUAGCCUGUGCUGAA UCUU**GUCAGCUGAU** **CAAUUA** **AGG**CU GCA GU GUCAGC A

A **A A** A U U UAUACUUU

PC-3p-54311-41:

UAAAAUUAACAUAGUCGACCG

Identified by sequence similarity

Stem-loop structure:

C A C GU--- C UACC UCGC

GUCAAAGUCGAAAUAU GGACACGACUUCAGAAC GUCGACUA GUUGAU UCGA UGUG GG \

CAGUUUCGGCUUUAUA CCUGUGCUGAAGUCUU**G** **CAGCUGAU** **CAAUUA** AGCU AUAC CU C

A **C** **A** **AAAU**C - UUUA UAAA

PC-3p-54311-42:

GAAAAUUAACAUAGUCGAGUG

Identified by sequence similarity

Stem-loop structure:

G C G C CCAAAUUU

CGAAAUAUCGGAUACGACUUCAGAACA UCGACUA GUUGAU UUCGACUGUGUA CGGUCG \

GCUUUAUAGCCUGUGCUGAAGUCUU**GU** **AGCUGAU CAAUUA** **AAG**CUGGCAUGU GUCAGC A

**G A A**  U UAUACUUU

PC-3p-54311-43:

GAAAAUAAACAUAGUCGACUG

Identified by sequence similarity

Stem-loop structure:

C A G C C CCAAAUUC

AGUCGAAAUAUCGGACACGACUUCAGAACAGUCGACUA GUU AU UUCGA UGUGUA CGGUUG \

UUAGCUUUAUAGCCUGUGCUGAAGUCUU**GUCAGCUGAU** **CAA UA** **AAG**CU GCACGU GCCAGC A

**A A A** A U CAUACUUU

PC-3p-54311-44:

GAACAUUAACGUAGUCGACUG

Identified by sequence similarity

Stem-loop structure:

U U C CAA CG AUGAAA

UCGAAAUAUCGGACACGACUUCAGAACAGUCGACUAUGUU AU UUCGAU GUG CGGU GU U

AGCUUUAUAGCCUGUGCUGAAGUCUU**GUCAGCUGAUGCAA** **UA** **AAG**CUG CAC GCCA CG G

**U C** A AUG A- GUUUAA

PC-3p-54311-45:

GAAAAUUAACAUAGUCCACUG

Identified by sequence similarity

Stem-loop structure:

C C C G C CCAAAUUC

UCAUUUCGACUUGUCAAAGUCGAAAUAUCGGACA GACUUCAGAACAGU GACUA GUUGAU UUCGACUGUGUA CGGUCG \

AGUAAAGCUGAACAGUUUCAGCUUUAUAGCCUGU CUGAAGUCUU**GUCA** **CUGAU CAAUUA** **AAG**CUGGCAUGU GUCAGC A

A **C A A** U UAUACUUU

PC-3p-54311-46:

GAAAAUUAACAUAGUUGACUG

Identified by sequence similarity

Stem-loop structure:

C G AC UACCAAUCGC

AAAUAUCGGACACGGCUUCAGAACAGUCGACUA GUUAAU UUCG UGUG \

UUUAUAGCCUGUGCUGAAGUCUU**GUCAGUUGAU** **CAAUUA** **AAG**C AUAC C

**A A** C- UUUACUUAAA

PC-3p-54311-47:

GAACAUUAACGUAGUCGACUG

Identified by sequence similarity

Stem-loop structure:

G A A U ----- G A AAU

GUCAAAGUC AAAUAUCGGACACG CUUCAGAACAGUC ACUAUGUUAAU UUCG GUAU AA UG \

CAGUUUCAG UUUAUAGCCUGUGC GAAGUCUU**GUCAG** **UGAUGCAAUUA AAG**C CAUG UU GC U

G C **C C** UGACA G A GGU

PC-3p-54311-48:

GAAAAUAAACAUAGUCGACUG

Identified by sequence similarity

Stem-loop structure:

C A GUC C CCAAAUUC

GACUUCAGAACAGUCGACUA GUU AU CGACUGUGUA CGGUUG \

CUGAAGUCUU**GUCAGCUGAU** **CAA UA** **G**UUGGCAUGU GCCAGC A

**A A AAA** U CAUACUUU

PC-3p-54311-49:

GAAAAUUAACAUAAUCGACUG

Identified by sequence similarity

Stem-loop structure:

C C G UACA CCAAACUC

GUCGAAAUAUCGGACACGACUUCAGAACAGUUGA UA GUUGAU UUCGACUGUG GGUCG \

CAGCUUUAUAGCCUGUGCUGAAGUCUU**GUCAGCU** **AU CAAUUA** **AAG**CUGGCAU UCAGC A

**A A A** GUUG UAUACUUU

PC-3p-54311-50:

GAAAAUUAACAUAGUUGACUG

Identified by sequence similarity

Stem-loop structure:

A C G C GCCAAAUUC

GUCAAAGUCGAAAUAUCGGACACGACUUCA AACAGUCGACUA GUUGAU UUCGACUGUGUA CGGUC \

CAGUUUCAGCUUUAUAGCCUGUGCUGAAGU UU**GUCAGUUGAU** **CAAUUA** **AAG**CUGGCAUGU GUCAG A

C **A A** U AUAUAUUUU

PC-3p-54311-51:

GAAAAUUAACAUAGUCGCUUU

Identified by sequence similarity

Stem-loop structure:

C U C A C CC-- UU

GAA AG CGACUG GUUGAU UUCGACUGUGUA CGGUCG AAA \

CUU UC **GCUGAU** **CAAUUA** **AAG**CUGGCAUGU GUCAGC UUU C

U - **A A** U UAUA UA

PC-3p-54311-52:

GAAAAUUGACAUAGUCGACUG

Identified by sequence similarity

Stem-loop structure:

G C U GUUA CA C CCAAAUUC

GUCAAAGUCGAAAUAUCGGA ACGACUUCAGAACAGUCGACUA GUU AU GA GUGUA CGGUCG \

CAGUUUCAGCUUUAUAGCCU UGCUGAAGUCUU**GUCAGCUGAU** **CAG UA** CU CAUGU GCCAGC A

G **A U** **AAAG** AG U UAUACUUU

PC-3p-54311-53:

GAAAAUUAAUAUAGUCGACUG

Identified by sequence similarity

Stem-loop structure:

CG G CUU C-- CCAAAUUC

GUCAAAGUUGAAAUAUCGGACACGACUUCAGAACAGUCGACUA UUAAU UUCGACC GUAC GGUCG \

CAGUUUCAGCUUUAUAGCCUGUGCUGAAGUCUU**GUCAGCUGAU** **AAUUA AAG**CUGG CAUG CCAGC A

**AU A** --- UUA CAUACUUU

PC-3p-54311-54:

GAACAUUAACGUAGUCGACUG

Identified by sequence similarity

Stem-loop structure:

UA U --- AA G UGAAA

GAAAUAUCGGACACGACUUCAGAACAGUCGACUA UUAAU UUCGACC GUAC UGGUCG UA \

CUUUAUAGCCUGUGCUGAAGUCUU**GUCAGCUGAU** **AAUUA** **AAG**CUGG CAUG GCCAGC GU U

**GC C** GAA -- G UUAAG

PC-3p-54311-55:

GAAAAUUAACUUAGUCGACUG

Identified by sequence similarity

Stem-loop structure:

A U C G C CU CCAAAUUC

GUCAAAGUCGAAAUAUCGGACACGACU CAGAACA UCGACUA GUUGAU UUCGA UGUGUA GGUCG \

CAGUUUCAGCUUUAUAGCCUGUGCUGA GUCUU**GU AGCUGAU CAAUUA** **AAG**CU GCAUGU UCAGC A

A **C U A**  A UG UAUACCUU

PC-3p-54311-56:

GAAAAUUAACUUAGUCGACUG

Identified by sequence similarity

Stem-loop structure:

A U C G C CU CCAAAUUC

GUCAAAGUCGAAAUAUCGGACACGACU CAGAACA UCGACUA GUUAAU UUCGA UGUGUA GGUCG \

CAGUUUCAGUUUUAUAGCCUGUGCUGA GUCUU**GU** **AGCUGAU CAAUUA** **AAG**CU GCAUGU UCAGC A

A **C U A** A UG UAUACUUU

PC-3p-54311-57:

GAACAUUAACGUAGUCGAAUG

Identified by sequence similarity

Stem-loop structure:

U G A U C-- AACA-| UAUGA

AAAUAUCGGACACGACU CAGAACA UCGACUA GUUAAU UUCGAU GUAC GUCGA A

UUUAUAGCCUGUGCUGA GUCUU**GU** **AGCUGAU CAAUUA** **AAG**CUG CAUG CGGUU A

U **A G C** ACA ACCAG^ UAAGU

PC-3p-54311-58:

GAAAAUUGACAUAGUCGACUG

Identified by sequence similarity

Stem-loop structure:

A C GU C GUUA C CC-- UU

AAAUAUCGG CA GAUUUCAGAACAGUCG UA GUUAAU GACUGUGUA CGGUCG AAA \

UUUAUAGCC GU CUGAAGUCUU**GUCAGC AU CAGUUA** CUGGCAUGU GCCAGC UUU C

C A **UG A** **AAAG** U CAUA UA

PC-3p-54311-59:

GAAAAUUAAUAUAGUCGACUG

Identified by sequence similarity

Stem-loop structure:

C A CG G C C CGCUAAAUUC

GAAAUAU GGACACGA UUCAGGACAGUCGACUA UUAAU UUUGA UGUGUA CGGU \

CUUUGUA CCUGUGCU AAGUUUU**GUCAGCUGAU** **AAUUA** **AAG**CU GCAUGU GCCA A

A G **AU A** A U ACCAUACUUU

PC-3p-54311-60:

AAACAUUAACGUAGUCGACUG

Identified by sequence similarity

Stem-loop structure:

C AA UA U C C-- AA G UGAAA

AUUGGACACGA UUCA ACAGUCGACUA UUAAU UU GAU GUAC CGGUUG UA \

UAGCCUGUGCU AAGU U**GUCAGCUGAU** **AAUUA AA** CUG CAUG GCCAGC AU U

U CC **GC C** **A** ACA -- G UUAAG

PC-3p-54311-61:

GAAAAUUAACGUAGUCGACUG

Identified by sequence similarity

Stem-loop structure:

A A UAA CUA C A C ACAA UU ----- ACU

AUUUCGACUAGUCAAAGUC AAA AUCG ACGA CAGAAUAGUCGACUAUGUU AUUUUC AC GU CG UCGCC CAU A

UAAAGCUGAUCAGUUUCAG UUU UAGC UGCU GUCUU**GUCAGCUGAUGCAA** **UAAAAG** UG CA GC AGCGG GUA U

C A CUG AAA **U** C A CAUA C- UUUAA AAG

PC-3p-54311-62:

GAAAAUGAACAUAGUCGACUA

Identified by sequence similarity

Stem-loop structure:

U GAC U C C A U G- AA- CAU

UUUCGACUAGUCAAAGUCGAAA AUCG ACGAUU CAGAA AGUCGACUA GUU AUUUUCGACUGUGUA CG UCGCC AUU U

AAAGCUGAUCAGUUUCAGUUUU UAGC UGCUGA **GUCUU UCAGCUGAU CAA** **UAAAAG**UUGGCAUGU GC AGCGG UGA U

U AUU U **A A G** U AA GUA UAC

PC-3p-54311-63:

GAACAUUAACGUAGUCGACUG

Identified by sequence similarity

Stem-loop structure:

C U A AAAUGAAU C

GGACACGACUUCAGAACAGUCGACUAUG UAAU UUCGAU UG UUGG \

CCUGUGUUGAAGUCUU**GUCAGCUGAUGC** **AUUA** **AAG**CUG AC GGCC G

**A C** - AAAU---- A

PC-3p-54311-64:

GAAAAUUAACGUAGUCGACUG

Identified by sequence similarity

Stem-loop structure:

UA A G CCAAAUU- UUUCA

AAACGGUUGACU GU AAU UUCGA CA U

UUU**GUCAGCUGA** **CA UUA** **AAG**CU GU A

**UG A A** AGCACUUU UAACA

PC-3p-54311-65:

AAAAAUUAACAUAUUCGACUG

Identified by sequence similarity

Stem-loop structure:

C C G C C ACCAAAUUC

AAAGUCGAAAUAUCGGACACGACUUCAGAACAGUCGA UA GUUGAU UU GACUGUGUA CGGUC \

UUUCAGCUUUAUAGCCUGUGCUGAAGUCUU**GUCAGCU** **AU CAAUUA AA** CUGGCAUGU GUCAG A

**U A A A** U CUAUACUUU

PC-3p-54311-66:

GAAAAUUGACAUAGUCGACUC

Identified by sequence similarity

Stem-loop structure:

GAAC C AG C CCAAAUUC

CUUCA AGUCGACUA GUUAA UUCGACUGUGUA CGGUCG \

GGAGU **UCAGCUGAU** **CAGUU** **AAG**CUGGCAUGU GCCAGC A

GA**C**- **A AA** U CAUACUUU

PC-5p-50354:

UGUUUGUAGAAAUUUCCAGUCGAUGU

Identified by deep-sequencing

Stem-loop structure:

**U** **AAUU** - G - UA

AA **GUUUGUAGA** **UCCAGU** **CGAUGU**UU UAAAC C \

UU CAAAUAUUU AGGUUA GCUGUAAA AUUUG G U

C AUC- A A A AG

PC-5p-45621

UGGGAAUAUGGACCAUGACAGGUGAA

Identified by deep-sequencing

Stem-loop structure:

-- UU**U** **U A**-- **C** CCGA---- AU

GC UUCUC **GGGAA** **AUGGACC** **UGA AGGUGAA** ACA U

CG AAGGG UCUUU UACCUGG GCU UCCAUUU UGU U

CU CU- U CCA U AAACAGAG AU

PC-5p-31567:

AAACACCAUUGUAUUCGUUUCAU

Identified by deep-sequencing

Stem-loop structure:

**C AU** **U**UUGUGC

GU**AAACAC AUUGU UCGUUUCA** U

CAUUUGUG UGACA AGCGAAGU A

U -- UUAAAAA

PC-3p-5216:

AUCACAAGAAUAAUCGUUUGG

Identified by deep-sequencing

Stem-loop structure:

C A U CU GC UU AAA

UG A UAAA CGAU UUUUUG GUU CAUA \

AC U **GUUU** GCUA AAGAAC **UA**A GUGU U

C G - **AU AC** C- CGU

PC-3p-45698:

UCAGUCGCCGAUCCGUUUGACA

Identified by deep-sequencing

Stem-loop structure:

-- C A UU

UUUGUCGGACGG CGGCGAC UGAGU UAG G

AG**ACAGUUUGCC** **GCCGCUG** **ACU**UA AUU A

**UA** - - UU

PC-3p-16936-1:

UAUUGAUUGCAUGUCCCAUAUG

Identified by deep-sequencing

Stem-loop structure:

C G U UAU

AUGGGGAACUUCAUAUGGGACA GCAAUUAAUGAA UA ACUU U

UACCCCUUGAA**GUAUACCCUGU** **CGUUAGUUAU**UU AU UGAA A

**A** A U UUA

PC-3p-16936-2:

CAUUAAUUGCGUGUCCCAUAUG

Identified by sequence similarity

Stem-loop structure:

C A U A AAU

AUGGGGAACUUCAUAUGGGACAUGCAAU AAU AA UA ACUU U

UACCCCUUGAA**GUAUACCCUGUGCGUUA** **UUA** UU AU UGAA A

**A** **C** C A AUA

PC-3p-15390-1

UAGAAUCUCUCCAUCGGAUUAA

Identified by deep-sequencing

Gene not in the draft genome sequence

PC-3p-15390-2:

UAGAAUCUCUCCAUCGGAUCAA

Identified by sequence similarity

Stem-loop structure:

U GUA G AA UU AA

AAAUUAG AAGGU GUC GAUGGAGAGA CU UUUGU A

UUUAAUC UUUUA **UAG CUACCUCUCU GA** AAACA G

U **AAC** **G AA** **U**- AA

PC-3p-15390-3:

UAGAAUCUCUCCAUCAGAUUAA

Identified by sequence similarity

Stem-loop structure:

- U C C A G AA

AUGGAUU GG AAGGU UAGUC GAUGGAGAGA UCUAUU UGU A

UACUUGA UC UUUCA **AUUAG CUACCUCUCU** **AGAU**AA ACA C

G U **A** **A A** - AA

PC-3p-15390-4:

UAGAUUCUCUCCAUCGGACUAG

Identified by sequence similarity

Stem-loop structure:

A U - UU

GUUUA UCUGAUGGAGAGA UCUAUU UGU G

CA**GAU** **AGGCUACCUCUCU** **AGAU**AA ACA U

**C U** C UU

PC-3p-15390-5:

UAGAAUUUCUCCAUCGGAUUGA

Identified by sequence similarity

Stem-loop structure:

GA U C U A - GCA

GACC UUAG GAGGU UAGUCCG UGGAGAGA UCUAUU GU A

CUGG AAUC UUUCA **GUUAGGC ACCUCUUU** **AGAU**AA CA A

-- U **A** **U A** A AAC

PC-3p-55176-1:

UAUUUUCUUAGACAAAUCGGCGGA

Identified by deep-sequencing

Stem-loop structure:

AUGAAAU G AU- CA A

UAUUUAGUCUA UUCCG C UUUGUCUAAGA AAAUG C

GUAAAUCAGAU A**AGGC** **G** **AAACAGAUUCU** **UUUAU** G

AUAAAU- - **GCU** -- A

PC-3p-55176-2:

UAUUUUCUUAGACAAAACGGCGGA

Identified by sequence similarity

Stem-loop structure:

AUGAAAU GCA CA A

UAUUUAGUCUA UUCCG UUUUGUCUAAGA AAAUG C

GUAAGUCAGAU A**AGGC** **AAAACAGAUUCU** **UUUAU** G

AUAAAU- **GGC**  -- A

PC-3p-55176-3:

UAUUUUCUUAGACAAAACGGCGGA

Identified by sequence similarity

Stem-loop structure:

C UA AG CA A

UGU CUGU UU GUCUAAGA AAAUG C

AUA **GGCG** **AA CAGAUUCU** **UUUAU** G

**A** **GC AA** -- A

PC-3p-29582-1:

UACCAGGAUAACGCGAUCACCAAACA

Identified by deep-sequencing

Gene not in the draft genome sequence

PC-3p-29582-2:

UACUAGGAUAACGCGAUCACCAAACA

Identified by sequence similarity

Stem-loop structure:

UG - ------- C C G AA AAUGGUGUAUCCUC

UCAAG GCG UUUCG U UGGUGAUCGCG UG CC AGUGGA G

AGUUC UGC GAAGC **A** **ACCACUAGCGC AU GG** **UCAU**CU G

GU C CCCCU**AC A** **A A A**- AGCUGUACGGCUAC

PC-3p-29582-3:

UACUAGGAUAACGCGAUCACCAAACA

Identified by sequence similarity

Stem-loop structure:

UG - ------- C C G AA AAUGAUGUAUCC C

UCAAG GCG UUU UGU UGGUGAUCGCG UG CC AGUGGA UCGGCAU G

AGUUC UGC GAA **ACA** **ACCACUAGCGC AU GG** **UCAU**CU AGCCGUA G

GU C GCCCCCU **A A A** **A**- ------------ C

PC-3p-66779:

UAACUUCAAUUUUGAUUAUCGGUAA

Identified by deep-sequencing

Stem-loop structure:

UUUUUUUUUUA CU UU -- CU AG

AUCGAUAA UCAG UUGA UUAUCGAUAA UC U

**UGGCUAUU** **AGUU AACU** **AAU**AGCUAUU AG U

UUUGACUUC**AA** -- **UU UC** -- UU

PC-5p-2948:

UGAAGAUACAAGAUAAGAUGAAGAUA

Identified by deep-sequencing

Stem-loop structure:

U A **GAUA**-- - **A** UGA

GAU AAGAU AGA**UGAA** **CAAGAU AAGAUGA** **GAUA**AGA \

CUA UUCUA UCUACUU GUUCUA UUCUACU CUAUUUU U

- C AGCGAA A - CUA

PC-3p-44258:

UCAGUGUUCUGUAAAGAUUAAGAUAA

Identified by deep-sequencing

Stem-loop structure:

GUCC A AUA CU A AC

CAUU AUUU UAGUU ACAG UA UG \

GUAG UAG**A** **AUUAG UGUC GU AC** A

AAA- - **AAA UU G** **U**A

PC-5p-5905:

GCUGAAAUCUCGUGGAUCUGCA

Identified by deep-sequencing

Stem-loop structure:

CUA **C A AU** UUGAUAA

GAAUUC UCA**G** **GCUGAA UCUCGUGG CUGCA** \

CUUGAG AGUC CGACUU AGAGUACC GAUGU U

UAC U - AC CAAAAAG

PC-3p-39629-1

UAAAGUACUUUGAAUUAGAUGAAUGA

Identified by deep-sequencing

Gene not in the draft genome sequence

PC-3p-39629-2:

UAAAGUAUUUUGAAUUAGAUGAAUGA

Identified by sequence similarity

Stem-loop structure:

G AAAC GU AU

AA CAUUCG AUUCAA GAUAUUUUAGA \

UU **GUAAGU** **UAAGUU UUAUGAAAU**UU C

**A** **AGAU** -- GC

PC-5p-12050-1:

UCAUCCUCGGCGUCAUAUAUAGGGAA

Identified by deep-sequencing

Stem-loop structure:

CCAUU**UCA C U**  AA

**UCCU GGCG CAUAUAUAGGGA** \

GGGA UCGU GUGUAUAUUCCU A

AAUUGCAA U U AU

PC-5p-12050-2:

UCAUCCUCGGCGUCAUAUAUAGGGAA

Identified by sequence similarity

Stem-loop structure:

- U**UCA** **C- U** AA

GGACAA CCAU **UCCU GGCG CAUAUAUAGGGA** \

UCUGUU GGUG AGGG UCGU GUGUAUAUUCCU A

U CA-- CU U AU

PC-3p-22425:

UAAGAGAAAGACAUGCAGACUUUUCU

Identified by deep-sequencing

Stem-loop structure:

AAAAAC C A C- AUA GAAU

AGA AA UUUGC GUUU UUUUC \

**UCU UU** **AGACG CAGA AAGAG** A

CC---- **U C UA** --- **AAU**A

PC-5p-36494:

UGAGAAUGAUAUUGUGAU

Identified by deep-sequencing

Stem-loop structure:

UG**UGA** **A UG** **U**U - U UAAUAA

CCG **GAAUGAU UUG A** CGU AUG CUGGAAUUGUU \

GGU CUUACUA AGC U GCA UAU GACUUUAACAA A

UUG-- - GU U- C - UAUCUU

PC-5p-36861:

AGCGAUUGUGAAAAGACG

Identified by deep-sequencing

Stem-loop structure:

UAUUUG **A** **A**- **U**- **CG** U AA A

GAU **GCG UUG GAAAAGA** UA AUUUG UG A

CUA CGC AGU UUUUUUU GU UAAAC AC U

AAA--- - CG UU UU U -- C

PC-5p-29265:

UGGGAAUGUCAAUGGCGAGA

Identified by deep-sequencing

Stem-loop structure:

**GG** **CA**  AU U C- UUU

GA**UG** **AAUGU AUGGCGAG** CG AGU UGGGU \

CUAC UUAUA UGCUGCUC GU UUA AUCCA G

UU UC AU U AA UAA

PC-3p-27532:

UUGGACUGAGUGAUGUGGUAUAUAAU

Identified by deep-sequencing

Stem-loop structure:

GC U AAAAC C CC

GGUUGUUGUA GCU GCAUCG CA UCUAAA \

CCAA**UAAUAU** **UGG UGUAGU** **GU** **AGGUU**U G

**A**- - **GA**--- **C** UC

PC-5p-57969-1:

UGUUUGCCUUCAACAAUCGUUGUGAC

Identified by deep-sequencing

Stem-loop structure:

U**GU** - **UU A G C** UU

**GUUU GCC CAAC AUCGUU UGA** GA U

UAGA CGG GUUG UGGUAA ACU UU U

C-- U CU G A U UU

PC-5p-57969-2:

UGUUUGCCUUCAACAAUCGUUGUGGC

Identified by sequence similarity

Stem-loop structure:

U**AU** - **UU A G** **C**G UU

**GUUU GCC CAAC AUCGUU** **UGG** GA U

UAGA CGG GUUG UGGUAA ACU UU U

C-- U CU G A UU UU

PC-5p-57969-3:

UGUUUGCCGUCAACAAUCGUUGUGAC

Identified by sequence similarity

Stem-loop structure:

U**GU** - **U A G C** UU

**GUUU GCCG CAAC AUCGUU UGA** GA U

UAGA CGGC GUUG UGGUAA ACU UU U

C-- U U G A U UU

PC-3p-31500:

UCGGUAUUGCUGCAUGAAGGGUUUUC

Identified by deep-sequencing

Stem-loop structure:

**UA**-- **CA**-- **G U** UC

UUU**UCGG** **UUGCUG** **UGAAG GU UUC** \

AAGAGUU AAUGAU GUUUU CG AAG A

UACG UUAG G U UU

PC-3p-58746-1:

AUUGGCAUCAUGAGAUUUGAC

Identified by deep-sequencing

Stem-loop structure:

UAAC-- A- UU C - UC

UGGGGGGA UCG GUUA UUU GAUGGU CUA \

AUCCCCCU **AGC CAGU** **AGA CUACUA GGU** G

CACCC**C** **CC UU** - **C** **UA**

PC-3p-58746-2:

AUUGGCAUCAUCAGAUUUGAC

Identified by sequence similarity

Stem-loop structure:

UCA CU C A - UC

GGUUA UUU GA GAU CUA \

C**CAGU AGA CU CUA GGU** A

CC **UU**  - **A** **C** **UA**

PC-3p-58746-3:

AUUGGCAUCAUGGGAUUUGAC

Identified by sequence similarity

Stem-loop structure:

**U C** **UG**- **UU A** UU

AUCG**A** **UGG AUCA GGAU G C**ACG \

UGGCU ACC UAGU UCUG C GUGU G

U U UUA -- - UU

PC-3p-58746-4:

AUUGGCAUCAUCAGAUUUGAC

Identified by sequence similarity

Stem-loop structure:

A UA **A G CA** - **U**- **A**

UUUUUCG UGCUC UCG **UU GCAU UC AGA UUG C**

AAAGAGU ACGAG AGC AA CGUA AG UCU AGC C

A -- - G -- C CC C

PC-3p-58746-5:

AUUGGCAUCAUCAGAUUUGAC

Identified by sequence similarity

Stem-loop structure:

AAUAA CA UU C A AUC

GGGGG CU GGUUA UUU GA GAUGU G

CCCCC GA C**CAGU** **AGA CU CUACG** **A**

A---- CC **UU - A GUU**

PC-3p-58746-6:

AAUGGCAUCAUCAGAUUUGAC

Identified by sequence similarity

Stem-loop structure:

AUAACUCA UU C A - C

GGUUA UUU GA GAU CUAU \

C**CAGU AGA CU CUA GGUA** A

AACC---- **UU** - **A C** **A**

PC-3p-58746-7:

AAUGGCAUCAUCAGAUUUGAC

Identified by sequence similarity

Stem-loop structure:

UU C A - C

GGUUA UUU GA GAU CUAU \

C**CAGU** **AGA CU CUA GGUA** A

**UU** - **A C** **A**

PC-3p-58746-8:

AUUGGCAUCAUCAGAUUUUUC

Identified by sequence similarity

Stem-loop structure:

A A U A C C UC--- AC

AUCUGUCGAAUUUGACAUUUGAC AAAGG CUGAUGAUGCCAAUCGUA UUUUUUCAAUUUUAC UCGAUUGC CCU AUUUGACAAUCAAUAUUUGACAUUUGACGG GAU \

UAGACAGUUUAAACUGUAAACUG **UUUUU** **GACUACUACGGUUA**GCAU AAAAAAGUUAAAAUG AGCUAACG GGA UAAACUGUUAGUUAUAAACUGUAAACUGUC CUG U

**C** **A** - C U C UUAAA UU

PC-3p-58746-9:

AUUGGCAUCAUCAGAUUUAAC

Identified by sequence similarity

Stem-loop structure:

C U A A **C** **CAG**- ------ - --- UCU CUCCAAAAU

GGGCUAGCCCCCUUGA CCC UCCUAG CUAAC UUACUUUUCGAAGUUUUAUCG**AUUGG** **AUCAU** **AUUUA** **AC**CCC CGAC CCCCU CC \

CCCGAUCGGGGGAACU GGG AGGAUC GAUUG AAUGAGAAGCUUCAAAAUAGCUAACC UGGUA UGAGU UGGGG GUUG GGGGA GG A

A U C A A ACCA AGAUCC A UGU UUU UGUAAAAAA

PC-3p-58746-10:

AUUGGCAUCAUCAGAUUUUUC

Identified by sequence similarity

Stem-loop structure:

A A A C UC--- AC

UUUGACAUUUGAC AAAGA CUGAUGAUGCCAAUCGUAUUUUUUUUUUUCAAUUUUACAUCGAUUGCACCUCAUUU ACAAUCAAUAUUUGACAUUUGA GG GAU \

AGACUGUAAACUG **UUUUU** **GACUACUACGGUUA**GCAUAAAAAAAAAAAGUUAAAAUGUAGCUAACGUGGAGUAAA UGUUAGUUAUAAACUGUAAACU UC CUG U

**C** **A** C U UUAAA UU

PC-3p-58746-11:

AUUGGCAUCAUCAGAUUUUUC

Identified by sequence similarity

Stem-loop structure:

C A C- CG UC--- AC

GACAUUUGAC AAAGA CUGAUGAUGCCAAUCGUAUUUUUU UCAAUUUUACAUCGAUUGCACCUCAUUUGACAAUCAAUAUUUGACAUUUGA G GAU \

CUGUAAACU**G** **UUUUU** **GACUACUACGGUUA**GCAUAAAAAA AGUUAAAAUGUAGCUAACGUGGAGUAAACUGUUAGUUAUAAACUGUAAACU C CUG U

**C A** AA AU UUAAA UU

PC-3p-58746-12:

AUUGGCAUCAUUAGAUUUAUU

Identified by sequence similarity

Stem-loop structure:

GACAAA AGAG C- AAU U U U

AAUAAAUCUAAUGAUGCCAAUCGAU GAGGUA GAU GGGGGG AA GUU C

**UUAUUUAGAUUACUACGGUUA**GCUA CUUCAU CUA CUCCCC UU CAG A

------ CCCA CC GC- - C G

PC-3p-58746-13:

AUUGGCGUCAUCAGAUUUGAC

Identified by sequence similarity

Stem-loop structure:

**G** **A**- **AC**CCCCGAUCCCCUCUUCCCCCCAAAA AU

UCG**AUU** **GCGUCAUCAG** **UUUG** AUGCG \

AGCUAG CGUAGUAGUC AAAC UAUGC U

A AA CUUUAAGGAAGUGCAAAGACC------

PC-3p-59454-1:

UCUGAAAUGCGUAGAAAAACA

Identified by deep-sequencing

Stem-loop structure:

UC **C** - **U**  **A**

GGAAA G**U** **UGAAAUG** **CG** **AGAAAA** **C**

CUUUU UA AUUUUAC GU UCUUUU **A**

UA C A U A

PC-3p-59454-2:

UCUGAAACGCGUAGAAAAACA

Identified by sequence similarity

Stem-loop structure:

C U - UAA AU GA

GAAA UU UUUUUCU CGC UCG CG \

CUUU A**A** **AAAAAGA GCG AGU** GC A

U **C U CAA** **CU** UA

PC-3p-59454-3:

UCUGAAACGCGUAGAAAAACA

Identified by sequence similarity

Stem-loop structure:

U - CAA - G

AAUU UUUUUUU CGC UC GAUUG A

UUA**A** **AAAAAGA GCG AG** **CU**GAU A

**C** **U CAA U** A

PC-3p-59454-4:

UCUAAAAUGCGUAGAAAAACA

Identified by sequence similarity

Stem-loop structure:

AAAUC **CU** **UA**- **C**- UU

G**U** **AAAAUGCG GAAAAA A**GUUU C

CG UUUUACGU CUUUUU UUAAG U

AA--- U- UCC UU CU

PC-3p-59454-5:

UCUGAAACGCGUAGAAAAACA

Identified by sequence similarity

Stem-loop structure:

U U AA AU AA

GAAAUU UUUUUUU CGCG UCG UG \

UUUUG**A** **AAAAAGA GCGC AGU** GC A

**C U AA** **CU** UA

PC-3p-59454-6:

UCUAAAAUGCGUAGAAAAACA

Identified by sequence similarity

Stem-loop structure:

U - U CAA AA

AAU UUUUUUU CG CAUU UUGGA \

UU**A** **AAAAAGA GC GUAA AAUCU** U

**C** **U** - --- GC

PC-3p-59454-7:

UCUGAAACGCGUAGAAAAACA

Identified by sequence similarity

Stem-loop structure:

UU UU - CAA AU A

UUUUUUU GAAAUU UUUUUCU CGU UCG UGG \

AAAAAAG CUUUAA **AAAAAGA GCG AGU** GCC A

GU **C**- **U CAA** **CU** A

PC-3p-59454-8:

UCUGAAACGCGUAGAAAAACA

Identified by sequence similarity

Stem-loop structure:

U - UAA AU GA

CGAAAUU UUUUUCU CGC UCG CG \

GCUUUAA **AAAAAGA GCG AGU** GC A

**C** **U CAA** **CU** UA

PC-3p-59454-9:

UCUGAAACGCGUAGAAAAACA

Identified by sequence similarity

Stem-loop structure:

U - --- UGUAAUU

AUU UUUUUUU CGU UUAG A

UA**A** **AAAAAGA GCG AGUC** U

**C** **U CAA** **U**GCCCUU

PC-3p-36826:

UAUGGCUUGAUGUAGACGAUAGGUUG

Identified by deep-sequencing

Stem-loop structure:

AU UAA AA- C AUA AACCCCCGU

GACU UAUCG AUAUC AGC AUC \

**UUGG AUAGC UGUAG UCG** **U**GG A

U**G** --- **AGA U GUA** AAAGUGAAA

PC-3p-66266-1:

UUUGGACCAGUUCGGACCAGUU

Identified by deep-sequencing

Stem-loop structure:

UCGUU

AAACUGGUCCGGGCUGGUCCAAAA A

U**UUGACCAGGCUUGACCAGGUUU**U U

CACCU

PC-3p-66266-2:

UUUGGACCAGUUCGGACCAGUU

Identified by sequence similarity

Stem-loop structure:

A A A U UC UG

GAAUACAAGA UUUUGGCGUUAA ACUG UCCGAAAAACUGGUCU AACUGGUCCAAAA GU U

CUUAUGUUCU AAAACCGCAAUU UGAC AGGCUUU**UUGACCAGG** **UUGACCAGGUUU**U CA U

C G A **C** C- UU

PC-3p-66266-3:

UUUGGACCAGUUCGGACCAGUU

Identified by sequence similarity

Stem-loop structure:

A U CCAC U A G-| AA

GAGAAUACAA AAUUU GGCGU ACUG UCCGAAAAACUG UCCGAGCUGGUCCAAAA GU A

CUCUUAUGUU UUAAA CCGCA UGAC AGGCUUU**UUGAC** **AGGCUUGACCAGGUU**UU CA A

C C AUUU U **C** AG^ AU

PC-3p-66266-4:

UUUGGACCAGUUCGGACCAGUU

Identified by sequence similarity

Stem-loop structure:

A U CCAC U A G- AA

GAGAAUACAA AAUUU GGCGU ACUG UCCGAAAAACUG UCCGAGCUGGUCCAAAA GU A

CUCUUAUGUU UUAAA CCGCA UGAC AGGCUUU**UUGAC** **AGGCUUGACCAGGUU**UU CA A

C C AUUU U **C** AG AU

PC-3p-66266-5:

UUUGGACCAGUUCGGACCAGUU

Identified by sequence similarity

Stem-loop structure:

C U A G-| AA

GGCGUAGAGAAUCAGUGGGGGAAUACAAGAAUUUUGGCGUUAA ACUG UCCGAAAAACUGGUCC AACUGGUCCAAAA GU A

CCGCAUCUCUUAGUCACUCCCUUAUGUUCUUAAAACUGUAAUU UGAC AGGCUUU**UUGACCAGG** **UUGACCAGGUU**UU CA A

U U **C** AG^ AU

PC-3p-66266-6:

UUUGGACCAGUUCGGACCAGUU

Identified by sequence similarity

Stem-loop structure:

C UUC- U

GGUUCGAAAAACUGG CCGAACUGGUCUAAA GG U

CCAGGUUUU**UUGACC** **GGCUUGACCAGGUU**U CU U

**A** UAAA U

PC-3p-66266-7:

UUUGGACCAGUUCGGACCAGUU

Identified by sequence similarity

Stem-loop structure:

GA A C U G- AA

AAUUUGGCGUAAAGAAUCAGU GGGAAUAC AGAGUUUUGGCGUUAA ACUG UCCGAAAAACUGGUCCGAACUGGUCCAAAA GU A

UUAAACCGCAUUUCUUAGUCA CUCUUAUG UCUUAAAACCGCAAUU UGAC AGGCUUU**UUGACCAGGCUUGACCAGGUU**UU CA A

AA C U U AG AU

PC-3p-66266-8:

UUCGGACCAGUUCGGACCAGUU

Identified by sequence similarity

Stem-loop structure:

AAACC - A U - AA AA

AAC GGUCC AACU GG CC AAUUUG A

**UUG CCAGG** **UUGA** **CC GG** **UU**AAGC A

GCUUU **A** **C** - **A** **C**- CA

PC-3p-66266-9:

UUUGGACCAGUUCGAACCAGUU

Identified by sequence similarity

Stem-loop structure:

C CCA AAG AG

UUUGGCGUUAAAACU AUCCGAAAAACUGGU AACUGGUCCAAAA GU A

AAACCGCAAUUUUGA UAGGCUUU**UUGACCA** **UUGACCAGGUUU**U CA A

C **AGC** AG- GU

PC-3p-66266-10:

UUUGGACCAGUUCGGACGAGUU

Identified by sequence similarity

Stem-loop structure:

G G A UACA

AAAAACU GU CGAA UGGUCCAAAAUU \

UUU**UUGA** **CA GCUU** **ACCAGGUUU**UAG A

**G G G** CAAA

PC-3p-66266-11:

UUUGGACCAGUUCGGGCCAGUU

Identified by sequence similarity

Stem-loop structure:

C A UUUUACA

AAGAAUUUUGG GUUAAAACUGGUCCGA AAACUGGCCCGAACUGGUCCAAA \

UUCUUAAAACC CAAUUUUGACCAGGCU U**UUGACCGGGCUUGACCAGGUUU** A

A C UAGCAAA

PC-3p-66266-12:

UUUGGACCAGUUUGGACCAGUU

Identified by sequence similarity

Stem-loop structure:

AG - --- U

UCC AAAACUG UUCGAACUGGUCCAAAAU CA U

GGG UU**UUGAC** **AGGUUUGACCAGGUUU**UA GU U

CU **C** AAU U

PC-3p-66266-13:

UUUGGAUCAGUUCGGACCAGUU

Identified by sequence similarity

Stem-loop structure:

A A A AA

AAAAACUGGUCC AACUGG CC AAAUUUG A

UUU**UUGACCAGG** **UUGACU GG** **UUU**AAGC A

**C A** - CU

PC-3p-66266-14:

UUUGGUCCAGUUUGGACCAGCU

Identified by sequence similarity

Stem-loop structure:

UUUU U U UUCGGUU

GAA CU GUCCGAACUGG CCAAA \

CUU **GA CAGGUUUGACC** **GGUUU** U

U**UUC** **C U** UAAACUG

PC-5p-47339:

UAAUGGAUUCUUCGAAUU

Identified by deep-sequencing

Stem-loop structure:

UGA**UAA** **UUC** - **U** UAU-- C--- AU

**UGGAUUC GA AU** UCG AG UGUA C

ACCUAAG CU UA AGC UC ACAU U

AGUAAA UGU A C UAACU UUUA AG

PC-3p-24964-1:

UAAGUCUAUGAUUUCGUUCGGAGCAU

Identified by deep-sequencing

Stem-loop structure:

UU A GA C---- A C UUGC A - UU

UUCAG CAG UAU UGUUUCC CU CUGAAUG AAUUAUA GA UUAA AUCA \

GAGUU GUC AUA ACAAGGG **GA GGCUUGC UUAGUAU CU** **AAU**U UGGU G

UG A AC CC**UAC** - **U** ---- G G CU

PC-3p-24964-2:

UAAAUCUAUGAUUUCGUUCGGAGCAU

Identified by sequence similarity

Stem-loop structure:

CCCA--- C UUGC A - UU

GUGUUUCC CUGAAUG AAUUAUA GA UUAA AUCA \

CACAAGGG **GGCUUGC UUAGUAU** **CU** **AAU**U UGGU G

CC**UACGA** **U** ---- **A** G CU

PC-3p-15252-1:

UAGCGUAUGUUAGGCGUCGUUGAUUG

Identified by deep-sequencing

Stem-loop structure:

U- AU UUA AUGUU C AC

GCUCCAUA GAC GAC AAUCAAU CGUC AACAUACG \

UGAGGUAU CUG CU**G** **UUAGUUG** **GCGG UUGUAUGC** **U**

UU -- --- **CU**--- **A** **GA**

PC-3p-15252-2:

UAGCGUAUGUUAGGCGUCGUUGAUUG

Identified by sequence similarity

Stem-loop structure:

- AU UUA AUGUU C AC

GCUCCAUA UGAC GAC AAUCAAU CGUC AACAUACG \

CGAGGUAU ACUG CU**G** **UUAGUUG GCGG UUGUAUGC** **U**

U -- --- **CU**--- **A GA**

PC-5p-36969:

UAGAAUGUCUGUGAAUAGAAACGAGC

Identified by deep-sequencing

Stem-loop structure:

AUGCA  **G UA**--- **C** **C**C

**UAGAAUGUCU UGAA GAAA GAG** A

GUUUUACGGA ACUU CUUU UUU A

CACUG - UGGAG U UU

PC-5p-63046:

UCGUUGAUCGGCAUAUGGGUGGCGU

Identified by deep-sequencing

Stem-loop structure:

UCUUUUUUCUCAAUUUGUAUU **UU A UG U**

**UCG** **GAUCGGCAU UGGG GCG** C

AGC UUAGCUGUA ACUU UGC U

CUGUUUCGGCAUUUUUGUCUU UU A -- U

PC-3p-53754:

AGAAAUCAAGAUACUGAAAUCG

Identified by deep-sequencing

Stem-loop structure:

AUU - - -- A

GUGUCGGUU UAGU UUUUGAUUUUUGA GAAUA \

CAUA**GCUAA** **GUCA** **AGAACUAAAGA**CU CUUAU U

AC- **A U** AC U

PC-3p-55944-1:

UCAGACGAUGAAAUCGACAUA

Identified by deep-sequencing

Stem-loop structure:

--- CAUA UUUUUA

GUAA AGAUAUGUUGAUUUCAUC CUGGUUU \

CAUU UCU**AUACAGCUAAAGUAG** **GACU**AAA A

AUA **CA**-- UUUCCA

PC-3p-55944-2:

UCAGACGAUGAAAUCGACAUA

Identified by sequence similarity

Stem-loop structure:

GCGG U C UCCAUA UU UAA

UGUGUAGU GGUAUAGAU UG CGAUUUCA UCGUUU UUU \

ACACAUCA UCAUAUCU**A** **AC GCUAAAGU**  **AGCAGA** AAA A

---- **U A** ------ **CU** UUC

PC-5p-40924:

UAAUUACUGAGUUCGUUUUAUCGGAU

Identified by deep-sequencing

Stem-loop structure:

UU**UAA** - **UU** --- **AU**C

**UUACU GAG CG UUUUAUCGG** A

AGUGA CUC GC AAAAUAGUC C

UAGUC G CG CUA AGG

PC-3p-45385-1:

AUGUAUCCAGGUAUUGCUGA

Identified by deep-sequencing

Stem-loop structure:

- UAC CC **U CA A C** **A**UCGUAU-- AAA-- AA

UAUG UCUGGUGG CA **AUG AUC GGU UUG** **UG** GC AGG A

AUGC AGACCACC GU UAC UAG CUA AAC AC CG UCC U

A CC- --^ - AA A U CAACUACCC GUUAC AC

PC-3p-45385-2:

AUGUAUCCAGGCAUUGCUGA

Identified by sequence similarity

Stem-loop structure:

AAAUACU U G UG UA

UUUGUAUGC GUA UGUC GG GUAC C

AAACGUAUG **CGU ACGG CC UAUG** C

CU**AGU**-- **U A** -- **UA**

PC-5p-54448:

UUCGAGAUACGGCUUACATGACUUUU

Identified by deep-sequencing

Stem-loop structure:

AAACAACA**UUC** -- **C A** A ------ G

**GAG AUACGGCUUA AUG** C**UUU**U CGA AGA A

CUC UAUGUUGAGU UGC GGAGA GUU UCU A

GCAACUUUUAA CU U - G AAUAUG A

PC-5p-34434:

UGCAAUGGAACUUGAUGAAAGGCGUU

Identified by deep-sequencing

Stem-loop structure:

AC CUU **UGC**- **AC UG A C**--- AGA UAA

UCUCU UAU UG **AAUGGA U AUGA AGG** **GUU**UUGU UC \

AGGGA AUA GC UUAUCU A UACU UCC CAAAAUA AG U

GA ACU UUUA C- GU G AAGU AA- UUG

PC-5p-27853-1:

CCGCUGAUCGGUGAGGGGCAA

Identified by deep-sequencing

Stem-loop structure:

AGA AG AG A AU AUC

GC GC GCC CC CAUC UCA \

CG CG **CGG GG GUGG AGU C**

GA- **AA** -- **A CU CGC**

PC-5p-27853-2:

CCGCUGAUCGGUGAGGGGCAA

Identified by sequence similarity

Stem-loop structure:

**A** **GCA** A CAG AG AGA

UC**C** **CGCUGAUCGGUG** **GGG** **A**GC GG GAC GGC \

UGG GUGGCUAGUCGC CCU UUG CU CUG CCG G

A - AAC - ACA A- GAC

PC-5p-27853-3:

CCGCUGAUCGGUGAGGGGCAA

Identified by sequence similarity

Stem-loop structure:

- **UG A** **A**AGCA C AG

UC**C** **CGC AUCGGUG GGGGC** GA GGGGC \

GGG GUG UAGUCGC CCUCG CU UCUCG C

A GU - ----- C AC

PC-5p-27853-4:

CCGCUGAUCGGUGAGGGGCAA

Identified by sequence similarity

Stem-loop structure:

- **UG A** **A**AGCA C AG

UC**C** **CGC AUCGGUG GGGGC** GA GGGGC \

GGG GUG UAGUCGC CCUCG CU UCUCG C

A GU - ----- C AC

PC-5p-40470-1:

UGCAAGGCUGAGCGCUGCAACGGCAA

Identified by deep-sequencing

Stem-loop structure:

GAAUU**UGC** **G A GCAA**  AA CA

**AA GCUG GCGCU** **CGGCAA**UCAUAUG UGUGA A

UU CGAC CGCGA GCCGUUAGUAUAC ACACU A

GCCUUUUU A - AACG C- AG

PC-5p-40470-2:

UGCAAGGCUGAGCGCUGCAACGGCAA

Identified by sequence similarity

Stem-loop structure:

CA C **A**- **GC AAC**  A CA

GUAUUGUU AA U**UGCAAGGCUG** **GC UGC** **GGCAA**UCAUAUG GUGUGA A

UAUAAUAA UU AACGUUUCGAC CG ACG CCGUUAGUAUAC CACACU A

AC U CA AA A-- - AG

PC-5p-40470-3:

UGCAAGGCUGAGCGCUGCAACGGCAG

Identified by sequence similarity

Stem-loop structure:

**A** -- **AA** A AACA

U**UGCAAGGCUG** **GCGC UGC** **CGGCAG**UCAUAUG GUGU A

GACGUUUCGAC CGCG ACG GCCGUUAGUAUAC CACA A

- UA -- - CUAG

PC-5p-40470-4:

UGCUAGGCUGAGCGCUGCAACGGCAA

Identified by sequence similarity

Stem-loop structure:

C **U CUGA GCAA** A C CA

GUAUUAUUUGAA C**UGC** **AGG GCGCU** C**GGCAA**UCAUAUG GUG GA A

CGUAAUAAACUU GACG UUC CGCGA GCCGUUAGUAUAC CAC CU A

A C AAA- AACG - A AG

PC-5p-13305-1:

UUCAUAUGGGACACGCAAUUAA

Identified by deep-sequencing

Stem-loop structure:

**C** G U UAU

AUGGGGAAC**UUCAUAUGGGACA** **GCAAUUAA**UGAA UA ACUU U

UACCCCUUGAAGUAUACCCUGU CGUUAGUUAUUU AU UGAA A

A A U UUA

PC-5p-13305-2:

UUCAUAUGGGACAUGCAAUCAA

Identified by sequence similarity

Stem-loop structure:

**C** A U A AAU

AUGGGGAAC**UUCAUAUGGGACAUGCAAU** **AA**U AA UA ACUU U

UACCCCUUGAAGUAUACCCUGUGCGUUA UUA UU AU UGAA A

A C C A AUA

PC-5p-34290:

UCCGAUCGUUGUAGCAGUAUUGAUGA

Identified by deep-sequencing

Stem-loop structure:

GU-- **C UGU AUU** G

GCA **UCCGAU GU AGCAGU GAUGA** A

CGU GGGUUA CG UUGUCA CUGCU A

AUUU U CUU CGU G

PC-5p-43858-1:

GUUGUGAUUUGUAUUGGUGUU

Identified by deep-sequencing

Stem-loop structure:

- GA CAA AAAUA

UUCAA CUU GUACACACA AAACGCCUUCUGUCAUA**GUUGUGAUUUGUAUUGGUGUU** \

AAGUU GGG UAUGUGUGU UUUGUGGAAGGCAGUAUUAACACUAAACAUAACCACAA U

A A- --- AGUAU

PC-5p-43858-2:

CUUGUGAUUUGUAUUGGUGUU

Identified by sequence similarity

Stem-loop structure:

**C** C -- A AAU ACAA GCGC

UUCUGUCAUA **UUGUGAUUUGUAUUGGUGUU**U AUAAUAUUACCACCAUUACUAUGGUG AUAGU GU AUUC AG \

AAGACAGUAU AACACUAAACAUAACCAUAAA UAUUAUAAUGGUGGUAAUGAUACCAC UAUCG CA UAAG UC G

U A AG A GUU CCA- AAGC

PC-5p-53148:

UAUGACUGCACCUUUAUUUUGUAUGA

Identified by deep-sequencing

Stem-loop structure:

UU**UAUGACU** **A** ---- **UUG** U

**GC CC** **UUUAUU** U**AUGA**AUUU G

CG GG AAGUAG GUAUUUAGA G

UAUGCUCGG A CUCC UAA A

PC-5p-34685-1:

UGGAUAGUUGAUAUAUAUGU

Identified by deep-sequencing

Stem-loop structure:

CUUCU GCUC - C UUC C AUC

AAUAUG UAUCG GCUAUUCGU GA GAG AGU UUUUGC \

U**UGUAU** **AUAGU** **UGAUAGGU**A CU CUU UUA AAAACG G

**AU**--- ---- A U UA- - CUC

PC-5p-34685-2:

UGGAUAGUUGAUAUAUAUGU

Identified by sequence similarity

Stem-loop structure:

AA**UG** - **A UAU** - U GC-- A --- AUUU

**GAU AGUUG UAUA GU**UC GCA UGAAG ACU GAU UAC \

CUG UUAAU AUAU CAAG CGU GCUUC UGG CUA AUG U

UAGU U A UU- U - ACAC A ACA CUUA

PC-5p-2087:

GGGUUCAAAGGAGAGCAUGUA

Identified by deep-sequencing

Stem-loop structure:

A **C C** UGUAUUA

GUUUAUC GU**GGGGUU** **AAAGGAGAG AUGUA** \

UAAAUAG UACUCUAG UUUCCUCUC UACAU U

C C A UAGGAGU

PC-5p-57422:

UAGUGAACUGGAAGAUGUUGUGCCAU

Identified by deep-sequencing

Stem-loop structure:

A **AGUG** **G G** - **CAU**

UU GUGG**U** **AACU GAA AUGUUGU GC** \

AA CACUA UUGA CUU UGCAGCG CG U

G AA-- G G G AAG

PC-5p-39653-1:

UCAGAACGGAGCAAGACAAAUUGAUC

Identified by deep-sequencing

Stem-loop structure:

UCAUCUU**UCAGA** **CA** ---- **C**AU

**ACGGAG AGAC** **AAAUUGAU** C

UGUCUC UCUG UUUGACUA A

CAAACACUAACC -- CUCU CUG

PC-5p-39653-2:

UCAGAACGGAGCAAGACAAAUUGAUC

Identified by sequence similarity

Stem-loop structure:

AUCCU**UCA** ---- **CA** **C**GU

GAAC **GGAUCAAGA AAUUGAU** C

CUUG UCUGGUUUU UUGACUA A

A------- UUUC -- CUG

PC-5p-39653-3:

UCGGAACGGAGUAAAAUAAAUUGAUC

Identified by sequence similarity

Stem-loop structure:

**C** - **UAAA** **UU U** AAG UCCAGU- CAUAAGU

U**U** **G** **GAACGGAG AUAAA** **GA C**AU CA CAUGAGU U

AA C CUUGCCUC UGUUU UU GUA GU GUGCUCA A

C A UC-- UU - CA- UAAGAAU CUUAAAU

PC-5p-60610:

AAGGUCAAGGAAGUUGGUG

Identified by deep-sequencing

Gene not in the draft genome sequence

PC-3p-15868:

UAAGUGAAGAAUUAGAAUUUACAGA

Identified by deep-sequencing

Stem-loop structure:

- A U**U**

CAUUUUG AAAUUUUAAUU UUUU **A**

GUG**AGAC** **UUUAAGAUUAA GAAG** **A**

**A** - **UG**

PC-3p-52772:

UAGUGAACUGGAAGAUGUUGUGCCAU

Identified by deep-sequencing

Stem-loop structure:

AA UA ---- UCC- U- CAGCUC

AAGG CUGA UGGUGUAA UCGU AG AGU \

U**UCC** **GGCU ACUACGUU**  **AGCA** UC UCA A

-- **CG AUUC** **U**ACU UU CCAUUU

PC-3p-16994:

UGACUAGAUUCAAUGCUCAUCU

Identified by deep-sequencing

Stem-loop structure:

A U - ----- GUUU

UGCUUC UUCAGU GGUGAGCAU GAAUUUGGUU CAC A

GCGAGG GAGUUA **CUACUCGUA CUUAGAUCAG** GUG U

C **U** **A** **U**UCUU AAAA

PC-5p-66350-1:

UGAUGUUUGUUUAUGGGUUGAA

Identified by deep-sequencing

Stem-loop structure:

G A AUA

GUUUGAAUUG **UGAUGUUUGUUU UGGGUUGAA** C

CAAACUUAAU ACUACAAACAAA ACCCAACUU A

A C AUU

PC-5p-66350-2:

UGAUGUUUGUUUGUGGGUUGAA

Identified by sequence similarity

Stem-loop structure:

AU UAA

GUUUGAAUU **UGAUGUUUGUUUGUGGGUUGAA** U

CAAACUUAA ACUACAAACAAAUACCCAACUU G

CC UAU

PC-5p-46103:

UCCUCAGAGCUCGUAAUUUCGGCG

Identified by deep-sequencing

Stem-loop structure:

GAG**U** **GC U UUU** UUUU

**CCUCAGA UCG AA** **CGGCG** G

GGAGUUU AGC UU GCUGU U

CGUG GU - UC- GCUA

PC-5p-60000-1:

UAAGACAUUUUCGUUCGGUUUGAAU

Identified by deep-sequencing

Stem-loop structure:

--------- G UA UCUG

GCUAUUU GAGAAUGU UUG UUAUAUU \

UGA**UAAG** **CUUUUACA** **AAU** AGUAUAA U

**UUUGGCUUG** **G** UA CGUU

PC-5p-60000-2:

UAAGACAUUUUCGUUCAGUUUGAAU

Identified by sequence similarity

Stem-loop structure:

UGGAU**UA** **C G**  A-- **G** AG

UGCAAUA **AGA AUUUUC UUC** **GUUU** **AAU** U

ACGUUGU UUU UGAAAG AAG CAAA UUA A

UUAAAAG A G GUA G AA

PC-5p-43996-1:

AAUAAAUCGAUCUUUUUUGGC

Identified by deep-sequencing

Stem-loop structure:

C **AU** **U CU**  - UUUC- A

UG **A** **AAA CGAU UUUUUGG** **C**GC GC A

AC U UUU GCUA AAGAACU GCG UG A

C GG - AU A UGUCA A

PC-5p-43996-2:

AAUAAAUCGAUCCUUUUUGGC

Identified by sequence similarity

Stem-loop structure:

GUGC**AAU** **U CC** **GGC** U AAA

**AAA CGAU UUUUU** GUUU CACA \

UUU GCUA AAAAA CAAG GUGU U

AACCUGG - AU --- C CAU

PC-5p-43996-3:

AAUAAAUCGAUCUUUUUUGGC

Identified by sequence similarity

Stem-loop structure:

C **AU** **U CU** - UUUC AAA

UG **A** **AAA CGAU UUUUUGG** **C**GC GCA \

AC U UUU GCUA AAGAACU GCG UGU C

C GG - AU A ---- CAU

PC-5p-43996-4:

AAUAAAUCGAUCCUUUUUGGC

Identified by sequence similarity

Stem-loop structure:

C **AU** U CC **GGC** U AAA

UG **A** **AAA CGAU UUUUU** GUUU CACA \

AC U UUU GCUA AAAAA CAAG GUGU U

C GG - AU --- C CAU

PC-5p-43996-5:

ACUAAAUCGAUCUUUUUUGGC

Identified by sequence similarity

Stem-loop structure:

GUG**C U CU** --- UUUCAAA

**ACUAAA CGAU UUUUUG GC**GC \

UGGUUU GCUA AAAAAC CGUG A

AAUC - AU AAG UCAUUAA

PC-5p-43996-6:

ACUAAAUCGAUCUUUUUUGGC

Identified by sequence similarity

Stem-loop structure:

UG**C**- U CU - AUUU AAA

**ACUAAA** **CGAU UUUUUGG** **CG** **C**ACA \

UGGUUU GCUA AAAAACU GC GUGU U

AAUC - AU A ---- CAU

PC-3p-39779:

AUUAAAUAAAAUGAUUCGUGC

Identified by deep-sequencing

Stem-loop structure:

- U --- GU UC

AUUG UUU UUGCAU UCGUUU UUUA \

UAGC AAA AA**CGUG** **AGUAAA** AAAU G

U U **CUU AU** **UA**

PC-5p-36024:

UGGAUUGUAAACGCCGCGUAUGAAAU

Identified by deep-sequencing

Stem-loop structure:

A**UG** **A CCG** - G

AAA **GAUUGUAA CG CGUAUG** **AAAU**A \

UUU CUGACAUU GC GCGUGC UUUAU A

AGG C AA- C A

PC-3p-16219:

UCACUGGGCUUUGUUUGUCGC

Identified by deep-sequencing

Stem-loop structure:

U U UAAAUU

UUGUGCGACGAAC AGGUUCAGU GAUG U

AACA**CGCUGUUUG** **UUCGGGUCA** **CU**AC U

**U** - UAAAGU

PC-5p-31927-1:

CGGUACGGGUUUCUUUUCGAG

Identified by deep-sequencing

Stem-loop structure:

UAUCCGAA - U AA- A

GAAAA AA UCUG CG U

**CUUUU UU GGGC** **GC** U

UGCUC**GAG** **C U** **AUG** G

PC-5p-31927-2:

UGGUACGGGUUUCUUUUCGAG

Identified by sequence similarity

Stem-loop structure:

CA G UG**U** A -- **A**

GCU UUC AU **GGU CGGGUUU CUUUUCG G**

CGA AAG UA UUA GUCUAGG GAAAAGC C

-- G U-- - UU U

PC-5p-12784:

UUUGUCUUAAGCUGUACGAGGCA

Identified by deep-sequencing

Stem-loop structure:

AA**U** -- **AGC** - UG AA

CGUU **UUAUUU GUCUUA UGUA CGAGGCA**A AG U

GCAA AAUGAG CAGAAU ACAU GCUUUGUU UU U

--- AU CAA U GU AC

PC-3p-50458:

UCAGAUAUGGUGGCUAAAUUUGGAAU

Identified by deep-sequencing

Stem-loop structure:

UG CCACU-- CG-- UC- CU - --- UU

UUUUCUUG CUCGUCGGU GGAA GUUCC UUU UCAUCA AUU GUC \

AAAAGAAC GAGCAGUUA CCUU **UAAGG** **AAA GGUGGU UAG** CGG A

CU AUACAAC CAAG **UUU UC A** **ACU** CG

PC-5p-60454:

UAUUCGCAAUAGACGGAAUGGACGAU

Identified by deep-sequencing

Stem-loop structure:

UGAUUGCA**UAUU** **U** **U**-- **U**

**CGCAA AGACGGAA GGACGA** A

GUGUU UUUGCUUU CUUGCU C

CCAGUCGAGAGU C CCC U

PC-3p-24245:

UGCGAAUUAUAUGUGCCGAAGGAUGA

Identified by deep-sequencing

Stem-loop structure:

UC AA A AAAUUG U UAC AU AA--- CU

AAUGCCG UG GGAAU UUA UC GGCAC GUGUAGUUU GAA AUUGAU \

UUAUGGU AC UUUUA **AGU** **AG CCGUG UAUAUUAAG** **U**UU UAGCUG A

UU CG A **AGGA**-- - --- **CG** AGGAG CU

PC-5p-20866-1:

UGCAGAACAACGAACCAGUGGACAGC

Identified by deep-sequencing

Stem-loop structure:

AA**UGCA** **A**  - **C** **C**G CAU

**GAACAACGA CCAGUG GA** **AG** GC \

CUUGUUGCU GGUCAC CU UC CG U

CGUUGG A U C A- UUU

PC-5p-20866-2:

UGCAGAACAACGAACCAGUGGACAGC

Identified by sequence similarity

Stem-loop structure:

ACUAA**UGCA** **A** - **C** **C**G CAU

**GAACAACGA CCAGUG GA** **AG** GC \

CUUGUUGCU GGUCAC CU UC CG U

UCGUGUUGA A U C A- UUU

PC-5p-20866-3:

UGCAGAACAACGAACCAGUGGACAGC

Identified by sequence similarity

Stem-loop structure:

ACUAA**UGCA** **A** - **C C**G CAU

**GAACAACGA CCAGUG GA AG** GC \

CUUGUUGCU GGUCAC CU UC CG U

UCGUGUUGA A U C A- UUU

PC-5p-20866-4:

UGCAGAACAACGAACCAGUGGACAGC

Identified by sequence similarity

Stem-loop structure:

A**U** A A - C **C**GGCCAUCU

**GCAGA CAACGA CCAGUG GA AG** \

UGUCU GUUGCU GGUCAC CU UC U

AU C A U C UUUUUUUUU

PC-5p-20866-5:

UGCAGAACAACGAACCAGUGGACAGC

Identified by sequence similarity

Stem-loop structure:

U AA**UGCA** **A** - **C** - CC

GCA ACU **GAACAACGA CCAGUG GA AG C**GG A

CGU UGG CUUGUUGCU GGUCAC CU UC GUU U

- ------ A U C A UU

PC-5p-20866-6:

UGCAGAACAACGAAACAGUGGACAGC

Identified by sequence similarity

Stem-loop structure:

UU UACUAA**U** AA - C - CC

GA UGCA **GCAGAACAACGA CAGUG GA AG C**GG \

CU GCGU UGUCUUGUUGCU GUCAC CU UC GUU A

UC ------- AG U C A UU

PC-3p-67661:

UAUCUUUUAUCUCAUCUUCAUCUUGA

Identified by deep-sequencing

Stem-loop structure:

U AA GAUA-- - A UGA

GAU AAGAU AGAUGAA CGAGAU AAGAUGA GAUAAAA \

CUA UUCUA UCUACUU **GUUCUA UUCUACU CUAUUUU** **U**

- C- AACGA**A** **C** - **CUA**

PC-3p-20321:

UGAAGACGAUGUGUAGUACGUUGGAG

Identified by deep-sequencing

Stem-loop structure:

CAUACAG U U - - UG GU

AC UACUAC UGUUG CU UAGU AUU \

**UG** **AUGAUG GUAGC GA GUUA** UGA G

UC**GAGGU** **C U A A**  **GU** AC

PC-5p-43905:

ACAUCUUAGAAUUUGCUUUUUACCU

Identified by deep-sequencing

Stem-loop structure:

**U UUU** G---- UAU

C**ACAUCU** **AGAAUUUGCUU** **ACCU** GC C

GUGUGGA UUUUAAACGAG UGGA CG G

C --- AGCAA UUA

PC-3p-31364-1:

UAGCUGGGUGACGUGACCUGAG

Identified by deep-sequencing

Stem-loop structure:

G A UG AGAA

AACGACGUACGGAGCAGGUAACGUAUUGCUCAGGUCA GUCACCCAGC G U \

UUGCUGCAUGCCUCGUCCAUUGCAUAGC**GAGUCCAGU** **CAGUGGGUCG** **U** A U

**G** **A** GU AACU

PC-3p-31364-2:

UAGCUGGGUGACGUGACCUGAG

Identified by sequence similarity

Stem-loop structure:

G C G A UG AGAA

AUCAGAGAUUACUUUUUCAUACUGGAACC UAACGACGUACGGAGCAGA AACGUAUCGCUCAGGUCA GUCACCCAGC G U \

UAGUCUCUAAUGAAAAAGUAUGACUUUGG AUUGCUGCAUGCUUCGUCU UUGCAUAGC**GAGUCCAGU** **CAGUGGGUCG** **U** A U

A A **G** A GU AAUU

PC-3p-31364-3:

UAGCUGGGUGACGUGACCUGAG

Identified by sequence similarity

Stem-loop structure:

GA - U A C UUGA

GUAAC CGU GCG UGCAGGUAAC UACCGCUCAGGUCACGUCACCCAGC ACAU \

CAUUG GCG CGC GCGUCCAUUG AUGGC**GAGUCCAGUGCAGUGGGUCG** **U**GUA A

A- G C C **A** UUUU

PC-3p-31364-4:

UAGCUAGGUGACGUGACCUGAG

Identified by sequence similarity

Stem-loop structure:

U A UC AUAA

CAGAGGUUGC UUUUUGUACAGACACCGUA CGACGUACGGCGCAGGUAACGUACCGCUCAGGUCACGUUAC AGCUACGU \

GUCUCCAACG AAAAACAUGUCUGUGGCAU GCUGCAUGCCGCGUCCAUUGCAUGGC**GAGUCCAGUGCAGUG** **UCGAU**GUA U

U C **GA** AACU

PC-3p-31364-5:

UAGCUAGGUGACGUGACCUGAG

Identified by sequence similarity

Stem-loop structure:

U UC AUAA

CAGAGGUUGC UUUUUGUACAGACACCGUAACGACGUACGGCGCAGGUAACGUACCGCUCAGGUCACGUCAC AGCUACGU \

GUCUCCAACG AAAAACAUGUCUGUGGCAUUGCUGCAUGCCGCGUCCAUUGCAUGGC**GAGUCCAGUGCAGUG** **UCGAU**GUA U

U GA AACU

PC-3p-31364-6:

UAGCUGGGUAACGUGACCUGAG

Identified by sequence similarity

Stem-loop structure:

GU G C A A U C A GGAA

CAGAG UGUUUUUU GUACAGGAACCGUAA GAUGUGCGGCGCAG UAACGUACCG UCAG UCACGU ACCCAGCUA AU \

GUCUC ACAAAAAA CAUGUCCUUGGCAUU CUGCACGCCGCGUC AUUGCAUGGC **AGUC AGUGCA** **UGGGUCGAU** UA U

AU A A C **G** **C A** G AACU

PC-3p-31364-7:

UAGCUGAGUGAUGUGACCUGAG

Identified by sequence similarity

Stem-loop structure:

G A C G C A UG AGAA

AUCAGAGAUUAUUUUUUCAUAUUGAAACC UAAC ACGUACGAAGCAGGUAACGUA CGCUCAGGUCA GUCAC CAGC G U \

UAGUCUCUAAUGAAAAAGUAUGACUUUGG AUUG UGCAUGCUUCGUCUAUUGCAU GC**GAGUCCAGU** **UAGUG GUCG** **U** A U

A C A **G A A** GU AAUU

PC-5p-66343-1:

UAAGAGCUUGAUAUGACCGACGGUGC

Identified by deep-sequencing

Gene not in the draft genome sequence

PC-5p-66343-2:

UAAGAGUUUGAUAUGACCGACGGUGC

Identified by sequence similarity

Stem-loop structure:

AA**UA** **A** -  **CGG** U

**AGAGUUUGAU UG ACCGA** **UGC**G C

UCUUAAGCUA AC UGGCU GUGC U

CAUG - A UUA U

PC-5p-66343-3:

UAAGAGUUUGAUAUAACCGACGGUGC

Identified by sequence similarity

Stem-loop structure:

AAA**UA** **AUA CGG** AU

**AGAGUUUGAU** **ACCGA** **UGC** C

UCUUAAGCUA UGGCU GUG C

ACAUG GCA CAA CU

PC-5p-66343-4:

UAAGAGUUUGAUACGACCGACGGUGC

Identified by sequence similarity

Stem-loop structure:

AAA**UA** **A** - **CGG** AU

**AGAGUUUGAU CG ACCGA** **UGC** C

UUUUAGGCUA GC UGGCU GUG U

GCUUG - A UAA CU

PC-5p-66343-5:

UAAGAGUUUGAUACGACCGACGGUGC

Identified by sequence similarity

Stem-loop structure:

**A** **A** - **CGG** AU

UAAA**UA** **GAGUUUGAU CG ACCGA UGC** U

GUUUGU CUUAAGCUA GC UGGCU GUG U

- - A UAA CU

PC-5p-66343-6:

UAAGAGUUUGAUACGACCGACGGUGC

Identified by sequence similarity

Stem-loop structure:

A UAAUUU --------- **UU A** - **CGG** AU

GACAA AUAGU GUAAA**UA** **AGAGU GAU CG ACCGA UGC** C

UUGUU UGUCA UAUUUGU UCUUA CUA GC UGGCU GUG U

C UAUCUU UCACACAUG CU - A UAA CU

PC-5p-66343-7:

UAAGAGUUUGAUACGACCGACGGUGC

Identified by sequence similarity

Stem-loop structure:

**A A** - **CGG** AU

UAAA**UA** **GAGUUUGAU CG ACCGA UGC** U

GUUUGU CUUAAGCUA GC UGGCU GUG U

- - A UAA CU

PC-5p-66343-8:

UAAGAGUUUGAUACGACCGACGGUGC

Identified by sequence similarity

Stem-loop structure:

A**UAA** **A** - **CGG** U

**GAGUUUGAU CG ACCGA UGC**A C

CUUAAGCUA GC UGGCU GUGU U

AUUG - A UAA U

PC-5p-66343-9:

UAAGAGUUUGAUACGACCGACGGUGC

Identified by sequence similarity

Stem-loop structure:

G**UA** **U A** - **CGGUG** **C**

**AGAG UUGAU CG ACCGA** **CA** U

UCUU AGCUA GC UGGCU GU C

UUG U - A UAA-- U

PC-5p-66343-10:

UAAGAGUUUGAUACGACCGACGGUGC

Identified by sequence similarity

Stem-loop structure:

G**UA** **U A** - **CGGUG C**

**AGAG UUGAU CG ACCGA CA** U

UCUU AGCUA GC UGGCU GU C

UUG U - A UAA-- U

PC-5p-66343-11:

UAAGAGUUUGAUACAACCGACGGUGC

Identified by sequence similarity

Stem-loop structure:

AA AC GA UG U AA

UGCGCC UCG UG AU G CUC A

A**CGUGG** **AGC AC UA U GAG** **U**

**C**- **CA A**- **GU U** **AA**

PC-5p-66343-12:

UAAGAGUUUGAUACAACCGACGGUGC

Identified by sequence similarity

Stem-loop structure:

UUUAA**UA** **ACA CGG** AU

**AGAGUUUGAU ACCGA UGC** C

UCUUAAGCUA UGGCU GUG C

AUACAUG ACA UAA CU

PC-5p-66343-13:

UAAGAGUUUGAUACGACUGACGGUGC

Identified by sequence similarity

Stem-loop structure:

AAA**UA** **A** - **CGG** AU

**AGAGUUUGAU CG ACUGA UGC** U

UCUUAAGCUA GC UGGCU GUG U

UGUUG - A UAA CU

PC-5p-66343-14:

UAAGAGUUUGAUACGACUGACGGUGC

Identified by sequence similarity

Stem-loop structure:

U UAAAUUU **A A** - **CGG** AU

GUGAUAAA AAU UAAA**UA** **GAGUUUGAU CG ACUGA UGC** U

UACUAUUU UUG GUUUGU CUUAAGCUA GC UGGCU GUG U

- UUCAU-- - - A UAA CU

PC-5p-66343-15:

UAAGAGUUUGAUACAACCGACGGUGC

Identified by sequence similarity

Stem-loop structure:

AA**UA** **ACA CG** C

**AGAGUUUGAU ACCGA GUGC**AU C

UCUUAAGCUA UGGCU UAUGUG U

CAUG GCA -- C

PC-5p-66343-16:

UAAGAGUUUGAUACGAUCGACGGUGC

Identified by sequence similarity

Stem-loop structure:

**A A** - **CGG** AU

UAAA**UA** **GAGUUUGAU CG AUCGA UGC** U

GUUUGU CUUAAGCUA GC UGGCU AUG U

- - A UAA CU

PC-5p-66343-17:

UAAGAGUUUGAUACGAUCGACGGUGC

Identified by sequence similarity

Stem-loop structure:

U UA AC AA**UAA** **A** - **CGG** AU

AGAAA ACA UAU GCA **GAGUUUGAU CG AUCGA UGC** C

UCUUU UGU AUA UGU CUUAAGCUA GC UGGCU GUG C

- UC CA ----- - A UAA CU

PC-5p-66343-18:

UAAGAGUUUGAUACAACCGACGGUGC

Identified by sequence similarity

Stem-loop structure:

AA**UA** **ACA CGG** AU

**AGAGUUUGAU ACCGA UGC** C

UCUUAAGCUA UGGCU GUG C

CAUG GCA UAA CU

PC-5p-66343-19:

UAAGAGUUUGAUACGACCGACAGUGC

Identified by sequence similarity

Stem-loop structure:

AAA**UA** **A** - **CAG** AU

**AGAGUUUGAU CG ACCGA UGC** C

UCUUAAGCUA GC UGGCU GUG U

UGUUG - A UAA CU

PC-5p-66343-20:

UAAGAGUUUGAUACAACCGACGGUGC

Identified by sequence similarity

Stem-loop structure:

AA**UA**- **ACA CGG**  AU

GUA **AGAGUUUGAU ACCGA UGC** C

CAU UCUUAGGCUA UGGCU GUG C

ACAUG GCA UAA CU

PC-5p-66343-21:

UAAGAGUUCGAUACGACCGACGGUGC

Identified by sequence similarity

Stem-loop structure:

AAA**UA** **A** - ---- **UG**- U

**AGAGUUCGAU CG ACCGA CGG** **C**AU U

UCUUAAGCUA GC UGGCU GCU GUG U

GUUUG - A UAUG UAA C

PC-5p-66343-22:

UAAGAGUUCGAUACGACCGACGGUGC

Identified by sequence similarity

Stem-loop structure:

UAAA**UA** **ACG CGG** AU

**AGAGUUCGAU ACCGA UGC** C

UCUUAAGCUA UGGCU GUG U

UGCUUG CCA UAA AU

PC-5p-61169-1:

GAGCGCGGUUAGUACUUGG

Identified by deep-sequencing

Stem-loop structure:

UU**GAG** **UA A** **UG** A G

**CGCGGU** **GU** **CU G** UGG U

GCGCCA CA GG U GCC G

GCUGU -- A GU C A

PC-5p-61169-2:

GAGCGCGGUUAGUACUUGG

Identified by sequence similarity

Stem-loop structure:

UU**GA** **UA A UG** A G

**GCGCGGU GU CU G** UGG U

UGCGCCA CA GG U GCC G

GCUG -- A GU C A

PC-5p-61169-3:

GAGCGCGGUUAGUACUUGG

Identified by sequence similarity

Stem-loop structure:

UU**GA** **UA A UG** A G

**GCGCGGU GU CU G** UGG U

UGCGCCA CA GG U GCC G

GCUG -- A GU C A

PC-5p-61169-4:

GAGCGCGGUUAGUACUUGG

Identified by sequence similarity

Stem-loop structure:

UU**GA** **UA A UG** A G

**GCGCGGU GU CU G** UGG U

UGCGCCA CA GG U GCC G

GCUG -- A GU C A

PC-5p-61169-5:

GAGCGCGGUUAGUACUUGG

Identified by sequence similarity

Stem-loop structure:

UU**GA** **UA A UG** A G

**GCGCGGU GU CU G** UGG U

UGCGCCA CA GG U GCC G

GCUG -- A GU C A

PC-5p-61169-6:

GAGCGCGGUUAGUACUUGG

Identified by sequence similarity

Stem-loop structure:

UU**GA** **UA A UG** A G

**GCGCGGU GU CU G** UGG U

UGCGCCA CA GG U GCC G

GCUG -- A GU C A

PC-5p-61169-7:

GAGCGCGGUUAGUACUUGG

Identified by sequence similarity

Stem-loop structure:

UU**GA** **UA A UG** A G

**GCGCGGU GU CU G** UGG U

UGCGCCA CA GG U GCC G

GCUG -- A GU C A

PC-5p-61169-8:

GAGCGCGGUUAGUACUUGG

Identified by sequence similarity

Stem-loop structure:

UU**GA** **UA A UG** A G

**GCGCGGU GU CU G** UGG U

UGCGCCA CA GG U GCC G

UG-- -- A GU C A

PC-5p-61169-9:

GAGCGCGGUUAGUACUUGA

Identified by sequence similarity

Stem-loop structure:

UU**GA** **UA A UG** C G

**GCGCGGU GU CU A** UGG C

UGCGCCA CA GG U GCC G

GCUG -- A GU U A

PC-5p-61169-10:

GAGCGCGGUUGGUACUUGG

Identified by sequence similarity

Stem-loop structure:

UU**GA** **UG A UG** A G

**GCGCGGU GU CU G** UGG U

UGCGCCA CA GG U GCC G

GCUG -- A GU C A

PC-5p-19388:

UAAAAUGCGUGUUGUUAGUUUGACGG

Identified by deep-sequencing

Stem-loop structure:

AAAG **CGU AG C** C

**UAAAAUG GUUGUU UUUGA GG** \

GUUUUAU UAGCAA AGACU UC U

GUAU CUU GA U U

PC-5p-34092:

UAUCCAUCGUUGCCUGUAAUGGUGUC

Identified by deep-sequencing

Stem-loop structure:

**UA**---- **U**- ---- **A** **GUC**

UGAUGGA **UCCA CGUUGCCU** **GUA UGGU** \

ACUGCCU AGGU GCAAUGGG CGU ACUA C

UUUACA UU UUCU A AAG

PC-5p-27025:

UCGAUUGAAUUGUUGAGGUUGGGCAU

Identified by deep-sequencing

Stem-loop structure:

AUUUA**UCGA** **AA G** **AU**U-- UG AA C- U

**UUG UUGUUGAGGUUG GC** C A CC CAAG C

AGC AACGACUUCAAC CG G U GG GUUC A

GUUAAUACA GC G GUCGU GU A- UU A

PC-3p-44517:

UGUGUUAAUCGUUGUUGUAAUUGGUA

Identified by deep-sequencing

Stem-loop structure:

GUA GGA UU U - UU C

GCCGAU GAUA CG UUGAC CAAU UU C

**UGGUUA** **UUGU GC AAUUG** **GU**UG AA A

UU**A** AUG U- U U UU G

PC-5p-35949:

CUCUCAAAAUGACUGUGAAAUG

Identified by deep-sequencing

Stem-loop structure:

UG G **UCA A A** UUG U

ACUUU G UUAAG**CUC** **AAAUG CUGUGA** **AUG** AU C

UGAAA C GGUUCGAG UUUAC GACACU UAC UA C

GU - UAG C A UUA U

PC-3p-66589:

UGCAGACGGCAAAUUCUUUUGAGGCA

Identified by deep-sequencing

Stem-loop structure:

GCCUAUA ACA AAA U AUG

GCUUCAAAAGAG UUGUCG GC CCUUCA \

**CGGAGUUUUCUU AACGGC** **CG** GGAAGU U

UUGUUA**A** **A**-- **AGA** **U** ACC

PC-3p-6605

AUAUGAAUGAAAAAGAACAA

Identified by deep-sequencing

Gene not in the draft genome sequence

PC-5p-23638-1

AUCAGGAAUAAUCGUUUGGUC

Identified by deep-sequencing

Gene not in the draft genome sequence

PC-5p-23638-2:

AUCAGGAAUAAUCGUUUGAUC

Identified by sequence similarity

Stem-loop structure:

A AUAA G- **C**G

GAUU **UCAGGA UC UUUGAU** \

CUAA AGUUCU AG AAACUA U

A CAGG AA CU

PC-5p-23638-3:

AUCAAGAAUAAUCGUUUGGUC

Identified by sequence similarity

Stem-loop structure:

CGUGCAA U CU - UUUC- A

UAAA CGAU UUUUUGG CGC GC A

**GUUU GCUA AAGAACU** GCG UG A

AAAC**CUG** - **AU** **A** UGUCA A

PC-5p-23638-4:

AUCAAGAAUAAUCGUUUGGUC

Identified by sequence similarity

Stem-loop structure:

CGUGCAA U CU - UUUC AAA

UAAA CGAU UUUUUGG CGC GCA \

**GUUU GCUA AAGAACU** GCG UGU C

AAAC**CUG** - **AU** **A** ---- CAU

PC-3p-28519-1:

UGCAGACGGCAAAUUCUUUUGAGGCA

Identified by deep-sequencing

Stem-loop structure:

U U ACA U -- GG

GGCAAAUAUUUU UGG CAAAUAUUUGCC UUUG CC ACC U

CCGUUUGUAAGA ACC **GUUUAUAAACGG AGAC GG** **U**GG U

U **C AUC U CA** AA

PC-3p-28519-2:

UACGGUCACAUUAGGCAAAUAUUUGG

Identified by sequence similarity

Stem-loop structure:

**AC** ---- - GAA

GG**U** **GGUCACAUUA GGCAAAUAUUUGG** C \

CCA CCAGUGUGAU CCGUUUAUAAACU G A

-- CCCC U GUA

PC-3p-28519-3:

UACGGUCAGACUAGGCAAAUGUUUGC

Identified by sequence similarity

Stem-loop structure:

A**GACUA** - **U**- CAGCA

**GGCAAAUGUUUG CCCAAAAU CUUUGC** A

CCGUUUACAAAC GGGUUUUA GAAACG A

AAAAUA U CU ACAUG

PC-3p-28519-4:

UACGUUCAGACCGGGCAAAUAUUUGC

Identified by sequence similarity

Stem-loop structure:

UC U AUU UUU - A UU

AUUUUG GCGA UAUUUGCUC GUUUG CGU UC UG C

UAAAGC **CGUU** **AUAAACGGG CAGAC GCA** GG AU C

-- **U**  **C**-- **UU**- **U** A UU

PC-5p-39989-1:

AGCGAUAUUGAACCUUAUUCACU

Identified by deep-sequencing

Stem-loop structure:

**U** A A

AGCGGUGUU**AGCGAUAUUGAACCUUAUUCAC** GG AC \

UCGCCACAAUCGCUAUAACUUGGAAUAAGUG CU UG U

- A A

PC-5p-39989-2:

AGCGAUAUUGAACCUUAUUCACG

Identified by sequence similarity

Stem-loop structure:

**G**AUACU

AGCGGUGUU**AGCGAUAUUGAACCUUAUUCAC** A

UCGCCACAAUCGCUAUAACUUGGAAUAAGUG U

ACCUUG

PC-5p-39989-3:

AGCGAUAUUGAACCUUAUUCACU

Identified by sequence similarity

Stem-loop structure:

**U** A A

CGGUGUU**AGCGAUAUUGAACCUUAUUCAC** GG AC \

GCCACAAUCGCUAUAACUUGGAGUAAGUG CU UG U

- A A

PC-5p-39989-4:

AGCGAUAUUGAACCUUAUUCACU

Identified by sequence similarity

Stem-loop structure:

**C** **UA** **U**GGAAC

GUU**AG** **GAUAUUGAACCU UUCAC** A

UAAUC CUAUAACUUGGA AAGUG U

U CA CUAUUA

PC-5p-39989-5:

AGCGAUAUUGAACCUUAUUCACU

Identified by sequence similarity

Stem-loop structure:

G **U** A A

CGGU UU**AGCGAUAUUGAACCUUAUUCAC** GG AC \

GCCA AAUCGCUAUAACUUGGAAUAAGUG CU UG U

G - A A

PC-5p-39989-6:

AGCGAUAUUGAACCUUAUUCACU

Identified by sequence similarity

Stem-loop structure:

**U**G G

AGCGCUGUU**AGCGAUAUUGAACCUUAUUCAC** GAA U

UCGCGACGAUUGCUAUAACUUGGAAUAAGUG CUU U

GU G

PC-5p-39989-7:

AGCGAUAUUGAACCUUAUUCACU

Identified by sequence similarity

Stem-loop structure:

**U**G G

AGCGCUGUU**AGCGAUAUUGAACCUUAUUCAC** GAA U

UCGCGACGAUUGCUAUAACUUGGAAUAAGUG CUU U

GU G

PC-5p-39989-8:

AGCGAUAUUGAACCUUAUUCACU

Identified by sequence similarity

Stem-loop structure:

**U**G G

AGCGCUGUU**AGCGAUAUUGAACCUUAUUCAC** GAA U

UCGCGACGAUUGCUAUAACUUGGAAUAAGUG UUU U

GU G

PC-5p-39989-9:

AGCGAUAUUGAACCUUAUUCACU

Identified by sequence similarity

Stem-loop structure:

**U** A A

GGUGUU**AGCGAUAUUGAACCUUAUUCAC** GG AC \

CCACAAUCGCUAUAACUUGGAAUAAGUG CU UG U

- A A

PC-5p-39989-10:

AGCGAUAUUGAACCUUAUUCACU

Identified by sequence similarity

Stem-loop structure:

C **A** **U** A A

AG GGUGUU**AGCGAU UUGAACCUUAUUCAC** GG AC \

UC CCACAAUCGCUA AACUUGGAAUAAGUG CU UG U

U C - A A

PC-5p-39989-11:

AGCGAUAUUGAACCUUAUUCACU

Identified by sequence similarity

Stem-loop structure:

**A** G

AGCGCUGUU**AGCGAUAUUGA** **CCUUAUUCACU**AGAA U

UCGCGACGAUUGCUAUAACU GGAAUAAGUGGUUUU U

C G

PC-5p-39989-12:

AGCGAUAUUGAACCUUAUUCACU

Identified by sequence similarity

Stem-loop structure:

**U** G

AGCGCUGUU**AGCGAUAUUGAACCUUAUUCACU** GAA U

UCGCGACGAUUGCUAUAACUUGGAAUAAGUGG UUU U

U G

PC-5p-39989-13:

AGCGAUAUUGAACCUUAUUCACU

Identified by sequence similarity

Stem-loop structure:

**U** A A

CGGUGUU**AGCGAUAUUGAACCUUAUUCAC** GG AC \

GCCACAAUCGCUAUAAUUUGGAAUAAGUG CU UG U

- A A

PC-5p-39989-14:

AGCGAUAUUGAACCUUAUUCACU

Identified by sequence similarity

Stem-loop structure:

U **U** A A

AGUGC GUU**AGCGAUAUUGAACCUUAUUCAC** GG AC \

UCGCG UAAUCGUUAUAACUUGGAAUAAGUG CU UG U

U - A A

PC-5p-39989-15:

AGCGAUAUUGAACCUUAUUCACA

Identified by sequence similarity

Stem-loop structure:

**A**AUACU

GGUGUU**AGCGAUAUUGAACCUUAUUCAC** A

CCACAAUCGUUAUAACUUGGAAUAAGUG U

ACCUUG

PC-5p-39989-16:

AGCGAUAUUGAACCUUAUUCACG

Identified by sequence similarity

Stem-loop structure:

**C** **G**AUACU

CGGUGUU**AGCGAUAUUGAAC UUAUUCAC** A

GCCACAAUCGCUAUAACUUG AAUAAGUG U

U ACCUUG

PC-5p-39989-17:

UGCGAUAUUGAACCUUAUUCACU

Identified by sequence similarity

Stem-loop structure:

**U C** **U** A A

UGUU**UGCGAUAUUGAACC** **UAUU AC** GG AC \

GCAAACGUUAUAACUUGG AUAA UG CC UG U

U C - A A

PC-5p-39989-18:

AGCGAUAUUGAACCUUAUUCACG

Identified by sequence similarity

Stem-loop structure:

UU**A** **G**AUACU

**GCGAUAUUGAACCUUAUUCAC** A

CGCUAUAACUUGGAAUAAGUG U

UGC ACCUUG

PC-5p-39989-19:

AGCGAUAUUGAACCUUAUUCAUA

Identified by sequence similarity

Stem-loop structure:

U **A**G

GUGC GUU**AGCGAUAUUGAACCUUAUUCAU** U

CGCG CAAUCGUUAUAACUUGGGAUAAGUG A

U CU

PC-5p-39989-20:

ACCGAUAUUGAACCUUAUUCACG

Identified by sequence similarity

Stem-loop structure:

**A** --- **G** AAG

GUC **CCGAUA UUGAACCUUAUUCAC** GG U

CAG GGUUGU AACUUGGAAUAAGUG CU U

C UAA G AUG

PC-5p-39989-21:

AACGAUAUUGAACCUUAUUCACC

Identified by sequence similarity

Stem-loop structure:

CU**AACGA** **C**AAAAC

GCGCUG **UAUUGAACCUUAUUCAC** A

CGCGAC AUAACUUGGAAUAAGUG A

A------ UCCUUC

PC-5p-39989-22:

AUCGAUAUUGACCAUUAUUCACU

Identified by sequence similarity

Stem-loop structure:

**U** - **A** - C

GCUGUU**A** **CGAUAUUGA CC UUAUUCA** **CU**GAA G

CGACGAU GCUAUAACU GG AAUAAGU GAUUU U

U U - U A

PC-5p-39989-23:

AACGAUAUUGAACCUUAUUCAAC

Identified by sequence similarity

Stem-loop structure:

C **A** **A** **CA** A U

GCUG U**A** **CGAUAUUGA CCUUAUU** **AC**U AA A

CGAC AU GCUAUAACU GGAAUAA UGA UU C

A A G A- C G

PC-5p-39989-24:

AGCGAUAUUGGACCUUAUUCACU

Identified by sequence similarity

Stem-loop structure:

UGAA **C** **U**UGAAC

**AGCGGUGUUAGCGAUAUUGGA** **CUUAUUCAC** A

UCGCCACAAUCGCUAUAACUU GAAUAAGUG U

CGUG U CUAUGU

PC-5p-39989-25:

AGCGACAUUGAACCUUAUUCACG

Identified by sequence similarity

Stem-loop structure:

UC **C G** AAG

G **AGCGA AUUGAACCUUAUUCAC** GG U

C UUGUU UAACUUGGAAUAAGUG CU U

GA A G AUG

PC-5p-39989-26:

UGCGAUAUUGAACCCUAUUCACU

Identified by sequence similarity

Stem-loop structure:

**A C C U** A A

GUU**UGCG** **UAUUGAACC UAUU AC** GG AC \

CAAACGU AUAACUUGG AUAA UG CC UG U

C U C - A A

PC-5p-39989-27:

AGCGACAUUGAACCUUAUUCACG

Identified by sequence similarity

Stem-loop structure:

GUUGUC **C** **G**A AGU

**AGCGA** **AUUGAACCUUAUUCAC** GA \

UUGUU UAACUUGGAAUAAGUG CU U

CAGCGA A G- AUG

PC-5p-39989-28:

AGCGACAUUGAACCUUAUUCACG

Identified by sequence similarity

Stem-loop structure:

UC **C** **G**A AGU

GUUG **AGCGA AUUGAACCUUAUUCAC** GA \

CAGC UUGUU UAACUUGGAAUAAGUG CU U

GA A G- AUG

PC-3p-14036:

UAGCUGCCUCGUGAAAGACGAUA

Identified by deep-sequencing

Stem-loop structure:

A CG AA CC A AGUG U

AUGUUUAUACA UA GUC UCAC GGCA CUAAU GUUU \

UGUAAAUAUGU **AU** **CAG AGUG CCGU** **GAU**UG UAAA A

C **AG AA CU C** AA-- U

PC-5p-52354:

AGCUGGUUGGUUCCGGGUCAGAU

Identified by deep-sequencing

Stem-loop structure:

AGAUAA AAAG - U GUC **UUCC GU** C AAAU

GAGAAUAU GUG UCAAUG C GU AU**AGCUGGUUGG** GG **CAGAU** UA \

CUUUUAUG UAC GGUUAC G CA UGUCGACCAACU CC GUUUA GU U

CAAAC- GAAA U U AA- UCC- UG U AAGU

PC-5p-62589:

UAAGAACACAAGGUGACAACGGAUCU

Identified by deep-sequencing

Stem-loop structure:

AA**UAA** **C AG** - **CU**- CCUUU

**GAA ACA GUGACA ACGGAU** UCCAUC C

CUU UGU UACUGU UGCUUA AGGUGG A

ACUA- U -- C UAC UCCUC

PC-3p-66604-1:

AUCAAAUCGAAUGGAUG

Identified by deep-sequencing

Stem-loop structure:

AGUUUUA G AG A-- GCAUU

GUU CAUC CAUUC UUGGUG \

CAG **GUAG** **GUAAG** **AACUA**C U

AGUUA-- - -- **CUA** AUUAC

PC-3p-66604-2:

AUCAAAUCGAAUGGAUG

Identified by sequence similarity

Stem-loop structure:

AC **C C**- AAUCUCUU A

**AU** **AAAU GAAUGGAUG**UGCA AAC U

UA UUUA CUUACUUACAUGU UUG U

AC C AA GC------ U

PC-3p-19591-1:

UCCUGGUCAAGUACUUCUUGAGGCU

Identified by deep-sequencing

Stem-loop structure:

GGUGC CGA GAUU CAA

GCCUCAAGAA ACU CCAG \

**CGGAGUUCUU** **UGA GGUC** G

UAUG**U** **CA**- **ACU**- **CU**A

PC-3p-19591-2:

UCCUGGUCAAGUACUUCUUGAGGCU

Identified by sequence similarity

Stem-loop structure:

GGUGC GAAG UU CAA

GCCUCAAGAAG UGA CCAG \

**CGGAGUUCUUC ACU GGUC** G

UAAG**U** **AUGA** -- **CU**A

PC-3p-19591-3:

UCCUGGUCAAAUACUUCUUGAGGCU

Identified by sequence similarity

Stem-loop structure:

GGUGC GAA UU CAA

GCCUUAAGAAG UUGA CCAG \

**CGGAGUUCUUC AACU GGUC** G

UAUGU **AUA** -- **CU**A

PC-3p-19591-4:

UCCUGGUCAAGUACUUCUUGAGGCU

Identified by sequence similarity

Stem-loop structure:

- GA GAUU CAA

CCGGUGCA CCUUAAGAAG ACU CCAG \

GGCUAUG**U** **GGAGUUCUUC UGA GGUC** G

**C A**- **ACU**- **CU**G

PC-3p-19591-5:

UCCUGGUCAAGUACUUCUUGUGGCU

Identified by sequence similarity

Stem-loop structure:

GGCAGGUGC U GA GAUU CAA

GCC CAAGAAG ACU CCAG \

**CGG GUUCUUC UGA GGUC** G

UGGCUAUG**U** **U** **A**- **ACU**- **CU**A

PC-3p-19591-6:

UCCUGAUCAAGUACUUCUUGUGGCU

Identified by sequence similarity

Stem-loop structure:

CGGUGU U GA - UC CAA

GCC UAAGAAG ACU GAU CAG \

**CGG GUUCUUC UGA CUA GUC** G

CUAUG**U U A**- **A** -- **CU**A

PC-3p-19591-7:

UCCUGUUCCAGUACUUCUUGAGGCU

Identified by sequence similarity

Stem-loop structure:

CGAUGC GA AUUCC CA

GCCUUAAGAAG ACUG GG A

**CGGAGUUCUUC UGAC** **CC** G

UUAUG**U** **A**- **CUUGU** **U**A

PC-3p-19591-8:

UCCUGUUCAAGGACUUCUUGAGGCU

Identified by sequence similarity

Stem-loop structure:

UU A**UC** **CAAG** - -- **U** UUCGU- GCC

CCAG AG **CUGUU GACUU CUU GAGGC** GUA GAUUU \

GGUU UC GACGA CUGGA GAA CUCUG CGU CUAAA G

U- AUA CAA- C CC C UCCUUU GAA

PC-3p-19591-9:

UCCUGGUUAAGUACUUCUUGUGGCU

Identified by sequence similarity

Stem-loop structure:

CCUGGUGCA U GA GAUU CAA

CC UAAGAAG ACU CCAG \

**GG GUUCUUC UGA GGUC** G

GGUUAUG**UC** **U** A- **AUU**- **CU**A

PC-3p-19591-10:

UCCUGUUCCAGUACUUCUUGAGGCU

Identified by sequence similarity

Stem-loop structure:

---- GA AUUCC CA

GCCUUAAGAAG ACUG GG A

**CGGAGUUCUUC UGAC CC** G

AUG**U** **A**- **CUUGU** **U**A

PC-3p-19591-11:

UCCUGUUCCAGUACUUCUUUAGGCU

Identified by sequence similarity

Stem-loop structure:

- U GA AUUCC CA

CCGAUGC GCCU AAGAAG ACUG GG A

GGUUAUG **CGGA UUCUUC UGAC** **CC** G

**U U A**- **CUUGU** **U**A

PC-3p-11315-1:

UAUCCUUAUAUGUGUUGCUUCGGGAA

Identified by deep-sequencing

Stem-loop structure:

- UUCA C- U AA

GGACAA CCAU UCCU GGCG CAUAUAUAGGGA \

UCUGUU GGUG **AGGG UCGU** **GUGUAUAUUCCU** A

U C**A**-- **CU U** **AU**

PC-3p-11315-2:

UAUCCUUAUAUGUGUUGCUUAGGGAA

Identified by sequence similarity

Stem-loop structure:

CCAUUUCA C U AA

UCCU GGCG CAUAUAUAGGGA \

**GGGA UCGU GUGUAUAUUCCU** A

AAUUGC**AA** **U U** **AU**

PC-5p-29991:

UACUAAUCGAAAAGCCUAAUACCGG

Identified by deep-sequencing

Stem-loop structure:

C-- **CU** --- **AAG** --- **C** U UAU

GAUGUU A**UA** **AAU CGAA CCUAAU AC** **GG**C GAA U

CUAUAA UAU UUA GCUU GGAUUA UG UCG CUU U

AAU AC CUC AAA AAC C - CAA

PC-5p-17380:

AGGGGUUUCUUUCGGCCUCCCG

Identified by deep-sequencing

Stem-loop structure:

-**GG** **UU C** UUAAAU

AAAGA**A** **GGUUUC UCGGCCU CCG** \

UUUCUU UCAAAG GGCCGGA GGC A

UCA U- U UUAGGC

PC-3p-59244-1:

UCGAGAUGGUUGUUGACGAUAAUUUC

Identified by deep-sequencing

Stem-loop structure:

UUUGGUUCAUC U G G - UCA--- UG GU GCU- ACC U

CGUCGACAA CCA UUUGG CGA GC GGUGU GU UGUUGA GGCA GA G

**GCAGUUGUU GGU** **GAGCU** GCU CG UUACG CG GCGAUU CCGU CU U

UGG**CUUUAAUA** - **A** G G UAAAAG GU GU AACU AAA U

PC-3p-59244-2:

UCGAGAUGGUUGUUGACGAUAAUUUC

Identified by sequence similarity

Stem-loop structure:

UUUGGUUCAUC U G G - UCA--- UG GU GCU- ACC U

CGUCGACAA CCA UUUGG CGA GC GGUGU GU UGUUGA GGCA GA G

**GCAGUUGUU GGU GAGCU** GCU CG UUACG CG GCGAUU CCGU CU U

UGG**CUUUAAUA** - **A** G G UAAAAG GU GU AACU AAA U

PC-5p-54579:

UCGAGGAGGAAAUACGUAGCAUGGAA

Identified by deep-sequencing

Stem-loop structure:

AGUUU **GG A** --- **GAA**AAUAGAGA UC

**UCGAGGA** **AAAU CGU AGCAUG** GCGU A

AGCUCUU UUUG GUA UCGUAC CGCG A

AAUUU AA A AAC A---------- GU

PC-5p-8545:

UAGACAUGUAAAAAGGAAGAUGAAUG

Identified by deep-sequencing

Stem-loop structure:

GUU **U AG A AU**

**UAGACAUG AAAA GAAG** **UGA G**

GUCUGUAC UUUU CUUC ACU G

AGU - A- G AA

PC-3p-62130:

UGUAUGAAAUAUGGACGCGGCUGAAA

Identified by deep-sequencing

Stem-loop structure:

- A G CGAU A

GCAUUUCA UCG UGUC AUAUUUCA UCACUCU U

UGU**AAAGU** **GGC GCAG UAUAAAGU** AGUGAGA A

**C** - **G** **AUGU** U

PC-3p-17249:

UGCAUGACGACUUUUAAAUUCAUCGA

Identified by deep-sequencing

Stem-loop structure:

AAA A U UUU UAU UCCA

CGA GAGUUUAA GGUU UCA UGC \

**GCU CUUAAAUU UCAG AGU ACG** G

AA**A** **A U C**-- --- **U**CUA

PC-5p-59796-1:

AGAGGAGGUAUGAUAAUGGGG

Identified by deep-sequencing

Stem-loop structure:

C GGUCCCCGAGAAAC

CCCCCCUCCAUUAUCAUACCUCCUC AU U

GGGGG**GGGGUAAUAGUAUGGAGGAG** UA A

**A** GCUAACCGUAGUA/

PC-5p-59796-2:

AGAGGAGGUACGAUAAUGGGC

Identified by sequence similarity

Stem-loop structure:

C C **G** **C**UCAUUCC

GGGUGAUUUUUUCCUUGGACAAAAAUAAAA UAAUGAUG CAAUCGAUAG**A** **GAGGUACGAUAAUGGG** \

CCCACUAAAAAAGGAACCUGUUUUUAUUUU AUUACUAC GUUAGCUAUCU CUCCAUGCUAUUACCC A

C A A CCCAAAAC

PC-3p-150-1:

CAUACUCUCCUUUCGAUCUCA

Identified by deep-sequencing

Stem-loop structure:

A C C UGUAUUA

GUUUAUC GUGGGGUU AAAGGAGAG AUGUA \

UAAAUAG U**ACUCUAG** **UUUCCUCUC** **UAC**AU U

C **C** A UAGGAGU

PC-3p-150-2:

UAUACUCUCCUUUCGAUCUCA

Identified by sequence similarity

Stem-loop structure:

U- A U A GUCU CA

GAAUC GUC CUGGGAUCGAA GGAGA UAUACA UUUG \

CUUAG UAG G**ACUCUAGCUU** **CCUCU** **AUAU**GU AAAC U

CU C **U C** ---- UU

PC-5p-41510:

UAGUAAUGAACAAGUAAGUGCCUUAG

Identified by deep-sequencing

Stem-loop structure:

UAAAAA - AA **CUUA**

C**UAGU** **AAUG CAAGUAAGUGC G**

GGUUA UUGC GUUUAUUCACG /

UCGGAU C GA AGUG

PC-3p-2808:

UGAUUCAAAACUGAUAAAU

Identified by deep-sequencing

Stem-loop structure:

U UUUC - A

GAAUUU UU UUGAA CA A

CU**UAAA** **AG AACUU** **GU** G

**U UCAA A** U

PC-3p-1992-1:

UGAUUCAAAACUGAUGAAU

Identified by deep-sequencing

Stem-loop structure:

- U U C A A

UGAG UC UUA UUU UGGA CA A

ACU**U** **AG** **AGU AAA ACUU** **GU** G

**A U C** - **A** U

PC-3p-1992-2:

UGAUUCAAAACUGAUGAAU

Identified by sequence similarity

Stem-loop structure:

ACU - A U U C

GAGU UCA UUUU AAUUAG CUUU UU U

CU**UA** **AGU AAAA** **UUAGU**U GAAA AA G

**AGU C C** - C G

PC-3p-1992-3:

UGAUUCAAAACUGAUGAAU

Identified by sequence similarity

Stem-loop structure:

UU UA C A A

AUGAGUUU UU UUU UGGA CA A

UGCU**UAAG** **AG AAA ACUU** **GU** G

**U**- **UC** - **A** U

PC-3p-1992-4:

UGAUUCAAAACUGAUGAAU

Identified by sequence similarity

Stem-loop structure:

UC-- - A

UGAAUUUAUU UUGAA CA A

GCU**UAAGUAG** **AACUU** **GU** G

**UCAA A** U

PC-5p-55109-1:

UCGGAUUGUGGAUUGACUUU

Identified by deep-sequencing

Stem-loop structure:

--- **U A G UU**

AAGGUGG UG**UCGGAUUG GG** **UU** **AC U**

UUCCAUU GCGGCUUGGC UC AA UG /

UGA U G - CU

PC-5p-55109-2:

UCGGAUUGUGGAUUGACUUU

Identified by sequence similarity

Stem-loop structure:

--- **U A G UU**

AAGGUGG UG**UCGGAUUG** **GG UU AC U**

UUCCAUU GCGGCUUGGC UC AA UG /

UGA U G - CU

PC-3p-52837:

UCAAUUGUUUCGAUUUCGAUCUCGGC

Identified by deep-sequencing

Stem-loop structure:

AGAUUGAUGU U--- UU CCAAA

GGGGUUGAAGUCGA GUUGGA UG A

**CUCUAGCUUUAGCU** **UAACU**U AC C

ACAGCCU**CGG** **UUGU** UU UUCUU

PC-3p-62087:

UCAUUGUCGUCUUCAGUUGGCAUUUC

Identified by deep-sequencing

Stem-loop structure:

GAGUUUC AU- G UACUU UUUUGA

AAUGUCGGC AGAUGAU AAUGG UUAAAUUU \

**UUACGGUUG UCUGCUG** **UUACU**  AAUUUAAG A

UUUAA**CU** **ACU** - UU--- UUUUUA

PC-5p-46633:

CAAUACUUUGUAAGAGCUGGA

Identified by deep-sequencing

Stem-loop structure:

G--- **U U AG**--- **G**

GAUAU **UCUCAA ACU UGUA** **AGCUG A**

CUAUA AGGGUU UGG ACAU UUGAC /

AAUA - U GAUAG A

PC-5p-63727-1:

CAAACAGCCAUUAUUCAUGCA

Identified by deep-sequencing

Stem-loop structure:

UUUACA**C** **G UAU**- **A A**

**AAACA CCAU UC UGC** C

UUUGU GGUA AG ACG G

AAACCGU A UAUU A A

PC-5p-63727-2:

AAAACAGCCAUUAUUCAUGCA

Identified by sequence similarity

Stem-loop structure:

AAUUUACA **G UAU**- **A A**

**AAAACA CCAU UC UGC** C

UUUUGU GGUA AG ACG G

AGAAGCCG A UUUU A A

PC-3p-18350:

UAUUGUAUUGCUUAAGGCUUGACGUC

Identified by deep-sequencing

Stem-loop structure:

UCUUU AAU UU A

AAGAC UCGAGCUU GU UGCAAUA \

UU**CUG** **AGUUCGGA CG** **AUGUUAU** A

**C**---- AUU UU A

PC-3p-28593:

UGGGUUGAUUAGUGCAGUUAU

Identified by deep-sequencing

Stem-loop structure:

AA - U AU CG

AUGAAUGA GCG UA UUAA CG \

UGCU**UAUU** **CGU AU AGUU** **GU** A

**GA G U GG** UU

PC-3p-67441:

UCGGGAACUGCAGUUGAACGUUCUAG

Identified by deep-sequencing

Stem-loop structure:

UAUUACU U ---- UU CA

UUGGGAC UUCAAUU UU CGA \

GAUCUUG AAGUUGA AA **GCU** A

UUUACUU **C CGUC GG**  AU

PC-3p-3802:

UAUCACAUCCGAAAGGUUUCCC

Identified by deep-sequencing

Stem-loop structure:

C C A A A UUAAUUU

AC GAAG GAAAUUUU CG AUGUGA AUG A

UG CUU**C** **CUUUGGAA GC UACACU** **U**AC U

U **C** **A C A** UAGAACU

PC-3p-47103-1:

AGUGUGACAACUGCCGGACA

Identified by deep-sequencing

Stem-loop structure:

ACAUUAU CGAUUU

CACAGGGA UCCGGCAGUUGUCGCACUGAU A

GUGUUCCU **AGGCCGUCAACAGUGUGA**CUA C

CUUUA**AC** ACCGUA

PC-3p-47103-2:

AGUGCGACAACUGCCGGAAU

Identified by sequence similarity

Stem-loop structure:

A AAAUUG A CAU

CACA GGAG UCCGGCAGUUGUC CACUGAUUGG G

GUGU CCUU **AGGCCGUCAACAG** **GUGA**CUAGCU U

C GUAA**UA** **C** AAA

PC-3p-47103-3:

AGUGUGACAACUGCCGGAAA

Identified by sequence similarity

Stem-loop structure:

A AA UAUC G

AGGAA AA UUCUGGUA UCACACU A

UCCUU UU **AAGGCCGU** **AGUGUGA** U

A A**A** **CAAC** C

PC-3p-47103-4:

AGUGUGACAACUGCCGGAAA

Identified by sequence similarity

Stem-loop structure:

A C A A CGU

ACACA GGAAAAAUUUUCCG CA UU UCACACUGAUUGG G

UGUGU CCUUUUUA**AAAGGC** **GU AA** **AGUGUGA**CUAGCC U

C **C C C** AAG

PC-3p-47103-5:

AGUGUGACAACUGCCGGAAA

Identified by sequence similarity

Stem-loop structure:

G A A-- U A G A

GCA AA GAAAAA UCCGGUA UU U ACACU A

UGU UU CUUUUU **AGGCCGU** **AA** **A** **UGUGA** U

G C G**AA** **C C G** C

PC-3p-47103-6:

AGUGUGACAACUGCCGGAAA

Identified by sequence similarity

Stem-loop structure:

-- GA A U A C UU

UUACAC GGA AAAUUU UCGGCAGUUGUC CACU AU GGU A

GAUGUG CCU UUU**AAA** **GGCCGUCAACAG** **GUGA** UA CCG C

UU UC - **U**  G A UA

PC-3p-47103-7:

AGUGUGACAACUGCCGGAAU

Identified by sequence similarity

Stem-loop structure:

AG U UA UUCA G

UACAA AAAAGU UUC GCA UCACACU A

GUGUU UUUUUA **AAG** **CGU** **AGUGUGA** U

CU **U** **GC CAAC** C

PC-3p-47103-8:

AGUGUGACAACUGCCGGAAU

Identified by sequence similarity

Stem-loop structure:

C **CA**--- **C**-- -- UG

AU **AGUGUGA** **ACUGC** **GGAAU** AUUUUUCU \

UA UUACACU UGACG CUUUA UGAAAAGA U

U UACUC UUU AU UG

PC-3p-47103-9:

AGUGUGACAACUGCCGGAAU

Identified by sequence similarity

Stem-loop structure:

A UAUA G

AAAAA AUUCCGGCA UCACACU A

UUUUU **UAAGGCCGU** **AGUGUGA** U

A **CAAC** C

PC-3p-47103-10:

AGUGUGACAACUGCCGGAAU

Identified by sequence similarity

Stem-loop structure:

U-- UCU AA U U G

UACACA AAAA UUCC CA UU UCACACU A

AUGUGU UUUU **AAGG GU AA AGUGUGA** U

UCC UA**U** **CC C C** C

PC-3p-47103-11:

AGUGUGACAACUGCCGGAAU

Identified by sequence similarity

Stem-loop structure:

- CU- AA U A G

UUACACA GAAAAG UUCC CA UU UCACACU A

GAUGUGU CUUUUU **AAGG GU AA AGUGUGA** U

U UA**U** **CC C C** C

PC-3p-47103-12:

AGUGUGACAACUGCCGGAAA

Identified by sequence similarity

Stem-loop structure:

UUUUU AA U A CA UC

UUAUACAGGGGAA UUUCC CA UU UCACACUGAU GC A

AAUGUGUUCCUUU **AAAGG** **GU AA** **AGUGUGA**CUA CG C

UUUU- **CC C C** AC UA

PC-3p-47103-13:

AGUGUGACAACUGCCGGAAA

Identified by sequence similarity

Stem-loop structure:

G A UUUUAUUC ACC

UACACA GGAAAAA UUUCCG GUCA \

AUGUGU CCUUUUU **AAAGGC** **CAGU A**

A C **CGUCAA**-- GUG

PC-3p-47103-14:

AGUGUGACAACUGCCGGAAA

Identified by sequence similarity

Stem-loop structure:

-- G U A C UU

UUACACGGAGAA AAUUU UCGGCAGUUGUC CACU AU GGU A

GAUGUGUUUCUU UU**AAA** **GGCCGUCAACAG** **GUGA** UA CCG C

CU - **U** G A UA

PC-3p-47103-15:

AGUGUGACAACUGCCGGAAU

Identified by sequence similarity

Stem-loop structure:

- - AA U A G

CAC AGAAAAGGU UUCC CA UU UCACACU A

GUG UCUUUUUU**A** **AAGG GU AA AGUGUGA** U

U **U**  **CC C C**  C

PC-3p-47103-16:

AGUGUGACAACUGCCGGAAA

Identified by sequence similarity

Stem-loop structure:

C C- AA- - A G

UACACAU GAAA UUUUC CA UUG UCACAUU A

AUGUGUG CUUU **AAAAG GU AAC** **AGUGUGA** U

C UU **GCC C** - C

PC-3p-47103-17:

AGUGUGACAACUACCGGAAA

Identified by sequence similarity

Stem-loop structure:

AC U CC UU

CACAGGGAAAAAUUUUCCG GGUU UCACACUGAU GU A

GUGUUCCUUUUUA**AAAGGC** **UCAA AGUGUGA**CUA CA U

**CA C** AC CA

PC-3p-47103-18:

AGUGUGACAAUUGCCGGAAU

Identified by sequence similarity

Stem-loop structure:

UA GCU-- AA U A G

UGCACA AAA UUCC CA UU UCACACU A

AUGUGU UUU **AAGG GU AA AGUGUGA** U

UC AUUA**U** CC U C C

PC-3p-47103-19:

AGUGUCACAACUGCCGGAAA

Identified by sequence similarity

Stem-loop structure:

G A AU AA-- G UAUUUC G

ACUGG AUUGGUAC CAG AAA UU CGGUA ACACU A

UGAUU UGAUCAUG GUU UUU **AA GCCGU UGUGA** U

A C CC AUA**A** **G CAACAC** C

PC-3p-47103-20:

AGUGUGAGAACUGCCGGAAU

Identified by sequence similarity

Stem-loop structure:

A - - UAUA G

AAUAC CA GAAAAAA AUUCCGGUA UCACAUU A

UUAUG GU CUUUUUU **UAAGGCCGU AGUGUGA** U

A U A **CAAG** C

PC-3p-47103-21:

AGUGGGACAACUGCCGGAAU

Identified by sequence similarity

Stem-loop structure:

---- - - CU- AA U A A G

AAAAGU AC AC AGAAAAG UUCC CA UU UC CACU A

UUUUCA UG UG UCUUUUU **AAGG GU AA AG** **GUGA** U

CUAA U U UA**U** **CC C C G** C

PC-3p-47103-22:

AGUGUGGCAACUGCCGGAAU

Identified by sequence similarity

Stem-loop structure:

U CUU- AA- U AU G

UACACA GAAAG UUC CA UU CACACU A

AUGUGU CUUUU **AAG GU AA** **GUGUGA** U

U UUA**U** **GCC C CG** C

PC-3p-47103-23:

AGUGUGACAGCUGCCGGAAA

Identified by sequence similarity

Stem-loop structure:

C UU

ACACAGGGAAAAAUUUUCCGGCGGUUGUCACACUGAU GGU A

UGUGUUCCUUUUUA**AAAGGCCGUCGACAGUGUGA**CUA CCA C

A CA

PC-3p-47103-24:

AGUGUGACAACCGCCGGAAA

Identified by sequence similarity

Stem-loop structure:

A AGC GU

UACACA GGAAAAAUUUUCCGGC UGUCACACUGAUUGGU G

GUGUGU CCUUUUUA**AAAGGCCG** **ACAGUGUGA**CUAGCCA U

C **CCA** AA

PC-5p-56089:

UCAGAAUCGAAAAGUGACGAUGAAAU

Identified by deep-sequencing

Stem-loop structure:

AAA**UC** **U G A** GAAAUCGA

**AGAA CGAAAAGU ACGAUG** **AAU**G \

UCUU GCUUUUUA UGUUGC UUAC U

GUUUU - A C AAAAUUGA

PC-5p-41281:

AUUGGAAUAUUGCUCAAGGAAU

Identified by deep-sequencing

Stem-loop structure:

UC A **A**- **G C** CCUUCA

CAA CAAACA UGUUCGAA **UUGGAAUAUU CU AAGGAAU** U

GUU GUUUGU GUAAGCUU AGCUUUAUAG GA UUCUUUA U

C- - AA G C CAACGU

PC-3p-61755:

UUGGUGAUUUGGAAUGAA

Identified by deep-sequencing

Stem-loop structure:

ACG UG --- AAGA-- A

CCG AUGGUU UUCCA CACCGA UAC \

GGC UGCC**AA** **AAGGU** **GUGGUU** AUG G

GUA **GU** **UUA** AAAACG U

PC-3p-1586:

UAUAGACGUGGAAAAUCGUUUA

Identified by deep-sequencing

Stem-loop structure:

G UC U U U A ---- A

AUUUUU UCGGAU GAUGAGCG UUUUCCA CGU CUA ACA UUAAU A

UAAAAA AGCUUG UU**AUUUGC** **AAAAGGU GCA GAU** **U**GU AGUUA U

A UA **U** - - **A** UAAA U

PC-3p-6908:

CGGAAUUCCAACUGAUAUACGC

Identified by deep-sequencing

Stem-loop structure:

CA A U CUUUUAUG

GAAAAGU AUAUCA UUGG AAUUCUGGG \

UUUUU**CG** **UAUAGU AACC** **UUAAGGC**UU A

**CA C** - UUGAUUGU

PC-5p-57896:

UGGAAUCUUGAUCGAGCACGAAUUUC

Identified by deep-sequencing

Stem-loop structure:

**A** - **CA** **UC**A GG

AA**UGG** **AUCUUGA UCGAG CGAAUU** UGU \

UUGCC UAGAGCU GGUUC GUUUAA AUA U

G C AA UA- AG

PC-3p-36631:

UAGGAGUUUUGAUCACGGUAAAGUCA

Identified by deep-sequencing

Stem-loop structure:

AUUUGA CAA C UUUG U

ACUU CGU AUCAA UUCUGG U

**UGAA** **GCA UAGUU** **AGGAU**U U

UCCG**AC** **AUG C UUG**- U

PC-5p-36542-1:

AUUGUUUUUUCAUUCGUUUGUU

Identified by deep-sequencing

Stem-loop structure:

UCUAACCUC - **U** **UUUG**

**AUUGUUUUU** **UCA UCG U**

UAGUAAAAA GGU AGC U

UCACGUGCU U U UAUU

PC-5p-36542-2:

AUUGUUUUUUCAUUCGUUUGUU

Identified by sequence similarity

Stem-loop structure:

CUUUC**A** - **U UUUG**

**UUGUUUU UUCA UCG U**

AGUAAAA AAGU AGC U

GUGCUC U U UAUU

PC-5p-36542-3:

AUUGUUUUUUCAUUCGUUUGUU

Identified by sequence similarity

Stem-loop structure:

CUUUC**A** - **U UUUG**

**UUGUUUU UUCA UCG U**

AGUAAAA AAGU AGC U

GUGCUC U U UAUU

PC-5p-36542-4:

ACUGUUUUUUCAUUCGUUUGUU

Identified by sequence similarity

Stem-loop structure:

ACA UU--- **CUG CAU UUUG**

CGAUU UGC UC**A** **UUUUUU UCG U**

GCUAA ACG AGU AAAAAA AGC U

AUC UGCUU AA- UU- UAUU

PC-5p-36542-3:

AUUGUUUUUUCAUUUGUUUGUU

Identified by sequence similarity

Stem-loop structure:

UAAUAUUCU CU- UC- U U

AAC CAAAU GAAACGAU AAA \

**UUG** **GUUUA** **UUUUGUUA** UUU G

UAUU----- **UUU** **CUU** C U

PC-5p-57811-1

UGGGACUUCUCGAAAUUGCGGA

Identified by deep-sequencing

Stem-loop truncate

PC-5p-57811-2

UGGGACUUCUCAAAACUGCGGA

Identified by sequence similarity

Stem-loop structure:

UA**UG C A** **C** CCCA AAA

AAA **GGACUU** **UC** **AAA** **UGCGGA**AGAUAAU GCAU \

UUU CUUGAA AG UUU ACGCCUUCUAUUG CGUA A

UAGG C C A ---- AAU

**126 Known miRNAs**

mde-bantam:

UGAGAUCAUUUUGAAAGCUGAUUUU

Identified by deep-sequencing

Stem-loop structure:

C UU U GUU

ACGAAA CAGUUUUCGA UGAUUUUA CUUU U

UG**UUUU** **GUCGAAAGUU** **ACUAGAGU** GAAA U

**A UU** - AAA

mde-miR-iab-4-5p:

ACGUAUACUGAAUGUAUCCUGA

Identified by deep-sequencing

Stem-loop structure:

AA- **U C AG** CU

UCGU **ACGUAUACUGAA** **GUAU CUG** UG \

AGCA UGCAUAUGACUU CAUA GGC AU A

CAA C U CU CU

mde-miR-iab-4-3p:

CGGUAUACCUUCAGUAUACGUA

Identified by deep-sequencing

Stem-loop structure:

AA- U C AG CU

UCGU ACGUAUACUGAA GUAU CUG UG \

AGCA **UGCAUAUGACUU CAUA** **GGC** AU A

CA**A** C U CU CU

mde-miR-iab-8-5p:

UUACGUAUACUGAAGGUAUACCG

Identified by deep-sequencing

Stem-loop structure:

GUCG U AC **UU** **G A G** UAGA

GAU GUG CA UCGUG **ACGUAUACUGAA GUAU CC G**A U

CUA CAC GU AGCAU UGCAUAUGACUU CAUA GG CU A

AAA- U GA U- A - A CACG

mde-miR-1-5p (PC-5p-28286):

CCAUUCUUCCUUGCAUUCGAUA

Identified by deep-sequencing

Stem-loop structure:

A- **U C G** GUCUU A

CGAA GUU**CCAU CUUC UUGCAUUC AUA** ACA A

GCUU CGAGGUA GAAG AAUGUAAG UAU UGU U

AG U A G GC--- A

mde-miR-1-3p:

UGGAAUGUAAAGAAGUAUGGAG

Identified by deep-sequencing

Stem-loop structure:

A- U C G GUCUU A

CGAA GUUCCAU CUUC UUGCAUUC AUA ACA A

GCUU C**GAGGUA** **GAAG AAUGUAAG** **U**AU UGU U

AG **U A G** GC--- A

mde-miR-2a-5p (PC-5p-334):

GGCAUCAAAUUUGGCUGUAUUA

Identified by deep-sequencing

Stem-loop structure:

A A **G**- **U AU** U- G

GAA AUGUGU UU**G** **CAUCAAA UUGGCUGU** **UA**G GUU C

CUU UAUACA AAC GUAGUUU GACCGACA AUC UAA C

C A GA C CU UU A

mde-miR-2a-3p:

UAUCACAGCCAGCUUUGAUGAGCA

Identified by deep-sequencing

Stem-loop structure:

A A G- U AU U- G

GAA AUGUGU UUG CAUCAAA UUGGCUGU UAG GUU C

CUU UAUACA A**AC** **GUAGUUU GACCGACA** **AU**C UAA C

C A **GA C CU** UU A

mde-miR-2b-5p (PC-5p-10538):

ACUCACAAAGUGGAUGUGAAAUG

Identified by deep-sequencing

Stem-loop structure:

UU U**A** - - **A A** UUGAUA

AAUAA UCUAA **CUCA CAAAG UGG UGUGA AUG** G

UUAUU AGAUU GAGU GUUUC ACC ACACU UGC U

UC C- A G G A UUAAGA

mde-miR-2b-3p:

UAUCACAGCCAGCUUUGAUGAGCU

Identified by deep-sequencing

Stem-loop structure:

UU UA - - A A UUGAUA

AAUAA UCUAA CUCA CAAAG UGG UGUGA AUG G

UUAUU AGAU**U** **GAGU GUUUC ACC ACACU** **U**GC U

UC **C**- **A G G A** UUAAGA

mde-miR-2c-3p:

UAUCACAGCCAGCUUUGAAGAGCG

Identified by deep-sequencing

Stem-loop structure:

C - - CU UUAUUC

UCAAUUGCGUC CUUU CAAAG UGGCUGUGA UG A

AGUUGGCGUA**G** **GAGA GUUUC ACCGACACU** AC A

**C A G** **AU** UUAUUA

mde-let-7a-5p:

UGAGGUAGUAGGUUGUAUAGU

Identified by deep-sequencing

Stem-loop structure:

AA UU- **UG** **G** CG---- CA

GAA AGGC UUGCGAUG **AG UAGUAGGUUGUAUAGU**A GAUA U

CUU UUCG AGCGUUAU UC AUCGUCUAGCAUGUCAU UUAU A

AA CUU GU A CAUACA AA

mde-let-7b-5p:

AUAUGAGGUAGUAGGUUGUAUAGU

Identified by deep-sequencing

Gene not in the draft genome sequence

mde-let-7c-5p (PC-5p-19011-1):

AUGUGAGGUAGUAGGUUGUAUAGU

Identified by sequence similarity

Stem-loop structure:

UU- **UG G** CG---- CA

AGGC UUGCG**AUG** **AG** **UAGUAGGUUGUAUAGU**A GAUA U

UUCG AGCGUUAU UC AUCGUCUAGCAUGUCAU UUAU A

CUU GU A CAUACA AA

mde-miR-7b-5p:

UGGAAGACUAGUGAUUUUGUU

Identified by deep-sequencing

Stem-loop structure:

U U **U** **U U** -- - GC

GGCA AU UUGUA **GGAAGAC AG GAUUU** **UGUU**GU UUU U

UCGU UG AACAU CUUUCUG UC CUAAA ACAAUA AAG U

U U - U U AA U UU

mde-miR-8-5p:

CAUCUUACCGGGCAGCAUUAGA

Identified by deep-sequencing

Stem-loop structure:

UUC - **G C**  **A**CUA GAA

A**CAUCUU** **ACC GGCAG AUUAG** AU \

UGUAGAA UGG CUGUC UAAUC UA U

UGC A A A GCA- AAU

mde-miR-8-3p:

UAAUACUGUCAGGUAAAGAUGUC

Identified by deep-sequencing

Stem-loop structure:

UUC - G C ACUA GAA

ACAUCUU ACC GGCAG AUUAG AU \

**UGUAGAA UGG CUGUC** **UAAU**C UA U

UG**C** **A A A** GCA- AAU

mde-miR-9a-5p:

UCUUUGGUUAUCUAGCUGUAUGA

Identified by deep-sequencing

Stem-loop structure:

CC **U** **U G** G AUA

GAUGCUA GUUG **CUUUGGUUA CUAGCU** U**AUGA** UGU \

CUGCGAU UAAU GAAGCCAGU GAUCGA AUACU GCA A

UA U U A - GGU

mde-miR-9a-3p (PC-3p-2253):

UAAAGCUAGUUGACCGAAGUUA

Identified by deep-sequencing

Stem-loop structure:

CC U U G G AUA

GAUGCUA GUUG CUUUGGUUA CUAGCU UAUGA UGU \

CUGCGAU UA**AU** **GAAGCCAGU GAUCGA** **AU**ACU GCA A

UA **U U A** - GGU

mde-miR-9b-5p:

GCUUUGGUAAUCUAGCUUUAUGA

Identified by sequence similarity

Stem-loop structure:

- **AAUC U** AAUAU

UUUA AU**GCUUUGGU** **UAGCU UAUGA** G

AAAU UACGAAACCG AUCGA AUACU U

G CGAA C AUCUU

mde-miR-9c-5p:

UCUUUGGUGGUUUUAGCUGUAU

Identified by deep-sequencing

Stem-loop structure:

UU U- **C UU** UUGAAAU

UAA AGCUAUCA G**U** **UUUGGUGGUU** **AGCUGUAU**G U

AUU UCGGUAGU CA AAACCACCAA UUGAUAUAC U

UU CC - U- UAAAGUA

mde-miR-9d-5p (PC-5p-24944):

UCUUUGGUGUUCUAGCUUAUGA

Identified by deep-sequencing

Stem-loop structure:

A A **CUU**- **UU** - **A** AU

UUUGUCA CA ACU**U** **UGGUG CUAGCUU AUGAUG** AU \

AAACAGU GU UGAG ACUAC GGUCGAA UACUAC UA C

G C UGUC CG A A AG

mde-miR-9d-3p (PC-3p-30583):

UAAAGCUGGGCCAUCACUGUGA

Identified by deep-sequencing

Stem-loop structure:

A A CUU- UU - A AU

UUGUCA CA ACUU UGGUG CUAGCUU AUGAUG AU \

AACAGU GU UG**AG** **ACUAC GGUCGAA** **U**ACUAC UA C

G C **UGUC CG A** A AG

mde-miR-9e-5p:

UCUUUGGUAUUCUAGCUGUAGA

Identified by deep-sequencing

Stem-loop structure:

C C**U** **CU G** UGUUUUUA

AUUUUUG UGAG **CUUUGGUAUU AGCU UAGA**U \

UAAAAGC AUUC GAGACCAUAA UCGA AUCUA A

- UG UU A UGUGUUAU

mde-miR-9e-3p:

UAAAGCUUUAAUACCAGAGGUC

Identified by deep-sequencing

Stem-loop structure:

C CU CU G UGUUUUUA

AUUUUUG UGAG CUUUGGUAUU AGCU UAGAU \

UAAAAGC AUU**C** **GAGACCAUAA UCGA** **AU**CUA A

- **UG UU A** UGUGUUAU

mde-miR-10-5p:

ACCCUGUAGAUCCGAAUUUGUU

Identified by deep-sequencing

Stem-loop structure:

CU - G U ------ A

AUGUUCUACAU **ACC CU UAGA CCGAAUUUGUU** UGAC U

UACAGGGUGUG UGG GA AUCU GGCUUAAACAG AUUG A

UU A G U CAUAAA A

mde-miR-10-3p:

CAAAUUCGGUUCUAGAGAGGUUU

Identified by deep-sequencing

Stem-loop structure:

CU - G U ------ A

AUGUUCUACAU ACC CU UAGA CCGAAUUUGUU UGAC U

UACAGGGUGUG **UGG GA AUCU** **GGCUUAAAC**AG AUUG A

**UU** **A G U** CAUAAA A

mde-miR-11-5p:

CGGGAACUCCGAUUGUGACUGC

Identified by sequence similarity

Stem-loop structure:

GC **C C** UUUAUUAAUUUUGAA A

GAAACU CUGAG**CGGGAACUC** **GAUUGUGA** **UGC**G ACAAUUGC U

CUUUGA GACUCGUUCUUGAG CUGACACU ACGC UGUUAACG A

GU U - UAC------------ U

mde-miR-11-3p:

CAUCACAGUCUGAGUUCUUGCU

Identified by deep-sequencing

Stem-loop structure:

GC C C UUUAUUAAUUUUGAA A

GAAACU CUGAGCGGGAACUC GAUUGUGA UGCG ACAAUUGC U

CUUUGA GAC**UCGUUCUUGAG** **CUGACACU ACGC** UGUUAACG A

GU **U** - **UAC**------------ U

mde-miR-12-5p:

UGAGUAUUACAUCAGGUACUG

Identified by deep-sequencing

Stem-loop structure:

UUU **U C** CCUUUUAA

CUAUCG UUGAUG**UGAGUAU** **ACAU AGGUACUG**GU \

GAUAGC AAUUACGCUCGUA UGUA UUCGUGACCG U

UC- U - AUCUUUAA

mde-miR-12-3p (PC-3p-13166):

CAGUGCUUAUGUUAUGCUCGCA

Identified by deep-sequencing

Stem-loop structure:

UUU U C CCUUUUAA

CUAUCG UUGAUGUGAGUAU ACAU AGGUACUGGU \

GAUAGC AAUU**ACGCUCGUA** **UGUA** **UUCGUGAC**CG U

UC- **U** - AUCUUUAA

mde-miR-13a-3p:

UAUCACAGCCAUUUGAUGAGCU

Identified by deep-sequencing

Stem-loop structure:

UCA A A UUG U

GUUAAGCUC AAAUG CUGUGA AUG AU C

CGGU**UCGAG UUUAC GACACU** **U**AC UA C

**UAG C**  A UUA U

mde-miR-13b-5p (PC-5p-4157):

UCGUGAAAAUGGUUGUGCAAUG

Identified by deep-sequencing

Stem-loop structure:

UA- **G CA** CUAU AA

UUCUGUUAUCA **UCGU** **AAAAUGGUUGUG** **AUG** UC A

AAGACGGUAGU AGCA UUUUACCGACAC UAC GG U

UUG G UA UU-- AA

mde-miR-13b-3p:

UAUCACAGCCAUUUUGACGAGUU

Identified by deep-sequencing

Stem-loop structure:

UA- G CA CUAU AA

UUCUGUUAUCA UCGU AAAAUGGUUGUG AUG UC A

AAGACGGUAGU **AGCA UUUUACCGACAC** **U**AC GG U

**UUG** **G UA** UU-- AA

mde-mir-14-5p (PC-5p-121):

GGGAGCGAGAUCGGGGCUUACU

Identified by deep-sequencing

Stem-loop structure:

U **C UC U** GUCAAU U

CUAUUGUAU CGAU**GGGAG** **GAGA GGGGCU** **ACU** UGAAAU \

GGUAACGUA GUUAUCCUC CUCU UUCUGA UGA ACUUUA U

- U UU C ------ U

mde-mir-14-3p:

UCAGUCUUUUUCUCUCUCCUAU

Identified by deep-sequencing

Stem-loop structure:

U C UC U GUCAAU U

CUAUUGUAU CGAUGGGAG GAGA GGGGCU ACU UGAAAU \

GGUAACGUA GU**UAUCCUC** **CUCU UUCUGA** **U**GA ACUUUA U

- **U UU C** ------ U

mde-miR-31a-5p:

UGGCAAGAUGUCGGCAUAGCU

Identified by deep-sequencing

Stem-loop structure:

CG U- **AA C** CAU---- A

AUGCAU AA **UGGC** **GAUGU GGCAUAGCU**GA UC A

UACGUA UU ACUG CUACA CCGUAUCGGCU AG A

AA UC AC - AUCCAUU C

mde-miR-34-5p:

UGGCAGUGUGGUUAGCUGGUUGU

Identified by deep-sequencing

Stem-loop structure:

AA UAAA U **U U C** --- A

UGAA AAGGU CGCG **UGGCAG** **GUGG UAG UGGUUGU** GUG A

ACUU UUUCG GCGU ACCGUC CGCC AUC ACCGACA UAC U

AC UACG U C U - AUA A

mde-miR-34-3p (PC-3p-4640):

AGCCACUAUCCGCCCUGCCAUU

Identified by deep-sequencing

Stem-loop structure:

AA UAAA U U U C --- A

UGAA AAGGU CGCG UGGCAG GUGG UAG UGGUUGU GUG A

ACUU UUUCG GCG**U** **ACCGUC CGCC AUC** **ACCGA**CA UAC U

AC UACG **U** **C U** - AUA A

mde-miR-79-3p:

UAAAGCUAGAUUACCAAAGCAU

Identified by deep-sequencing

Stem-loop structure:

C CGC G UAGAA

UUUA AUGCUUUGG UUUAGCU UAUGA A

AAAU **UACGAAACC** **AGAUCGA** **AU**ACU C

- **AUU** **A** UUAUA

mde-miR-87a-3p:

GUGAGCAAAUUUUCAGGUGUGU

Identified by deep-sequencing

Stem-loop structure:

- G A A GU UUG-------- U AUA

ACGC UUAAC UUGAUG GCCUGAAAGUU UGCU UACCU CGC ACCA C

UGUG AAUUG AACUGU **UGGACUUUUAA ACGA** **GUG**GA GUG UGGU C

U A **G** - -- UUUAAAUUUAA C AAA

mde-miR-87b-3p:

GUGAGCAAAUUUUCAGGUGUGU

Identified by similarity

Stem-loop structure:

UU U UUU G---- UAU

CGG CACAUCU AGAAUUUGCUU ACCU GC C

GUU GU**GUGGA UUUUAAACGAG UG**GA CG G

U- **C** --- AGCAA UUA

mde-miR-92a-3p:

UAUUGCACUUGUCCCGGCCUAU

Identified by deep-sequencing

Stem-loop structure:

-- U C G GU ---- GCAU

GCAG CAAU UGCA AGG UGGGACAAG CAAUGU UGU \

CGUC GUUG GUG**U** **UCC GCCCUGUUC** **GUUAU**A ACA U

AA U **A G AC**  AGAA AAUU

mde-mir-92b-3p:

CAUUGCACUAGUCCCGGCCUGC

Identified by deep-sequencing

Stem-loop structure:

UU AUA A UGU U C C AU

AUGG UUU UUGUAGG CG CUGGUGC AU UG UAU A

UACC AAA AA**CGUCC** **GC GAUCACG UA** AC GUA U

UU A-- **G CCU U** **C** U AA

mde-mir-92c-3p (PC-3p-5899):

CAUUGCACUAGUCCCGGCCUGU

Identified by deep-sequencing

Stem-loop structure:

UU AUA A UGU U C C AU

AUGG UUU UUGUAGG CG CUGGUGC AU UG UAU A

UACC AAA AAC**GUCC** **GC GAUCACG UA** AC GUA U

UU A-- **G CCU U** **C** U AA

mde-miR-100-5p:

AACCCGUAGAUCCGAACUUGU

Identified by deep-sequencing

Stem-loop structure:

C **AA** **GA A** CUGA G

AC GA **CCCGUA UCCG** **ACUUGU**G AUU A

UG CU GGGUAU AGGC UGAACAU UGA U

C AG AC A UG-- A

mde-miR-124-3p:

UAAGGCACGCGGUGAAUGCCAA

Identified by deep-sequencing

Stem-loop structure:

UU C C AG G UUCGA

CGUUU CUC UGGUAU CACUGU GCCU UAUG U

GCAAG GAG **ACCGUA GUGGCG CGGA** **AU**AC A

C- **A** **A CA** - UUAAA

mde-miR-125a-5p:

UAAUCCCUGAGACCCUAACUUGUGA

Identified by deep-sequencing

Gene not in the draft genome sequence

mde-miR-125b-5p:

GAUUCCCUGAGACCCUAAUUUGUGA

Identified by sequence similarity

Stem loop structure:

A U**G** **UC** **U CCCU** -- GUC

UUGU GC **AU** **CC GAGA** **AAUUUGUGA** CU G

AACA CG UA GG CUCU UUAAACACU GA A

G GU U- C AGCU UU AUU

mde-miR-133a-3p:

UUGGUCCCCUUCAACCAGCUGU

Identified by deep-sequencing

Stem-loop structure:

- U GUC UUCC GU C AAAU

UCAAUG C GU AUAGCUGGUUGG GG CAGAU UA \

GGUUAC G CA **UGUCGACCAACU** **CC** **GUU**UA GU U

U U AA- **UCC**- **UG** U AAGU

mde-miR-137-5p:

ACGCGUAUUCUUGGGUUAUUA

Identified by deep-sequencing

Stem-loop structure:

C **C UG UAU** C AU

GC **ACG** **GUAUUCU GGU UA**ACA ACA \

UG UGC CAUAAGA UCG AUUGU UGU U

A A GU UU- - AA

mde-miR-137-3p:

UAUUGCUUGAGAAUACACGUAG

Identified by deep-sequencing

Stem-loop structure:

C C UG UAU C AU

GC ACG GUAUUCU GGU UAACA ACA \

U**G** **UGC CAUAAGA UCG** **AU**UGU UGU U

**A A GU UU**- - AA

mde-miR-184-5p:

CCCUUAUCAUUCUAUCGCCCCG

Identified by sequence similarity

Stem-loop structure:

CC U UA - **A** - **CCG** GAAU

GGC GC CG **CCCUUAUCA UUCU UCG CC** UGU \

CCG UG GC GGGAAUAGU AAGA GGC GG ACG U

C- U UC C - A UCA AGUU

mde-miR-184-3p:

UGGACGGAGAACUGAUAAGGGC

Identified by deep-sequencing

Stem-loop structure:

CC U UA - A - CCG GAAU

GGC GC CG CCCUUAUCA UUCU UCG CC UGU \

CCG UG GC **GGGAAUAGU AAGA GGC GG** ACG U

C- U U**C** **C** - **A** **U**CA AGUU

mde-miR-190-5p:

AGAUAUGUUUGAUAUUCUUGGUUG

Identified by deep-sequencing

Stem-loop structure:

U - **AU U** UUUUUUA

UGAUUAC GUA**A** **GAUAUGUUUGAU UCUUGG UG** \

ACUGGUG UAUU UUAUACAAACUA AGGACC AC U

U A -- C UUAUAAA

mde-miR-190-3p:

CCCAGGAAUCAAACAUAUUAUUA

Identified by deep-sequencing

Stem-loop structure:

U - AU U UUUUUUA

UGAUUAC GUAA GAUAUGUUUGAU UCUUGG UG \

ACUGGUG U**AUU** **UUAUACAAACUA AGGACC** AC U

U **A** -- **C** UUAUAAA

mde-miR-210-5p:

CUUGUGCGUGUGACAGCGGCUAU

Identified by deep-sequencing

Stem-loop structure:

AUUGC GC - U UUAGA

AGCUGCUG CAC UGCACA GA A

**UCGGCGAC GUG GCGUGU** CU U

GUG**UA** **A**- **U** **U** CAACU

mde-miR-210-3p:

AGCUGCUGGCCACUGCACAUGAU

Identified by deep-sequencing

Stem-loop structure:

AUUGC **GC** - **U** **U**UAGA

**AGCUGCUG** **CAC UGCACA GA** A

UCGGCGAC GUG GCGUGU CU U

GUGUA A- U U CAACU

mde-miR-219-5p:

UGAUUGUCCAAACGCAAUUCUUG

Identified by deep-sequencing

Stem-loop structure:

U UC **U AAC**  UGAUAUUU

UUUC AGC **UGAU GUCCA GCAAUUCUUG** \

AAAG UCG GCUG CAGGU UGUUAGGAAC U

C UU - CUA UGAACAUU

mde-miR-219-3p (PC-3p-6839):

AGGAUUGUAUCUGGACGUCGUU

Identified by deep-sequencing

Stem-loop structure:

U UC U AAC UGAUAUUU

UUUC AGC UGAU GUCCA GCAAUUCUUG \

AAAG UCG **GCUG CAGGU** **UGUUAGGA**AC U

C **UU** - **CUA** UGAACAUU

mde-miR-252-5p:

CUAAGUACUAGUGCCGCAGGAG

Identified by deep-sequencing

Stem-loop structure:

U**C** **AGU C** **AG**AGAU

CGAUUCUUU **UAAGUACU GC GCAGG** \

GUUGAGAAA AUUCGUGA CG CGUCC A

CU ACC U CCAAAU

mde-miR-252-3p:

CCUGCUGCCCAAGUGCUUAUCA

Identified by deep-sequencing

Stem-loop structure:

UC AGU C AGAGAU

CGAUUCUUU UAAGUACU GC GCAGG \

GUUGAGAA**A** **AUUCGUGA CG** **CGUC**C A

**CU** **ACC U** CCAAAU

mde-miR-263a-5p:

AAUGGCACUGGAAGAAUUCACGGG

Identified by deep-sequencing

Stem-loop structure:

- - C**A** **G UG A AU** UUG U

CCA UCAC AUGG **AUG CAC G AGA UCACGGG** AU U

GGU AGUG UACU UAC GUG U UCU AGUGCCC UA U

G A AC G GU C -- UUA A

mde-miR-263a-3p (PC-3p-8699):

CGUGAUCUCUUGGUGGCAUCAU

Identified by deep-sequencing

Stem-loop structure:

- - CA G UG A AU UUG U

CCA UCAC AUGG AUG CAC G AGA UCACGGG AU U

GGU AGUG **UACU** **UAC GUG U UCU** **AGUGC**CC UA U

G A AC G GU C -- UUA A

mde-miR-263b-5p:

CUUGGCACUGGGAGAAUUCACAG

Identified by deep-sequencing

Stem-loop structure:

UA G **CU** **UG A**  U- AU

UUGU UG UGAAU **UGGCAC GGAGA UUCACAG** UGUU U

AACA AC ACUUA ACCGUG UUUCU AGGUGUC AUAA U

UC A AC UU - UU GU

mde-miR-274-5P:

UUUGUGACCGACACUAACGGGU

Identified by deep-sequencing

Stem-loop structure:

G U**U**-- **C A CU** GUG- UA

UUCGU GCAGUUUUA **UUGUGA CG CA AACGGGU**A AUU U

AAGCA CGUUAAAGU AACACU GC GU UUGCUCAU UAG U

A CACC A - UU AUCA UU

mde-mir-275-3P:

UCAGGUACCUGAAGUAGCGCGCG

Identified by deep-sequencing

Stem-loop structure:

AG- G A --- UCUCUUUCUCUCCGA

CGCGCGCUA CAGG ACC UGGCU UUGA A

GC**GCGCGAU** **GUCC UGG** **ACU**GA AACU U

**GAA A** - UUA UACGAUUAGCGACAU

mde-miR-276a-5p:

AGCGAGGUUUAGAGUUCCUACG

Identified by deep-sequencing

Stem-loop structure:

AA C **A UUA** UUCAAU

AGAACAU CCAG **AGCG** **GGU GAGUUCCUACG** A

UUUUGUA GGUU UCGU CCA UUCAAGGAUGC U

A- C G UAC UUAAUU

mde-miR-276a-3p:

UAGGAACUUCAUACCGUGCUCU

Identified by deep-sequencing

Stem-loop structure:

AA C A UUA UUCAAU

AGAACAU CCAG AGCG GGU GAGUUCCUACG A

UUUUGUA GGU**U** **UCGU CCA** **UUCAAGGAU**GC U

A- **C G UAC** UUAAUU

mde-miR-276b-3p:

UAGGAACUUAAUACCGUGCUCU

Identified by deep-sequencing

Gene is not in the draft genome sequence

mde-miR-277-3P:

UAAAUGCACUAUCUGGUACGACA

Identified by deep-sequencing

Stem-loop structure:

A- - UAAAAAU

GUUUUGGAGU CGUAUCAGA AGUGCGUUUGCA \

CGAGACCUC**A** **GCAUGGUCU** **UCACGUAAAU**GU U

**CA A** UCAUAAC

mde-miR-278-5p (PC-5p-9391):

CCGGAUGACAGUCUCAAACGGUC

Identified by deep-sequencing

Stem-loop structure:

- A AU C CA G C**C** **C AA** GU A

GCGAU UUA AUA UU AAUAAG UG UACA **CGGAUGA AGUCUCA** **CGGUC** UAU A

CGUUA AAU UAU AA UUAUUC AC AUGU GCCUGCU UCAGGGU GCUAG AUA U

U A AC C -- A UU U G- AC U

mde-miR-278-3p:

UCGGUGGGACUUUCGUCCGUUU

Identified by deep-sequencing

Stem-loop structure:

- A AU C CA G CC C AA GU A

GCGAU UUA AUA UU AAUAAG UG UACA CGGAUGA AGUCUCA CGGUC UAU A

CGUUA AAU UAU AA UUAUUC AC AUG**U** **GCCUGCU UCAGGGU** **GCU**AG AUA U

U A AC C -- A **UU** **U G**- AC U

mde-miR-279a-3p:

UGACUAGAUCCACACUCAUUA

Identified by deep-sequencing

Stem-loop structure:

- A AA ------ G UGA

CUC AUUGUUUUUGAUGAGUG GGG UAGUU UC CAUUA A

GAG UAAUAAAA**AUUACUCAC** **CCU AUCAG** AG GUAAU U

A **A AG** **U**GUUCA - CCA

mde-miR-279b-5p (PC-5p-18554):

GAUGAGUGUUAGUUUGGUGCAUA

Identified by deep-sequencing

Stem-loop structure:

A **U** **G** --- AUU

UGC AUG**GAUGAGUGU** **AGUUUGGU** **CAUA** UUCA \

ACG UACCUACUCACA UUAGAUCA GUAU AAGU A

G U - UAA AGA

mde-miR-279c-3p:

UGACUAGAUUUACACUCAUCCA

Identified by deep-sequencing

Stem-loop structure:

UUU A U G --- AUU

GGU UGC AUGGAUGAGUGU AGUUUGGU CAUA UUCA \

CCA ACG U**ACCUACUCACA** **UUAGAUCA** **GU**AU AAGU A

UAU G **U** - UAA AGA

mde-miR-279d-3p:

UGACUAGAUUUUCACUCAUCU

Identified by deep-sequencing

Stem-loop structure:

AG U C U G UUUAUA

UAAUUUU CGGUGGAUG GUG AA UCUAGU CAUG \

AUUAAAG GCUAU**CUAC** **CAC UU AGAUCA** **GU**AC U

AA **U U U** - UUACAG

mde-miR-281-1-3p:

CUGUCAUGGAAUUGCUCUCUUU

Identified by deep-sequencing

Stem-loop structure:

UGAAA UA- C A AU

CGAAUA AUGAAGAGAGC UCCGU GACAGU GGG U

GCUUGU UA**UUUCUCUCG** **AGGUA** **CUGUC**A CUU U

CAUC- **UUA** - - AG

mde-miR-281-2b-5p:

AAGAGAGCUAUCCGUCGACAGU

Identified by deep-sequencing

Stem-loop structure:

UGAAA **UA**- **C** A AU

CGAAUA AUG**AAGAGAGC** **UCCGU** **GACAG**U GGG U

GCUUGU UAUUUCUCUCG AGGUA CUGUCA CUU U

CAUC- UUA - - AG

mde-miR-281-2b-3p:

UGUCAUGGAAUUGCUCUCUUUAU

Identified by deep-sequencing

Stem-loop structure:

UGAAA UA- C A AU

CGAAUA AUGAAGAGAGC UCCGU GACAGU GGG U

GCUUGU **UAUUUCUCUCG AGGUA CUGU**CA CUU U

CAUC- **UUA** - - AG

mde-miR-283-5p (PC-5p-176):

CAAUAUCAAUUGGUAAUUCUGG

Identified by deep-sequencing

Stem-loop structure:

C **CA** **A U** CUUUUAUG

CAUUU GAAAAGU **AUAUCA UUGG AAUUCUGG**G \

GUAAA UUUUUCG UAUAGU AACC UUAAGGCUU A

- CA C - UUGAUUGU

mde-miR-285-5p:

UAGCACCAUUCGAAAUCAGUCC

Identified by deep-sequencing

Stem-loop structure:

A U A GG AA UUG--------- C -- GCAAAU

AUCCG UU GAG ACUGA UCGAGUGGUG UAGA UUG CAUUUG UUGAAU \

UAGGC AA UU**C** **UGACU AGCUUACCAC** **AU**CU AAC GUAAAC AACUUA A

- U C AA G- UAAAGGUUUUAA - UU AAGUAA

mde-miR-286-3p:

UGACUAGACCGAACACUCGCGU

Identified by deep-sequencing

Stem-loop structure:

U AA-- - A UUGCAAU

UUUAUAAG CAGGGCG UGU CGGUUUGGU ACUG A

AGAUAUUU GUCC**UGC** **ACA GCCAGAUCA** **U**GAC A

U **GCUC A G** CUAUAAU

mde-miR-304-5p (PC-5p-5983):

AAAUCUCAAAUUGUAAUUGUGGG

Identified by deep-sequencing

Stem-loop structure:

U G **A** **A U** - AA UGU

UGCAUU UAU GAG **AAUCUCAA UUGUAAU GUGGG** CG UA \

ACGUGA AUG CUC UUGGGGUU GACGUUG CACCU GC GU A

U G A A - A GA UAA

mde-miR-304-3p (PC-3p-760):

CACGUUGCAGAUUGGGGUUACU

Identified by deep-sequencing

Stem-loop structure:

U G A A U - AA UGU

UGCAUU UAU GAG AAUCUCAA UUGUAAU GUGGG CG UA \

ACGUGA AUG C**UC** **UUGGGGUU GACGUUG** **CAC**CU GC GU A

U G **A A**  - A GA UAA

mde-miR-305-5p:

AUUGUACUUCAUCAGGUGCUCUGG

Identified by deep-sequencing

Stem-loop structure:

- CU**AU** **U G CU** AAUA

GUC CCAUGU **UGUACUUCA CA GUGCU** **GG**U U

CAG GGUGUA ACAUGAGGU GU CACGG CCA U

U ACUC U G C- ACGU

mde-miR-305-3p (PC-3p-1975):

CGGCACGUGUUGGAGUACACUCA

Identified by deep-sequencing

Stem-loop structure:

- CUAU U G CU AAUA

GUC CCAUGU UGUACUUCA CA GUGCU GGU U

CAG GGUGUA **ACAUGAGGU GU CACGG** CCA U

U **ACUC U G** **C**- ACGU

mde-miR-306-5p:

UCAGGUACUGAGUGACUCUCAG

Identified by deep-sequencing

Stem-loop structure:

U C G**U** **G U**- **G** -- U

GAAAAU GCU G **CAG UAC GAGU** **ACUCUCAG** UAAAU \

CUUUUA CGA C GUC AUG CUCG UGAGAGUC AUUUA C

- A UG G UU - UU A

mde-miR-306-3p (PC-3p-22886):

GAGAGUGCUCUUGUAGCUGGUC

Identified by deep-sequencing

Stem-loop structure:

U C GU G U- G -- U

GAAAAU GCU G CAG UAC GAGU ACUCUCAG UAAAU \

CUUUUA CGA **C** **GUC AUG CUCG UGAGAG**UC AUUUA C

- A **UG G UU** - UU A

mde-miR-307-5p:

ACUCACUCAACCUGGGUGUGAUG

Identified by deep-sequencing

Stem-loop structure:

A U **A** **CCU G** C A

UCA UUUUA UC **CUCACUCAA GG UGUGAUG** UUU \

AGU AGAAU AG GAGUGAGUU CC ACACUAC AAG U

- C C CCU A C U

mde-miR-307-3p:

CACAACCUCCUUGAGUGAGCGA

Identified by deep-sequencing

Stem-loop structure:

A U A CCU G C A

UCA UUUUA UC CUCACUCAA GG UGUGAUG UUU \

AGU AGAAU **AG** **GAGUGAGUU CC** **ACAC**UAC AAG U

- C **C CCU A** C U

mde-miR-308-3p:

AAUCACAGGAGUAUACUGUGAG

Identified by deep-sequencing

Stem-loop structure:

G UU C UG UU

GUUUGGUUCGAUGUCUCGCAGUAUAU CUUGUG AUUGA G U \

UAGAUUAGGUUAUA**GAGUGUCAUAUG** **GGACAC** **UAA**CU C G G

**A** -- - GU AA

mde-miR-315-5p (PC-5p-607):

UUUUGAUUGUUGCUCGAAAUUC

Identified by deep-sequencing

Stem-loop structure:

CUAUCA **C**  **UU** UAU

UGUGACAC U**UUUGAUUGUUG** **UCGAAA**  **C**CGUG \

ACACUGUG GAAAUUAAUAAU AGCUUU GGUAU U

UAAACA A C- UGU

mde-miR-316-5p:

UGUCUUUUUCUGCUUACUGCCG

Identified by sequence similarity

Stem-loop structure:

UC**U** **UGCUUAC** **C** -- UA

**GUCUUUUUC UGC G**UC UAUU C

CGGAGAAAG ACG CAG AUAA U

ACU UCAA--- A CU UU

mde-miR-317-5p (PC-5p-5576):

UGGGGUACUCCCUGUGGUCGCU

Identified by deep-sequencing

Stem-loop structure:

UG - -- G UAUUUUUAA AA

CAAUU C**UGGGGUACU** **CC CUGUG** **UCGCU**U AUGU \

GUUAA GACUCUAUGG GG GACAC AGUGAA UACA U

GU U UC A --------- AC

mde-miR-317-3p:

UGAACACAGCUGGUGGUAUCUCAGU

Identified by deep-sequencing

Stem-loop structure:

UG - -- G UAUUUUUAA AA

CAAUU CUGGGGUACU CC CUGUG UCGCUU AUGU \

GUUAA **GACUCUAUGG GG GACAC** **AGU**GAA UACA U

G**U** **U UC A** --------- AC

mde-miR-375-3p (PC-3p-468):

UUUGUUCGUUUGGCUUGAGUUAA

Identified by deep-sequencing

Stem-loop structure:

- - AAUU U UUU UGCG A

AACAA GAU GAUG ACUUAA CCAAAUGAA CAAACUG UUUAA \

UUGUU CUA CUAC **UGAGUU** **GGUUUGCUU** **GUUU**GGU AAGUU C

U U GU**AU** **C** --- UA-- A

mde-miR-927-5p:

UUUAGAAUUCCUACGCUUUAUC

Identified by deep-sequencing

Stem-loop structure:

AGU UG G **CU AU** AUUAAACG AUA

GAA UU UUUG UUUGC**UUUAGAAUUC** **ACGCUUU** **C**A AUGAAA \

CUU AA AAGC AAACGAAGUCUUAGG UGCGAAA GU UAUUUU A

AUU GU A UU CG CGCUAA-- GCA

mde-miR-927-3p:

CAAAGCGUUUGGAUUCUGAAGC

Identified by deep-sequencing

Stem-loop structure:

AGU UG G CU AU AUUAAACG AUA

GAA UU UUUG UUUGCUUUAGAAUUC ACGCUUU CA AUGAAA \

CUU AA AAGC AAA**CGAAGUCUUAGG** **UGCGAAA** GU UAUUUU A

AUU GU A **UU** **C**G CGCUAA-- GCA

mde-miR-929-5p:

AAAUUGACUCUAGUAGGGAGU

Identified by deep-sequencing

Stem-loop structure:

U AA **A AG** CUUGUAA

ACCUAU UG AUC**AA** **UUGACUCU UAGGGAGU**C \

UGGAUA AC UAGUU AACUGAGG AUCCCUCAG C

U AG A CA UUAAAAA

mde-miR-932-5p:

UCAAUUCCGAAGUGCAUUGCAGU

Identified by deep-sequencing

Stem-loop structure:

U **A AA A** GUUAAAACA

UUGGAGACGGC **UCA** **UUCCG GUGC UUGCAGU** U

AAUCUUUGUCG AGU AGGGC CACG AACGUCA G

U G GG - AAAAAGAUA

mde-miR-957-5p (PC-5p-24815):

GUUAGUUUUGAACGGGUUUUGGU

Identified by deep-sequencing

Stem-loop structure:

UG **G** **A G UG** A C A

UCUG GGC **UUAGUUUUG ACGG UUU GU** UG AUUU U

AGAC UCG AGUCAAAAC UGCC AAA UA AC UAAA U

GU G C - GU A - A

mde-miR-957-3p:

UGAAACCGUCCAAAACUGAGGC

Identified by deep-sequencing

Stem-loop structure:

UG G A G UG A C A

UCUG GGC UUAGUUUUG ACGG UUU GU UG AUUU U

AGAC U**CG** **AGUCAAAAC UGCC AAA** UA AC UAAA U

GU **G C** - **GU**  A - A

mde-miR-965-3P (PC-3p-49057):

UAAGCGUAGAGCUUUUCCCCUUUU

Identified by deep-sequencing

Stem-loop structure:

CAC A U UUGCCAG

UAUCGAAGGGG GGCU UACG CUUAUG U

AUAG**UUUCCCC** **UCGA AUGC** **GAAU**AC U

**UUU G** - UUAUAAG

mde-miR-970-3P:

UCAUAAGACACACGCGGCUAU

Identified by deep-sequencing

Stem-loop structure:

A UC U UUGAAUG

CAUUGGCCGUAGCUG CGU GUCUUAU GGUA A

GUAGCCGG**UAUCGGC** **GCA CAGAAUA** **CU**AU U

- **CA** - UAAAACA

mde-miR-971-3P:

UUGGUGUUAUAUCUUACAGUGAG

Identified by sequence similarity

Stem-loop structure:

- - U U G A ACA UUGUAAA

GAUG AUA CGCUA UC GCU GCUGUAAGA AUAA CAGGC \

CUAU UAU GUGGU AG C**GA** **UGACAUUCU UAUU** **GUU**CG U

A A U U **G A GUG** UUGAAAC

mde-miR-981-3P (PC-3p-26480):

UUCGUUGUCGACGAAACCUUUA

Identified by deep-sequencing

Stem-loop structure:

UGAAUC - A C GUUAUAAA

GACAAUGUA GGGUUUCG CGAUAA CG AC \

CUGUUACAU **UCCAAAGC GCUGUU GC** **U**G U

UGC**AUU** **A** - **U** ACUAAAAG

mde-miR-989-3P:

UGUGAUGUGACGUAGUGGAU

Identified by deep-sequencing

Stem-loop structure:

U A- CG C U U - AAUU

AAUCA AAG GAA CCAUUGC UCAC AUCAC UG GU U

UUAGU UUC CU**U** **GGUGAUG AGUG UAGUG** AC CA A

U AC **A**- **C** - **U** A AUUU

mde-miR-993a-5P:

UACCCUGUAGUUCCGGGCUUUU

Identified by deep-sequencing

Stem-loop structure:

- GC UC **C UUC** --------- U

CU CCU GUGA **UACC** **UGUAG** **CGGGCUUUU**G UUGUUUU U

GA GGG CACU AUGG ACAUC GCUCGAAGAC AAUAAGA A

U A- CU - UCU UAUCUUUUU A

mde-miR-993a-3P:

GAAGCUCGUCUCUACAGGUAUCU

Identified by deep-sequencing

Stem-loop structure:

- GC UC C UUC --------- U

CU CCU GUGA UACC UGUAG CGGGCUUUUG UUGUUUU U

GA GGG CAC**U** **AUGG ACAUC** **GCUCGAAG**AC AAUAAGA A

U A- **CU** - **UCU** UAUCUUUUU A

mde-miR-993b-5P:

UACCCUGUAGCUCCGGGCUUUU

Identified by deep-sequencing

Gene not in the draft genome sequence

mde-miR-994-5p (PC-5p-808):

UAUCACAGUUGCUAUUUCUGUCA

Identified by deep-sequencing

Stem-loop structure:

U A- AAAAA **CA AUU**- **CA**

UGGUU GUCGU UUU CCA**UAUCA** **GUUGCU UCUGU** G

ACCAA CAGCG AAA GGUAUAGU CAACGA AGGCG A

- AG GAA-- UA GCUU UA

mde-miR-998a-3p:

UAGCACCAUGAGAUUCAGCUC

Identified by deep-sequencing

Stem-loop structure:

CUA C A AU UUGAUAA

GAAUUC UCAG GCUGAA UCUCGUGG CUGCA \

CUUGAG AGU**C** **CGACUU** **AGAGUACC GAU**GU U

UAC **U** - **AC** CAAAAAG

mde-miR-999-3p:

UGUUAACUGUAAGACUGUGUCU

Identified by deep-sequencing

Stem-loop structure:

G CAA - UG A C AC - UA

GA GA UACA GU ACG AGACAUAGUCU ACAG AAUAUU GUG \

CU CU AUGU CA UG**U** **UCUGUGUCAGA UGUC** **UUGU**AA CGC U

A AAC A GU **C A AA**  U GU

mde-miR-1000-5p (PC-5p-3410):

AUAUUGUCCUGUCACAGCAUUAG

Identified by deep-sequencing

Stem-loop structure:

- - UA U**AUAU** **UC** UUUG A

GGA GGC UUGA ACAAU **UGUCCUG** **ACAGCAUUAG** GAU \

CUU UCG AACU UGUUA ACAGGGC UGUUGUAAUU UUA U

G U C- CAAUU UU UCAA A

mde-miR-1000-3p (PC-3p-7013):

AAUGUUGUUUCGGGACAUUA

Identified by deep-sequencing

Stem-loop structure:

- - UA UAUAU UC UUUG A

GGA GGC UUGA ACAAU UGUCCUG ACAGCAUUAG GAU \

CUU UCG AACU UGUUA **ACAGGGC** **UGUUGUAA**UU UUA U

G U C- CA**AUU** UU UCAA A

mde-miR-1174-3p:

UCAGAUCUAACAAUACCCACUUC

Identified by sequence similarity

Stem-loop structure:

A C A CG-- GC AU C GAGCAUUUUUU

UCG UUUGUAUUCAA AGGUGG UAU AGAUC UGACAUU UU GAA G

AGC AAAUAUGAGUU **UUCACC AUA UCUAG** **ACU**GUAA AA CUU /

C **C** **C ACAA** -- GU - GGAAGGCCACC

mde-miR-1175-5p (PC-5p-8731):

AAGUGGAGCAGUGGUCUCAUCG

Identified by deep-sequencing

Stem-loop structure:

GUGA - **C UC** **A** **G** AGU

UGUGUAC AGU**AAGU** **GGAG AGUGG UC** **UC** CUUU U

ACACAUG UUAUUCA UCUU UCACC AG AG GAAA U

GAGG A U UU - A AAG

mde-miR-1175-3p (PC-3p-1632):

AGAGAUUCCACUUUUCUAACUUAU

Identified by deep-sequencing

Stem-loop structure:

GUGA - C UC A G AGU

UGUGUAC AGUAAGU GGAG AGUGG UC UC CUUU U

ACACAUG U**UAUUCA** **UCUU UCACC AG AG** GAAA U

GAGG **A U UU** - **A** AAG

mde-miR-2765-5p:

UGGUAACUCCACCACCGUUGG

Identified by deep-sequencing

Stem-loop structure:

A C **A** - **CA** AUGAAU

UUUGG AC **UGGUA CUCCA C CCGUUGG**C A

AAGCC UG GCCAU GAGGU G GGUAACCG U

G C C U AA AAAAUA

mde-miR-2765-3p (PC-3p-30302):

CAAUGGAAGUUGGAGCUACCGCG

Identified by deep-sequencing

Stem-loop structure:

A C A - CA AUGAAU

UUUGG AC UGGUA CUCCA C CCGUUGGC A

AAGCC U**G** **GCCAU GAGGU G** **GGUAAC**CG U

G **C C U AA** AAAAUA

mde-miR-2779-3p:

UCCGGUUCGAAGGACCAU

Identified by deep-sequencing

Stem-loop structure:

ACUAUCCAAUUUUUUUUUUUUUGAAA C UCACCGA

UUUGAGCC GA A

**AAGCUUGG CU** A

(Gene truncate at 3’) **C** CUAAUUC

mde-miR-2796-3p:

GUAGGCCGGUGAAACUACUUUC

Identified by deep-sequencing

Stem-loop structure:

- UU C UUAAAU

AAAGAAGG GGUUUC UCGGCCU CCG \

UUU**CUUUC** **UCAAAG GGCCGGA** **G**GC A

**A U**- **U** UUAGGC

mde-miR-2838-5P:

UACGGGUUGAAAUGGUUUAAAUUCAG

Identified by deep-sequencing

Stem-loop structure:

**A** **AAUGGU A**  U

GUA**U** **CGGGUUGA UUAA UUCAG**CCUU A

CGUA GUCCGACU AAUU AAGUCGGAG U

A AU---- A A

mde-miR-2944-5p:

AAGGAACUCCCGGUGUGAUAU

Identified by deep-sequencing

Stem-loop structure:

C A A**A** - **CC** - UU C

GUUAUC AG UA **AGG AACU CGG** **UGUGAUAU**G CAA A

CAAUAG UU AU UCC UUGA GUC ACACUAUAC GUU U

A A CG A A- G C- U

mde-miR-2944-3p:

UAUCACAGCUGAAGUUACCUG

Identified by deep-sequencing

Stem-loop structure:

C A AA - CC - UU C

GUUAUC AG UA AGG AACU CGG UGUGAUAUG CAA A

CAAUAG UU AU **UCC UUGA GUC** **ACACUAU**AC GUU U

A A C**G** **A A**- **G** C- U
