# Supplementary material for: Deep sequencing and genome-wide analysis reveals the expansion of MicroRNA genes in the gall midge Mayetiola destructor
Source: BMC Genomics. 2013 Mar 18;14:187. doi: 10.1186/1471-2164-14-187 (PMC3608969; doi:10.1186/1471-2164-14-187)
Supplement: Additional file 5: Figure S3 — Abundance of miRNAs affected by host genotypes. miRNA names are given on the top of each graph. N1, M1, N3, and M3 represent one day larvae feeding in Newton (a susceptible cultivar) seedlings, one day larvae in Molly (a resistant cultivar) seedlings, three day larvae in Newton seedlings, and three day larvae in Molly seedlings, respectively. The small letters in each graph indicate different groups based on statistical analysis. [file 1471-2164-14-187-S5.pptx]

## Slide 1
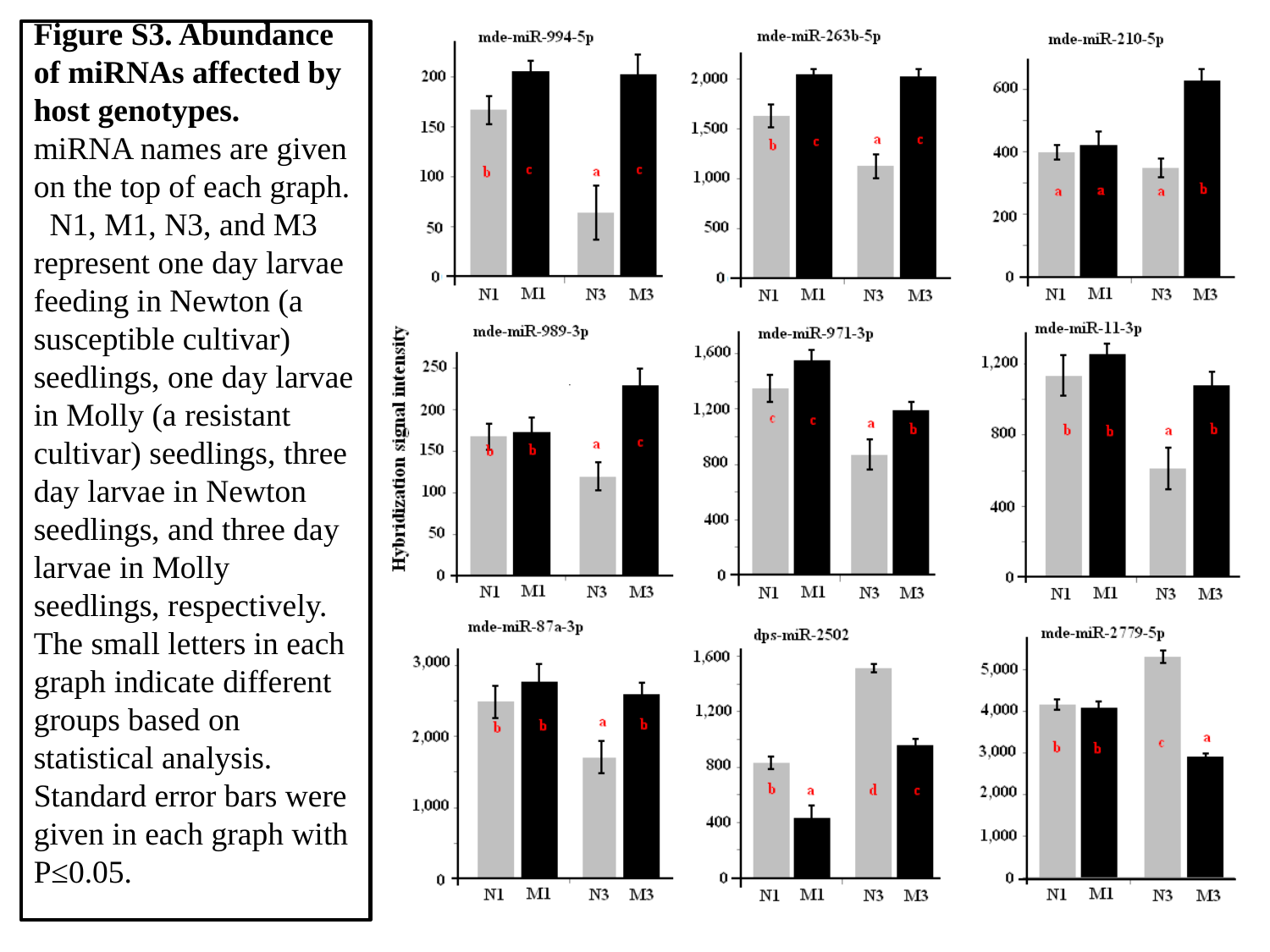

Figure S3. Abundance of miRNAs affected by host genotypes.
miRNA names are given on the top of each graph. N1, M1, N3, and M3 represent one day larvae feeding in Newton (a susceptible cultivar) seedlings, one day larvae in Molly (a resistant cultivar) seedlings, three day larvae in Newton seedlings, and three day larvae in Molly seedlings, respectively. The small letters in each graph indicate different groups based on statistical analysis. Standard error bars were given in each graph with P≤0.05.
