# Supplementary material for: Deep sequencing and genome-wide analysis reveals the expansion of MicroRNA genes in the gall midge Mayetiola destructor
Source: BMC Genomics. 2013 Mar 18;14:187. doi: 10.1186/1471-2164-14-187 (PMC3608969; doi:10.1186/1471-2164-14-187)
Supplement: Additional file 6: Figure S4 — Nucleotide sequence alignments of regions surround miRNA coding regions. The miRNA coding regions and 5’- or 3’-complementary regions are marked with signs “├starts and ┤ends”. [file 1471-2164-14-187-S6.docx]

**Figure S4. Nucleotide sequence alignments of regions surround miRNA coding regions.** The miRNA coding regions and 5’- or 3’-complementary regions are marked signs “├starts, ┤ends”.

**A: The 67443 and 57811 families**

| PC-5p-67443-1 | - | - | - | - | - | - | - | T | A | C | A | T | T | G | C | A | A | C | A | A | T | C | G | - | - | - | - | - | - | - | - | A | A | C | T | G | A | G | A | A | A | A | T | G | T | A | T | C | G | A | T | T | G | C | A | A | A | A | C | G |
| --- | --- | --- | --- | --- | --- | --- | --- | --- | --- | --- | --- | --- | --- | --- | --- | --- | --- | --- | --- | --- | --- | --- | --- | --- | --- | --- | --- | --- | --- | --- | --- | --- | --- | --- | --- | --- | --- | --- | --- | --- | --- | --- | --- | --- | --- | --- | --- | --- | --- | --- | --- | --- | --- | --- | --- | --- | --- | --- | --- | --- |
| PC-5p-67443-12 | - | - | - | - | - | - | - | - | A | A | A | T | T | - | - | T | T | T | A | T | T | C | T | - | - | - | - | - | - | - | - | T | C | C | T | T | A | A | T | A | T | T | A | T | T | G | T | G | T | A | T | T | T | C | T | T | T | G | A | A |
| PC-5p-67443-7 | - | - | - | - | - | - | - | T | T | A | T | G | A | G | C | C | T | T | T | T | C | T | A | - | - | - | - | - | - | - | - | T | C | - | - | T | T | C | A | C | A | C | A | T | C | T | G | G | G | C | A | T | T | A | A | T | T | G | A | A |
| PC-5p-67443-89 | - | - | - | - | - | - | - | T | T | A | T | G | A | G | C | C | T | T | T | T | C | T | A | - | - | - | - | - | - | - | - | T | C | - | - | T | T | C | A | C | A | C | A | T | C | T | G | G | G | C | A | T | T | A | A | T | T | G | A | A |
| PC-5p-67443-23 | - | - | - | - | - | - | - | A | G | A | T | G | T | G | C | A | A | T | A | C | T | T | T | - | - | - | - | - | - | - | - | T | T | G | G | T | T | G | G | C | C | A | G | A | C | G | G | T | T | C | A | C | T | C | A | T | T | - | A | A |
| PC-5p-67443-24 | - | - | C | T | T | G | C | T | T | T | T | G | C | C | G | A | A | T | T | T | C | T | - | - | - | - | - | - | - | - | - | T | C | C | A | A | A | T | T | G | C | C | G | A | A | T | T | G | C | C | C | T | C | C | A | T | A | A | T | C |
| PC-5p-67443-66 | - | - | - | - | T | T | C | G | T | C | T | C | C | A | C | C | G | T | T | G | C | T | G | - | - | - | - | - | - | - | - | T | C | C | T | T | T | T | T | T | A | C | T | T | G | G | T | - | A | C | A | T | T | A | T | T | T | T | A | C |
| PC-5p-67443-69 | - | - | - | - | A | C | T | A | T | A | A | A | A | A | A | T | C | T | C | A | A | T | G | - | - | - | - | - | - | - | - | T | C | A | T | T | A | A | C | A | A | C | T | A | C | G | T | C | T | C | C | T | G | T | A | A | A | A | A | T |
| PC-5p-67443-49 | - | - | - | - | - | - | - | - | T | A | T | T | T | A | C | C | C | C | T | A | T | T | T | - | - | - | - | - | - | - | - | A | T | T | A | T | T | T | T | A | T | T | T | T | G | A | T | A | T | T | T | G | A | G | G | T | A | A | A | A |
| PC-5p-67443-83 | - | - | - | A | T | T | A | C | T | A | T | T | T | T | C | T | C | C | A | A | T | T | G | - | - | - | - | - | - | - | - | A | A | T | A | T | G | C | C | T | G | T | A | A | G | A | T | G | A | A | A | A | A | G | A | C | G | T | A | A |
| PC-5p-67443-53 | - | - | - | - | - | T | G | C | A | A | A | C | T | G | G | C | C | C | A | T | C | G | C | - | - | - | - | - | - | - | - | A | T | T | A | A | G | A | A | A | G | A | T | T | C | A | T | A | G | A | A | C | A | C | G | - | A | T | T | A |
| PC-5p-67443-62 | - | - | - | - | - | - | - | - | A | C | A | C | G | T | A | A | A | A | A | T | T | G | C | - | - | - | - | - | - | - | - | A | A | T | A | A | G | C | T | C | G | A | T | G | C | C | T | G | A | T | G | A | T | G | G | - | C | T | G | A |
| PC-5p-67443-37 | - | - | A | T | C | T | G | A | A | T | G | G | A | T | A | A | A | T | G | C | T | G | A | - | - | - | - | - | - | - | - | G | - | G | T | T | G | A | T | A | C | T | G | G | C | G | T | T | T | - | T | T | A | A | C | T | T | T | G | G |
| PC-5p-67443-40 | A | A | G | A | A | T | T | A | T | T | C | T | T | G | A | A | T | T | T | C | A | G | T | - | - | - | - | - | - | - | - | G | T | G | A | T | G | A | A | G | C | T | C | G | A | A | G | A | G | - | T | G | G | A | A | T | G | C | G | T |
| PC-5p-67443-48 | - | - | - | - | - | - | C | A | A | T | T | T | A | A | T | C | G | G | - | A | G | G | T | - | - | - | - | - | - | - | - | C | A | C | C | T | T | A | T | G | C | A | A | A | C | A | T | T | T | C | T | G | A | G | C | T | C | C | G | T |
| PC-5p-67443-60 | - | - | A | A | A | A | A | A | A | T | G | G | C | C | T | C | G | G | G | T | G | A | T | - | - | - | - | - | - | - | - | T | A | T | C | T | T | A | G | G | C | T | T | T | C | A | A | A | T | - | - | - | - | G | G | T | C | C | A | A |
| PC-5p-67443-26 | - | - | - | - | A | A | G | G | T | T | T | T | T | C | A | T | A | A | A | T | A | T | T | - | - | - | - | - | - | - | - | T | T | A | T | G | C | T | G | C | T | C | T | G | C | T | T | A | T | T | A | T | C | A | T | A | T | G | T | T |
| PC-5p-67443-59 | - | - | - | - | - | - | T | C | C | A | T | T | T | A | A | A | A | A | A | T | A | T | A | - | - | - | - | - | - | - | - | T | T | C | G | A | C | T | - | T | T | C | T | T | C | A | G | A | A | T | - | - | - | - | T | A | T | G | T | T |
| PC-5p-67443-35 | - | - | - | - | - | - | T | T | G | A | T | T | T | A | A | A | A | A | T | T | C | C | T | - | - | - | - | - | - | - | - | T | T | T | T | C | T | T | T | A | A | A | T | A | A | A | T | G | T | C | A | T | T | - | T | T | T | T | C | A |
| PC-5p-67443-44 | - | - | - | - | A | T | T | T | G | A | G | T | T | A | T | T | C | T | T | T | C | T | A | - | - | - | - | - | - | - | - | A | T | T | T | G | T | T | C | T | A | C | T | T | A | T | A | A | T | A | A | C | T | - | T | T | T | T | C | T |
| PC-5p-67443-2 | - | - | - | - | - | - | - | - | C | C | G | A | T | T | G | A | A | A | A | T | T | A | A | - | - | - | - | - | - | - | - | A | - | C | A | T | A | A | T | G | C | C | G | T | C | A | C | A | A | T | A | T | A | T | A | C | T | T | A | C |
| PC-5p-67443-34 | - | - | - | - | - | - | - | - | - | A | A | C | T | T | G | T | C | A | A | T | A | A | T | - | - | - | - | - | - | - | - | C | - | C | G | T | T | T | C | A | C | G | G | - | - | - | - | A | A | T | A | A | A | C | G | C | A | T | A | G |
| PC-5p-67443-5 | - | - | - | - | - | T | C | G | A | T | C | C | G | T | G | A | A | T | A | T | T | C | T | - | - | - | - | - | - | - | - | C | A | T | G | G | A | A | C | G | A | A | G | A | G | A | A | T | C | A | A | A | A | A | T | A | T | T | C | T |
| PC-5p-67443-67 | - | - | - | - | - | - | - | - | - | G | A | A | G | T | G | A | C | A | G | T | T | - | - | - | - | - | - | - | - | - | - | - | - | T | G | A | T | T | C | G | G | A | G | A | A | A | T | A | A | A | A | T | A | T | T | T | A | T | T | T |
| PC-5p-67443-52 | - | - | - | - | - | - | T | C | A | T | A | C | - | A | G | T | G | G | C | C | G | A | T | - | - | - | - | - | - | - | - | C | A | T | A | C | T | A | A | C | A | C | G | C | C | C | G | A | C | T | A | T | T | - | - | T | G | G | T | A |
| PC-5p-67443-63 | - | - | - | - | - | - | - | - | - | - | - | - | - | - | - | - | - | - | - | - | - | - | - | - | - | - | - | - | - | - | - | - | - | - | - | - | - | - | - | - | - | - | - | - | - | - | - | - | - | - | - | - | - | - | - | - | - | - | - | - |
| PC-5p-67443-70 | - | - | - | - | - | - | - | - | - | - | G | A | - | T | G | T | A | T | C | A | T | A | T | - | - | - | - | - | - | - | - | C | A | A | G | C | T | T | T | A | A | A | A | T | C | A | G | T | T | T | T | T | A | G | A | T | G | T | T | T |
| PC-5p-67443-61 | - | - | - | - | - | - | A | C | A | G | T | T | - | C | G | T | A | A | T | A | T | T | A | - | - | - | - | - | - | - | - | T | A | T | T | T | T | A | A | T | C | C | T | T | A | A | A | A | C | C | A | T | T | T | A | T | T | T | T | T |
| PC-5p-67443-82 | - | - | - | - | - | T | T | C | A | C | A | T | G | T | G | T | C | G | C | G | A | T | G | - | - | - | - | - | - | - | - | A | T | T | A | T | T | T | G | T | T | G | A | A | T | T | G | T | C | T | T | A | A | T | T | T | T | T | C | A |
| PC-5p-67443-30 | - | - | A | T | G | A | A | A | T | A | A | T | A | A | A | A | T | G | A | T | C | A | C | - | - | - | - | - | - | - | - | G | A | T | A | C | C | T | T | G | T | C | C | C | T | C | A | A | C | A | G | T | - | A | T | T | G | T | A | A |
| PC-5p-67443-87 | - | - | - | - | G | A | A | C | A | A | A | C | G | A | T | T | C | A | A | G | C | C | C | - | - | - | - | - | - | - | - | T | G | - | - | - | A | A | T | G | G | C | C | G | T | A | G | T | C | A | T | T | - | A | T | A | G | C | C | G |
| PC-5p-67443-77 | - | - | - | - | - | - | G | A | T | G | A | T | G | C | T | A | T | C | G | T | C | G | G | - | - | - | - | - | - | - | - | T | G | - | - | - | A | G | A | T | T | T | T | T | T | G | G | G | C | A | A | T | G | G | A | T | A | C | C | G |
| PC-5p-67443-13 | - | - | - | - | - | - | - | - | A | G | A | T | G | T | T | G | - | - | - | - | A | G | G | - | - | - | - | - | - | - | - | A | C | A | G | T | T | A | T | A | C | C | A | A | C | C | C | A | T | A | A | A | A | A | A | A | A | A | A | T |
| PC-5p-67443-36 | - | - | - | - | - | - | - | - | A | A | A | C | T | T | T | A | T | T | C | C | A | G | A | - | - | - | - | - | - | - | - | A | A | A | G | A | A | T | A | A | C | T | C | A | T | T | C | A | A | A | A | T | G | A | A | G | T | T | T | T |
| PC-5p-67443-27 | - | - | - | - | - | - | A | T | C | A | C | C | C | T | A | A | T | C | A | T | A | G | G | - | - | - | - | - | - | - | - | T | G | A | G | C | A | T | - | A | G | G | G | G | G | G | T | G | T | A | T | C | G | C | C | G | G | T | T | T |
| PC-5p-67443-65 | - | - | - | - | - | C | A | A | C | T | C | T | G | A | A | G | T | A | A | A | G | C | G | - | - | - | - | - | - | - | - | T | G | T | G | T | T | T | G | A | G | G | T | G | C | A | T | T | T | C | T | C | A | A | T | G | A | G | A | A |
| PC-5p-67443-16 | - | - | - | - | C | C | G | A | A | T | A | C | T | G | A | T | G | C | A | C | A | A | T | - | - | - | - | - | - | - | - | T | G | C | G | T | G | T | T | T | T | T | T | C | A | A | A | T | A | A | G | C | C | A | T | G | C | T | A | G |
| PC-5p-67443-19 | - | - | - | A | T | G | G | A | T | T | A | T | G | G | A | T | T | G | A | C | A | A | A | - | - | - | - | - | - | - | - | A | T | A | T | T | G | G | C | A | T | G | T | G | T | A | A | T | G | T | G | A | A | A | A | C | G | T | A | G |
| PC-5p-67443-22 | - | - | - | T | C | T | A | T | G | A | A | T | T | G | T | T | G | G | A | A | T | T | T | - | - | - | - | - | - | - | - | C | A | T | T | C | G | C | T | G | A | T | T | T | C | A | G | A | A | T | A | A | G | C | T | G | T | G | A | G |
| PC-5p-67443-38 | - | - | - | - | - | - | - | C | A | C | T | T | G | A | C | T | G | G | T | T | - | - | - | - | - | - | - | - | - | - | - | C | G | C | A | G | A | T | C | T | A | A | A | G | T | A | A | T | A | C | G | A | A | A | T | A | T | T | G | C |
| PC-5p-67443-39 | - | - | - | - | - | - | A | T | A | A | A | T | G | C | G | T | T | A | T | T | T | T | C | - | - | - | - | - | - | - | - | C | G | C | A | A | T | T | T | C | G | A | A | G | A | A | T | C | C | C | A | T | A | T | T | T | A | T | T | C |
| PC-5p-67443-43 | - | - | - | - | G | C | A | A | A | T | A | A | T | C | A | T | A | G | A | A | T | T | T | - | - | - | - | - | - | - | - | T | G | A | T | C | A | A | A | A | T | T | T | C | G | A | A | A | A | T | A | T | A | A | T | T | T | A | C | A |
| PC-5p-67443-72 | - | - | - | - | - | - | - | - | - | - | T | T | G | C | A | T | T | C | A | A | T | G | T | - | - | - | - | - | - | - | - | G | T | A | T | G | A | T | G | T | C | T | T | C | G | C | A | T | C | T | T | T | G | A | T | T | A | T | C | A |
| PC-5p-67443-73 | - | - | - | - | - | - | - | - | - | - | A | C | A | A | G | A | C | G | T | A | C | T | T | - | - | - | - | - | - | - | - | C | A | G | G | G | T | A | C | T | C | A | A | G | G | T | A | T | A | T | T | T | A | A | A | T | G | A | A | A |
| PC-5p-67443-4 | - | - | - | A | G | T | A | A | C | G | G | A | T | G | A | A | G | G | C | C | T | G | - | - | - | - | - | - | - | - | - | A | T | T | T | G | T | A | T | G | A | A | C | A | T | A | G | T | C | T | T | C | G | T | C | T | G | A | C | G |
| PC-5p-67443-75 | - | A | T | G | T | T | A | A | C | T | G | T | T | C | G | G | C | T | C | A | T | G | T | - | - | - | - | - | - | - | - | G | A | C | A | G | A | A | T | A | A | C | A | A | G | A | A | T | G | C | T | C | G | - | C | G | G | A | A | A |
| PC-5p-67443-10 | - | - | A | T | T | T | G | G | C | A | A | T | T | C | G | C | C | C | T | A | A | T | T | G | G | A | - | - | - | - | - | A | A | T | T | G | G | G | T | A | T | T | C | C | A | A | T | C | G | T | A | C | A | A | C | A | T | T | C | G |
| PC-5p-67443-41 | - | - | - | C | A | T | G | - | - | - | - | - | C | T | A | T | C | C | A | A | C | C | T | - | - | - | - | - | - | - | - | A | T | T | T | T | A | A | T | A | T | T | C | C | A | G | A | A | G | C | A | C | A | T | C | G | - | - | - | A |
| PC-5p-67443-88 | - | T | C | C | A | T | G | G | A | T | G | A | A | T | G | T | C | A | T | T | C | G | T | - | - | - | - | - | - | - | - | A | T | T | G | T | A | A | A | A | T | A | A | A | A | T | T | A | - | C | G | A | A | T | C | G | G | T | T | A |
| PC-5p-67443-54 | - | - | - | - | A | A | T | A | A | A | T | G | T | T | A | T | C | T | A | A | A | T | G | - | - | - | - | - | - | - | - | A | A | T | A | T | C | G | A | T | T | C | A | C | A | T | T | G | A | A | T | C | G | G | G | T | C | A | A | C |
| PC-5p-67443-3 | - | - | - | - | - | - | - | C | T | A | T | C | G | T | T | T | T | C | T | A | T | G | - | - | - | - | - | - | T | T | C | A | T | T | T | C | G | T | A | C | T | C | T | - | T | A | T | G | C | T | A | C | A | T | G | A | T | C | A | T |
| PC-5p-67443-25 | - | - | - | - | - | - | - | A | G | C | A | T | G | C | A | T | G | C | T | T | T | A | - | - | - | - | - | - | T | T | T | G | C | A | T | C | A | A | A | A | G | T | T | G | T | A | T | T | C | A | A | C | G | A | T | A | T | T | G | T |
| PC-5p-67443-80 | - | - | - | - | - | - | - | - | T | G | C | C | T | T | T | C | A | T | T | T | T | A | - | - | - | - | - | - | T | A | G | T | T | T | T | A | C | A | T | G | T | C | T | G | C | G | T | G | T | G | C | C | G | A | A | A | A | C | T | A |
| PC-5p-67443-6 | - | - | - | - | - | - | - | - | - | - | - | T | A | T | A | T | C | A | T | C | G | A | A | - | - | - | - | - | T | A | A | G | T | T | T | T | C | T | C | A | A | G | T | A | A | A | A | T | T | T | C | C | C | A | A | G | T | A | A | G |
| PC-5p-67443-91 | - | - | - | - | - | - | - | - | - | - | - | - | - | - | - | - | - | - | - | - | - | - | - | - | - | - | - | - | - | - | - | - | - | - | - | - | - | - | - | - | - | - | - | - | - | - | - | - | - | - | - | - | - | - | - | - | - | - | - | - |
| PC-5p-67443-17 | - | - | - | - | - | - | - | - | - | G | C | T | G | T | T | T | C | A | C | A | C | A | A | - | - | - | - | - | T | C | C | C | A | G | T | A | G | C | A | A | T | G | T | G | A | A | T | T | T | T | A | T | T | T | A | T | A | A | A | T |
| PC-5p-67443-9 | - | - | - | - | - | - | - | - | - | A | G | T | A | T | T | T | C | T | G | T | T | G | G | - | - | - | - | - | G | A | C | A | A | T | A | C | T | C | T | G | C | A | A | A | G | A | A | A | T | A | A | G | G | G | A | A | A | T | C | G |
| PC-5p-67443-58 | - | - | - | - | - | - | - | - | - | - | - | T | A | T | T | T | C | T | T | A | T | G | G | - | - | - | - | - | A | A | C | A | T | T | T | T | T | G | T | T | C | C | G | T | T | C | A | T | T | T | C | C | T | A | T | G | G | A | A | C |
| PC-5p-67443-28 | - | - | - | - | - | G | A | C | A | C | A | A | T | G | T | T | T | T | T | T | T | C | C | - | - | - | - | - | A | C | A | T | T | T | T | A | A | C | T | G | A | A | T | T | A | C | A | T | - | - | - | C | A | T | A | T | A | A | T | A |
| PC-5p-67443-31 | - | - | - | - | - | - | - | - | - | T | G | T | A | G | A | A | T | A | G | A | G | C | G | - | - | - | - | - | A | C | T | C | T | T | T | T | T | C | G | A | T | A | T | T | C | A | T | T | G | C | A | C | A | T | T | G | A | T | T | A |
| PC-5p-67443-47 | - | - | - | - | - | G | T | A | G | C | C | C | A | A | T | C | A | A | T | T | T | A | C | - | - | - | - | - | G | T | A | A | A | A | T | A | T | C | A | A | A | A | C | A | A | A | A | T | - | - | T | C | A | A | T | A | G | C | T | T |
| PC-5p-67443-71 | - | - | - | - | - | T | T | C | A | T | T | C | G | A | G | C | A | T | T | T | T | G | C | - | - | - | - | - | G | - | - | - | - | - | - | G | T | C | A | A | T | G | G | A | A | A | A | T | - | - | T | C | C | A | A | T | G | A | C | A |
| PC-5p-67443-51 | - | - | - | - | G | A | A | A | G | C | C | G | A | A | T | T | A | T | A | G | C | G | G | - | - | - | - | - | A | T | A | A | A | A | A | A | A | T | G | G | T | T | T | T | A | A | T | - | C | G | G | C | T | A | G | A | G | G | C | A |
| PC-5p-67443-74 | - | - | - | - | - | - | A | A | G | C | A | T | G | T | T | T | T | C | A | G | T | G | A | - | - | - | - | - | T | C | A | A | A | G | T | T | T | T | A | T | T | T | T | A | A | A | T | T | C | A | G | T | T | T | G | A | A | A | T | T |
| PC-5p-67443-21 | - | - | - | - | - | - | G | G | T | T | T | T | G | T | A | T | C | T | A | A | T | A | A | - | - | - | - | - | G | C | A | A | A | T | T | T | T | A | G | - | - | - | T | C | G | A | T | T | A | G | T | G | T | T | A | A | T | C | T | T |
| PC-5p-67443-20 | - | - | - | - | - | - | - | - | A | T | T | A | G | A | A | A | C | T | A | C | G | T | - | - | - | - | - | - | - | - | - | C | T | C | T | T | G | T | T | A | A | A | A | T | T | T | G | A | A | C | A | A | A | A | A | T | A | A | A | A |
| PC-5p-67443-42 | - | - | - | - | - | - | - | - | - | - | T | T | G | T | C | A | C | A | T | C | G | T | G | - | - | - | - | - | T | A | A | C | G | A | C | C | G | A | T | T | T | T | C | T | T | T | G | A | C | T | G | T | C | G | A | A | T | A | A | G |
| PC-5p-67443-90 | - | - | - | - | - | - | - | - | - | A | T | T | T | T | T | G | A | T | T | T | G | A | - | - | - | - | - | - | - | - | - | A | C | A | T | T | G | A | A | T | T | C | T | G | T | T | G | A | A | T | T | T | G | T | A | G | T | A | T | G |
| PC-5p-67443-14 | - | - | - | - | - | - | - | - | - | - | - | - | A | T | T | T | A | T | T | T | T | T | - | - | - | - | - | G | T | G | G | A | A | A | A | A | T | C | A | T | T | C | G | A | C | A | G | T | A | T | T | C | A | A | A | A | T | - | - | T |
| PC-5p-67443-18 | - | - | - | - | - | - | - | - | - | T | A | A | A | A | A | T | T | G | T | A | T | T | - | - | - | - | - | T | T | T | T | C | T | A | T | T | T | C | A | G | T | A | T | A | A | G | T | T | G | T | T | C | G | G | A | T | T | G | A | T |
| PC-5p-67443-15 | - | - | - | - | - | C | A | C | T | G | A | C | A | T | G | A | T | T | T | A | G | T | - | - | - | - | - | A | T | T | A | T | T | A | T | T | A | T | T | A | T | T | T | - | - | - | T | T | C | C | - | - | - | - | A | T | T | T | G | A |
| PC-5p-67443-50 | - | - | - | - | - | - | - | - | - | - | A | T | A | T | T | A | T | T | T | T | G | T | - | - | - | - | - | A | T | T | A | C | A | A | G | C | A | G | C | G | C | C | G | - | - | - | T | A | G | C | T | A | G | A | A | T | T | T | T | T |
| PC-5p-67443-45 | - | - | - | - | - | - | - | - | T | G | T | C | T | C | G | T | T | T | T | C | T | G | - | - | - | - | - | G | A | T | T | C | A | A | T | G | C | T | C | A | T | T | G | - | - | C | T | C | A | C | A | T | A | A | A | A | T | T | G | A |
| PC-5p-67443-57 | - | - | - | - | - | - | - | - | - | G | G | C | A | A | - | T | A | T | A | C | - | - | - | - | - | - | - | G | A | T | A | C | A | T | G | C | C | T | A | A | A | A | A | A | T | A | T | C | G | C | T | A | T | A | A | T | T | T | G | T |
| PC-5p-67443-76 | - | - | - | - | - | - | - | - | T | A | T | C | T | G | T | A | A | T | T | T | G | A | - | - | - | - | - | A | A | T | A | G | A | A | T | G | T | T | A | A | T | T | T | C | G | C | C | T | C | C | A | T | T | C | C | T | A | T | G | C |
| PC-5p-67443-86 | - | - | - | - | - | - | - | - | C | G | A | C | T | G | A | T | C | T | A | T | A | A | - | - | - | - | - | A | T | T | G | T | C | A | T | C | A | T | A | T | T | T | T | A | - | T | T | T | T | A | T | T | T | C | C | A | A | T | G | A |
| PC-5p-67443-55 | - | - | - | - | - | - | - | - | - | - | - | A | C | A | G | A | A | G | A | T | T | G | - | - | - | - | - | - | G | C | T | T | T | A | T | A | A | A | T | C | G | A | G | A | A | A | T | T | C | A | A | T | G | C | A | C | A | A | T | T |
| PC-5p-67443-56 | - | - | - | - | - | A | A | A | A | A | G | A | A | C | A | A | A | A | C | T | T | G | - | - | - | - | - | - | G | C | A | T | C | A | T | G | A | G | G | A | T | T | G | A | T | C | A | T | A | C | A | T | G | - | - | C | T | T | T | T |
| PC-5p-67443-68 | - | - | - | - | - | - | - | C | A | T | G | T | T | C | T | C | G | T | C | T | A | T | - | - | - | - | - | - | T | C | C | C | A | T | T | C | G | T | A | A | T | C | G | A | A | G | T | C | A | C | A | C | A | C | A | T | A | T | T | - |
| PC-5p-67443-79 | - | - | - | - | - | - | - | - | A | T | G | A | G | G | G | A | G | A | C | A | A | A | G | C | A | T | T | C | T | T | C | C | A | A | T | T | T | T | C | G | G | C | A | G | C | A | T | A | A | C | A | T | A | C | G | A | A | G | A | G |
| PC-5p-67443-84 | - | - | - | - | - | A | C | A | A | C | A | C | A | C | T | A | A | A | A | A | A | A | - | - | - | - | - | - | A | A | G | G | A | A | A | A | A | A | A | G | C | A | A | G | G | A | A | A | A | C | A | G | A | C | A | G | A | C | A | G |
| PC-5p-67443-81 | - | - | - | - | - | - | A | C | A | C | A | C | A | C | C | C | A | C | A | C | A | C | - | - | - | - | - | - | - | A | C | A | C | A | C | C | G | A | T | G | A | C | A | A | A | G | A | A | A | C | C | T | C | C | G | A | A | A | G | - |
| PC-5p-67443-8 | - | - | - | - | - | - | - | G | T | T | C | C | T | C | G | A | G | T | T | C | T | - | - | - | - | - | - | - | C | C | T | C | C | T | T | C | T | A | A | T | A | C | A | T | G | T | G | C | G | A | T | T | T | T | T | T | C | C | A | A |
| PC-5p-67443-46 | - | - | - | - | - | - | - | A | G | C | C | C | A | C | T | C | C | T | T | C | G | - | - | - | - | - | - | - | C | C | T | C | - | - | - | - | - | A | A | A | A | A | A | A | A | T | G | G | G | T | T | T | T | A | C | G | C | A | A | A |
| PC-5p-67443-29 | - | - | - | - | - | - | - | - | - | - | G | T | T | C | A | G | A | T | G | A | G | - | - | - | - | - | - | - | A | A | T | G | T | T | T | C | A | G | A | A | A | A | G | A | T | C | A | G | T | C | C | A | T | A | - | T | G | C | A | G |
| PC-5p-67443-78 | - | - | - | - | - | - | - | - | C | A | T | T | T | G | G | A | A | T | T | A | A | - | - | - | - | - | - | - | A | A | T | A | T | A | T | G | C | G | T | T | C | C | A | G | A | C | T | T | T | A | T | T | T | C | G | T | A | T | A | T |
| PC-5p-67443-11 | - | - | - | - | - | - | A | C | A | A | A | A | T | G | A | A | A | T | A | T | C | A | G | - | - | - | - | - | - | T | T | C | A | A | A | A | T | A | T | A | A | C | C | A | A | T | C | T | C | T | G | G | G | T | G | A | T | G | T | C |
| PC-5p-67443-32 | - | - | - | - | - | - | - | C | G | A | T | T | T | G | A | A | T | T | - | - | - | - | - | - | - | - | - | - | - | T | T | T | T | A | A | A | T | A | A | T | T | A | C | A | A | G | T | A | C | A | A | G | G | A | A | A | A | A | T | A |
| PC-5p-67443-33 | - | - | - | - | - | - | A | A | C | C | A | T | A | G | C | T | C | T | A | T | T | T | - | - | - | - | - | - | - | T | G | A | A | A | A | C | G | G | A | G | C | C | C | C | C | G | C | T | T | T | T | T | T | C | A | A | A | A | T | A |
| PC-5p-67443-64 | - | - | - | - | - | - | - | - | - | - | - | - | - | A | A | A | A | G | A | A | T | G | - | - | - | - | - | - | - | A | T | G | A | G | T | A | A | A | C | A | A | A | A | A | T | T | G | A | T | T | T | G | T | A | G | C | C | T | A | G |
| PC-5p-67443-85 | - | - | - | - | - | - | - | - | T | T | T | T | G | C | T | T | T | G | A | T | T | G | - | - | - | - | - | - | - | A | T | T | T | G | T | A | A | T | T | G | A | T | G | T | A | T | A | A | T | - | - | G | T | T | C | A | T | T | A | G |
|  |  |  |  |  |  |  |  |  |  |  |  |  |  |  |  |  |  |  |  |  |  |  |  |  |  |  |  |  |  |  |  |  |  |  |  |  |  |  |  |  |  |  |  |  |  |  |  |  |  |  |  |  |  |  |  |  |  |  |  |  |
| PC-5p-67443-1 | T | T | A | A | A | T | G | G | T | T | G | A | A | T | G | G | T | C | G | A | A | T | T | A | T | G | G | G | A | A | A | T | A | G | G | T | A | A | A | A | T | C | A | A | G | A | G | A | G | - | A | A | A | A | T | A | C | G | T | - |
| PC-5p-67443-12 | A | T | T | G | A | C | T | A | T | T | A | T | T | G | T | G | C | T | A | A | A | A | T | A | T | - | - | - | A | T | A | T | A | T | G | T | A | A | A | A | A | C | G | T | G | T | A | T | G | G | A | A | A | A | T | G | C | A | T | T |
| PC-5p-67443-7 | T | T | A | T | G | T | G | A | G | A | A | A | T | A | A | T | G | A | G | A | A | A | A | T | A | A | G | C | T | G | C | C | C | A | A | T | C | T | G | G | A | A | C | T | G | T | A | C | A | C | T | G | T | T | T | T | T | A | T | T |
| PC-5p-67443-89 | T | T | A | T | G | T | G | A | G | A | A | A | T | A | A | T | G | A | G | A | A | A | A | T | A | A | G | C | T | G | C | C | C | A | A | T | C | T | G | G | A | A | C | T | G | T | A | C | A | C | T | G | T | T | T | T | T | A | T | T |
| PC-5p-67443-23 | T | G | C | A | G | A | C | A | A | G | T | C | A | A | A | A | A | A | A | T | A | A | A | T | A | A | A | C | T | T | C | A | A | T | A | A | C | T | C | C | A | T | T | T | T | A | A | T | A | T | T | G | G | - | A | A | A | A | T | G |
| PC-5p-67443-24 | A | T | T | T | A | T | A | C | A | A | T | A | G | T | A | C | A | T | - | - | - | A | T | T | G | T | T | G | A | T | A | A | T | A | A | T | T | G | C | A | A | T | A | T | A | A | T | T | T | T | C | G | A | C | A | G | C | A | G | - |
| PC-5p-67443-66 | T | T | A | A | A | A | A | A | G | A | T | T | T | T | C | G | A | T | - | - | - | G | T | T | T | T | C | G | C | G | A | T | T | T | A | A | A | C | A | A | A | T | A | T | C | A | G | C | T | A | A | G | T | C | A | T | C | A | T | A |
| PC-5p-67443-69 | T | T | G | A | A | C | G | A | A | A | T | C | A | T | A | G | A | T | T | C | G | G | T | T | T | T | C | G | A | A | A | T | A | T | T | T | A | G | C | C | T | T | G | - | A | T | T | C | A | C | T | T | T | C | T | C | C | A | T | - |
| PC-5p-67443-49 | C | T | A | A | T | A | T | C | C | A | A | T | A | T | T | A | A | T | T | G | T | T | C | T | C | A | T | A | T | A | T | C | A | A | A | A | T | A | T | C | T | C | G | A | A | A | C | C | A | A | A | A | A | A | A | A | T | A | T | T |
| PC-5p-67443-83 | A | A | A | A | A | T | T | C | C | G | T | A | G | A | A | A | A | T | A | T | T | A | A | T | G | T | T | C | A | A | T | C | A | C | G | - | T | G | A | A | T | C | G | T | A | C | G | A | G | T | G | A | T | A | T | A | C | A | T | A |
| PC-5p-67443-53 | A | A | T | - | C | G | C | C | A | A | A | A | A | A | A | A | A | T | T | G | T | G | C | T | A | G | T | G | G | A | T | A | A | A | A | T | T | C | G | A | A | C | G | A | A | A | T | C | A | A | T | T | T | G | A | A | G | A | A | G |
| PC-5p-67443-62 | T | G | T | G | T | G | T | T | G | A | G | C | T | T | G | G | G | G | C | G | T | T | T | T | A | A | C | G | A | A | A | A | C | G | T | A | A | C | C | A | G | T | G | A | A | A | A | C | A | T | A | A | T | G | A | T | A | A | T | A |
| PC-5p-67443-37 | A | G | T | A | C | A | C | A | A | A | G | T | G | C | A | A | A | A | T | T | T | T | A | A | T | A | T | A | T | T | G | G | - | - | C | C | A | A | A | C | A | T | T | T | A | T | T | C | A | A | A | A | T | A | A | A | C | T | G | T |
| PC-5p-67443-40 | G | A | T | C | C | A | G | A | C | A | G | T | A | T | A | T | T | A | A | A | T | C | G | A | T | C | T | C | T | T | G | G | T | A | A | C | A | G | G | C | A | A | A | T | G | T | T | T | T | A | T | A | T | G | T | A | C | - | - | - |
| PC-5p-67443-48 | T | G | A | T | T | A | T | T | T | T | G | T | A | T | G | A | A | C | T | A | C | C | C | T | A | T | G | C | A | A | G | G | - | G | A | T | A | A | G | A | T | G | A | G | G | A | A | T | A | G | T | G | A | A | T | T | A | A | A | T |
| PC-5p-67443-60 | A | T | T | A | C | A | G | G | G | C | A | T | A | A | G | A | C | C | C | A | T | C | C | C | A | C | A | C | C | G | G | G | - | T | T | A | G | A | G | C | T | G | G | T | A | C | A | T | A | - | T | A | T | A | T | A | C | A | T | T |
| PC-5p-67443-26 | T | T | G | A | C | T | C | T | G | T | T | T | T | A | A | C | T | - | T | C | T | C | T | A | G | A | A | T | G | C | A | G | T | - | - | - | C | G | A | T | T | T | T | C | G | G | C | T | G | C | T | G | G | G | T | T | C | C | T | C |
| PC-5p-67443-59 | T | C | G | A | A | T | A | A | G | G | A | A | A | A | A | T | T | G | A | C | A | C | A | C | G | G | A | T | C | C | A | C | T | - | - | - | C | A | A | A | A | A | A | A | A | A | G | A | A | T | T | A | T | T | T | T | C | A | T | T |
| PC-5p-67443-35 | G | C | T | G | T | A | A | C | A | T | T | C | T | A | C | A | G | T | G | C | C | A | T | C | - | - | A | T | A | T | A | G | T | G | C | T | A | A | C | T | T | T | G | T | A | A | A | C | A | A | A | G | G | C | A | T | C | G | T | T |
| PC-5p-67443-44 | - | C | A | T | T | T | G | T | G | A | A | A | T | A | A | T | T | A | G | C | C | A | T | A | G | A | A | A | A | A | A | G | T | G | G | T | C | T | A | T | T | T | G | G | A | G | A | T | A | C | T | G | - | - | - | T | A | C | A | T |
| PC-5p-67443-2 | T | T | A | T | A | A | C | T | C | A | C | T | - | - | - | T | A | G | C | T | C | C | T | A | G | C | T | A | C | T | A | G | - | - | - | - | - | - | A | C | G | T | A | T | A | T | A | T | A | T | A | A | A | T | A | T | A | A | T | A |
| PC-5p-67443-34 | A | A | T | T | T | A | A | T | C | C | G | A | - | - | - | T | G | A | C | T | T | T | T | T | T | G | T | G | T | G | T | G | - | - | - | - | - | - | A | G | G | T | A | T | G | A | G | T | A | A | T | G | C | A | A | T | A | A | T | A |
| PC-5p-67443-5 | C | A | T | T | T | G | A | T | C | C | G | G | - | - | - | - | - | G | A | A | T | A | A | T | T | - | - | - | - | G | A | G | - | - | - | - | - | - | A | A | G | T | T | T | T | T | A | A | G | G | A | A | C | G | A | A | A | A | A | A |
| PC-5p-67443-67 | A | T | T | T | T | G | A | T | C | C | G | G | C | A | A | T | C | G | A | A | T | A | A | C | A | T | G | A | A | G | C | G | - | - | - | - | - | - | C | A | G | T | A | T | G | C | T | C | A | T | T | A | A | G | T | G | T | G | G | T |
| PC-5p-67443-52 | G | - | - | C | A | G | A | A | C | G | T | A | G | - | - | - | T | T | T | A | C | C | G | C | A | G | C | G | T | C | T | A | - | - | - | - | - | - | C | G | T | T | C | A | G | T | T | G | T | G | T | T | G | A | A | A | A | A | A | G |
| PC-5p-67443-63 | - | - | - | - | - | - | - | - | - | - | - | - | - | - | - | - | - | - | - | - | - | - | - | - | - | - | - | - | - | - | - | - | - | - | - | - | - | - | - | - | - | - | - | - | - | - | - | - | - | - | - | - | - | - | - | - | - | - | - | - |
| PC-5p-67443-70 | G | T | A | C | C | A | A | T | C | A | T | A | A | - | - | - | C | T | C | A | T | C | G | C | G | G | T | A | C | A | C | A | - | - | - | - | - | - | C | C | T | A | G | A | G | T | C | T | T | G | A | A | A | T | T | T | T | A | A | C |
| PC-5p-67443-61 | A | T | - | C | T | G | A | T | C | G | T | T | - | - | - | - | - | T | C | C | T | C | T | T | T | T | T | T | G | T | T | T | - | - | - | - | - | - | A | A | T | T | C | T | G | A | A | A | T | - | T | C | G | T | T | T | T | T | T | G |
| PC-5p-67443-82 | T | T | T | C | T | G | C | T | T | G | A | T | G | - | - | - | T | G | A | A | A | C | A | T | T | T | T | G | C | T | T | G | - | - | - | - | - | - | A | T | G | T | G | A | A | A | C | A | T | T | A | A | G | T | T | A | T | A | A | A |
| PC-5p-67443-30 | T | T | A | T | T | T | T | C | T | A | T | T | C | G | - | - | - | C | C | A | C | C | A | T | T | C | G | A | T | T | - | - | - | - | - | - | - | - | T | T | T | A | T | C | G | A | T | T | T | T | C | A | C | T | G | C | C | T | T | T |
| PC-5p-67443-87 | T | C | T | A | T | T | C | C | T | T | T | T | T | G | T | T | T | T | C | T | T | C | A | A | T | C | A | A | T | T | G | - | - | - | - | - | - | - | C | A | T | T | T | C | G | A | C | C | G | T | T | A | C | G | A | T | T | A | T | A |
| PC-5p-67443-77 | A | C | A | C | T | G | T | C | T | T | G | A | A | T | - | - | T | C | A | T | T | C | A | T | C | C | A | A | T | T | C | C | - | - | - | - | - | - | C | A | T | T | A | C | G | G | G | C | T | T | C | G | A | A | T | A | T | G | T | A |
| PC-5p-67443-13 | A | A | T | T | T | C | C | C | T | A | C | G | A | T | T | T | G | G | - | - | G | C | G | G | G | C | T | T | T | T | T | A | - | - | - | - | - | - | A | A | T | T | C | A | A | A | A | G | T | C | C | C | C | T | T | A | C | T | T | T |
| PC-5p-67443-36 | T | G | T | T | A | G | C | T | T | T | C | C | T | T | T | T | T | G | T | A | A | C | T | T | C | C | A | T | A | G | C | T | - | - | - | - | - | - | A | T | T | T | T | G | G | T | G | G | T | C | A | G | G | T | A | A | C | T | T | G |
| PC-5p-67443-27 | A | C | C | C | C | C | C | C | C | C | C | C | T | A | A | A | G | C | C | T | G | T | G | G | C | C | A | G | A | C | C | C | - | - | - | - | - | - | C | C | C | C | C | C | C | C | C | C | A | T | T | A | T | A | T | A | T | T | C | - |
| PC-5p-67443-65 | A | A | T | G | C | A | T | T | C | A | T | C | G | A | T | A | G | A | A | G | T | T | G | A | G | C | A | G | A | G | G | C | - | - | - | - | - | - | A | A | C | A | T | T | A | A | A | T | G | A | G | A | A | T | T | A | T | A | A | G |
| PC-5p-67443-16 | - | A | A | T | G | A | T | A | A | G | T | G | A | T | C | T | C | T | T | C | A | A | A | T | C | G | A | A | T | T | T | T | T | - | - | - | A | C | G | A | A | A | A | A | A | T | G | A | A | A | A | A | A | A | T | G | C | A | C | A |
| PC-5p-67443-19 | G | C | T | T | G | A | A | T | T | C | C | A | A | A | T | T | A | T | T | T | G | A | A | T | T | A | A | A | A | T | T | G | - | - | - | - | - | C | T | G | A | T | T | A | T | T | - | T | T | T | T | T | T | G | T | A | C | T | C | A |
| PC-5p-67443-22 | T | T | C | T | G | T | G | A | T | C | A | A | A | A | C | T | A | T | T | T | C | T | G | A | C | A | A | A | T | A | C | A | - | - | - | - | - | - | T | A | T | A | A | T | C | T | G | C | T | C | T | G | T | A | T | T | T | A | A | T |
| PC-5p-67443-38 | T | C | G | A | T | T | G | A | A | C | C | A | T | G | T | C | A | C | T | T | T | A | T | G | A | A | A | G | T | C | A | A | T | T | A | T | T | G | T | A | A | G | G | T | T | T | T | T | T | T | T | T | T | C | A | T | T | A | T | A |
| PC-5p-67443-39 | T | C | - | A | G | C | G | A | T | T | C | A | T | A | T | A | T | G | T | A | T | A | T | A | T | A | T | A | T | C | G | - | - | - | - | - | T | A | T | A | A | G | A | T | G | T | C | A | T | G | T | G | C | A | A | A | C | C | A | A |
| PC-5p-67443-43 | T | A | C | A | A | A | C | C | T | G | A | A | T | A | A | A | A | T | T | C | G | T | A | T | G | T | T | A | A | A | T | G | - | - | - | - | T | C | C | G | T | T | G | G | A | T | C | A | A | A | T | T | A | T | C | A | T | C | A | T |
| PC-5p-67443-72 | T | T | C | G | T | A | G | A | A | A | A | T | T | G | A | A | C | G | T | C | A | C | A | A | A | A | C | A | G | C | A | A | - | - | - | - | A | A | C | A | C | A | T | A | T | T | T | T | A | A | T | G | T | A | C | A | A | A | A | T |
| PC-5p-67443-73 | T | A | A | G | A | A | T | A | C | A | T | G | C | G | A | A | A | G | T | A | C | A | A | A | T | A | A | A | A | A | T | T | - | - | - | - | A | A | A | A | A | A | A | T | A | T | C | A | A | T | T | A | T | T | A | A | C | A | A | T |
| PC-5p-67443-4 | A | T | T | - | - | - | - | C | C | G | T | T | T | G | - | - | - | - | A | G | C | A | C | A | C | A | C | A | C | A | C | - | A | C | A | A | T | T | C | A | A | C | T | G | G | A | C | A | A | A | T | T | G | A | T | C | T | G | A | A |
| PC-5p-67443-75 | A | T | T | - | - | - | - | C | A | A | G | T | T | G | G | A | A | T | T | G | A | T | A | A | A | T | C | A | G | G | C | - | A | T | G | A | A | T | G | T | T | T | C | G | G | A | T | C | A | A | A | T | G | T | T | - | T | G | - | - |
| PC-5p-67443-10 | A | T | T | G | A | T | - | C | T | A | A | A | T | A | T | T | - | A | A | G | T | C | A | A | C | T | C | T | G | A | C | G | A | T | G | A | A | T | T | T | A | T | C | C | G | T | T | C | A | A | T | A | G | T | A | A | A | A | A | - |
| PC-5p-67443-41 | A | T | A | G | A | A | T | T | T | C | A | T | T | G | A | G | G | G | T | G | T | C | A | C | T | T | G | A | C | G | C | G | G | T | C | A | T | T | G | T | G | T | C | C | G | A | A | T | T | T | C | G | A | A | T | A | T | T | C | T |
| PC-5p-67443-88 | A | T | T | T | A | A | T | T | C | C | A | T | T | T | T | T | T | G | C | A | T | A | A | T | T | G | A | T | A | A | T | T | G | A | T | T | T | C | C | A | T | T | T | C | G | C | A | A | T | T | T | - | A | G | T | T | T | T | T | T |
| PC-5p-67443-54 | G | T | T | T | T | A | A | A | T | A | T | T | C | A | T | A | A | T | A | A | T | A | A | A | A | T | A | A | A | G | T | G | - | - | - | A | T | G | G | A | A | T | G | A | T | T | C | A | T | T | T | T | A | C | C | A | A | A | A | G |
| PC-5p-67443-3 | T | C | A | A | T | A | T | A | T | C | C | G | G | A | A | A | A | C | T | T | G | - | - | - | - | T | A | T | A | T | T | T | T | G | G | C | T | G | T | A | T | G | C | G | G | G | C | G | T | G | C | C | T | T | C | A | A | C | A | A |
| PC-5p-67443-25 | T | G | A | A | T | A | A | C | A | G | A | T | G | A | G | A | A | C | A | A | T | G | A | C | A | A | A | A | A | G | T | T | T | G | C | C | T | A | T | T | T | - | - | T | G | A | A | A | T | G | - | - | - | T | T | A | A | C | A | C |
| PC-5p-67443-80 | C | T | A | T | T | G | A | G | T | G | T | A | T | T | A | C | A | C | A | T | T | T | G | - | C | T | T | T | A | A | A | A | A | G | C | G | A | A | T | G | T | A | T | C | T | C | A | T | T | G | - | - | - | - | - | A | A | C | G | T |
| PC-5p-67443-6 | A | G | C | T | T | G | C | T | T | G | T | C | A | T | T | C | A | A | G | C | G | G | A | C | G | A | A | - | - | - | T | C | T | A | C | A | G | A | T | T | C | A | T | A | T | A | T | A | T | A | T | A | T | A | T | A | A | T | G | G |
| PC-5p-67443-91 | - | - | - | - | - | - | - | - | - | - | - | - | - | - | - | - | - | - | - | - | - | - | - | - | - | - | - | - | - | - | - | - | - | - | - | - | - | - | - | - | - | - | - | - | - | - | - | - | - | - | - | - | - | - | - | - | - | - | - | - |
| PC-5p-67443-17 | C | T | T | A | T | G | A | A | T | A | A | T | G | A | C | A | A | T | G | T | T | C | G | C | G | G | A | - | - | - | T | G | G | C | C | A | G | - | - | - | - | - | - | - | - | - | - | - | - | T | A | A | G | A | T | A | A | T | T | G |
| PC-5p-67443-9 | T | T | T | T | C | A | A | C | T | T | T | A | A | A | T | A | T | T | A | T | T | T | G | T | T | T | T | - | - | - | T | G | G | T | T | A | T | C | A | C | C | A | A | T | T | - | - | G | G | T | C | A | C | A | T | T | G | T | A | A |
| PC-5p-67443-58 | A | T | T | T | T | G | G | T | T | T | T | G | C | G | C | A | A | C | T | C | T | T | G | T | A | T | G | - | - | - | T | C | A | A | T | C | T | G | A | A | A | G | G | T | T | C | C | A | T | T | T | T | C | A | T | T | C | T | G | A |
| PC-5p-67443-28 | T | T | T | T | G | A | C | G | A | T | G | A | C | G | G | C | C | A | T | A | T | C | A | T | C | G | - | - | - | - | - | - | C | C | A | A | A | T | T | G | G | T | A | T | T | C | A | A | A | C | C | A | T | T | T | T | T | T | T | A |
| PC-5p-67443-31 | T | G | G | T | G | T | A | A | T | G | A | T | C | A | G | C | T | T | A | G | T | C | G | C | C | T | T | - | - | - | - | - | T | C | A | A | G | T | A | G | T | T | G | A | T | C | G | A | G | A | T | T | C | G | T | T | T | C | T | C |
| PC-5p-67443-47 | T | A | C | T | A | G | A | G | A | A | A | A | C | G | A | A | T | T | G | C | A | A | A | A | - | - | - | - | - | - | A | T | G | G | T | A | A | A | T | T | A | A | G | A | T | G | G | A | G | A | A | T | C | G | A | A | T | A | A | A |
| PC-5p-67443-71 | T | T | T | T | T | C | C | A | T | C | A | A | C | C | A | A | T | C | G | C | A | C | A | A | G | G | T | C | C | A | A | T | C | A | A | T | A | A | T | A | A | T | G | A | T | A | A | A | A | A | T | A | A | T | A | A | T | A | A | T |
| PC-5p-67443-51 | A | G | A | A | G | C | C | G | T | A | A | A | G | A | G | A | A | A | A | T | C | C | A | G | T | C | G | - | - | - | C | C | G | A | T | T | - | - | G | A | C | A | A | - | - | - | T | A | C | T | C | A | C | G | - | A | T | A | T | A |
| PC-5p-67443-74 | G | G | A | A | T | T | C | A | T | T | A | T | - | - | G | A | A | A | A | C | C | A | A | G | G | A | A | - | - | - | A | A | T | A | T | T | - | - | C | A | A | T | A | G | A | T | T | A | C | A | C | T | T | G | - | A | T | T | A | G |
| PC-5p-67443-21 | T | A | T | T | T | G | C | G | T | A | C | G | - | - | G | A | A | A | A | G | T | T | T | G | T | T | G | - | - | - | T | C | G | C | A | T | G | C | G | A | T | T | A | C | C | T | T | G | T | T | T | A | C | G | T | A | T | C | A | A |
| PC-5p-67443-20 | T | C | G | G | A | A | C | G | T | G | A | A | A | - | - | A | C | T | A | A | A | A | A | A | C | G | G | A | A | C | C | C | A | A | A | A | T | C | T | A | G | A | A | T | C | T | G | T | T | C | C | C | A | A | A | A | A | A | C | A |
| PC-5p-67443-42 | T | T | G | A | C | A | C | A | T | C | A | C | A | - | - | A | T | T | C | A | A | A | T | G | G | A | T | T | T | C | A | T | T | T | C | A | T | T | T | C | G | T | T | T | C | - | G | A | T | G | G | A | A | A | A | C | A | A | C | A |
| PC-5p-67443-90 | A | T | G | G | T | T | C | A | T | G | A | A | T | G | G | A | C | A | C | T | C | A | A | A | G | G | T | T | G | A | A | T | T | T | C | A | A | T | C | A | A | T | A | T | C | - | A | C | T | G | G | C | A | C | T | T | A | T | T | G |
| PC-5p-67443-14 | G | A | A | T | T | G | C | A | T | T | G | A | A | C | T | G | A | T | G | A | A | C | A | A | A | T | A | T | A | G | C | A | T | T | A | G | A | A | T | T | G | C | A | T | A | G | A | A | T | A | T | T | C | C | C | A | G | G | C | C |
| PC-5p-67443-18 | G | A | A | C | A | A | C | A | T | T | G | A | C | C | T | - | - | T | G | A | A | A | A | G | T | C | A | A | G | G | A | A | A | C | G | A | A | A | C | G | T | C | A | A | T | G | A | A | G | G | T | A | C | A | C | G | - | - | - | T |
| PC-5p-67443-15 | T | T | T | G | G | A | A | A | G | C | T | A | T | T | T | A | A | A | T | G | C | T | A | A | T | T | A | T | T | T | A | T | T | A | A | T | A | G | C | A | G | - | - | T | G | G | A | A | G | A | T | G | T | A | T | G | T | A | C | A |
| PC-5p-67443-50 | T | T | T | G | A | G | G | G | G | C | T | G | C | T | C | A | G | C | A | C | C | G | A | A | A | G | A | A | A | A | A | T | A | A | T | A | A | A | C | T | G | T | T | T | G | A | A | A | A | A | G | T | T | G | C | C | T | T | A | A |
| PC-5p-67443-45 | A | A | C | A | A | A | T | T | C | T | T | A | A | T | T | G | A | T | A | A | T | A | A | T | C | T | A | G | T | A | A | A | T | A | T | T | A | A | A | T | A | A | G | C | G | T | T | G | - | T | T | T | T | G | T | A | C | A | T | T |
| PC-5p-67443-57 | A | T | C | G | G | A | T | T | A | T | T | G | T | T | T | C | G | C | A | C | A | A | A | A | G | T | A | A | C | G | A | A | C | A | C | G | - | A | T | T | G | C | C | C | C | A | C | A | - | A | T | T | G | G | A | A | T | A | C | T |
| PC-5p-67443-76 | T | A | A | T | G | A | T | A | A | C | A | A | T | A | A | T | A | A | T | G | C | G | A | C | A | T | T | T | C | C | G | A | T | C | G | T | C | A | T | G | C | C | - | - | - | - | A | A | A | G | T | T | C | G | T | C | A | A | A | T |
| PC-5p-67443-86 | T | A | A | A | T | A | T | A | T | T | C | A | T | A | A | T | A | A | T | - | - | - | - | C | A | T | T | T | T | A | - | A | T | T | G | A | A | A | T | G | A | T | - | - | - | - | A | A | C | G | C | C | C | T | G | C | A | A | A | A |
| PC-5p-67443-55 | A | A | C | A | T | G | T | A | T | C | A | A | T | A | A | C | G | T | A | T | T | A | T | G | G | T | T | C | C | C | A | G | T | G | A | A | A | A | T | G | T | A | - | - | A | A | T | A | A | A | A | T | C | C | A | T | A | T | T | G |
| PC-5p-67443-56 | A | C | C | A | A | G | T | T | T | G | C | A | A | G | A | T | G | A | A | A | G | T | T | G | G | - | - | - | C | C | A | T | A | A | A | A | A | G | A | A | A | A | - | - | A | A | G | C | G | T | G | T | C | A | A | T | A | T | C | - |
| PC-5p-67443-68 | - | A | T | T | G | T | G | T | A | T | T | A | T | G | T | T | G | G | T | G | A | T | T | C | A | A | A | - | - | A | A | T | T | T | G | A | T | T | C | A | T | T | - | - | T | A | T | C | G | A | A | A | T | G | C | C | G | G | T | A |
| PC-5p-67443-79 | C | T | C | T | G | T | G | T | A | T | T | T | A | G | C | T | G | G | T | G | G | C | T | G | A | A | T | G | G | A | A | A | A | G | G | A | A | T | C | T | T | T | - | - | A | A | T | C | A | A | A | G | T | - | - | - | - | - | - | - |
| PC-5p-67443-84 | T | T | G | T | T | T | G | C | A | G | G | C | A | G | C | T | T | A | T | T | T | C | T | T | A | T | T | A | T | T | A | T | T | G | A | C | A | T | T | T | T | C | - | - | A | A | A | T | A | G | A | A | C | A | T | A | C | T | A | C |
| PC-5p-67443-81 | C | G | A | T | C | A | A | T | A | A | T | A | A | C | T | G | T | C | T | T | T | C | T | T | A | C | A | C | A | C | T | T | A | A | A | C | A | T | C | G | A | C | - | - | T | G | A | A | A | T | A | T | C | A | A | T | G | - | - | - |
| PC-5p-67443-8 | A | A | C | A | G | T | C | A | T | T | C | C | T | C | T | A | T | T | T | T | C | A | - | - | - | A | C | G | A | C | A | A | C | A | A | A | T | C | A | T | C | G | T | A | C | C | A | T | A | A | T | T | C | A | A | T | C | G | T | C |
| PC-5p-67443-46 | C | T | A | A | C | G | C | G | T | T | C | G | T | G | G | G | G | A | C | G | C | C | G | T | T | T | C | T | G | A | T | A | C | G | A | G | T | A | T | T | C | G | T | A | T | C | C | A | G | A | T | T | C | A | A | A | T | - | A | C |
| PC-5p-67443-29 | C | T | C | G | A | T | T | T | A | T | T | G | A | A | T | A | C | T | T | C | C | G | T | C | G | T | G | C | T | C | A | A | T | C | A | C | A | A | A | T | T | A | A | T | T | C | T | A | T | T | T | G | A | T | T | G | C | - | A | T |
| PC-5p-67443-78 | T | T | C | A | T | T | C | G | T | T | T | T | G | A | A | A | A | T | G | A | A | A | T | A | A | T | G | T | G | T | A | T | A | T | G | C | T | - | - | T | T | A | T | T | T | C | G | T | T | T | T | T | C | A | T | A | T | - | A | T |
| PC-5p-67443-11 | A | A | T | A | G | T | C | C | C | A | G | G | A | A | A | T | T | G | A | A | T | G | A | A | T | T | T | - | - | T | T | T | C | C | A | G | A | A | A | T | G | A | T | A | T | A | A | A | A | T | - | - | - | - | - | - | A | T | A | T |
| PC-5p-67443-32 | C | A | C | A | T | T | T | C | A | T | T | G | A | A | T | T | G | G | A | T | A | C | A | T | T | T | A | - | - | T | T | G | C | T | T | G | A | T | G | T | G | A | A | A | C | A | T | T | A | A | G | T | T | A | T | A | A | A | A | T |
| PC-5p-67443-33 | G | G | T | T | T | T | T | T | T | T | G | A | A | G | A | T | A | T | T | C | A | G | A | A | T | T | T | G | G | T | T | C | A | C | T | A | A | A | G | G | C | A | T | T | C | A | A | A | A | T | - | - | - | - | - | - | - | A | A | A |
| PC-5p-67443-64 | G | G | T | T | T | C | C | A | C | A | A | A | A | A | T | T | T | C | G | T | T | C | T | A | T | G | A | T | C | A | A | A | A | A | A | C | C | A | T | T | T | T | T | A | G | A | T | T | C | T | T | T | G | G | G | A | C | A | T | T |
| PC-5p-67443-85 | G | T | T | T | T | T | C | A | C | A | A | A | A | A | T | T | C | C | G | A | T | C | T | A | T | G | A | T | C | A | G | A | C | G | - | - | C | A | T | T | T | T | T | A | G | A | T | T | C | A | T | T | G | G | G | A | A | A | T | T |

**57811 starts**├ ├**67443 starts**

| PC-5p-67443-1 | - | - | - | - | - | - | - | - | - | - | - | - | - | - | - | - | A | A | T | A | C | A | A | T | T | G | A | A | T | C | G | C | T | G | A | G | A | - | - | - | - | - | A | T | A | A | A | T | A | T | G | G | G | A | C | G | T | C | T | C |
| --- | --- | --- | --- | --- | --- | --- | --- | --- | --- | --- | --- | --- | --- | --- | --- | --- | --- | --- | --- | --- | --- | --- | --- | --- | --- | --- | --- | --- | --- | --- | --- | --- | --- | --- | --- | --- | --- | --- | --- | --- | --- | --- | --- | --- | --- | --- | --- | --- | --- | --- | --- | --- | --- | --- | --- | --- | --- | --- | --- | --- |
| PC-5p-67443-12 | T | G | G | A | - | - | - | - | - | - | - | - | - | - | - | - | A | C | T | A | C | A | A | T | T | G | A | A | T | C | A | C | T | G | A | G | A | - | - | - | - | - | A | T | A | A | A | T | A | T | G | G | G | A | C | T | T | C | T | C |
| PC-5p-67443-7 | - | - | - | - | - | - | - | - | - | - | - | - | - | - | - | - | T | G | T | A | C | A | A | T | T | G | A | A | T | C | G | C | T | G | A | G | A | - | - | - | - | - | A | T | A | A | A | T | A | T | G | G | G | A | C | T | T | C | T | C |
| PC-5p-67443-89 | - | - | - | - | - | - | - | - | - | - | - | - | - | - | - | - | T | G | T | A | C | A | A | T | T | G | A | A | T | C | G | C | T | G | A | G | A | - | - | - | - | - | A | T | A | A | A | T | A | T | G | G | G | A | C | T | T | C | T | C |
| PC-5p-67443-23 | - | - | - | - | - | - | - | - | - | - | - | - | - | - | - | - | A | A | T | A | A | A | A | T | T | G | A | A | T | C | G | C | T | G | A | G | A | - | - | - | - | - | A | T | A | A | A | T | A | T | G | G | G | A | C | T | T | C | T | C |
| PC-5p-67443-24 | - | - | - | - | - | - | - | - | - | - | - | - | - | - | - | - | - | - | T | A | C | A | A | T | T | G | A | A | T | C | G | C | T | G | A | G | A | - | - | - | - | - | A | T | A | A | A | T | A | T | G | G | G | A | C | T | T | C | T | C |
| PC-5p-67443-66 | - | - | - | - | - | - | - | - | - | - | - | - | - | - | - | - | T | - | T | C | C | A | A | T | T | G | A | A | T | G | G | C | T | G | A | G | A | - | - | - | - | - | A | T | G | A | A | T | A | T | G | G | G | A | A | T | T | C | T | C |
| PC-5p-67443-69 | - | - | - | - | - | - | - | - | - | - | - | - | - | - | - | - | - | - | - | A | C | A | A | C | T | G | G | A | T | C | G | C | T | G | A | G | A | - | - | - | - | - | A | T | A | A | A | T | A | T | G | G | G | A | C | T | T | C | T | C |
| PC-5p-67443-49 | T | - | - | - | - | - | - | - | - | - | - | - | - | - | - | - | - | - | T | T | C | A | A | A | T | G | A | A | T | G | G | C | A | C | A | G | A | - | - | - | - | - | A | T | G | A | A | A | A | T | G | G | T | C | C | T | T | T | T | C |
| PC-5p-67443-83 | G | - | - | - | - | - | - | - | - | - | - | - | - | - | - | - | - | - | T | A | C | A | T | A | T | G | A | A | T | C | G | C | T | G | A | G | A | - | - | - | - | - | A | T | A | A | A | T | A | T | G | G | G | A | C | T | T | C | T | C |
| PC-5p-67443-53 | - | - | - | - | - | - | - | - | - | - | - | - | - | - | - | - | - | - | A | T | C | A | A | T | T | G | A | A | T | C | G | C | T | G | A | G | A | - | - | - | - | - | A | T | A | A | A | T | A | T | G | G | G | A | C | T | T | C | T | C |
| PC-5p-67443-62 | G | T | - | - | - | - | - | - | - | - | - | - | - | - | - | - | - | - | T | A | T | A | A | T | T | G | A | A | T | C | G | C | C | G | A | G | A | - | - | - | - | - | A | T | A | A | A | T | A | T | G | G | G | A | T | T | T | C | T | C |
| PC-5p-67443-37 | G | G | C | T | - | - | - | - | - | - | - | - | - | - | - | - | G | A | A | A | C | A | A | T | T | G | A | A | T | C | G | C | T | G | A | G | A | - | - | - | - | - | A | T | A | A | A | T | A | T | G | G | G | A | C | T | T | C | T | C |
| PC-5p-67443-40 | - | - | - | - | - | - | - | - | - | - | - | - | - | - | - | - | - | A | T | A | T | A | T | A | T | G | A | A | T | C | G | C | T | G | A | G | - | - | - | - | - | - | - | - | - | A | A | T | A | T | G | G | G | A | C | T | T | T | T | C |
| PC-5p-67443-48 | - | - | - | - | - | - | - | - | - | - | - | - | - | - | - | - | - | A | T | A | C | A | T | A | T | G | A | A | T | C | G | C | T | G | A | G | A | - | - | - | - | - | A | T | A | A | A | T | A | T | G | G | G | A | C | T | T | C | T | C |
| PC-5p-67443-60 | - | - | - | - | - | - | - | - | - | - | - | - | - | - | - | - | - | A | T | A | C | A | T | A | T | G | A | A | T | C | G | C | T | G | A | G | A | - | - | - | - | - | A | T | A | A | A | T | A | T | G | G | G | A | C | T | T | C | T | C |
| PC-5p-67443-26 | T | - | - | - | - | - | - | - | - | - | - | - | - | - | - | - | A | G | T | A | T | A | T | A | T | G | A | A | T | C | A | C | T | G | A | G | A | - | - | - | - | - | A | T | A | A | A | T | A | T | G | G | G | A | C | T | T | T | T | C |
| PC-5p-67443-59 | T | G | - | - | - | - | - | - | - | - | - | - | - | A | A | T | T | G | T | G | T | A | T | T | T | G | A | A | T | C | G | C | T | G | A | G | A | - | - | - | - | - | A | T | A | A | A | T | A | T | G | G | G | T | C | T | T | T | T | C |
| PC-5p-67443-35 | T | - | - | - | - | - | - | - | - | - | - | - | - | - | - | - | - | A | T | A | C | A | T | A | T | G | T | A | T | C | G | C | T | G | A | G | A | - | - | - | - | - | A | T | A | A | A | A | A | T | G | G | G | A | C | T | T | T | T | C |
| PC-5p-67443-44 | T | - | - | - | - | - | - | - | - | - | - | - | - | - | - | - | - | G | T | A | C | A | T | A | T | G | A | A | T | C | G | C | T | G | A | G | A | - | - | - | - | - | A | T | A | A | A | T | A | T | G | G | G | A | C | T | T | T | T | C |
| PC-5p-67443-2 | A | - | - | - | - | - | - | - | T | T | A | A | T | C | A | T | A | G | T | T | T | A | T | T | T | G | A | A | T | C | G | C | T | G | A | G | A | - | - | - | - | - | A | T | A | A | A | T | A | T | G | G | G | A | C | T | T | C | T | C |
| PC-5p-67443-34 | C | G | G | A | A | T | T | T | A | A | A | A | A | C | C | A | A | G | T | A | C | A | T | A | T | G | A | A | T | C | G | C | T | T | A | G | A | - | - | - | - | - | A | T | A | A | A | T | A | T | G | G | G | A | C | T | T | C | T | C |
| PC-5p-67443-5 | A | A | - | - | - | - | - | T | A | A | G | G | C | C | C | A | C | A | T | T | C | A | A | T | T | G | A | A | T | C | G | C | T | G | A | G | A | - | - | - | - | - | A | T | A | A | A | T | A | T | G | G | G | A | C | C | T | C | T | C |
| PC-5p-67443-67 | A | T | - | - | - | - | - | T | A | A | T | G | C | G | C | - | C | T | T | A | T | A | T | T | T | G | A | A | T | T | G | C | T | G | A | G | A | - | - | - | - | - | A | C | A | A | A | T | A | T | G | G | G | A | C | T | T | C | T | C |
| PC-5p-67443-52 | G | T | - | - | - | - | - | A | T | C | A | A | A | A | A | A | C | G | A | C | T | A | A | C | T | G | G | A | T | C | G | C | T | G | A | G | A | - | - | - | - | - | A | T | A | A | A | T | A | T | G | G | G | A | C | T | T | C | T | C |
| PC-5p-67443-63 | - | - | - | - | - | - | - | - | - | - | - | - | - | - | - | - | - | - | - | - | - | - | - | T | T | G | A | A | T | C | G | C | T | G | A | G | A | - | - | - | - | - | A | T | A | A | A | T | A | T | G | G | G | A | C | T | T | C | T | C |
| PC-5p-67443-70 | A | G | - | - | - | - | - | T | A | G | A | T | T | G | A | A | A | T | T | G | A | G | A | T | T | G | A | A | T | C | G | C | T | G | A | G | A | - | - | - | - | - | A | T | A | A | A | T | A | T | G | G | G | A | C | T | T | C | T | C |
| PC-5p-67443-61 | C | T | - | - | - | - | - | T | G | A | T | A | T | G | A | A | A | C | A | T | A | A | T | A | T | G | A | A | T | C | G | C | T | G | A | G | A | - | - | - | - | - | A | T | A | A | A | T | A | T | G | G | G | A | C | T | T | C | T | C |
| PC-5p-67443-82 | A | T | - | - | - | - | - | C | G | T | T | T | T | - | - | - | - | - | - | A | T | A | T | A | T | G | A | A | T | C | G | C | T | G | A | G | A | - | - | - | - | - | A | T | A | A | A | T | A | T | G | G | G | A | C | T | T | C | T | C |
| PC-5p-67443-30 | T | - | - | - | - | - | - | - | - | - | - | T | T | C | C | A | A | T | - | A | C | A | G | T | T | G | A | A | T | C | T | C | T | G | A | G | A | - | - | - | - | - | A | T | A | A | A | T | A | T | G | G | G | A | C | T | T | C | T | C |
| PC-5p-67443-87 | T | - | - | - | - | - | - | - | - | - | - | T | T | A | T | A | C | T | T | A | C | A | T | A | T | G | A | A | T | G | G | C | T | G | A | G | A | - | - | - | - | - | A | T | A | A | A | T | A | T | G | G | G | A | C | T | T | C | T | C |
| PC-5p-67443-77 | G | G | - | - | - | - | - | - | - | - | C | A | T | A | T | T | A | T | T | G | T | A | T | A | T | G | A | A | T | C | A | C | T | G | A | G | A | - | - | - | - | - | T | T | A | A | A | T | A | T | G | G | G | A | C | T | T | T | T | C |
| PC-5p-67443-13 | T | T | - | - | - | - | - | G | A | T | C | A | C | A | G | T | A | G | T | A | T | A | T | A | T | G | A | A | T | C | G | C | T | G | A | G | A | - | - | - | - | - | A | T | A | A | A | T | A | T | G | G | G | A | C | T | T | C | T | C |
| PC-5p-67443-36 | - | - | - | - | - | - | - | - | - | - | - | A | C | A | A | A | C | A | T | A | T | A | T | A | T | G | A | A | T | C | G | C | T | G | A | G | A | - | - | - | - | - | A | A | A | A | A | T | A | T | T | G | G | A | C | T | T | T | T | C |
| PC-5p-67443-27 | - | - | - | - | - | - | - | - | - | - | - | - | - | - | - | - | A | T | T | A | T | A | T | A | T | G | A | A | T | C | G | C | T | G | A | G | A | G | A | G | A | A | A | T | A | A | A | T | A | T | G | G | G | A | C | T | T | C | T | C |
| PC-5p-67443-65 | - | - | - | - | - | - | - | - | - | - | - | - | - | - | G | G | A | A | T | G | A | A | T | A | T | G | A | A | T | C | G | C | T | G | A | G | A | - | - | - | - | - | A | T | A | A | A | T | A | T | G | G | A | A | C | T | T | C | T | C |
| PC-5p-67443-16 | - | - | - | - | - | - | - | - | - | - | - | - | - | - | - | - | - | A | T | A | T | A | T | A | T | G | A | A | T | C | G | C | T | G | A | G | A | A | T | - | - | - | - | - | A | A | A | T | A | T | G | G | G | A | C | T | T | C | T | C |
| PC-5p-67443-19 | G | - | - | - | - | - | - | - | - | - | - | - | - | - | - | - | - | A | T | A | T | A | T | A | T | G | A | A | T | C | G | C | T | G | A | G | A | A | T | - | - | - | - | - | A | A | A | T | A | T | G | G | G | A | C | T | T | C | T | C |
| PC-5p-67443-22 | T | - | - | - | - | - | - | - | - | - | - | - | - | - | - | - | - | G | T | A | T | A | C | A | T | G | A | A | T | C | G | C | T | G | A | G | A | A | T | - | - | - | - | - | A | A | A | T | A | T | G | G | G | A | C | T | T | C | T | C |
| PC-5p-67443-38 | T | - | - | - | - | - | - | - | - | - | - | - | - | - | - | A | C | A | T | A | C | A | T | A | T | G | A | A | T | C | G | C | T | G | A | G | A | A | T | - | - | - | - | - | A | A | A | T | A | T | G | G | A | A | C | T | T | - | T | C |
| PC-5p-67443-39 | T | - | - | - | - | - | - | - | - | - | - | - | - | - | - | C | C | A | A | A | C | C | A | A | T | G | A | T | A | C | T | G | A | A | C | A | T | A | T | G | - | - | - | T | A | A | A | T | A | T | G | G | A | A | C | T | T | - | T | C |
| PC-5p-67443-43 | - | - | - | - | - | - | - | - | - | - | - | - | - | - | - | - | - | C | A | T | T | A | T | A | T | G | A | A | T | C | G | C | T | G | A | G | A | A | T | - | - | - | - | - | A | A | A | T | A | T | A | G | G | A | C | T | T | T | T | C |
| PC-5p-67443-72 | T | A | - | - | - | - | - | - | - | - | - | - | - | T | T | C | C | A | A | T | T | A | T | T | T | G | A | A | T | C | G | C | T | G | A | G | A | A | T | - | - | - | - | - | A | A | A | T | A | T | G | G | G | A | C | T | T | T | T | C |
| PC-5p-67443-73 | T | T | - | - | - | - | - | - | - | - | - | - | - | A | T | C | A | A | T | A | T | A | T | A | T | G | A | A | T | C | G | C | T | G | A | G | A | A | T | - | - | - | - | - | T | A | A | T | A | T | G | G | G | A | C | T | T | C | T | G |
| PC-5p-67443-4 | A | C | - | - | - | - | - | - | - | - | - | - | - | - | T | A | A | T | A | T | A | A | A | T | T | G | A | A | T | C | G | C | T | G | A | G | A | - | - | - | - | - | A | T | A | A | A | T | A | T | G | G | G | A | C | T | T | C | T | C |
| PC-5p-67443-75 | - | - | - | - | - | - | - | - | - | - | - | - | - | - | - | C | A | G | T | T | T | A | A | A | T | G | A | A | T | C | G | C | T | G | A | G | A | - | - | - | - | - | A | T | A | A | A | T | A | T | G | G | G | A | C | T | T | C | G | T |
| PC-5p-67443-10 | - | - | - | - | - | - | - | - | - | - | - | - | - | - | - | T | A | A | A | T | T | A | T | A | T | G | A | A | T | C | G | C | T | G | A | G | A | - | - | - | - | - | A | T | A | A | A | T | A | T | G | G | G | A | C | T | T | T | T | C |
| PC-5p-67443-41 | - | - | - | - | - | - | - | - | - | - | - | - | - | - | C | T | T | C | T | A | C | A | A | A | T | G | A | A | T | C | G | C | T | G | A | G | A | - | - | - | - | - | A | T | A | A | A | T | A | T | G | G | G | A | C | T | T | C | T | C |
| PC-5p-67443-88 | - | - | - | - | - | - | - | - | - | - | - | - | - | - | T | T | T | C | T | T | C | T | A | A | T | G | A | A | T | C | G | C | T | G | A | G | A | - | - | - | - | - | A | T | A | A | A | T | A | T | G | G | G | A | C | T | T | T | T | C |
| PC-5p-67443-54 | - | - | - | - | - | - | - | - | - | - | - | - | - | - | - | - | - | - | T | G | C | A | A | T | T | G | A | A | T | T | G | C | T | G | A | G | A | - | - | - | - | - | A | T | A | A | A | T | A | T | G | G | G | A | T | T | T | T | T | C |
| PC-5p-67443-3 | C | T | A | - | - | - | - | - | - | - | - | - | - | - | - | - | - | - | A | A | C | A | A | T | T | A | A | A | T | G | G | C | A | C | A | G | A | A | - | - | - | - | - | T | G | A | A | A | A | T | G | G | T | C | T | T | T | T | T | C |
| PC-5p-67443-25 | A | T | A | - | - | - | - | - | - | - | - | - | - | - | - | - | - | - | C | A | C | A | T | A | T | G | A | A | T | G | T | C | A | C | A | G | A | A | - | - | - | - | - | T | G | A | A | A | A | T | G | G | T | C | C | T | T | T | T | C |
| PC-5p-67443-80 | A | T | T | G | - | - | - | - | - | - | - | - | - | - | - | - | - | T | T | T | C | A | T | A | T | G | A | A | T | G | G | C | A | C | A | G | A | A | - | - | - | - | - | T | G | A | A | A | A | T | G | G | T | T | C | T | T | T | T | C |
| PC-5p-67443-6 | A | T | A | - | - | - | - | - | - | - | - | - | - | - | - | - | - | T | C | A | T | A | T | A | T | G | A | A | T | G | G | C | A | C | A | G | A | A | - | - | - | - | - | T | G | A | A | A | A | T | G | G | T | C | C | T | T | T | T | C |
| PC-5p-67443-91 | - | - | - | - | - | - | - | - | - | - | - | - | - | - | - | - | - | - | - | - | - | - | - | - | - | G | A | A | T | G | G | C | A | C | A | G | A | A | - | - | - | - | - | T | G | A | A | A | A | T | G | G | T | C | C | T | T | T | T | C |
| PC-5p-67443-17 | A | A | T | - | - | - | - | - | - | - | - | - | - | - | - | - | - | G | A | G | C | A | T | A | T | G | A | A | T | G | G | C | A | C | A | G | A | A | - | - | - | - | - | T | G | A | A | A | A | T | G | G | T | C | C | T | T | T | T | C |
| PC-5p-67443-9 | A | T | A | - | - | - | - | - | - | - | - | - | - | - | - | - | - | T | T | A | C | A | T | A | T | G | A | A | T | G | A | C | A | C | A | G | A | A | - | - | - | - | - | T | G | A | A | A | A | T | G | G | T | C | C | T | T | T | T | C |
| PC-5p-67443-58 | A | C | C | - | - | - | - | - | - | - | - | - | - | - | - | - | - | G | T | T | C | A | T | A | T | G | A | A | T | G | G | C | A | C | A | G | A | A | - | - | - | - | - | T | G | A | A | A | A | T | G | G | T | C | C | T | T | T | T | C |
| PC-5p-67443-28 | T | T | G | - | - | - | - | - | - | - | - | - | - | - | - | - | - | A | A | A | T | A | T | A | T | G | A | A | T | G | G | C | A | C | A | G | A | A | - | - | - | - | - | T | G | A | A | A | A | T | G | G | T | C | C | T | T | T | T | C |
| PC-5p-67443-31 | C | G | T | - | - | - | - | - | - | - | - | - | - | - | - | - | - | T | G | A | C | A | T | A | T | G | A | A | T | G | G | C | A | C | A | G | A | A | - | - | - | - | - | T | G | A | A | A | A | T | G | G | T | C | C | T | T | T | T | C |
| PC-5p-67443-47 | T | G | - | - | - | - | - | - | - | - | - | - | - | - | - | - | - | T | T | A | C | A | A | A | T | G | A | A | T | G | G | C | A | C | A | G | A | A | - | - | - | - | - | T | G | A | A | A | A | T | G | G | T | C | C | T | T | T | T | C |
| PC-5p-67443-71 | C | T | - | - | - | - | - | - | - | - | - | - | - | - | - | - | - | G | G | A | C | A | A | T | T | G | A | A | T | G | G | C | A | C | A | G | A | A | - | - | - | - | - | T | G | A | A | A | A | T | G | G | T | C | C | T | T | T | T | C |
| PC-5p-67443-51 | A | A | C | - | - | - | - | - | - | - | - | - | - | - | - | - | - | T | T | T | T | A | T | A | T | G | A | A | T | G | G | C | A | C | A | G | A | A | - | - | - | - | - | T | G | A | A | A | A | T | G | G | T | C | C | T | T | T | T | C |
| PC-5p-67443-74 | T | T | C | - | - | - | - | - | - | - | - | - | - | - | - | - | - | T | T | T | C | A | A | T | T | G | A | A | T | G | G | C | A | C | A | G | A | A | - | - | - | - | - | T | G | A | A | A | A | T | G | G | T | C | C | T | T | T | T | C |
| PC-5p-67443-21 | A | A | T | - | - | - | - | - | - | - | - | - | - | - | - | - | - | A | A | T | A | A | T | A | T | G | A | A | T | G | G | C | A | C | A | T | A | A | - | - | - | - | - | T | G | A | A | A | A | T | G | G | T | C | C | T | T | T | T | C |
| PC-5p-67443-20 | A | G | T | - | - | - | - | - | - | - | - | - | - | - | - | - | - | C | T | C | C | A | T | A | T | G | A | A | T | G | G | C | A | C | A | G | A | A | - | - | - | - | - | T | G | A | A | A | A | T | G | G | T | C | C | T | T | T | T | C |
| PC-5p-67443-42 | A | - | C | - | - | - | - | - | - | - | - | - | - | - | - | - | - | C | T | T | G | A | T | A | T | G | A | A | T | G | T | C | A | C | A | G | A | A | - | - | - | - | - | T | G | A | A | A | A | T | G | G | T | C | C | T | T | T | T | C |
| PC-5p-67443-90 | A | A | G | - | - | - | - | - | - | - | - | - | - | - | - | - | - | A | T | A | C | A | T | A | T | A | A | A | T | G | T | C | A | C | A | G | A | A | - | - | - | - | - | T | G | A | A | A | A | T | G | G | T | C | C | T | T | T | T | C |
| PC-5p-67443-14 | C | A | G | - | - | - | - | - | - | - | - | - | - | - | - | - | - | A | T | A | C | A | T | T | T | G | A | A | T | G | G | C | A | C | A | G | A | A | - | - | - | - | - | T | G | A | A | A | A | T | G | G | T | A | C | T | T | T | T | C |
| PC-5p-67443-18 | A | G | T | - | - | - | - | - | - | - | - | - | - | - | - | - | - | C | T | T | C | A | T | A | T | G | A | A | T | G | G | C | A | C | A | G | A | A | - | - | - | - | - | T | G | A | A | A | A | T | G | G | T | C | C | T | T | T | T | C |
| PC-5p-67443-15 | G | T | A | - | - | - | - | - | - | - | - | - | - | - | - | - | - | G | A | C | C | A | A | T | T | G | A | A | T | G | G | C | A | C | A | G | A | A | - | - | - | - | - | T | G | A | A | A | A | T | G | G | T | C | C | T | T | T | T | C |
| PC-5p-67443-50 | G | T | T | - | - | - | - | - | - | - | - | - | - | - | - | - | - | G | - | T | T | A | A | T | T | G | A | A | T | G | G | C | A | C | A | G | A | A | - | - | - | - | - | T | G | A | A | A | A | T | G | G | T | C | C | T | T | T | T | C |
| PC-5p-67443-45 | G | T | - | - | - | - | - | - | - | - | - | - | - | - | - | - | - | - | - | A | C | A | T | T | T | G | A | A | T | G | G | C | A | C | A | G | A | A | - | - | - | - | - | T | G | A | A | A | A | T | G | G | T | C | C | T | T | T | T | C |
| PC-5p-67443-57 | G | T | T | - | - | - | - | - | - | - | - | - | - | - | - | - | - | C | G | A | C | A | A | T | T | G | A | A | T | G | G | C | A | C | A | G | A | A | - | - | - | - | - | T | G | A | A | A | A | T | G | G | T | C | C | T | T | T | T | C |
| PC-5p-67443-76 | G | A | T | - | - | - | - | - | - | - | - | - | - | - | - | - | - | A | T | - | - | A | T | T | T | G | A | A | T | G | G | C | A | C | A | G | A | A | - | - | - | - | - | T | G | A | A | A | A | T | G | G | C | C | T | T | T | T | T | C |
| PC-5p-67443-86 | C | T | T | - | - | - | - | - | - | - | - | - | - | - | - | - | - | T | T | T | C | A | A | T | T | G | A | A | T | G | G | C | A | C | A | G | A | A | - | - | - | - | - | T | G | A | A | A | A | T | G | G | T | C | C | T | T | T | T | C |
| PC-5p-67443-55 | A | A | T | - | - | - | - | - | - | - | - | - | - | - | - | - | - | A | T | A | C | A | T | A | T | G | A | A | T | G | T | C | A | C | A | G | A | A | - | - | - | - | - | T | G | A | A | A | A | T | G | G | T | C | C | T | T | T | T | C |
| PC-5p-67443-56 | A | A | T | - | - | - | - | - | - | - | - | - | - | - | - | - | - | C | T | A | T | A | T | A | T | G | A | A | T | G | G | C | A | C | A | G | A | A | - | - | - | - | - | T | G | A | A | A | A | T | G | G | T | C | C | T | T | T | T | C |
| PC-5p-67443-68 | G | A | C | - | - | - | - | - | - | - | - | - | - | - | - | - | - | G | A | A | C | A | T | A | T | G | A | A | T | G | G | C | A | C | A | G | A | A | - | - | - | - | - | T | G | A | A | A | A | T | G | G | T | C | C | T | T | T | T | C |
| PC-5p-67443-79 | - | - | T | - | - | - | - | - | - | - | - | - | - | - | - | - | - | A | A | T | A | A | T | A | T | G | A | A | T | G | G | C | A | C | A | G | A | A | - | - | - | - | - | T | G | A | A | A | A | T | G | A | T | C | C | T | T | T | T | C |
| PC-5p-67443-84 | A | A | C | - | - | - | - | - | - | - | - | - | - | - | - | - | - | A | T | A | C | A | T | A | T | G | A | A | T | G | G | C | A | C | A | G | A | A | - | - | - | - | - | T | G | A | A | A | A | T | G | G | T | C | C | T | T | T | T | C |
| PC-5p-67443-81 | A | T | T | - | - | - | - | - | - | - | - | - | - | - | - | - | - | A | T | A | C | A | T | A | T | G | A | A | T | G | G | C | A | C | A | G | A | A | - | - | - | - | - | T | G | A | A | A | A | T | G | G | T | C | C | T | T | T | T | C |
| PC-5p-67443-8 | C | C | C | A | - | - | - | - | - | - | - | - | - | - | - | - | - | A | T | T | C | G | A | A | C | G | A | G | T | - | - | - | T | T | A | T | T | G | - | - | - | - | - | A | T | C | A | A | A | T | G | G | T | C | C | T | T | T | T | C |
| PC-5p-67443-46 | T | C | C | G | - | - | - | - | - | - | - | - | - | - | - | - | - | G | T | A | C | A | T | A | T | G | A | A | T | G | G | C | A | C | A | G | A | A | - | - | - | - | - | T | G | A | A | A | A | T | G | G | T | C | C | T | T | T | T | C |
| PC-5p-67443-29 | T | C | A | C | - | - | - | - | - | - | - | - | - | - | - | - | - | C | T | A | C | A | A | A | T | G | A | A | T | G | G | C | A | C | A | G | A | A | - | - | - | - | - | T | A | A | A | A | A | T | G | G | T | C | C | T | T | T | T | C |
| PC-5p-67443-78 | T | T | T | G | - | - | - | - | - | - | - | - | - | - | - | - | - | T | T | G | - | A | A | A | T | G | A | A | T | G | G | C | A | T | A | G | A | A | - | - | - | - | - | T | G | A | A | A | G | C | G | A | T | C | C | T | C | T | T | C |
| PC-5p-67443-11 | G | G | C | A | - | - | - | - | - | - | - | - | - | - | - | - | - | C | A | G | A | A | T | G | A | A | A | A | T | G | G | C | A | C | A | G | A | A | - | - | - | - | - | T | G | A | A | A | A | T | G | G | T | C | C | T | T | T | T | C |
| PC-5p-67443-32 | C | T | T | T | - | - | - | - | - | - | - | - | - | - | - | - | - | C | T | A | T | A | T | A | T | G | A | A | T | G | G | C | A | C | A | G | A | A | - | - | - | - | - | T | A | A | A | A | A | T | G | G | T | C | C | T | T | T | T | C |
| PC-5p-67443-33 | T | T | C | C | - | - | - | - | - | - | - | - | - | - | - | - | - | C | C | A | T | A | T | A | T | G | A | A | T | G | G | C | A | C | A | G | A | A | - | - | - | - | - | T | G | A | A | A | A | T | G | G | T | C | C | T | T | T | T | C |
| PC-5p-67443-64 | T | A | A | A | - | - | - | - | - | - | - | - | - | - | - | - | - | A | C | A | C | A | A | T | T | G | A | A | T | G | G | C | A | C | A | G | A | A | - | - | - | - | - | T | G | A | A | A | A | T | G | G | C | C | C | T | T | T | T | C |
| PC-5p-67443-85 | - | G | A | A | - | - | - | - | - | - | - | - | - | - | - | - | - | A | C | A | C | A | A | T | T | G | A | A | T | C | G | C | T | G | A | G | A | A | - | - | - | - | - | T | A | A | A | T | A | T | G | G | A | A | C | T | T | C | T | C |

**57811 ends┤** ┤**67443 ends ├Complementary region starts**

| PC-5p-67443-1 | A | A | - | A | A | C | T | G | C | G | G | A | A | G | A | T | A | A | T | C | C | A | A | G | C | A | T | A | A | A | A | T | A | A | A | T | G | - | - | - | - | - | - | - | - | C | G | T | T | - | - | - | - | - | - | - | - | - | - | - |
| --- | --- | --- | --- | --- | --- | --- | --- | --- | --- | --- | --- | --- | --- | --- | --- | --- | --- | --- | --- | --- | --- | --- | --- | --- | --- | --- | --- | --- | --- | --- | --- | --- | --- | --- | --- | --- | --- | --- | --- | --- | --- | --- | --- | --- | --- | --- | --- | --- | --- | --- | --- | --- | --- | --- | --- | --- | --- | --- | --- | --- |
| PC-5p-67443-12 | A | A | - | A | A | C | T | G | C | G | G | A | A | G | A | T | A | A | T | C | C | C | A | G | C | A | T | A | A | A | A | T | A | A | A | T | G | - | - | - | - | - | - | - | - | C | A | T | T | - | - | - | - | - | - | - | - | - | - | - |
| PC-5p-67443-7 | A | A | - | A | A | C | T | G | C | G | G | A | A | G | A | T | A | A | T | C | C | C | A | G | C | A | T | A | A | A | A | T | A | A | A | T | G | - | - | - | - | - | - | - | - | C | G | T | T | - | - | - | - | - | - | - | - | - | - | - |
| PC-5p-67443-89 | A | A | - | A | A | C | T | G | C | G | G | A | A | G | A | T | A | A | T | C | C | C | A | G | C | A | T | A | A | A | A | T | A | A | A | T | G | - | - | - | - | - | - | - | - | C | G | T | T | - | - | - | - | - | - | - | - | - | - | - |
| PC-5p-67443-23 | A | A | - | A | A | C | T | G | C | G | G | A | A | G | A | T | A | A | T | C | C | C | A | G | C | A | T | A | A | A | A | T | A | A | A | T | G | - | - | - | - | - | - | - | - | C | G | T | T | - | - | - | - | - | - | - | - | - | - | - |
| PC-5p-67443-24 | A | A | - | A | A | C | T | G | C | G | G | A | A | G | A | T | A | A | T | C | C | C | A | G | C | A | T | A | A | A | A | T | A | A | A | T | G | - | - | - | - | - | - | - | - | C | G | T | T | - | - | - | - | - | - | - | - | - | - | - |
| PC-5p-67443-66 | A | A | - | A | A | C | T | G | C | G | G | A | A | G | A | T | A | A | T | C | T | C | A | G | C | A | T | A | A | A | A | T | A | A | A | T | G | - | - | - | - | - | - | - | - | C | G | T | T | - | - | - | - | - | - | - | - | - | - | - |
| PC-5p-67443-69 | A | A | - | A | A | C | T | G | C | G | G | A | A | G | A | T | A | A | T | C | A | C | A | G | C | A | T | A | A | A | A | T | A | A | A | T | G | - | - | - | - | - | - | - | - | C | G | T | T | - | - | - | - | - | - | - | - | - | - | - |
| PC-5p-67443-49 | A | A | - | A | A | C | T | G | C | G | G | A | A | G | A | T | A | A | T | C | C | C | A | G | C | A | T | A | A | A | A | T | A | A | A | T | G | - | - | - | - | - | - | - | - | C | G | T | T | - | - | - | - | - | - | - | - | - | - | - |
| PC-5p-67443-83 | A | A | - | A | A | C | A | G | C | G | G | A | A | G | A | T | A | A | T | C | C | C | A | G | C | A | T | A | A | A | A | T | A | A | A | T | G | - | - | - | - | - | - | - | - | C | G | T | T | - | - | - | - | - | - | - | - | - | - | - |
| PC-5p-67443-53 | A | A | - | A | A | C | T | G | C | G | G | A | A | G | A | T | A | A | T | C | C | C | A | G | C | A | T | A | A | A | A | T | A | A | A | T | G | - | - | - | - | - | - | - | - | C | G | T | T | - | - | - | - | - | - | - | - | - | - | - |
| PC-5p-67443-62 | A | A | - | A | A | C | T | G | C | G | G | A | A | G | A | T | A | A | T | C | C | C | A | G | C | A | T | A | A | A | A | T | A | A | A | A | G | - | - | - | - | - | - | - | - | C | G | T | T | - | - | - | - | - | - | - | - | - | - | - |
| PC-5p-67443-37 | A | A | - | A | A | C | T | G | C | G | G | A | A | G | A | T | A | A | T | C | C | C | A | G | C | A | T | A | A | A | A | T | A | A | A | T | G | - | - | - | - | - | - | - | - | C | G | T | T | - | - | - | - | - | - | - | - | - | - | - |
| PC-5p-67443-40 | A | A | - | A | A | C | T | G | C | G | G | A | A | G | A | T | A | A | T | C | C | C | A | G | C | A | C | A | A | A | A | T | A | A | A | T | G | - | - | - | - | - | - | - | - | C | G | T | T | - | - | - | - | - | - | - | - | - | - | - |
| PC-5p-67443-48 | A | A | - | A | A | C | T | G | C | G | G | A | A | G | A | T | A | A | T | C | C | C | A | G | C | A | T | A | A | A | A | T | A | A | A | T | G | - | - | - | - | - | - | - | - | C | G | T | T | - | - | - | - | - | - | - | - | - | - | - |
| PC-5p-67443-60 | A | A | - | A | A | C | T | G | C | G | G | A | A | G | A | T | A | A | T | C | C | C | A | G | C | A | T | A | A | A | A | T | A | A | A | T | G | - | - | - | - | - | - | - | - | C | G | T | T | - | - | - | - | - | - | - | - | - | - | - |
| PC-5p-67443-26 | A | A | - | A | A | C | T | G | C | G | G | A | A | G | A | T | A | A | T | C | C | C | A | G | C | A | T | A | A | A | A | T | A | A | A | T | G | - | - | - | - | - | - | - | - | C | G | T | T | - | - | - | - | - | - | - | - | - | - | - |
| PC-5p-67443-59 | A | A | - | A | A | C | T | G | C | G | A | A | A | G | A | T | A | A | T | C | C | C | A | G | C | A | T | A | A | A | A | T | A | A | A | T | G | - | - | - | - | - | - | - | - | C | G | T | A | - | - | - | - | - | - | - | - | - | - | - |
| PC-5p-67443-35 | A | A | - | A | A | C | T | G | C | G | G | A | A | G | A | T | A | A | T | C | C | C | A | G | C | A | T | G | A | A | A | T | A | A | A | T | G | - | - | - | - | - | - | - | - | C | G | T | T | - | - | - | - | - | - | - | - | - | - | - |
| PC-5p-67443-44 | A | A | - | A | A | C | T | G | C | G | G | A | A | G | A | T | A | A | T | C | C | C | A | G | C | A | T | A | A | A | A | T | A | A | A | T | G | - | - | - | - | - | - | - | - | C | G | T | T | - | - | - | - | - | - | - | - | - | - | - |
| PC-5p-67443-2 | A | A | - | A | A | C | T | G | C | G | G | A | A | G | A | T | A | A | T | C | C | T | A | G | C | A | T | A | A | A | A | T | A | A | A | T | G | - | - | - | - | - | - | - | - | C | G | T | T | - | - | - | - | - | - | - | - | - | - | - |
| PC-5p-67443-34 | A | A | - | A | A | C | T | G | C | G | G | A | A | G | A | T | A | A | T | C | C | C | A | G | C | A | T | A | A | A | A | T | A | A | A | T | G | - | - | - | - | - | - | - | - | C | G | T | T | - | - | - | - | - | - | - | - | - | - | - |
| PC-5p-67443-5 | A | A | - | A | A | C | T | G | C | G | G | A | A | G | A | T | A | A | T | C | C | C | A | G | C | A | T | C | T | A | A | T | A | A | A | T | G | - | - | - | - | - | - | - | - | C | G | T | T | - | - | - | - | - | - | - | - | - | - | - |
| PC-5p-67443-67 | A | A | - | A | A | C | T | G | C | G | G | A | A | G | A | T | A | A | T | C | A | C | A | G | C | A | T | A | A | A | A | T | A | A | A | T | G | - | - | - | - | - | - | - | - | C | G | T | T | - | - | - | - | - | - | - | - | - | - | - |
| PC-5p-67443-52 | A | A | - | A | A | T | T | G | C | G | G | A | A | G | A | T | A | A | C | G | C | A | T | T | T | A | T | T | T | T | A | T | G | C | T | G | G | - | - | - | - | - | - | - | - | G | A | T | T | - | - | - | - | - | - | - | - | - | - | - |
| PC-5p-67443-63 | A | A | - | A | A | T | T | G | C | G | G | A | A | G | A | T | A | A | C | G | C | T | T | T | T | A | T | T | T | T | A | T | G | C | T | G | G | - | - | - | - | - | - | - | - | G | A | T | T | - | - | - | - | - | - | - | - | - | - | - |
| PC-5p-67443-70 | A | A | - | A | A | T | T | G | C | G | G | A | A | G | A | T | A | A | C | G | C | A | T | T | T | A | T | T | T | T | A | T | G | C | T | G | T | - | - | - | - | - | - | - | - | G | A | T | T | - | - | - | - | - | - | - | - | - | - | - |
| PC-5p-67443-61 | A | A | - | A | A | C | T | G | C | G | G | A | A | G | A | T | A | A | C | G | C | A | T | T | T | A | T | T | T | T | A | T | G | C | T | G | G | - | - | - | - | - | - | - | - | G | A | T | T | - | - | - | - | - | - | - | - | - | - | - |
| PC-5p-67443-82 | A | A | - | A | A | C | T | G | C | G | G | G | A | G | A | T | A | A | T | C | C | C | A | G | C | A | T | A | A | A | A | T | A | A | A | T | G | - | - | - | - | - | - | - | - | C | G | T | T | - | - | - | - | - | - | - | - | - | - | - |
| PC-5p-67443-30 | A | A | - | A | A | C | T | G | C | G | G | A | A | G | A | T | A | A | T | C | C | C | A | G | C | A | T | A | A | A | A | T | A | A | A | T | G | - | - | - | - | - | - | - | - | C | G | T | T | - | - | - | - | - | - | - | - | - | - | - |
| PC-5p-67443-87 | A | A | - | A | A | C | T | G | C | G | G | A | C | G | A | T | A | A | T | C | C | C | A | G | C | A | T | A | A | A | A | T | G | A | A | T | G | - | - | - | - | - | - | - | - | C | G | T | T | - | - | - | - | - | - | - | - | - | - | - |
| PC-5p-67443-77 | A | A | - | A | A | C | T | G | C | G | G | A | A | G | A | T | A | A | A | C | C | C | A | G | C | A | T | A | A | A | A | T | A | A | A | T | G | - | - | - | - | - | - | - | - | C | G | T | T | - | - | - | - | - | - | - | - | - | - | - |
| PC-5p-67443-13 | A | A | - | A | A | C | T | G | C | G | G | A | A | G | A | T | A | A | T | C | C | C | A | G | C | A | T | A | A | A | A | T | A | A | A | T | G | - | - | - | - | - | - | - | - | C | G | T | T | - | - | - | - | - | - | - | - | - | - | - |
| PC-5p-67443-36 | A | A | - | A | A | C | T | G | C | G | G | A | A | G | A | T | A | A | T | C | C | C | A | G | C | A | T | A | A | A | A | T | A | A | A | T | G | - | - | - | - | - | - | - | - | C | G | T | T | - | - | - | - | - | - | - | - | - | - | - |
| PC-5p-67443-27 | A | A | - | A | A | C | T | G | C | G | G | A | A | G | A | T | A | A | T | C | C | C | A | G | C | A | T | A | A | A | A | T | A | A | A | T | G | - | - | - | - | - | - | - | - | C | G | T | T | - | - | - | - | - | - | - | - | - | - | - |
| PC-5p-67443-65 | A | A | - | A | A | C | T | G | C | G | G | A | A | G | A | T | A | A | T | C | C | C | A | G | C | A | T | A | A | A | A | T | A | A | A | T | G | - | - | - | - | - | - | - | - | C | G | T | T | - | - | - | - | - | - | - | - | - | - | - |
| PC-5p-67443-16 | A | A | - | A | A | C | T | G | C | G | G | A | A | G | A | T | A | A | T | C | C | C | A | G | C | A | T | A | A | A | A | T | A | A | A | T | G | - | - | - | - | - | - | - | - | C | G | T | T | - | - | - | - | - | - | - | - | - | - | - |
| PC-5p-67443-19 | A | A | - | A | A | C | T | G | C | G | G | A | A | G | A | T | A | A | T | C | C | C | A | G | C | A | T | A | A | A | A | T | A | A | A | T | G | - | - | - | - | - | - | - | - | C | G | T | T | - | - | - | - | - | - | - | - | - | - | - |
| PC-5p-67443-22 | A | A | - | A | A | C | T | G | C | G | G | A | A | G | A | T | A | A | T | C | C | C | A | G | C | A | T | A | A | A | A | T | A | A | A | T | G | - | - | - | - | - | - | - | - | C | G | T | T | - | - | - | - | - | - | - | - | - | - | - |
| PC-5p-67443-38 | A | A | - | A | A | C | T | G | C | G | G | A | A | G | A | T | A | A | T | C | C | C | A | G | C | A | T | A | A | A | A | T | A | A | A | T | G | - | - | - | - | - | - | - | - | C | G | T | T | - | - | - | - | - | - | - | - | - | - | - |
| PC-5p-67443-39 | A | A | - | A | A | C | T | G | C | G | G | A | A | G | A | T | A | A | T | C | C | C | A | G | C | A | T | A | A | A | A | T | A | A | A | T | G | - | - | - | - | - | - | - | - | C | G | T | T | - | - | - | - | - | - | - | - | - | - | - |
| PC-5p-67443-43 | A | A | - | A | A | C | T | G | C | G | G | A | A | G | A | T | A | A | T | C | C | C | A | G | C | A | T | A | A | A | A | T | A | A | A | T | G | - | - | - | - | - | - | - | - | C | A | T | T | - | - | - | - | - | - | - | - | - | - | - |
| PC-5p-67443-72 | A | A | - | A | A | C | T | G | C | G | G | A | A | G | A | T | A | A | T | T | C | C | A | G | C | A | T | A | A | A | A | T | A | A | A | T | G | - | - | - | - | - | - | - | - | C | G | T | T | - | - | - | - | - | - | - | - | - | - | - |
| PC-5p-67443-73 | A | A | - | A | A | C | T | G | C | G | G | A | A | G | A | T | A | A | T | C | C | C | A | G | C | A | T | A | A | A | A | T | A | A | A | T | G | - | - | - | - | - | - | - | - | C | G | T | T | - | - | - | - | - | - | - | - | - | - | - |
| PC-5p-67443-4 | A | A | - | A | A | C | T | G | C | G | G | A | A | G | A | T | A | A | T | C | C | C | A | G | C | A | T | A | A | A | A | T | A | A | A | T | G | - | - | - | - | - | - | - | - | C | G | T | T | - | - | - | - | - | - | - | - | - | - | - |
| PC-5p-67443-75 | A | A | - | A | A | C | T | G | C | G | G | A | A | G | A | T | A | A | T | C | C | C | A | G | C | A | T | A | A | A | A | T | A | A | A | T | G | - | - | - | - | - | - | - | - | C | G | T | T | - | - | - | - | - | - | - | - | - | - | - |
| PC-5p-67443-10 | A | A | - | A | A | C | T | G | C | G | G | A | A | G | A | T | A | A | T | C | C | C | A | G | C | A | T | A | A | A | A | T | A | A | A | T | G | - | - | - | - | - | - | - | - | C | G | T | T | - | - | - | - | - | - | - | - | - | - | - |
| PC-5p-67443-41 | A | A | - | A | A | C | T | G | C | G | G | A | A | G | A | T | A | A | T | C | C | C | A | G | C | A | T | A | A | A | A | T | A | A | A | T | G | - | - | - | - | - | - | - | - | C | G | T | T | - | - | - | - | - | - | - | - | - | - | - |
| PC-5p-67443-88 | A | A | - | A | A | C | T | G | C | G | G | A | A | G | A | T | A | A | T | C | C | C | A | G | C | A | T | A | A | A | A | T | A | A | A | T | G | - | - | - | - | - | - | - | - | C | G | T | T | - | - | - | - | - | - | - | - | - | - | - |
| PC-5p-67443-54 | A | A | - | A | A | C | T | G | C | G | G | A | A | G | A | T | A | A | T | C | C | C | A | G | C | A | T | A | A | A | A | T | G | A | A | T | G | - | - | - | - | - | - | - | - | C | A | T | T | - | - | - | - | - | - | - | - | - | - | - |
| PC-5p-67443-3 | A | A | - | A | A | C | T | G | C | G | G | A | A | G | A | T | A | A | T | C | C | C | A | G | C | A | T | A | A | A | A | T | A | A | A | T | G | - | - | - | - | - | - | - | - | C | G | T | T | - | - | - | - | - | - | - | - | - | - | - |
| PC-5p-67443-25 | A | A | - | A | A | C | T | G | C | G | G | A | A | G | A | T | A | A | T | C | C | C | A | G | C | A | T | A | A | A | G | T | A | A | A | T | G | - | - | - | - | - | - | - | - | C | G | T | T | - | - | - | - | - | - | - | - | - | - | - |
| PC-5p-67443-80 | A | A | - | A | A | C | T | G | C | G | G | A | A | T | A | T | A | A | T | C | C | C | A | G | C | A | T | A | A | A | A | T | A | A | A | T | G | - | - | - | - | - | - | - | - | C | G | T | T | - | - | - | - | - | - | - | - | - | - | - |
| PC-5p-67443-6 | A | A | - | A | A | C | T | G | C | G | G | A | A | G | A | T | A | A | T | C | C | C | A | G | C | A | T | A | A | A | A | T | A | A | A | T | G | - | - | - | - | - | - | - | - | C | G | T | T | - | - | - | - | - | - | - | - | - | - | - |
| PC-5p-67443-91 | A | A | - | A | A | C | T | G | C | G | G | A | A | G | A | T | A | A | T | C | C | C | A | G | C | A | T | A | A | A | A | T | A | A | A | T | G | - | - | - | - | - | - | - | - | C | G | T | T | - | - | - | - | - | - | - | - | - | - | - |
| PC-5p-67443-17 | A | A | - | A | A | C | T | G | C | G | G | A | A | G | A | T | A | A | T | C | C | C | A | G | C | A | T | A | A | A | A | T | A | A | A | G | G | - | - | - | - | - | - | - | - | C | G | T | T | - | - | - | - | - | - | - | - | - | - | - |
| PC-5p-67443-9 | A | A | - | A | A | C | T | G | C | G | G | A | A | G | A | T | A | A | T | C | C | C | A | G | C | A | T | A | A | A | A | T | A | A | A | T | G | - | - | - | - | - | - | - | - | C | G | T | T | - | - | - | - | - | - | - | - | - | - | - |
| PC-5p-67443-58 | A | A | - | A | A | C | T | G | C | G | G | A | A | G | A | T | A | A | T | C | C | C | A | G | C | A | T | A | A | A | A | T | A | A | A | T | G | - | - | - | - | - | - | - | - | C | G | T | T | - | - | - | - | - | - | - | - | - | - | - |
| PC-5p-67443-28 | A | A | - | A | A | C | T | G | C | G | G | A | A | G | A | T | A | A | T | C | C | C | A | G | C | A | T | A | A | A | A | T | A | A | A | T | G | - | - | - | - | - | - | - | - | C | G | T | T | - | - | - | - | - | - | - | - | - | - | - |
| PC-5p-67443-31 | A | A | - | A | A | C | T | G | C | G | G | A | A | G | A | T | A | A | T | C | C | C | A | G | C | A | T | A | A | A | A | T | C | A | A | T | G | - | - | - | - | - | - | - | - | C | G | T | T | - | - | - | - | - | - | - | - | - | - | - |
| PC-5p-67443-47 | A | A | - | A | A | C | T | G | C | G | G | A | A | G | A | T | A | A | T | C | C | C | A | G | C | A | T | A | A | A | A | T | A | A | A | T | G | - | - | - | - | - | - | - | - | C | G | T | T | - | - | - | - | - | - | - | - | - | - | - |
| PC-5p-67443-71 | A | A | - | A | A | C | T | G | C | G | G | A | A | G | A | T | A | A | T | C | T | C | A | G | C | A | T | A | A | A | A | T | A | A | A | T | G | - | - | - | - | - | - | - | - | C | G | T | T | - | - | - | - | - | - | - | - | - | - | - |
| PC-5p-67443-51 | A | A | - | A | A | C | T | G | C | G | G | A | A | G | A | T | A | A | T | C | C | C | A | G | C | A | T | A | A | A | A | T | A | A | A | T | G | - | - | - | - | - | - | - | - | C | G | T | T | - | - | - | - | - | - | - | - | - | - | - |
| PC-5p-67443-74 | A | A | - | A | A | C | T | G | C | G | G | A | A | G | A | T | A | A | T | A | A | C | A | G | C | A | T | A | A | A | A | T | A | A | A | T | G | - | - | - | - | - | - | - | - | C | G | T | T | - | - | - | - | - | - | - | - | - | - | - |
| PC-5p-67443-21 | A | A | - | A | A | C | T | G | C | G | G | A | A | G | A | T | A | A | T | C | C | C | A | G | C | A | T | A | A | A | A | T | A | A | A | C | A | A | T | A | A | T | A | A | A | C | A | A | T | A | A | T | A | T | A | A | G | A | A | G |
| PC-5p-67443-20 | A | A | - | A | A | C | T | G | C | G | G | A | A | G | A | T | A | A | T | C | C | C | A | G | C | A | T | A | A | A | A | T | A | A | A | T | G | - | - | - | - | - | - | - | - | C | G | T | T | - | - | - | - | - | - | - | - | - | - | - |
| PC-5p-67443-42 | A | A | - | A | A | C | T | G | C | G | G | A | A | G | A | T | A | A | T | C | C | C | A | G | C | A | T | A | A | A | A | T | A | A | A | T | G | - | - | - | - | - | - | - | - | C | G | T | T | - | - | - | - | - | - | - | - | - | - | - |
| PC-5p-67443-90 | A | A | - | A | A | C | T | G | C | G | G | A | A | G | A | T | A | A | T | C | C | C | A | G | C | A | T | A | A | A | A | T | A | A | A | T | G | - | - | - | - | - | - | - | - | C | G | T | T | - | - | - | - | - | - | - | - | - | - | - |
| PC-5p-67443-14 | A | A | - | A | A | C | T | G | C | G | G | A | A | G | A | T | A | A | T | C | C | C | A | G | C | A | T | A | A | A | A | T | A | A | A | T | G | - | - | - | - | - | - | - | - | C | G | T | T | - | - | - | - | - | - | - | - | - | - | - |
| PC-5p-67443-18 | A | A | - | A | A | C | T | G | C | G | G | A | A | G | A | T | A | A | T | C | C | C | A | G | A | A | T | A | A | A | A | T | A | A | A | T | G | - | - | - | - | - | - | - | - | C | G | T | T | - | - | - | - | - | - | - | - | - | - | - |
| PC-5p-67443-15 | A | A | - | A | A | C | T | G | C | G | G | A | A | G | A | T | A | A | T | C | C | C | A | G | C | A | T | A | A | A | A | T | A | A | A | T | G | - | - | - | - | - | - | - | - | C | G | T | T | - | - | - | - | - | - | - | - | - | - | - |
| PC-5p-67443-50 | A | A | - | A | A | C | T | G | C | G | G | A | A | G | A | T | A | A | T | C | C | C | A | C | C | A | T | A | A | A | A | T | A | A | A | T | G | - | - | - | - | - | - | - | - | C | G | T | T | - | - | - | - | - | - | - | - | - | - | - |
| PC-5p-67443-45 | A | A | - | A | A | C | T | G | C | G | G | A | A | G | A | T | A | A | T | C | C | C | A | G | C | A | T | A | A | A | A | T | A | A | A | T | G | - | - | - | - | - | - | - | - | C | G | T | T | - | - | - | - | - | - | - | - | - | - | - |
| PC-5p-67443-57 | A | A | - | A | A | C | T | G | C | G | G | A | A | G | A | T | A | A | T | C | C | C | A | G | C | A | T | A | A | A | A | T | A | A | A | T | G | - | - | - | - | - | - | - | - | C | G | T | T | - | - | - | - | - | - | - | - | - | - | - |
| PC-5p-67443-76 | A | A | A | A | A | C | T | G | C | G | G | A | A | G | A | T | A | A | T | C | C | C | A | G | C | A | T | A | A | A | A | T | A | A | A | T | G | - | - | - | - | - | - | - | - | C | G | T | T | - | - | - | - | - | - | - | - | - | - | - |
| PC-5p-67443-86 | A | G | - | A | A | C | T | G | C | G | G | A | A | G | A | T | A | A | T | C | C | C | A | G | C | A | T | A | A | A | A | T | A | A | A | T | G | - | - | - | - | - | - | - | - | C | G | T | T | - | - | - | - | - | - | - | - | - | - | - |
| PC-5p-67443-55 | A | A | - | A | A | C | T | G | C | G | G | A | A | G | A | T | A | A | T | C | C | C | A | G | C | A | T | A | A | A | A | T | A | A | A | T | G | - | - | - | - | - | - | - | - | C | G | T | T | - | - | - | - | - | - | - | - | - | - | - |
| PC-5p-67443-56 | A | A | - | A | A | C | T | G | C | G | G | A | A | G | A | T | A | A | T | C | C | C | A | G | C | A | T | G | A | A | A | T | A | A | A | T | G | - | - | - | - | - | - | - | - | C | G | T | T | - | - | - | - | - | - | - | - | - | - | - |
| PC-5p-67443-68 | A | A | - | A | A | C | T | G | C | G | G | A | A | G | A | T | A | A | T | C | T | C | A | G | C | A | T | A | A | A | A | T | A | A | A | T | G | - | - | - | - | - | - | - | - | C | G | T | T | - | - | - | - | - | - | - | - | - | - | - |
| PC-5p-67443-79 | A | A | - | A | A | C | T | G | C | G | A | A | A | G | A | T | A | A | T | C | C | C | A | G | C | A | T | A | A | A | A | T | A | A | A | T | A | - | - | - | - | - | - | - | - | C | G | T | T | - | - | - | - | - | - | - | - | - | - | - |
| PC-5p-67443-84 | A | T | - | A | A | C | T | G | C | G | G | A | A | G | A | T | A | A | T | C | C | C | A | G | C | A | T | A | A | A | A | T | A | A | A | T | G | - | - | - | - | - | - | - | - | C | G | T | T | - | - | - | - | - | - | - | - | - | - | - |
| PC-5p-67443-81 | A | A | - | A | A | C | T | G | C | G | A | A | A | G | A | T | A | A | T | C | C | C | A | G | C | A | T | A | A | A | A | T | A | A | A | T | G | - | - | - | - | - | - | - | - | C | A | T | T | - | - | - | - | - | - | - | - | - | - | - |
| PC-5p-67443-8 | A | A | - | A | A | C | T | G | C | G | G | A | A | G | A | T | A | A | T | C | C | C | A | G | C | A | T | A | G | A | A | T | A | A | A | T | G | - | - | - | - | - | - | - | - | C | G | T | T | - | - | - | - | - | - | - | - | - | - | - |
| PC-5p-67443-46 | A | A | - | A | A | C | T | G | C | G | G | A | A | G | A | T | A | A | T | C | C | A | A | G | C | A | T | A | A | A | A | T | A | A | A | T | G | - | - | - | - | - | - | - | - | C | G | T | T | - | - | - | - | - | - | - | - | - | - | - |
| PC-5p-67443-29 | A | A | - | A | A | C | T | G | C | G | G | A | A | G | A | T | A | A | T | C | C | C | A | G | C | A | T | A | A | A | A | T | A | A | A | T | G | - | - | - | - | - | - | - | - | C | G | T | T | - | - | - | - | - | - | - | - | - | - | - |
| PC-5p-67443-78 | A | A | - | A | A | C | T | G | C | G | G | A | A | G | A | T | A | G | T | C | C | C | A | G | C | A | T | A | A | A | A | T | A | A | A | T | G | - | - | - | - | - | - | - | - | C | G | T | T | - | - | - | - | - | - | - | - | - | - | - |
| PC-5p-67443-11 | A | A | - | A | A | C | T | G | C | G | G | A | A | G | A | T | A | A | T | C | C | C | A | G | C | A | T | A | A | A | A | T | A | A | A | T | G | - | - | - | - | - | - | - | - | C | G | T | T | - | - | - | - | - | - | - | - | - | - | - |
| PC-5p-67443-32 | A | A | - | A | A | C | T | G | C | G | G | A | A | G | A | T | A | A | T | C | C | C | A | G | C | A | T | A | A | A | A | T | A | A | A | G | G | - | - | - | - | - | - | - | - | C | G | T | T | - | - | - | - | - | - | - | - | - | - | - |
| PC-5p-67443-33 | A | A | - | A | A | C | T | G | C | G | G | A | A | G | A | T | A | A | T | C | C | C | A | G | C | A | T | A | A | A | A | T | A | A | A | T | G | - | - | - | - | - | - | - | - | C | G | T | T | - | - | - | - | - | - | - | - | - | - | - |
| PC-5p-67443-64 | A | A | - | A | A | C | T | G | C | G | G | A | A | G | A | T | A | A | T | C | C | C | A | G | C | A | T | A | A | A | A | T | A | A | A | T | G | - | - | - | - | - | - | - | - | C | G | T | T | - | - | - | - | - | - | - | - | - | - | - |
| PC-5p-67443-85 | A | A | - | A | A | C | T | G | C | A | G | A | A | G | A | T | A | A | T | C | C | C | A | G | C | A | T | A | A | A | A | T | A | A | A | T | G | - | - | - | - | - | - | - | - | C | G | T | T | - | - | - | - | - | - | - | - | - | - | - |

**┤Complementary region ends**

| PC-5p-67443-1 | - | - | - | - | - | - | A | T | C | T | T | C | C | - | G | C | A | A | T | T | T | C | G | A | G | A | A | G | T | C | C | T | A | T | A | T | T | T | A | T | T | C | T | C | A | G | - | C | A | - | - | A | T | T | C | A | A | T | T | - |
| --- | --- | --- | --- | --- | --- | --- | --- | --- | --- | --- | --- | --- | --- | --- | --- | --- | --- | --- | --- | --- | --- | --- | --- | --- | --- | --- | --- | --- | --- | --- | --- | --- | --- | --- | --- | --- | --- | --- | --- | --- | --- | --- | --- | --- | --- | --- | --- | --- | --- | --- | --- | --- | --- | --- | --- | --- | --- | --- | --- | --- |
| PC-5p-67443-12 | - | - | - | - | - | - | A | T | C | T | T | C | C | - | G | C | A | A | T | T | T | C | G | A | G | A | A | G | T | C | C | C | A | T | A | T | T | T | A | T | T | C | T | C | A | G | - | C | G | - | - | A | T | T | C | A | A | T | T | T |
| PC-5p-67443-7 | - | - | - | - | - | - | A | T | C | T | T | C | C | - | G | C | A | A | T | T | T | C | G | A | G | A | A | G | T | C | C | C | T | T | A | T | T | T | A | T | T | C | T | G | A | G | - | C | A | - | - | A | T | T | C | A | A | T | T | G |
| PC-5p-67443-89 | - | - | - | - | - | - | A | T | C | T | T | C | C | - | G | C | A | A | T | T | T | C | G | A | G | A | A | G | T | C | C | C | T | T | A | T | T | T | A | T | T | C | T | G | A | G | - | C | A | - | - | A | T | T | C | A | A | T | T | G |
| PC-5p-67443-23 | - | - | - | - | - | - | A | T | C | T | T | C | C | - | G | C | A | A | T | T | T | C | G | A | G | G | A | G | T | C | C | C | A | T | A | T | T | T | A | T | T | C | T | C | A | G | - | C | A | - | - | A | T | T | C | A | A | T | T | A |
| PC-5p-67443-24 | - | - | - | - | - | - | A | T | C | T | T | C | C | - | G | C | A | A | T | T | T | C | G | A | G | A | A | G | T | C | C | C | A | T | A | T | T | T | A | T | T | C | T | C | A | G | - | C | G | - | - | A | T | T | C | A | A | T | T | C |
| PC-5p-67443-66 | - | - | - | - | - | - | A | T | C | T | T | C | C | - | G | C | A | A | T | T | T | C | G | A | G | A | A | G | T | C | C | C | A | T | A | T | T | T | A | T | T | C | T | C | A | G | - | C | G | - | - | A | T | T | C | A | A | T | T | G |
| PC-5p-67443-69 | - | - | - | - | - | - | A | T | C | T | T | C | C | - | G | C | A | A | T | T | T | T | G | A | G | A | A | G | T | C | C | C | A | T | A | T | T | T | A | T | T | C | T | C | A | G | - | C | G | - | - | A | T | T | C | A | A | T | - | C |
| PC-5p-67443-49 | - | - | - | - | - | - | A | T | C | T | T | C | C | - | G | C | A | A | T | T | T | C | G | A | G | A | A | G | T | C | C | C | A | T | A | T | T | T | A | T | T | C | T | C | A | G | - | C | G | - | - | A | T | T | C | A | T | A | T | A |
| PC-5p-67443-83 | - | - | - | - | - | - | A | T | C | T | T | C | C | - | G | C | A | A | T | T | T | C | G | A | G | A | A | G | T | C | C | C | A | T | A | T | T | T | A | T | T | C | T | C | A | G | - | C | G | - | - | A | T | T | C | A | T | A | T | G |
| PC-5p-67443-53 | - | - | - | - | - | - | A | T | C | T | T | C | C | - | G | C | A | A | T | T | T | T | G | A | G | A | A | G | T | C | C | C | A | T | A | T | T | T | A | T | T | C | T | C | A | G | - | C | G | - | - | A | T | C | C | A | G | T | T | A |
| PC-5p-67443-62 | - | - | - | - | - | - | A | T | C | T | T | C | C | - | G | C | A | A | T | T | T | T | G | A | G | A | A | G | T | C | C | C | A | T | A | T | T | T | A | T | T | C | T | C | A | G | - | C | G | - | - | A | T | T | C | A | A | - | - | - |
| PC-5p-67443-37 | - | - | - | - | - | - | A | T | C | T | T | C | C | - | G | C | A | A | T | T | C | C | G | A | G | A | A | G | T | C | T | C | A | T | A | T | T | T | A | T | T | C | T | C | A | G | - | C | G | - | - | A | T | T | C | A | A | T | T | G |
| PC-5p-67443-40 | - | - | - | - | - | - | A | T | C | T | A | C | C | - | G | C | A | A | T | T | T | C | G | A | A | G | A | G | T | C | C | C | A | T | A | T | T | T | A | T | T | C | T | C | A | G | - | C | G | - | - | A | T | T | C | A | T | A | T | - |
| PC-5p-67443-48 | - | - | - | - | - | - | A | T | C | T | T | C | A | T | G | C | G | T | T | A | T | C | T | A | A | A | A | A | T | G | T | T | A | G | A | T | A | A | A | T | T | A | T | T | T | T | C | C | G | - | - | T | T | T | C | G | A | A | C | G |
| PC-5p-67443-60 | - | - | - | - | - | - | A | T | C | T | T | C | C | - | G | C | A | G | T | T | T | T | G | A | G | A | A | G | T | C | C | C | A | T | A | T | T | T | A | T | T | C | T | C | A | G | - | C | G | - | - | A | T | T | C | A | T | A | T | T |
| PC-5p-67443-26 | - | - | - | - | - | - | A | T | C | T | T | C | C | - | G | C | A | A | T | T | T | C | G | A | A | A | A | G | T | C | C | C | A | T | A | T | T | T | A | T | T | T | G | G | C | A | T | A | G | - | - | G | T | G | C | A | A | T | C | C |
| PC-5p-67443-59 | - | - | - | - | - | - | A | T | C | T | T | T | C | - | G | C | A | A | T | T | T | C | G | A | A | A | A | A | T | C | C | C | A | T | A | T | T | T | A | T | T | C | T | - | C | A | G | A | G | - | - | A | T | T | C | A | T | T | T | C |
| PC-5p-67443-35 | - | - | - | - | - | - | A | T | C | T | T | T | C | - | G | C | A | A | T | T | T | G | G | A | A | A | A | A | A | C | C | C | A | T | T | T | T | T | A | T | T | C | T | - | C | A | G | C | G | - | - | A | T | A | T | A | T | A | T | A |
| PC-5p-67443-44 | - | - | - | - | - | - | A | T | C | T | T | C | C | - | G | C | A | A | T | T | T | C | G | A | G | A | A | G | T | C | C | C | A | T | A | T | T | T | A | T | T | C | T | - | C | A | G | C | G | - | - | A | T | T | C | A | T | A | T | A |
| PC-5p-67443-2 | - | - | - | - | - | - | A | T | C | T | T | C | C | - | G | C | A | A | T | T | T | C | A | A | G | A | A | G | T | C | C | C | A | T | A | T | T | T | A | T | T | C | T | - | C | A | G | C | G | - | - | A | T | T | C | A | T | T | T | G |
| PC-5p-67443-34 | - | - | - | - | - | - | A | T | T | T | T | C | C | - | G | C | A | A | T | T | T | C | G | A | G | A | A | G | T | C | C | C | A | T | A | T | T | T | A | T | T | C | T | - | C | A | G | C | G | - | - | A | T | T | C | A | T | A | T | A |
| PC-5p-67443-5 | - | - | - | - | - | - | A | T | C | T | T | C | C | - | G | C | A | A | T | T | T | C | G | A | G | A | A | G | T | C | C | C | A | T | A | T | T | T | A | T | T | C | T | - | C | A | G | C | G | - | - | A | T | T | C | A | A | T | T | G |
| PC-5p-67443-67 | - | - | - | - | - | - | A | T | C | T | T | C | C | - | G | C | A | A | T | T | T | C | G | A | G | A | A | G | T | C | C | C | A | T | A | T | T | T | A | T | T | C | T | - | C | A | G | C | G | - | - | A | T | T | C | A | T | T | T | G |
| PC-5p-67443-52 | - | - | - | - | - | - | A | T | C | T | T | C | C | - | G | C | A | G | T | T | T | T | G | A | G | A | A | G | T | C | C | C | A | T | A | T | T | T | A | T | T | C | T | - | C | A | G | C | G | - | - | A | T | T | C | A | A | T | T | G |
| PC-5p-67443-63 | - | - | - | - | - | - | A | T | C | T | T | C | C | - | G | C | A | G | T | T | T | T | G | A | G | A | A | A | T | C | C | C | A | T | A | T | T | T | A | T | T | C | T | - | C | G | G | C | G | - | - | A | T | T | C | A | A | T | T | A |
| PC-5p-67443-70 | - | - | - | - | - | - | A | T | C | T | T | C | C | - | G | C | A | G | T | T | T | T | G | A | G | A | A | G | T | C | C | C | A | T | A | T | T | T | A | T | T | C | T | - | C | A | G | C | G | - | - | A | T | C | C | A | G | T | T | G |
| PC-5p-67443-61 | - | - | - | - | - | - | A | T | C | T | T | C | C | - | G | C | A | G | T | T | T | T | G | A | G | A | A | G | T | C | C | C | A | T | A | T | T | T | A | T | T | C | T | - | C | A | G | C | G | - | - | A | T | T | C | A | T | A | T | G |
| PC-5p-67443-82 | - | - | - | - | - | - | A | T | C | T | T | C | C | - | G | C | A | A | T | T | T | C | G | A | G | A | A | G | T | C | C | C | A | T | A | T | T | T | A | T | T | T | T | - | C | A | G | C | G | - | - | A | T | T | C | A | T | A | T | A |
| PC-5p-67443-30 | - | - | - | - | - | - | A | T | C | T | T | C | C | - | G | C | A | A | T | T | T | C | G | A | G | A | A | G | T | C | C | C | A | T | A | T | T | T | A | T | T | C | T | - | C | A | G | G | G | - | - | A | T | T | C | A | G | T | T | C |
| PC-5p-67443-87 | - | - | - | - | - | - | A | T | C | T | T | C | C | - | G | C | A | A | T | T | T | C | G | A | G | A | A | G | T | C | C | C | A | T | A | T | T | T | A | T | T | C | T | - | C | A | G | C | G | - | - | A | T | T | C | A | T | A | T | T |
| PC-5p-67443-77 | - | - | - | - | - | - | A | T | C | T | T | C | C | - | G | C | A | A | T | T | T | C | G | A | A | A | A | G | T | C | C | T | A | T | A | T | T | T | A | T | T | C | T | - | C | A | G | C | G | - | - | A | T | T | C | A | T | A | T | G |
| PC-5p-67443-13 | - | - | - | - | - | - | A | T | C | T | T | C | C | - | G | C | A | A | T | T | T | C | G | A | G | A | A | G | T | C | C | C | A | T | A | T | T | T | A | T | T | C | T | - | C | A | G | C | G | - | - | A | T | T | C | A | T | A | T | A |
| PC-5p-67443-36 | - | - | - | - | - | - | A | T | C | T | T | T | C | - | G | C | A | A | T | T | T | C | G | A | A | A | A | G | T | C | C | C | A | T | A | T | T | T | A | T | T | C | T | - | C | A | G | C | G | - | - | A | T | T | C | A | T | A | T | G |
| PC-5p-67443-27 | - | - | - | - | - | - | A | T | C | T | T | C | C | - | G | C | A | A | T | T | T | C | G | A | G | A | A | T | T | C | C | C | A | T | A | T | T | T | A | T | T | C | T | - | C | A | G | C | G | - | - | A | T | T | C | A | A | A | T | A |
| PC-5p-67443-65 | - | - | - | - | - | - | A | T | C | T | T | C | C | - | G | C | A | A | T | T | T | C | G | A | G | A | A | G | T | C | C | C | A | T | C | T | T | T | A | T | T | C | T | - | C | A | G | C | G | - | - | A | T | T | C | A | T | A | T | G |
| PC-5p-67443-16 | - | - | - | - | - | - | A | T | C | T | T | C | C | - | G | C | A | A | T | T | T | C | G | A | G | A | A | G | T | T | C | C | A | T | A | T | T | T | A | T | T | C | T | - | C | A | G | C | G | - | - | T | T | T | C | A | T | A | T | A |
| PC-5p-67443-19 | - | - | - | - | - | - | A | T | C | T | T | C | C | - | G | C | A | A | T | T | T | C | G | A | A | A | A | G | T | C | T | A | A | T | A | T | T | T | A | T | T | C | T | - | C | A | G | C | A | - | - | A | T | T | C | A | T | A | T | A |
| PC-5p-67443-22 | - | - | - | - | - | - | A | T | C | T | T | C | C | - | G | C | A | A | T | T | T | G | G | A | G | A | A | G | T | C | C | C | A | T | A | T | T | T | A | T | T | C | T | - | C | A | G | C | G | - | - | A | T | T | C | A | C | A | T | G |
| PC-5p-67443-38 | - | - | - | - | - | - | A | T | T | T | T | C | C | - | G | C | A | A | T | T | T | C | G | A | A | G | A | A | T | C | C | C | A | T | A | T | T | T | A | T | T | C | T | - | C | A | G | C | G | - | - | A | T | T | C | A | T | A | T | A |
| PC-5p-67443-39 | - | - | - | - | - | - | A | T | T | T | T | C | C | - | G | C | A | A | T | T | T | C | G | A | A | G | A | A | T | C | C | C | A | T | A | T | T | T | A | T | T | C | T | - | C | A | G | C | G | - | - | A | T | T | C | A | T | A | T | A |
| PC-5p-67443-43 | - | - | - | - | - | - | A | T | C | T | T | C | C | - | G | C | A | A | T | T | T | C | G | G | A | G | A | G | T | C | C | T | A | T | A | T | T | T | A | T | T | C | T | - | A | A | G | C | G | - | - | A | T | T | C | A | T | A | T | G |
| PC-5p-67443-72 | - | - | - | - | - | - | A | T | C | T | T | C | C | - | G | C | A | A | T | T | T | C | G | A | A | A | A | G | T | C | T | C | A | T | A | T | T | T | A | T | T | C | T | - | C | A | G | C | G | - | - | A | T | T | C | A | T | T | T | A |
| PC-5p-67443-73 | - | - | - | - | - | - | A | T | C | T | T | C | C | - | G | C | A | A | T | T | T | C | G | A | A | A | A | G | T | C | C | C | A | T | A | T | T | T | C | T | T | T | T | - | A | A | G | C | G | - | - | A | T | T | C | A | A | A | T | A |
| PC-5p-67443-4 | - | - | - | - | - | - | A | G | C | T | T | T | C | - | A | C | A | A | T | T | T | C | G | A | G | A | A | G | T | C | C | C | A | T | A | T | T | T | A | T | T | C | T | - | C | A | G | T | G | - | - | A | T | T | C | A | A | T | T | A |
| PC-5p-67443-75 | - | - | - | - | - | - | A | T | C | T | T | C | C | - | G | C | A | A | T | T | T | C | G | A | G | A | A | G | T | C | C | C | A | T | A | T | T | T | A | T | T | C | T | - | C | A | G | C | A | - | - | A | T | T | C | A | A | A | T | A |
| PC-5p-67443-10 | - | - | - | - | - | - | A | T | C | T | T | C | C | - | G | C | A | A | T | T | T | C | G | A | A | A | A | G | T | C | C | C | A | T | A | T | T | T | A | T | T | A | T | - | C | A | G | C | G | - | - | A | T | T | C | A | T | A | T | G |
| PC-5p-67443-41 | - | - | - | - | - | - | A | T | C | T | T | C | C | - | G | C | A | A | T | T | T | C | G | A | G | A | A | G | T | C | C | C | A | T | A | T | T | T | A | T | T | C | T | - | C | A | G | C | G | - | - | A | T | T | C | A | A | A | T | A |
| PC-5p-67443-88 | - | - | - | - | - | - | A | T | C | T | T | T | C | - | G | C | A | A | T | T | T | C | G | A | A | A | A | G | T | C | C | C | A | T | A | T | T | T | A | T | T | C | T | - | C | A | G | T | G | - | - | A | T | T | C | T | A | A | T | T |
| PC-5p-67443-54 | - | - | - | - | - | - | A | T | C | T | T | C | C | - | G | A | A | A | T | T | T | C | G | A | A | A | A | G | T | C | C | T | A | T | A | T | T | T | A | T | T | C | T | - | C | A | G | C | G | - | - | A | T | T | C | A | A | A | T | A |
| PC-5p-67443-3 | - | - | - | - | - | - | A | T | C | T | T | C | C | - | G | C | A | A | T | T | T | C | G | A | A | A | A | G | G | A | C | C | A | T | T | T | T | C | A | T | T | C | T | G | T | G | C | C | - | - | - | A | T | T | C | A | A | T | T | G |
| PC-5p-67443-25 | - | - | - | - | - | - | A | T | C | T | T | C | C | - | G | C | A | A | T | T | T | C | G | A | A | A | A | G | G | A | C | C | G | C | T | T | T | C | A | T | T | C | T | G | T | G | C | C | - | - | - | A | T | T | C | A | T | A | T | G |
| PC-5p-67443-80 | - | - | - | - | - | - | A | T | C | T | T | C | C | - | G | C | A | T | T | T | T | C | G | A | A | A | A | G | G | A | C | C | A | T | T | T | T | C | A | T | T | C | T | G | T | G | C | C | - | - | - | A | T | T | C | A | T | A | T | T |
| PC-5p-67443-6 | - | - | - | - | - | - | A | T | C | T | T | C | C | - | G | C | A | A | T | T | T | C | G | A | A | A | A | G | G | A | C | C | G | C | T | T | T | C | A | T | T | C | T | A | T | G | C | C | - | - | - | A | T | T | C | A | T | A | T | G |
| PC-5p-67443-91 | - | - | - | - | - | - | A | T | C | T | T | C | C | - | G | C | A | A | T | T | C | C | A | A | A | A | A | G | G | A | C | C | G | C | T | T | T | C | A | T | T | C | T | G | T | G | C | C | - | - | - | A | T | T | C | A | A | A | T | G |
| PC-5p-67443-17 | - | - | - | - | - | - | A | T | C | T | T | C | C | - | G | C | A | A | T | T | T | C | G | A | A | A | A | G | G | A | C | C | G | C | T | T | T | C | A | T | T | C | T | G | T | G | C | C | - | - | - | A | T | T | C | A | T | A | T | G |
| PC-5p-67443-9 | - | - | - | - | - | - | A | T | C | T | T | C | C | - | G | C | A | A | T | T | T | C | G | A | A | A | A | G | G | A | C | C | G | C | T | T | T | C | A | T | T | C | T | G | T | A | C | T | - | - | - | A | T | T | C | A | T | A | T | G |
| PC-5p-67443-58 | - | - | - | - | - | - | A | T | C | T | T | C | C | - | G | C | A | A | T | T | T | C | G | A | A | A | A | G | G | A | C | C | A | C | T | T | T | C | A | T | T | C | T | G | T | G | C | C | - | - | - | A | T | T | C | A | T | A | T | G |
| PC-5p-67443-28 | - | - | - | - | - | - | A | T | C | T | T | C | C | - | G | C | A | A | T | T | T | C | G | A | A | A | A | G | G | A | C | C | G | C | T | T | T | C | A | T | T | C | T | G | T | G | C | C | - | - | - | A | T | T | C | A | T | A | T | G |
| PC-5p-67443-31 | - | - | - | - | - | - | A | T | C | T | T | C | C | - | G | C | A | A | T | T | T | C | G | A | A | A | A | G | G | A | C | C | G | C | T | T | T | C | A | T | T | C | T | G | T | G | C | C | - | - | - | A | T | T | C | A | T | A | T | G |
| PC-5p-67443-47 | - | - | - | - | - | - | A | T | C | T | T | C | C | - | G | C | A | A | T | T | T | C | G | A | A | A | A | G | G | A | C | C | G | C | T | T | T | C | A | T | T | C | T | G | T | G | C | C | - | - | - | A | T | T | C | A | A | A | T | G |
| PC-5p-67443-71 | - | - | - | - | - | - | A | T | C | T | T | C | C | - | G | C | A | A | T | T | T | C | G | A | A | A | A | G | G | A | C | C | G | C | T | T | T | C | A | T | T | C | T | G | T | G | C | C | - | - | - | A | T | T | C | A | A | T | T | G |
| PC-5p-67443-51 | - | - | - | - | - | - | A | T | C | T | T | C | C | - | G | C | A | A | T | T | T | C | G | A | A | A | T | G | G | A | C | C | G | C | T | T | T | C | A | T | T | C | T | G | T | T | C | C | - | - | - | A | T | T | C | A | T | A | T | A |
| PC-5p-67443-74 | - | - | - | - | - | - | A | T | C | T | T | C | C | - | G | C | A | A | T | T | T | C | G | A | A | A | A | G | G | A | C | C | A | T | T | T | T | C | A | T | T | C | T | G | T | G | C | C | - | - | - | A | T | T | C | A | A | T | T | G |
| PC-5p-67443-21 | A | T | A | A | C | G | A | T | C | T | T | C | C | - | G | C | A | A | T | T | T | G | G | A | A | A | A | A | G | A | C | C | A | T | T | T | T | C | A | T | T | C | T | G | T | G | C | C | - | - | - | A | T | T | C | A | T | A | T | G |
| PC-5p-67443-20 | - | - | - | - | - | - | A | T | C | T | T | C | C | - | G | C | A | A | T | T | T | C | G | A | A | A | A | G | G | A | C | C | G | C | T | T | T | C | A | T | T | C | T | G | T | G | C | C | - | - | - | A | T | T | C | A | T | A | T | G |
| PC-5p-67443-42 | - | - | - | - | - | - | A | T | C | T | T | C | C | - | G | C | A | A | T | T | T | C | G | A | A | A | A | G | G | A | C | C | G | C | T | T | T | C | A | T | T | C | T | G | T | G | C | C | - | - | - | A | T | T | C | A | T | A | T | A |
| PC-5p-67443-90 | - | - | - | - | - | - | A | T | C | T | T | C | C | - | G | C | A | A | T | T | C | C | A | A | A | A | A | G | G | A | C | C | G | C | T | T | T | C | A | T | T | C | T | G | T | G | C | C | - | - | - | A | T | T | C | A | T | A | T | A |
| PC-5p-67443-14 | - | - | - | - | - | - | A | T | C | T | T | C | C | - | G | C | A | A | T | T | T | C | G | A | A | A | A | G | G | A | C | C | G | C | T | T | T | C | A | T | T | C | T | G | T | G | C | C | - | - | - | A | T | T | C | A | T | T | T | G |
| PC-5p-67443-18 | - | - | - | - | - | - | A | T | C | T | T | C | C | - | G | C | A | A | T | T | T | C | G | G | - | A | A | G | G | A | C | C | A | T | T | T | T | C | A | T | T | C | T | G | T | G | C | C | - | - | - | A | T | T | C | A | T | A | T | G |
| PC-5p-67443-15 | - | - | - | - | - | - | A | T | C | T | T | C | C | - | G | C | A | A | T | T | T | C | G | A | A | A | A | G | G | A | C | C | G | C | T | T | T | C | A | T | T | C | T | G | T | G | C | C | - | - | - | A | T | T | C | A | A | T | T | C |
| PC-5p-67443-50 | - | - | - | - | - | - | A | T | C | T | T | C | C | - | G | C | A | A | T | T | T | C | G | A | A | A | A | G | G | A | C | C | G | C | T | T | T | C | A | T | T | C | T | G | T | G | C | C | - | - | - | A | T | T | C | A | A | T | T | G |
| PC-5p-67443-45 | - | - | - | - | - | - | A | T | C | T | T | C | C | - | G | C | A | A | T | T | T | C | G | A | A | A | A | G | G | A | C | C | G | C | T | T | T | C | A | T | T | C | T | G | T | G | C | C | - | - | - | A | T | T | C | A | T | T | T | G |
| PC-5p-67443-57 | - | - | - | - | - | - | A | T | C | T | T | C | C | - | G | C | A | A | T | T | T | C | G | A | A | A | A | G | G | A | C | C | G | C | T | C | T | C | A | T | T | C | T | G | T | G | C | C | - | - | - | A | T | T | C | A | A | T | T | G |
| PC-5p-67443-76 | - | - | - | - | - | - | A | T | C | T | T | C | C | - | G | C | A | A | T | T | T | C | G | A | A | A | A | G | G | A | C | C | G | C | T | T | T | C | A | T | T | C | T | G | T | G | C | C | - | - | - | A | T | T | C | A | T | T | T | A |
| PC-5p-67443-86 | - | - | - | - | - | - | A | T | C | T | T | C | C | - | G | C | A | A | T | T | T | C | G | A | A | A | A | G | G | A | C | C | G | C | T | T | T | C | A | T | T | C | T | G | T | G | C | C | - | - | - | A | T | T | C | A | A | T | T | G |
| PC-5p-67443-55 | - | - | - | - | - | - | A | T | C | T | T | C | C | - | G | C | A | A | T | T | T | C | G | A | A | A | A | G | G | A | C | C | G | C | T | T | T | C | A | T | T | C | T | G | T | G | C | C | - | - | - | A | T | T | C | A | T | A | T | G |
| PC-5p-67443-56 | - | - | - | - | - | - | A | T | C | T | T | C | C | - | G | C | A | A | T | T | T | C | G | A | A | A | A | G | G | A | C | C | G | C | T | T | T | C | A | T | T | C | T | G | T | G | C | C | - | - | - | A | T | T | C | A | T | A | T | A |
| PC-5p-67443-68 | - | - | - | - | - | - | A | T | C | T | T | C | C | - | G | C | A | A | T | T | T | C | G | A | G | A | A | G | G | A | C | C | G | C | T | T | T | C | A | T | T | C | T | G | T | G | C | C | - | - | - | A | T | T | C | A | T | A | T | G |
| PC-5p-67443-79 | - | - | - | - | - | - | A | T | C | T | T | C | C | - | G | C | A | A | T | T | T | C | G | A | A | A | A | G | G | A | C | C | A | C | T | T | T | C | A | T | T | C | T | G | T | G | C | C | - | - | - | A | T | T | C | A | T | A | T | A |
| PC-5p-67443-84 | - | - | - | - | - | - | A | T | C | T | T | C | C | - | G | C | A | A | T | T | T | C | G | A | A | A | A | G | G | A | C | C | G | C | T | T | T | C | A | T | T | C | T | G | T | G | C | C | - | - | - | A | T | T | C | A | T | A | T | A |
| PC-5p-67443-81 | - | - | - | - | - | - | A | T | C | T | T | C | C | - | G | C | A | A | T | T | T | C | G | A | A | A | A | G | G | A | C | C | G | T | T | T | T | C | A | T | T | C | T | G | T | G | C | C | - | - | - | A | T | T | C | A | T | A | T | G |
| PC-5p-67443-8 | - | - | - | - | - | - | A | T | C | T | T | C | C | - | G | C | A | A | T | T | T | C | G | A | A | A | A | G | G | A | C | C | G | C | T | T | T | T | A | T | T | C | T | G | T | G | C | C | - | - | - | A | T | T | C | A | - | A | T | A |
| PC-5p-67443-46 | - | - | - | - | - | - | A | T | C | T | T | C | C | - | G | T | A | A | T | T | T | C | G | A | A | A | A | G | G | A | C | C | A | T | T | T | T | C | A | T | T | C | T | G | T | G | C | C | - | - | - | A | T | T | C | A | T | A | T | G |
| PC-5p-67443-29 | - | - | - | - | - | - | A | T | C | T | T | C | C | - | G | C | A | A | T | T | T | C | G | A | A | A | A | G | A | A | C | C | G | C | T | T | T | C | A | T | T | C | T | G | T | G | C | C | - | - | - | A | T | T | C | A | A | A | T | G |
| PC-5p-67443-78 | - | - | - | - | - | - | A | T | C | T | T | C | C | - | G | C | A | A | T | T | T | C | G | A | A | A | A | G | G | T | C | C | A | A | T | T | T | C | A | T | T | C | T | G | T | G | C | C | - | - | - | A | T | T | C | A | A | A | T | G |
| PC-5p-67443-11 | - | - | - | - | - | - | A | T | C | T | T | C | C | - | G | C | A | A | T | T | T | C | G | A | A | A | A | G | G | A | C | C | G | - | - | - | - | C | T | T | T | G | A | G | C | G | A | A | - | - | - | - | T | T | C | A | G | A | C | C |
| PC-5p-67443-32 | - | - | - | - | - | - | A | T | C | T | T | C | C | - | G | C | A | A | T | T | A | C | G | A | A | A | A | G | C | A | C | G | C | - | - | - | - | A | A | A | A | G | T | G | C | C | A | G | - | - | - | - | C | T | C | T | A | A | C | G |
| PC-5p-67443-33 | - | - | - | - | - | - | A | T | C | T | T | C | C | - | G | C | A | A | T | T | T | C | G | A | A | A | A | G | G | A | C | C | A | T | T | T | T | C | A | A | A | G | T | C | C | C | A | A | A | A | T | A | T | T | A | C | C | G | C | C |
| PC-5p-67443-64 | - | - | - | - | - | - | A | T | C | T | T | C | C | - | G | C | A | A | T | T | T | C | G | A | A | A | A | G | G | A | C | C | A | T | T | T | T | C | A | T | T | C | T | G | T | G | C | C | - | - | - | A | T | T | C | A | A | T | T | G |
| PC-5p-67443-85 | - | - | - | - | - | - | A | T | C | T | T | C | C | - | G | C | A | A | T | T | T | C | G | A | G | A | A | G | T | C | C | C | A | T | T | T | T | T | A | T | T | C | T | A | A | G | C | G | - | - | - | A | T | T | C | A | A | T | T | G |

| PC-5p-67443-1 | G | - | - | - | - | - | - | T | A | T | T | G | A | T | A | A | C | A | A | T | C | A | C | T | A | C | A | T | A | A | G | A | T | T | - | - | - | - | - | C | G | C | A | T | G | A | T | T | T | C | T | G | G | C | A | G | T | T | T | T |
| --- | --- | --- | --- | --- | --- | --- | --- | --- | --- | --- | --- | --- | --- | --- | --- | --- | --- | --- | --- | --- | --- | --- | --- | --- | --- | --- | --- | --- | --- | --- | --- | --- | --- | --- | --- | --- | --- | --- | --- | --- | --- | --- | --- | --- | --- | --- | --- | --- | --- | --- | --- | --- | --- | --- | --- | --- | --- | --- | --- | --- |
| PC-5p-67443-12 | A | - | - | - | - | - | - | T | T | T | C | C | A | T | G | G | C | T | T | T | C | T | C | A | A | T | T | A | T | A | A | T | T | T | T | T | G | A | C | A | G | C | A | A | A | T | T | T | G | A | T | A | A | C | A | G | T | A | A | A |
| PC-5p-67443-7 | T | - | - | - | - | - | - | A | T | A | A | T | A | C | A | T | T | G | C | G | - | - | C | A | T | G | C | T | - | A | C | A | - | A | C | A | A | A | G | - | C | T | G | T | A | A | A | C | A | T | T | T | A | T | T | T | G | A | A | A |
| PC-5p-67443-89 | T | - | - | - | - | - | - | A | T | A | A | T | A | C | A | T | T | G | C | G | - | - | C | A | T | G | C | T | - | A | C | A | - | A | C | A | A | A | G | - | C | T | G | T | A | A | A | C | A | T | T | T | A | T | T | T | G | A | A | A |
| PC-5p-67443-23 | A | - | - | - | - | - | - | A | G | A | A | A | A | A | T | T | A | T | C | A | G | A | C | A | G | A | C | G | G | A | C | A | G | A | C | A | A | A | G | A | A | A | G | A | G | A | A | C | G | C | T | C | A | C | G | T | G | C | A | A |
| PC-5p-67443-24 | T | - | - | - | - | - | - | A | A | A | C | A | A | G | A | A | A | A | - | - | - | - | T | A | A | A | A | T | C | C | A | A | C | A | A | A | A | C | C | C | T | C | A | G | T | A | C | G | T | A | T | A | G | T | C | A | G | C | T | A |
| PC-5p-67443-66 | T | - | - | - | - | - | - | A | T | C | C | T | T | T | A | A | A | A | G | A | T | C | T | G | A | A | A | T | A | G | G | G | T | T | A | A | A | C | A | A | G | G | A | T | C | A | A | T | T | A | G | A | A | T | A | G | G | T | T | A |
| PC-5p-67443-69 | T | - | - | - | - | - | - | C | A | A | T | T | T | C | A | A | T | C | - | - | - | - | T | A | C | T | G | T | T | A | A | A | A | T | T | T | C | A | A | G | A | C | T | C | T | A | G | G | T | G | T | G | T | A | C | C | G | C | G | A |
| PC-5p-67443-49 | T | - | - | - | - | - | - | A | T | A | A | T | A | A | T | A | A | T | A | A | T | T | C | C | A | T | T | G | T | C | A | C | A | T | C | G | G | G | C | A | T | A | T | G | C | G | G | T | G | A | A | T | A | T | T | T | G | T | G | - |
| PC-5p-67443-83 | T | - | - | - | - | - | - | A | C | A | T | A | T | C | G | T | A | T | G | G | G | C | C | C | A | A | T | - | - | - | A | C | C | T | - | - | - | G | T | T | T | A | T | T | T | G | A | T | G | A | A | T | G | T | T | T | G | T | G | G |
| PC-5p-67443-53 | G | - | - | - | - | - | - | T | C | G | T | T | T | T | - | - | T | T | G | A | T | A | C | C | T | T | T | - | T | T | T | C | A | A | C | A | C | A | A | C | T | G | A | A | C | G | T | A | G | A | C | G | - | - | C | T | G | C | G | G |
| PC-5p-67443-62 | - | - | - | - | - | - | - | - | - | - | - | - | - | - | - | - | - | - | - | - | - | - | - | - | - | - | - | - | - | - | - | - | - | - | - | - | - | - | - | - | - | - | - | - | - | - | - | - | - | - | - | - | - | - | - | - | - | - | - | - |
| PC-5p-67443-37 | T | - | - | - | - | - | - | A | A | T | A | C | A | G | T | T | A | A | A | A | T | T | G | T | A | T | A | T | T | T | A | C | G | T | T | T | C | A | T | A | C | T | C | A | A | C | A | C | T | A | A | A | T | G | - | - | A | A | A | G |
| PC-5p-67443-40 | - | - | - | - | - | - | - | A | A | T | T | T | A | A | A | A | A | A | A | A | C | C | C | C | A | T | C | A | C | T | G | C | A | T | C | C | G | A | T | T | C | T | A | T | G | A | A | C | A | A | A | C | G | G | T | T | C | A | G | A |
| PC-5p-67443-48 | A | - | - | - | - | - | - | T | T | T | T | T | T | T | G | A | G | A | A | A | T | C | G | C | C | A | G | T | C | A | A | A | G | T | T | T | T | T | A | G | G | A | C | C | T | C | C | A | T | T | T | T | T | T | - | - | - | A | G | G |
| PC-5p-67443-60 | A | - | - | - | - | - | - | T | G | T | T | T | C | A | T | A | T | C | A | A | G | C | A | A | A | A | A | A | C | G | A | A | T | T | T | C | A | G | A | A | T | T | A | A | A | C | A | A | A | A | A | A | G | A | - | - | - | G | G | A |
| PC-5p-67443-26 | G | - | - | - | - | - | - | - | - | - | A | A | T | C | A | C | C | C | T | A | A | T | A | T | G | T | A | T | G | T | A | C | A | T | G | A | A | A | T | A | A | T | C | G | C | T | A | A | A | A | T | T | A | A | T | A | T | - | C | T |
| PC-5p-67443-59 | A | T | A | - | - | - | - | - | - | - | T | A | T | A | A | A | C | T | T | T | A | A | T | T | G | G | A | A | G | A | A | A | T | T | C | A | C | A | C | C | A | G | A | A | A | A | T | C | A | A | A | A | A | T | C | A | T | - | C | G |
| PC-5p-67443-35 | A | T | T | - | - | - | - | - | - | - | C | A | C | A | A | A | T | C | A | A | T | G | A | T | A | A | G | G | T | G | G | C | G | T | C | C | A | A | T | A | T | C | A | G | A | T | T | A | G | C | T | A | A | A | A | A | T | T | C | C |
| PC-5p-67443-44 | T | A | A | - | - | - | - | - | - | - | A | A | A | A | A | A | T | G | G | T | G | T | T | T | T | T | G | A | A | T | C | A | G | A | A | A | A | T | T | A | A | A | T | G | A | A | G | C | T | A | T | T | T | T | A | T | T | G | A | A |
| PC-5p-67443-2 | T | A | - | - | - | - | - | - | T | T | T | G | A | A | G | C | A | T | T | T | T | A | T | T | T | T | T | T | T | T | T | C | C | T | C | A | C | A | C | A | A | G | A | C | A | C | A | A | C | A | T | A | T | T | C | A | A | T | T | T |
| PC-5p-67443-34 | T | T | G | - | - | - | - | G | A | C | T | G | T | A | G | T | C | A | T | G | G | A | T | A | T | T | G | A | T | A | C | A | A | A | T | A | C | T | C | G | T | T | A | T | T | C | A | T | T | T | G | A | A | T | C | A | T | C | G | T |
| PC-5p-67443-5 | - | C | A | - | - | - | - | A | G | T | T | G | A | A | A | A | C | A | A | A | T | T | T | A | A | T | C | T | A | T | T | A | A | A | C | - | T | A | C | A | G | A | T | T | G | A | A | C | C | A | T | A | A | C | A | A | T | G | - | T |
| PC-5p-67443-67 | T | A | T | - | - | - | - | T | G | T | T | A | A | T | T | G | T | T | T | A | T | T | T | A | G | T | C | T | T | T | C | C | G | T | C | G | C | T | C | A | T | T | G | T | G | C | G | C | T | A | T | T | T | C | A | A | A | A | G | T |
| PC-5p-67443-52 | - | A | - | - | - | - | - | T | C | T | T | C | T | - | T | C | A | A | A | T | T | G | A | T | T | T | C | G | T | T | - | C | G | A | A | T | T | T | T | A | T | C | - | - | - | C | A | C | T | A | G | C | A | C | A | A | T | T | T | T |
| PC-5p-67443-63 | T | A | - | - | - | - | - | A | C | T | A | T | T | A | T | C | A | T | T | A | T | G | T | T | T | T | C | A | C | T | - | G | G | T | T | A | C | G | T | T | T | T | - | - | - | C | G | T | T | A | A | A | A | C | G | C | C | C | C | A |
| PC-5p-67443-70 | - | - | - | - | - | - | - | - | - | T | A | T | G | G | A | G | A | A | A | G | T | G | A | A | T | C | A | A | G | G | - | C | T | A | A | A | T | A | T | T | T | C | G | A | A | A | A | C | C | G | A | A | T | C | T | A | T | G | A | T |
| PC-5p-67443-61 | T | A | - | - | - | - | - | T | A | A | T | G | T | A | T | A | T | A | T | A | T | G | T | A | C | C | A | G | C | T | - | C | T | A | A | C | C | C | G | G | T | G | - | - | - | T | G | G | G | A | T | G | G | G | T | C | T | T | A | T |
| PC-5p-67443-82 | T | A | T | A | T | - | - | A | C | A | T | A | T | A | T | A | T | A | C | T | T | G | T | G | G | G | A | G | C | T | T | C | T | A | A | C | T | C | G | G | C | G | - | - | - | T | G | G | G | A | T | G | G | G | T | T | T | T | A | T |
| PC-5p-67443-30 | T | A | T | - | - | - | - | - | - | A | C | C | G | G | T | C | A | T | T | A | G | A | C | A | T | A | A | A | T | T | T | G | A | T | T | A | A | A | T | A | A | T | A | A | T | A | A | T | G | A | A | C | G | T | A | T | T | A | T | G |
| PC-5p-67443-87 | T | A | T | T | A | T | A | A | C | A | C | A | T | G | T | G | T | T | T | A | T | A | C | A | A | G | C | G | C | A | C | A | T | T | T | G | A | T | T | T | A | G | T | A | G | C | C | T | C | T | C | T | A | T | C | A | C | A | T | C |
| PC-5p-67443-77 | T | A | T | - | - | - | - | - | - | - | T | T | G | G | T | C | T | C | G | A | G | T | C | A | C | G | T | G | T | G | A | A | T | G | A | A | A | A | C | A | C | A | T | C | G | C | T | A | A | A | G | T | T | T | G | G | A | A | A | T |
| PC-5p-67443-13 | T | A | C | - | - | - | - | T | A | C | A | A | T | A | T | T | C | G | A | T | G | G | C | G | T | A | A | A | T | G | T | C | G | - | T | C | A | C | A | T | C | G | A | A | T | A | T | T | C | T | G | G | A | A | T | G | T | T | C | T |
| PC-5p-67443-36 | A | T | C | - | - | - | - | T | T | C | G | G | A | A | T | T | C | - | - | - | - | A | C | A | A | A | A | T | T | A | T | C | G | G | T | T | C | C | T | T | C | T | A | A | T | A | T | A | T | T | T | - | A | C | A | G | C | G | C | T |
| PC-5p-67443-27 | A | A | T | - | - | - | - | G | A | A | A | A | A | A | A | A | A | A | A | T | - | T | A | T | T | T | A | T | T | C | T | C | T | G | G | A | A | C | G | A | T | A | A | T | T | C | C | A | C | G | C | A | T | C | A | T | G | T | C | G |
| PC-5p-67443-65 | T | A | T | - | - | - | - | A | A | - | - | - | - | - | - | - | - | - | - | - | - | - | - | - | - | - | - | - | - | - | - | - | - | - | - | - | - | - | - | - | - | - | - | - | - | - | - | - | - | - | - | - | - | - | - | - | - | - | - | - |
| PC-5p-67443-16 | T | A | T | - | - | - | - | A | T | A | T | A | T | A | T | A | T | A | A | A | C | T | G | A | - | C | C | G | T | C | G | A | A | A | T | G | A | T | T | T | G | C | C | A | C | A | A | T | C | T | C | T | G | C | - | - | - | - | A | C |
| PC-5p-67443-19 | T | A | T | - | - | - | - | G | T | A | T | A | T | T | C | T | T | A | G | A | A | G | A | T | - | T | G | A | T | C | G | G | G | C | A | A | T | A | T | C | G | C | C | A | C | G | C | A | T | T | C | G | G | C | G | A | C | T | T | C |
| PC-5p-67443-22 | A | A | C | - | - | - | - | C | T | T | G | A | C | G | - | A | T | A | T | A | A | G | A | A | - | A | C | A | T | C | T | G | T | C | G | A | A | C | T | G | A | G | A | T | C | A | A | A | A | A | C | G | G | T | - | T | A | A | A | C |
| PC-5p-67443-38 | T | G | T | - | - | - | - | A | T | A | T | A | T | A | T | A | T | C | G | T | A | T | A | A | G | A | T | G | T | C | A | T | G | T | G | C | A | A | A | C | C | A | A | T | C | C | A | A | A | C | C | A | A | T | G | A | T | A | C | T |
| PC-5p-67443-39 | T | G | T | - | - | - | - | A | T | A | T | A | T | A | T | A | T | C | G | T | A | T | A | A | G | A | T | G | T | C | A | T | G | T | G | C | A | A | A | C | C | A | A | T | C | C | A | A | A | C | C | A | A | T | G | A | T | A | C | T |
| PC-5p-67443-43 | T | - | - | - | - | - | - | A | T | G | T | A | T | T | T | T | T | T | G | T | T | T | T | T | G | A | T | G | T | A | A | A | A | A | C | A | G | A | T | G | T | T | G | T | T | T | A | C | C | A | A | A | A | T | T | C | T | T | T | C |
| PC-5p-67443-72 | T | - | - | - | - | - | - | A | T | A | T | T | T | A | T | T | T | A | A | T | T | T | T | G | C | G | T | T | T | A | A | A | A | A | A | A | A | T | G | A | A | T | T | T | A | T | A | C | A | A | C | T | T | T | G | A | A | C | G | T |
| PC-5p-67443-73 | T | - | - | - | - | - | - | A | T | A | T | A | T | A | T | A | T | A | T | G | T | A | T | - | - | - | A | A | T | A | A | T | T | C | A | C | T | T | T | T | T | G | T | T | C | T | G | C | T | C | A | G | T | T | C | C | A | C | T | T |
| PC-5p-67443-4 | T | - | - | - | - | - | - | - | A | C | T | T | A | A | T | A | A | A | A | A | A | T | A | A | A | T | A | A | A | T | G | A | - | - | - | A | A | T | T | G | T | A | T | A | T | C | G | G | T | T | C | T | G | A | G | T | C | C | G | A |
| PC-5p-67443-75 | T | - | - | - | - | - | - | - | A | C | T | T | T | A | G | A | A | A | A | A | T | A | A | A | C | A | A | A | C | C | G | A | G | C | T | A | A | T | T | G | A | T | T | C | G | A | G | G | T | T | C | A | A | A | - | - | C | T | G | A |
| PC-5p-67443-10 | T | - | - | - | - | - | - | - | T | C | T | A | C | A | T | T | C | T | A | C | A | T | A | T | A | T | G | A | A | T | T | G | - | - | - | T | G | T | C | G | A | T | T | T | G | G | C | T | T | A | C | T | G | T | C | C | T | C | T | G |
| PC-5p-67443-41 | T | - | - | - | - | - | - | - | A | G | T | G | T | T | A | A | A | A | T | G | T | G | A | A | A | T | G | A | G | A | A | G | T | T | T | G | G | A | G | C | A | T | T | A | A | G | G | T | T | A | C | C | A | T | T | T | T | C | A | T |
| PC-5p-67443-88 | T | - | - | - | - | - | - | - | T | T | T | G | T | T | G | A | A | T | G | - | - | - | - | A | A | T | G | A | T | T | C | A | T | G | T | G | C | T | A | T | A | T | T | T | A | G | - | - | - | T | C | G | A | T | T | T | A | C | A | A |
| PC-5p-67443-54 | T | - | - | - | - | - | - | - | A | A | A | T | G | A | T | G | T | T | C | A | T | T | A | A | A | C | A | T | C | A | C | G | T | T | T | G | T | T | G | C | T | T | T | C | G | G | T | C | T | G | A | T | G | A | A | A | A | T | A | C |
| PC-5p-67443-3 | T | A | G | - | - | - | - | A | A | T | C | A | A | C | A | A | A | C | A | G | T | C | T | G | C | A | A | - | - | A | A | A | A | T | C | A | A | A | A | T | A | A | A | A | C | A | A | T | C | A | G | A | G | A | T | A | A | A | A | A |
| PC-5p-67443-25 | A | T | G | - | - | - | - | A | A | - | - | A | A | C | G | A | T | T | C | A | T | A | T | G | C | A | C | - | - | A | A | T | G | - | C | A | C | A | T | G | T | T | A | C | T | C | A | T | A | G | T | A | A | A | A | A | A | C | T | C |
| PC-5p-67443-80 | A | T | G | - | - | - | - | T | A | T | - | G | T | G | T | A | T | G | T | G | T | T | G | G | C | A | T | T | G | A | A | T | G | A | C | A | A | T | G | C | C | A | C | A | G | C | A | A | C | G | G | A | G | A | A | A | T | G | A | A |
| PC-5p-67443-6 | A | T | - | - | - | - | - | A | T | C | C | A | G | T | T | A | T | A | A | - | - | A | T | T | C | G | A | A | C | A | C | G | G | A | T | G | - | - | C | G | A | T | T | C | C | G | A | A | G | G | G | G | C | C | A | T | C | A | G | T |
| PC-5p-67443-91 | T | T | C | - | - | - | - | A | T | A | C | T | G | C | A | T | T | C | A | - | - | A | T | T | A | G | T | A | T | T | C | G | G | A | C | G | G | A | C | A | A | A | A | T | C | A | G | T | C | T | G | T | T | T | C | A | C | A | T | T |
| PC-5p-67443-17 | T | A | T | - | - | - | - | A | T | T | G | A | - | C | G | T | A | A | A | - | - | A | T | T | T | G | T | A | T | T | C | T | C | C | T | G | - | T | T | G | A | T | A | T | T | C | A | T | T | T | T | T | A | T | T | T | G | A | A | T |
| PC-5p-67443-9 | T | A | C | - | - | - | - | A | T | T | G | T | A | C | A | A | A | C | C | G | A | A | A | T | C | A | T | A | A | A | T | A | A | A | T | G | - | A | A | A | A | C | T | T | T | T | C | T | T | C | T | A | G | A | A | A | A | A | A | T |
| PC-5p-67443-58 | T | A | T | - | - | - | - | G | T | T | - | A | A | C | G | T | A | T | - | - | A | T | T | A | C | C | T | T | G | T | T | G | C | T | T | A | - | A | T | A | A | T | T | T | A | C | C | T | A | G | A | C | G | A | A | C | A | A | A | T |
| PC-5p-67443-28 | - | - | A | - | - | - | - | T | T | C | G | T | C | T | A | T | G | G | T | - | T | T | G | A | T | A | A | A | C | - | - | G | A | A | C | G | A | A | G | T | A | T | T | A | A | A | A | T | G | T | G | G | G | A | C | A | C | A | T | T |
| PC-5p-67443-31 | T | C | A | - | - | - | - | T | T | T | G | T | C | A | G | C | A | T | T | - | T | T | G | G | C | A | C | A | A | - | - | A | A | A | C | T | A | T | T | T | C | T | T | T | A | T | T | T | A | A | T | T | A | A | C | A | T | T | G | A |
| PC-5p-67443-47 | - | A | A | - | - | - | - | T | A | A | A | A | T | A | A | A | C | T | T | - | T | T | G | A | C | A | A | A | T | - | - | G | A | T | C | - | - | - | T | G | A | A | A | T | A | T | G | A | A | A | A | A | T | G | G | A | A | G | T | T |
| PC-5p-67443-71 | - | T | C | - | - | - | - | C | A | T | C | A | A | T | T | T | G | A | A | - | T | C | G | A | C | C | G | A | T | T | T | G | A | T | C | G | A | C | T | G | A | C | A | T | T | T | A | T | G | A | A | A | T | T | A | A | A | A | T | T |
| PC-5p-67443-51 | T | A | T | - | - | - | - | A | T | T | G | A | A | G | T | G | T | A | T | T | G | A | G | A | G | C | G | A | G | T | G | A | A | A | A | T | - | - | G | G | A | A | A | A | A | T | T | A | T | T | A | T | T | C | A | A | T | A | T | T |
| PC-5p-67443-74 | A | A | A | - | - | - | - | A | C | G | A | A | A | A | A | A | T | A | T | C | A | A | C | T | G | A | - | A | T | T | G | T | A | T | T | G | - | - | T | G | A | C | T | T | T | G | A | C | G | T | G | T | G | A | A | A | A | T | G | T |
| PC-5p-67443-21 | T | A | T | - | - | - | - | A | A | T | A | A | T | - | - | - | - | - | T | T | T | A | C | T | A | C | G | A | T | T | G | A | T | T | G | - | - | - | - | G | A | A | A | T | G | - | - | - | - | A | G | T | A | C | T | A | T | T | G | T |
| PC-5p-67443-20 | T | A | A | - | - | - | - | - | - | - | G | C | T | A | T | G | T | A | T | T | T | T | C | C | A | G | A | A | A | T | C | G | G | G | T | A | A | C | T | T | T | T | T | G | G | C | A | T | C | - | G | G | T | T | G | T | A | T | G | A |
| PC-5p-67443-42 | A | A | C | - | - | - | - | T | - | - | G | C | T | A | C | A | T | G | A | G | T | C | A | C | A | T | T | T | G | T | T | G | - | - | - | A | A | C | A | G | T | T | A | G | C | T | G | T | C | T | G | G | A | T | A | C | T | G | G | A |
| PC-5p-67443-90 | T | T | C | - | - | - | - | A | A | T | T | C | A | A | C | G | A | A | A | T | T | A | G | A | A | A | A | A | A | T | C | A | - | - | - | T | G | C | T | A | T | A | A | A | C | A | A | T | A | - | - | - | A | T | A | A | T | C | A | A |
| PC-5p-67443-14 | T | A | T | - | - | - | - | - | - | - | - | - | - | C | T | T | A | - | - | - | - | - | - | - | - | - | - | - | - | - | - | - | - | - | - | - | - | - | - | - | - | - | - | - | - | - | - | - | - | - | - | - | - | - | - | - | - | - | - | - |
| PC-5p-67443-18 | A | A | T | - | - | - | - | G | T | A | T | A | G | C | G | T | A | G | T | T | A | C | T | A | C | G | T | G | T | T | G | G | T | T | G | T | T | C | T | G | C | A | T | G | - | G | C | G | T | A | A | C | C | A | A | T | A | A | C | G |
| PC-5p-67443-15 | A | A | A | - | - | - | - | C | A | A | G | A | T | C | G | A | C | A | G | A | A | T | T | C | G | T | T | C | G | T | A | C | C | G | A | A | T | T | C | A | A | T | A | G | T | A | T | G | C | A | C | C | A | C | A | A | A | T | C | G |
| PC-5p-67443-50 | T | C | A | - | - | - | - | A | C | G | - | - | - | C | G | T | C | A | - | A | T | T | A | T | T | T | T | T | T | T | A | A | A | A | A | G | G | T | T | A | A | G | T | G | T | A | A | G | T | T | T | C | G | C | A | G | A | T | C | T |
| PC-5p-67443-45 | T | - | A | - | - | - | - | C | A | A | A | T | - | - | G | A | T | G | - | - | A | T | G | A | A | G | A | T | G | A | A | A | C | G | T | A | A | - | - | A | A | A | T | G | T | T | G | G | G | G | A | T | A | A | T | C | A | T | C | G |
| PC-5p-67443-57 | T | G | A | - | - | - | - | T | A | G | C | T | - | - | G | T | T | A | G | C | A | T | G | C | A | A | T | A | A | T | A | A | T | A | A | T | G | - | - | A | A | A | T | T | T | A | A | A | G | A | A | T | A | C | A | A | A | A | T | A |
| PC-5p-67443-76 | T | A | T | - | - | - | - | T | T | T | T | G | - | - | - | T | T | T | G | T | T | T | T | T | C | T | T | T | T | T | T | T | C | T | T | G | G | A | A | T | T | T | T | A | T | G | A | T | G | G | T | T | T | T | T | T | C | C | T | T |
| PC-5p-67443-86 | - | - | A | - | - | - | - | T | A | T | T | G | - | - | - | C | T | T | G | A | T | G | - | - | - | - | - | T | G | A | A | A | C | A | T | T | A | A | G | T | T | A | T | A | A | A | A | T | C | G | T | T | A | T | A | T | A | C | A | A |
| PC-5p-67443-55 | T | A | T | - | - | - | - | G | A | A | A | A | A | A | A | A | - | - | T | T | A | T | T | T | C | T | T | T | G | A | G | A | A | T | T | T | C | G | T | T | T | T | C | T | T | T | C | T | G | - | T | G | T | T | T | A | T | G | T | A |
| PC-5p-67443-56 | T | A | - | - | - | - | - | - | - | A | A | A | G | G | C | A | - | - | A | T | G | T | C | T | G | T | G | T | G | T | G | T | A | A | G | T | C | C | T | A | T | A | G | A | C | T | C | A | A | - | A | A | A | C | T | A | T | C | G | A |
| PC-5p-67443-68 | T | G | A | - | - | - | - | A | C | G | A | C | C | C | G | G | - | - | C | A | T | C | T | C | T | A | C | T | G | A | T | T | T | T | T | T | C | A | A | A | C | T | G | T | C | T | C | A | C | - | T | C | T | T | T | C | T | C | T | - |
| PC-5p-67443-79 | T | - | - | - | - | - | - | C | C | T | G | C | A | C | A | G | - | - | T | T | T | T | A | T | G | A | A | T | G | A | T | T | C | T | C | T | A | A | A | A | A | G | A | T | T | T | A | T | G | - | C | G | T | T | G | C | T | T | T | G |
| PC-5p-67443-84 | T | A | C | - | - | - | - | T | C | G | C | C | T | C | A | A | T | T | A | T | T | T | A | T | T | T | A | T | G | T | A | T | C | T | G | T | T | T | C | A | C | T | A | T | T | T | C | G | C | T | A | C | T | T | A | T | T | C | A | G |
| PC-5p-67443-81 | T | A | T | - | - | - | - | T | G | T | A | T | A | A | G | A | - | - | C | C | A | G | A | T | G | C | A | T | G | - | - | T | C | T | C | A | G | T | A | T | T | T | G | T | T | C | G | C | C | G | A | A | T | T | G | A | A | T | T | T |
| PC-5p-67443-8 | T | A | G | - | - | - | - | - | - | - | A | T | G | T | G | A | A | G | T | T | T | - | - | - | - | - | C | A | A | A | A | C | A | A | A | T | T | T | T | G | G | A | A | C | A | A | A | A | A | T | G | G | A | A | G | A | A | A | A | A |
| PC-5p-67443-46 | T | A | C | - | - | - | - | - | - | - | T | T | A | A | G | C | G | A | G | A | T | - | - | - | - | - | T | T | T | G | A | C | A | A | A | G | C | C | T | G | G | C | T | T | C | A | A | T | A | C | A | A | A | C | T | A | C | T | C | T |
| PC-5p-67443-29 | T | A | C | - | - | - | - | T | G | G | A | T | C | A | G | A | A | T | C | T | T | T | C | G | T | G | A | A | A | T | A | A | A | T | A | A | A | T | C | G | C | A | T | A | A | A | A | C | A | C | G | A | G | C | G | T | A | A | C | C |
| PC-5p-67443-78 | T | A | T | - | - | - | - | - | - | - | A | T | T | A | G | A | C | T | G | T | T | T | C | G | G | A | T | T | T | G | C | A | A | C | A | A | T | G | T | G | G | A | G | T | A | T | C | G | A | C | T | T | G | C | G | G | C | A | A | G |
| PC-5p-67443-11 | A | A | G | - | - | - | - | A | T | T | T | G | G | T | T | C | G | A | T | T | C | T | T | T | C | A | A | A | A | T | - | - | T | T | G | A | T | T | G | G | A | T | T | C | G | A | T | T | C | T | T | T | G | A | A | G | G | A | A | A |
| PC-5p-67443-32 | C | T | G | - | - | - | - | T | G | T | A | G | G | A | T | A | G | G | - | T | C | T | T | A | T | G | C | C | T | T | - | - | A | T | A | A | T | T | A | G | G | A | C | C | A | T | T | C | A | A | A | A | G | C | T | T | G | A | G | A |
| PC-5p-67443-33 | C | A | T | - | - | - | - | T | A | T | C | G | T | A | C | C | T | C | C | T | C | T | A | T | C | G | A | T | T | G | G | C | A | T | C | A | T | T | A | G | T | T | T | T | A | T | T | T | T | T | G | T | C | C | A | A | G | G | A | A |
| PC-5p-67443-64 | - | - | - | - | - | - | - | - | - | - | - | - | - | - | - | - | - | - | - | - | - | - | - | - | - | - | - | - | - | - | - | - | - | - | - | - | - | - | - | - | - | - | - | - | - | - | - | - | - | - | - | - | - | - | - | - | - | - | - | - |
| PC-5p-67443-85 | T | A | A | - | - | - | - | A | A | T | T | T | T | T | T | T | T | G | A | A | A | A | A | A | A | A | A | G | T | T | T | C | C | C | C | G | T | C | T | T | C | T | A | A | T | T | A | C | G | A | A | T | A | G | A | A | A | T | T | C |

| PC-5p-67443-1 | T | G | C | C | A | T | C | A | A | A | A | T | G | G | A | G | A | T | G | T | T | C | - | - | - | - | - |  |  |  |  |  |  |  |  |  |  |  |  |  |  |  |  |  |  |  |  |  |  |  |  |  |  |  |  |  |  |  |  |  |
| --- | --- | --- | --- | --- | --- | --- | --- | --- | --- | --- | --- | --- | --- | --- | --- | --- | --- | --- | --- | --- | --- | --- | --- | --- | --- | --- | --- | --- | --- | --- | --- | --- | --- | --- | --- | --- | --- | --- | --- | --- | --- | --- | --- | --- | --- | --- | --- | --- | --- | --- | --- | --- | --- | --- | --- | --- | --- | --- | --- | --- |
| PC-5p-67443-12 | T | T | A | A | T | T | C | T | A | T | T | T | T | C | A | G | T | - | - | - | - | - | - | - | - | - | - |  |  |  |  |  |  |  |  |  |  |  |  |  |  |  |  |  |  |  |  |  |  |  |  |  |  |  |  |  |  |  |  |  |
| PC-5p-67443-7 | T | G | C | T | T | A | C | T | A | A | A | C | C | A | T | T | A | T | T | A | T | - | - | - | - | - | - |  |  |  |  |  |  |  |  |  |  |  |  |  |  |  |  |  |  |  |  |  |  |  |  |  |  |  |  |  |  |  |  |  |
| PC-5p-67443-89 | T | G | C | T | T | A | C | T | A | A | A | C | C | A | T | T | A | T | T | A | T | - | - | - | - | - | - |  |  |  |  |  |  |  |  |  |  |  |  |  |  |  |  |  |  |  |  |  |  |  |  |  |  |  |  |  |  |  |  |  |
| PC-5p-67443-23 | T | G | C | A | A | G | C | G | T | A | C | A | C | A | G | T | A | - | - | - | - | - | - | - | - | - | - |  |  |  |  |  |  |  |  |  |  |  |  |  |  |  |  |  |  |  |  |  |  |  |  |  |  |  |  |  |  |  |  |  |
| PC-5p-67443-24 | G | G | G | C | G | G | A | T | C | A | C | A | G | A | A | G | G | T | A | A | - | - | - | - | - | - | - |  |  |  |  |  |  |  |  |  |  |  |  |  |  |  |  |  |  |  |  |  |  |  |  |  |  |  |  |  |  |  |  |  |
| PC-5p-67443-66 | T | G | A | C | T | G | A | A | A | A | T | T | G | G | A | T | - | - | - | - | - | - | - | - | - | - | - |  |  |  |  |  |  |  |  |  |  |  |  |  |  |  |  |  |  |  |  |  |  |  |  |  |  |  |  |  |  |  |  |  |
| PC-5p-67443-69 | T | G | A | G | T | T | A | T | G | A | T | T | G | G | T | A | C | A | A | A | - | - | - | - | - | - | - |  |  |  |  |  |  |  |  |  |  |  |  |  |  |  |  |  |  |  |  |  |  |  |  |  |  |  |  |  |  |  |  |  |
| PC-5p-67443-49 | - | A | A | T | T | T | T | C | A | T | T | G | - | - | - | - | T | A | T | T | C | T | - | - | - | - | - |  |  |  |  |  |  |  |  |  |  |  |  |  |  |  |  |  |  |  |  |  |  |  |  |  |  |  |  |  |  |  |  |  |
| PC-5p-67443-83 | T | A | T | G | T | G | T | C | T | T | T | G | G | T | G | C | T | A | T | T | T | C | - | - | - | - | - |  |  |  |  |  |  |  |  |  |  |  |  |  |  |  |  |  |  |  |  |  |  |  |  |  |  |  |  |  |  |  |  |  |
| PC-5p-67443-53 | T | A | A | A | C | T | A | C | G | T | T | C | T | G | - | C | T | A | C | C | A | A | - | - | - | - | - |  |  |  |  |  |  |  |  |  |  |  |  |  |  |  |  |  |  |  |  |  |  |  |  |  |  |  |  |  |  |  |  |  |
| PC-5p-67443-62 | - | - | - | - | - | - | - | - | - | - | - | - | - | - | - | - | - | - | - | - | - | - | - | - | - | - | - |  |  |  |  |  |  |  |  |  |  |  |  |  |  |  |  |  |  |  |  |  |  |  |  |  |  |  |  |  |  |  |  |  |
| PC-5p-67443-37 | A | A | C | G | A | A | T | A | A | T | C | A | A | G | A | C | T | T | - | - | - | - | - | - | - | - | - |  |  |  |  |  |  |  |  |  |  |  |  |  |  |  |  |  |  |  |  |  |  |  |  |  |  |  |  |  |  |  |  |  |
| PC-5p-67443-40 | A | A | T | G | T | C | A | A | A | A | C | T | G | A | A | C | G | A | - | - | - | - | - | - | - | - | - |  |  |  |  |  |  |  |  |  |  |  |  |  |  |  |  |  |  |  |  |  |  |  |  |  |  |  |  |  |  |  |  |  |
| PC-5p-67443-48 | A | C | C | - | C | C | C | A | G | T | C | A | A | A | T | A | T | A | - | - | - | - | - | - | - | - | - |  |  |  |  |  |  |  |  |  |  |  |  |  |  |  |  |  |  |  |  |  |  |  |  |  |  |  |  |  |  |  |  |  |
| PC-5p-67443-60 | A | A | C | G | A | T | C | A | G | A | T | A | A | A | A | A | T | A | A | - | - | - | - | - | - | - | - |  |  |  |  |  |  |  |  |  |  |  |  |  |  |  |  |  |  |  |  |  |  |  |  |  |  |  |  |  |  |  |  |  |
| PC-5p-67443-26 | G | A | A | T | T | A | A | A | T | C | T | C | A | A | A | T | A | T | A | - | - | - | - | - | - | - | - |  |  |  |  |  |  |  |  |  |  |  |  |  |  |  |  |  |  |  |  |  |  |  |  |  |  |  |  |  |  |  |  |  |
| PC-5p-67443-59 | A | G | T | T | T | A | C | A | A | A | T | C | C | A | T | A | T | G | - | - | - | - | - | - | - | - | - |  |  |  |  |  |  |  |  |  |  |  |  |  |  |  |  |  |  |  |  |  |  |  |  |  |  |  |  |  |  |  |  |  |
| PC-5p-67443-35 | G | T | T | A | T | T | G | A | T | A | A | C | G | A | A | A | A | - | - | - | - | - | - | - | - | - | - |  |  |  |  |  |  |  |  |  |  |  |  |  |  |  |  |  |  |  |  |  |  |  |  |  |  |  |  |  |  |  |  |  |
| PC-5p-67443-44 | G | G | C | G | A | T | C | A | T | A | A | G | A | C | A | A | A | - | - | - | - | - | - | - | - | - | - |  |  |  |  |  |  |  |  |  |  |  |  |  |  |  |  |  |  |  |  |  |  |  |  |  |  |  |  |  |  |  |  |  |
| PC-5p-67443-2 | G | C | T | A | A | T | T | T | A | A | T | G | T | G | A | T | - | - | - | - | - | - | - | - | - | - | - |  |  |  |  |  |  |  |  |  |  |  |  |  |  |  |  |  |  |  |  |  |  |  |  |  |  |  |  |  |  |  |  |  |
| PC-5p-67443-34 | G | C | G | A | A | C | T | T | A | T | G | - | - | A | A | T | - | - | - | - | - | - | - | - | - | - | - |  |  |  |  |  |  |  |  |  |  |  |  |  |  |  |  |  |  |  |  |  |  |  |  |  |  |  |  |  |  |  |  |  |
| PC-5p-67443-5 | C | T | T | C | A | T | T | T | T | C | C | G | C | A | T | A | T | - | - | - | - | - | - | - | - | - | - |  |  |  |  |  |  |  |  |  |  |  |  |  |  |  |  |  |  |  |  |  |  |  |  |  |  |  |  |  |  |  |  |  |
| PC-5p-67443-67 | G | T | A | C | A | A | T | - | - | - | - | G | C | T | T | A | C | T | - | - | - | - | - | - | - | - | - |  |  |  |  |  |  |  |  |  |  |  |  |  |  |  |  |  |  |  |  |  |  |  |  |  |  |  |  |  |  |  |  |  |
| PC-5p-67443-52 | T | T | T | T | T | G | G | C | G | A | T | T | T | A | A | T | C | G | - | - | - | - | - | - | - | - | - |  |  |  |  |  |  |  |  |  |  |  |  |  |  |  |  |  |  |  |  |  |  |  |  |  |  |  |  |  |  |  |  |  |
| PC-5p-67443-63 | A | G | C | T | C | A | A | C | - | - | A | C | A | C | A | T | C | A | - | - | - | - | - | - | - | - | - |  |  |  |  |  |  |  |  |  |  |  |  |  |  |  |  |  |  |  |  |  |  |  |  |  |  |  |  |  |  |  |  |  |
| PC-5p-67443-70 | T | T | C | G | T | T | C | A | A | A | T | T | T | T | T | A | C | A | G | G | - | - | - | - | - | - | - |  |  |  |  |  |  |  |  |  |  |  |  |  |  |  |  |  |  |  |  |  |  |  |  |  |  |  |  |  |  |  |  |  |
| PC-5p-67443-61 | G | C | C | C | T | G | T | A | A | T | T | T | G | G | A | C | C | A | - | - | - | - | - | - | - | - | - |  |  |  |  |  |  |  |  |  |  |  |  |  |  |  |  |  |  |  |  |  |  |  |  |  |  |  |  |  |  |  |  |  |
| PC-5p-67443-82 | G | C | C | T | T | G | T | A | A | T | A | T | G | G | A | - | - | - | - | - | - | - | - | - | - | - | - |  |  |  |  |  |  |  |  |  |  |  |  |  |  |  |  |  |  |  |  |  |  |  |  |  |  |  |  |  |  |  |  |  |
| PC-5p-67443-30 | T | G | C | G | G | A | G | G | A | T | A | T | C | A | G | C | - | - | - | - | - | - | - | - | - | - | - |  |  |  |  |  |  |  |  |  |  |  |  |  |  |  |  |  |  |  |  |  |  |  |  |  |  |  |  |  |  |  |  |  |
| PC-5p-67443-87 | T | T | C | G | A | C | T | T | A | T | - | - | - | - | - | - | - | - | - | - | - | - | - | - | - | - | - |  |  |  |  |  |  |  |  |  |  |  |  |  |  |  |  |  |  |  |  |  |  |  |  |  |  |  |  |  |  |  |  |  |
| PC-5p-67443-77 | G | T | C | T | A | A | A | T | G | T | G | T | T | C | T | T | - | - | - | - | - | - | - | - | - | - | - |  |  |  |  |  |  |  |  |  |  |  |  |  |  |  |  |  |  |  |  |  |  |  |  |  |  |  |  |  |  |  |  |  |
| PC-5p-67443-13 | A | G | A | A | T | T | T | T | C | T | A | G | A | A | T | - | - | - | - | - | - | - | - | - | - | - | - |  |  |  |  |  |  |  |  |  |  |  |  |  |  |  |  |  |  |  |  |  |  |  |  |  |  |  |  |  |  |  |  |  |
| PC-5p-67443-36 | G | G | C | G | G | T | G | A | A | T | C | G | T | A | A | G | T | C | C | - | - | - | - | - | - | - | - |  |  |  |  |  |  |  |  |  |  |  |  |  |  |  |  |  |  |  |  |  |  |  |  |  |  |  |  |  |  |  |  |  |
| PC-5p-67443-27 | A | G | C | G | G | G | A | G | T | G | G | G | A | A | A | - | - | - | - | - | - | - | - | - | - | - | - |  |  |  |  |  |  |  |  |  |  |  |  |  |  |  |  |  |  |  |  |  |  |  |  |  |  |  |  |  |  |  |  |  |
| PC-5p-67443-65 | - | - | - | - | - | - | - | - | - | - | - | - | - | - | - | - | - | - | - | - | - | - | - | - | - | - | - |  |  |  |  |  |  |  |  |  |  |  |  |  |  |  |  |  |  |  |  |  |  |  |  |  |  |  |  |  |  |  |  |  |
| PC-5p-67443-16 | G | - | T | A | A | A | T | G | C | T | T | C | - | - | A | T | C | C | A | A | A | G | - | - | - | - | - |  |  |  |  |  |  |  |  |  |  |  |  |  |  |  |  |  |  |  |  |  |  |  |  |  |  |  |  |  |  |  |  |  |
| PC-5p-67443-19 | G | A | T | A | A | A | A | A | C | A | A | C | T | T | A | T | C | C | G | A | - | - | - | - | - | - | - |  |  |  |  |  |  |  |  |  |  |  |  |  |  |  |  |  |  |  |  |  |  |  |  |  |  |  |  |  |  |  |  |  |
| PC-5p-67443-22 | G | - | T | A | C | A | T | A | T | C | T | C | A | T | C | T | C | C | - | - | - | - | - | - | - | - | - |  |  |  |  |  |  |  |  |  |  |  |  |  |  |  |  |  |  |  |  |  |  |  |  |  |  |  |  |  |  |  |  |  |
| PC-5p-67443-38 | G | A | A | C | - | - | - | A | T | A | T | G | T | A | A | A | T | - | - | - | - | - | - | - | - | - | - |  |  |  |  |  |  |  |  |  |  |  |  |  |  |  |  |  |  |  |  |  |  |  |  |  |  |  |  |  |  |  |  |  |
| PC-5p-67443-39 | G | A | A | C | - | - | - | A | T | A | T | G | T | G | G | C | G | - | - | - | - | - | - | - | - | - | - |  |  |  |  |  |  |  |  |  |  |  |  |  |  |  |  |  |  |  |  |  |  |  |  |  |  |  |  |  |  |  |  |  |
| PC-5p-67443-43 | G | A | T | T | - | - | - | C | G | A | A | T | T | G | A | A | G | G | A | - | - | - | - | - | - | - | - |  |  |  |  |  |  |  |  |  |  |  |  |  |  |  |  |  |  |  |  |  |  |  |  |  |  |  |  |  |  |  |  |  |
| PC-5p-67443-72 | A | A | T | T | - | - | - | A | T | T | T | T | T | T | G | A | T | A | A | - | - | - | - | - | - | - | - |  |  |  |  |  |  |  |  |  |  |  |  |  |  |  |  |  |  |  |  |  |  |  |  |  |  |  |  |  |  |  |  |  |
| PC-5p-67443-73 | C | G | T | T | G | A | T | A | A | A | T | T | T | T | C | A | C | T | A | - | - | - | - | - | - | - | - |  |  |  |  |  |  |  |  |  |  |  |  |  |  |  |  |  |  |  |  |  |  |  |  |  |  |  |  |  |  |  |  |  |
| PC-5p-67443-4 | A | T | A | T | G | T | A | C | T | C | A | G | A | T | G | C | T | C | A | A | - | - | - | - | - | - | - |  |  |  |  |  |  |  |  |  |  |  |  |  |  |  |  |  |  |  |  |  |  |  |  |  |  |  |  |  |  |  |  |  |
| PC-5p-67443-75 | A | A | T | T | T | T | A | T | T | A | G | C | A | T | - | C | A | A | T | A | - | - | - | - | - | - | - |  |  |  |  |  |  |  |  |  |  |  |  |  |  |  |  |  |  |  |  |  |  |  |  |  |  |  |  |  |  |  |  |  |
| PC-5p-67443-10 | A | A | A | G | T | T | A | C | T | T | A | C | T | A | T | C | G | C | T | G | - | - | - | - | - | - | - |  |  |  |  |  |  |  |  |  |  |  |  |  |  |  |  |  |  |  |  |  |  |  |  |  |  |  |  |  |  |  |  |  |
| PC-5p-67443-41 | A | T | A | T | G | T | A | T | C | A | C | A | C | T | T | T | C | - | - | - | - | - | - | - | - | - | - |  |  |  |  |  |  |  |  |  |  |  |  |  |  |  |  |  |  |  |  |  |  |  |  |  |  |  |  |  |  |  |  |  |
| PC-5p-67443-88 | G | C | T | C | A | A | G | T | T | A | T | A | - | - | T | C | C | - | - | - | - | - | - | - | - | - | - |  |  |  |  |  |  |  |  |  |  |  |  |  |  |  |  |  |  |  |  |  |  |  |  |  |  |  |  |  |  |  |  |  |
| PC-5p-67443-54 | A | A | T | T | G | C | A | T | G | A | T | G | A | A | T | G | T | A | - | - | - | - | - | - | - | - | - |  |  |  |  |  |  |  |  |  |  |  |  |  |  |  |  |  |  |  |  |  |  |  |  |  |  |  |  |  |  |  |  |  |
| PC-5p-67443-3 | T | G | A | A | G | A | T | A | T | A | A | T | G | A | A | A | A | T | - | - | - | - | - | - | - | - | - |  |  |  |  |  |  |  |  |  |  |  |  |  |  |  |  |  |  |  |  |  |  |  |  |  |  |  |  |  |  |  |  |  |
| PC-5p-67443-25 | T | T | T | A | C | A | C | G | C | A | T | C | G | A | T | A | G | T | A | - | - | - | - | - | - | - | - |  |  |  |  |  |  |  |  |  |  |  |  |  |  |  |  |  |  |  |  |  |  |  |  |  |  |  |  |  |  |  |  |  |
| PC-5p-67443-80 | T | G | T | A | A | G | C | A | C | G | A | C | A | A | C | - | - | - | - | - | - | - | - | - | - | - | - |  |  |  |  |  |  |  |  |  |  |  |  |  |  |  |  |  |  |  |  |  |  |  |  |  |  |  |  |  |  |  |  |  |
| PC-5p-67443-6 | C | A | A | A | A | G | C | C | A | A | A | A | T | T | T | C | T | T | T | - | - | - | - | - | - | - | - |  |  |  |  |  |  |  |  |  |  |  |  |  |  |  |  |  |  |  |  |  |  |  |  |  |  |  |  |  |  |  |  |  |
| PC-5p-67443-91 | C | C | A | T | G | A | C | C | A | A | C | T | G | A | T | A | - | - | - | - | - | - | - | - | - | - | - |  |  |  |  |  |  |  |  |  |  |  |  |  |  |  |  |  |  |  |  |  |  |  |  |  |  |  |  |  |  |  |  |  |
| PC-5p-67443-17 | A | T | T | G | T | A | C | G | A | A | G | A | C | C | A | T | T | T | - | - | - | - | - | - | - | - | - |  |  |  |  |  |  |  |  |  |  |  |  |  |  |  |  |  |  |  |  |  |  |  |  |  |  |  |  |  |  |  |  |  |
| PC-5p-67443-9 | A | T | A | A | A | A | A | T | A | A | G | - | - | - | C | C | G | - | - | - | - | - | - | - | - | - | - |  |  |  |  |  |  |  |  |  |  |  |  |  |  |  |  |  |  |  |  |  |  |  |  |  |  |  |  |  |  |  |  |  |
| PC-5p-67443-58 | G | G | A | G | A | A | A | C | G | A | A | T | T | T | C | C | T | C | - | - | - | - | - | - | - | - | - |  |  |  |  |  |  |  |  |  |  |  |  |  |  |  |  |  |  |  |  |  |  |  |  |  |  |  |  |  |  |  |  |  |
| PC-5p-67443-28 | T | A | T | T | A | G | A | T | T | G | A | A | G | A | T | G | A | T | C | - | - | - | - | - | - | - | - |  |  |  |  |  |  |  |  |  |  |  |  |  |  |  |  |  |  |  |  |  |  |  |  |  |  |  |  |  |  |  |  |  |
| PC-5p-67443-31 | T | G | A | A | A | G | T | T | T | G | T | T | T | T | T | T | T | - | - | - | - | - | - | - | - | - | - |  |  |  |  |  |  |  |  |  |  |  |  |  |  |  |  |  |  |  |  |  |  |  |  |  |  |  |  |  |  |  |  |  |
| PC-5p-67443-47 | T | T | T | G | T | T | G | A | A | G | A | C | A | A | A | T | A | T | A | A | T | - | - | - | - | - | - |  |  |  |  |  |  |  |  |  |  |  |  |  |  |  |  |  |  |  |  |  |  |  |  |  |  |  |  |  |  |  |  |  |
| PC-5p-67443-71 | T | G | T | G | T | G | A | A | T | G | T | C | A | T | T | A | - | - | - | - | - | - | - | - | - | - | - |  |  |  |  |  |  |  |  |  |  |  |  |  |  |  |  |  |  |  |  |  |  |  |  |  |  |  |  |  |  |  |  |  |
| PC-5p-67443-51 | T | A | A | T | T | T | A | A | A | T | A | T | T | G | A | T | - | - | - | - | - | - | - | - | - | - | - |  |  |  |  |  |  |  |  |  |  |  |  |  |  |  |  |  |  |  |  |  |  |  |  |  |  |  |  |  |  |  |  |  |
| PC-5p-67443-74 | T | C | T | T | T | T | T | T | A | T | A | T | T | T | T | T | T | - | - | - | - | - | - | - | - | - | - |  |  |  |  |  |  |  |  |  |  |  |  |  |  |  |  |  |  |  |  |  |  |  |  |  |  |  |  |  |  |  |  |  |
| PC-5p-67443-21 | A | A | - | - | - | - | - | - | - | - | - | - | - | - | - | - | - | - | - | - | - | - | - | - | - | - | - |  |  |  |  |  |  |  |  |  |  |  |  |  |  |  |  |  |  |  |  |  |  |  |  |  |  |  |  |  |  |  |  |  |
| PC-5p-67443-20 | T | G | A | T | A | C | A | A | A | A | A | A | T | A | A | A | T | T | G | - | - | - | - | - | - | - | - |  |  |  |  |  |  |  |  |  |  |  |  |  |  |  |  |  |  |  |  |  |  |  |  |  |  |  |  |  |  |  |  |  |
| PC-5p-67443-42 | T | A | T | C | A | G | A | A | G | A | G | A | G | A | A | A | A | A | A | - | - | - | - | - | - | - | - |  |  |  |  |  |  |  |  |  |  |  |  |  |  |  |  |  |  |  |  |  |  |  |  |  |  |  |  |  |  |  |  |  |
| PC-5p-67443-90 | A | A | G | T | T | C | G | T | C | C | A | T | T | A | T | A | C | A | G | T | - | - | - | - | - | - | - |  |  |  |  |  |  |  |  |  |  |  |  |  |  |  |  |  |  |  |  |  |  |  |  |  |  |  |  |  |  |  |  |  |
| PC-5p-67443-14 | - | - | - | - | - | - | - | - | - | - | - | - | - | - | - | - | - | - | - | - | - | - | - | - | - | - | - |  |  |  |  |  |  |  |  |  |  |  |  |  |  |  |  |  |  |  |  |  |  |  |  |  |  |  |  |  |  |  |  |  |
| PC-5p-67443-18 | T | T | T | C | A | C | T | C | C | C | T | T | G | T | G | A | A | - | - | - | - | - | - | - | - | - | - |  |  |  |  |  |  |  |  |  |  |  |  |  |  |  |  |  |  |  |  |  |  |  |  |  |  |  |  |  |  |  |  |  |
| PC-5p-67443-15 | T | A | T | C | A | A | A | C | - | - | - | - | T | C | G | A | A | T | - | - | - | - | - | - | - | - | - |  |  |  |  |  |  |  |  |  |  |  |  |  |  |  |  |  |  |  |  |  |  |  |  |  |  |  |  |  |  |  |  |  |
| PC-5p-67443-50 | T | T | T | C | A | A | C | C | G | A | A | A | A | C | G | A | A | T | - | - | - | - | - | - | - | - | - |  |  |  |  |  |  |  |  |  |  |  |  |  |  |  |  |  |  |  |  |  |  |  |  |  |  |  |  |  |  |  |  |  |
| PC-5p-67443-45 | A | A | T | C | C | A | G | A | - | - | A | T | A | C | G | A | T | T | A | T | T | C | A | - | - | - | - |  |  |  |  |  |  |  |  |  |  |  |  |  |  |  |  |  |  |  |  |  |  |  |  |  |  |  |  |  |  |  |  |  |
| PC-5p-67443-57 | A | A | G | C | A | A | - | - | - | - | T | G | A | C | A | A | T | T | A | T | T | C | - | - | - | - | - |  |  |  |  |  |  |  |  |  |  |  |  |  |  |  |  |  |  |  |  |  |  |  |  |  |  |  |  |  |  |  |  |  |
| PC-5p-67443-76 | T | C | C | C | C | A | A | T | C | T | A | A | C | C | A | A | A | - | - | - | - | - | - | - | - | - | - |  |  |  |  |  |  |  |  |  |  |  |  |  |  |  |  |  |  |  |  |  |  |  |  |  |  |  |  |  |  |  |  |  |
| PC-5p-67443-86 | A | C | C | A | A | T | A | T | A | T | A | T | A | T | A | T | A | T | A | T | T | C | T | T | G | T | A |  |  |  |  |  |  |  |  |  |  |  |  |  |  |  |  |  |  |  |  |  |  |  |  |  |  |  |  |  |  |  |  |  |
| PC-5p-67443-55 | A | A | T | G | C | A | T | G | A | C | A | T | G | G | C | A | C | - | - | - | - | - | - | - | - | - | - |  |  |  |  |  |  |  |  |  |  |  |  |  |  |  |  |  |  |  |  |  |  |  |  |  |  |  |  |  |  |  |  |  |
| PC-5p-67443-56 | T | C | C | A | A | C | C | T | C | G | G | T | G | G | A | A | C | A | T | T | - | - | - | - | - | - | - |  |  |  |  |  |  |  |  |  |  |  |  |  |  |  |  |  |  |  |  |  |  |  |  |  |  |  |  |  |  |  |  |  |
| PC-5p-67443-68 | - | C | T | A | A | A | T | T | T | T | A | T | T | C | C | T | G | C | A | - | - | - | - | - | - | - | - |  |  |  |  |  |  |  |  |  |  |  |  |  |  |  |  |  |  |  |  |  |  |  |  |  |  |  |  |  |  |  |  |  |
| PC-5p-67443-79 | C | A | T | T | C | C | C | G | G | A | A | A | A | A | C | C | G | A | A | - | - | - | - | - | - | - | - |  |  |  |  |  |  |  |  |  |  |  |  |  |  |  |  |  |  |  |  |  |  |  |  |  |  |  |  |  |  |  |  |  |
| PC-5p-67443-84 | A | A | T | G | A | G | T | T | T | T | G | T | G | C | A | T | T | C | T | T | - | - | - | - | - | - | - |  |  |  |  |  |  |  |  |  |  |  |  |  |  |  |  |  |  |  |  |  |  |  |  |  |  |  |  |  |  |  |  |  |
| PC-5p-67443-81 | A | A | C | T | A | C | C | T | A | A | A | A | T | T | C | T | G | A | - | - | - | - | - | - | - | - | - |  |  |  |  |  |  |  |  |  |  |  |  |  |  |  |  |  |  |  |  |  |  |  |  |  |  |  |  |  |  |  |  |  |
| PC-5p-67443-8 | A | A | T | T | T | G | G | G | A | A | A | A | A | A | A | C | A | T | T | T | G | G | A | - | - | - | - |  |  |  |  |  |  |  |  |  |  |  |  |  |  |  |  |  |  |  |  |  |  |  |  |  |  |  |  |  |  |  |  |  |
| PC-5p-67443-46 | T | G | C | A | T | G | T | G | T | A | C | A | A | A | A | A | A | G | T | T | G | A | - | - | - | - | - |  |  |  |  |  |  |  |  |  |  |  |  |  |  |  |  |  |  |  |  |  |  |  |  |  |  |  |  |  |  |  |  |  |
| PC-5p-67443-29 | A | C | T | T | C | G | T | - | A | A | A | A | G | A | A | - | - | - | - | - | - | - | - | - | - | - | - |  |  |  |  |  |  |  |  |  |  |  |  |  |  |  |  |  |  |  |  |  |  |  |  |  |  |  |  |  |  |  |  |  |
| PC-5p-67443-78 | A | T | T | T | T | T | T | G | A | A | A | A | G | T | T | C | - | - | - | - | - | - | - | - | - | - | - |  |  |  |  |  |  |  |  |  |  |  |  |  |  |  |  |  |  |  |  |  |  |  |  |  |  |  |  |  |  |  |  |  |
| PC-5p-67443-11 | T | A | T | T | T | C | G | G | C | T | A | T | G | T | G | T | G | A | T | A | T | - | - | - | - | - | - |  |  |  |  |  |  |  |  |  |  |  |  |  |  |  |  |  |  |  |  |  |  |  |  |  |  |  |  |  |  |  |  |  |
| PC-5p-67443-32 | A | A | A | T | C | C | C | C | C | G | A | T | A | C | C | A | T | T | T | T | T | T | - | - | - | - | - |  |  |  |  |  |  |  |  |  |  |  |  |  |  |  |  |  |  |  |  |  |  |  |  |  |  |  |  |  |  |  |  |  |
| PC-5p-67443-33 | A | A | A | A | T | C | A | G | C | C | A | - | - | - | - | - | - | - | - | - | - | - | - | - | - | - | - |  |  |  |  |  |  |  |  |  |  |  |  |  |  |  |  |  |  |  |  |  |  |  |  |  |  |  |  |  |  |  |  |  |
| PC-5p-67443-64 | - | - | - | - | - | - | - | - | - | - | - | - | - | - | - | - | - | - | - | - | - | - | - | - | - | - | - |  |  |  |  |  |  |  |  |  |  |  |  |  |  |  |  |  |  |  |  |  |  |  |  |  |  |  |  |  |  |  |  |  |
| PC-5p-67443-85 | C | G | T | G | G | A | T | C | T | T | G | C | T | A | - | - | - | - | - | - | - | - | - | - | - | - | - |  |  |  |  |  |  |  |  |  |  |  |  |  |  |  |  |  |  |  |  |  |  |  |  |  |  |  |  |  |  |  |  |  |

**B: The 66343 family**

| PC-5p-66343-16 | - | - | - | - | A | A | T | T | T | - | - | - | - | - | T | C | A | A | A | T | T | A | A | A | T | T | T | T | T | T | T | C | A | A | A | A | T | T | A | A | T | - | - | T | T | T | C | C | T | C | T | A | A | A | T | T | T | A | T | T |
| --- | --- | --- | --- | --- | --- | --- | --- | --- | --- | --- | --- | --- | --- | --- | --- | --- | --- | --- | --- | --- | --- | --- | --- | --- | --- | --- | --- | --- | --- | --- | --- | --- | --- | --- | --- | --- | --- | --- | --- | --- | --- | --- | --- | --- | --- | --- | --- | --- | --- | --- | --- | --- | --- | --- | --- | --- | --- | --- | --- | --- |
| PC-5p-66343-21 | - | - | C | A | A | A | A | T | T | - | - | - | - | - | T | C | C | A | A | A | A | A | A | - | - | - | - | - | T | T | T | C | A | A | A | A | T | - | - | - | - | - | - | - | T | T | C | C | A | A | A | A | T | A | T | T | T | C | A | A |
| PC-5p-66343-22 | - | - | - | - | A | G | T | T | T | G | - | - | - | A | T | A | C | G | A | C | C | T | G | T | G | G | T | G | C | T | T | C | C | C | A | T | T | G | A | A | T | T | C | G | G | T | A | C | G | A | C | C | A | A | A | T | T | C | T | G |
| PC-5p-66343-13 | - | - | - | - | - | - | G | A | T | - | - | - | - | - | T | A | C | G | A | A | T | A | A | T | A | A | - | - | - | A | T | C | G | T | G | A | A | G | T | A | A | T | C | A | A | T | T | A | A | A | A | A | T | T | C | A | A | T | T | - |
| PC-5p-66343-14 | T | T | C | T | C | G | C | A | T | - | - | - | - | - | T | T | C | G | T | T | T | A | A | T | G | - | - | - | - | A | T | A | A | T | A | G | T | G | C | G | G | A | A | A | A | G | T | G | A | G | T | T | T | T | A | A | T | C | T | - |
| PC-5p-66343-7 | - | T | C | A | A | A | A | T | T | - | - | - | - | - | T | T | T | G | G | A | A | A | A | T | T | - | - | - | - | T | T | C | A | A | A | T | T | A | A | A | T | T | T | T | T | T | T | C | A | A | A | A | T | T | A | A | T | T | T | T |
| PC-5p-66343-9 | - | T | T | C | A | A | A | A | T | - | - | - | - | T | T | C | C | A | A | A | A | A | A | A | T | - | - | - | - | T | T | C | A | A | A | G | T | T | T | C | C | A | A | A | A | A | T | T | T | T | G | A | A | A | T | T | - | T | T | T |
| PC-5p-66343-10 | - | T | T | C | A | A | A | A | T | - | - | - | - | T | T | C | C | A | A | A | A | A | A | A | T | - | - | - | - | T | T | C | A | A | A | G | T | T | T | C | C | A | A | A | A | A | T | T | T | T | G | A | A | A | T | T | - | T | T | T |
| PC-5p-66343-5 | - | - | - | - | - | - | - | - | - | - | - | - | - | - | - | - | - | - | - | - | - | - | - | - | - | - | - | - | - | - | - | - | - | - | - | - | - | - | - | - | - | - | - | - | - | - | - | - | - | - | - | - | - | - | - | - | - | - | - | - |
| PC-5p-66343-8 | - | A | T | C | A | C | A | T | T | G | T | A | T | T | T | C | C | G | A | A | A | A | A | A | A | - | - | - | - | T | T | C | C | G | A | A | T | T | T | - | - | - | - | T | T | T | T | T | C | T | G | A | A | T | T | T | - | T | T | C |
| PC-5p-66343-4 | - | A | A | G | A | A | T | A | G | - | - | - | C | A | T | T | G | G | A | T | A | G | A | T | G | - | - | - | - | G | C | G | C | T | A | G | T | T | T | C | T | A | T | G | C | G | G | T | T | T | T | A | A | A | A | A | - | A | A | G |
| PC-5p-66343-19 | - | - | - | - | A | T | A | A | T | - | - | - | - | - | T | T | T | A | A | A | A | A | A | A | T | - | - | - | - | T | T | C | A | A | A | A | T | T | T | C | A | A | A | A | G | T | T | T | C | C | A | A | T | A | A | A | G | A | A | C |
| PC-5p-66343-6 | - | - | - | - | - | T | T | T | T | - | - | - | - | C | T | C | G | A | A | A | T | A | A | T | T | T | T | T | T | T | T | C | A | A | A | A | T | T | A | A | T | T | T | T | C | T | C | A | A | A | A | T | T | A | T | T | T | T | T | T |
| PC-5p-66343-2 | - | - | - | - | A | G | C | T | T | - | - | - | - | A | T | G | C | T | A | A | A | A | A | T | C | T | - | - | C | G | T | G | A | G | A | T | T | T | T | G | T | C | A | A | A | T | A | T | T | T | A | T | T | T | T | G | T | G | A | A |
| PC-5p-66343-3 | - | - | - | - | - | - | - | - | - | - | - | - | - | - | - | A | G | T | C | A | T | A | G | A | T | - | - | - | - | T | T | G | A | A | C | T | T | T | T | G | - | - | - | A | T | C | G | C | T | A | G | A | C | C | C | T | T | T | T | T |
| PC-5p-66343-15 | - | - | - | T | T | T | T | G | G | - | - | - | - | - | A | A | A | T | T | T | T | T | T | T | T | - | - | - | - | T | G | G | A | T | A | T | T | T | T | T | - | - | - | T | T | T | C | C | A | A | A | A | A | T | T | T | T | T | T | T |
| PC-5p-66343-18 | - | - | - | - | - | T | T | G | G | - | - | - | - | - | A | C | A | A | G | T | T | T | T | T | G | - | - | - | - | A | C | C | C | A | C | T | T | G | T | A | G | A | A | T | T | T | T | T | T | T | T | T | C | A | C | C | C | A | T | T |
| PC-5p-66343-17 | - | - | - | - | G | A | A | A | T | - | - | - | - | - | T | T | T | C | G | A | A | A | T | T | T | - | - | - | - | A | T | C | A | A | A | A | T | T | T | T | C | A | C | A | A | A | T | T | T | T | C | C | A | A | A | T | G | - | T | T |
| PC-5p-66343-20 | - | - | - | - | A | T | G | A | G | - | - | - | - | - | A | A | A | C | A | A | T | G | T | A | A | - | - | - | - | A | G | C | C | A | C | A | T | T | T | T | G | A | C | T | A | G | - | - | C | A | C | G | A | T | A | T | G | G | T | T |
| PC-5p-66343-11 | - | A | A | C | G | T | T | T | G | - | - | - | - | - | A | T | T | T | T | G | A | T | T | T | A | - | - | - | - | T | T | A | A | A | T | T | T | T | T | T | C | A | A | A | T | T | T | A | A | A | A | A | A | G | C | A | G | T | T | T |
| PC-5p-66343-12 | - | - | - | - | - | - | - | - | - | - | - | - | - | - | - | - | - | T | T | G | A | T | T | T | A | - | - | - | - | T | T | A | A | A | T | T | T | T | T | T | C | A | A | A | T | T | T | A | A | A | A | A | A | G | C | A | G | T | T | T |
|  |  |  |  |  |  |  |  |  |  |  |  |  |  |  |  |  |  |  |  |  |  |  |  |  |  |  |  |  |  |  |  |  |  |  |  |  |  |  |  |  |  |  |  |  |  |  |  |  |  |  |  |  |  |  |  |  |  |  |  |  |
| PC-5p-66343-16 | T | A | T | T | T | C | G | A | A | T | C | A | A | T | T | T | T | T | T | A | A | T | T | T | - | - | A | T | T | T | T | T | C | T | C | A | A | A | A | T | T | A | A | T | T | - | - | - | - | - | - | - | - | - | - | - | T | T | T | T |
| PC-5p-66343-21 | A | A | T | T | T | C | - | - | - | - | C | A | A | T | T | T | T | T | T | A | A | T | T | T | - | - | A | T | T | T | T | T | C | T | C | A | A | A | A | T | T | A | A | T | T | - | - | - | - | - | - | - | - | - | - | - | T | T | T | T |
| PC-5p-66343-22 | T | G | T | T | T | A | - | - | - | - | - | C | T | T | G | T | T | T | T | A | A | T | T | T | - | - | A | T | T | T | T | T | C | T | C | A | A | A | A | T | T | T | A | T | T | - | - | - | - | - | - | - | - | - | - | - | T | T | T | T |
| PC-5p-66343-13 | - | - | A | C | A | A | A | T | C | A | G | G | T | T | A | T | G | T | T | C | A | C | A | T | G | G | A | A | G | A | T | T | C | T | T | T | C | A | T | T | T | T | G | A | T | G | C | - | - | - | - | - | - | - | - | - | G | T | A | A |
| PC-5p-66343-14 | - | - | T | T | A | A | A | A | C | C | G | T | T | T | A | T | T | T | T | A | A | T | T | T | - | - | A | T | T | T | T | T | C | T | C | A | A | A | A | T | T | A | A | T | T | - | - | - | - | - | - | - | - | - | - | - | T | T | T | T |
| PC-5p-66343-7 | T | T | T | C | G | A | A | T | C | A | A | T | T | T | T | T | - | - | T | A | A | T | T | T | - | - | A | T | T | T | T | T | C | T | C | A | A | A | A | T | T | A | A | T | T | - | - | - | - | - | - | - | - | - | - | - | T | T | T | T |
| PC-5p-66343-9 | T | C | T | A | A | A | T | T | T | T | A | A | A | A | T | T | T | C | C | A | A | A | A | A | - | - | - | - | A | T | T | A | T | T | T | T | T | T | C | G | A | A | A | T | T | - | - | - | - | - | - | - | - | - | - | - | - | T | T | T |
| PC-5p-66343-10 | T | C | T | A | A | A | T | T | T | T | A | A | A | A | T | T | T | C | C | A | A | A | A | A | - | - | - | - | A | T | T | A | T | T | T | T | T | T | C | G | A | A | A | T | T | - | - | - | - | - | - | - | - | - | - | - | - | T | T | T |
| PC-5p-66343-5 | - | - | - | - | - | - | - | - | - | - | - | - | - | - | - | - | - | - | - | - | - | - | - | - | - | - | - | - | - | - | - | - | - | - | - | - | - | - | - | - | - | - | - | - | - | - | - | - | - | - | - | - | - | - | - | - | - | - | - | - |
| PC-5p-66343-8 | T | T | T | T | T | A | A | T | T | T | A | T | T | T | T | T | C | T | C | A | A | A | A | T | - | - | - | - | T | A | T | T | T | T | T | T | T | T | T | T | T | T | A | A | T | - | - | - | - | - | - | - | - | - | - | - | - | T | T | T |
| PC-5p-66343-4 | G | C | A | G | A | A | A | C | C | G | G | C | A | T | T | G | C | T | C | T | T | T | A | T | - | - | - | - | T | T | T | T | C | T | C | A | A | A | A | T | T | A | A | T | T | - | - | - | - | - | - | - | - | - | - | - | - | T | T | T |
| PC-5p-66343-19 | A | A | C | A | T | T | T | T | C | A | A | A | A | C | A | G | T | T | C | A | A | A | A | T | T | C | A | T | T | T | T | C | C | T | C | T | A | A | A | T | T | A | A | T | T | - | - | - | - | - | - | - | - | - | - | - | - | T | T | T |
| PC-5p-66343-6 | T | T | T | C | A | A | A | A | T | T | A | A | T | T | T | T | T | T | T | C | G | A | G | A | G | - | - | - | - | T | A | T | T | T | T | T | T | T | T | T | T | A | A | T | T | - | - | - | - | - | - | - | - | - | - | - | - | T | T | C |
| PC-5p-66343-2 | A | T | A | G | G | C | C | A | C | T | G | G | T | T | T | G | T | T | G | C | A | C | A | G | - | - | - | - | - | T | T | T | C | A | C | T | A | A | T | T | A | T | A | T | T | - | - | - | - | - | - | - | - | - | - | - | - | - | A | T |
| PC-5p-66343-3 | T | G | A | T | T | T | T | T | T | C | T | T | T | T | T | G | A | A | A | - | - | - | - | - | - | - | - | - | A | C | T | T | T | T | T | T | T | T | T | T | T | A | A | T | T | T | G | T | T | T | T | C | T | T | T | A | A | A | A | A |
| PC-5p-66343-15 | T | G | A | A | A | T | T | T | T | T | T | T | T | T | T | A | A | A | T | - | - | - | - | - | - | - | - | - | T | T | A | A | A | A | A | T | T | T | T | T | T | A | A | A | A | A | - | - | - | - | - | C | T | T | T | A | G | - | A | A |
| PC-5p-66343-18 | G | G | A | A | T | T | T | T | T | T | T | T | T | T | C | A | C | C | T | - | - | - | - | - | - | - | - | - | A | T | T | G | G | A | A | T | T | T | T | T | T | T | T | T | T | G | - | - | - | - | - | - | - | - | A | A | A | C | T | T |
| PC-5p-66343-17 | G | G | A | A | A | A | T | T | T | T | C | C | T | T | A | C | T | T | T | - | - | - | - | - | - | - | - | - | C | T | T | T | T | T | T | T | G | A | G | A | T | A | T | T | T | T | - | - | - | - | - | - | T | T | T | C | T | - | A | C |
| PC-5p-66343-20 | C | G | A | A | A | A | T | T | T | T | C | C | T | A | A | A | T | T | T | - | - | - | - | - | - | - | - | - | C | T | T | T | T | T | T | T | G | A | A | T | A | A | T | T | T | T | - | - | - | - | - | - | T | T | T | C | C | C | A | C |
| PC-5p-66343-11 | T | T | T | T | T | A | T | T | T | T | A | T | T | T | C | - | - | - | - | - | - | - | - | - | - | - | - | - | - | G | C | T | C | A | A | T | G | A | T | T | A | C | T | T | T | T | G | - | - | - | - | - | - | - | G | A | T | C | T | A |
| PC-5p-66343-12 | T | T | T | T | T | A | T | T | T | T | A | T | T | T | C | T | T | T | T | G | G | C | T | T | C | - | - | - | C | G | C | T | C | A | A | T | G | A | T | T | A | C | T | T | T | T | G | - | - | - | - | - | - | - | G | A | T | C | T | A |

**├miRNA starts**

| PC-5p-66343-16 | T | T | - | G | A | A | T | T | A | T | - | T | A | A | T | T | T | T | T | T | G | A | A | G | T | G | A | - | A | A | A | A | T | A | A | T | T | A | A | T | T | T | - | - | - | - | T | T | A | A | A | T | A | A | G | A | G | T | T | T |
| --- | --- | --- | --- | --- | --- | --- | --- | --- | --- | --- | --- | --- | --- | --- | --- | --- | --- | --- | --- | --- | --- | --- | --- | --- | --- | --- | --- | --- | --- | --- | --- | --- | --- | --- | --- | --- | --- | --- | --- | --- | --- | --- | --- | --- | --- | --- | --- | --- | --- | --- | --- | --- | --- | --- | --- | --- | --- | --- | --- | --- |
| PC-5p-66343-21 | T | T | - | G | A | A | T | T | A | T | - | T | A | A | T | T | T | T | T | T | G | A | A | G | T | G | A | - | A | A | A | A | T | A | A | T | A | A | A | A | T | T | - | - | - | - | T | T | A | A | A | T | A | A | G | A | G | T | T | C |
| PC-5p-66343-22 | T | T | C | G | A | A | T | T | A | T | - | T | A | A | T | T | T | T | T | T | G | A | A | T | T | G | A | - | A | A | A | A | T | A | A | T | T | A | A | T | T | T | - | - | - | - | T | T | A | A | A | T | A | A | G | A | G | T | T | C |
| PC-5p-66343-13 | T | T | C | G | T | A | T | T | C | T | G | T | C | A | A | G | T | G | A | A | C | A | T | A | A | C | T | - | T | A | A | A | T | A | A | T | T | A | A | A | T | T | - | - | - | - | T | T | A | A | A | T | A | A | G | A | G | T | T | T |
| PC-5p-66343-14 | T | T | C | G | A | A | T | T | T | T | - | T | A | A | T | T | T | T | T | T | G | A | A | G | T | G | A | - | T | A | A | A | T | A | A | T | T | A | A | A | T | T | - | - | - | - | T | T | A | A | A | T | A | A | G | A | G | T | T | T |
| PC-5p-66343-7 | T | T | C | G | A | A | T | T | T | T | - | T | A | A | T | T | T | T | T | T | G | A | A | G | T | G | G | - | T | A | A | A | T | A | A | T | T | A | A | A | T | T | - | - | - | - | T | T | A | A | A | T | A | A | G | A | G | T | T | T |
| PC-5p-66343-9 | T | T | C | G | A | A | C | T | A | T | T | A | A | T | T | T | T | T | T | T | G | A | A | G | T | G | A | - | A | A | A | A | T | A | A | T | T | A | A | A | T | T | - | - | - | - | T | G | A | A | G | T | A | A | G | A | G | T | T | T |
| PC-5p-66343-10 | T | T | C | G | A | A | C | T | A | T | T | A | A | T | T | T | T | T | T | T | G | A | A | G | T | G | A | - | A | A | A | A | T | A | A | T | T | A | A | A | T | T | - | - | - | - | T | G | A | A | G | T | A | A | G | A | G | T | T | T |
| PC-5p-66343-5 | - | - | - | - | - | - | - | - | - | - | - | - | - | - | - | - | - | - | - | - | - | A | A | G | T | G | A | - | A | A | A | A | T | A | A | T | T | A | A | A | T | T | - | - | - | - | T | T | A | A | A | T | A | A | G | A | G | T | T | T |
| PC-5p-66343-8 | T | T | C | G | A | A | T | C | A | T | T | A | A | T | T | C | A | T | T | G | A | A | A | T | G | T | C | - | A | A | A | A | T | A | A | T | T | A | A | A | T | T | - | - | - | - | G | T | A | A | A | T | A | A | G | A | G | T | T | T |
| PC-5p-66343-4 | T | T | C | G | A | A | T | T | A | T | T | A | A | T | T | T | T | T | T | G | - | A | A | G | T | G | A | - | A | A | T | A | T | A | A | T | T | A | A | A | T | T | - | - | - | - | T | T | A | A | A | T | A | A | G | A | G | T | T | T |
| PC-5p-66343-19 | T | T | C | G | A | A | T | T | A | T | T | A | A | T | T | T | T | T | C | G | - | A | A | G | T | G | A | - | A | A | A | A | T | A | A | T | T | A | A | A | T | T | - | - | - | - | T | T | A | A | A | T | A | A | G | A | G | T | T | T |
| PC-5p-66343-6 | T | T | T | T | C | A | A | A | A | T | T | A | A | A | T | T | T | T | T | G | A | A | G | T | G | A | C | - | A | A | A | A | T | A | G | T | T | A | A | T | T | T | - | - | - | - | G | T | A | A | A | T | A | A | G | A | G | T | T | T |
| PC-5p-66343-2 | G | T | A | T | C | T | T | T | C | T | G | A | A | T | A | T | T | T | T | C | G | A | A | G | T | G | A | C | A | A | A | A | T | A | A | T | T | A | A | A | T | T | - | - | - | - | T | T | A | A | A | T | A | A | G | A | G | T | T | T |
| PC-5p-66343-3 | A | A | A | T | T | T | T | T | G | T | A | A | A | T | T | T | T | T | T | T | T | T | A | T | T | G | G | C | A | A | T | C | G | A | C | T | G | G | A | A | T | T | G | G | T | C | T | A | A | A | A | T | A | A | G | A | G | T | T | T |
| PC-5p-66343-15 | C | T | T | T | T | T | T | T | A | T | G | A | A | T | T | T | T | T | T | T | T | T | G | - | C | C | C | C | A | A | T | C | G | A | C | T | G | G | A | A | T | T | G | G | T | C | T | A | A | A | A | T | A | A | G | A | G | T | T | T |
| PC-5p-66343-18 | T | T | T | T | T | T | T | C | G | A | A | A | T | T | T | T | T | T | T | T | T | T | T | G | C | G | C | C | A | A | T | C | G | A | C | T | G | G | A | A | T | T | G | G | T | C | T | A | A | A | A | T | A | A | G | A | G | T | T | T |
| PC-5p-66343-17 | C | A | A | T | C | G | A | T | G | G | G | A | A | A | T | C | A | T | C | T | G | A | A | A | C | T | T | A | T | A | G | A | - | A | A | T | A | C | A | T | A | T | A | T | A | C | G | C | A | A | A | T | A | A | G | A | G | T | T | T |
| PC-5p-66343-20 | C | A | A | T | C | G | A | T | A | G | A | A | A | A | T | C | A | T | C | T | G | A | A | A | C | T | T | A | T | A | G | A | - | A | A | T | A | C | A | T | A | T | A | T | A | C | G | T | A | A | A | T | A | A | G | A | G | T | T | T |
| PC-5p-66343-11 | A | A | A | G | T | T | T | T | A | G | A | T | G | A | G | C | A | T | T | T | T | T | G | A | A | G | G | G | A | A | A | A | A | T | A | G | T | A | A | T | T | T | C | A | A | T | T | T | T | A | A | T | A | A | G | A | G | T | T | T |
| PC-5p-66343-12 | A | A | A | G | T | T | T | T | A | G | A | T | G | A | G | C | A | T | T | T | T | T | G | A | A | G | G | G | A | A | A | A | A | T | A | G | T | A | A | T | T | T | C | A | T | T | T | T | T | A | A | T | A | A | G | A | G | T | T | T |

**Complementary Complementary**

**miRNA ends┤ ├sequence starts ┤ sequence ends**

| PC-5p-66343-16 | G | A | T | A | C | G | A | T | C | G | A | C | G | G | T | G | C | A | T | T | T | T | C | G | T | A | A | A | T | T | C | G | - | - | - | - | - | - | - | G | T | A | C | G | A | T | C | G | A | A | T | T | C | T | G | T | T | T | G | T |
| --- | --- | --- | --- | --- | --- | --- | --- | --- | --- | --- | --- | --- | --- | --- | --- | --- | --- | --- | --- | --- | --- | --- | --- | --- | --- | --- | --- | --- | --- | --- | --- | --- | --- | --- | --- | --- | --- | --- | --- | --- | --- | --- | --- | --- | --- | --- | --- | --- | --- | --- | --- | --- | --- | --- | --- | --- | --- | --- | --- | --- |
| PC-5p-66343-21 | G | A | T | A | C | G | A | C | C | G | A | C | G | G | T | G | C | A | T | T | T | T | C | G | T | G | A | A | T | T | C | G | G | T | A | T | T | C | G | G | T | A | C | G | A | T | C | G | A | A | T | T | C | T | G | T | T | T | G | T |
| PC-5p-66343-22 | G | A | T | A | C | G | A | C | C | G | A | C | G | G | T | G | C | A | T | C | T | T | A | G | T | G | A | A | T | T | C | G | - | - | - | - | - | - | - | G | T | A | C | C | A | T | C | G | A | A | T | T | C | T | G | T | T | C | G | T |
| PC-5p-66343-13 | G | A | T | A | C | G | A | C | T | G | A | C | G | G | T | G | C | A | T | T | T | T | C | G | T | G | A | A | T | T | C | G | - | - | - | - | - | - | - | G | T | A | C | G | A | T | C | G | A | A | T | T | C | T | G | T | T | T | G | T |
| PC-5p-66343-14 | G | A | T | A | C | G | A | C | T | G | A | C | G | G | T | G | C | A | T | T | T | T | C | G | T | G | A | A | T | T | C | G | - | - | - | - | - | - | - | G | T | A | C | G | A | T | C | G | A | A | T | T | C | T | G | T | T | T | G | T |
| PC-5p-66343-7 | G | A | T | A | C | G | A | C | C | G | A | C | G | G | T | G | C | A | T | T | T | T | C | G | T | G | A | A | T | T | C | G | - | - | - | - | - | - | - | G | T | A | C | G | A | T | C | G | A | A | T | T | C | T | G | T | T | T | G | T |
| PC-5p-66343-9 | G | A | T | A | C | G | A | C | C | G | A | C | G | G | T | G | C | A | C | T | - | - | C | T | T | G | A | A | T | T | C | G | - | - | - | - | - | - | - | G | T | A | C | G | A | T | C | G | A | T | T | T | C | T | G | T | T | C | G | T |
| PC-5p-66343-10 | G | A | T | A | C | G | A | C | C | G | A | C | G | G | T | G | C | A | C | T | - | - | C | T | T | G | A | A | T | T | C | G | - | - | - | - | - | - | - | G | T | A | C | G | A | T | C | G | A | T | T | T | C | T | G | T | T | C | G | T |
| PC-5p-66343-5 | G | A | T | A | C | G | A | C | C | G | A | C | G | G | T | G | C | A | T | T | T | T | C | G | T | G | A | A | T | T | C | G | - | - | - | - | - | - | - | G | T | A | C | G | A | T | C | G | A | A | T | T | C | T | G | T | T | T | G | T |
| PC-5p-66343-8 | G | A | T | A | C | G | A | C | C | G | A | C | G | G | T | G | C | A | T | C | T | T | T | G | T | G | A | A | T | T | C | G | - | - | - | - | - | - | - | G | T | A | C | G | A | T | C | G | A | A | T | T | C | G | T | T | A | C | G | T |
| PC-5p-66343-4 | G | A | T | A | C | G | A | C | C | G | A | C | G | G | T | G | C | A | T | C | T | T | C | G | T | G | A | A | T | T | C | G | - | - | - | - | - | - | - | G | T | A | C | G | A | T | C | G | G | A | T | T | T | T | G | T | T | C | G | T |
| PC-5p-66343-19 | G | A | T | A | C | G | A | C | C | G | A | C | A | G | T | G | C | A | T | C | T | T | C | G | T | G | A | A | T | T | C | G | - | - | - | - | - | - | - | G | T | A | C | G | A | T | C | G | A | A | T | T | C | T | G | T | T | - | G | T |
| PC-5p-66343-6 | G | A | T | A | C | G | A | C | C | G | A | C | G | G | T | G | C | A | T | C | T | T | C | G | T | G | A | A | T | T | C | G | - | - | - | - | - | - | - | G | T | A | C | G | A | T | C | T | C | A | T | T | C | T | G | T | A | C | A | C |
| PC-5p-66343-2 | G | A | T | A | T | G | A | C | C | G | A | C | G | G | T | G | C | G | T | C | T | T | C | G | T | G | A | T | T | T | C | G | - | - | - | - | - | - | - | G | T | A | C | A | A | T | C | G | A | A | T | T | C | T | G | T | A | C | A | T |
| PC-5p-66343-3 | G | A | T | A | T | A | A | C | C | G | A | C | G | G | T | G | C | A | T | C | C | T | C | G | T | G | A | A | C | T | C | G | - | - | - | - | - | - | - | G | T | A | C | G | A | T | C | G | A | A | T | T | C | T | G | T | A | C | A | T |
| PC-5p-66343-15 | G | A | T | A | C | A | A | C | C | G | A | C | G | G | T | G | C | A | T | C | C | T | C | G | T | G | T | A | T | T | C | G | - | - | - | - | - | - | - | G | T | A | C | G | A | T | C | G | A | A | T | T | C | T | G | T | A | C | A | T |
| PC-5p-66343-18 | G | A | T | A | C | A | A | C | C | G | A | C | G | G | T | G | C | A | T | C | C | T | C | G | T | G | A | A | T | T | C | G | - | - | - | - | - | - | - | G | T | A | C | G | A | T | C | G | A | A | T | T | C | T | G | T | A | C | A | T |
| PC-5p-66343-17 | G | A | T | A | C | G | A | T | C | G | A | C | G | G | T | G | C | A | T | C | C | T | C | G | T | G | A | A | T | T | C | G | - | - | - | - | - | - | - | G | T | A | C | G | A | T | C | G | A | A | T | T | C | T | G | T | A | C | A | T |
| PC-5p-66343-20 | G | A | T | A | C | A | A | C | C | G | A | C | G | G | T | G | C | A | T | C | C | T | C | G | T | G | A | A | T | T | C | G | - | - | - | - | - | - | - | G | T | A | C | G | A | T | C | G | G | A | T | T | C | T | G | T | A | C | A | T |
| PC-5p-66343-11 | G | A | T | A | C | A | A | C | C | G | A | C | G | G | T | G | C | A | T | C | C | T | C | G | T | G | A | A | T | T | C | G | - | - | - | - | - | - | - | G | T | A | C | G | A | T | C | G | A | A | T | T | C | T | G | T | A | C | A | T |
| PC-5p-66343-12 | G | A | T | A | C | A | A | C | C | G | A | C | G | G | T | G | C | A | T | C | C | T | C | G | T | G | A | A | T | T | C | G | - | - | - | - | - | - | - | G | T | A | C | A | A | T | C | G | A | A | T | T | C | T | G | T | A | C | A | T |

| PC-5p-66343-16 | A | C | T | T | G | T | T | T | T | - | - | - | - | - | - | - | - | A | C | G | C | G | T | T | - | - | - | T | T | A | G | G | C | G | A | T | T | T | C | C | A | A | T | C | G | A | T | T | - | C | G | G | G | A | G | A | A | A | T | A |
| --- | --- | --- | --- | --- | --- | --- | --- | --- | --- | --- | --- | --- | --- | --- | --- | --- | --- | --- | --- | --- | --- | --- | --- | --- | --- | --- | --- | --- | --- | --- | --- | --- | --- | --- | --- | --- | --- | --- | --- | --- | --- | --- | --- | --- | --- | --- | --- | --- | --- | --- | --- | --- | --- | --- | --- | --- | --- | --- | --- | --- |
| PC-5p-66343-21 | A | C | T | T | G | T | T | T | T | T | T | C | C | A | A | A | A | A | T | T | A | G | T | T | - | - | - | T | T | T | C | G | C | G | T | T | T | T | C | C | - | - | T | C | G | - | - | - | - | C | G | A | G | G | G | T | A | A | A | C |
| PC-5p-66343-22 | A | C | T | T | G | T | T | T | T | T | - | C | A | T | T | A | A | A | A | T | A | G | T | C | - | - | - | T | T | C | T | G | A | A | A | A | A | A | C | T | T | - | T | C | A | T | T | A | - | A | A | T | A | C | G | G | C | A | A | C |
| PC-5p-66343-13 | A | C | T | T | G | T | T | T | T | T | - | - | - | - | A | T | C | A | T | C | T | G | T | C | - | - | - | T | C | C | T | T | C | T | A | A | C | A | C | T | A | T | T | T | T | T | G | A | - | G | A | A | T | C | A | T | T | T | T | A |
| PC-5p-66343-14 | A | C | T | T | G | T | T | T | T | T | - | - | - | - | A | T | C | A | T | C | T | G | T | C | - | - | - | T | C | C | T | T | C | T | A | A | C | A | C | T | A | T | T | T | T | T | G | A | - | G | A | A | T | C | A | T | T | T | T | A |
| PC-5p-66343-7 | A | C | T | T | G | T | T | T | T | T | - | - | - | - | A | T | C | A | T | C | T | G | T | C | - | - | - | T | C | C | T | T | C | T | A | A | C | A | C | T | A | T | T | T | C | T | G | T | - | C | A | C | T | T | C | T | A | C | T | - |
| PC-5p-66343-9 | A | C | T | T | G | - | T | T | T | C | T | C | T | G | T | T | T | A | T | T | T | A | C | - | - | - | - | - | A | G | T | T | C | G | A | G | A | A | A | G | A | A | C | C | A | T | T | G | C | A | A | C | G | T | A | T | A | A | T | T |
| PC-5p-66343-10 | A | C | T | T | G | - | T | T | T | C | T | C | T | G | T | T | T | A | T | T | T | A | C | - | - | - | - | - | A | G | T | T | C | G | A | G | A | A | A | G | A | A | C | C | A | T | T | G | C | A | A | C | G | T | A | T | A | A | T | T |
| PC-5p-66343-5 | A | C | T | T | G | T | T | T | T | T | T | C | T | T | C | A | T | T | T | T | T | C | C | C | A | C | C | T | A | A | T | T | C | A | A | A | A | A | T | T | A | A | A | C | A | T | T | T | T | T | A | T | G | - | A | T | T | T | T | T |
| PC-5p-66343-8 | A | C | T | T | G | - | T | T | T | T | T | A | A | A | T | T | T | T | T | T | T | A | C | C | - | - | - | - | C | C | T | T | G | G | A | C | A | A | A | T | T | T | T | T | G | A | C | G | - | A | G | C | T | T | G | T | A | G | T | G |
| PC-5p-66343-4 | A | C | T | T | G | - | T | T | T | T | T | G | A | G | T | C | A | A | A | T | C | G | A | A | - | - | - | - | A | C | G | T | C | A | A | T | C | C | A | T | A | T | T | A | G | T | C | T | C | C | A | A | T | C | G | T | G | G | A | G |
| PC-5p-66343-19 | A | C | T | T | G | T | T | T | T | T | T | C | T | G | T | C | A | T | A | A | A | T | T | G | - | - | - | A | A | C | T | T | C | G | A | - | A | A | A | T | A | A | A | A | G | A | A | A | A | A | G | C | T | - | A | A | G | A | A | A |
| PC-5p-66343-6 | A | C | T | T | G | T | T | T | A | T | T | T | C | T | A | T | A | C | T | G | T | C | T | T | G | - | - | T | T | C | T | T | G | G | T | T | G | A | G | T | G | - | T | C | A | T | T | T | - | - | - | C | A | A | T | A | A | A | G | T |
| PC-5p-66343-2 | A | C | T | C | C | T | T | A | A | T | - | - | - | - | - | - | G | A | G | A | A | G | T | T | - | - | - | C | T | C | A | T | C | A | A | A | A | T | C | A | A | A | A | A | A | A | A | A | G | A | A | A | A | A | A | A | A | T | G | T |
| PC-5p-66343-3 | A | - | - | - | - | - | - | - | - | - | - | - | - | - | - | - | - | - | - | - | - | - | - | - | - | - | - | - | - | - | - | - | - | - | - | - | - | - | - | - | - | - | - | - | - | - | - | - | - | - | - | - | - | - | - | - | - | - | - | - |
| PC-5p-66343-15 | A | C | T | T | G | T | T | T | T | T | T | A | C | T | A | A | T | T | - | C | G | T | G | C | C | T | - | A | A | A | T | A | G | T | T | A | A | A | C | C | G | A | G | A | G | C | A | G | A | A | G | A | G | - | - | - | - | T | T | G |
| PC-5p-66343-18 | A | C | T | T | G | T | T | T | A | A | G | G | T | T | A | T | T | T | T | T | G | T | T | A | A | A | - | T | A | A | A | A | T | T | T | G | A | A | C | T | G | C | G | A | A | A | A | G | T | A | C | T | T | - | - | - | - | T | T | T |
| PC-5p-66343-17 | A | C | T | T | G | T | T | T | T | C | T | T | T | C | C | A | A | - | - | T | A | C | A | T | C | T | - | A | C | A | G | C | A | C | T | G | C | A | G | A | A | G | G | A | C | T | G | T | A | A | G | T | C | - | - | - | - | C | A | T |
| PC-5p-66343-20 | A | C | T | T | G | T | T | T | T | A | T | A | T | T | C | A | A | A | A | T | A | A | T | T | T | T | - | C | C | A | A | A | A | T | T | T | T | T | T | T | T | G | T | T | C | G | G | A | A | A | A | T | T | - | - | - | - | C | T | G |
| PC-5p-66343-11 | A | C | T | T | G | T | T | T | C | A | A | A | T | T | C | G | T | C | C | T | G | T | T | C | A | T | - | T | A | T | T | G | A | T | G | C | G | G | A | G | T | A | T | G | A | A | A | A | A | A | G | T | C | G | A | G | A | A | T | T |
| PC-5p-66343-12 | A | C | T | T | G | T | T | T | T | T | A | T | T | T | G | - | - | - | - | T | A | T | T | C | A | A | - | A | A | C | A | C | T | T | G | A | A | T | A | - | T | G | T | G | A | C | A | C | A | A | G | T | T | - | - | A | T | C | T | T |
|  |  |  |  |  |  |  |  |  |  |  |  |  |  |  |  |  |  |  |  |  |  |  |  |  |  |  |  |  |  |  |  |  |  |  |  |  |  |  |  |  |  |  |  |  |  |  |  |  |  |  |  |  |  |  |  |  |  |  |  |  |
| PC-5p-66343-16 | A | A | A | A | A | T | T | T | T | A | A | A | A | A | C | T | T | C | A | A | A | A | A | T | T | T | T | C | A | A | A | T | T | T | T | T | T | T | T | C | T | T | C | T | G | A | A | A | A | A | A | T | G | C | A | A |  |  |  |  |
| PC-5p-66343-21 | C | A | A | G | A | C | T | T | T | T | C | T | C | G | C | A | T | C | T | C | A | A | A | T | T | A | T | G | G | - | - | - | - | - | - | - | - | - | T | C | T | G | C | G | G | A | T | A | A | G | T | A | A | A | A | - |  |  |  |  |
| PC-5p-66343-22 | C | A | A | A | T | T | T | A | T | T | A | T | A | G | A | A | A | T | T | G | T | T | C | A | T | C | A | A | A | A | T | T | - | - | - | - | - | - | A | G | T | T | T | A | A | A | C | C | A | T | A | A | A | C | G | G |  |  |  |  |
| PC-5p-66343-13 | A | T | C | A | A | A | G | T | A | A | T | C | G | A | T | T | A | C | G | A | T | T | A | C | G | A | A | T | A | A | T | A | A | A | T | C | G | - | T | G | A | A | G | T | A | A | T | C | A | A | T | T | A | - | - | - |  |  |  |  |
| PC-5p-66343-14 | A | T | C | A | A | A | G | T | A | A | T | C | G | A | T | T | A | C | G | A | T | T | A | C | G | A | A | T | A | A | T | A | A | A | T | C | G | - | T | G | A | A | G | T | A | A | T | C | A | A | T | T | A | - | - | - |  |  |  |  |
| PC-5p-66343-7 | C | T | G | A | A | A | A | T | C | A | A | C | T | T | G | G | T | C | T | C | T | A | T | C | G | A | T | T | C | T | T | - | - | C | T | C | G | A | T | G | T | C | C | A | A | T | T | C | A | T | G | A | A | - | - | - |  |  |  |  |
| PC-5p-66343-9 | G | T | T | T | A | T | T | A | G | C | T | T | G | G | T | T | T | A | A | T | T | T | G | T | T | G | G | A | T | T | T | T | G | T | T | C | A | G | C | A | A | T | G | T | G | C | G | C | A | A | A | A | - | - | - | - |  |  |  |  |
| PC-5p-66343-10 | G | T | T | T | A | T | T | A | G | C | T | T | G | G | T | T | T | A | A | T | T | T | G | T | T | G | G | A | T | T | T | T | G | T | T | C | A | G | C | A | A | T | G | T | G | C | G | C | A | A | A | A | - | - | - | - |  |  |  |  |
| PC-5p-66343-5 | T | T | T | C | A | A | A | T | G | A | A | T | A | T | C | A | T | T | T | T | A | T | G | T | T | T | A | A | T | T | T | T | T | G | T | G | G | G | A | A | A | T | A | T | G | - | - | - | - | - | - | - | - | - | - | - |  |  |  |  |
| PC-5p-66343-8 | G | T | C | G | G | A | T | C | G | G | G | C | T | G | A | A | A | A | T | T | T | T | A | T | T | - | A | T | T | T | C | T | T | T | T | C | C | G | C | A | A | A | T | T | A | C | A | G | A | A | A | - | - | - | - | - |  |  |  |  |
| PC-5p-66343-4 | A | A | T | G | C | A | T | T | G | G | A | T | T | G | - | - | A | A | T | T | G | T | A | T | T | G | A | A | T | T | G | - | A | T | T | A | G | A | A | T | T | T | T | T | A | G | G | C | A | T | T | G | - | - | - | - |  |  |  |  |
| PC-5p-66343-19 | A | A | A | G | A | C | A | G | G | A | A | T | C | G | T | C | G | G | A | T | G | A | T | G | T | A | A | T | A | T | T | A | T | T | G | C | T | C | A | A | A | T | C | G | A | G | T | C | A | G | T | - | - | - | - | - |  |  |  |  |
| PC-5p-66343-6 | G | T | C | T | T | C | C | T | T | A | A | A | A | G | T | T | A | T | A | C | G | G | A | A | T | C | A | A | A | T | A | A | A | T | T | A | G | C | C | A | C | A | A | A | G | G | A | G | A | A | - | - | - | - | - | - |  |  |  |  |
| PC-5p-66343-2 | C | T | C | A | T | C | A | C | T | A | A | A | T | A | C | A | A | T | T | A | T | T | T | C | A | A | T | T | T | T | A | A | A | A | T | C | G | A | A | T | T | T | A | G | A | G | A | T | C | A | A | G | T | - | - | - |  |  |  |  |
| PC-5p-66343-3 | - | - | - | - | - | - | - | - | - | - | - | - | - | - | - | - | - | - | - | - | - | - | - | - | - | - | - | - | - | - | - | - | - | - | - | - | - | - | - | - | - | - | - | - | - | - | - | - | - | - | - | - | - | - | - | - |  |  |  |  |
| PC-5p-66343-15 | C | A | C | A | G | C | A | A | G | - | G | C | G | C | G | T | G | C | C | T | T | T | A | A | T | C | C | A | T | T | T | A | A | A | T | A | A | A | T | G | A | T | A | A | A | T | A | T | C | A | A | - | - | - | - | - |  |  |  |  |
| PC-5p-66343-18 | T | T | C | A | A | A | T | C | G | - | A | C | T | C | A | C | T | G | C | T | T | T | T | T | T | T | A | A | A | T | T | C | G | - | - | A | A | G | T | G | A | A | A | T | A | T | A | C | T | G | T | T | - | - | - | - |  |  |  |  |
| PC-5p-66343-17 | T | T | C | A | G | A | T | A | T | - | T | C | A | T | A | T | T | A | A | C | C | A | A | T | C | A | A | G | C | C | A | A | A | A | A | C | A | A | A | T | T | T | A | G | T | A | A | T | T | T | T | A | - | - | - | - |  |  |  |  |
| PC-5p-66343-20 | G | A | A | A | A | T | T | T | T | - | T | C | A | G | T | C | G | A | C | A | G | C | A | T | T | T | C | C | C | A | T | T | T | G | T | C | A | T | A | A | C | A | A | A | T | A | A | C | A | G | - | - | - | - | - | - |  |  |  |  |
| PC-5p-66343-11 | T | G | C | A | C | T | T | T | - | - | T | G | T | T | T | C | A | A | A | A | T | C | G | T | G | G | T | G | - | - | - | - | - | - | T | C | G | G | A | A | A | T | G | A | A | C | G | C | A | A | A | A | A | - | - | - |  |  |  |  |
| PC-5p-66343-12 | C | A | C | A | A | A | T | C | G | C | T | G | T | T | A | G | A | A | G | A | T | C | A | C | G | G | A | A | G | G | T | T | T | T | T | T | T | T | C | T | A | T | G | C | A | C | T | C | T | T | T | T | - | - | - | - |  |  |  |  |

**C: The 61169 family**

| PC-5p-61169-3 | - | - | T | A | A | A | A | T | C | G | A | T | - | T | G | A | G | T | G | A | A | A | T | A | A | T | T | T | G | A | A | C | A | A | A | C | C | G | A | T | G | A | A | C | T | T | T | G | T | A | A | T | A | A | G | T | C | T | T | G |
| --- | --- | --- | --- | --- | --- | --- | --- | --- | --- | --- | --- | --- | --- | --- | --- | --- | --- | --- | --- | --- | --- | --- | --- | --- | --- | --- | --- | --- | --- | --- | --- | --- | --- | --- | --- | --- | --- | --- | --- | --- | --- | --- | --- | --- | --- | --- | --- | --- | --- | --- | --- | --- | --- | --- | --- | --- | --- | --- | --- | --- |
| PC-5p-61169-10 | - | A | T | T | T | T | T | A | C | G | A | T | G | T | T | T | G | T | C | A | T | T | T | G | T | T | T | G | G | A | A | A | A | T | A | T | T | A | A | T | A | T | T | G | A | T | T | G | T | - | - | T | A | A | G | T | C | - | - | G |
| PC-5p-61169-1 | - | - | - | - | T | G | G | G | T | G | G | A | G | A | T | G | G | T | A | A | T | T | G | C | T | C | A | T | C | T | C | T | G | A | T | T | T | G | C | C | T | G | A | A | A | T | T | G | T | A | A | A | A | T | A | T | C | T | T | T |
| PC-5p-61169-2 | - | - | A | T | T | T | G | G | A | T | T | T | G | T | C | A | T | G | A | C | A | A | - | - | T | G | A | A | T | G | A | A | A | A | A | T | G | G | A | T | G | G | A | C | T | T | A | G | C | A | T | T | A | G | G | T | A | T | T | G |
| PC-5p-61169-6 | - | - | - | - | T | T | G | A | C | A | C | C | A | C | C | A | C | G | A | C | G | A | G | C | C | A | A | T | C | G | A | C | G | A | A | T | G | G | C | T | G | G | A | T | C | T | C | G | C | A | T | A | A | G | G | T | G | T | T | G |
| PC-5p-61169-5 | - | - | C | T | A | T | T | T | T | T | C | C | A | A | A | C | T | G | A | A | G | C | C | A | T | T | C | T | T | - | A | A | A | C | T | T | C | G | A | A | C | G | A | A | A | T | A | A | C | G | T | T | T | - | - | A | T | A | A | A |
| PC-5p-61169-4 | A | A | T | G | C | C | A | A | A | T | A | T | G | A | A | T | G | T | A | A | A | A | A | A | A | A | A | T | T | A | A | A | A | T | A | T | T | G | T | T | A | G | A | G | T | - | - | - | C | A | T | A | C | G | - | T | A | T | T | T |
| PC-5p-61169-9 | C | A | C | A | G | T | A | T | A | A | T | C | G | A | C | A | A | C | A | C | C | A | A | A | T | A | A | T | C | G | A | A | A | A | G | C | A | A | C | A | T | G | T | A | T | G | - | - | C | A | T | A | C | - | - | - | T | A | T | C |
| PC-5p-61169-7 | - | - | C | T | T | C | A | A | T | T | T | G | G | A | T | T | C | G | A | G | C | G | A | C | A | T | T | T | T | T | A | T | C | C | A | T | T | G | T | G | A | C | A | T | T | T | G | - | - | G | C | C | A | G | T | T | G | A | T | - |
| PC-5p-61169-8 | - | - | - | T | T | G | T | G | T | A | T | A | G | A | T | A | A | T | A | A | A | A | A | T | A | G | C | C | C | T | T | C | G | C | T | T | T | G | G | A | T | T | C | C | A | A | G | - | - | C | T | T | A | G | T | T | G | A | T | C |
|  |  |  |  |  |  |  |  |  |  |  |  |  |  |  |  |  |  |  |  |  |  |  |  |  |  |  |  |  |  |  |  |  |  |  |  |  |  |  |  |  |  |  |  |  |  |  |  |  |  |  |  |  |  |  |  |  |  |  |  |  |
| PC-5p-61169-3 | C | T | T | T | T | G | A | A | G | T | C | G | T | G | T | T | T | A | T | - | T | T | G | T | C | A | A | C | G | A | C | C | A | T | A | T | C | A | T | G | T | - | A | A | A | A | A | A | C | A | C | C | A | G | T | T | C | T | C | G |
| PC-5p-61169-10 | C | G | T | C | A | A | T | A | T | C | A | A | T | G | A | T | T | A | A | - | T | T | G | T | C | A | A | C | G | A | C | C | A | T | A | T | C | A | T | G | T | A | A | A | A | A | A | A | C | A | C | C | A | G | T | T | C | T | C | G |
| PC-5p-61169-1 | C | A | A | T | T | A | A | A | T | T | G | G | T | A | T | C | T | A | T | - | T | C | G | T | C | A | A | C | G | A | C | C | A | T | A | T | C | A | T | G | T | - | A | A | A | A | A | A | C | A | C | C | A | G | T | T | C | T | C | G |
| PC-5p-61169-2 | C | A | T | C | A | A | A | A | G | T | C | G | T | A | T | T | C | A | T | - | T | C | G | T | C | A | A | C | G | A | C | C | A | T | A | T | C | A | T | G | T | - | A | A | A | A | A | A | C | A | C | C | A | G | T | T | C | T | C | G |
| PC-5p-61169-6 | C | A | T | C | A | A | A | A | A | T | C | G | T | T | T | T | C | A | T | - | T | C | G | T | C | A | A | C | G | A | C | C | A | T | A | T | C | A | T | G | T | - | A | A | A | A | A | A | C | A | C | C | A | G | T | T | C | T | C | G |
| PC-5p-61169-5 | C | G | G | A | A | A | G | A | T | T | C | G | T | A | T | T | C | A | C | A | T | C | G | T | C | A | A | C | G | A | C | C | A | T | A | T | C | A | T | G | T | - | A | A | A | A | A | A | C | A | C | C | A | G | T | T | C | T | C | G |
| PC-5p-61169-4 | C | A | T | A | A | A | T | A | - | T | A | G | T | A | A | T | G | A | A | T | T | T | G | T | C | A | A | C | G | G | C | C | A | T | A | T | C | A | T | G | T | - | A | A | A | A | A | A | C | A | C | C | A | G | T | T | C | T | C | G |
| PC-5p-61169-9 | C | A | T | T | T | G | C | A | - | T | C | G | T | A | A | T | A | C | A | T | T | C | G | T | C | A | A | C | G | A | C | C | A | T | A | T | C | A | T | G | T | - | A | A | A | A | A | A | C | A | C | C | A | G | T | T | C | T | C | G |
| PC-5p-61169-7 | T | G | T | T | T | T | G | C | G | C | T | G | A | A | T | T | T | C | A | T | T | T | G | T | C | A | A | C | G | A | C | C | A | T | A | T | C | A | T | G | T | - | A | A | A | A | A | A | C | A | C | C | A | G | T | T | C | T | C | G |
| PC-5p-61169-8 | A | G | T | C | T | T | A | T | G | T | T | G | A | G | T | T | T | C | A | T | T | T | G | T | C | A | A | C | G | A | C | C | A | T | A | T | C | A | T | G | T | - | A | A | A | A | A | G | C | A | C | C | A | G | T | T | C | T | C | G |

**Complementary**

**miRNA starts├ miRNA ends ┤ ├ sequence starts**

| PC-5p-61169-3 | T | C | C | G | A | T | C | A | C | T | G | A | A | G | T | T | A | A | G | C | T | G | C | A | T | T | G | A | G | C | G | C | G | G | T | T | A | G | T | A | C | T | T | G | G | A | T | G | G | G | T | G | A | C | C | G | C | T | T | G |
| --- | --- | --- | --- | --- | --- | --- | --- | --- | --- | --- | --- | --- | --- | --- | --- | --- | --- | --- | --- | --- | --- | --- | --- | --- | --- | --- | --- | --- | --- | --- | --- | --- | --- | --- | --- | --- | --- | --- | --- | --- | --- | --- | --- | --- | --- | --- | --- | --- | --- | --- | --- | --- | --- | --- | --- | --- | --- | --- | --- | --- |
| PC-5p-61169-10 | T | C | C | G | A | T | C | A | C | T | G | A | A | G | T | T | A | A | G | C | T | G | C | A | T | T | G | A | G | C | G | C | G | G | T | T | G | G | T | A | C | T | T | G | G | A | T | G | G | G | T | G | A | C | C | G | C | T | T | G |
| PC-5p-61169-1 | T | C | C | G | A | T | C | A | C | T | G | A | A | G | T | T | A | A | G | C | T | G | C | A | T | T | G | A | G | C | G | C | G | G | T | T | A | G | T | A | C | T | T | G | G | A | T | G | G | G | T | G | A | C | C | G | C | T | T | G |
| PC-5p-61169-2 | T | C | C | G | A | T | C | A | C | T | G | A | A | G | T | T | A | A | G | C | T | G | C | A | T | T | G | A | G | C | G | C | G | G | T | T | A | G | T | A | C | T | T | G | G | A | T | G | G | G | T | G | A | C | C | G | C | T | T | G |
| PC-5p-61169-6 | T | C | C | G | A | T | C | A | C | T | G | A | A | G | T | T | A | A | G | C | T | G | C | A | T | T | G | A | G | C | G | C | G | G | T | T | A | G | T | A | C | T | T | G | G | A | T | G | G | G | T | G | A | C | C | G | C | T | T | G |
| PC-5p-61169-5 | T | C | C | G | A | T | C | A | C | T | G | A | A | G | T | T | A | A | G | C | T | G | C | A | T | T | G | A | G | C | G | C | G | G | T | T | A | G | T | A | C | T | T | G | G | A | T | G | G | G | T | G | A | C | C | G | C | T | T | G |
| PC-5p-61169-4 | T | C | C | G | A | T | C | A | C | T | G | A | A | G | T | T | A | A | G | C | T | G | C | A | T | T | G | A | G | C | G | C | G | G | T | T | A | G | T | A | C | T | T | G | G | A | T | G | G | G | T | G | A | C | C | G | C | T | T | G |
| PC-5p-61169-9 | T | G | C | G | A | T | C | A | C | T | G | A | A | G | C | T | T | A | G | C | T | G | C | A | T | T | G | A | G | C | G | C | G | G | T | T | A | G | T | A | C | T | T | G | A | C | T | G | G | G | C | G | A | C | C | G | T | T | T | G |
| PC-5p-61169-7 | T | C | C | G | A | T | C | A | C | T | G | A | A | G | T | T | A | A | G | C | T | G | C | A | T | T | G | A | G | C | G | C | G | G | T | T | A | G | T | A | C | T | T | G | G | A | T | G | G | G | T | G | A | C | C | G | C | T | T | G |
| PC-5p-61169-8 | T | C | C | G | A | T | C | A | C | T | G | A | A | G | T | T | A | A | G | C | T | G | C | A | T | T | G | A | G | C | G | C | G | G | T | T | A | G | T | A | C | T | T | G | G | A | T | G | G | G | T | G | A | C | C | G | C | T | T | G |

**Complementary**

**┤ sequence ends**

| PC-5p-61169-3 | G | G | A | A | C | A | C | C | G | C | G | T | G | T | C | G | T | T | G | G | C | A | C | C | A | T | T | T | T | T | T | T | C | T | C | T | C | G | A | T | T | C | T | A | T | - | T | C | A | T | T | T | T | T | - | T | A | T | T | G |
| --- | --- | --- | --- | --- | --- | --- | --- | --- | --- | --- | --- | --- | --- | --- | --- | --- | --- | --- | --- | --- | --- | --- | --- | --- | --- | --- | --- | --- | --- | --- | --- | --- | --- | --- | --- | --- | --- | --- | --- | --- | --- | --- | --- | --- | --- | --- | --- | --- | --- | --- | --- | --- | --- | --- | --- | --- | --- | --- | --- | --- |
| PC-5p-61169-10 | G | G | A | A | C | A | C | C | G | C | G | T | G | T | C | G | T | T | G | G | C | A | C | C | T | C | C | A | T | T | T | T | T | T | T | - | - | - | - | T | T | C | T | T | G | - | T | C | A | T | T | T | T | T | G | T | G | A | T | G |
| PC-5p-61169-1 | G | G | A | A | C | A | C | C | G | C | G | T | G | T | C | G | T | T | G | G | C | A | C | A | A | T | T | C | T | T | T | T | T | T | T | T | - | - | - | T | T | C | T | T | T | - | T | T | T | T | T | T | C | A | A | C | A | G | A | A |
| PC-5p-61169-2 | G | G | A | A | C | A | C | C | G | C | G | T | G | T | C | G | T | T | G | G | C | A | T | A | A | A | C | T | T | T | T | T | T | G | - | C | A | T | T | T | T | C | T | T | T | C | T | T | C | T | T | T | T | T | A | T | - | - | C | G |
| PC-5p-61169-6 | G | G | A | A | C | A | C | C | G | C | G | T | G | T | C | G | T | T | G | G | C | A | T | A | A | A | C | T | T | T | T | T | T | T | T | C | C | T | C | G | A | T | T | T | T | A | T | T | C | T | A | T | T | T | T | A | G | G | C | G |
| PC-5p-61169-5 | G | G | A | A | C | A | C | C | G | C | G | T | G | T | C | G | T | T | G | G | C | A | C | A | T | C | G | T | T | T | T | T | T | T | A | A | A | T | C | A | T | T | T | T | - | - | T | T | C | T | A | A | T | A | A | T | A | T | T | T |
| PC-5p-61169-4 | G | G | A | A | C | A | C | C | G | C | G | T | G | T | C | G | T | T | G | G | C | A | C | A | A | T | T | A | T | T | T | T | T | T | C | T | C | T | C | C | T | T | T | T | T | - | T | C | C | T | T | T | A | T | T | A | T | T | A | T |
| PC-5p-61169-9 | G | G | A | A | C | A | C | C | G | C | G | T | G | T | C | G | T | T | G | A | C | A | T | A | A | T | - | - | - | T | T | T | T | T | C | T | T | C | C | A | T | T | T | T | T | - | T | T | C | C | T | G | T | T | T | A | T | T | C | C |
| PC-5p-61169-7 | G | G | A | A | C | A | C | C | G | C | G | T | G | T | C | G | T | T | G | G | C | A | T | A | C | A | T | T | T | T | T | T | T | T | T | T | C | G | T | G | T | T | - | G | G | T | T | T | G | A | A | T | C | T | T | - | A | A | G | T |
| PC-5p-61169-8 | G | G | A | A | C | A | C | C | G | C | G | T | G | T | T | G | T | T | G | C | C | A | T | A | C | A | T | T | T | T | T | T | T | T | C | A | A | T | T | T | T | T | C | G | G | T | T | T | T | A | A | T | C | T | T | C | A | A | G | T |

| PC-5p-61169-3 | A | T | C | A | C | T | T | T | T | T | C | G | G | G | A | C | G | A | A | A | T | A | A | A | T | A | C | C | A | A | A | A | A | T | - | - | C | G | T | C | A | A | A | G | A | A | A | A | A | C | A | A | C | A | A | A | A | T | A | A |
| --- | --- | --- | --- | --- | --- | --- | --- | --- | --- | --- | --- | --- | --- | --- | --- | --- | --- | --- | --- | --- | --- | --- | --- | --- | --- | --- | --- | --- | --- | --- | --- | --- | --- | --- | --- | --- | --- | --- | --- | --- | --- | --- | --- | --- | --- | --- | --- | --- | --- | --- | --- | --- | --- | --- | --- | --- | --- | --- | --- | --- |
| PC-5p-61169-10 | G | T | A | C | C | A | A | C | C | A | C | A | G | A | T | A | A | T | T | T | T | T | C | T | T | T | T | C | A | T | T | A | A | C | T | A | C | G | T | C | T | T | A | T | C | C | A | C | A | A | A | A | A | A | T | A | A | A | A | A |
| PC-5p-61169-1 | T | T | T | T | A | A | A | A | A | G | T | A | A | A | T | C | A | A | T | C | C | T | T | T | T | C | G | C | T | A | T | A | A | T | A | T | A | T | G | T | A | G | T | T | A | T | A | A | T | C | A | A | A | A | T | A | A | A | A | T |
| PC-5p-61169-2 | C | A | T | T | - | - | T | T | A | T | A | A | T | A | T | T | C | A | A | A | C | C | A | T | T | T | T | C | T | A | C | T | A | G | A | T | G | A | G | - | - | A | A | A | T | T | T | T | T | G | G | T | A | G | G | C | T | T | T | A |
| PC-5p-61169-6 | A | A | A | T | G | C | A | T | G | T | T | A | G | A | T | A | C | A | C | A | T | C | T | A | T | T | T | A | T | A | A | T | A | A | A | T | T | T | - | - | - | - | A | A | T | T | T | T | A | A | A | A | A | T | G | A | A | T | T | G |
| PC-5p-61169-5 | T | T | T | T | T | T | T | A | T | T | C | A | G | A | T | G | G | A | T | A | T | A | A | A | T | A | T | T | T | A | A | T | T | T | T | A | T | T | - | - | - | - | T | A | T | T | T | T | T | T | A | T | T | A | G | T | T | T | T | T |
| PC-5p-61169-4 | T | T | A | A | A | A | T | A | T | T | C | G | A | A | T | G | G | T | T | T | T | C | T | T | T | T | T | A | T | G | A | A | T | T | C | G | A | T | T | T | T | A | T | G | C | T | T | T | C | C | A | G | A | A | C | C | T | T | A | A |
| PC-5p-61169-9 | C | - | C | A | T | T | T | A | T | T | C | G | A | T | T | - | C | A | T | G | A | C | A | A | T | C | A | A | C | A | A | A | T | T | G | G | T | T | A | G | C | C | G | A | A | A | A | T | T | C | A | A | A | T | G | C | T | A | G | A |
| PC-5p-61169-7 | T | G | A | G | T | T | T | T | C | A | T | C | A | T | A | G | G | T | T | T | T | T | C | T | T | A | T | C | A | T | A | T | A | T | T | C | A | T | - | - | - | - | G | A | A | T | T | C | T | C | C | T | T | T | A | T | T | T | T | C |
| PC-5p-61169-8 | T | G | A | G | T | T | T | T | T | A | T | C | A | A | A | G | - | A | T | T | T | T | C | T | T | T | T | T | T | C | A | T | A | T | C | T | A | T | - | - | - | - | A | A | A | T | T | C | G | C | C | T | G | T | A | T | A | T | T | C |
|  |  |  |  |  |  |  |  |  |  |  |  |  |  |  |  |  |  |  |  |  |  |  |  |  |  |  |  |  |  |  |  |  |  |  |  |  |  |  |  |  |  |  |  |  |  |  |  |  |  |  |  |  |  |  |  |  |  |  |  |  |
| PC-5p-61169-3 | T | G | T | T | - | T | T | C | G | G | - | - | - | - | - |  |  |  |  |  |  |  |  |  |  |  |  |  |  |  |  |  |  |  |  |  |  |  |  |  |  |  |  |  |  |  |  |  |  |  |  |  |  |  |  |  |  |  |  |  |
| PC-5p-61169-10 | T | T | T | T | A | T | T | C | A | T | - | - | - | - | - |  |  |  |  |  |  |  |  |  |  |  |  |  |  |  |  |  |  |  |  |  |  |  |  |  |  |  |  |  |  |  |  |  |  |  |  |  |  |  |  |  |  |  |  |  |
| PC-5p-61169-1 | T | A | G | A | A | A | G | C | G | - | - | - | - | - | - |  |  |  |  |  |  |  |  |  |  |  |  |  |  |  |  |  |  |  |  |  |  |  |  |  |  |  |  |  |  |  |  |  |  |  |  |  |  |  |  |  |  |  |  |  |
| PC-5p-61169-2 | T | G | T | A | A | T | A | A | A | A | A | T | A | C | - |  |  |  |  |  |  |  |  |  |  |  |  |  |  |  |  |  |  |  |  |  |  |  |  |  |  |  |  |  |  |  |  |  |  |  |  |  |  |  |  |  |  |  |  |  |
| PC-5p-61169-6 | G | G | - | - | - | - | A | C | G | A | A | T | G | - | - |  |  |  |  |  |  |  |  |  |  |  |  |  |  |  |  |  |  |  |  |  |  |  |  |  |  |  |  |  |  |  |  |  |  |  |  |  |  |  |  |  |  |  |  |  |
| PC-5p-61169-5 | T | T | T | - | - | - | T | A | G | A | A | T | T | T | - |  |  |  |  |  |  |  |  |  |  |  |  |  |  |  |  |  |  |  |  |  |  |  |  |  |  |  |  |  |  |  |  |  |  |  |  |  |  |  |  |  |  |  |  |  |
| PC-5p-61169-4 | A | T | A | - | - | - | T | C | A | - | - | - | - | - | - |  |  |  |  |  |  |  |  |  |  |  |  |  |  |  |  |  |  |  |  |  |  |  |  |  |  |  |  |  |  |  |  |  |  |  |  |  |  |  |  |  |  |  |  |  |
| PC-5p-61169-9 | G | G | T | - | - | - | T | A | G | A | G | G | T | - | - |  |  |  |  |  |  |  |  |  |  |  |  |  |  |  |  |  |  |  |  |  |  |  |  |  |  |  |  |  |  |  |  |  |  |  |  |  |  |  |  |  |  |  |  |  |
| PC-5p-61169-7 | G | G | T | - | - | - | C | G | A | A | A | T | T | C | A |  |  |  |  |  |  |  |  |  |  |  |  |  |  |  |  |  |  |  |  |  |  |  |  |  |  |  |  |  |  |  |  |  |  |  |  |  |  |  |  |  |  |  |  |  |
| PC-5p-61169-8 | G | T | T | - | - | - | C | A | A | A | A | T | T | C | - |  |  |  |  |  |  |  |  |  |  |  |  |  |  |  |  |  |  |  |  |  |  |  |  |  |  |  |  |  |  |  |  |  |  |  |  |  |  |  |  |  |  |  |  |  |

**D: The 39989 family**

| PC-5p-39989-1 | - | - | - | - | - | - | - | - | - | - | - | - | - | - | - | - | - | - | - | - | - | - | - | - | - | - | - | - | - | - | - | - | - | - | - | - | - | - | - | - | - | - | - | - | - | - | - | - | - | T | T | T | T | G | - | - | - | - | - | - |
| --- | --- | --- | --- | --- | --- | --- | --- | --- | --- | --- | --- | --- | --- | --- | --- | --- | --- | --- | --- | --- | --- | --- | --- | --- | --- | --- | --- | --- | --- | --- | --- | --- | --- | --- | --- | --- | --- | --- | --- | --- | --- | --- | --- | --- | --- | --- | --- | --- | --- | --- | --- | --- | --- | --- | --- | --- | --- | --- | --- | --- |
| PC-5p-39989-3 | - | - | - | - | - | - | - | - | - | - | - | - | - | - | - | - | G | G | T | A | C | - | - | - | - | - | - | - | - | C | G | A | T | T | C | - | - | C | A | G | C | G | T | - | - | T | G | - | - | T | T | T | C | G | G | A | A | C | C | G |
| PC-5p-39989-13 | - | - | - | - | - | - | - | - | - | - | - | - | - | A | A | A | A | A | C | A | C | - | - | - | - | - | - | - | - | C | A | T | T | T | G | - | - | A | A | A | G | G | T | T | C | T | A | - | - | T | T | T | T | G | - | - | - | - | - | - |
| PC-5p-39989-24 | - | - | - | - | - | - | - | - | - | - | G | A | G | A | C | A | A | A | C | A | C | - | - | - | - | - | - | - | - | C | A | T | T | T | G | - | - | A | A | A | G | G | T | T | C | T | G | - | - | T | T | T | T | G | - | - | - | - | - | - |
| PC-5p-39989-5 | - | - | - | - | - | - | - | - | - | - | - | - | - | - | - | - | - | - | - | - | - | - | - | - | - | - | - | - | - | - | - | - | - | - | - | - | - | - | - | - | - | - | - | - | - | - | - | - | - | T | T | T | T | G | - | - | - | - | - | - |
| PC-5p-39989-9 | - | - | - | - | - | - | - | - | - | - | G | A | G | A | C | A | A | A | C | A | C | - | - | - | - | - | - | - | - | C | A | T | T | T | G | - | - | A | A | A | A | G | T | T | C | T | G | - | - | T | T | T | T | G | - | - | - | - | - | - |
| PC-5p-39989-4 | - | - | - | - | - | - | - | - | - | - | - | - | G | A | C | A | A | A | C | A | C | - | - | - | - | - | - | - | - | C | A | T | T | T | G | - | - | A | A | A | G | G | T | T | C | T | G | - | - | T | T | T | T | G | - | - | - | - | - | - |
| PC-5p-39989-10 | - | - | - | - | - | - | - | - | - | - | G | A | G | A | C | A | A | A | C | A | C | - | - | - | - | - | - | - | - | C | A | T | T | T | G | - | - | A | A | A | G | G | T | T | C | T | G | - | - | T | T | C | T | G | - | - | - | - | - | - |
| PC-5p-39989-6 | - | - | - | - | - | - | - | - | A | T | A | A | T | T | C | C | T | G | C | A | C | - | - | - | - | - | - | - | - | C | A | T | A | T | G | - | - | G | A | A | G | G | T | G | C | T | G | - | - | T | G | C | T | G | - | - | - | - | - | - |
| PC-5p-39989-7 | - | - | - | - | - | - | - | - | A | T | A | A | T | T | C | C | T | G | C | A | C | - | - | - | - | - | - | - | - | C | A | T | A | T | G | - | - | G | A | A | G | G | T | G | C | T | G | - | - | T | G | C | T | G | - | - | - | - | - | - |
| PC-5p-39989-12 | - | - | - | - | - | - | - | - | - | - | - | - | - | - | - | - | - | - | - | A | C | - | - | - | - | - | - | - | - | C | A | T | A | T | G | - | - | A | A | A | G | G | T | G | C | T | G | - | - | T | G | C | T | G | - | - | - | - | - | - |
| PC-5p-39989-11 | - | - | - | - | - | - | - | - | - | - | - | - | - | - | - | - | A | G | G | C | A | - | - | - | - | - | - | - | - | C | T | T | T | T | G | - | - | G | A | A | T | T | T | C | C | G | A | - | - | T | A | C | T | C | G | A | A | A | A | C |
| PC-5p-39989-8 | - | - | - | - | - | - | - | - | A | T | A | A | T | T | C | C | T | G | C | A | C | - | - | - | - | - | - | - | - | C | A | T | A | T | G | - | - | A | A | A | G | G | T | G | C | T | G | - | - | T | G | C | T | G | - | - | - | - | - | - |
| PC-5p-39989-14 | - | - | - | - | - | - | - | - | - | C | A | A | T | T | C | G | G | A | A | A | C | - | - | - | - | - | - | - | - | C | T | T | G | T | T | G | C | A | A | A | G | - | - | - | C | T | G | - | - | T | G | T | T | G | - | - | - | - | - | - |
| PC-5p-39989-19 | - | - | - | - | - | - | - | - | - | - | - | A | T | T | C | G | G | A | A | A | C | - | - | - | - | - | - | - | - | C | T | T | A | T | T | G | C | A | A | A | G | G | T | G | C | T | G | - | - | T | G | T | T | G | - | - | - | - | - | - |
| PC-5p-39989-17 | - | - | - | - | - | - | - | - | - | - | - | - | A | C | C | C | T | T | C | T | T | - | - | - | - | - | - | - | - | C | T | C | A | T | A | C | C | A | A | G | A | A | A | A | A | T | T | G | A | A | T | C | C | G | - | - | - | - | - | - |
| PC-5p-39989-26 | - | - | - | - | - | - | - | - | - | - | - | - | - | T | C | G | G | A | G | A | C | - | - | - | - | - | - | - | - | C | T | C | A | T | T | G | C | A | A | A | G | C | T | G | C | T | G | - | - | A | G | C | T | G | - | - | - | - | - | - |
| PC-5p-39989-27 | - | - | - | - | - | - | - | - | - | - | - | - | - | - | - | - | - | - | - | - | - | - | - | - | - | - | - | - | - | - | - | - | - | - | - | - | - | - | - | - | - | - | - | - | - | - | - | - | - | - | - | - | - | - | - | - | - | - | - | - |
| PC-5p-39989-28 | - | - | - | - | - | - | - | G | C | A | C | T | T | T | T | G | G | A | A | T | T | T | - | - | - | - | - | - | C | C | G | C | T | A | C | - | - | T | C | G | A | A | A | A | C | - | A | C | C | T | - | - | - | - | - | - | - | - | - | - |
| PC-5p-39989-25 | - | - | - | - | - | - | - | - | C | C | A | T | T | T | C | G | G | A | A | A | A | T | T | T | A | T | G | T | C | C | G | C | A | T | T | - | - | T | G | G | G | C | T | G | C | - | C | C | G | T | G | C | T | G | - | - | - | - | - | - |
| PC-5p-39989-20 | - | - | - | - | - | - | - | - | - | - | - | T | T | T | G | G | G | A | T | A | G | - | - | - | - | - | - | - | - | C | T | C | A | T | T | - | - | T | G | A | A | A | G | C | C | T | C | T | G | T | G | C | T | G | - | - | - | - | - | - |
| PC-5p-39989-2 | - | - | - | - | - | - | - | - | - | - | - | - | G | T | G | T | C | A | A | A | C | A | - | - | - | - | - | - | - | C | A | C | A | T | A | T | T | C | T | T | T | A | T | C | A | A | A | C | G | C | A | C | A | - | - | - | - | - | - | - |
| PC-5p-39989-18 | C | A | C | A | C | C | T | C | C | T | C | T | C | T | G | T | C | A | A | A | C | A | - | - | - | - | - | - | - | C | A | C | A | T | A | T | T | C | T | T | T | A | T | C | A | A | A | C | G | C | A | C | A | - | - | - | - | - | - | - |
| PC-5p-39989-15 | - | - | - | - | - | - | - | - | - | - | - | - | - | - | - | - | - | - | - | - | - | - | - | - | - | - | - | - | - | - | - | - | - | - | - | - | - | C | T | T | T | A | T | C | A | A | A | C | G | C | A | C | A | - | - | - | - | - | - | - |
| PC-5p-39989-16 | - | - | - | - | - | - | - | - | - | - | - | - | - | - | G | C | T | G | T | A | T | A | - | - | - | - | - | - | - | C | A | C | G | - | - | T | T | T | A | T | G | C | G | C | C | A | C | T | T | C | G | T | T | - | - | - | - | - | - | - |
| PC-5p-39989-21 | - | - | - | - | - | - | - | - | - | - | - | - | - | - | A | A | T | A | T | A | T | T | - | - | - | - | - | - | - | G | A | G | A | A | A | T | T | C | A | T | A | C | A | T | A | C | A | T | A | C | A | C | A | G | - | - | - | - | - | - |
| PC-5p-39989-22 | - | - | - | - | - | - | G | A | A | T | A | T | T | C | A | C | A | - | T | A | C | A | - | - | - | - | - | - | - | T | T | C | A | G | - | - | - | C | G | A | G | C | G | T | G | T | A | G | A | A | A | C | A | T | - | - | - | - | - | - |
| PC-5p-39989-23 | - | - | - | - | - | - | - | - | A | C | A | T | A | C | G | C | A | G | T | A | C | A | - | - | - | - | - | - | - | C | T | C | A | T | C | A | C | C | G | A | C | T | C | A | G | T | G | A | G | C | G | C | G | C | - | - | - | - | - | - |

| PC-5p-39989-1 | - | - | - | T | A | G | - | - | A | T | G | T | - | - | - | - | - | - | - | G | C | A | G | T | - | - | - | - | - | T | T | C | C | G | A | A | T | T | T | T | G | A | T | T | T | T | T | T | A | A | T | T | T | T | A | A | A | T | T | A |
| --- | --- | --- | --- | --- | --- | --- | --- | --- | --- | --- | --- | --- | --- | --- | --- | --- | --- | --- | --- | --- | --- | --- | --- | --- | --- | --- | --- | --- | --- | --- | --- | --- | --- | --- | --- | --- | --- | --- | --- | --- | --- | --- | --- | --- | --- | --- | --- | --- | --- | --- | --- | --- | --- | --- | --- | --- | --- | --- | --- | --- |
| PC-5p-39989-3 | A | T | T | T | T | G | - | - | T | T | G | T | - | - | - | - | - | - | - | G | C | A | G | T | A | A | A | G | - | T | T | T | C | G | A | A | T | T | T | T | G | A | T | T | T | T | T | T | A | A | T | T | T | T | A | A | A | T | T | A |
| PC-5p-39989-13 | - | - | - | T | A | C | - | - | A | T | G | T | - | - | - | - | - | - | - | G | C | A | G | T | A | A | A | G | T | T | T | C | C | G | A | A | T | T | T | T | G | A | T | T | T | T | T | T | A | A | T | T | T | T | A | A | A | T | T | A |
| PC-5p-39989-24 | - | - | - | T | A | G | - | - | A | T | G | T | - | - | - | - | - | - | - | G | C | A | G | T | A | A | A | G | T | T | T | C | C | G | A | A | T | T | C | T | G | A | T | T | T | T | T | T | A | A | T | T | T | T | A | A | A | T | T | A |
| PC-5p-39989-5 | - | - | - | T | A | C | - | - | A | T | G | T | - | - | - | - | - | - | - | G | C | A | G | T | A | A | A | G | T | C | T | C | C | G | A | A | T | T | T | T | G | A | T | T | T | T | T | T | A | A | T | T | T | T | A | A | A | T | T | A |
| PC-5p-39989-9 | - | - | - | T | A | G | - | - | A | T | G | T | - | - | - | - | - | - | - | G | C | A | G | T | A | A | A | G | T | T | T | C | C | G | A | A | T | T | T | T | G | A | T | T | T | T | T | C | A | A | T | T | T | T | A | A | A | T | T | A |
| PC-5p-39989-4 | - | - | - | T | A | G | - | - | A | T | G | T | - | - | - | - | - | - | - | G | C | A | G | T | A | A | A | G | T | T | T | C | A | A | A | A | T | T | T | T | G | A | T | T | T | T | T | T | A | A | T | T | T | T | A | A | A | T | T | A |
| PC-5p-39989-10 | - | - | - | T | A | G | - | - | A | T | A | T | - | - | - | - | - | - | - | G | C | A | G | T | A | A | A | C | T | T | T | C | C | G | A | A | T | T | T | T | G | A | T | T | T | T | T | T | A | A | T | T | T | T | A | A | A | T | T | A |
| PC-5p-39989-6 | - | - | - | T | A | G | - | - | A | T | G | T | - | - | - | - | - | - | - | G | C | A | C | A | A | T | G | T | G | T | T | T | C | C | G | A | A | G | T | T | T | A | A | T | T | T | T | T | T | T | T | T | T | T | - | - | A | T | T | A |
| PC-5p-39989-7 | - | - | - | T | A | G | - | - | A | T | G | T | - | - | - | - | - | - | - | G | C | A | C | A | A | T | G | T | G | T | T | T | C | C | G | A | A | G | T | T | T | A | A | T | T | T | T | T | T | T | T | T | T | T | - | - | A | T | T | A |
| PC-5p-39989-12 | - | - | - | T | A | G | - | - | A | T | G | T | G | C | A | A | T | G | T | G | C | A | C | A | A | T | G | T | G | T | T | T | C | C | G | A | A | G | T | T | T | A | A | T | T | T | T | T | T | T | T | T | T | T | A | A | A | T | T | A |
| PC-5p-39989-11 | A | C | C | T | T | A | - | - | A | T | G | T | - | - | - | - | - | - | - | G | C | A | C | A | A | T | G | T | G | T | T | T | C | C | G | A | A | G | T | T | T | A | A | T | T | T | T | T | T | T | T | T | A | A | - | - | - | T | T | A |
| PC-5p-39989-8 | - | - | - | T | A | G | - | - | A | T | G | T | - | - | - | - | - | - | - | G | C | A | C | A | A | T | G | T | G | T | T | T | C | C | G | A | A | G | T | T | T | A | A | T | T | T | T | T | C | T | T | T | T | T | - | A | A | T | T | A |
| PC-5p-39989-14 | - | - | - | T | A | G | - | - | A | T | G | T | - | - | - | - | - | - | - | G | C | A | G | T | A | A | G | G | T | T | T | C | C | G | A | A | T | T | T | T | A | A | T | T | T | A | T | T | A | A | A | T | T | C | A | A | A | T | T | A |
| PC-5p-39989-19 | - | - | - | T | A | G | - | - | A | T | G | T | - | - | - | - | - | - | - | G | C | A | G | T | A | A | G | G | T | T | T | C | C | G | A | A | T | T | T | T | A | A | T | T | T | A | T | T | A | A | A | T | T | T | A | A | A | T | T | A |
| PC-5p-39989-17 | - | - | - | T | A | T | G | A | A | A | A | T | - | - | - | - | - | - | - | G | A | A | C | A | A | G | A | A | T | T | T | T | C | C | A | A | - | - | A | A | A | T | G | A | C | G | T | T | T | T | C | C | C | G | T | G | G | T | G | T |
| PC-5p-39989-26 | - | - | - | T | A | G | - | - | A | T | G | T | - | - | - | - | - | - | - | G | C | A | G | T | A | A | A | G | T | C | T | T | C | G | A | A | T | T | A | A | T | T | T | T | T | T | T | T | T | A | T | T | C | T | A | A | A | T | T | A |
| PC-5p-39989-27 | - | - | - | - | - | - | - | - | - | - | - | - | - | - | - | - | - | - | - | - | - | - | - | - | A | A | G | G | - | C | T | G | G | A | A | T | T | T | A | A | T | A | A | A | A | A | T | T | A | A | T | T | C | T | A | A | G | T | T | T |
| PC-5p-39989-28 | - | - | - | - | T | A | - | - | A | T | G | T | - | - | - | - | - | - | - | G | C | A | A | T | A | A | G | G | - | C | T | G | G | A | A | T | T | T | A | A | T | A | A | A | A | A | T | T | A | A | T | T | C | T | A | A | G | T | T | T |
| PC-5p-39989-25 | - | - | - | T | A | G | - | - | A | T | G | T | - | - | - | - | - | - | - | G | C | A | A | T | A | A | G | G | - | C | T | G | G | A | A | T | T | T | A | A | T | T | A | A | A | A | T | T | A | A | T | T | C | T | A | A | G | T | T | T |
| PC-5p-39989-20 | - | - | - | T | A | G | - | - | A | T | G | T | - | - | - | - | - | - | - | G | C | A | A | T | A | A | G | G | - | C | T | G | G | A | A | T | T | T | A | A | T | T | G | A | A | A | T | T | A | A | T | T | C | T | A | A | G | T | T | T |
| PC-5p-39989-2 | - | - | - | C | C | G | A | T | A | C | T | A | - | - | - | - | - | - | A | A | A | C | A | A | C | T | G | T | A | C | T | G | C | A | C | A | G | A | A | A | A | A | A | A | C | A | T | G | A | A | A | A | T | T | A | A | A | T | G | A |
| PC-5p-39989-18 | - | - | - | C | C | G | A | C | A | C | T | A | - | - | - | - | - | - | A | A | A | C | T | A | C | T | G | T | A | C | T | G | C | A | C | A | G | A | A | A | A | A | A | A | A | A | T | G | A | A | A | A | T | T | A | A | A | T | G | A |
| PC-5p-39989-15 | - | - | - | C | C | G | A | C | A | C | T | A | - | - | - | - | - | - | A | A | A | C | T | A | C | T | G | T | A | C | T | G | C | A | C | A | G | A | A | A | A | A | A | A | C | A | T | G | A | A | A | A | T | T | A | A | A | T | G | A |
| PC-5p-39989-16 | - | - | - | T | G | A | T | G | T | C | G | C | - | - | - | - | - | - | G | A | G | C | T | T | G | - | G | T | A | C | T | G | C | A | C | A | G | A | A | A | A | A | A | A | C | A | T | C | A | A | A | A | T | T | A | A | A | T | G | A |
| PC-5p-39989-21 | - | - | - | C | A | C | A | C | T | C | A | T | - | - | - | - | - | - | C | A | C | C | A | G | C | T | C | A | G | T | G | A | G | T | C | A | G | T | G | A | T | G | A | G | T | G | T | G | C | T | G | C | G | T | A | T | G | T | A | T |
| PC-5p-39989-22 | - | - | - | G | T | A | G | T | T | T | A | T | - | - | - | - | - | - | A | C | A | G | T | T | T | T | C | G | C | T | G | A | G | T | C | G | G | C | G | A | T | G | A | G | T | G | T | - | - | - | - | - | - | - | - | - | - | - | G | G |
| PC-5p-39989-23 | - | - | - | G | A | A | A | T | A | T | A | T | - | - | - | - | - | - | A | C | G | A | T | A | T | T | C | A | C | T | G | A | G | T | C | G | G | T | G | T | T | G | A | G | T | G | T | G | C | T | G | C | G | T | A | G | G | T | G | T |

| PC-5p-39989-1 | A | T | A | A | A | A | A | A | T | C | G | A | A | A | A | A | C | A | A | A | A | G | T | A | C | A | T | A | G | A | T | T | T | T | G | C | G | T | T | T | - | - | - | - | - | - | - | T | C | T | G | G | A | A | T | G | A | A | A | G |
| --- | --- | --- | --- | --- | --- | --- | --- | --- | --- | --- | --- | --- | --- | --- | --- | --- | --- | --- | --- | --- | --- | --- | --- | --- | --- | --- | --- | --- | --- | --- | --- | --- | --- | --- | --- | --- | --- | --- | --- | --- | --- | --- | --- | --- | --- | --- | --- | --- | --- | --- | --- | --- | --- | --- | --- | --- | --- | --- | --- | --- |
| PC-5p-39989-3 | A | T | A | A | A | A | A | T | T | C | G | G | A | A | A | A | C | A | A | A | A | G | T | A | C | A | T | A | G | A | T | T | T | T | G | C | G | T | T | T | - | - | - | - | - | - | - | T | C | T | G | C | A | A | T | G | A | A | A | T |
| PC-5p-39989-13 | A | T | A | A | A | A | A | T | T | C | G | G | A | A | A | A | C | A | A | A | A | G | T | A | C | A | T | A | G | A | T | T | T | T | G | C | G | T | T | T | - | - | - | - | - | - | - | T | C | T | G | C | A | A | T | G | A | A | A | T |
| PC-5p-39989-24 | A | T | A | A | A | A | A | A | T | C | G | A | A | A | A | A | C | A | A | A | A | G | T | A | C | A | T | A | G | A | T | T | T | T | G | C | G | T | T | T | - | - | - | - | - | - | - | T | C | T | G | C | A | A | T | G | A | A | A | G |
| PC-5p-39989-5 | A | T | A | A | A | A | A | T | T | C | G | C | A | A | A | A | C | A | A | A | A | G | T | A | C | A | T | A | G | A | T | T | T | T | G | C | G | T | T | T | - | - | - | - | - | - | - | T | C | T | G | C | A | A | T | G | A | A | A | T |
| PC-5p-39989-9 | A | T | A | A | A | A | A | A | T | C | G | A | A | A | A | A | C | A | A | A | A | G | T | A | C | A | T | A | G | A | T | T | T | T | G | C | G | T | T | T | - | - | - | - | - | - | - | T | C | T | G | C | A | A | T | G | A | G | A | G |
| PC-5p-39989-4 | A | T | A | A | A | A | A | A | T | C | G | A | A | A | A | A | C | A | A | A | A | G | T | A | C | A | T | A | G | A | T | T | T | T | G | C | G | T | T | T | - | - | - | - | - | - | - | T | C | T | A | G | A | A | T | G | A | A | A | G |
| PC-5p-39989-10 | A | T | A | A | A | A | A | A | T | C | G | A | A | A | A | A | C | A | A | A | A | G | T | A | C | A | T | A | G | A | T | T | T | T | G | C | G | T | T | T | - | - | - | - | - | - | - | T | C | T | G | C | A | A | T | A | A | A | A | G |
| PC-5p-39989-6 | A | T | A | A | A | A | A | A | T | C | G | A | A | A | A | T | A | A | A | A | A | G | T | A | G | G | T | A | G | T | T | T | T | T | C | G | T | T | T | T | - | - | - | - | - | - | - | T | C | T | G | G | A | T | T | A | A | A | A | G |
| PC-5p-39989-7 | A | T | A | A | A | A | A | A | T | C | G | A | A | A | A | T | A | A | A | A | A | G | T | A | G | G | T | A | G | T | T | T | T | T | C | G | T | T | T | T | - | - | - | - | - | - | - | T | C | T | G | G | A | T | T | A | A | A | A | G |
| PC-5p-39989-12 | A | T | A | A | A | A | A | A | T | C | G | A | A | A | A | T | A | A | A | A | A | G | T | A | G | G | T | A | G | T | T | T | T | T | C | G | T | T | T | T | - | - | - | - | - | - | - | T | C | T | G | G | A | T | T | A | A | A | A | G |
| PC-5p-39989-11 | A | T | A | A | A | A | A | A | T | C | G | A | A | A | A | T | A | A | A | A | A | G | T | A | G | G | T | A | G | T | T | T | T | T | C | G | T | T | T | T | - | - | - | - | - | - | - | T | C | T | G | G | A | G | T | A | A | A | A | G |
| PC-5p-39989-8 | A | T | A | A | A | A | A | A | T | C | G | A | A | A | A | T | A | A | A | A | A | G | T | A | G | G | T | A | G | - | T | T | T | T | C | G | T | T | T | T | - | - | - | - | - | - | - | T | C | T | G | G | A | A | T | A | A | A | A | G |
| PC-5p-39989-14 | A | T | A | A | A | A | A | A | T | C | G | A | A | A | A | G | A | A | A | A | A | G | T | A | T | G | T | A | G | A | T | T | T | T | G | A | G | T | T | T | - | - | - | - | - | - | - | T | C | T | G | G | A | A | T | A | A | A | A | G |
| PC-5p-39989-19 | A | T | A | A | A | A | A | A | T | C | G | A | A | A | A | - | A | A | A | A | A | G | T | A | T | G | T | A | G | A | T | T | T | T | G | A | G | T | T | T | - | - | - | - | - | - | - | T | C | T | G | G | A | A | T | G | A | A | A | G |
| PC-5p-39989-17 | A | T | T | C | A | A | A | C | T | T | - | - | - | T | T | G | G | C | C | T | G | T | A | C | T | G | T | A | G | A | T | T | T | T | G | C | G | T | T | T | - | - | - | - | - | - | - | T | T | C | G | G | A | A | T | G | A | T | T | T |
| PC-5p-39989-26 | A | T | T | A | A | A | A | A | T | T | G | A | A | A | A | G | A | A | A | A | A | G | T | A | T | G | T | A | G | A | T | T | T | T | G | C | G | T | T | T | - | - | - | - | - | - | - | T | C | T | G | G | A | A | T | G | A | T | T | T |
| PC-5p-39989-27 | A | T | T | A | A | A | A | A | T | C | C | G | A | T | T | A | T | A | A | A | A | G | T | G | G | G | A | A | G | A | T | T | T | T | G | C | G | T | T | T | - | - | - | - | - | - | - | T | C | T | G | G | A | A | G | G | A | A | A | T |
| PC-5p-39989-28 | A | T | T | A | A | A | A | A | T | C | C | G | A | T | T | A | T | A | A | A | A | G | T | G | G | G | A | A | G | A | T | T | T | T | G | T | G | T | T | T | - | - | - | - | - | - | - | T | C | T | G | G | A | A | G | G | A | A | A | T |
| PC-5p-39989-25 | A | T | T | A | A | A | A | A | T | C | C | G | A | T | T | A | T | A | A | A | A | G | T | A | G | G | A | A | G | A | T | T | T | T | G | C | G | T | T | T | - | - | - | - | - | - | - | T | C | T | G | - | - | - | - | - | - | - | - | - |
| PC-5p-39989-20 | A | T | T | A | A | A | A | A | T | C | C | G | A | T | T | C | T | A | A | A | A | G | T | A | G | G | A | A | G | A | T | T | T | T | G | C | G | T | T | T | - | - | - | - | - | - | - | T | T | T | G | G | A | A | G | G | A | A | A | T |
| PC-5p-39989-2 | T | G | A | A | G | A | A | G | A | A | G | A | A | G | A | A | G | T | T | T | A | C | A | C | A | T | C | T | C | C | G | T | A | A | T | C | A | A | A | G | - | - | - | - | - | - | - | - | C | A | G | C | A | C | A | G | C | G | - | - |
| PC-5p-39989-18 | T | G | A | A | T | A | A | G | A | A | G | T | A | - | - | - | - | - | - | - | - | - | - | - | A | T | C | A | A | A | G | C | A | A | A | A | C | A | T | - | - | - | - | - | - | - | - | - | C | A | G | C | A | C | A | G | C | G | - | - |
| PC-5p-39989-15 | C | G | A | A | G | A | A | G | A | A | G | A | A | G | - | - | - | T | A | T | A | C | A | C | A | T | C | T | C | C | G | T | A | A | T | C | A | A | A | G | C | A | A | A | A | C | A | G | C | A | G | C | A | C | A | G | C | G | A | T |
| PC-5p-39989-16 | T | G | A | A | G | A | A | G | A | A | G | A | A | G | - | - | - | T | A | T | A | C | A | C | A | T | C | T | C | C | G | T | A | A | T | C | A | A | T | G | C | A | A | A | A | C | A | G | C | A | G | C | A | C | A | T | C | G | - | - |
| PC-5p-39989-21 | G | T | A | T | G | A | A | T | T | T | G | A | C | A | A | T | A | T | C | A | T | G | A | A | T | G | T | T | C | A | T | T | T | A | G | C | T | A | T | A | G | - | - | - | - | - | - | - | C | A | A | C | A | C | A | G | C | G | - | - |
| PC-5p-39989-22 | G | T | A | T | G | A | A | T | T | T | C | A | C | A | A | - | - | T | A | T | T | G | T | G | A | A | T | G | T | G | T | A | T | T | G | G | T | G | T | A | G | - | - | - | - | - | - | - | C | A | G | C | A | C | A | G | C | G | G | C |
| PC-5p-39989-23 | G | T | A | T | G | A | A | T | T | T | C | A | C | A | A | - | - | C | A | T | T | G | - | A | A | T | T | G | T | - | A | A | T | A | G | C | T | A | T | A | G | - | - | - | - | - | - | - | C | A | G | C | A | C | A | G | C | G | - | - |

**Complementary**

**miRNA starts ├ miRNA ends ┤ ├ region starts**

| PC-5p-39989-1 | C | - | - | - | - | - | - | - | - | - | G | G | T | G | T | T | A | G | C | G | A | T | A | T | T | G | A | A | C | C | T | T | A | T | T | C | A | C | T | G | G | A | A | C | A | T | A | G | T | A | T | C | G | T | G | A | A | T | A | A |
| --- | --- | --- | --- | --- | --- | --- | --- | --- | --- | --- | --- | --- | --- | --- | --- | --- | --- | --- | --- | --- | --- | --- | --- | --- | --- | --- | --- | --- | --- | --- | --- | --- | --- | --- | --- | --- | --- | --- | --- | --- | --- | --- | --- | --- | --- | --- | --- | --- | --- | --- | --- | --- | --- | --- | --- | --- | --- | --- | --- | --- |
| PC-5p-39989-3 | C | - | - | - | - | - | - | - | - | - | G | G | T | G | T | T | A | G | C | G | A | T | A | T | T | G | A | A | C | C | T | T | A | T | T | C | A | C | T | G | G | A | A | C | A | T | A | G | T | A | T | C | G | T | G | A | A | T | G | A |
| PC-5p-39989-13 | C | - | - | - | - | - | - | - | - | - | G | G | T | G | T | T | A | G | C | G | A | T | A | T | T | G | A | A | C | C | T | T | A | T | T | C | A | C | T | G | G | A | A | C | A | T | A | G | T | A | T | C | G | T | G | A | A | T | A | A |
| PC-5p-39989-24 | C | - | - | - | - | - | - | - | - | - | G | G | T | G | T | T | A | G | C | G | A | T | A | T | T | G | G | A | C | C | T | T | A | T | T | C | A | C | T | T | G | A | A | C | A | T | T | G | T | A | T | C | G | T | G | A | A | T | A | A |
| PC-5p-39989-5 | C | - | - | - | - | - | - | - | - | - | G | G | T | G | T | T | A | G | C | G | A | T | A | T | T | G | A | A | C | C | T | T | A | T | T | C | A | C | T | G | G | A | A | C | A | T | A | G | T | A | T | C | G | T | G | A | A | T | A | A |
| PC-5p-39989-9 | C | - | - | - | - | - | - | - | - | - | G | G | T | G | T | T | A | G | C | G | A | T | A | T | T | G | A | A | C | C | T | T | A | T | T | C | A | C | T | G | G | A | A | C | A | T | A | G | T | A | T | C | G | T | G | A | A | T | A | A |
| PC-5p-39989-4 | C | - | - | - | - | - | - | - | - | - | G | G | T | G | T | T | A | G | C | G | A | T | A | T | T | G | A | A | C | C | T | T | A | T | T | C | A | C | T | G | G | A | A | C | A | T | A | T | T | A | T | C | G | T | G | A | A | A | C | A |
| PC-5p-39989-10 | C | - | - | - | - | - | - | - | - | - | G | G | T | G | T | T | A | G | C | G | A | T | A | T | T | G | A | A | C | C | T | T | A | T | T | C | A | C | T | G | G | A | A | C | A | T | A | G | T | A | T | C | G | T | G | A | A | T | A | A |
| PC-5p-39989-6 | C | - | - | - | - | - | - | - | - | - | G | C | T | G | T | T | A | G | C | G | A | T | A | T | T | G | A | A | C | C | T | T | A | T | T | C | A | C | T | G | G | A | A | G | T | T | G | T | T | C | T | G | G | T | G | A | A | T | A | A |
| PC-5p-39989-7 | C | - | - | - | - | - | - | - | - | - | G | C | T | G | T | T | A | G | C | G | A | T | A | T | T | G | A | A | C | C | T | T | A | T | T | C | A | C | T | G | G | A | A | G | T | T | G | T | T | C | T | G | G | T | G | A | A | T | A | A |
| PC-5p-39989-12 | C | - | - | - | - | - | - | - | - | - | G | C | T | G | T | T | A | G | C | G | A | T | A | T | T | G | A | A | C | C | T | T | A | T | T | C | A | C | T | T | G | A | A | G | T | T | G | T | T | T | T | G | G | T | G | A | A | T | A | A |
| PC-5p-39989-11 | C | - | - | - | - | - | - | - | - | - | G | C | T | G | T | T | A | G | C | G | A | T | A | T | T | G | A | A | C | C | T | T | A | T | T | C | A | C | T | A | G | A | A | G | T | T | G | T | T | T | T | G | G | T | G | A | A | T | A | A |
| PC-5p-39989-8 | C | - | - | - | - | - | - | - | - | - | G | C | T | G | T | T | A | G | C | G | A | T | A | T | T | G | A | A | C | C | T | T | A | T | T | C | A | C | T | G | G | A | A | G | T | T | G | T | T | T | T | G | G | T | G | A | A | T | A | A |
| PC-5p-39989-14 | T | - | - | - | - | - | - | - | - | - | G | C | T | G | T | T | A | G | C | G | A | T | A | T | T | G | A | A | C | C | T | T | A | T | T | C | A | C | T | G | G | A | A | C | A | T | A | G | T | A | T | C | G | T | G | A | A | T | A | A |
| PC-5p-39989-19 | T | - | - | - | - | - | - | - | - | - | G | C | T | G | T | T | A | G | C | G | A | T | A | T | T | G | A | A | C | C | T | T | A | T | T | - | - | - | - | - | - | - | - | C | A | T | A | G | T | A | T | C | G | T | G | A | A | T | A | G |
| PC-5p-39989-17 | G | - | - | - | - | - | - | - | - | - | C | C | T | G | T | T | T | G | C | G | A | T | A | T | T | G | A | A | C | C | T | T | A | T | T | C | A | C | T | G | G | A | A | C | A | T | A | G | T | A | C | C | G | T | C | A | A | T | A | T |
| PC-5p-39989-26 | T | - | - | - | - | - | - | - | - | - | C | C | T | G | T | T | T | G | C | G | A | T | A | T | T | G | A | A | C | C | C | T | A | T | T | C | A | C | T | G | G | A | A | C | A | T | A | G | T | A | C | C | G | T | C | A | A | T | A | T |
| PC-5p-39989-27 | G | - | - | - | - | - | - | - | - | - | G | T | T | G | T | C | A | G | C | G | A | C | A | T | T | G | A | A | C | C | T | T | A | T | T | C | A | C | G | A | G | A | A | G | T | T | G | T | A | T | C | G | G | T | G | A | A | T | A | A |
| PC-5p-39989-28 | G | - | - | - | - | - | - | - | - | - | G | T | T | G | T | C | A | G | C | G | A | C | A | T | T | G | A | A | C | C | T | T | A | T | T | C | A | C | G | A | G | A | A | G | T | T | G | T | A | T | C | G | G | T | G | A | A | T | A | A |
| PC-5p-39989-25 | - | - | - | - | - | - | - | - | - | - | G | C | T | G | T | C | A | G | C | G | A | C | A | T | T | G | A | A | C | C | T | T | A | T | T | C | A | C | G | G | G | A | A | G | T | T | G | T | A | T | C | G | G | T | G | A | A | T | A | A |
| PC-5p-39989-20 | G | - | - | - | - | - | - | - | - | - | A | C | T | G | T | C | A | C | C | G | A | T | A | T | T | G | A | A | C | C | T | T | A | T | T | C | A | C | G | G | G | A | A | G | T | T | G | T | A | T | C | G | G | T | G | A | A | T | A | A |
| PC-5p-39989-2 | - | - | - | - | - | - | - | - | - | - | - | G | T | G | T | T | A | G | C | G | A | T | A | T | T | G | A | A | C | C | T | T | A | T | T | C | A | C | G | A | T | A | C | T | A | T | G | T | T | C | C | A | G | T | G | A | A | T | A | A |
| PC-5p-39989-18 | - | - | - | - | - | - | - | - | - | - | - | G | T | G | T | T | A | G | C | G | A | T | A | T | T | G | A | A | C | C | T | T | A | T | T | C | A | C | G | A | T | A | C | T | A | T | G | T | T | C | C | A | G | T | G | A | A | T | A | A |
| PC-5p-39989-15 | A | T | T | G | A | A | C | C | T | T | G | G | T | G | T | T | A | G | C | G | A | T | A | T | T | G | A | A | C | C | T | T | A | T | T | C | A | C | A | A | T | A | C | T | A | T | G | T | T | C | C | A | G | T | G | A | A | T | A | A |
| PC-5p-39989-16 | - | - | - | - | - | - | - | - | - | - | - | G | T | G | T | T | A | G | C | G | A | T | A | T | T | G | A | A | C | C | T | T | A | T | T | C | A | C | G | A | T | A | C | T | A | T | G | T | T | C | C | A | G | T | G | A | A | T | A | A |
| PC-5p-39989-21 | - | - | - | - | - | - | - | - | - | - | - | C | T | G | C | T | A | A | C | G | A | T | A | T | T | G | A | A | C | C | T | T | A | T | T | C | A | C | C | A | A | A | A | C | A | A | C | T | T | C | C | T | G | T | G | A | A | T | A | A |
| PC-5p-39989-22 | A | G | A | G | - | - | - | - | - | T | G | C | T | G | T | T | A | T | C | G | A | T | A | T | T | G | A | C | C | A | T | T | A | T | T | C | A | C | T | G | A | A | C | G | T | A | T | T | T | A | G | T | - | T | G | A | A | T | A | A |
| PC-5p-39989-23 | - | - | - | - | - | - | - | - | - | - | - | C | T | G | C | T | A | A | C | G | A | T | A | T | T | G | A | A | C | C | T | T | A | T | T | C | A | A | C | T | A | A | A | T | A | C | G | T | T | C | A | G | - | T | A | A | A | T | A | A |

**┤ Complementary region ends**

| PC-5p-39989-1 | G | G | T | T | C | A | A | T | A | T | C | G | C | T | A | A | C | - | - | A | C | C | G | - | - | - | - | - | - | - | - | - | - | - | - | - | C | T | G | T | G | C | T | G | C | T | - | - | - | - | - | - | - | - | T | T | G | A | T | T |
| --- | --- | --- | --- | --- | --- | --- | --- | --- | --- | --- | --- | --- | --- | --- | --- | --- | --- | --- | --- | --- | --- | --- | --- | --- | --- | --- | --- | --- | --- | --- | --- | --- | --- | --- | --- | --- | --- | --- | --- | --- | --- | --- | --- | --- | --- | --- | --- | --- | --- | --- | --- | --- | --- | --- | --- | --- | --- | --- | --- | --- |
| PC-5p-39989-3 | G | G | T | T | C | A | A | T | A | T | C | G | C | T | A | A | C | - | - | A | C | C | G | - | - | - | - | - | - | - | - | - | - | - | - | - | C | T | G | T | G | C | T | G | C | T | G | T | T | T | T | A | C | T | T | T | G | A | T | T |
| PC-5p-39989-13 | G | G | T | T | T | A | A | T | A | T | C | G | C | T | A | A | C | - | - | A | C | C | G | - | - | - | - | - | - | - | - | - | - | - | - | - | C | T | G | T | G | C | T | G | C | T | G | T | T | T | T | G | C | T | T | C | G | A | T | T |
| PC-5p-39989-24 | G | T | T | T | C | A | A | T | A | T | C | G | C | T | A | A | C | - | - | A | C | C | G | - | - | - | - | - | - | - | - | - | - | - | - | - | C | T | G | T | G | C | T | G | C | T | G | T | T | T | T | G | C | T | T | T | G | A | T | T |
| PC-5p-39989-5 | G | G | T | T | C | A | A | T | A | T | C | G | C | T | A | A | G | - | - | A | C | C | G | - | - | - | - | - | - | - | - | - | - | - | - | - | C | T | G | T | G | C | T | G | C | T | G | T | T | T | T | G | C | T | T | T | G | A | T | T |
| PC-5p-39989-9 | G | G | T | T | C | A | A | T | A | T | C | G | C | T | A | A | C | - | - | A | C | C | A | A | G | G | T | T | C | A | A | T | A | T | C | A | C | T | G | T | G | C | T | G | C | T | G | T | T | T | T | G | C | T | T | T | G | A | T | T |
| PC-5p-39989-4 | G | G | T | T | C | A | A | T | A | T | C | T | C | T | A | A | T | G | T | G | C | C | G | G | T | G | - | - | - | - | - | - | - | - | - | - | - | T | G | T | G | T | T | T | G | C | G | T | G | A | G | A | G | A | A | G | G | T | G | T |
| PC-5p-39989-10 | G | G | T | T | C | A | A | C | A | T | C | G | C | T | A | A | C | - | - | A | C | C | T | C | T | - | - | - | - | - | - | - | - | - | - | - | - | - | - | - | - | - | - | - | - | - | - | - | - | - | - | - | - | - | - | - | - | - | - | - |
| PC-5p-39989-6 | G | G | T | T | C | A | A | T | A | T | C | G | T | T | A | G | C | - | - | A | G | C | G | - | - | - | - | - | - | - | - | - | - | - | - | - | C | T | G | T | G | C | T | G | C | T | A | T | - | - | A | G | C | T | A | T | G | A | A | C |
| PC-5p-39989-7 | G | G | T | T | C | A | A | T | A | T | C | G | T | T | A | G | C | - | - | A | G | C | G | - | - | - | - | - | - | - | - | - | - | - | - | - | C | T | G | T | G | C | T | G | C | T | A | T | - | - | A | G | C | T | A | T | G | A | A | C |
| PC-5p-39989-12 | G | G | T | T | C | A | A | T | A | T | C | G | T | T | A | G | C | - | - | A | G | C | G | - | - | - | - | - | - | - | - | - | - | - | - | - | C | T | G | T | G | C | T | G | C | T | A | T | - | - | A | G | C | T | A | T | G | A | A | C |
| PC-5p-39989-11 | G | G | C | T | C | A | A | T | A | T | C | G | T | T | A | G | C | - | - | A | G | C | G | - | - | - | - | - | - | - | - | - | - | - | - | - | C | T | G | T | G | C | T | G | C | T | A | T | - | - | A | G | C | T | A | T | G | A | A | C |
| PC-5p-39989-8 | G | G | T | T | C | A | A | T | A | T | C | G | T | T | A | G | C | - | - | A | G | C | G | - | - | - | - | - | - | - | - | - | - | - | - | - | C | T | G | T | G | C | T | G | C | T | A | T | - | - | A | G | C | T | A | T | G | A | A | C |
| PC-5p-39989-14 | G | G | T | T | C | A | A | T | A | T | T | G | C | T | A | A | T | - | - | T | G | C | G | - | - | - | - | - | - | - | - | - | - | - | - | - | C | T | G | T | G | C | T | G | C | T | G | T | T | C | T | G | C | T | A | T | - | A | G | C |
| PC-5p-39989-19 | G | G | T | T | C | A | A | T | A | T | T | G | C | T | A | A | C | - | - | T | G | C | G | - | - | - | - | - | - | - | - | - | - | - | - | - | C | C | G | T | G | C | T | G | C | T | G | T | T | C | T | G | C | T | A | T | - | A | G | C |
| PC-5p-39989-17 | G | G | T | T | C | A | A | T | A | T | T | G | C | A | A | A | C | - | - | G | A | C | G | - | - | - | - | - | - | - | - | - | - | - | - | - | C | T | G | T | G | C | T | G | C | T | G | T | T | C | T | G | C | T | A | T | G | A | T | A |
| PC-5p-39989-26 | G | G | T | T | C | A | A | T | A | C | T | G | C | A | A | A | C | - | - | G | A | C | G | - | - | - | - | - | - | - | - | - | - | - | - | - | C | T | G | T | G | C | T | G | C | C | G | T | T | C | T | G | C | T | A | T | G | A | T | A |
| PC-5p-39989-27 | G | G | T | T | C | A | A | T | A | T | T | G | T | T | A | G | C | - | - | G | A | C | G | - | - | - | - | - | - | - | - | - | - | - | - | - | C | T | G | T | G | C | T | G | T | T | T | T | T | T | G | - | G | T | C | A | T | G | T | - |
| PC-5p-39989-28 | G | G | T | T | C | A | A | T | A | T | T | G | T | T | A | G | C | - | - | G | A | C | G | - | - | - | - | - | - | - | - | - | - | - | - | - | C | T | G | T | G | C | T | A | T | T | T | T | T | T | G | - | A | T | C | A | T | G | T | - |
| PC-5p-39989-25 | G | G | T | T | C | A | A | T | A | T | T | G | T | T | A | G | C | - | - | G | A | C | G | - | - | - | - | - | - | - | - | - | - | - | - | - | C | T | G | T | G | C | T | G | T | T | T | T | T | T | G | - | G | T | C | A | T | G | T | - |
| PC-5p-39989-20 | G | G | T | T | C | A | A | A | A | T | T | G | T | T | G | G | C | - | - | G | A | C | G | - | - | - | - | - | - | - | - | - | - | - | - | - | C | T | A | T | G | C | T | G | T | T | T | G | A | G | G | G | A | A | T | G | T | G | T | G |
| PC-5p-39989-2 | G | G | T | T | C | A | A | T | A | T | C | G | C | T | A | A | C | - | - | A | C | C | G | - | - | - | - | - | - | - | - | - | - | - | - | - | C | T | T | T | C | A | T | T | C | C | A | G | - | - | - | A | A | A | A | C | G | C | A | A |
| PC-5p-39989-18 | G | G | T | T | C | A | A | T | A | T | C | G | - | - | - | - | - | - | - | - | C | C | G | - | - | - | - | - | - | - | - | - | - | - | - | - | T | T | T | T | C | A | T | T | G | C | A | G | - | - | - | A | A | A | A | C | G | C | A | A |
| PC-5p-39989-15 | G | G | T | T | C | A | A | T | A | T | T | G | C | T | A | A | C | - | - | A | C | C | G | - | - | - | - | - | - | - | - | - | - | - | - | - | C | T | C | T | C | A | T | T | G | C | A | G | - | - | - | A | A | A | A | C | G | C | A | A |
| PC-5p-39989-16 | T | G | T | T | C | A | A | T | A | T | C | G | C | T | A | A | C | - | - | A | C | C | G | - | - | - | - | - | - | - | - | - | - | - | - | - | C | T | T | T | T | A | T | T | C | C | A | G | - | - | - | A | A | A | A | C | G | C | A | A |
| PC-5p-39989-21 | G | G | T | T | C | A | A | T | A | A | C | A | G | - | - | - | - | - | - | - | - | C | G | - | - | - | - | - | - | - | - | - | - | - | - | - | C | T | T | T | T | A | A | T | C | C | A | G | - | - | - | A | A | A | A | A | C | G | A | A |
| PC-5p-39989-22 | G | G | T | T | C | A | A | T | A | T | C | G | T | T | A | G | C | - | - | A | G | C | G | - | - | - | - | - | - | - | - | - | - | - | - | - | C | T | G | T | G | C | T | G | C | T | A | T | - | - | A | G | C | T | A | T | T | A | C | A |
| PC-5p-39989-23 | G | G | G | T | C | A | A | T | A | T | C | G | A | T | A | A | C | - | - | A | G | C | A | - | - | - | - | - | - | - | - | - | - | - | - | - | C | T | G | T | A | C | C | G | C | T | G | T | G | C | T | G | C | T | A | C | A | C | C | A |

| PC-5p-39989-1 | A | C | G | G | A | G | - | - | A | T | G | T | G | - | T | A | A | A | C | T | T | C | T | T | C | T | T | C | T | T | C | T | T | C | A | T | C | A | T | T | T | A | A | T | T | T | T | C | A | T | G | T | T | T | T | T | T | T | C | T |
| --- | --- | --- | --- | --- | --- | --- | --- | --- | --- | --- | --- | --- | --- | --- | --- | --- | --- | --- | --- | --- | --- | --- | --- | --- | --- | --- | --- | --- | --- | --- | --- | --- | --- | --- | --- | --- | --- | --- | --- | --- | --- | --- | --- | --- | --- | --- | --- | --- | --- | --- | --- | --- | --- | --- | --- | --- | --- | --- | --- | --- |
| PC-5p-39989-3 | T | C | G | G | A | G | - | - | A | T | G | T | G | - | T | A | T | A | C | T | T | C | T | T | C | T | T | C | T | T | C | T | T | C | A | T | C | G | T | T | T | A | A | T | T | T | T | C | A | T | G | T | T | T | T | T | T | T | C | T |
| PC-5p-39989-13 | A | C | G | G | A | G | - | - | A | T | G | T | G | - | T | A | T | A | C | T | T | C | T | T | C | T | - | - | - | T | C | T | T | C | A | T | C | A | T | T | T | A | A | T | T | T | T | C | A | T | G | T | T | T | T | T | T | T | C | T |
| PC-5p-39989-24 | A | C | G | G | A | G | - | - | A | T | G | T | G | - | T | A | T | A | C | T | T | C | T | T | C | T | - | - | - | T | C | T | T | C | A | T | C | A | T | T | T | A | A | T | T | T | T | C | A | G | G | T | T | T | T | T | C | T | C | T |
| PC-5p-39989-5 | A | C | G | G | A | G | - | - | A | T | - | - | - | - | - | - | - | - | - | - | - | - | - | - | - | - | - | - | - | - | - | - | - | - | - | - | - | - | - | - | - | - | - | - | - | - | - | - | - | - | - | - | - | - | - | - | - | - | - | - |
| PC-5p-39989-9 | A | C | G | G | A | G | - | - | A | T | G | T | G | - | T | A | T | A | C | T | T | C | T | T | C | T | - | - | - | T | C | T | T | C | A | T | C | A | T | T | T | A | A | T | T | T | T | C | A | T | G | - | T | T | T | T | T | T | C | T |
| PC-5p-39989-4 | G | T | A | T | C | G | - | - | G | T | G | T | G | - | T | G | T | C | G | G | T | G | T | G | T | G | - | - | - | - | - | T | T | T | G | T | G | T | G | A | T | A | G | A | G | G | G | T | G | T | G | T | G | T | C | G | G | T | G | T |
| PC-5p-39989-10 | - | - | - | - | - | - | - | - | - | - | - | - | - | - | - | - | - | - | - | - | - | - | - | - | - | - | - | - | - | - | - | - | - | - | - | - | - | - | - | - | - | - | - | - | - | - | - | - | - | - | - | - | - | - | - | - | - | - | - | - |
| PC-5p-39989-6 | A | T | T | C | A | T | G | A | T | A | T | T | G | - | T | C | A | A | A | T | T | C | A | T | A | C | A | T | A | C | A | T | A | C | G | C | A | G | C | A | C | A | C | T | C | A | T | C | A | C | T | - | - | - | G | A | C | T | C | A |
| PC-5p-39989-7 | A | T | T | C | A | T | G | A | T | A | T | T | G | - | T | C | A | A | A | T | T | C | A | T | A | C | A | T | A | C | A | T | A | C | G | C | A | G | C | A | C | A | C | T | C | A | T | C | A | C | T | - | - | - | G | A | C | T | C | A |
| PC-5p-39989-12 | A | T | T | C | A | T | G | A | T | A | T | T | G | - | T | C | A | A | A | T | T | C | A | T | A | C | A | T | A | C | A | T | A | C | G | C | A | G | C | A | C | A | C | T | C | A | T | C | A | C | T | - | - | - | G | A | C | T | C | A |
| PC-5p-39989-11 | A | T | T | C | A | T | G | A | T | A | T | T | G | - | T | C | A | A | C | T | T | C | A | T | A | C | A | T | A | C | A | T | A | C | G | C | A | G | C | A | C | A | C | T | C | A | T | C | G | - | - | - | - | - | G | A | C | A | T | G |
| PC-5p-39989-8 | A | T | T | C | G | T | G | A | T | A | T | T | G | - | T | C | A | A | - | T | T | C | A | T | A | C | A | T | A | C | A | T | A | C | G | C | A | G | C | A | C | A | C | T | G | T | T | T | G | A | T | A | G | G | A | A | A | T | A | T |
| PC-5p-39989-14 | T | A | T | G | A | G | - | - | C | A | T | T | G | - | T | C | A | A | A | A | A | C | A | T | A | C | A | T | A | C | A | T | A | C | G | C | A | G | C | A | C | A | C | T | C | A | T | C | A | C | C | - | - | - | G | A | C | T | C | A |
| PC-5p-39989-19 | T | A | T | G | A | G | - | - | C | G | T | T | G | - | T | C | A | A | A | A | A | C | A | T | A | C | A | T | A | C | A | T | A | C | G | C | A | G | C | A | C | A | C | T | C | A | T | C | A | C | C | - | - | - | G | A | C | T | C | A |
| PC-5p-39989-17 | T | T | C | C | A | G | - | - | C | A | T | T | G | - | T | G | A | A | A | T | A | T | A | T | C | A | A | T | A | C | A | T | A | C | G | C | A | G | C | A | C | A | C | T | C | A | T | C | A | C | C | - | - | - | G | A | T | T | C | A |
| PC-5p-39989-26 | T | T | C | C | A | G | - | - | C | A | T | T | G | - | T | G | A | A | A | T | A | C | A | T | C | A | A | T | A | C | A | T | A | C | G | C | A | G | C | A | C | A | C | T | C | A | T | C | A | C | C | - | - | - | G | A | C | T | C | A |
| PC-5p-39989-27 | T | G | A | C | G | G | C | G | G | A | C | A | G | - | - | C | T | G | T | C | G | C | G | T | T | T | A | T | A | T | G | A | A | T | T | T | C | G | T | T | G | T | A | T | T | T | G | C | C | A | A | - | - | - | A | A | T | A | C | T |
| PC-5p-39989-28 | T | G | A | C | A | G | C | G | G | A | C | A | G | - | - | C | T | G | T | C | G | C | G | T | T | T | A | T | A | C | G | A | A | T | T | T | C | G | T | T | G | T | A | T | T | T | G | C | C | A | A | - | - | - | A | A | T | A | C | T |
| PC-5p-39989-25 | T | G | A | C | A | G | C | G | G | A | C | A | G | - | - | C | G | G | T | C | G | C | G | T | T | T | A | T | A | C | G | A | T | T | T | C | T | A | A | T | C | G | A | T | T | A | A | C | G | A | A | - | - | - | A | A | T | T | A | A |
| PC-5p-39989-20 | T | G | T | C | C | G | T | G | T | A | T | G | T | T | T | C | A | G | - | A | G | A | G | G | G | A | A | T | G | T | G | T | G | T | G | T | C | G | T | T | G | T | C | T | G | T | C | G | G | T | G | - | - | - | T | G | T | T | T | G |
| PC-5p-39989-2 | A | A | T | C | T | A | T | G | T | A | C | T | T | T | T | G | T | T | T | T | T | C | G | A | T | T | T | T | T | T | A | T | T | A | A | T | T | T | A | A | A | A | T | T | A | A | A | A | A | A | T | - | - | - | C | A | A | A | A | T |
| PC-5p-39989-18 | A | A | T | C | T | A | T | G | T | A | C | T | T | T | T | G | T | T | T | T | T | C | A | A | T | T | T | T | T | T | A | T | T | A | A | T | T | T | A | A | A | A | T | T | A | A | A | A | A | A | T | - | - | - | C | A | A | A | A | T |
| PC-5p-39989-15 | A | A | T | C | T | A | T | G | T | A | C | T | T | T | T | G | T | T | T | T | T | C | G | A | T | T | T | T | T | T | A | T | T | A | A | T | T | T | A | A | A | A | T | T | G | A | A | A | A | A | T | - | - | - | C | A | A | A | A | T |
| PC-5p-39989-16 | A | - | T | C | T | A | T | G | T | A | C | T | T | T | T | G | T | T | C | T | T | C | G | A | T | T | T | T | T | T | A | T | T | - | A | T | T | T | T | A | A | A | T | T | A | A | A | A | A | A | T | - | - | - | C | A | A | A | A | A |
| PC-5p-39989-21 | A | A | A | C | T | A | C | C | T | A | C | T | T | T | T | T | A | T | T | T | T | C | G | A | T | T | T | T | T | T | A | T | T | A | A | T | T | A | A | A | A | A | - | - | - | A | A | A | A | A | T | - | - | - | T | A | A | A | C | T |
| PC-5p-39989-22 | A | T | T | C | A | A | T | G | T | T | G | T | G | A | A | A | T | T | C | A | T | A | C | A | C | A | C | A | T | A | G | G | C | A | - | - | - | C | C | A | C | A | C | T | C | A | T | C | A | C | C | - | - | - | G | A | C | T | C | A |
| PC-5p-39989-23 | A | T | A | C | A | C | A | T | T | C | A | C | A | A | T | A | C | A | T | G | T | A | T | A | G | T | G | A | A | A | T | T | C | A | T | A | C | C | C | A | C | A | C | T | C | A | T | C | A | T | C | - | - | - | G | A | A | T | C | A |
|  |  |  |  |  |  |  |  |  |  |  |  |  |  |  |  |  |  |  |  |  |  |  |  |  |  |  |  |  |  |  |  |  |  |  |  |  |  |  |  |  |  |  |  |  |  |  |  |  |  |  |  |  |  |  |  |  |  |  |  |  |
| PC-5p-39989-1 | G | T | G | C | A | G | T | A | C | A | G | - | - | - | - | - | T | T | G | T | T | T | T | A | G | T | A | T | C | G | - | - | - | - | - |  |  |  |  |  |  |  |  |  |  |  |  |  |  |  |  |  |  |  |  |  |  |  |  |  |
| PC-5p-39989-3 | G | T | G | C | A | T | T | A | C | A | G | - | - | - | - | - | T | A | G | T | T | T | - | - | - | - | - | - | - | - | - | - | - | - | - |  |  |  |  |  |  |  |  |  |  |  |  |  |  |  |  |  |  |  |  |  |  |  |  |  |
| PC-5p-39989-13 | G | T | G | C | A | T | T | A | C | A | G | - | - | - | - | - | T | A | G | T | T | T | T | G | T | - | - | - | - | - | - | - | - | - | - |  |  |  |  |  |  |  |  |  |  |  |  |  |  |  |  |  |  |  |  |  |  |  |  |  |
| PC-5p-39989-24 | G | T | G | C | A | G | T | A | C | A | G | - | - | - | - | - | T | A | G | T | T | T | T | A | G | T | G | T | C | G | G | T | - | - | - |  |  |  |  |  |  |  |  |  |  |  |  |  |  |  |  |  |  |  |  |  |  |  |  |  |
| PC-5p-39989-5 | - | - | - | - | - | - | - | - | - | - | - | - | - | - | - | - | - | - | - | - | - | - | - | - | - | - | - | - | - | - | - | - | - | - | - |  |  |  |  |  |  |  |  |  |  |  |  |  |  |  |  |  |  |  |  |  |  |  |  |  |
| PC-5p-39989-9 | G | T | G | C | A | G | T | A | - | - | - | - | - | - | - | - | - | - | - | - | - | - | - | - | - | - | - | - | - | - | - | - | - | - | - |  |  |  |  |  |  |  |  |  |  |  |  |  |  |  |  |  |  |  |  |  |  |  |  |  |
| PC-5p-39989-4 | G | T | G | T | T | T | G | A | T | G | G | - | - | - | - | - | A | A | A | A | T | A | T | - | - | - | - | - | - | - | - | - | - | - | - |  |  |  |  |  |  |  |  |  |  |  |  |  |  |  |  |  |  |  |  |  |  |  |  |  |
| PC-5p-39989-10 | - | - | - | - | - | - | - | - | - | - | - | - | - | - | - | - | - | - | - | - | - | - | - | - | - | - | - | - | - | - | - | - | - | - | - |  |  |  |  |  |  |  |  |  |  |  |  |  |  |  |  |  |  |  |  |  |  |  |  |  |
| PC-5p-39989-6 | G | T | G | A | G | A | A | T | C | G | - | - | - | - | - | - | T | T | C | G | C | G | C | G | C | T | - | - | - | - | - | - | - | - | - |  |  |  |  |  |  |  |  |  |  |  |  |  |  |  |  |  |  |  |  |  |  |  |  |  |
| PC-5p-39989-7 | G | T | G | A | G | A | A | T | C | G | - | - | - | - | - | - | T | T | C | G | C | G | C | G | C | T | C | G | A | T | G | A | G | C | C |  |  |  |  |  |  |  |  |  |  |  |  |  |  |  |  |  |  |  |  |  |  |  |  |  |
| PC-5p-39989-12 | G | T | G | A | G | A | A | T | C | G | - | - | - | - | - | - | T | A | A | A | T | A | T | - | T | T | C | - | - | - | - | - | - | - | - |  |  |  |  |  |  |  |  |  |  |  |  |  |  |  |  |  |  |  |  |  |  |  |  |  |
| PC-5p-39989-11 | G | - | G | A | G | C | A | A | C | T | - | - | - | - | - | - | T | A | A | C | T | T | T | T | G | C | C | T | - | - | - | - | - | - | - |  |  |  |  |  |  |  |  |  |  |  |  |  |  |  |  |  |  |  |  |  |  |  |  |  |
| PC-5p-39989-8 | G | T | G | T | G | T | G | T | C | G | G | - | - | - | - | - | T | G | T | G | T | G | T | - | - | - | - | - | - | - | - | - | - | - | - |  |  |  |  |  |  |  |  |  |  |  |  |  |  |  |  |  |  |  |  |  |  |  |  |  |
| PC-5p-39989-14 | G | T | G | A | G | A | A | T | C | G | - | - | - | - | - | - | T | A | T | A | T | T | T | C | G | C | G | - | - | - | - | - | - | - | - |  |  |  |  |  |  |  |  |  |  |  |  |  |  |  |  |  |  |  |  |  |  |  |  |  |
| PC-5p-39989-19 | G | T | G | A | G | A | A | T | C | G | - | - | - | - | - | - | T | A | T | A | T | T | T | C | G | C | G | C | G | C | T | C | A | C | T |  |  |  |  |  |  |  |  |  |  |  |  |  |  |  |  |  |  |  |  |  |  |  |  |  |
| PC-5p-39989-17 | G | T | G | A | G | A | A | T | C | G | - | - | - | - | - | - | T | A | T | A | T | T | T | C | G | T | - | - | - | - | - | - | - | - | - |  |  |  |  |  |  |  |  |  |  |  |  |  |  |  |  |  |  |  |  |  |  |  |  |  |
| PC-5p-39989-26 | G | T | G | A | G | A | A | T | C | G | - | - | - | - | - | - | T | A | T | A | T | T | T | C | G | T | G | T | G | C | - | - | - | - | - |  |  |  |  |  |  |  |  |  |  |  |  |  |  |  |  |  |  |  |  |  |  |  |  |  |
| PC-5p-39989-27 | A | T | T | T | G | G | T | T | A | T | A | - | - | - | - | - | C | A | C | A | T | A | A | T | C | - | - | - | - | - | - | - | - | - | - |  |  |  |  |  |  |  |  |  |  |  |  |  |  |  |  |  |  |  |  |  |  |  |  |  |
| PC-5p-39989-28 | A | T | T | T | G | G | T | T | A | T | A | - | - | - | - | - | C | A | C | A | T | A | A | T | C | G | - | - | - | - | - | - | - | - | - |  |  |  |  |  |  |  |  |  |  |  |  |  |  |  |  |  |  |  |  |  |  |  |  |  |
| PC-5p-39989-25 | A | A | A | T | - | - | T | T | C | C | G | - | - | - | - | - | A | A | A | A | A | T | T | T | C | G | T | T | T | T | - | - | - | - | - |  |  |  |  |  |  |  |  |  |  |  |  |  |  |  |  |  |  |  |  |  |  |  |  |  |
| PC-5p-39989-20 | A | G | A | T | - | - | - | - | - | A | G | - | - | - | - | - | A | G | A | A | T | G | T | G | T | G | - | - | - | - | - | - | - | - | - |  |  |  |  |  |  |  |  |  |  |  |  |  |  |  |  |  |  |  |  |  |  |  |  |  |
| PC-5p-39989-2 | T | C | G | G | A | A | A | C | T | - | - | - | - | - | - | G | C | A | C | A | T | C | T | A | C | A | - | - | - | - | - | - | - | - | - |  |  |  |  |  |  |  |  |  |  |  |  |  |  |  |  |  |  |  |  |  |  |  |  |  |
| PC-5p-39989-18 | T | C | G | G | A | A | A | C | T | T | T | A | C | - | T | G | C | A | C | A | T | C | T | A | C | A | A | - | - | - | - | - | - | - | - |  |  |  |  |  |  |  |  |  |  |  |  |  |  |  |  |  |  |  |  |  |  |  |  |  |
| PC-5p-39989-15 | T | C | G | G | A | A | A | C | T | - | - | - | - | - | - | - | C | T | A | C | T | G | C | A | C | A | - | - | - | - | - | - | - | - | - |  |  |  |  |  |  |  |  |  |  |  |  |  |  |  |  |  |  |  |  |  |  |  |  |  |
| PC-5p-39989-16 | T | C | G | G | A | A | A | T | T | - | - | - | - | - | - | - | T | T | A | C | T | G | C | A | C | A | T | T | - | - | - | - | - | - | - |  |  |  |  |  |  |  |  |  |  |  |  |  |  |  |  |  |  |  |  |  |  |  |  |  |
| PC-5p-39989-21 | T | C | G | G | A | A | A | C | A | C | A | T | T | G | T | G | C | A | C | A | T | C | T | A | C | A | G | C | A | C | - | - | - | - | - |  |  |  |  |  |  |  |  |  |  |  |  |  |  |  |  |  |  |  |  |  |  |  |  |  |
| PC-5p-39989-22 | G | T | G | A | G | A | A | T | C | G | - | - | - | - | - | T | A | T | A | T | A | T | T | T | C | G | C | G | C | G | - | - | - | - | - |  |  |  |  |  |  |  |  |  |  |  |  |  |  |  |  |  |  |  |  |  |  |  |  |  |
| PC-5p-39989-23 | G | T | G | A | A | A | A | C | T | G | - | - | - | - | - | T | A | T | A | A | A | C | T | A | C | - | - | - | - | - | - | - | - | - | - |  |  |  |  |  |  |  |  |  |  |  |  |  |  |  |  |  |  |  |  |  |  |  |  |  |

**E: The 54311 family**

| PC-3p-54311-5 | - | - | G | T | C | C | T | T | G | C | T | C | A | G | T | A | A | A | A | A | A | A | A | A | A | A | T | T | G | A | C | G | T | T | A | A | A | C | G | C | A | C | T | - | - | C | A | T | T | T | C | G | A | C | T | A | G | - | - | - |
| --- | --- | --- | --- | --- | --- | --- | --- | --- | --- | --- | --- | --- | --- | --- | --- | --- | --- | --- | --- | --- | --- | --- | --- | --- | --- | --- | --- | --- | --- | --- | --- | --- | --- | --- | --- | --- | --- | --- | --- | --- | --- | --- | --- | --- | --- | --- | --- | --- | --- | --- | --- | --- | --- | --- | --- | --- | --- | --- | --- | --- |
| PC-3p-54311-46 | - | T | A | A | T | A | A | T | G | C | C | C | A | G | T | A | A | A | A | A | A | A | A | A | T | T | T | T | - | A | C | G | T | T | A | A | A | C | G | C | A | C | T | - | - | C | A | T | T | T | T | G | A | C | T | A | G | - | - | - |
| PC-3p-54311-7 | - | - | - | - | - | - | - | - | - | - | - | - | - | - | - | - | - | A | A | A | A | A | A | A | A | T | T | T | G | A | C | G | T | T | A | A | A | C | G | C | A | C | T | - | - | C | A | T | T | T | C | G | A | C | T | A | C | - | - | - |
| PC-3p-54311-39 | - | - | - | - | - | - | - | - | - | - | - | - | - | - | - | - | A | A | A | A | A | A | A | A | A | T | T | T | G | A | C | G | T | T | A | A | A | C | G | C | A | C | T | - | - | C | A | T | T | T | C | G | A | C | T | A | G | - | - | - |
| PC-3p-54311-66 | - | - | - | - | - | - | - | - | - | - | - | - | - | - | - | - | - | A | A | A | A | A | G | A | A | T | T | T | G | A | C | G | T | T | A | A | A | C | G | C | A | C | T | - | - | C | A | T | T | T | C | G | A | C | C | A | G | - | - | - |
| PC-3p-54311-51 | - | - | - | - | - | - | - | - | - | - | - | - | - | - | - | - | - | G | A | A | A | A | A | A | A | A | T | T | G | A | C | G | T | A | A | A | A | A | G | T | A | G | T | - | - | C | A | T | T | T | C | G | A | C | T | T | G | - | - | - |
| PC-3p-54311-59 | - | - | - | - | - | - | - | - | - | - | - | - | - | - | - | - | - | A | G | T | G | T | T | G | A | T | T | T | G | A | C | G | T | T | A | A | A | C | G | C | A | C | T | - | - | C | A | T | T | T | C | G | A | C | T | A | G | - | - | - |
| PC-3p-54311-43 | - | - | - | - | - | - | - | - | - | - | - | - | - | - | - | - | - | A | A | A | A | A | A | A | A | T | T | G | A | A | C | G | T | T | A | A | A | C | G | C | A | C | T | - | - | C | A | T | T | T | C | G | A | C | A | A | G | - | - | - |
| PC-3p-54311-48 | - | - | - | - | - | - | - | - | - | - | - | - | - | - | - | - | - | A | A | A | A | A | G | A | A | C | T | T | G | A | C | G | G | T | A | A | A | C | G | C | A | C | C | - | - | C | A | T | T | T | C | G | A | C | T | A | G | - | - | - |
| PC-3p-54311-53 | - | - | - | - | - | - | - | - | - | - | - | - | - | - | - | - | - | - | A | A | A | A | A | A | A | A | T | T | G | A | C | G | G | T | A | A | A | T | G | C | A | C | T | - | - | C | A | T | T | T | C | G | A | C | T | A | G | - | - | - |
| PC-3p-54311-62 | - | - | - | - | - | - | - | - | - | - | - | - | - | - | - | - | - | - | - | - | - | - | - | - | A | T | T | T | G | A | C | G | T | T | A | A | A | C | G | C | A | C | T | - | - | C | A | T | T | T | C | G | A | C | T | A | G | - | - | - |
| PC-3p-54311-58 | - | - | - | - | - | - | - | - | - | - | - | - | - | - | - | - | - | T | A | A | A | A | A | A | A | T | T | T | G | A | C | G | T | T | A | A | A | C | G | C | A | C | T | - | - | C | A | T | T | T | C | G | A | C | T | A | G | - | - | - |
| PC-3p-54311-52 | - | - | - | - | - | - | - | - | - | - | - | - | - | - | - | - | - | A | A | A | A | A | A | A | A | A | A | T | G | A | C | G | T | T | A | A | A | C | G | C | A | C | T | - | - | C | A | T | T | T | C | G | A | C | T | A | G | - | - | - |
| PC-3p-54311-56 | - | - | - | - | - | - | - | - | - | - | - | - | - | - | - | - | - | T | A | A | A | A | A | A | A | A | T | T | A | A | C | G | T | T | A | A | A | C | G | C | A | C | T | - | - | C | A | T | T | T | C | G | A | A | T | A | G | - | - | - |
| PC-3p-54311-13 | - | - | - | - | - | - | - | - | - | - | - | - | - | - | - | - | - | - | - | - | - | - | - | - | A | A | T | T | G | A | C | G | T | T | C | A | A | C | G | C | A | C | T | - | - | C | A | T | T | T | T | G | A | C | T | A | G | - | - | - |
| PC-3p-54311-33 | - | - | - | - | - | - | - | - | - | - | - | - | - | - | - | - | - | - | - | - | - | - | - | - | - | - | - | - | - | - | - | - | - | - | - | - | - | - | - | - | - | - | - | - | - | - | A | T | T | T | C | G | A | C | T | A | G | - | - | - |
| PC-3p-54311-25 | - | - | - | - | - | - | - | - | - | - | - | - | - | - | - | - | - | T | A | A | A | A | A | A | A | A | A | T | T | G | C | T | G | T | T | A | A | C | A | A | A | C | T | - | - | C | A | T | T | T | T | G | A | C | T | T | G | - | - | - |
| PC-3p-54311-34 | - | - | - | - | - | - | - | - | - | - | - | - | - | - | - | - | - | - | - | - | - | - | A | A | A | A | T | T | T | G | C | T | T | T | T | A | A | C | A | A | A | C | T | - | - | C | A | T | T | T | C | G | A | C | T | A | G | - | - | - |
| PC-3p-54311-54 | - | - | - | - | - | - | - | - | - | - | - | - | - | - | - | - | - | - | A | A | A | A | A | A | A | A | T | T | T | G | C | T | T | T | T | A | A | C | A | A | A | C | T | - | - | C | A | T | T | T | C | G | A | C | A | G | G | - | - | - |
| PC-3p-54311-8 | - | - | - | - | - | - | - | - | - | - | - | - | - | - | - | - | - | A | A | A | A | A | A | A | A | A | T | T | T | G | C | T | T | T | T | A | A | A | A | A | A | C | T | - | - | C | A | T | T | T | C | G | A | C | T | T | G | - | - | - |
| PC-3p-54311-32 | - | - | - | - | - | - | - | - | - | - | - | - | - | - | - | - | - | - | - | - | - | - | - | - | - | - | - | T | T | G | C | T | T | T | T | A | A | C | A | A | A | C | T | - | - | C | A | T | T | T | C | G | A | C | T | A | G | - | - | - |
| PC-3p-54311-57 | - | - | - | - | - | - | - | - | - | - | - | - | - | - | - | - | A | G | T | G | A | A | A | A | A | A | A | T | T | G | C | T | T | T | T | A | A | C | A | A | A | C | T | - | - | C | A | T | T | T | C | C | A | C | T | T | G | - | - | - |
| PC-3p-54311-44 | - | - | - | - | - | - | - | - | - | - | - | - | - | - | - | - | - | A | A | A | A | A | A | A | A | A | A | T | T | G | C | T | T | T | T | A | A | C | A | A | A | C | T | - | - | C | A | T | T | T | C | G | A | C | T | T | G | - | - | - |
| PC-3p-54311-26 | - | - | - | - | - | - | - | - | - | - | - | - | - | - | - | - | - | A | A | A | A | A | A | A | A | A | A | T | T | C | C | T | T | T | T | A | A | C | A | T | A | C | T | - | - | C | A | T | T | T | C | G | A | C | T | T | G | - | - | - |
| PC-3p-54311-61 | - | - | - | - | - | - | - | - | - | - | - | - | - | - | - | - | - | - | - | - | - | - | - | - | A | A | T | T | T | G | C | T | T | T | T | A | A | C | A | A | A | C | T | - | - | A | A | T | T | T | C | G | A | C | T | A | G | - | - | - |
| PC-3p-54311-47 | G | A | A | A | A | C | C | C | A | C | T | C | A | G | T | G | - | - | A | A | A | T | A | A | A | A | T | T | T | G | C | T | T | T | T | A | A | C | A | A | A | C | T | - | - | C | A | T | T | T | C | G | A | C | T | T | G | - | - | - |
| PC-3p-54311-63 | G | T | G | A | A | A | G | T | A | C | T | C | A | G | T | G | - | - | A | A | A | A | A | A | G | A | T | T | T | G | C | T | T | T | T | A | A | C | A | A | A | C | T | - | - | C | A | T | T | T | C | G | A | C | T | T | G | - | - | - |
| PC-3p-54311-60 | T | A | T | C | T | A | C | T | G | C | T | C | A | G | T | A | A | - | A | A | A | A | A | A | A | A | C | T | T | G | C | T | T | T | T | A | A | A | A | A | A | A | - | - | - | - | - | - | - | - | C | G | - | - | - | - | - | - | - | - |
| PC-3p-54311-38 | - | - | - | T | G | C | T | T | T | C | T | C | A | G | T | G | A | A | A | A | A | A | A | A | A | A | T | T | T | G | C | C | T | T | T | A | A | A | A | A | A | C | T | - | - | C | A | T | T | T | C | G | A | C | T | T | G | - | - | - |
| PC-3p-54311-17 | - | - | - | - | - | - | - | - | - | - | - | - | - | - | - | - | A | A | A | A | A | A | A | A | A | A | A | T | G | A | C | G | T | A | A | A | A | A | G | T | A | G | - | - | T | C | A | T | T | T | C | G | A | C | T | T | G | - | - | - |
| PC-3p-54311-36 | - | - | - | - | - | - | - | - | - | - | - | - | - | - | - | - | - | - | - | A | A | A | A | A | A | A | T | T | G | T | C | G | A | A | A | T | G | A | C | T | A | C | T | T | T | C | A | T | T | T | C | G | A | C | T | T | G | - | - | - |
| PC-3p-54311-12 | - | - | - | - | - | - | - | - | - | - | - | - | - | - | - | - | - | G | A | A | A | A | A | A | A | A | T | T | T | A | C | G | T | A | A | A | A | A | G | T | A | G | - | - | T | C | A | T | T | T | C | G | A | C | T | T | G | - | - | - |
| PC-3p-54311-3 | - | - | - | - | - | - | - | - | - | - | - | - | - | - | - | - | - | - | - | - | - | - | A | A | A | A | T | T | G | A | C | G | T | T | A | A | A | A | G | T | A | C | T | - | - | C | A | T | T | T | C | G | A | C | T | A | G | - | - | - |
| PC-3p-54311-10 | - | - | - | - | - | - | - | - | - | - | - | - | - | - | - | - | G | T | A | A | A | A | A | A | A | A | T | T | G | A | C | G | T | T | A | A | A | A | G | T | A | C | T | - | - | C | A | T | T | T | C | G | A | C | A | A | G | - | - | - |
| PC-3p-54311-22 | - | - | - | - | - | - | - | - | - | - | - | - | - | - | - | - | - | T | C | T | A | C | C | T | A | A | T | T | G | A | C | G | T | T | A | A | A | A | G | T | A | C | T | - | - | T | A | T | T | T | C | G | A | C | T | A | G | - | - | - |
| PC-3p-54311-49 | - | - | - | - | - | - | - | - | - | - | - | - | - | - | - | - | - | A | A | A | A | A | A | A | A | A | T | T | G | A | C | G | T | T | A | A | A | A | G | T | A | C | T | - | - | C | A | T | T | T | C | G | A | C | T | A | G | - | - | - |
| PC-3p-54311-1 | - | - | - | - | - | - | - | - | - | - | - | - | - | - | - | - | - | A | T | C | G | A | A | A | A | A | T | T | G | A | C | G | T | T | A | A | A | A | G | T | A | C | T | - | - | C | A | T | T | T | C | G | A | C | C | A | G | - | - | - |
| PC-3p-54311-55 | - | - | - | - | - | - | - | - | - | - | - | - | - | - | - | - | - | A | A | A | A | A | A | A | A | A | T | G | G | A | C | G | T | T | A | A | A | A | G | T | A | C | T | - | - | C | A | T | T | T | C | G | A | C | T | A | G | - | - | - |
| PC-3p-54311-2 | - | - | - | - | - | - | - | - | - | - | - | - | - | - | - | - | - | A | A | A | A | A | A | A | A | A | T | T | G | A | C | G | T | T | A | A | A | A | G | T | A | C | T | - | - | C | A | T | T | T | C | G | A | C | C | A | G | - | - | - |
| PC-3p-54311-9 | - | - | - | - | - | - | - | - | - | - | - | - | - | - | - | - | - | T | A | A | A | A | A | A | A | A | T | T | G | A | C | G | T | T | A | A | A | A | G | T | A | C | T | - | - | T | A | T | T | T | C | G | A | C | T | A | G | - | - | - |
| PC-3p-54311-20 | - | - | - | - | - | - | - | - | - | - | - | - | - | - | - | - | - | A | A | A | A | A | A | A | A | A | T | T | G | A | C | G | T | T | A | A | A | A | G | T | A | C | T | - | - | T | A | T | T | T | C | G | A | C | T | A | G | - | - | - |
| PC-3p-54311-18 | - | - | - | - | - | - | - | - | - | - | - | - | - | - | - | - | - | A | A | A | A | A | A | A | A | A | T | T | G | A | C | G | T | T | A | A | A | A | G | T | A | C | T | - | - | C | A | T | T | T | C | G | A | C | T | A | G | - | - | - |
| PC-3p-54311-37 | - | - | - | - | - | - | - | - | - | - | - | - | - | - | - | - | - | - | A | A | A | A | A | A | A | A | T | T | G | A | C | G | T | A | A | A | A | A | G | T | A | G | T | - | - | C | A | T | T | T | C | G | A | C | T | A | G | - | - | - |
| PC-3p-54311-45 | - | - | - | - | - | - | - | - | - | - | - | - | - | - | - | - | - | A | A | A | A | A | A | A | A | A | T | T | G | A | C | G | T | T | A | A | A | A | G | T | A | C | T | - | - | C | A | T | T | T | C | G | A | C | T | T | G | - | - | - |
| PC-3p-54311-35 | - | - | - | - | - | - | - | - | - | - | - | - | - | - | - | - | - | - | - | - | A | A | A | A | A | A | T | T | G | A | C | G | T | T | A | A | A | A | G | T | A | C | T | - | - | C | A | T | T | T | C | G | A | C | T | A | C | T | A | C |
| PC-3p-54311-41 | - | - | - | - | - | T | A | C | A | T | A | C | C | C | A | G | T | A | A | A | A | A | A | A | A | A | T | T | G | A | C | G | T | T | A | A | A | A | G | T | A | C | T | - | - | C | A | T | T | T | C | G | A | C | T | A | G | - | - | - |
| PC-3p-54311-40 | - | - | - | - | - | - | - | - | - | - | - | - | - | - | - | - | - | A | A | A | A | A | A | A | A | A | T | T | G | A | C | G | T | T | A | A | G | A | G | T | A | C | T | - | - | C | A | T | T | T | C | G | A | C | T | A | G | - | - | - |
| PC-3p-54311-4 | - | - | - | - | - | - | - | - | - | - | - | - | - | - | - | - | - | A | A | A | A | A | A | A | A | A | T | T | G | A | C | G | T | T | A | A | A | A | G | T | A | C | T | - | - | C | A | T | T | T | C | G | A | C | T | T | G | - | - | - |
| PC-3p-54311-42 | - | - | - | - | - | - | - | - | - | - | - | - | - | - | - | - | - | A | A | A | A | A | A | A | A | A | T | T | G | T | C | G | T | T | A | A | A | A | G | T | A | C | T | - | - | C | A | T | T | T | C | G | A | C | T | A | G | - | - | - |
| PC-3p-54311-16 | - | - | - | - | - | - | - | - | - | - | - | - | - | - | - | - | - | A | A | A | A | A | A | A | A | A | T | T | G | A | C | G | T | T | A | A | A | A | G | T | A | C | T | - | - | T | A | T | T | C | C | G | A | C | T | A | G | - | - | - |
| PC-3p-54311-27 | - | - | - | - | - | - | - | - | - | - | - | - | - | - | - | - | - | A | A | A | A | A | A | A | A | A | T | T | G | A | C | G | T | T | A | A | A | A | G | A | A | C | T | - | - | C | A | T | T | T | C | G | A | C | T | A | G | - | - | - |
| PC-3p-54311-15 | - | - | - | - | - | - | - | - | - | - | - | - | - | - | - | - | T | A | G | A | A | A | A | A | A | A | T | T | G | A | C | G | T | T | A | A | A | G | - | T | A | C | T | - | - | C | A | T | T | T | C | G | A | C | T | A | G | - | - | - |
| PC-3p-54311-14 | - | - | - | - | - | - | - | - | - | - | - | - | - | - | - | - | - | T | A | A | A | A | A | A | A | A | A | T | G | A | C | G | A | T | A | A | G | A | G | T | A | C | T | - | - | C | A | T | T | T | C | G | A | C | T | A | G | - | - | - |
| PC-3p-54311-21 | - | - | - | - | - | - | - | - | - | - | - | - | - | - | - | - | - | A | A | A | A | A | A | A | A | A | T | T | G | A | C | G | T | T | A | A | A | A | G | T | A | C | T | - | - | T | A | T | T | T | C | G | A | C | T | A | G | - | - | - |
| PC-3p-54311-24 | - | - | - | - | - | - | - | - | - | - | - | - | - | - | - | - | - | A | A | G | A | A | A | A | A | A | T | T | G | A | C | G | T | T | A | A | A | A | G | T | A | C | T | - | - | C | A | T | T | T | C | G | A | C | T | A | G | - | - | - |
| PC-3p-54311-65 | - | - | - | - | - | - | - | - | - | - | - | - | - | - | - | - | - | A | A | A | A | A | G | A | A | A | T | T | G | A | C | G | T | T | A | A | A | A | G | T | A | C | T | - | - | T | A | T | T | T | C | G | A | C | T | A | G | - | - | - |
| PC-3p-54311-28 | - | - | - | - | - | - | - | - | - | - | - | - | - | - | - | - | - | T | A | A | A | A | A | A | A | A | T | T | G | A | C | G | T | T | A | A | A | A | G | T | A | C | T | - | - | T | A | T | T | T | C | G | A | C | T | A | G | - | - | - |
| PC-3p-54311-50 | - | - | - | - | - | - | - | - | - | - | - | - | - | - | - | - | - | - | A | A | A | A | A | A | A | A | T | T | G | A | C | G | T | T | A | A | A | A | G | T | A | C | T | - | - | C | A | T | T | T | C | G | A | C | T | A | G | - | - | - |
| PC-3p-54311-30 | - | - | - | - | - | - | - | - | - | - | - | - | - | - | - | - | - | - | - | - | - | - | - | - | - | - | - | - | - | - | - | - | - | - | - | - | - | A | G | T | A | C | T | - | - | T | A | T | T | T | C | G | A | C | T | A | G | - | - | - |
| PC-3p-54311-11 | - | - | - | - | - | - | - | - | - | - | - | - | - | - | - | - | - | - | A | A | A | A | A | A | A | T | T | T | G | A | C | G | T | T | A | A | A | C | G | C | A | C | T | - | - | C | A | T | T | T | C | G | A | C | T | A | G | - | - | - |
| PC-3p-54311-19 | - | - | - | - | - | - | - | - | - | - | - | - | - | - | - | - | - | A | A | G | G | C | A | T | A | A | G | G | C | A | A | T | T | T | A | A | A | A | A | A | A | A | T | - | - | A | A | T | A | T | A | T | A | C | T | C | A | G | - | - |
| PC-3p-54311-23 | - | - | - | - | - | - | - | - | - | - | - | - | - | - | - | - | - | - | - | - | G | A | G | G | A | T | T | T | G | T | A | - | A | G | T | C | C | A | T | T | T | C | A | G | - | A | T | A | C | T | C | A | G | T | A | A | A | A | A | A |
| PC-3p-54311-29 | - | - | - | - | - | - | - | - | - | - | - | - | - | - | - | - | - | - | A | T | C | A | C | A | A | T | T | A | G | A | A | - | A | T | G | T | C | T | T | T | G | G | T | G | - | A | A | A | A | T | C | G | - | - | A | A | A | T | A | T |
| PC-3p-54311-31 | - | - | - | - | - | - | - | - | - | - | - | - | - | - | - | - | - | - | A | T | C | T | T | G | A | A | T | A | T | T | A | G | A | A | A | T | A | T | T | A | G | C | A | A | - | A | T | A | T | T | A | G | C | G | C | T | C | A | T | C |
| PC-3p-54311-6 | - | - | - | - | - | - | - | - | - | - | - | - | - | - | - | - | - | - | A | A | C | T | T | T | C | A | T | T | G | A | T | A | T | A | C | A | A | T | A | T | A | C | A | G | T | A | C | A | T | A | C | T | C | A | T | T | A | A | - | - |
| PC-3p-54311-64 | - | - | - | - | - | - | - | - | - | - | - | C | G | A | T | T | T | T | A | T | T | A | A | A | A | G | G | T | G | A | A | T | A | T | T | A | T | G | G | C | A | C | C | A | - | T | T | T | T | A | C | T | C | A | G | T | G | - | - | - |

**├ Complementary region starts**

| PC-3p-54311-5 | T | C | A | A | A | G | T | G | G | T | A | A | T | A | T | C | G | G | A | C | A | C | G | A | C | T | - | - | - | - | - | - | - | T | C | A | G | A | A | C | A | G | T | C | G | A | C | T | - | - | - | - | A | C | G | T | T | G | A | T |
| --- | --- | --- | --- | --- | --- | --- | --- | --- | --- | --- | --- | --- | --- | --- | --- | --- | --- | --- | --- | --- | --- | --- | --- | --- | --- | --- | --- | --- | --- | --- | --- | --- | --- | --- | --- | --- | --- | --- | --- | --- | --- | --- | --- | --- | --- | --- | --- | --- | --- | --- | --- | --- | --- | --- | --- | --- | --- | --- | --- | --- |
| PC-3p-54311-46 | T | C | A | A | A | G | T | C | C | A | A | A | T | A | T | C | G | G | A | C | A | C | G | G | C | T | - | - | - | - | - | - | - | T | C | A | G | A | A | C | A | G | T | C | G | A | C | T | - | - | - | - | A | C | G | T | T | A | A | T |
| PC-3p-54311-7 | T | C | A | A | A | G | T | C | G | A | A | A | T | A | T | C | G | G | A | C | A | C | G | A | C | T | - | - | - | - | - | - | - | T | C | A | G | A | A | C | A | G | T | C | G | A | C | T | - | - | - | - | A | C | G | T | T | A | A | T |
| PC-3p-54311-39 | T | C | A | A | A | G | T | C | G | A | A | A | T | A | T | C | G | G | A | C | A | C | G | A | C | T | - | - | - | - | - | - | - | T | C | A | G | A | A | C | A | G | T | C | G | A | G | T | - | - | - | - | A | C | G | T | T | A | A | T |
| PC-3p-54311-66 | T | C | A | A | A | G | T | T | G | A | A | A | T | A | T | C | G | G | A | C | A | C | G | A | C | T | - | - | - | - | - | - | - | T | C | A | G | A | A | C | A | G | T | C | G | A | C | T | - | - | - | - | A | C | G | T | T | A | A | A |
| PC-3p-54311-51 | T | C | A | A | A | G | T | C | G | A | A | A | T | A | T | C | G | G | A | C | A | C | G | A | C | T | - | - | - | - | - | - | - | T | C | A | G | A | A | C | A | G | T | C | G | A | C | T | - | - | - | - | G | C | G | T | T | G | A | T |
| PC-3p-54311-59 | T | C | A | A | A | G | T | C | G | A | A | A | T | A | T | C | G | G | A | C | A | C | G | A | A | T | - | - | - | - | - | - | - | T | C | A | G | G | A | C | A | G | T | C | G | A | C | T | - | - | - | - | A | C | G | T | T | A | A | T |
| PC-3p-54311-43 | T | C | A | A | A | G | T | C | G | A | A | A | T | A | T | C | G | G | A | C | A | C | G | A | C | T | - | - | - | - | - | - | - | T | C | A | G | A | A | C | A | G | T | C | G | A | C | T | - | - | - | - | A | C | G | T | T | A | A | T |
| PC-3p-54311-48 | T | C | A | A | A | G | T | C | G | A | A | A | T | A | T | C | G | G | A | C | A | A | G | A | C | T | - | - | - | - | - | - | - | T | C | A | G | A | A | C | A | G | T | C | G | A | C | T | - | - | - | - | A | C | G | T | T | A | A | T |
| PC-3p-54311-53 | T | C | A | A | A | G | T | T | G | A | A | A | T | A | T | C | G | G | A | C | A | C | G | A | C | T | - | - | - | - | - | - | - | T | C | A | G | A | A | C | A | G | T | C | G | A | C | T | - | - | - | - | A | C | G | T | T | A | A | T |
| PC-3p-54311-62 | T | C | A | A | A | G | T | C | G | A | A | A | T | A | T | C | G | G | A | C | A | C | G | A | T | T | - | - | - | - | - | - | - | T | C | A | G | A | A | C | A | G | T | C | G | A | C | T | - | - | - | - | A | C | G | T | T | A | A | T |
| PC-3p-54311-58 | T | C | A | A | A | G | T | C | A | A | A | A | T | A | T | C | G | G | A | C | A | C | G | A | T | T | - | - | - | - | - | - | - | T | C | A | G | A | A | C | A | G | T | C | G | G | T | T | - | - | - | - | A | C | G | T | T | A | A | T |
| PC-3p-54311-52 | T | C | A | A | A | G | T | C | G | A | A | A | T | A | T | C | G | G | A | G | A | C | G | A | C | T | - | - | - | - | - | - | - | T | C | A | G | A | A | C | A | G | T | C | G | A | C | T | - | - | - | - | A | C | G | T | T | T | A | T |
| PC-3p-54311-56 | T | C | A | A | A | G | T | C | G | A | A | A | T | A | T | C | G | G | A | C | A | C | G | A | C | T | - | - | - | - | - | - | - | A | C | A | G | A | A | C | A | T | T | C | G | A | C | T | - | - | - | - | A | C | G | T | T | A | A | T |
| PC-3p-54311-13 | T | C | A | A | A | G | T | C | G | T | A | A | T | A | T | C | G | G | A | C | A | C | G | A | C | T | - | - | - | - | - | - | - | T | C | A | G | A | A | C | A | G | T | C | G | A | C | T | - | - | - | - | A | T | G | T | T | A | A | T |
| PC-3p-54311-33 | T | C | A | A | A | G | T | C | G | A | A | A | T | A | T | C | G | G | A | C | A | C | G | A | C | T | - | - | - | - | - | - | - | T | C | A | G | A | A | C | A | G | T | C | G | A | C | T | - | - | - | - | A | C | G | T | T | A | A | T |
| PC-3p-54311-25 | G | C | A | A | A | G | T | C | G | A | A | A | T | A | T | C | G | G | A | C | A | C | G | A | C | T | - | - | - | - | - | - | - | T | C | A | G | A | A | C | A | G | T | C | G | A | C | T | - | - | - | - | A | T | G | T | T | A | A | T |
| PC-3p-54311-34 | T | C | A | A | A | G | T | C | G | A | A | A | T | A | T | C | G | A | A | C | A | C | G | A | C | A | G | - | - | - | - | - | T | T | C | A | G | A | A | C | A | G | T | C | G | A | C | T | - | - | - | - | A | T | G | T | T | A | A | T |
| PC-3p-54311-54 | T | C | A | A | A | G | T | C | G | A | A | A | T | A | T | C | G | G | A | C | A | C | G | A | C | T | - | - | - | - | - | - | - | T | C | A | G | A | A | C | A | G | T | C | G | A | C | T | - | - | - | - | A | T | A | T | T | A | A | T |
| PC-3p-54311-8 | T | C | A | A | A | G | T | C | G | A | A | A | T | A | T | C | G | G | A | C | A | C | G | A | C | T | - | - | - | - | - | - | - | T | C | A | G | A | A | C | A | G | T | C | G | A | C | T | - | - | - | - | A | T | G | T | T | A | A | T |
| PC-3p-54311-32 | T | C | A | A | A | G | T | C | G | A | A | A | T | A | T | C | G | A | A | C | A | C | G | A | C | A | G | - | - | - | - | - | T | T | C | A | G | A | A | C | A | G | T | C | G | A | C | T | - | - | - | - | A | T | G | T | T | A | A | T |
| PC-3p-54311-57 | T | C | A | A | A | G | T | C | A | A | A | A | T | A | T | C | G | G | A | C | A | C | G | A | C | T | - | - | - | - | - | - | - | T | C | A | G | A | A | C | A | G | T | C | G | A | C | T | - | - | - | - | A | A | G | T | T | A | A | T |
| PC-3p-54311-44 | T | C | A | G | A | A | T | C | G | A | A | A | T | A | T | C | G | G | A | C | A | C | G | A | C | T | - | - | - | - | - | - | - | T | C | A | G | A | A | C | A | G | T | C | G | A | C | T | - | - | - | - | A | T | G | T | T | T | A | T |
| PC-3p-54311-26 | G | C | A | A | A | G | T | C | G | A | A | G | T | A | T | C | G | G | A | A | A | C | G | A | C | T | - | - | - | - | - | - | - | T | C | A | G | A | A | C | A | G | T | C | G | A | C | T | - | - | - | - | A | T | G | T | T | A | A | T |
| PC-3p-54311-61 | T | C | A | A | A | G | T | C | A | A | A | A | A | A | T | C | G | T | A | A | A | C | G | A | C | T | - | - | - | - | - | - | - | A | C | A | G | A | A | T | A | G | T | C | G | A | C | T | - | - | - | - | A | T | G | T | T | C | A | T |
| PC-3p-54311-47 | T | C | A | A | A | G | T | C | G | A | A | A | T | A | T | C | G | G | A | C | A | C | G | A | C | T | - | - | - | - | - | - | - | T | C | A | G | A | A | C | A | G | T | C | A | A | C | T | - | - | - | - | A | T | G | T | T | A | A | T |
| PC-3p-54311-63 | T | C | A | A | A | G | T | C | G | A | A | A | T | A | T | C | G | G | A | C | A | C | G | A | C | T | - | - | - | - | - | - | - | T | C | A | G | A | A | C | A | G | T | C | G | A | C | T | - | - | - | - | A | T | G | C | T | A | A | T |
| PC-3p-54311-60 | - | - | A | A | A | G | - | - | - | A | A | A | C | A | T | T | G | G | A | C | A | C | G | A | C | T | - | - | - | - | - | - | - | T | C | A | A | A | A | C | A | G | T | C | G | A | C | T | - | - | - | - | A | T | A | T | T | A | A | T |
| PC-3p-54311-38 | T | C | A | A | A | G | T | C | G | A | A | A | T | A | T | C | A | G | A | A | A | C | G | A | C | T | - | - | - | - | - | - | - | T | C | A | A | A | A | T | A | G | T | C | G | A | C | T | - | - | - | - | A | T | G | T | T | A | A | T |
| PC-3p-54311-17 | T | C | A | A | A | G | T | C | G | A | A | A | T | A | T | C | G | G | A | C | A | C | G | A | C | T | - | - | - | - | - | - | - | T | C | A | G | A | A | C | A | G | T | C | G | A | C | T | - | - | - | - | A | T | G | T | T | A | A | T |
| PC-3p-54311-36 | T | C | A | A | A | G | T | A | G | A | A | A | T | A | T | C | G | G | A | C | A | C | G | A | C | T | - | - | - | - | - | - | - | T | C | A | G | A | A | C | A | G | T | C | G | A | C | T | - | - | - | - | A | T | G | T | T | A | A | T |
| PC-3p-54311-12 | T | C | A | A | A | G | T | C | G | A | A | A | T | A | T | C | G | G | A | C | A | C | A | A | C | T | - | - | - | - | - | - | - | T | C | A | T | A | A | C | A | G | T | C | G | A | C | T | - | - | - | - | A | T | G | T | T | A | A | T |
| PC-3p-54311-3 | T | C | A | A | A | G | T | C | G | A | A | A | T | A | T | C | G | G | A | C | A | C | G | A | C | T | - | - | - | - | - | - | - | T | C | A | G | A | A | C | A | G | T | C | G | A | C | T | G | T | T | G | A | C | G | T | T | G | A | T |
| PC-3p-54311-10 | T | C | A | A | A | G | T | C | G | A | A | A | T | A | T | C | G | G | A | C | A | C | - | A | C | T | - | - | - | - | - | - | - | A | C | A | G | A | A | C | A | G | T | C | A | A | C | T | - | - | - | - | A | C | G | T | T | G | A | T |
| PC-3p-54311-22 | T | C | A | T | A | A | T | C | G | A | A | A | T | A | T | T | G | G | A | C | A | C | G | A | C | T | - | - | - | - | - | - | - | T | C | A | G | A | A | C | A | G | T | C | G | A | C | T | - | - | - | - | A | C | G | T | T | G | A | T |
| PC-3p-54311-49 | T | C | A | A | A | G | T | C | G | A | A | A | T | A | T | C | G | G | A | C | A | C | G | A | C | T | - | - | - | - | - | - | - | T | C | A | G | A | A | C | A | G | T | T | G | A | C | T | - | - | - | - | A | C | G | T | T | G | A | T |
| PC-3p-54311-1 | T | C | A | A | A | G | T | C | G | A | A | A | T | A | T | C | G | G | A | C | A | C | G | A | C | T | - | - | - | - | - | - | - | T | C | A | G | A | A | C | A | G | T | C | G | A | C | T | - | - | - | - | A | T | G | T | T | G | A | T |
| PC-3p-54311-55 | T | C | A | A | A | G | T | C | G | A | A | A | T | A | T | C | G | G | A | C | A | C | G | A | C | T | - | - | - | - | - | - | - | A | C | A | G | A | A | C | A | T | T | C | G | A | C | T | - | - | - | - | A | C | G | T | T | G | A | T |
| PC-3p-54311-2 | T | C | A | A | A | G | T | C | G | A | A | A | T | A | T | C | G | G | A | C | A | C | G | A | C | T | - | - | - | - | - | - | - | T | C | A | G | A | A | C | A | G | T | C | G | A | C | T | - | - | - | - | A | T | G | T | T | G | A | T |
| PC-3p-54311-9 | T | C | A | A | A | G | T | T | G | A | A | A | T | A | T | C | G | G | A | C | A | C | A | A | C | T | - | - | - | - | - | - | - | T | C | A | G | A | A | C | A | G | T | C | G | A | C | T | - | - | - | - | A | C | A | T | T | G | A | T |
| PC-3p-54311-20 | T | C | A | A | A | G | T | C | G | A | A | A | T | A | T | C | G | G | A | C | A | C | G | A | C | T | - | - | - | - | - | - | - | T | C | A | G | A | A | C | A | G | T | C | G | A | C | T | - | - | - | - | A | C | G | T | T | G | A | T |
| PC-3p-54311-18 | T | C | A | A | A | A | T | C | G | A | A | A | T | A | T | C | G | G | A | C | A | C | G | A | C | T | - | - | - | - | - | - | - | T | C | A | G | A | A | C | A | G | T | C | G | A | C | T | - | - | - | - | A | C | G | T | T | G | A | T |
| PC-3p-54311-37 | T | C | A | A | A | G | T | T | G | A | A | A | T | A | T | C | G | G | A | C | A | C | G | A | C | C | - | - | - | - | - | - | - | T | C | A | G | A | A | C | A | G | T | C | G | A | C | T | - | - | - | - | A | C | G | T | T | G | A | T |
| PC-3p-54311-45 | T | C | A | A | A | G | T | C | G | A | A | A | T | A | T | C | G | G | A | C | A | C | G | A | C | T | - | - | - | - | - | - | - | T | C | A | G | A | A | C | A | G | T | C | G | A | C | T | - | - | - | - | A | C | G | T | T | G | A | T |
| PC-3p-54311-35 | T | T | T | T | C | G | T | C | G | A | A | A | T | A | T | C | G | G | A | C | A | C | G | A | C | T | - | - | - | - | - | - | - | T | C | A | G | A | A | C | A | G | T | C | G | A | C | T | - | - | - | - | A | C | G | T | T | G | A | T |
| PC-3p-54311-41 | T | C | A | A | A | G | T | C | G | A | A | A | T | A | T | C | G | G | A | C | A | C | G | A | C | T | - | - | - | - | - | - | - | T | C | A | G | A | A | C | A | G | T | C | G | A | C | T | - | - | - | - | A | C | G | T | T | G | A | T |
| PC-3p-54311-40 | T | C | A | A | A | G | A | C | G | A | A | A | T | A | T | C | G | G | A | C | A | C | G | A | C | T | - | - | - | - | - | - | - | T | C | A | G | A | A | C | G | G | T | C | G | A | C | T | - | - | - | - | A | C | G | T | T | G | A | T |
| PC-3p-54311-4 | T | C | A | A | A | G | T | C | G | A | A | A | T | A | T | C | G | G | A | G | A | C | G | A | C | T | - | - | - | - | - | - | - | T | T | A | G | A | A | C | A | G | T | C | G | A | C | T | - | - | - | - | A | C | G | T | T | G | A | T |
| PC-3p-54311-42 | T | C | A | A | A | G | C | C | G | A | A | A | T | A | T | C | G | G | A | T | A | C | G | A | C | T | - | - | - | - | - | - | - | T | C | A | G | A | A | C | A | G | T | C | G | A | C | T | - | - | - | - | A | C | G | T | T | G | A | T |
| PC-3p-54311-16 | T | C | A | A | A | G | T | T | G | A | G | A | T | A | T | C | G | G | A | C | A | C | G | A | C | T | - | - | - | - | - | - | - | T | C | A | G | A | A | C | A | G | T | C | G | A | C | T | - | - | - | - | A | C | G | T | T | A | A | T |
| PC-3p-54311-27 | T | C | A | A | A | G | T | C | G | A | A | A | T | A | T | C | G | G | A | C | A | C | G | A | C | T | - | - | - | - | - | - | - | T | C | A | G | A | A | C | A | G | T | C | G | A | C | T | - | - | - | - | A | C | G | T | T | G | A | T |
| PC-3p-54311-15 | T | C | A | A | A | G | T | C | G | A | A | A | T | A | T | C | G | G | A | C | A | C | G | A | C | T | - | - | - | - | - | - | - | T | C | A | G | A | A | C | A | G | T | C | G | A | C | T | - | - | - | - | A | C | G | T | T | G | A | T |
| PC-3p-54311-14 | T | C | A | A | A | G | T | C | G | A | A | A | T | A | T | C | G | G | A | C | A | C | G | A | C | T | - | - | - | - | - | - | - | T | C | A | G | A | A | C | A | G | T | C | G | A | T | T | - | - | - | - | A | C | G | T | T | G | A | T |
| PC-3p-54311-21 | T | C | A | A | A | G | T | C | G | A | A | A | T | A | T | C | G | G | A | C | A | C | G | A | C | T | - | - | - | - | - | - | - | T | C | A | G | A | A | C | A | G | T | C | G | A | C | T | - | - | - | - | A | C | G | T | T | G | A | T |
| PC-3p-54311-24 | T | C | A | A | A | G | T | C | G | A | A | A | T | A | T | C | G | G | A | C | A | C | G | A | C | T | - | - | - | - | - | - | - | T | C | A | G | A | A | C | A | G | T | C | G | A | C | T | - | - | - | - | A | C | G | T | T | G | A | T |
| PC-3p-54311-65 | T | G | A | A | A | G | T | C | G | A | A | A | T | A | T | C | G | G | A | C | A | C | G | A | C | T | - | - | - | - | - | - | - | T | C | A | G | A | A | C | A | G | T | C | G | A | C | T | - | - | - | - | A | C | G | T | T | G | A | T |
| PC-3p-54311-28 | T | C | A | A | A | G | T | T | G | A | A | A | T | A | T | C | G | G | A | C | A | C | G | A | C | T | - | - | - | - | - | - | - | T | C | A | G | A | A | C | A | G | T | C | G | A | C | T | - | - | - | - | A | C | G | T | T | G | A | T |
| PC-3p-54311-50 | T | C | A | A | A | G | T | C | G | A | A | A | T | A | T | C | G | G | A | C | A | C | G | A | C | T | - | - | - | - | - | - | - | T | C | A | A | A | A | C | A | G | T | C | G | A | C | T | - | - | - | - | A | C | G | T | T | G | A | T |
| PC-3p-54311-30 | T | C | A | A | A | G | T | C | G | A | A | A | T | A | T | C | G | G | A | C | A | C | G | A | C | T | - | - | - | - | - | - | - | T | C | A | G | A | A | C | A | G | T | C | G | A | C | T | - | - | - | - | A | C | G | T | T | G | A | T |
| PC-3p-54311-11 | T | A | A | A | A | G | T | C | G | A | A | A | T | A | T | T | G | G | A | C | A | A | G | A | C | T | - | - | - | - | - | - | - | T | C | A | G | A | A | C | A | G | T | C | G | A | C | T | - | - | - | - | A | C | G | T | T | A | A | T |
| PC-3p-54311-19 | - | A | A | A | A | G | T | C | G | A | A | A | T | A | T | C | G | G | A | C | A | C | G | A | C | T | - | - | - | - | - | - | - | T | C | A | G | A | A | C | A | G | T | C | G | A | C | T | - | - | - | - | A | C | G | T | T | G | A | T |
| PC-3p-54311-23 | A | A | A | A | A | T | G | A | C | G | T | T | A | A | A | A | G | T | A | G | T | C | G | A | C | T | - | - | - | - | - | - | - | T | C | A | G | A | A | C | A | G | T | C | G | A | C | T | - | - | - | - | A | C | G | T | T | G | A | T |
| PC-3p-54311-29 | A | T | T | T | C | C | G | A | T | A | T | A | T | A | T | C | G | G | A | C | A | C | G | A | C | T | - | - | - | - | - | - | - | T | C | A | G | A | A | C | A | G | T | C | G | A | C | T | - | - | - | - | A | C | G | T | T | G | A | T |
| PC-3p-54311-31 | A | A | A | T | T | T | G | G | T | G | T | C | T | A | T | C | - | - | - | C | C | C | G | A | C | T | - | - | - | - | - | - | - | T | C | G | A | A | A | C | A | G | T | C | G | A | C | T | - | - | - | - | A | C | G | T | T | G | A | T |
| PC-3p-54311-6 | T | A | G | A | G | A | T | T | G | A | C | G | G | T | A | A | A | C | G | C | A | C | T | C | A | T | - | - | - | - | - | - | - | T | T | C | G | A | C | T | A | G | T | C | A | A | A | G | - | - | - | - | T | C | G | A | A | A | T | A |
| PC-3p-54311-64 | A | A | A | A | A | G | A | A | G | G | C | T | T | T | T | A | A | C | A | A | A | C | T | T | A | T | T | T | C | G | A | C | T | T | C | A | A | A | A | C | G | G | T | T | G | A | C | T | - | - | - | - | T | A | G | T | A | A | A | T |

**┤Complementary region ends**

| PC-3p-54311-5 | G | T | - | - | - | - | - | - | - | T | C | G | A | C | T | G | - | - | - | - | T | G | T | - | - | A | C | C | G | G | T | C | G | C | C | A | - | - | - | - | A | A | T | T | C | A | T | T | - | - | - | - | - | - | - | - | - | - | - | - |
| --- | --- | --- | --- | --- | --- | --- | --- | --- | --- | --- | --- | --- | --- | --- | --- | --- | --- | --- | --- | --- | --- | --- | --- | --- | --- | --- | --- | --- | --- | --- | --- | --- | --- | --- | --- | --- | --- | --- | --- | --- | --- | --- | --- | --- | --- | --- | --- | --- | --- | --- | --- | --- | --- | --- | --- | --- | --- | --- | --- | --- |
| PC-3p-54311-46 | G | T | - | - | - | - | - | - | - | T | C | G | A | C | T | G | - | - | - | - | T | G | T | - | - | A | C | C | A | A | T | C | G | C | C | A | - | - | - | - | A | A | T | T | C | A | T | T | - | - | - | - | - | - | - | - | - | - | - | - |
| PC-3p-54311-7 | G | T | - | - | - | - | - | - | - | T | C | G | G | C | T | G | - | - | - | - | T | A | T | - | - | A | C | C | G | G | T | C | G | C | C | A | - | - | - | - | A | A | T | T | C | A | T | T | - | - | - | - | - | - | - | - | - | - | - | - |
| PC-3p-54311-39 | G | T | - | - | - | - | - | - | - | T | C | G | A | C | T | G | - | - | - | - | T | G | T | G | T | A | C | C | G | G | T | C | G | C | C | A | - | - | - | - | A | A | T | T | C | A | T | T | - | - | - | - | - | - | - | - | - | - | - | - |
| PC-3p-54311-66 | G | T | - | - | - | - | - | - | - | T | C | G | A | C | T | G | - | - | - | - | T | G | T | - | - | A | C | C | G | G | T | C | G | C | C | A | - | - | - | - | A | A | T | T | C | A | T | T | - | - | - | - | - | - | - | - | - | - | - | - |
| PC-3p-54311-51 | A | T | - | - | - | - | - | - | - | T | C | G | A | C | T | G | - | - | - | - | T | G | T | - | - | A | C | C | G | G | T | C | G | C | C | A | - | - | - | - | A | A | T | T | C | A | T | T | - | - | - | - | - | - | - | - | - | - | - | - |
| PC-3p-54311-59 | G | T | - | - | - | - | - | - | - | T | T | G | A | C | T | G | - | - | - | - | T | G | T | - | - | A | C | C | G | G | T | C | G | C | T | A | - | - | - | - | A | A | T | T | C | A | T | T | - | - | - | - | - | - | - | - | - | - | - | - |
| PC-3p-54311-43 | G | T | - | - | - | - | - | - | - | T | C | G | A | C | T | G | - | - | - | - | T | G | T | - | - | A | C | C | G | G | T | T | G | C | C | A | - | - | - | - | A | A | T | T | C | A | T | T | - | - | - | - | - | - | - | - | - | - | - | - |
| PC-3p-54311-48 | G | T | - | - | - | - | - | - | - | C | C | G | A | C | T | G | - | - | - | - | T | G | T | - | - | A | C | C | G | G | T | T | G | C | C | A | - | - | - | - | A | A | T | T | C | A | T | T | - | - | - | - | - | - | - | - | - | - | - | - |
| PC-3p-54311-53 | G | T | - | - | - | - | - | - | - | T | C | G | A | C | C | C | T | - | - | - | T | G | T | - | - | A | C | C | G | G | T | C | G | C | C | A | - | - | - | - | A | A | T | T | C | A | T | T | - | - | - | - | - | - | - | - | - | - | - | - |
| PC-3p-54311-62 | T | T | - | - | - | - | - | - | - | T | C | G | A | C | T | G | - | - | - | - | T | G | T | - | - | A | T | C | G | G | T | C | G | C | C | A | - | - | - | - | A | A | T | T | C | A | T | T | - | - | - | - | - | - | - | - | - | - | - | - |
| PC-3p-54311-58 | G | T | - | - | - | - | - | - | - | T | A | G | A | C | T | G | - | - | - | - | T | G | T | - | - | A | C | C | G | G | T | C | G | C | C | A | - | - | - | - | A | A | T | T | C | A | T | T | - | - | - | - | - | - | - | - | - | - | - | - |
| PC-3p-54311-52 | G | T | - | - | - | - | - | - | - | T | A | G | A | C | A | G | - | - | - | - | T | G | T | - | - | A | C | C | G | G | T | C | G | C | C | A | - | - | - | - | A | A | T | T | C | A | T | T | - | - | - | - | - | - | - | - | - | - | - | - |
| PC-3p-54311-56 | G | T | - | - | - | - | - | - | - | T | C | G | A | C | T | G | - | - | - | - | T | G | T | - | - | A | C | T | G | G | T | C | G | C | C | A | - | - | - | - | A | A | T | T | C | A | T | T | - | - | - | - | - | - | - | - | - | - | - | - |
| PC-3p-54311-13 | G | T | G | T | T | A | A | T | G | T | A | A | A | C | T | G | - | - | - | - | T | G | T | - | - | A | C | T | G | G | T | C | G | C | C | A | - | - | - | - | A | A | T | T | C | A | T | T | - | - | - | - | - | - | - | - | - | - | - | - |
| PC-3p-54311-33 | G | T | - | - | - | - | - | - | - | T | C | G | A | C | T | G | - | - | - | - | T | G | T | - | - | A | C | C | G | G | T | C | G | C | C | A | - | - | - | - | A | A | T | T | C | A | T | T | - | - | - | - | - | - | - | - | - | - | - | - |
| PC-3p-54311-25 | G | T | - | - | - | - | - | - | - | T | C | G | A | C | T | G | - | - | - | - | T | G | T | - | - | A | T | C | G | G | T | C | G | C | C | G | - | - | - | - | A | A | T | T | C | A | T | T | - | - | - | - | - | - | - | - | - | - | - | - |
| PC-3p-54311-34 | T | T | - | - | - | - | - | - | - | T | C | G | A | C | T | G | T | A | T | A | T | A | C | A | - | A | C | G | G | - | T | C | G | G | - | - | - | - | - | - | T | A | T | A | A | A | A | T | - | - | - | - | - | - | - | - | - | - | - | - |
| PC-3p-54311-54 | T | T | - | - | - | - | - | - | - | T | C | G | A | C | C | G | - | - | - | - | T | A | C | A | - | A | T | G | G | - | T | C | G | G | - | - | - | - | - | - | T | A | T | G | A | A | A | T | - | - | - | - | - | - | - | - | - | - | - | - |
| PC-3p-54311-8 | T | T | - | - | - | - | - | - | - | T | C | G | A | C | C | T | - | - | - | - | T | A | C | G | - | A | C | G | A | - | T | C | G | G | - | - | - | - | - | - | T | A | T | G | A | A | A | T | - | - | - | - | - | - | - | - | - | - | - | - |
| PC-3p-54311-32 | T | T | - | - | - | - | - | - | - | T | C | G | A | C | T | G | T | A | T | A | T | A | C | A | - | A | C | G | G | - | T | C | G | G | - | - | - | - | - | - | T | A | T | A | A | A | A | T | - | - | - | - | - | - | - | - | - | - | - | - |
| PC-3p-54311-57 | T | T | - | - | - | - | - | - | - | T | C | G | A | T | C | G | - | - | - | - | T | A | C | A | - | A | C | A | G | - | T | C | G | A | - | - | - | - | - | - | T | A | T | G | A | A | A | T | - | - | - | - | - | - | - | - | - | - | - | - |
| PC-3p-54311-44 | T | T | - | - | - | - | - | - | - | T | C | G | A | T | C | G | - | - | - | - | T | G | C | A | - | A | C | G | G | - | T | C | G | G | - | - | - | - | - | - | T | A | T | G | A | A | A | T | - | - | - | - | - | - | - | - | - | - | - | - |
| PC-3p-54311-26 | T | T | - | - | - | - | - | - | - | T | C | G | A | C | C | G | - | - | - | - | T | G | C | A | - | A | C | G | G | - | T | C | A | G | - | - | - | - | - | - | T | A | T | G | A | A | A | T | - | - | - | - | - | - | - | - | - | - | - | - |
| PC-3p-54311-61 | T | T | - | - | - | - | - | - | - | T | C | A | A | C | C | G | - | - | - | - | T | A | C | A | - | A | C | G | T | T | T | C | G | C | C | C | A | T | A | C | T | A | T | G | A | A | A | T | - | - | - | - | - | - | - | - | - | - | - | - |
| PC-3p-54311-47 | T | T | - | - | - | - | - | - | - | T | C | G | G | - | - | - | - | - | - | - | - | - | - | - | - | - | - | - | - | - | - | - | - | - | - | - | - | - | - | - | T | A | T | G | A | A | A | T | - | - | - | - | - | - | - | - | - | - | - | - |
| PC-3p-54311-63 | T | T | - | - | - | - | - | - | - | T | C | G | A | - | - | - | - | - | - | - | - | - | - | - | - | - | - | - | - | - | - | - | - | - | - | - | - | - | - | - | T | A | T | G | A | A | A | T | - | - | - | - | - | - | - | - | - | - | - | - |
| PC-3p-54311-60 | T | T | - | - | - | - | - | - | - | T | C | G | A | T | C | G | - | - | - | - | T | A | C | A | - | A | C | G | G | T | T | G | G | - | - | - | - | - | - | - | T | A | T | G | A | A | A | T | - | - | - | - | - | - | - | - | - | - | - | - |
| PC-3p-54311-38 | T | T | - | - | - | - | - | - | - | T | C | G | A | G | - | - | - | - | - | - | - | - | - | - | - | - | - | - | - | - | T | G | A | - | - | - | - | - | - | - | T | A | T | G | A | A | A | T | - | - | - | - | - | - | - | - | - | - | - | - |
| PC-3p-54311-17 | T | T | - | - | - | - | - | - | - | T | C | G | A | C | C | G | - | - | - | - | T | A | C | A | - | A | C | A | T | - | T | C | G | A | - | - | - | - | - | - | T | A | T | G | A | A | A | T | - | - | - | - | - | - | - | - | - | - | - | - |
| PC-3p-54311-36 | T | T | - | - | - | - | - | - | - | T | C | G | A | C | C | G | - | - | - | - | T | A | C | A | - | A | C | A | G | - | T | C | G | A | - | - | - | - | - | - | T | A | T | G | A | A | A | T | - | - | - | - | - | - | - | - | - | - | - | - |
| PC-3p-54311-12 | T | T | - | - | - | - | - | - | - | T | C | G | A | C | C | G | - | - | - | - | T | A | C | A | - | A | C | G | G | - | T | C | G | G | - | - | - | - | - | - | T | A | T | G | A | T | A | T | - | - | - | - | - | - | - | - | - | - | - | - |
| PC-3p-54311-3 | A | T | - | - | - | - | - | - | - | T | C | G | A | C | T | G | - | - | - | - | T | G | T | - | - | A | C | C | G | G | T | C | G | C | A | - | - | - | C | A | A | A | T | T | A | A | T | T | - | - | - | - | - | - | - | - | - | - | - | - |
| PC-3p-54311-10 | G | T | - | - | - | - | - | - | - | T | C | G | A | C | T | G | - | - | - | - | T | G | T | - | - | A | C | C | G | G | T | C | G | C | - | - | - | - | C | A | A | A | T | T | C | A | T | T | - | - | - | - | - | - | - | - | - | - | - | - |
| PC-3p-54311-22 | G | T | - | - | - | - | - | - | - | T | C | G | A | C | T | G | - | - | - | - | A | G | T | - | - | A | C | C | G | G | T | C | G | C | - | - | - | - | C | A | A | A | T | T | C | A | T | T | - | - | - | - | - | - | - | - | - | - | - | - |
| PC-3p-54311-49 | G | T | - | - | - | - | - | - | - | T | C | G | A | C | T | G | - | - | - | - | T | G | T | - | - | A | C | A | G | G | T | C | G | C | - | - | - | - | C | A | A | A | C | T | C | A | T | T | - | - | - | - | - | - | - | - | - | - | - | - |
| PC-3p-54311-1 | G | T | - | - | - | - | - | - | - | T | C | G | A | C | T | G | - | - | - | - | T | G | T | - | - | A | C | C | G | G | T | C | G | C | - | - | - | - | C | A | A | A | T | T | C | A | T | T | - | - | - | - | - | - | - | - | - | - | - | - |
| PC-3p-54311-55 | G | T | - | - | - | - | - | - | - | T | C | G | A | C | T | G | - | - | - | - | T | G | T | - | - | A | C | T | G | G | T | C | G | C | - | - | - | - | C | A | A | A | T | T | C | A | T | T | - | - | - | - | - | - | - | - | - | - | - | - |
| PC-3p-54311-2 | G | T | - | - | - | - | - | - | - | T | C | G | A | C | T | G | - | - | - | - | T | G | T | - | - | A | C | C | G | G | T | C | G | C | - | - | - | - | C | A | A | A | T | T | C | A | T | T | - | - | - | - | - | - | - | - | - | - | - | - |
| PC-3p-54311-9 | G | T | - | - | - | - | - | - | - | T | A | G | A | C | T | G | - | - | - | - | T | G | T | - | - | A | C | C | G | G | T | C | G | C | - | - | - | - | C | A | A | A | T | T | C | A | T | T | - | - | - | - | - | - | - | - | - | - | - | - |
| PC-3p-54311-20 | G | T | - | - | - | - | - | - | - | T | T | G | A | T | T | G | - | - | - | - | T | G | T | - | - | A | C | C | G | G | T | C | G | C | - | - | - | - | C | A | A | A | T | T | C | A | T | T | - | - | - | - | - | - | - | - | - | - | - | - |
| PC-3p-54311-18 | G | T | - | - | - | - | - | - | - | T | C | G | A | C | T | G | - | - | - | - | T | G | T | - | - | A | C | C | G | G | T | C | G | C | - | - | - | - | C | G | A | A | T | T | C | A | T | T | - | - | - | - | - | - | - | - | - | - | - | - |
| PC-3p-54311-37 | G | T | - | - | - | - | - | - | - | T | C | G | A | C | T | G | - | - | - | - | T | G | C | - | - | A | C | C | G | G | T | C | G | C | - | - | - | - | C | A | A | A | T | T | C | A | T | T | - | - | - | - | - | - | - | - | - | - | - | - |
| PC-3p-54311-45 | G | T | - | - | - | - | - | - | - | T | C | G | A | C | T | G | - | - | - | - | T | G | T | - | - | A | C | C | G | G | T | C | G | C | - | - | - | - | C | A | A | A | T | T | C | A | T | T | - | - | - | - | - | - | - | - | - | - | - | - |
| PC-3p-54311-35 | T | T | - | - | - | - | - | - | - | T | C | G | A | C | T | G | - | - | - | - | T | G | T | - | - | A | C | C | A | G | T | C | G | C | - | - | - | - | C | A | A | A | T | T | C | A | T | T | - | - | - | - | - | - | - | - | - | - | - | - |
| PC-3p-54311-41 | G | T | - | - | - | - | - | - | - | T | C | G | A | C | T | G | - | - | - | - | T | G | T | - | - | A | C | C | G | G | T | C | G | C | - | - | - | - | C | A | A | A | T | T | C | A | T | T | - | - | - | - | - | - | - | - | - | - | - | - |
| PC-3p-54311-40 | G | T | - | - | - | - | - | - | - | T | C | G | A | C | T | G | - | - | - | - | T | C | T | - | - | A | C | C | G | G | T | C | G | C | - | - | - | - | C | A | A | A | T | T | T | A | T | T | - | - | - | - | - | - | - | - | - | - | - | - |
| PC-3p-54311-4 | G | T | - | - | - | - | - | - | - | T | C | G | A | C | T | G | - | - | - | - | T | G | T | - | - | A | C | C | G | G | T | C | G | C | - | - | - | - | A | A | A | A | T | T | C | A | T | T | - | - | - | - | - | - | - | - | - | - | - | - |
| PC-3p-54311-42 | G | T | - | - | - | - | - | - | - | T | C | G | A | C | T | G | - | - | - | - | T | G | T | - | - | A | C | C | G | G | T | C | G | C | - | - | - | - | C | A | A | A | T | T | T | A | T | T | - | - | - | - | - | - | - | - | - | - | - | - |
| PC-3p-54311-16 | G | T | - | - | - | - | - | - | - | T | C | G | A | C | T | G | - | - | - | - | A | G | T | - | - | A | C | C | G | G | T | C | G | C | - | - | - | - | C | C | A | A | A | T | C | A | T | T | - | - | - | - | - | - | - | - | - | - | - | - |
| PC-3p-54311-27 | G | T | - | - | - | - | - | - | - | T | C | G | A | C | T | G | - | - | - | - | T | G | T | - | - | A | C | C | G | G | T | T | G | C | - | - | - | - | C | A | A | A | T | T | C | A | T | T | - | - | - | - | - | - | - | - | - | - | - | - |
| PC-3p-54311-15 | G | T | - | - | - | - | - | - | - | T | C | G | A | C | T | G | - | - | - | - | T | G | T | - | - | A | C | A | G | G | T | C | G | C | - | - | - | - | C | A | A | A | T | T | C | A | T | T | - | - | - | - | - | - | - | - | - | - | - | - |
| PC-3p-54311-14 | A | T | - | - | - | - | - | - | - | T | C | G | A | C | T | G | - | - | - | - | T | G | T | - | - | A | C | C | G | G | T | C | G | C | - | - | - | - | C | A | A | A | T | T | C | A | T | T | - | - | - | - | - | - | - | - | - | - | - | - |
| PC-3p-54311-21 | G | T | - | - | - | - | - | - | - | T | C | G | A | C | T | G | - | - | - | - | T | G | T | - | - | A | C | T | G | G | T | C | G | C | - | - | - | - | C | A | A | A | T | T | C | A | T | T | - | - | - | - | - | - | - | - | - | - | - | - |
| PC-3p-54311-24 | A | T | - | - | - | - | - | - | - | T | C | G | A | C | T | G | - | - | - | - | T | G | T | - | - | A | C | C | G | G | T | C | G | C | - | - | - | - | C | A | A | A | T | T | C | A | T | T | - | - | - | - | - | - | - | - | - | - | - | - |
| PC-3p-54311-65 | G | T | - | - | - | - | - | - | - | T | C | G | A | C | T | G | - | - | - | - | T | G | T | - | - | A | C | C | G | G | T | C | A | C | - | - | - | - | C | A | A | A | T | T | C | A | T | T | - | - | - | - | - | - | - | - | - | - | - | - |
| PC-3p-54311-28 | G | T | - | - | - | - | - | - | - | T | C | G | A | C | T | G | - | - | - | - | T | G | T | - | - | A | C | C | G | G | T | C | G | C | - | - | - | - | C | A | A | G | T | T | C | A | T | T | - | - | - | - | - | - | - | - | - | - | - | - |
| PC-3p-54311-50 | G | T | - | - | - | - | - | - | - | T | C | G | A | C | T | G | - | - | - | - | T | G | T | - | - | A | C | C | G | G | T | C | G | C | - | - | - | - | C | A | A | A | T | T | C | A | T | T | - | - | - | - | - | - | - | - | - | - | - | - |
| PC-3p-54311-30 | G | T | - | - | - | - | - | - | - | T | C | G | A | C | T | G | - | - | - | - | A | G | T | - | - | A | C | C | G | G | T | C | G | C | - | - | - | - | C | A | A | A | T | T | C | A | T | T | T | C | A | T | A | A | T | A | T | G | A | A |
| PC-3p-54311-11 | A | T | - | - | - | - | - | - | - | T | C | G | A | C | T | G | - | - | - | - | T | G | T | - | - | A | C | C | T | G | T | C | G | C | T | - | - | - | - | A | A | A | T | T | T | A | T | A | - | - | - | - | - | - | - | - | - | - | - | - |
| PC-3p-54311-19 | A | T | - | - | - | - | - | - | - | T | C | G | A | C | T | G | - | - | - | - | T | G | T | - | - | A | C | C | G | G | T | C | G | C | C | A | - | - | - | - | A | A | T | T | C | A | T | T | - | - | - | - | - | - | - | - | - | - | - | - |
| PC-3p-54311-23 | G | T | - | - | - | - | - | - | - | T | T | G | A | C | T | G | - | - | - | - | T | G | T | - | - | A | C | C | G | G | T | C | G | C | C | A | - | - | - | - | A | A | T | T | C | A | T | T | - | - | - | - | - | - | - | - | - | - | - | - |
| PC-3p-54311-29 | G | T | - | - | - | - | - | - | - | T | C | G | A | C | T | G | - | - | - | - | T | G | T | - | - | A | C | C | G | G | T | C | G | C | C | A | - | - | - | - | A | A | T | T | C | A | T | T | - | - | - | - | - | - | - | - | - | - | - | - |
| PC-3p-54311-31 | G | T | - | - | - | - | - | - | - | T | C | G | A | C | T | G | - | - | - | - | A | G | T | - | - | A | C | C | G | G | T | C | G | C | C | A | - | - | - | - | A | A | T | T | T | A | T | T | - | - | - | - | - | - | - | - | - | - | - | - |
| PC-3p-54311-6 | T | - | - | - | - | - | - | - | - | C | C | G | A | C | T | G | - | - | - | - | T | G | - | - | - | A | T | C | G | G | T | C | G | C | C | A | - | - | - | - | A | A | T | T | C | A | T | T | - | - | - | - | - | - | - | - | - | - | - | - |
| PC-3p-54311-64 | G | T | - | - | - | - | - | - | - | T | C | G | A | C | C | A | - | - | - | - | - | - | - | - | - | - | - | - | - | - | - | - | - | - | - | - | - | - | - | - | A | A | T | T | C | A | T | T | - | - | - | - | - | - | - | - | - | - | - | - |
|  |  |  |  |  |  |  |  |  |  |  |  |  |  |  |  |  |  |  |  |  |  |  |  |  |  |  |  |  |  |  |  |  |  |  |  |  |  |  |  |  |  |  |  |  |  |  |  |  |  |  |  |  |  |  |  |  |  |  |  |  |
|  |  |  |  |  |  |  |  |  |  |  |  |  |  |  |  |  |  |  |  |  |  |  |  |  |  |  |  |  |  |  |  |  |  |  |  |  |  |  |  |  |  |  |  |  |  |  |  |  |  |  |  |  |  |  |  |  |  |  |  |  |
|  |  |  |  |  |  |  |  |  |  |  |  |  |  |  |  |  |  |  |  |  |  |  |  |  |  |  |  |  |  |  |  |  |  |  |  |  |  |  |  |  |  |  |  |  |  |  |  |  |  |  |  |  |  |  |  |  |  |  |  |  |
|  |  |  |  |  |  |  |  |  |  |  |  |  |  |  |  |  |  |  |  |  |  |  |  |  |  |  |  |  |  |  |  |  |  |  |  |  |  |  |  |  |  |  |  |  |  |  |  |  |  |  |  |  |  |  |  |  |  |  |  |  |
|  |  |  |  |  |  |  |  |  |  |  |  |  |  |  |  |  |  |  |  |  |  |  |  |  |  |  |  |  |  |  |  |  |  |  |  |  |  |  |  |  |  |  |  |  |  |  |  |  |  |  |  |  |  |  |  |  |  |  |  |  |
|  |  |  |  |  |  |  |  |  |  |  |  |  |  |  |  |  |  |  |  |  |  |  |  |  |  |  |  |  |  |  |  |  |  |  |  |  |  |  |  |  |  |  |  |  |  |  |  |  |  |  |  |  |  |  |  |  |  |  |  |  |
|  |  |  |  |  |  |  |  |  |  |  |  |  |  |  |  |  |  |  |  |  |  |  |  |  |  |  |  |  |  |  |  |  |  |  |  |  |  |  |  |  |  |  |  |  |  |  |  |  |  |  |  |  |  |  |  |  |  |  |  |  |
|  |  |  |  |  |  |  |  |  |  |  |  |  |  |  |  |  |  |  |  |  |  |  |  |  |  |  |  |  |  |  |  |  |  |  |  |  |  |  |  |  |  |  |  |  |  |  |  |  |  |  |  |  |  |  |  |  |  |  |  |  |
|  |  |  |  |  |  |  |  |  |  |  |  |  |  |  |  |  |  |  |  |  |  |  |  |  |  |  |  |  |  |  |  |  |  |  |  |  |  |  |  |  |  |  |  |  |  |  |  |  |  |  |  |  |  |  |  |  |  |  |  |  |
|  |  |  |  |  |  |  |  |  |  |  |  |  |  |  |  |  |  |  |  |  |  |  |  |  |  |  |  |  |  |  |  |  |  |  |  |  |  |  |  |  |  |  |  |  |  |  |  |  |  |  |  |  |  |  |  |  |  |  |  |  |
|  |  |  |  |  |  |  |  |  |  |  |  |  |  |  |  |  |  |  |  |  |  |  |  |  |  |  |  |  |  |  |  |  |  |  |  |  |  |  |  |  |  |  |  |  |  |  |  |  |  |  |  |  |  |  |  |  |  |  |  |  |
|  |  |  |  |  |  |  |  |  |  |  |  |  |  |  |  |  |  |  |  |  |  |  |  |  |  |  |  |  |  |  |  |  |  |  |  |  |  |  |  |  |  |  |  |  |  |  |  |  |  |  |  |  |  |  |  |  |  |  |  |  |
|  |  |  |  |  |  |  |  |  |  |  |  |  |  |  |  |  |  |  |  |  |  |  |  |  |  |  |  |  |  |  |  |  |  |  |  |  |  |  |  |  |  |  |  |  |  |  |  |  |  |  |  |  |  |  |  |  |  |  |  |  |
|  |  |  |  |  |  |  |  |  |  |  |  |  |  |  |  |  |  |  |  |  |  |  |  |  |  |  |  |  |  |  |  |  |  |  |  |  |  |  |  |  |  |  |  |  |  |  |  |  |  |  |  |  |  |  |  |  |  |  |  |  |
|  |  |  |  |  |  |  |  |  |  |  |  |  |  |  |  |  |  |  |  |  |  |  |  |  |  |  |  |  |  |  |  |  |  |  |  |  |  |  |  |  |  |  |  |  |  |  |  |  |  |  |  |  |  |  |  |  |  |  |  |  |
|  |  |  |  |  |  |  |  |  |  |  |  |  |  |  |  |  |  |  |  |  |  |  |  |  |  |  |  |  |  |  |  |  |  |  |  |  |  |  |  |  |  |  |  |  |  |  |  |  |  |  |  |  |  |  |  |  |  |  |  |  |
|  |  |  |  |  |  |  |  |  |  |  |  |  |  |  |  |  |  |  |  |  |  |  |  |  |  |  |  |  |  |  |  |  |  |  |  |  |  |  |  |  |  |  |  |  |  |  |  |  |  |  |  |  |  |  |  |  |  |  |  |  |
|  |  |  |  |  |  |  |  |  |  |  |  |  |  |  |  |  |  |  |  |  |  |  |  |  |  |  |  |  |  |  |  |  |  |  |  |  |  |  |  |  |  |  |  |  |  |  |  |  |  |  |  |  |  |  |  |  |  |  |  |  |
|  |  |  |  |  |  |  |  |  |  |  |  |  |  |  |  |  |  |  |  |  |  |  |  |  |  |  |  |  |  |  |  |  |  |  |  |  |  |  |  |  |  |  |  |  |  |  |  |  |  |  |  |  |  |  |  |  |  |  |  |  |
|  |  |  |  |  |  |  |  |  |  |  |  |  |  |  |  |  |  |  |  |  |  |  |  |  |  |  |  |  |  |  |  |  |  |  |  |  |  |  |  |  |  |  |  |  |  |  |  |  |  |  |  |  |  |  |  |  |  |  |  |  |
|  |  |  |  |  |  |  |  |  |  |  |  |  |  |  |  |  |  |  |  |  |  |  |  |  |  |  |  |  |  |  |  |  |  |  |  |  |  |  |  |  |  |  |  |  |  |  |  |  |  |  |  |  |  |  |  |  |  |  |  |  |
|  |  |  |  |  |  |  |  |  |  |  |  |  |  |  |  |  |  |  |  |  |  |  |  |  |  |  |  |  |  |  |  |  |  |  |  |  |  |  |  |  |  |  |  |  |  |  |  |  |  |  |  |  |  |  |  |  |  |  |  |  |
|  |  |  |  |  |  |  |  |  |  |  |  |  |  |  |  |  |  |  |  |  |  |  |  |  |  |  |  |  |  |  |  |  |  |  |  |  |  |  |  |  |  |  |  |  |  |  |  |  |  |  |  |  |  |  |  |  |  |  |  |  |
|  |  |  |  |  |  |  |  |  |  |  |  |  |  |  |  |  |  |  |  |  |  |  |  |  |  |  |  |  |  |  |  |  |  |  |  |  |  |  |  |  |  |  |  |  |  |  |  |  |  |  |  |  |  |  |  |  |  |  |  |  |
|  |  |  |  |  |  |  |  |  |  |  |  |  |  |  |  |  |  |  |  |  |  |  |  |  |  |  |  |  |  |  |  |  |  |  |  |  |  |  |  |  |  |  |  |  |  |  |  |  |  |  |  |  |  |  |  |  |  |  |  |  |
|  |  |  |  |  |  |  |  |  |  |  |  |  |  |  |  |  |  |  |  |  |  |  |  |  |  |  |  |  |  |  |  |  |  |  |  |  |  |  |  |  |  |  |  |  |  |  |  |  |  |  |  |  |  |  |  |  |  |  |  |  |
|  |  |  |  |  |  |  |  |  |  |  |  |  |  |  |  |  |  |  |  |  |  |  |  |  |  |  |  |  |  |  |  |  |  |  |  |  |  |  |  |  |  |  |  |  |  |  |  |  |  |  |  |  |  |  |  |  |  |  |  |  |
|  |  |  |  |  |  |  |  |  |  |  |  |  |  |  |  |  |  |  |  |  |  |  |  |  |  |  |  |  |  |  |  |  |  |  |  |  |  |  |  |  |  |  |  |  |  |  |  |  |  |  |  |  |  |  |  |  |  |  |  |  |
|  |  |  |  |  |  |  |  |  |  |  |  |  |  |  |  |  |  |  |  |  |  |  |  |  |  |  |  |  |  |  |  |  |  |  |  |  |  |  |  |  |  |  |  |  |  |  |  |  |  |  |  |  |  |  |  |  |  |  |  |  |
|  |  |  |  |  |  |  |  |  |  |  |  |  |  |  |  |  |  |  |  |  |  |  |  |  |  |  |  |  |  |  |  |  |  |  |  |  |  |  |  |  |  |  |  |  |  |  |  |  |  |  |  |  |  |  |  |  |  |  |  |  |
|  |  |  |  |  |  |  |  |  |  |  |  |  |  |  |  |  |  |  |  |  |  |  |  |  |  |  |  |  |  |  |  |  |  |  |  |  |  |  |  |  |  |  |  |  |  |  |  |  |  |  |  |  |  |  |  |  |  |  |  |  |
|  |  |  |  |  |  |  |  |  |  |  |  |  |  |  |  |  |  |  |  |  |  |  |  |  |  |  |  |  |  |  |  |  |  |  |  |  |  |  |  |  |  |  |  |  |  |  |  |  |  |  |  |  |  |  |  |  |  |  |  |  |
|  |  |  |  |  |  |  |  |  |  |  |  |  |  |  |  |  |  |  |  |  |  |  |  |  |  |  |  |  |  |  |  |  |  |  |  |  |  |  |  |  |  |  |  |  |  |  |  |  |  |  |  |  |  |  |  |  |  |  |  |  |
|  |  |  |  |  |  |  |  |  |  |  |  |  |  |  |  |  |  |  |  |  |  |  |  |  |  |  |  |  |  |  |  |  |  |  |  |  |  |  |  |  |  |  |  |  |  |  |  |  |  |  |  |  |  |  |  |  |  |  |  |  |
|  |  |  |  |  |  |  |  |  |  |  |  |  |  |  |  |  |  |  |  |  |  |  |  |  |  |  |  |  |  |  |  |  |  |  |  |  |  |  |  |  |  |  |  |  |  |  |  |  |  |  |  |  |  |  |  |  |  |  |  |  |
|  |  |  |  |  |  |  |  |  |  |  |  |  |  |  |  |  |  |  |  |  |  |  |  |  |  |  |  |  |  |  |  |  |  |  |  |  |  |  |  |  |  |  |  |  |  |  |  |  |  |  |  |  |  |  |  |  |  |  |  |  |
|  |  |  |  |  |  |  |  |  |  |  |  |  |  |  |  |  |  |  |  |  |  |  |  |  |  |  |  |  |  |  |  |  |  |  |  |  |  |  |  |  |  |  |  |  |  |  |  |  |  |  |  |  |  |  |  |  |  |  |  |  |
|  |  |  |  |  |  |  |  |  |  |  |  |  |  |  |  |  |  |  |  |  |  |  |  |  |  |  |  |  |  |  |  |  |  |  |  |  |  |  |  |  |  |  |  |  |  |  |  |  |  |  |  |  |  |  |  |  |  |  |  |  |
|  |  |  |  |  |  |  |  |  |  |  |  |  |  |  |  |  |  |  |  |  |  |  |  |  |  |  |  |  |  |  |  |  |  |  |  |  |  |  |  |  |  |  |  |  |  |  |  |  |  |  |  |  |  |  |  |  |  |  |  |  |
|  |  |  |  |  |  |  |  |  |  |  |  |  |  |  |  |  |  |  |  |  |  |  |  |  |  |  |  |  |  |  |  |  |  |  |  |  |  |  |  |  |  |  |  |  |  |  |  |  |  |  |  |  |  |  |  |  |  |  |  |  |
|  |  |  |  |  |  |  |  |  |  |  |  |  |  |  |  |  |  |  |  |  |  |  |  |  |  |  |  |  |  |  |  |  |  |  |  |  |  |  |  |  |  |  |  |  |  |  |  |  |  |  |  |  |  |  |  |  |  |  |  |  |
|  |  |  |  |  |  |  |  |  |  |  |  |  |  |  |  |  |  |  |  |  |  |  |  |  |  |  |  |  |  |  |  |  |  |  |  |  |  |  |  |  |  |  |  |  |  |  |  |  |  |  |  |  |  |  |  |  |  |  |  |  |
|  |  |  |  |  |  |  |  |  |  |  |  |  |  |  |  |  |  |  |  |  |  |  |  |  |  |  |  |  |  |  |  |  |  |  |  |  |  |  |  |  |  |  |  |  |  |  |  |  |  |  |  |  |  |  |  |  |  |  |  |  |
|  |  |  |  |  |  |  |  |  |  |  |  |  |  |  |  |  |  |  |  |  |  |  |  |  |  |  |  |  |  |  |  |  |  |  |  |  |  |  |  |  |  |  |  |  |  |  |  |  |  |  |  |  |  |  |  |  |  |  |  |  |
|  |  |  |  |  |  |  |  |  |  |  |  |  |  |  |  |  |  |  |  |  |  |  |  |  |  |  |  |  |  |  |  |  |  |  |  |  |  |  |  |  |  |  |  |  |  |  |  |  |  |  |  |  |  |  |  |  |  |  |  |  |
|  |  |  |  |  |  |  |  |  |  |  |  |  |  |  |  |  |  |  |  |  |  |  |  |  |  |  |  |  |  |  |  |  |  |  |  |  |  |  |  |  |  |  |  |  |  |  |  |  |  |  |  |  |  |  |  |  |  |  |  |  |
|  |  |  |  |  |  |  |  |  |  |  |  |  |  |  |  |  |  |  |  |  |  |  |  |  |  |  |  |  |  |  |  |  |  |  |  |  |  |  |  |  |  |  |  |  |  |  |  |  |  |  |  |  |  |  |  |  |  |  |  |  |
|  |  |  |  |  |  |  |  |  |  |  |  |  |  |  |  |  |  |  |  |  |  |  |  |  |  |  |  |  |  |  |  |  |  |  |  |  |  |  |  |  |  |  |  |  |  |  |  |  |  |  |  |  |  |  |  |  |  |  |  |  |
|  |  |  |  |  |  |  |  |  |  |  |  |  |  |  |  |  |  |  |  |  |  |  |  |  |  |  |  |  |  |  |  |  |  |  |  |  |  |  |  |  |  |  |  |  |  |  |  |  |  |  |  |  |  |  |  |  |  |  |  |  |
|  |  |  |  |  |  |  |  |  |  |  |  |  |  |  |  |  |  |  |  |  |  |  |  |  |  |  |  |  |  |  |  |  |  |  |  |  |  |  |  |  |  |  |  |  |  |  |  |  |  |  |  |  |  |  |  |  |  |  |  |  |
|  |  |  |  |  |  |  |  |  |  |  |  |  |  |  |  |  |  |  |  |  |  |  |  |  |  |  |  |  |  |  |  |  |  |  |  |  |  |  |  |  |  |  |  |  |  |  |  |  |  |  |  |  |  |  |  |  |  |  |  |  |
|  |  |  |  |  |  |  |  |  |  |  |  |  |  |  |  |  |  |  |  |  |  |  |  |  |  |  |  |  |  |  |  |  |  |  |  |  |  |  |  |  |  |  |  |  |  |  |  |  |  |  |  |  |  |  |  |  |  |  |  |  |
|  |  |  |  |  |  |  |  |  |  |  |  |  |  |  |  |  |  |  |  |  |  |  |  |  |  |  |  |  |  |  |  |  |  |  |  |  |  |  |  |  |  |  |  |  |  |  |  |  |  |  |  |  |  |  |  |  |  |  |  |  |
|  |  |  |  |  |  |  |  |  |  |  |  |  |  |  |  |  |  |  |  |  |  |  |  |  |  |  |  |  |  |  |  |  |  |  |  |  |  |  |  |  |  |  |  |  |  |  |  |  |  |  |  |  |  |  |  |  |  |  |  |  |
|  |  |  |  |  |  |  |  |  |  |  |  |  |  |  |  |  |  |  |  |  |  |  |  |  |  |  |  |  |  |  |  |  |  |  |  |  |  |  |  |  |  |  |  |  |  |  |  |  |  |  |  |  |  |  |  |  |  |  |  |  |
|  |  |  |  |  |  |  |  |  |  |  |  |  |  |  |  |  |  |  |  |  |  |  |  |  |  |  |  |  |  |  |  |  |  |  |  |  |  |  |  |  |  |  |  |  |  |  |  |  |  |  |  |  |  |  |  |  |  |  |  |  |
|  |  |  |  |  |  |  |  |  |  |  |  |  |  |  |  |  |  |  |  |  |  |  |  |  |  |  |  |  |  |  |  |  |  |  |  |  |  |  |  |  |  |  |  |  |  |  |  |  |  |  |  |  |  |  |  |  |  |  |  |  |
|  |  |  |  |  |  |  |  |  |  |  |  |  |  |  |  |  |  |  |  |  |  |  |  |  |  |  |  |  |  |  |  |  |  |  |  |  |  |  |  |  |  |  |  |  |  |  |  |  |  |  |  |  |  |  |  |  |  |  |  |  |
|  |  |  |  |  |  |  |  |  |  |  |  |  |  |  |  |  |  |  |  |  |  |  |  |  |  |  |  |  |  |  |  |  |  |  |  |  |  |  |  |  |  |  |  |  |  |  |  |  |  |  |  |  |  |  |  |  |  |  |  |  |
|  |  |  |  |  |  |  |  |  |  |  |  |  |  |  |  |  |  |  |  |  |  |  |  |  |  |  |  |  |  |  |  |  |  |  |  |  |  |  |  |  |  |  |  |  |  |  |  |  |  |  |  |  |  |  |  |  |  |  |  |  |
|  |  |  |  |  |  |  |  |  |  |  |  |  |  |  |  |  |  |  |  |  |  |  |  |  |  |  |  |  |  |  |  |  |  |  |  |  |  |  |  |  |  |  |  |  |  |  |  |  |  |  |  |  |  |  |  |  |  |  |  |  |
|  |  |  |  |  |  |  |  |  |  |  |  |  |  |  |  |  |  |  |  |  |  |  |  |  |  |  |  |  |  |  |  |  |  |  |  |  |  |  |  |  |  |  |  |  |  |  |  |  |  |  |  |  |  |  |  |  |  |  |  |  |
|  |  |  |  |  |  |  |  |  |  |  |  |  |  |  |  |  |  |  |  |  |  |  |  |  |  |  |  |  |  |  |  |  |  |  |  |  |  |  |  |  |  |  |  |  |  |  |  |  |  |  |  |  |  |  |  |  |  |  |  |  |
|  |  |  |  |  |  |  |  |  |  |  |  |  |  |  |  |  |  |  |  |  |  |  |  |  |  |  |  |  |  |  |  |  |  |  |  |  |  |  |  |  |  |  |  |  |  |  |  |  |  |  |  |  |  |  |  |  |  |  |  |  |
|  |  |  |  |  |  |  |  |  |  |  |  |  |  |  |  |  |  |  |  |  |  |  |  |  |  |  |  |  |  |  |  |  |  |  |  |  |  |  |  |  |  |  |  |  |  |  |  |  |  |  |  |  |  |  |  |  |  |  |  |  |
|  |  |  |  |  |  |  |  |  |  |  |  |  |  |  |  |  |  |  |  |  |  |  |  |  |  |  |  |  |  |  |  |  |  |  |  |  |  |  |  |  |  |  |  |  |  |  |  |  |  |  |  |  |  |  |  |  |  |  |  |  |

**├miRNA starts**

| PC-3p-54311-5 | - | - | - | - | - | - | - | - | C | C | A | T | A | - | - | - | - | - | - | - | T | C | G | - | - | - | - | - | - | - | - | - | - | - | - | - | - | - | - | - | - | - | - | A | A | A | A | T | T | A | A | C | A | T | A | G | T | C | G | A |
| --- | --- | --- | --- | --- | --- | --- | --- | --- | --- | --- | --- | --- | --- | --- | --- | --- | --- | --- | --- | --- | --- | --- | --- | --- | --- | --- | --- | --- | --- | --- | --- | --- | --- | --- | --- | --- | --- | --- | --- | --- | --- | --- | --- | --- | --- | --- | --- | --- | --- | --- | --- | --- | --- | --- | --- | --- | --- | --- | --- | --- |
| PC-3p-54311-46 | - | - | - | - | - | - | - | - | T | C | A | T | A | - | - | - | - | - | - | - | C | C | G | - | - | - | - | - | - | - | - | - | - | - | - | - | - | - | - | - | - | - | - | A | A | A | A | T | T | A | A | C | A | T | A | G | T | T | G | A |
| PC-3p-54311-7 | - | - | - | - | - | - | - | - | T | C | A | T | A | - | - | - | - | - | - | - | C | C | G | A | T | C | G | T | C | G | - | - | - | - | T | A | A | G | G | - | T | C | G | A | A | A | A | T | T | A | A | C | A | T | A | G | T | C | G | A |
| PC-3p-54311-39 | - | - | - | - | - | - | - | - | T | C | A | T | A | - | - | - | - | - | - | - | C | C | A | A | C | G | - | - | - | G | - | - | - | - | T | A | C | G | G | - | T | C | A | A | A | A | A | T | T | A | A | C | A | T | A | G | T | C | G | A |
| PC-3p-54311-66 | - | - | - | - | - | - | - | - | T | C | A | T | A | - | - | - | - | - | - | - | C | C | G | A | C | C | G | T | T | G | - | - | - | - | T | A | C | G | G | - | T | C | G | A | A | A | A | T | T | G | A | C | A | T | A | G | T | C | G | A |
| PC-3p-54311-51 | - | - | - | - | - | - | - | - | T | T | A | T | A | - | - | - | - | - | - | - | T | C | G | A | C | T | G | T | T | G | - | - | - | - | T | A | C | G | G | - | T | C | G | A | A | A | A | T | T | A | A | C | A | T | A | G | T | C | G | C |
| PC-3p-54311-59 | - | - | - | - | - | - | - | - | T | C | A | T | A | - | - | - | - | - | - | - | C | C | A | A | C | C | G | T | T | G | - | - | - | - | T | A | C | G | A | - | T | C | G | A | A | A | A | T | T | A | A | T | A | T | A | G | T | C | G | A |
| PC-3p-54311-43 | - | - | - | - | - | - | - | - | T | C | A | T | A | - | - | - | - | - | - | - | C | C | G | A | C | C | G | T | T | G | - | - | - | - | C | A | C | G | A | - | T | C | G | A | A | A | A | T | A | A | A | C | A | T | A | G | T | C | G | A |
| PC-3p-54311-48 | - | - | - | - | - | - | - | - | T | C | A | T | A | - | - | - | - | - | - | - | C | C | G | A | C | C | G | T | T | G | - | - | - | - | T | A | C | G | G | - | T | T | G | A | A | A | A | T | A | A | A | C | A | T | A | G | T | C | G | A |
| PC-3p-54311-53 | - | - | - | - | - | - | - | - | T | C | A | T | A | - | - | - | - | - | - | - | C | C | G | A | C | C | A | T | T | G | - | - | - | - | T | A | C | G | G | - | T | C | G | A | A | A | A | T | T | A | A | T | A | T | A | G | T | C | G | A |
| PC-3p-54311-62 | - | - | - | - | - | - | - | - | T | C | A | T | A | G | T | A | T | G | G | G | C | G | A | A | A | C | G | T | T | G | - | - | - | - | T | A | C | G | G | - | T | T | G | A | A | A | A | T | G | A | A | C | A | T | A | G | T | C | G | A |
| PC-3p-54311-58 | - | - | - | - | - | - | - | - | T | T | A | T | A | - | - | - | - | - | - | - | C | C | G | A | C | C | G | T | T | G | - | - | - | - | T | A | C | G | G | - | T | C | G | A | A | A | A | T | T | G | A | C | A | T | A | G | T | C | G | A |
| PC-3p-54311-52 | - | - | - | - | - | - | - | - | T | C | A | T | A | - | - | - | - | - | - | - | T | C | G | A | C | C | G | T | T | G | - | - | - | - | T | A | C | G | A | - | T | C | G | A | A | A | A | T | T | G | A | C | A | T | A | G | T | C | G | A |
| PC-3p-54311-56 | - | - | - | - | - | - | - | - | T | C | A | T | A | - | - | - | - | - | - | - | T | C | G | A | C | T | G | T | T | G | - | - | - | - | T | A | C | G | A | - | T | C | G | A | A | A | A | T | T | A | A | C | T | T | A | G | T | C | G | A |
| PC-3p-54311-13 | - | - | - | - | - | - | - | - | T | C | A | T | A | - | - | - | - | - | - | - | C | C | G | A | C | C | G | T | T | G | - | - | - | - | T | A | C | G | G | - | T | C | G | A | A | A | A | T | T | A | A | C | A | T | A | G | T | C | G | A |
| PC-3p-54311-33 | - | - | - | - | - | - | - | - | T | T | A | T | A | - | - | - | - | - | - | - | C | C | G | A | C | C | G | T | T | G | T | A | T | A | T | A | C | A | G | - | T | C | G | A | A | A | A | T | T | A | A | C | A | T | A | G | T | C | G | A |
| PC-3p-54311-25 | - | - | - | - | - | - | - | - | T | C | A | T | A | - | - | - | - | - | - | - | C | T | G | A | C | C | G | T | T | G | - | - | - | - | C | A | C | G | G | - | T | C | G | A | A | A | A | T | T | A | A | C | A | T | A | G | T | C | G | A |
| PC-3p-54311-34 | - | - | - | - | - | - | - | - | G | A | A | T | T | T | G | - | - | - | - | - | G | C | G | A | C | C | G | G | T | A | - | - | - | - | C | A | C | A | G | - | T | C | G | A | A | C | A | T | T | A | A | C | G | T | A | G | T | C | G | A |
| PC-3p-54311-54 | - | - | - | - | - | - | - | - | G | A | A | T | T | T | G | - | - | - | - | - | G | C | G | A | C | C | G | G | T | A | - | - | - | - | C | A | A | G | G | G | T | C | G | A | A | C | A | T | T | A | A | C | G | T | A | G | T | C | G | A |
| PC-3p-54311-8 | - | - | - | - | - | - | - | - | G | A | A | T | T | T | G | - | - | - | - | - | G | C | G | A | C | C | G | G | T | A | - | - | - | - | T | A | C | A | G | - | C | C | G | A | A | C | A | T | T | A | A | C | G | T | A | G | T | C | G | A |
| PC-3p-54311-32 | - | - | - | - | - | - | - | - | G | A | A | T | T | T | G | - | - | - | - | - | G | C | G | A | C | C | G | G | T | A | - | - | - | - | C | A | C | A | G | - | T | C | G | A | A | C | A | T | T | A | A | C | G | T | A | G | T | C | G | A |
| PC-3p-54311-57 | - | - | - | - | - | - | - | - | G | A | A | T | T | T | G | - | - | - | - | - | G | C | G | A | C | C | A | G | T | A | - | - | - | - | C | A | C | A | G | - | T | C | G | A | A | C | A | T | T | A | A | C | G | T | A | G | T | C | G | A |
| PC-3p-54311-44 | - | - | - | - | - | - | - | - | G | A | A | T | T | T | G | - | - | - | - | - | G | C | A | A | C | C | G | G | T | A | - | - | - | - | C | A | C | A | G | - | T | C | G | A | A | C | A | T | T | A | A | C | G | T | A | G | T | C | G | A |
| PC-3p-54311-26 | - | - | - | - | - | - | - | - | G | A | A | T | T | C | G | - | - | - | - | - | G | C | G | A | C | C | G | A | T | A | - | - | - | - | C | A | C | A | G | - | T | C | G | A | A | C | A | T | T | A | A | C | A | T | A | G | T | C | G | A |
| PC-3p-54311-61 | - | - | - | - | - | - | - | - | G | A | A | T | T | T | G | - | - | - | - | - | G | C | G | A | C | C | G | A | T | A | - | - | - | - | C | A | C | A | G | - | T | C | G | A | A | A | A | T | T | A | A | C | G | T | A | G | T | C | G | A |
| PC-3p-54311-47 | - | - | - | - | - | - | - | - | G | A | A | T | T | T | G | - | - | - | - | - | G | C | G | A | T | T | G | G | T | A | - | - | - | - | C | A | C | A | G | - | T | C | G | A | A | C | A | T | T | A | A | C | G | T | A | G | T | C | G | A |
| PC-3p-54311-63 | - | - | - | - | - | - | - | - | G | A | A | T | T | T | G | - | - | - | - | - | G | C | G | A | C | C | G | G | T | A | - | - | - | - | A | A | C | A | G | - | T | C | G | A | A | C | A | T | T | A | A | C | G | T | A | G | T | C | G | A |
| PC-3p-54311-60 | - | - | - | - | - | - | - | - | G | A | A | T | T | T | A | - | - | - | - | - | G | C | G | A | C | C | G | G | T | A | - | - | - | - | C | A | C | A | G | - | T | C | A | A | A | C | A | T | T | A | A | C | G | T | A | G | T | C | G | A |
| PC-3p-54311-38 | - | - | - | - | - | - | - | - | G | A | A | T | T | C | A | - | - | - | - | - | G | C | G | A | C | C | G | G | T | A | - | - | - | - | C | A | C | A | G | - | T | T | G | A | A | C | A | T | T | A | A | C | G | T | A | G | T | C | G | A |
| PC-3p-54311-17 | - | - | - | - | - | - | - | - | G | A | T | T | T | G | G | - | - | - | - | - | G | C | G | A | C | C | G | G | T | A | - | - | - | - | C | T | C | A | G | - | T | C | G | A | A | C | A | T | T | A | A | C | G | T | A | G | T | C | G | A |
| PC-3p-54311-36 | - | - | - | - | - | - | - | - | G | A | A | T | T | T | G | - | - | - | - | - | G | C | G | A | C | T | G | G | T | A | - | - | - | - | C | A | C | A | G | - | T | C | G | A | A | A | A | T | C | A | A | C | G | T | A | G | T | C | G | A |
| PC-3p-54311-12 | - | - | - | - | - | - | - | - | A | A | A | T | T | T | A | - | - | - | - | - | G | C | G | A | C | A | G | G | T | A | - | - | - | - | C | A | C | A | G | - | T | C | G | A | A | T | A | T | T | A | A | C | G | T | A | G | T | C | G | A |
| PC-3p-54311-3 | - | - | - | - | - | - | - | - | T | C | A | T | A | T | - | - | - | - | - | - | - | C | G | A | C | T | G | T | T | G | - | - | - | - | T | A | C | G | G | - | T | C | G | A | A | A | A | T | T | A | A | C | A | T | A | G | T | C | G | A |
| PC-3p-54311-10 | - | - | - | - | - | - | - | - | T | C | A | T | A | T | - | - | - | - | - | - | - | C | G | A | C | T | G | T | T | G | - | - | - | - | T | A | C | G | G | - | T | C | G | A | A | A | A | T | T | A | A | C | A | T | A | G | T | C | G | A |
| PC-3p-54311-22 | - | - | - | - | - | - | - | - | T | C | A | T | A | T | - | - | - | - | - | - | - | C | G | A | C | C | G | T | T | G | - | - | - | - | T | A | C | G | G | - | T | C | G | A | A | A | A | T | T | A | A | C | A | T | A | G | T | C | G | A |
| PC-3p-54311-49 | - | - | - | - | - | - | - | - | T | C | A | T | A | T | - | - | - | - | - | - | - | C | G | A | C | T | G | T | T | G | - | - | - | - | T | A | C | G | G | - | T | C | G | A | A | A | A | T | T | A | A | C | A | T | A | A | T | C | G | A |
| PC-3p-54311-1 | - | - | - | - | - | - | - | - | T | C | A | T | A | T | - | - | - | - | - | - | - | C | G | A | C | T | G | T | T | G | - | - | - | - | T | A | C | G | G | - | T | C | G | A | A | A | A | T | T | A | A | C | A | T | A | G | T | C | G | A |
| PC-3p-54311-55 | - | - | - | - | - | - | - | - | C | C | A | T | A | T | - | - | - | - | - | - | - | C | G | A | C | T | G | T | T | G | - | - | - | - | T | A | C | G | A | - | T | C | G | A | A | A | A | T | T | A | A | C | T | T | A | G | T | C | G | A |
| PC-3p-54311-2 | - | - | - | - | - | - | - | - | T | C | A | T | A | T | - | - | - | - | - | - | - | C | G | A | C | T | G | T | T | G | - | - | - | - | T | A | C | G | G | - | T | C | G | A | A | A | A | T | T | A | A | C | A | T | A | G | T | C | G | A |
| PC-3p-54311-9 | - | - | - | - | - | - | - | - | T | C | A | T | A | T | - | - | - | - | - | - | - | C | G | A | C | T | G | T | T | G | - | - | - | - | T | A | C | G | G | - | T | C | G | A | A | A | A | T | T | A | A | C | A | T | A | G | T | C | G | A |
| PC-3p-54311-20 | - | - | - | - | - | - | - | - | T | C | A | T | A | T | - | - | - | - | - | - | - | C | G | A | C | T | G | T | T | G | - | - | - | - | T | A | C | G | G | - | T | C | G | A | A | A | A | T | T | A | A | C | A | T | A | G | T | C | G | A |
| PC-3p-54311-18 | - | - | - | - | - | - | - | - | T | C | A | T | A | T | - | - | - | - | - | - | - | C | G | A | C | T | G | T | T | G | - | - | - | - | T | A | C | G | G | - | C | C | G | A | A | A | A | T | T | A | A | C | A | T | A | G | T | C | G | A |
| PC-3p-54311-37 | - | - | - | - | - | - | - | - | T | T | A | T | A | T | - | - | - | - | - | - | - | C | G | A | C | T | G | T | T | G | - | - | - | - | T | A | C | G | G | - | T | C | A | A | A | A | A | T | T | A | A | C | A | T | A | G | T | C | G | A |
| PC-3p-54311-45 | - | - | - | - | - | - | - | - | T | C | A | T | A | T | - | - | - | - | - | - | - | C | G | A | C | T | G | T | T | G | - | - | - | - | T | A | C | G | G | - | T | C | G | A | A | A | A | T | T | A | A | C | A | T | A | G | T | C | C | A |
| PC-3p-54311-35 | - | - | - | - | - | - | - | - | T | C | A | T | A | T | - | - | - | - | - | - | - | C | G | A | C | T | G | T | T | G | - | - | - | - | T | A | C | G | G | - | T | C | G | A | A | A | A | T | T | A | A | C | A | T | A | G | T | C | G | A |
| PC-3p-54311-41 | - | - | - | - | - | - | - | - | T | C | A | T | A | T | - | - | - | - | - | - | - | C | G | A | C | T | - | - | - | - | - | - | - | - | - | - | - | - | - | - | - | - | - | A | A | A | A | T | T | A | A | C | A | T | A | G | T | C | G | A |
| PC-3p-54311-40 | - | - | - | - | - | - | - | - | T | C | A | T | A | T | - | - | - | - | - | - | - | C | G | A | C | T | G | T | T | G | - | - | - | - | T | A | C | G | A | - | T | C | G | G | A | A | A | T | T | A | A | C | A | T | A | G | T | C | G | A |
| PC-3p-54311-4 | - | - | - | - | - | - | - | - | T | C | A | T | A | T | - | - | - | - | - | - | - | C | G | A | C | T | G | T | T | G | - | - | - | - | T | A | C | G | G | - | T | C | G | A | A | A | A | T | T | A | A | C | A | T | A | G | T | C | G | A |
| PC-3p-54311-42 | - | - | - | - | - | - | - | - | T | C | A | T | A | T | - | - | - | - | - | - | - | C | G | A | C | T | G | T | T | G | - | - | - | - | T | A | C | G | G | - | T | C | G | A | A | A | A | T | T | A | A | C | A | T | A | G | T | C | G | A |
| PC-3p-54311-16 | - | - | - | - | - | - | - | - | T | C | A | T | A | T | - | - | - | - | - | - | - | C | G | A | A | T | G | T | T | G | - | - | - | - | T | A | C | G | G | - | T | C | G | A | A | A | A | T | T | A | A | C | A | T | A | G | T | C | G | A |
| PC-3p-54311-27 | - | - | - | - | - | - | - | - | T | C | A | T | A | T | - | - | - | - | - | - | - | C | G | A | C | T | A | T | T | G | - | - | - | - | T | A | C | G | G | - | T | C | G | A | A | A | A | T | T | A | A | C | A | T | A | G | T | C | G | A |
| PC-3p-54311-15 | - | - | - | - | - | - | - | - | T | C | A | T | A | T | - | - | - | - | - | - | - | C | G | A | C | T | G | T | T | G | - | - | - | - | T | A | C | G | G | - | T | C | G | A | A | A | A | T | T | A | A | C | A | T | A | G | T | C | G | A |
| PC-3p-54311-14 | - | - | - | - | - | - | - | - | T | C | A | T | A | T | - | - | - | - | - | - | - | C | G | A | C | T | G | T | T | G | - | - | - | - | T | A | C | G | G | - | T | C | G | A | A | A | A | T | T | A | A | C | A | T | A | G | T | C | G | A |
| PC-3p-54311-21 | - | - | - | - | - | - | - | - | T | C | A | T | A | T | - | - | - | - | - | - | - | A | G | A | C | T | G | T | T | G | - | - | - | - | T | A | C | G | G | - | T | C | G | A | A | A | A | T | T | A | A | C | A | T | A | G | T | C | G | A |
| PC-3p-54311-24 | - | - | - | - | - | - | - | - | T | C | A | T | A | T | - | - | - | - | - | - | - | C | G | A | C | T | G | T | T | G | - | - | - | - | T | A | C | G | G | - | T | C | G | A | A | A | A | T | T | A | A | C | A | T | A | G | T | C | G | A |
| PC-3p-54311-65 | - | - | - | - | - | - | - | - | T | C | A | T | A | T | - | - | - | - | - | - | - | C | G | A | C | T | G | T | T | G | - | - | - | - | T | A | C | G | G | - | T | C | A | A | A | A | A | T | T | A | A | C | A | T | A | T | T | C | G | A |
| PC-3p-54311-28 | - | - | - | - | - | - | - | - | T | C | A | T | A | T | - | - | - | - | - | - | - | C | G | A | C | T | G | T | T | G | - | - | - | - | T | A | C | G | G | - | T | C | G | A | A | A | A | T | T | A | A | C | A | T | A | G | T | C | G | A |
| PC-3p-54311-50 | - | - | - | - | - | - | - | - | T | T | A | T | A | T | - | - | - | - | - | - | - | A | G | A | C | T | G | T | T | G | - | - | - | - | T | A | C | G | G | - | T | C | G | A | A | A | A | T | T | A | A | C | A | T | A | G | T | T | G | A |
| PC-3p-54311-30 | T | A | T | G | A | A | A | T | T | C | A | T | A | T | - | - | - | - | - | - | - | C | G | A | C | C | G | T | T | T | - | - | - | - | T | A | C | G | G | - | T | C | G | A | A | A | A | T | T | A | A | C | A | T | A | G | T | C | G | A |
| PC-3p-54311-11 | - | - | - | - | - | - | - | - | T | C | A | T | A | C | - | - | - | - | - | - | - | C | G | A | C | C | G | T | T | G | - | - | - | - | T | A | C | G | G | - | T | C | G | A | A | A | A | T | T | A | A | C | A | T | A | G | T | C | G | A |
| PC-3p-54311-19 | - | - | - | - | - | - | - | - | T | C | A | T | A | T | - | - | - | - | - | - | - | C | G | A | C | C | G | T | T | G | - | - | - | - | T | A | C | G | G | - | T | C | G | A | A | A | A | T | T | A | A | C | A | T | A | G | T | C | G | A |
| PC-3p-54311-23 | - | - | - | - | - | - | - | - | T | C | A | T | A | T | - | - | - | - | - | - | - | C | G | A | C | T | G | T | T | G | - | - | - | - | T | A | C | G | G | - | T | C | G | A | A | A | A | T | T | A | A | C | A | T | A | G | T | C | G | A |
| PC-3p-54311-29 | - | - | - | - | - | - | - | - | T | C | A | T | A | T | - | - | - | - | - | - | - | C | G | A | C | T | G | T | T | G | - | - | - | - | T | A | C | G | G | - | T | C | G | A | A | A | A | T | T | A | A | C | A | T | A | G | T | C | G | A |
| PC-3p-54311-31 | - | - | - | - | - | - | - | - | T | C | A | T | A | T | - | - | - | - | - | - | - | C | G | A | C | C | G | T | T | G | - | - | - | - | T | A | C | G | G | - | T | C | G | A | A | A | A | T | T | A | A | C | A | T | A | G | T | C | G | A |
| PC-3p-54311-6 | - | - | - | - | - | - | - | - | G | C | A | T | A | C | - | - | - | - | - | - | - | C | G | C | T | C | G | T | T | G | - | - | - | - | T | A | C | G | G | - | T | T | G | A | A | A | A | T | T | A | A | C | A | T | A | G | T | C | G | A |
| PC-3p-54311-64 | - | - | - | - | - | - | - | - | T | C | A | T | A | A | - | - | - | - | - | - | - | C | A | A | T | T | G | T | T | T | - | - | - | - | C | A | C | G | A | - | T | C | G | A | A | A | A | T | T | A | A | C | G | T | A | G | T | C | G | A |

**┤miRNA ends**

| PC-3p-54311-5 | C | T | G | - | T | T | C | T | G | A | A | G | - | - | - | - | - | - | - | - | - | - | - | - | - | - | T | C | G | T | G | T | C | C | G | A | T | A | T | T | T | C | G | A | C | T | T | T | G | - | A | T | T | A | G | - | - | - | T | C |
| --- | --- | --- | --- | --- | --- | --- | --- | --- | --- | --- | --- | --- | --- | --- | --- | --- | --- | --- | --- | --- | --- | --- | --- | --- | --- | --- | --- | --- | --- | --- | --- | --- | --- | --- | --- | --- | --- | --- | --- | --- | --- | --- | --- | --- | --- | --- | --- | --- | --- | --- | --- | --- | --- | --- | --- | --- | --- | --- | --- | --- |
| PC-3p-54311-46 | C | T | G | - | T | T | C | T | G | A | A | G | - | - | - | - | - | - | - | - | - | - | - | - | - | - | T | C | G | T | G | T | C | C | G | A | T | A | T | T | T | C | G | A | C | T | T | T | G | - | A | C | A | A | G | - | - | - | T | C |
| PC-3p-54311-7 | C | T | G | - | T | T | C | T | G | A | A | G | - | - | - | - | - | - | - | - | - | - | - | - | - | - | T | C | G | T | G | T | C | C | G | A | T | A | T | T | T | C | G | A | C | T | T | T | G | - | A | C | A | A | G | - | - | - | T | C |
| PC-3p-54311-39 | C | T | G | - | T | T | C | T | G | A | A | G | T | C | T | G | - | - | - | - | - | - | - | - | - | - | A | A | G | T | T | T | C | A | A | T | G | G | A | G | T | T | G | A | C | A | A | C | A | - | - | A | A | A | T | - | - | - | T | T |
| PC-3p-54311-66 | C | T | - | - | C | A | G | T | G | A | G | G | - | G | T | A | - | - | - | - | - | - | - | - | - | - | A | T | C | A | A | T | C | T | A | T | - | A | T | G | T | C | T | C | C | A | T | T | G | - | - | C | G | A | T | - | - | - | T | T |
| PC-3p-54311-51 | T | T | T | T | C | G | C | A | A | T | A | T | A | C | C | T | - | - | - | - | - | - | - | - | - | - | T | T | C | T | A | A | A | A | A | A | A | A | A | G | A | T | T | A | T | C | T | T | G | - | - | C | A | C | T | - | - | - | A | A |
| PC-3p-54311-59 | C | T | G | - | T | T | T | T | G | A | A | G | - | - | - | - | - | - | - | - | - | - | - | - | - | - | T | C | G | T | G | T | C | C | A | A | T | G | T | T | T | C | T | T | T | C | G | T | T | - | - | T | T | T | T | - | - | - | T | A |
| PC-3p-54311-43 | C | T | G | - | T | T | C | T | G | A | A | G | T | C | G | T | G | T | C | C | G | A | T | A | T | T | T | C | G | A | T | T | C | T | G | A | C | A | A | G | T | C | G | A | A | A | T | G | A | G | T | T | T | G | T | - | - | - | T | A |
| PC-3p-54311-48 | C | T | G | - | T | T | C | T | G | A | A | G | T | C | G | T | - | - | - | - | - | - | - | - | - | - | - | - | - | - | - | - | T | T | G | A | T | A | A | G | T | C | G | A | A | A | T | G | A | G | T | T | T | G | T | - | - | - | T | A |
| PC-3p-54311-53 | C | T | G | - | T | T | C | T | G | A | A | G | - | - | - | - | - | - | - | - | - | - | - | - | - | - | T | C | G | T | G | T | C | C | G | A | T | A | T | T | T | C | G | A | C | T | T | T | G | - | A | C | C | T | G | - | - | - | T | C |
| PC-3p-54311-62 | C | T | A | - | T | T | C | T | G | T | A | G | - | - | - | - | - | - | - | - | - | - | - | - | - | - | T | C | G | T | T | T | A | C | G | A | T | T | T | T | T | T | G | A | C | T | T | T | G | - | A | C | T | A | G | - | - | - | T | C |
| PC-3p-54311-58 | C | T | G | - | T | T | C | T | G | A | A | G | - | - | - | - | - | - | - | - | - | - | - | - | - | - | T | C | A | T | G | C | C | C | G | A | T | A | T | T | T | C | G | A | C | T | T | T | G | - | A | G | A | A | G | - | - | - | T | C |
| PC-3p-54311-52 | C | T | G | - | T | T | C | T | G | A | A | G | - | - | - | - | - | - | - | - | - | - | - | - | - | - | T | C | G | T | G | T | C | C | G | A | T | A | T | T | T | C | G | A | C | T | T | T | G | - | A | C | A | A | G | - | - | - | T | C |
| PC-3p-54311-56 | C | T | G | - | T | T | C | T | G | A | A | G | - | - | - | - | - | - | - | - | - | - | - | - | - | - | T | C | G | T | G | T | C | C | G | A | T | A | T | T | T | T | G | A | C | T | T | T | G | - | A | C | A | A | G | - | - | - | T | G |
| PC-3p-54311-13 | C | T | G | - | T | T | C | T | G | A | A | - | - | - | - | - | - | - | - | - | - | - | - | - | - | G | T | C | G | T | G | T | C | C | G | A | T | A | T | T | A | C | G | A | C | T | T | T | G | - | A | C | A | A | G | - | - | - | T | C |
| PC-3p-54311-33 | C | T | G | - | T | T | C | T | G | A | A | C | - | - | - | - | - | - | - | - | - | - | - | - | T | G | T | C | G | T | G | T | T | C | G | A | T | A | T | T | T | C | G | A | C | T | T | T | G | - | A | C | T | A | G | - | - | - | T | C |
| PC-3p-54311-25 | C | T | G | - | T | T | C | T | G | A | A | - | - | - | - | - | - | - | - | - | - | - | - | - | - | G | T | C | G | T | T | T | C | C | G | A | T | A | C | T | T | C | G | A | C | T | T | T | G | - | C | C | A | A | G | - | - | - | T | C |
| PC-3p-54311-34 | C | T | G | - | T | T | C | T | G | A | A | G | - | - | - | - | - | - | - | - | - | - | - | - | - | - | T | C | G | T | G | T | C | C | G | A | T | A | T | T | T | C | G | A | C | T | - | T | T | G | A | C | T | A | G | - | - | - | T | C |
| PC-3p-54311-54 | C | T | G | - | T | T | C | T | G | A | A | G | - | - | - | - | - | - | - | - | - | - | - | - | - | - | T | C | G | T | G | T | C | C | G | A | T | A | T | T | T | C | A | A | C | T | - | T | T | G | A | C | T | A | G | - | - | - | T | C |
| PC-3p-54311-8 | C | T | G | - | T | T | C | T | G | A | A | G | - | - | - | - | - | - | - | - | - | - | - | - | - | - | T | C | G | T | G | T | C | C | G | A | T | A | T | T | T | C | G | A | C | T | - | T | T | G | A | G | T | A | G | - | - | - | T | C |
| PC-3p-54311-32 | C | T | G | - | T | T | C | T | G | A | A | G | - | - | - | - | - | - | - | - | - | - | - | - | - | - | T | C | G | T | G | T | C | C | G | A | T | A | T | T | T | C | G | A | C | T | - | T | T | G | A | C | T | A | G | - | - | - | T | C |
| PC-3p-54311-57 | A | T | G | - | T | T | C | T | G | T | A | G | - | - | - | - | - | - | - | - | - | - | - | - | - | - | T | C | G | T | G | T | C | C | G | A | T | A | T | T | T | C | G | A | C | T | - | T | T | G | A | C | T | A | T | - | - | - | T | C |
| PC-3p-54311-44 | C | T | G | - | T | T | C | T | G | A | A | G | - | - | - | - | - | - | - | - | - | - | - | - | - | - | T | C | G | T | G | T | C | C | G | A | T | A | T | T | T | C | G | A | C | T | - | T | T | G | A | C | T | T | G | - | - | - | T | C |
| PC-3p-54311-26 | C | T | G | - | T | T | C | T | G | A | A | G | - | - | - | - | - | - | - | - | - | - | - | - | - | - | T | C | G | T | G | T | C | C | G | A | T | A | T | T | T | C | G | A | C | T | - | T | T | G | C | C | A | A | G | - | - | - | T | C |
| PC-3p-54311-61 | C | T | G | - | T | T | C | T | G | A | A | A | - | - | - | - | - | - | - | - | - | - | - | - | - | - | T | C | G | T | G | T | C | C | G | A | T | A | T | T | T | C | G | A | C | T | - | T | T | G | A | C | T | A | G | - | - | - | T | C |
| PC-3p-54311-47 | C | T | G | - | T | T | C | T | G | A | A | G | - | - | - | - | - | - | - | - | - | - | - | - | - | - | C | C | G | T | G | T | C | C | G | A | T | A | T | T | T | G | G | A | C | T | - | T | T | G | A | C | T | A | G | - | - | - | T | C |
| PC-3p-54311-63 | C | T | G | - | T | T | C | T | G | A | A | G | - | - | - | - | - | - | - | - | - | - | - | - | - | - | T | T | G | T | G | T | C | C | A | A | T | A | T | T | T | C | G | G | C | T | G | A | T | G | G | C | T | T | G | - | - | - | T | G |
| PC-3p-54311-60 | C | T | G | - | T | C | C | T | G | A | A | T | - | - | - | - | - | - | - | - | - | - | - | - | - | - | T | C | G | T | G | T | C | C | G | A | T | A | T | T | T | C | G | A | C | T | - | T | T | G | A | C | T | A | G | - | - | - | T | C |
| PC-3p-54311-38 | C | T | G | - | T | T | C | T | G | A | A | G | - | - | - | - | - | - | - | - | - | - | - | - | - | - | A | C | G | T | G | T | C | C | G | A | T | A | T | T | T | C | G | A | C | T | - | T | T | G | A | C | T | G | G | - | - | - | T | C |
| PC-3p-54311-17 | C | T | G | - | T | T | C | T | G | A | A | G | - | - | - | - | - | - | - | - | - | - | - | - | - | - | T | C | G | T | G | T | C | C | G | A | T | A | T | C | T | C | A | A | C | T | - | T | T | G | A | C | T | A | G | - | - | - | T | C |
| PC-3p-54311-36 | C | T | G | - | T | T | C | T | G | A | A | G | - | - | - | - | - | - | - | - | - | - | - | - | - | - | T | C | G | T | G | T | C | C | G | A | T | A | T | T | T | C | G | A | C | G | - | A | A | A | A | G | T | A | G | T | A | G | T | C |
| PC-3p-54311-12 | C | T | G | - | T | T | C | T | G | A | A | G | - | - | - | - | - | - | - | - | - | - | - | - | - | - | T | C | T | T | G | T | C | C | A | A | T | A | T | T | T | C | G | A | C | T | - | T | T | T | A | C | T | A | G | - | - | - | T | C |
| PC-3p-54311-3 | C | T | G | - | T | T | C | T | G | G | A | G | - | - | - | - | - | - | - | - | - | - | - | - | - | - | T | C | G | T | G | T | C | C | A | A | T | A | T | T | T | C | G | A | C | T | T | T | - | G | A | C | A | A | G | - | - | - | T | C |
| PC-3p-54311-10 | C | T | G | - | T | T | C | T | G | A | A | G | - | - | - | - | - | - | - | - | - | - | - | - | - | - | T | C | G | T | G | T | C | C | G | A | T | A | T | T | T | C | G | A | C | T | T | T | - | G | A | C | A | A | G | - | - | - | T | T |
| PC-3p-54311-22 | C | T | G | - | T | T | C | T | G | A | A | G | - | - | - | - | - | - | - | - | - | - | - | - | - | - | T | C | G | T | G | T | C | C | G | A | T | A | T | T | T | C | G | A | C | T | T | T | - | G | A | C | T | A | G | - | - | - | T | C |
| PC-3p-54311-49 | C | T | G | - | T | T | C | T | G | A | A | G | - | - | - | - | - | - | - | - | - | - | - | - | - | - | T | C | G | T | G | T | C | C | G | A | T | A | T | T | T | C | G | A | C | C | T | T | - | G | A | C | A | A | G | - | - | - | T | C |
| PC-3p-54311-1 | C | T | G | - | T | T | C | T | G | A | A | G | - | - | - | - | - | - | - | - | - | - | - | - | - | - | C | T | G | T | G | T | C | C | G | A | T | A | T | T | T | C | G | A | C | T | T | T | - | G | A | C | A | A | G | - | - | - | T | C |
| PC-3p-54311-55 | C | T | G | - | T | T | C | T | G | A | A | G | - | - | - | - | - | - | - | - | - | - | - | - | - | - | T | C | G | T | G | T | C | C | G | A | T | A | T | T | T | C | G | A | C | T | T | T | - | G | A | C | A | A | G | - | - | - | T | C |
| PC-3p-54311-2 | C | T | G | - | T | T | C | T | G | A | A | G | - | - | - | - | - | - | - | - | - | - | - | - | - | - | T | C | G | T | G | T | C | C | G | A | T | A | T | T | T | T | G | A | C | T | T | T | - | G | A | C | T | A | G | - | - | - | T | C |
| PC-3p-54311-9 | C | T | G | - | T | T | C | T | G | A | A | G | - | - | - | - | - | - | - | - | - | - | - | - | - | - | T | T | G | T | G | T | C | C | G | A | T | A | T | T | T | C | A | A | C | T | T | T | - | G | A | C | T | A | G | - | - | - | T | C |
| PC-3p-54311-20 | C | T | G | - | T | T | C | T | G | A | A | G | - | - | - | - | - | - | - | - | - | - | - | - | - | - | T | C | G | T | G | T | C | C | G | A | T | A | T | T | T | C | G | A | C | T | T | T | - | G | A | C | A | A | G | - | - | - | T | C |
| PC-3p-54311-18 | C | T | G | - | T | T | C | C | G | A | A | G | - | - | - | - | - | - | - | - | - | - | - | - | - | - | T | C | G | C | A | T | C | C | G | A | T | A | T | T | T | C | G | A | C | T | T | T | - | G | A | C | A | A | G | - | - | - | T | C |
| PC-3p-54311-37 | C | T | G | - | T | T | C | T | G | A | A | A | - | - | - | - | - | - | - | - | - | - | - | - | - | - | T | C | G | - | - | - | - | - | - | - | - | - | - | - | - | - | - | - | - | - | - | - | - | - | - | - | - | T | G | - | - | - | T | C |
| PC-3p-54311-45 | C | T | G | - | T | T | C | T | G | A | A | G | - | - | - | - | - | - | - | - | - | - | - | - | - | - | T | C | A | T | G | T | C | C | G | A | T | A | T | T | T | C | G | A | C | T | T | T | - | G | A | C | A | A | G | - | - | - | T | C |
| PC-3p-54311-35 | C | T | G | - | T | T | C | T | G | A | A | G | - | - | - | - | - | - | - | - | - | - | - | - | - | - | T | C | G | T | G | T | C | C | G | A | T | A | T | T | T | C | T | A | C | T | T | T | - | G | A | C | A | A | G | - | - | - | T | C |
| PC-3p-54311-41 | C | C | G | - | T | T | C | T | G | A | A | G | - | - | - | - | - | - | - | - | - | - | - | - | - | - | T | C | G | T | G | T | C | C | A | A | T | A | T | T | T | C | G | G | C | T | T | T | - | G | A | C | A | A | G | - | - | - | T | C |
| PC-3p-54311-40 | C | T | G | - | T | T | C | T | A | A | A | G | - | - | - | - | - | - | - | - | - | - | - | - | - | - | T | C | G | T | G | T | C | C | G | A | T | A | T | T | T | C | G | A | C | T | T | T | - | G | C | C | T | A | G | - | - | - | T | C |
| PC-3p-54311-4 | C | T | G | - | T | T | C | T | G | A | A | G | - | - | - | - | - | - | - | - | - | - | - | - | - | - | T | C | G | T | G | T | C | C | G | A | T | A | T | T | T | C | G | A | C | T | T | T | - | G | A | C | A | A | G | - | - | - | T | C |
| PC-3p-54311-42 | G | T | G | - | T | T | C | T | G | A | A | G | - | - | - | - | - | - | - | - | - | - | - | - | - | - | T | C | G | T | G | T | C | C | G | A | T | A | T | T | T | C | G | A | C | T | T | T | - | G | A | C | A | A | G | - | - | - | T | C |
| PC-3p-54311-16 | C | T | G | - | T | T | C | T | G | A | A | G | - | - | - | - | - | - | - | - | - | - | - | - | - | - | T | C | G | T | G | T | C | C | G | A | T | A | T | T | T | C | G | A | C | T | T | T | - | G | A | C | A | A | G | - | - | - | T | C |
| PC-3p-54311-27 | C | T | G | - | T | T | C | T | G | A | A | G | - | - | - | - | - | - | - | - | - | - | - | - | - | - | T | C | G | T | A | T | C | C | G | A | T | C | T | T | T | C | G | A | C | T | T | T | - | G | A | C | A | A | G | - | - | - | T | C |
| PC-3p-54311-15 | C | T | G | - | T | T | C | T | G | A | A | G | - | - | - | - | - | - | - | - | - | - | - | - | - | - | T | C | G | T | G | T | C | C | G | A | T | A | T | T | T | C | G | A | C | T | T | T | T | G | A | C | A | A | G | - | - | - | T | C |
| PC-3p-54311-14 | C | T | G | - | T | T | C | T | G | A | A | G | - | - | - | - | - | - | - | - | - | - | - | - | - | - | T | C | G | T | G | T | C | C | G | A | T | A | T | T | T | C | G | A | C | T | T | T | G | A | C | A | A | - | G | - | - | - | T | C |
| PC-3p-54311-21 | C | T | G | - | T | T | C | T | G | A | A | G | - | - | - | - | - | - | - | - | - | - | - | - | - | - | T | C | G | T | G | T | C | C | G | A | T | A | T | T | T | T | G | A | C | T | T | T | G | A | C | A | A | - | G | - | - | - | T | C |
| PC-3p-54311-24 | C | T | G | - | T | T | C | T | G | A | A | G | - | - | - | - | - | - | - | - | - | - | - | - | - | - | T | C | G | T | G | T | C | C | G | A | T | A | T | T | T | C | G | A | C | A | T | T | G | A | C | A | A | - | G | - | - | - | T | C |
| PC-3p-54311-65 | C | T | G | - | T | T | C | T | G | A | A | G | - | - | - | - | - | - | - | - | - | - | - | - | - | - | T | C | G | T | G | T | C | C | G | A | T | A | T | T | T | C | G | A | C | T | T | T | G | A | A | A | A | T | G | - | - | - | T | C |
| PC-3p-54311-28 | C | T | G | - | T | T | C | T | G | A | A | G | - | - | - | - | - | - | - | - | - | - | - | - | - | - | T | C | G | T | G | T | C | C | G | A | T | A | T | T | T | C | G | A | C | A | T | T | G | A | C | A | A | - | G | - | - | - | T | C |
| PC-3p-54311-50 | C | T | G | - | T | T | C | T | G | A | A | G | - | - | - | - | - | - | - | - | - | - | - | - | - | - | T | C | G | T | G | T | C | C | G | A | T | A | T | T | T | C | G | A | C | T | T | T | G | A | C | A | A | - | G | - | - | - | C | C |
| PC-3p-54311-30 | C | T | G | - | T | T | C | T | G | A | A | G | - | - | - | - | - | - | - | - | - | - | - | - | - | - | T | C | G | T | G | T | C | C | G | A | T | A | T | T | T | C | G | A | C | T | T | T | - | G | A | C | A | A | G | - | - | - | T | C |
| PC-3p-54311-11 | C | T | G | - | T | T | A | T | G | A | A | G | - | - | - | - | - | - | - | - | - | - | - | - | - | - | T | T | G | T | G | T | C | C | G | A | T | A | T | T | T | C | G | A | C | T | T | T | G | - | A | C | A | A | G | - | - | - | T | C |
| PC-3p-54311-19 | C | T | G | - | T | T | C | T | G | A | A | G | - | - | - | - | - | - | - | - | - | - | - | - | - | - | T | T | G | T | G | T | C | C | G | A | T | A | T | T | T | C | G | A | C | T | T | T | G | - | A | C | A | A | G | - | - | - | T | C |
| PC-3p-54311-23 | C | T | G | - | T | T | A | T | G | A | A | G | - | - | - | - | - | - | - | - | - | - | - | - | - | - | T | C | G | T | G | T | C | C | G | A | T | A | T | T | T | C | G | A | A | T | T | T | G | - | A | C | A | A | G | - | - | - | T | C |
| PC-3p-54311-29 | C | T | G | - | T | T | C | T | G | A | A | G | - | - | - | - | - | - | - | - | - | - | - | - | - | - | T | C | A | T | G | T | C | C | G | A | T | A | T | T | T | C | G | A | C | T | T | T | C | - | A | C | A | A | G | - | - | - | T | C |
| PC-3p-54311-31 | C | T | G | - | T | T | C | T | G | A | A | G | - | - | - | - | - | - | - | - | - | - | - | - | - | - | T | C | G | T | G | T | C | C | G | A | T | A | T | T | T | C | G | A | C | T | T | T | G | - | A | C | A | A | G | - | - | - | T | C |
| PC-3p-54311-6 | C | T | G | - | T | T | C | T | G | A | A | G | - | - | - | - | - | - | - | - | - | - | - | - | - | - | T | C | G | T | G | T | C | C | G | A | T | A | T | T | T | T | G | A | C | T | T | T | G | - | A | C | A | A | G | - | - | - | T | C |
| PC-3p-54311-64 | C | T | G | - | - | - | - | - | - | - | - | - | - | - | - | - | - | - | - | - | - | - | - | - | - | T | T | T | G | T | G | T | C | C | G | A | T | A | T | T | T | C | G | A | C | T | T | T | G | - | A | C | A | A | G | - | - | - | C | C |

| PC-3p-54311-5 | G | A | A | A | T | G | - | - | A | G | T | T | T | G | T | T | A | A | G | A | G | C | A | A | A | T | T | T | T | - | T | T | T | C | - | A | C | T | G | - | - | - | - | - | - | - | - | - | - | - | - | A | G | T | G | G | T | A | A | A |
| --- | --- | --- | --- | --- | --- | --- | --- | --- | --- | --- | --- | --- | --- | --- | --- | --- | --- | --- | --- | --- | --- | --- | --- | --- | --- | --- | --- | --- | --- | --- | --- | --- | --- | --- | --- | --- | --- | --- | --- | --- | --- | --- | --- | --- | --- | --- | --- | --- | --- | --- | --- | --- | --- | --- | --- | --- | --- | --- | --- | --- |
| PC-3p-54311-46 | G | A | A | A | T | G | - | - | A | G | T | T | T | G | T | T | A | A | A | A | G | C | A | A | A | T | T | T | T | A | T | T | T | C | - | A | C | T | G | - | - | - | - | - | - | - | - | - | - | - | - | A | G | T | G | G | - | - | - | - |
| PC-3p-54311-7 | G | A | A | A | T | G | - | - | A | G | T | T | T | T | T | T | A | A | A | A | G | C | A | A | A | T | T | T | T | T | T | T | T | T | - | A | C | T | G | - | - | - | - | - | - | - | - | - | - | - | - | A | G | T | G | C | - | - | - | - |
| PC-3p-54311-39 | T | A | T | A | T | T | - | - | G | G | T | T | G | A | A | T | - | - | T | T | A | C | A | G | C | A | A | C | A | A | T | C | G | A | - | A | C | G | - | - | - | - | A | - | - | - | - | - | - | - | - | A | T | G | A | C | G | C | A | C |
| PC-3p-54311-66 | T | T | T | A | G | T | - | - | A | A | T | T | C | A | C | C | G | A | T | G | A | T | A | G | C | A | C | C | A | T | C | T | A | A | - | G | C | T | - | - | - | - | C | - | - | - | - | - | - | - | - | T | A | T | A | T | A | C | G | C |
| PC-3p-54311-51 | A | T | T | C | G | A | - | - | A | A | A | T | C | C | C | A | - | - | T | T | T | T | G | A | T | A | T | G | A | G | A | A | A | T | - | G | C | A | G | - | - | - | A | - | - | - | - | - | - | - | - | A | T | A | T | T | A | C | A | C |
| PC-3p-54311-59 | A | A | A | G | C | A | - | - | A | G | T | T | T | T | T | T | T | T | T | T | A | C | T | G | A | G | C | A | G | T | A | G | A | T | - | A | A | G | T | - | - | - | A | - | - | - | - | - | - | - | - | A | G | C | A | A | A | G | A | C |
| PC-3p-54311-43 | A | A | A | G | C | A | - | - | A | T | T | T | T | T | T | T | T | T | T | T | A | C | T | G | A | G | C | A | A | T | T | A | T | T | - | T | C | C | - | - | - | - | T | - | - | - | - | - | - | - | - | A | A | A | T | A | G | T | T | T |
| PC-3p-54311-48 | A | A | A | G | C | A | - | - | A | A | T | T | T | T | T | T | T | T | T | C | A | C | T | G | A | G | A | A | A | A | G | A | A | A | - | T | T | G | - | - | - | - | C | - | - | - | - | - | - | - | - | A | G | T | A | A | A | T | A | T |
| PC-3p-54311-53 | G | A | A | A | T | G | - | - | A | G | T | T | T | G | T | T | A | A | A | A | G | C | A | A | A | T | T | T | T | T | T | T | T | T | - | C | A | T | T | - | - | - | - | - | - | - | - | - | - | - | - | G | A | C | A | T | G | A | G | T |
| PC-3p-54311-62 | G | A | A | A | T | T | - | - | A | G | T | T | T | G | T | T | A | A | A | A | G | C | A | A | A | T | T | T | T | T | T | T | T | T | - | T | T | T | C | T | A | C | C | - | - | - | - | - | - | - | - | G | A | C | A | T | G | A | G | T |
| PC-3p-54311-58 | G | A | A | A | T | G | - | - | A | A | T | T | T | G | T | T | A | A | A | A | G | C | A | A | A | T | T | T | T | T | T | T | T | T | - | C | A | C | T | - | - | - | G | - | - | - | - | - | - | - | - | A | G | T | A | A | C | A | A | T |
| PC-3p-54311-52 | A | T | A | A | T | G | - | - | A | G | T | T | T | G | T | T | A | A | A | A | G | C | A | A | A | T | T | T | T | T | T | T | T | T | - | T | - | T | T | C | A | C | T | - | - | - | - | - | - | - | - | G | A | G | T | A | C | T | A | G |
| PC-3p-54311-56 | G | A | A | A | T | G | - | - | A | G | T | T | T | G | T | T | A | A | A | A | G | C | A | A | T | T | T | T | T | T | T | C | A | C | - | T | C | T | T | T | T | T | T | - | - | - | - | - | - | - | - | G | A | G | T | A | C | T | G | G |
| PC-3p-54311-13 | G | A | A | A | T | G | - | - | T | A | T | T | T | G | T | T | A | A | A | A | G | C | A | A | A | T | T | T | T | A | T | T | T | - | - | C | A | C | T | G | - | A | G | - | - | - | - | - | - | - | - | T | A | T | A | T | - | - | - | T |
| PC-3p-54311-33 | G | A | A | A | T | G | - | - | A | G | T | T | T | G | T | T | A | A | A | A | G | C | A | A | A | T | T | T | T | T | T | T | T | T | - | C | A | C | T | G | - | A | G | - | - | - | - | - | - | - | - | T | A | T | G | - | - | - | - | A |
| PC-3p-54311-25 | G | A | A | A | T | G | - | - | A | G | T | A | T | G | T | T | A | A | A | A | G | G | A | A | T | T | T | T | T | T | T | T | T | T | - | C | A | C | T | G | - | A | G | - | - | - | - | - | - | - | - | T | A | T | T | T | G | T | A | C |
| PC-3p-54311-34 | G | A | A | A | T | - | - | - | - | - | - | - | - | - | - | - | - | - | - | - | - | - | - | - | - | - | - | - | - | - | - | - | - | - | - | - | - | - | - | - | - | - | - | - | - | - | - | - | - | - | - | - | - | - | - | - | - | - | - | - |
| PC-3p-54311-54 | G | A | A | A | T | G | - | - | A | G | T | G | C | A | T | T | T | A | C | C | G | T | C | A | A | T | T | T | T | T | T | T | T | T | - | A | A | C | T | G | A | A | C | - | - | - | - | - | - | - | - | A | G | T | - | - | T | T | G | C |
| PC-3p-54311-8 | G | A | A | A | T | G | - | - | A | G | T | G | C | G | T | T | T | A | A | C | G | T | C | A | A | A | T | T | T | T | T | T | T | T | - | T | A | T | T | G | A | G | C | - | - | - | - | - | - | - | - | A | G | T | C | A | T | A | A | C |
| PC-3p-54311-32 | G | A | A | A | T | - | - | - | - | - | - | - | - | - | - | - | - | - | - | - | - | - | - | - | - | - | - | - | - | - | - | - | - | - | - | - | - | - | - | - | - | - | - | - | - | - | - | - | - | - | - | - | - | - | - | - | - | - | - | - |
| PC-3p-54311-57 | G | A | A | A | T | G | - | - | A | G | T | G | C | G | T | T | T | A | A | C | G | T | T | A | A | T | T | T | T | T | T | T | T | - | - | - | A | C | T | G | A | C | C | - | - | - | - | - | - | - | - | A | G | G | - | - | A | A | A | G |
| PC-3p-54311-44 | G | A | A | A | T | G | - | - | A | G | T | G | C | G | T | T | T | A | A | C | G | T | T | C | A | A | T | T | T | T | T | T | T | T | - | T | A | C | T | G | A | G | T | - | - | - | - | - | - | - | - | G | C | A | - | - | A | A | A | T |
| PC-3p-54311-26 | A | A | A | A | T | G | - | - | A | G | T | T | T | G | T | T | A | A | C | A | G | - | - | C | A | A | T | T | T | T | T | T | T | T | - | T | A | C | T | G | A | G | T | - | - | - | - | - | - | - | - | A | C | A | A | A | G | T | A | C |
| PC-3p-54311-61 | G | A | A | A | T | G | - | - | A | G | T | G | C | G | T | T | T | A | A | C | G | T | C | A | A | A | T | T | T | T | T | T | T | T | - | - | A | C | T | G | A | G | C | - | - | - | - | - | - | - | - | A | G | T | T | G | T | T | T | C |
| PC-3p-54311-47 | A | A | A | A | T | G | - | - | A | G | T | G | C | G | T | T | T | A | A | C | G | T | A | A | A | A | T | T | T | T | T | T | T | T | - | T | A | C | T | G | G | G | C | - | - | - | - | - | - | - | - | A | T | T | A | T | T | A | A | A |
| PC-3p-54311-63 | T | C | G | G | T | - | - | - | - | - | A | G | C | A | A | T | T | T | G | - | G | T | C | T | C | A | T | A | A | A | A | A | A | T | - | G | A | G | A | G | G | T | G | - | - | - | - | - | - | - | - | A | A | A | A | A | T | T | G | G |
| PC-3p-54311-60 | G | A | A | A | T | G | - | - | A | G | T | G | C | G | T | T | T | A | A | C | G | T | C | A | A | A | T | C | A | A | C | A | C | T | - | G | A | G | T | G | - | T | G | - | - | - | - | - | - | - | - | T | T | A | T | A | T | T | A | T |
| PC-3p-54311-38 | G | A | A | A | T | G | - | - | A | A | T | G | C | G | T | T | T | A | G | C | G | T | C | A | A | T | T | T | T | T | T | T | T | T | - | C | A | C | T | G | A | G | T | - | - | - | - | - | - | - | - | T | C | A | T | T | T | T | T | T |
| PC-3p-54311-17 | G | G | A | A | T | A | - | - | A | G | T | A | C | T | T | T | T | A | A | C | G | T | C | A | A | T | T | T | T | T | T | T | T | - | - | T | A | C | T | G | A | G | C | - | - | - | - | - | - | - | - | A | C | A | T | A | C | T | A | T |
| PC-3p-54311-36 | G | A | A | A | T | G | - | - | A | G | T | A | C | T | T | T | T | A | A | C | G | T | C | A | A | T | T | T | T | T | T | T | T | - | - | T | A | C | T | G | A | G | T | - | - | - | - | - | - | - | - | A | G | A | T | - | - | T | G | T |
| PC-3p-54311-12 | G | A | A | A | T | G | - | - | A | G | T | G | C | G | T | T | T | A | A | C | G | T | C | A | A | A | T | T | T | T | T | T | T | A | - | T | A | A | T | G | A | G | C | - | - | - | - | - | - | - | - | A | T | T | G | - | A | C | G | A |
| PC-3p-54311-3 | A | A | A | A | T | G | - | - | A | C | T | A | C | T | T | T | T | T | A | C | G | T | C | A | A | T | T | T | T | T | T | T | T | - | - | C | A | C | T | G | A | G | T | - | - | - | - | - | - | - | - | - | - | T | T | A | T | T | A | T |
| PC-3p-54311-10 | G | A | A | A | G | G | - | - | A | C | T | A | C | T | T | T | T | T | A | C | G | T | C | A | G | T | T | T | T | T | T | T | T | - | - | C | A | C | T | G | A | A | C | - | - | - | - | - | - | - | - | - | - | C | T | A | C | T | A | G |
| PC-3p-54311-22 | G | A | A | A | T | A | - | - | A | G | T | A | C | T | T | T | T | A | A | C | G | T | T | A | A | T | T | T | T | T | T | T | T | - | - | - | A | C | T | G | A | G | T | - | - | - | - | - | - | - | - | - | - | A | T | C | T | A | A | A |
| PC-3p-54311-49 | G | A | A | G | T | G | - | - | A | C | T | A | C | T | T | T | T | T | A | C | G | T | C | A | A | T | T | T | T | T | T | T | T | T | T | C | A | C | T | G | A | G | A | - | - | - | - | - | - | - | - | - | - | G | T | C | T | A | A | A |
| PC-3p-54311-1 | G | A | A | A | T | G | - | - | A | C | T | A | C | T | T | C | T | T | A | C | G | T | C | A | A | T | T | T | T | T | T | T | T | T | - | C | A | C | T | G | A | G | T | - | - | - | - | - | - | - | - | A | C | A | T | A | T | T | G | C |
| PC-3p-54311-55 | A | A | A | A | T | G | - | - | A | C | T | A | C | T | T | T | T | T | A | C | G | T | C | A | G | - | T | T | T | T | T | T | T | T | - | C | A | C | T | G | A | G | T | - | - | - | - | - | - | - | - | A | T | T | T | A | T | T | A | A |
| PC-3p-54311-2 | G | A | A | A | T | G | - | - | A | G | T | A | C | T | T | T | T | A | A | C | G | T | C | A | A | T | T | T | T | T | T | T | T | T | - | C | A | C | T | G | A | G | T | - | - | - | - | - | - | - | - | A | T | G | T | A | A | A | A | A |
| PC-3p-54311-9 | G | A | A | A | T | G | - | - | A | C | T | A | C | T | T | T | T | A | A | C | G | T | C | A | A | T | T | T | T | T | T | T | T | T | - | C | A | C | T | G | A | G | T | - | - | - | - | - | - | - | - | A | T | A | T | T | C | G | T | A |
| PC-3p-54311-20 | G | A | A | A | T | G | - | - | A | C | T | A | C | T | T | T | T | T | A | C | G | T | C | A | A | T | T | T | T | T | T | T | T | T | - | C | A | C | T | G | A | G | T | - | - | - | - | - | - | - | - | G | T | G | T | T | T | A | T | A |
| PC-3p-54311-18 | G | G | A | A | T | G | - | - | A | C | T | A | C | T | T | T | T | T | A | C | G | T | C | A | A | T | T | T | T | T | T | T | T | - | - | C | A | C | T | G | A | G | T | - | A | C | G | A | T | C | T | A | T | T | T | T | C | T | G | G |
| PC-3p-54311-37 | G | A | A | A | T | G | - | - | A | C | T | A | C | T | T | C | T | T | A | C | G | T | C | A | A | T | T | T | T | T | T | T | T | T | - | C | A | C | A | G | A | G | T | G | A | C | T | A | T | T | A | A | A | A | T | A | A | G | G | A |
| PC-3p-54311-45 | G | A | A | A | T | G | - | - | A | C | T | A | C | G | T | T | T | T | A | C | G | T | C | A | A | T | T | T | T | T | T | T | T | - | - | C | A | C | T | G | A | G | T | - | G | C | A | A | A | C | T | A | A | A | T | G | A | T | - | - |
| PC-3p-54311-35 | G | A | A | A | T | G | A | A | A | G | T | A | G | T | C | A | T | T | T | C | G | A | C | A | A | T | T | T | T | T | T | T | T | - | - | C | A | C | T | G | A | G | T | - | - | - | - | - | - | - | - | A | T | T | A | C | T | A | - | - |
| PC-3p-54311-41 | G | A | A | A | T | G | - | - | A | G | T | A | A | T | T | T | T | T | A | C | G | T | C | A | A | T | T | T | T | G | T | T | T | - | - | C | A | C | T | G | A | G | T | - | - | - | - | - | - | - | - | A | T | G | A | A | A | A | G | T |
| PC-3p-54311-40 | G | A | A | A | T | G | - | - | A | C | T | A | C | T | T | T | T | T | A | T | G | T | C | A | A | T | T | T | T | T | T | T | T | - | - | C | A | C | T | G | A | G | T | - | - | - | - | - | - | - | - | A | G | A | C | A | A | A | G | A |
| PC-3p-54311-4 | G | A | A | A | T | G | - | - | A | C | T | A | C | T | T | T | T | T | A | C | G | T | C | A | A | T | T | T | T | - | T | T | T | T | G | - | A | C | T | G | A | G | T | - | - | - | - | - | - | - | - | G | C | C | A | A | T | T | T | T |
| PC-3p-54311-42 | G | A | A | A | T | G | - | - | A | C | T | A | C | T | T | T | T | T | A | C | G | T | C | A | A | T | T | T | T | - | T | T | T | T | T | C | A | C | T | G | A | G | T | - | - | - | - | - | - | - | - | G | C | T | T | A | A | T | T | T |
| PC-3p-54311-16 | G | A | A | A | T | G | - | - | A | C | T | A | C | T | T | T | T | T | A | C | G | T | C | A | T | T | T | T | T | T | T | T | T | T | T | C | A | C | T | G | A | G | T | - | - | - | - | - | - | - | - | G | A | T | T | T | C | A | A | T |
| PC-3p-54311-27 | G | A | A | A | T | G | - | - | A | C | T | A | C | T | T | T | T | T | A | C | G | T | C | A | A | T | T | T | T | T | T | T | T | T | - | C | A | C | T | G | A | G | T | - | - | - | - | - | - | - | - | A | C | A | T | A | C | G | C | T |
| PC-3p-54311-15 | G | A | A | G | T | G | - | - | A | C | T | A | C | T | T | T | T | T | A | C | G | T | C | A | A | T | T | T | T | T | T | T | T | T | C | T | A | C | T | G | A | G | C | - | - | - | - | - | - | - | - | T | C | G | T | - | - | - | A | T |
| PC-3p-54311-14 | G | A | A | A | T | G | - | - | A | C | T | A | C | T | T | T | T | T | A | C | G | T | C | A | A | T | T | T | T | T | T | T | - | - | - | C | A | C | T | G | A | G | C | A | G | - | - | - | - | - | - | T | G | A | A | A | T | T | G | A |
| PC-3p-54311-21 | G | A | A | A | T | G | - | - | A | C | T | A | C | T | T | T | T | T | A | C | G | T | C | A | A | T | T | T | T | T | T | T | T | - | - | C | A | C | T | G | A | G | T | A | A | - | - | - | - | - | - | T | G | A | A | A | T | T | G | T |
| PC-3p-54311-24 | G | A | A | A | T | G | - | - | A | C | T | A | C | T | T | T | T | T | A | C | G | G | C | A | A | T | T | T | T | T | T | T | T | - | - | C | A | A | T | G | A | G | T | A | - | - | - | - | - | - | - | A | T | A | T | A | T | T | C | A |
| PC-3p-54311-65 | A | A | A | A | T | G | - | - | A | C | T | A | C | T | T | T | T | T | A | C | G | T | C | A | A | T | T | T | T | T | T | T | T | - | - | C | A | C | T | G | A | G | T | A | G | - | - | - | - | - | - | C | T | G | T | T | T | A | A | T |
| PC-3p-54311-28 | G | A | A | A | T | G | - | - | A | C | T | A | C | T | T | T | T | T | A | C | G | T | C | A | A | T | T | T | C | T | T | T | T | - | - | C | A | C | T | G | A | G | T | G | G | - | - | - | - | - | - | C | G | A | T | G | A | A | A | T |
| PC-3p-54311-50 | G | A | A | A | T | G | - | - | A | C | T | A | C | T | T | T | T | T | A | C | G | T | C | A | A | T | T | T | T | T | T | T | T | - | - | C | A | C | T | G | A | G | T | A | A | - | - | - | - | - | - | C | - | - | - | - | - | A | C | T |
| PC-3p-54311-30 | G | A | A | A | T | G | - | - | A | C | T | A | C | T | T | T | T | T | A | C | G | T | C | A | A | T | T | T | T | T | T | T | T | T | - | C | A | C | T | G | A | G | T | - | - | - | - | - | - | - | - | - | G | T | G | A | T | A | G | T |
| PC-3p-54311-11 | G | A | A | A | T | G | - | - | A | C | T | A | C | T | T | T | T | T | A | C | G | T | A | A | A | T | T | T | T | T | T | T | T | - | - | C | A | C | T | A | T | G | T | - | - | - | - | - | - | - | - | - | - | G | C | T | T | T | A | T |
| PC-3p-54311-19 | G | A | A | A | T | G | - | - | A | C | T | A | C | T | T | T | T | T | A | C | G | T | C | A | A | T | T | A | T | T | T | T | T | - | - | C | A | C | T | G | A | G | T | - | - | - | - | - | - | - | - | - | A | T | A | T | A | T | A | T |
| PC-3p-54311-23 | G | A | A | A | T | G | - | - | A | C | T | A | C | T | T | T | T | T | A | C | G | T | C | A | A | T | T | T | T | T | T | T | C | - | - | C | A | C | T | G | A | G | T | - | - | - | - | - | - | - | - | T | A | T | A | A | T | T | A | C |
| PC-3p-54311-29 | G | A | A | A | T | G | - | - | A | C | T | A | C | T | C | T | T | T | A | C | G | T | C | A | A | T | T | T | T | T | T | T | T | - | - | C | A | C | T | G | A | G | T | - | - | - | - | - | - | - | - | A | T | C | A | T | A | A | G | C |
| PC-3p-54311-31 | G | A | A | A | T | G | - | - | A | C | T | A | C | T | T | T | T | T | A | C | G | T | C | A | A | T | T | T | T | T | T | T | - | - | - | C | A | C | T | G | A | G | T | - | - | - | - | - | - | - | - | A | C | T | A | C | G | T | T | G |
| PC-3p-54311-6 | G | A | A | A | T | G | - | - | A | A | T | T | T | G | T | T | A | C | A | T | G | T | T | G | T | A | T | A | G | T | G | A | A | - | - | C | A | T | T | - | - | - | - | - | - | - | - | - | - | - | - | T | T | T | C | T | G | T | G | C |
| PC-3p-54311-64 | G | A | A | A | T | G | - | - | A | G | T | T | T | G | T | T | C | A | A | A | G | C | C | T | C | T | T | T | T | C | A | C | T | G | A | A | T | A | T | G | G | A | T | - | - | - | - | - | - | - | - | A | T | T | C | A | A | T | A | A |

| PC-3p-54311-5 | A | T | T | T | T | C | C | A | A | A | A | A | - | A | T | G | A | A | A | G | A | A | A | A | A | A | A | A | T | T | T | T | T | T | T | G | G | C | A | A | A | A | T | - | A | T | T | G | A | C | C | G | A | T | T | T | C | T | A | G |
| --- | --- | --- | --- | --- | --- | --- | --- | --- | --- | --- | --- | --- | --- | --- | --- | --- | --- | --- | --- | --- | --- | --- | --- | --- | --- | --- | --- | --- | --- | --- | --- | --- | --- | --- | --- | --- | --- | --- | --- | --- | --- | --- | --- | --- | --- | --- | --- | --- | --- | --- | --- | --- | --- | --- | --- | --- | --- | --- | --- | --- |
| PC-3p-54311-46 | G | T | T | T | T | C | C | A | T | T | A | T | - | T | T | G | T | C | T | C | A | A | C | T | A | T | T | T | A | G | A | T | C | A | A | A | A | C | G | A | A | G | G | - | A | T | G | A | G | C | A | A | A | A | A | T | A | T | A | A |
| PC-3p-54311-7 | C | T | T | T | T | C | G | T | A | G | A | C | G | T | T | C | A | G | G | T | A | T | C | A | A | G | T | T | C | T | A | A | C | G | A | T | T | G | G | A | T | G | C | G | A | T | T | T | T | C | C | A | A | T | T | T | C | G | G | A |
| PC-3p-54311-39 | G | A | A | A | - | A | C | A | T | C | T | G | T | T | T | C | C | T | G | T | C | T | C | G | A | A | A | C | T | T | T | T | A | A | A | A | A | T | A | A | C | A | A | G | A | T | - | C | G | G | C | A | C | G | T | T | T | C | - | - |
| PC-3p-54311-66 | T | A | T | A | G | A | A | A | T | T | T | A | T | A | T | T | C | A | T | T | C | A | T | C | A | T | C | C | A | C | C | G | C | G | T | A | C | G | T | G | T | T | T | T | T | T | - | G | T | G | A | A | T | G | A | A | G | G | - | - |
| PC-3p-54311-51 | T | A | T | A | A | C | T | A | A | G | T | A | A | A | A | T | A | G | - | A | C | T | A | C | T | T | T | T | G | T | T | T | A | A | A | A | A | T | T | T | G | A | T | G | A | T | - | T | T | G | G | - | - | G | A | A | A | C | - | - |
| PC-3p-54311-59 | G | G | A | G | G | G | T | A | A | A | A | T | A | G | G | T | T | C | A | A | A | T | A | C | C | A | T | A | A | T | G | A | A | C | C | A | A | T | A | G | C | A | A | A | C | C | - | G | T | C | C | A | C | A | A | T | C | C | - | - |
| PC-3p-54311-43 | T | A | T | T | T | G | G | T | T | T | G | A | T | T | T | T | A | A | T | G | T | A | A | C | A | T | T | A | A | T | A | A | T | G | T | T | T | A | T | G | A | T | - | - | - | - | - | - | - | - | - | - | - | - | - | - | - | - | - | - |
| PC-3p-54311-48 | T | A | T | A | T | C | G | C | T | T | A | A | A | A | T | T | A | T | T | G | A | T | A | A | T | T | T | G | C | T | T | T | T | G | T | T | T | T | T | C | A | G | C | G | C | T | C | A | T | G | C | C | C | G | G | C | G | A | - | - |
| PC-3p-54311-53 | G | C | T | A | A | C | C | A | A | G | C | A | A | T | T | C | A | T | T | G | T | T | C | A | C | A | G | C | A | T | C | T | A | G | T | G | A | T | A | A | T | A | T | G | T | G | A | A | T | A | A | T | T | A | A | G | A | - | - | - |
| PC-3p-54311-62 | G | C | T | A | A | C | C | A | A | G | C | A | A | T | C | C | A | T | T | G | T | T | C | A | A | A | G | C | A | T | C | T | A | G | T | G | A | T | G | A | T | A | T | G | T | G | A | A | T | G | A | T | T | - | - | - | - | - | - | - |
| PC-3p-54311-58 | T | T | T | G | T | A | A | C | T | T | C | G | T | G | C | T | T | A | T | G | T | T | A | C | A | A | A | T | G | A | G | T | T | C | C | A | C | A | T | A | A | C | C | A | T | T | T | T | G | T | A | A | C | T | T | T | - | - | - | - |
| PC-3p-54311-52 | T | T | T | G | A | A | C | T | T | T | T | T | C | G | T | T | C | G | G | T | G | C | T | G | C | T | A | C | A | C | T | C | C | A | T | T | C | T | C | C | A | A | T | A | A | A | T | A | T | A | G | T | A | A | - | - | - | - | - | - |
| PC-3p-54311-56 | G | T | T | G | A | A | G | G | A | A | A | T | C | A | T | T | T | A | A | A | A | A | T | G | A | A | C | G | A | T | T | C | C | A | G | T | C | A | T | T | A | A | A | G | A | A | A | G | C | A | A | A | C | - | - | - | - | - | - | - |
| PC-3p-54311-13 | A | T | G | A | T | A | G | G | T | C | G | A | A | T | T | - | T | T | T | C | G | C | T | T | T | T | A | A | T | A | T | T | T | T | C | C | G | T | T | T | C | A | T | T | C | A | T | T | T | T | T | T | T | A | A | T | C | C | C | - |
| PC-3p-54311-33 | G | T | G | A | G | T | G | A | T | T | G | A | A | A | T | - | A | A | T | C | A | A | A | T | T | T | C | A | A | T | C | A | T | T | C | A | G | A | A | A | A | A | G | - | - | A | G | T | G | A | T | T | A | G | A | A | C | A | T | - |
| PC-3p-54311-25 | A | T | T | A | T | A | C | A | T | T | T | C | G | A | T | G | C | T | T | C | A | T | T | T | A | T | C | T | T | T | T | T | T | T | C | G | G | C | A | A | C | A | A | - | C | A | A | A | A | A | G | G | A | G | A | - | - | - | - | - |
| PC-3p-54311-34 | - | - | - | - | - | - | - | - | - | - | - | - | - | - | - | - | - | - | - | - | - | - | - | - | - | - | - | - | - | - | - | - | - | - | - | - | - | - | - | - | - | - | - | - | - | - | - | - | - | - | - | - | - | - | - | - | - | - | - | - |
| PC-3p-54311-54 | G | - | A | A | A | C | A | T | A | T | A | G | G | A | C | A | A | - | - | T | A | T | G | C | G | A | A | T | A | T | A | T | A | A | A | A | T | T | T | T | C | T | C | T | G | T | C | T | T | C | A | T | T | G | G | T | T | - | - | - |
| PC-3p-54311-8 | A | T | T | A | A | A | C | A | A | T | T | G | A | A | T | A | G | - | - | T | A | T | G | - | G | C | A | A | C | C | T | C | C | T | A | A | T | T | T | G | C | A | T | G | A | A | T | A | T | C | T | A | T | C | T | C | - | - | - | - |
| PC-3p-54311-32 | - | - | - | - | - | - | - | - | - | - | - | - | - | - | - | - | - | - | - | - | - | - | - | - | - | - | - | - | - | - | - | - | - | - | - | - | - | - | - | - | - | - | - | - | - | - | - | - | - | - | - | - | - | - | - | - | - | - | - | - |
| PC-3p-54311-57 | G | T | C | C | C | C | G | A | C | T | A | G | G | A | A | T | G | T | T | T | T | T | T | T | T | C | A | A | A | A | T | C | G | T | T | A | T | T | G | T | C | A | T | C | G | T | G | T | T | G | G | G | C | A | T | C | A | - | - | - |
| PC-3p-54311-44 | T | G | A | A | A | A | G | T | T | T | G | T | G | A | T | A | G | - | - | - | - | T | G | C | T | T | G | A | G | T | C | A | T | T | C | A | A | T | G | T | C | A | G | A | G | A | A | T | A | C | G | T | T | A | T | C | A | G | A | - |
| PC-3p-54311-26 | T | - | A | C | A | T | A | C | C | T | C | G | A | C | T | T | G | - | - | C | T | G | T | C | G | T | A | G | A | G | C | C | A | A | C | A | T | G | A | C | C | A | A | A | T | A | A | A | T | T | T | G | T | A | T | T | T | G | - | - |
| PC-3p-54311-61 | T | - | T | T | G | A | G | C | A | G | C | G | A | T | T | T | C | - | - | C | A | A | T | C | T | A | T | T | G | A | C | G | A | G | A | A | A | A | G | A | A | A | T | T | T | C | G | A | A | A | G | A | A | A | A | A | T | - | - | - |
| PC-3p-54311-47 | A | T | T | C | C | A | A | A | T | T | G | A | A | T | T | A | G | G | C | G | C | A | G | G | C | A | T | G | T | A | C | C | C | C | T | G | T | C | G | A | A | A | A | A | A | C | A | G | C | A | T | A | T | - | - | - | - | - | - | - |
| PC-3p-54311-63 | G | T | G | - | - | A | A | C | A | T | T | A | T | T | T | G | A | A | C | T | C | T | T | T | T | T | T | T | T | A | C | G | A | T | T | A | T | A | G | G | T | G | A | A | G | A | A | T | T | A | T | A | G | T | T | A | A | A | - | - |
| PC-3p-54311-60 | G | T | T | T | T | A | T | A | T | T | T | T | A | T | T | C | A | A | C | A | A | A | A | G | A | T | A | T | A | A | T | C | A | T | G | A | C | A | A | A | T | T | T | T | T | C | T | G | A | A | G | A | C | C | - | - | - | - | - | - |
| PC-3p-54311-38 | C | A | A | T | A | A | A | A | C | T | T | C | A | T | A | A | T | T | A | A | T | T | T | T | T | C | A | A | A | A | A | A | A | T | T | A | T | C | G | A | A | C | G | T | T | C | G | C | T | T | C | C | C | - | - | - | - | - | - | - |
| PC-3p-54311-17 | C | A | A | A | G | A | T | G | T | A | A | T | C | A | T | C | T | T | A | T | T | G | A | A | T | G | C | T | G | T | T | T | G | G | T | T | A | A | A | T | T | T | A | A | G | T | G | - | A | G | T | C | A | A | T | - | - | - | - | - |
| PC-3p-54311-36 | T | G | A | T | A | T | T | C | C | G | T | C | C | A | G | C | G | A | A | T | T | G | T | G | A | A | A | A | G | T | T | T | T | T | T | T | T | A | T | T | T | A | A | T | A | T | G | G | A | G | T | G | A | - | - | - | - | - | - | - |
| PC-3p-54311-12 | A | T | T | T | T | C | T | G | G | A | A | A | A | A | G | C | A | C | A | A | T | - | C | G | C | A | A | T | G | T | A | G | A | A | A | G | G | A | T | T | T | C | A | T | T | G | T | C | T | G | T | A | A | G | G | - | - | - | - | - |
| PC-3p-54311-3 | C | C | G | A | T | T | - | A | A | A | A | A | C | C | G | T | C | A | A | A | A | A | C | A | T | T | C | A | A | A | A | T | T | C | T | T | A | T | A | A | A | A | T | C | C | A | T | A | A | T | T | T | A | A | A | A | A | - | - | - |
| PC-3p-54311-10 | C | C | T | A | C | T | - | C | A | C | T | C | G | T | C | T | T | G | A | G | C | A | A | A | A | T | T | A | C | C | C | T | A | C | T | - | A | C | T | T | A | C | T | C | A | A | C | C | T | A | T | T | A | G | A | T | C | - | - | - |
| PC-3p-54311-22 | T | A | T | A | T | A | A | A | A | A | T | C | A | C | G | T | G | A | T | G | T | T | C | G | T | A | A | G | T | A | T | T | T | T | A | T | A | C | A | T | T | C | C | C | A | A | A | C | T | A | T | C | A | A | A | C | C | - | - | - |
| PC-3p-54311-49 | T | T | A | A | T | - | - | A | A | A | T | C | A | A | T | T | - | T | T | G | T | C | T | A | T | T | A | T | C | C | C | T | T | T | G | C | A | G | A | C | G | T | T | C | A | A | A | T | C | T | G | C | A | G | A | A | C | - | - | - |
| PC-3p-54311-1 | C | - | T | T | G | T | A | C | A | A | A | - | A | A | A | G | A | A | T | A | T | A | A | A | T | T | G | A | A | C | T | C | A | C | C | A | G | G | A | A | A | T | T | A | C | T | C | T | C | A | G | T | T | T | G | - | - | - | - | - |
| PC-3p-54311-55 | A | G | A | T | A | T | A | G | A | G | A | G | A | A | T | G | G | A | G | T | C | G | C | T | T | A | G | G | A | C | C | T | G | G | G | A | C | G | C | A | A | T | G | A | G | A | A | A | T | G | A | T | T | T | - | - | - | - | - | - |
| PC-3p-54311-2 | G | A | A | T | A | T | A | T | G | A | T | T | T | T | T | T | A | A | C | C | A | A | C | C | T | G | T | T | G | A | T | A | T | C | G | A | C | - | A | A | C | G | C | A | C | A | C | A | C | G | A | A | A | A | - | - | - | - | - | - |
| PC-3p-54311-9 | A | - | - | - | A | G | A | G | G | G | A | G | A | C | G | G | G | G | C | C | G | A | A | C | T | T | T | T | G | G | C | A | T | C | G | G | G | A | T | A | T | G | G | G | G | A | G | A | T | A | T | C | A | T | A | A | - | - | - | - |
| PC-3p-54311-20 | A | - | - | - | T | G | A | A | G | T | A | A | A | A | T | A | A | A | T | C | G | T | A | T | T | T | G | A | A | A | T | A | T | A | G | A | A | C | T | A | G | A | A | T | C | A | A | C | T | G | T | T | T | A | A | C | - | - | - | - |
| PC-3p-54311-18 | A | C | A | T | T | T | A | T | C | G | G | C | T | C | A | A | A | T | G | T | G | A | A | T | T | G | A | T | - | T | A | G | T | T | - | - | C | T | C | A | G | A | G | G | A | T | G | A | T | T | - | - | - | - | - | - | - | - | - | - |
| PC-3p-54311-37 | C | C | G | G | A | G | A | A | C | G | G | T | A | G | A | A | A | T | A | T | G | T | A | T | T | C | A | T | - | T | C | G | T | T | - | - | A | T | T | C | G | T | G | T | A | A | A | C | T | T | T | C | T | A | T | G | C | A | T | G |
| PC-3p-54311-45 | T | T | G | A | T | A | A | C | C | G | G | T | G | G | T | T | A | A | G | T | G | G | T | T | T | C | A | T | C | T | C | G | T | T | T | C | A | T | T | A | A | T | G | C | A | A | A | C | T | - | - | - | - | - | - | - | - | - | - | - |
| PC-3p-54311-35 | T | C | A | T | T | A | C | T | T | A | A | C | T | T | A | T | A | T | T | T | T | G | A | T | G | C | A | T | - | A | C | A | A | G | - | G | G | G | A | A | T | A | A | T | A | G | A | A | T | T | G | A | A | A | T | G | - | - | - | - |
| PC-3p-54311-41 | A | C | C | A | A | A | T | A | T | T | G | C | T | T | G | - | C | T | G | G | T | A | T | T | A | C | T | G | - | C | G | G | T | G | - | C | T | A | A | A | T | A | G | C | A | G | A | C | T | A | T | T | A | G | T | A | C | - | - | - |
| PC-3p-54311-40 | C | C | A | G | T | G | T | T | T | G | T | T | C | T | A | C | A | T | T | T | G | G | C | T | G | C | A | T | - | C | C | G | C | T | - | T | T | T | A | A | G | G | A | T | A | A | G | T | T | G | A | C | T | A | T | T | - | - | - | - |
| PC-3p-54311-4 | T | T | T | A | A | T | T | T | A | A | A | T | T | T | T | T | T | G | G | A | A | A | T | G | G | A | T | G | A | T | G | T | C | G | T | - | - | - | A | A | T | T | A | T | G | T | C | C | G | A | A | T | T | A | C | G | T | - | - | - |
| PC-3p-54311-42 | T | C | T | C | T | - | - | - | - | C | A | T | T | G | T | A | T | G | G | G | T | A | T | T | T | T | C | A | C | A | A | T | C | A | T | T | T | C | A | A | T | T | T | G | T | T | C | A | G | A | G | T | T | T | C | A | T | - | - | - |
| PC-3p-54311-16 | G | A | T | A | C | G | - | T | T | C | A | A | T | G | G | G | A | C | T | T | C | T | T | T | T | T | C | A | G | C | G | A | T | G | A | T | C | T | T | G | - | T | G | C | A | T | C | A | A | A | T | T | A | C | - | - | - | - | - | - |
| PC-3p-54311-27 | G | C | C | A | T | T | - | T | T | A | A | C | T | G | - | - | C | T | T | T | C | A | G | A | A | T | C | A | G | - | G | A | T | - | - | T | C | T | G | G | C | T | T | T | A | T | C | A | A | A | T | T | C | C | C | G | T | T | T | - |
| PC-3p-54311-15 | G | A | G | T | G | G | - | T | C | G | A | C | T | A | T | C | C | A | C | A | G | G | T | T | A | T | T | G | G | G | G | G | T | G | G | A | T | T | A | G | - | - | C | T | A | T | G | C | G | G | G | T | C | C | T | G | G | - | - | - |
| PC-3p-54311-14 | G | T | A | A | A | T | T | C | A | A | G | - | T | T | C | A | A | T | T | G | A | A | G | T | A | T | C | G | G | T | T | G | A | A | G | - | C | T | C | T | G | G | T | T | C | A | A | C | T | T | C | G | A | T | T | - | - | - | - | - |
[truncated: 99,102 more chars]
