# Supplementary material for: Deep sequencing and genome-wide analysis reveals the expansion of MicroRNA genes in the gall midge Mayetiola destructor
Source: BMC Genomics. 2013 Mar 18;14:187. doi: 10.1186/1471-2164-14-187 (PMC3608969; doi:10.1186/1471-2164-14-187)
Supplement: Additional file 7: Figure S5 — qPCR validation of microarray data and further analysis of miRNA levels in other developmental stages of Hessian fly. Samples were collected from several life stages of Hessian fly reared on Newton plants, as well as, from larvae reared on Newton and Molly plants. The expression of the U6 small nuclear RNA gene was used as an internal control. Three biological replications and two technical replications were used in this analysis. The bars with and asterik (*) were used for qPCR and microarray data comparison. Egg 1 and 3: one and three days old eggs. ML1 and ML3: larvae collected after one and three day of incompatible interaction (reared on Molly). NL1, NL3, and NL8: larvae collected after one, three, and eight days of compatible interaction (reared on Newton). [file 1471-2164-14-187-S7.ppt]

## Slide 1
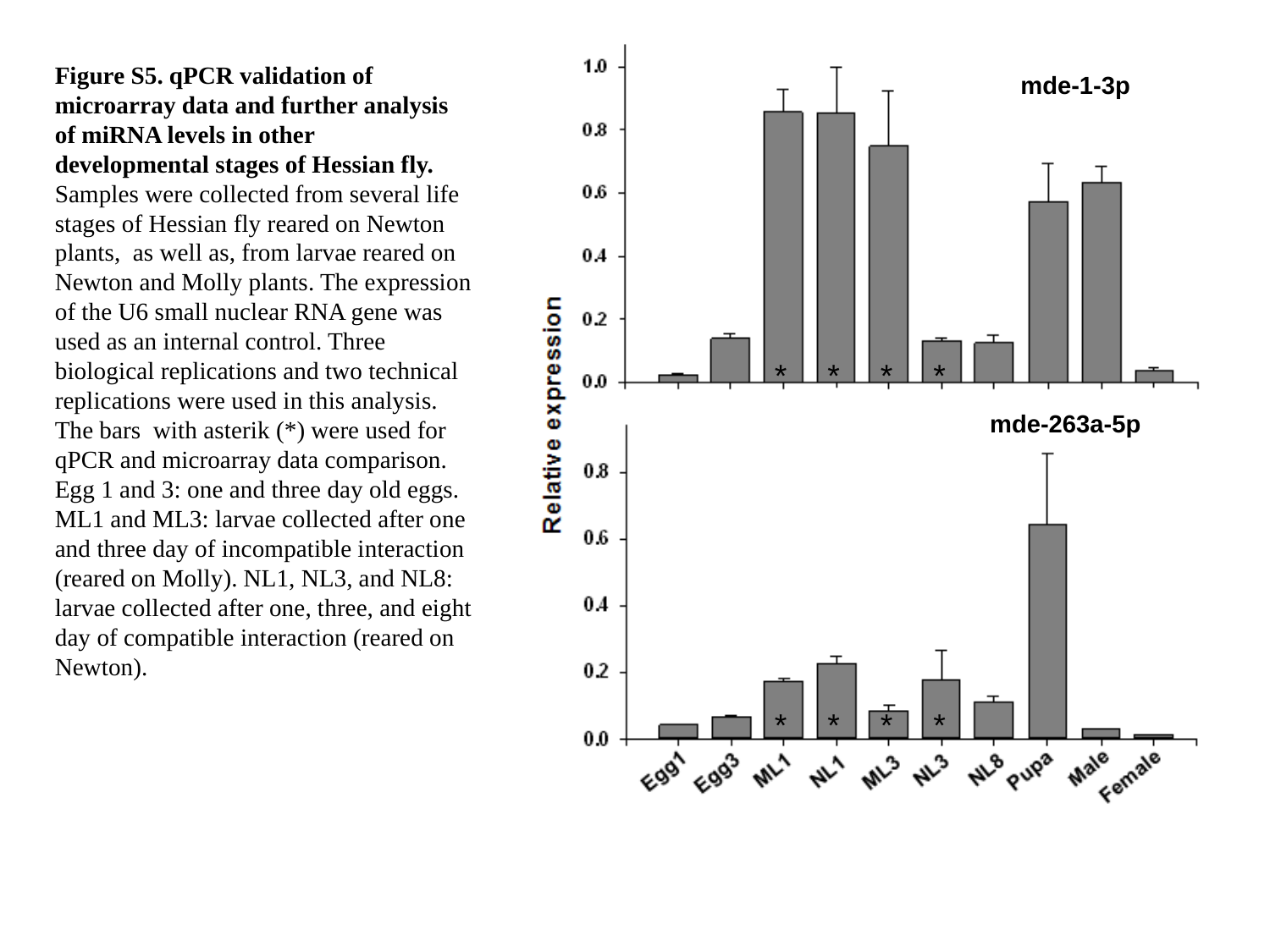

Figure S5. qPCR validation of microarray data and further analysis of miRNA levels in other developmental stages of Hessian fly. Samples were collected from several life stages of Hessian fly reared on Newton plants, as well as, from larvae reared on Newton and Molly plants. The expression of the U6 small nuclear RNA gene was used as an internal control. Three biological replications and two technical replications were used in this analysis. The bars with asterik (*) were used for qPCR and microarray data comparison. Egg 1 and 3: one and three day old eggs. ML1 and ML3: larvae collected after one and three day of incompatible interaction (reared on Molly). NL1, NL3, and NL8: larvae collected after one, three, and eight day of compatible interaction (reared on Newton).
mde-1-3p
*
*
*
*
mde-263a-5p
*
*
*
*
